# *E*‐Selective Radical Difunctionalization of Unactivated Alkynes: Preparation of Functionalized Allyl Alcohols from Aliphatic Alkynes

**DOI:** 10.1002/advs.202309022

**Published:** 2024-02-13

**Authors:** Jie Wang, Xinxin Wu, Zhu Cao, Xu Zhang, Xinxin Wang, Jie Li, Chen Zhu

**Affiliations:** ^1^ Key Laboratory of Organic Synthesis of Jiangsu Province College of Chemistry Chemical Engineering and Materials Science Soochow University 199 Ren‐Ai Road Suzhou Jiangsu 215123 China; ^2^ Frontiers Science Center for Transformative Molecules and Shanghai Key Laboratory for Molecular Engineering of Chiral Drugs Shanghai Jiao Tong University 800 Dongchuan Road Shanghai 200240 China

**Keywords:** aliphatic alkynes, allyl alcohols, functional group migration, photoredox, radical reactions

## Abstract

Radical difunctionalization of aliphatic alkynes provides direct access to valuable multi‐substituted alkenes, but achieving a high level of chemo‐ and stereo‐control remains a formidable challenge. Herein a novel photoredox neutral alkyne di‐functionalization is reported through functional group migration followed by a radical‐polar crossover and energy transfer‐enabled stereoconvergent isomerization of alkenes. In this sequence, a hydroxyalkyl and an aryl group are incorporated concomitantly into an alkyne, leading to diversely functionalized E‐allyl alcohols. The scope of alkynes is noteworthy, and the reaction tolerates aliphatic alkynes containing hydrogen donating C─H bonds that are prone to intramolecular hydrogen atom transfer. The protocol features broad functional group compatibility, high product diversity, and exclusive chemo‐ and stereoselectivity, thus providing a practical strategy for the elusive radical di‐functionalization of unactivated alkynes.

## Graphical abstract

An ingenious photocatalytic method is developed for the radical difunctionalization of unactivated alkynes, by which hydroxyalkyl and (hetero)aryl groups can be incorporated into alkynes in a reaction that stereoselectively delivers densely functionalized *E*‐allylic alcohols. The method offers broad functional group compatibility, high product diversity, and unique chemical and stereoselectivity, thus providing a practical strategy for the elusive radical difunctionalization of unactivated alkynes.

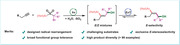

## 1 Introduction

**Figure 1:**
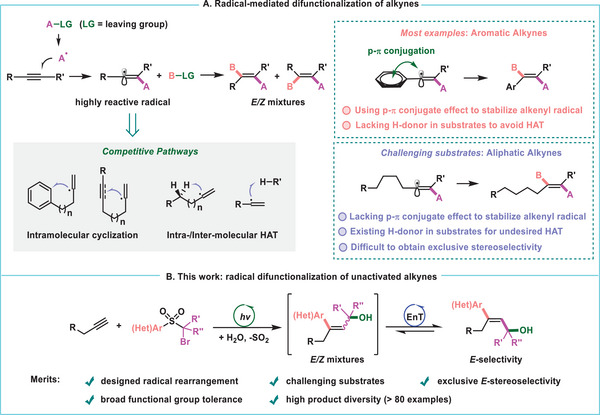
Radical‐mediated difunctionalization of alkynes.

Direct transformation of unsaturated carbon‐carbon bonds in alkenes or alkynes into valuable molecular skeletons is important because it has high synthetic value and uses broadly available raw materials. In recent decades, research into radical‐mediated difunctionalization of alkenes has seen significant progress,^[^ ^1^ ^]^ but radical difunctionalization of alkynes, which can generate diversely multi‐substituted alkenes, has lagged far behind.^[^ ^2^ ^]^ This can be ascribed to widely recognized kinetic and thermodynamic obstacles which include (**Figure** **1**A): 1) radical addition to alkynes generating highly reactive vinyl radicals that usually are subject to undesired rapid cyclization or addition to other π systems;^[^ ^3^ ^]^ 2) vinyl radicals engaging in fast hydrogen abstraction with rate coefficients of > 10^5^ M^−1^ s^−1^ for intramolecular 1,5‐hydrogen atom transfer (HAT)^[^ ^4^ ^]^ and ≈10^6^ M^−1^ s^−1^ for intermolecular HAT.^[^ ^5^ ^]^ As a result, the currently available approaches are largely dependent on the conversion of activated alkynes, such as phenylacetylene derivatives in which vinyl radicals are stabilized by a p‐π conjugate effect. Unactivated aliphatic alkynes lacking such p‐π conjugation however remain challenging substrates. Moreover, free radical‐mediated alkyne di‐functionalization without transition‐metal catalysis often leads to alkene products as *Z/E* mixtures,^[^ ^6^ ^]^ compromising the synthetic value of the method. Though a few reports achieve single stereoisomeric products, the most use of aryl alkynes limits product diversity and can not be applied to more common alkynes.^[^ ^7^ ^]^ Therefore, strategic innovation to convert unactivated alkynes to functional alkenes with superior chemo‐ or stereoselectivity is highly desirable but has not been achieved.

Radical rearrangement has been shown to be a pre‐eminent synthetic tactic for the transformation of unsaturated carbon‐carbon bonds,^[^ ^8^ ^]^ and may support the di‐functionalization of unactivated alkynes. Taking advantage of radical rearrangement we report a proof‐of‐concept study, in which aliphatic alkynes can be transformed stereoselectively into valuable multisubstituted *E*‐allyl alcohols (Figure 1B). This photoredox neutral cascade proceeds through sequential radical migration, radical‐polar crossover, and energy transfer (ET)‐promoted stereoconvergent alkene isomerization. Density functional theory (DFT) calculations have been carried out to rationalize the unique selectivity of functional group migration beyond the alkenyl radical‐induced 1,5‐HAT that probably occurs with aliphatic alkynes.

## 2 Results and Discussion

### 2.1 Optimization Reaction Conditions

At the outset, di‐functionalization of the aliphatic alkyne (**1a**) was investigated (**Table** **1**). This alkyne (**1a**) is one of the most challenging substrates for radical‐mediated alkyne difunctionalization and has never been used in such transformations. The existing *O*‐benzylic C─H bonds with relatively low bond dissociation energy (BDE = 79.8 kcal mol^−1^) could serve as a hydrogen donor in a 1,5‐HAT and could affect the functionalization of an alkenyl radical, leading to undesired hydrofunctionalization products. In fact, DFT calculations indicated that the alkenyl radical intermediate has a strong tendency (Δ*G* = ‐23 kcal mol^−1^) to abstract an H‐atom from the benzylic site. The reaction of **1a** with a sulfone (**2a**) using *fac*‐Ir(ppy)_3_ as photosensitizer and acetone/H_2_O as co‐solvent under green light (510 nm wavelength) irradiation led to a good yield of the allylic alcohol (**3a**) with exclusive *E*‐configuration (*E*/*Z* > 20:1, entry 1). A brief survey of reaction parameters in terms of photocatalyst, solvent, additive, and light source was carried out (for details, see the Supporting Information). Replacement of the photocatalyst by other catalysts did not improve the reaction outcome (entries 2–6). Sodium acetate was used to neutralize the hydrobromic acid that was generated in the reaction, and a decreased yield was obtained in the absence of this base (entry 7). Control experiments showed that photocatalyst and light were crucial to the transformation (entries 8–9), and the hydroxyl group in **3a** was derived from water. The amount of water in the cosolvent appears to influence the reaction (entries 11–12). Reducing the reaction temperature slowed down the reaction rate and sharply decreased the yield (entry 13).

**Table 1:** Optimized reaction conditions.

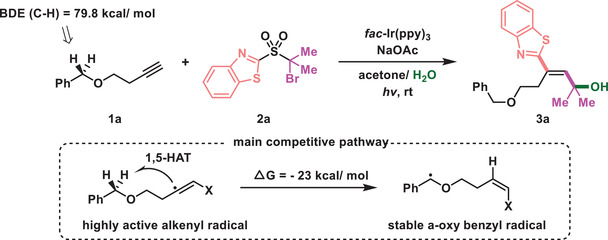			
Entry	Variation	Yield (%)^a)^	E/Z
1	none	78	>20:1
2	[lr(dF(CF_3_)_2_ ppy)_2_(dtbbpy)]PF_6_ as photocat.	0	‐
3	[lr(dtbbpy)(ppy)_2_]PF_6_ as photocat.	10	>20:1
4	Ru(bpy)_3_Cl_2_ 6H_2_O as photocat.	0	‐
5	4CzIPN as photocat.	0	‐
6	Eosin Y as photocat.	<5	>20:1
7	no base	44	>20:1
8	no photocat.	0	‐
9	no light	0	‐
10	no water	0	‐
11	0.1ml water	47	>20:1
12	1.0ml water	35	3:1
13^b)^	none	12	>20:1

**1a** (0.4 mmol), **2a** (0.2 mmol), *fac*‐Ir(ppy)_3_ (1 mol%) and NaOAc (0.1 mmol) in acetone/H_2_O (2 mL/0.6 mL), irradiated with 30 W 510 nm green LED (5 cm away from the light) at rt under N_2_ for 72 h. LED = light emitting diode

^a)^ Yields of isolated products are given

^b)^ 0 °C.

### 2.2 Substrate Scope

**Figure 2:**
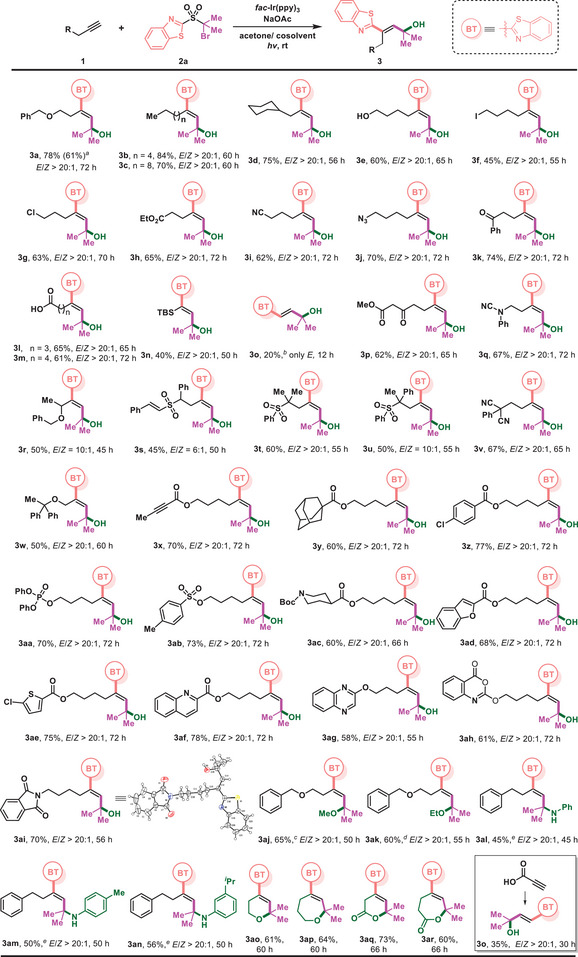
Scope of aliphatic alkynes. Reaction conditions: 1 (0.4 mmol), 2a (0.2 mmol), *fac*‐Ir(ppy)_3_ (1 mol%), and NaOAc (0.1 mmol) in acetone/H_2_O (2/0.6 mL), irradiated with 30 W 510 nm green LED (5 cm away from the light) at rt under N_2_. ^[a]^Scaled‐up preparation with 1a (4 mmol) and 2a (2 mmol). ^[b]^2a (0.2 mmol), *fac*‐Ir(ppy)_3_ (1 mol%) and NaOAc (0.1 mmol) in acetone/H_2_O (2/0.6 mL), irradiated with 50 W blue LED at rt with an acetylene balloon (1 atm) for 12 h. ^[c]^KH_2_PO_4_ (0.4 mmol), MeCN/MeOH (2/0.5 mL). ^[d]^KH_2_PO_4_ (0.4 mmol), MeCN/EtOH (2/0.5 mL). ^[e]^Aromatic amine (0.4 mmol), 2,6‐lutidine (0.4 mmol), MeCN (2 mL).

With the optimized reaction conditions in hand, the scope of unactivated alkynes was examined (**Figure** **2**). The reaction has broad functional group compatibility and a plethora of aliphatic alkynes proved to be suitable substrates, delivering synthetically useful yields with good stereoselectivity. The preparation of **3a** could be scaled up, giving a slightly decreased but acceptable yield. A diversity of susceptible groups, such as an unprotected alcohol (**3e**), iodide (**3f**), azide (**3j**), carboxylic acid (**3l**, **3m**) or silane (**3n**), remained intact in the reaction. Notably, acetylene gas was also suitable for this reaction to afford the corresponding *E*‐allylic alcohol (**3o**). A cyanoamide (**3q**) that could act as a radical acceptor via 5‐exo‐dig cyclization did not interfere with the desired difunctionalization.^[^ ^9^ ^]^ Though phenylsulfonyl^[^ ^10^ ^]^ alkenylsulfonyl^[^ ^11^ ^]^ and malonitrile^[^ ^12^ ^]^ moieties all possess some migratory aptitude, the competitive migration of those groups did not occur in the cases of **3s‐3v**, indicating that the migration of the benzothiazolyl group was faster. The reaction proceeded selectively at a terminal alkyne in the presence of an additional internal alkyne (**3x**). Alkyl carboxylate, phosphonate or sulfonate esters as substrates afforded the corresponding products (**3y‐3ab**) with comparable yields. Substrates containing various heterocyclic fragments including piperidyl (**3ac**), benzofuryl (**3ad**), thienyl (**3ae**), quinolyl (**3af**), quinoxalinol (**3ag**), or isatoic anhydride (**3ah**) were also compatible with the reaction conditions. The *E*‐configuration of the alkene in the product was unambiguously confirmed by the crystal structure of **3ai** (see Supporting Information).^[^ ^13^ ^]^ Using alcohols or aromatic amines as a nucleophilic solvent instead of water resulted in the corresponding allylic ethers (**3aj**, **3ak**) or allylic amines (**3al**‐**3an**). Remarkably, the method could be used to synthesize valuable cyclic compounds in one step. For instance, the reaction of alkynols under the current conditions led to 2H‐pyran (**3ao**) or oxepene (**3ap**) by intramolecular cyclization. Moreover, the reaction of alkynoic acids gave rise to δ‐pentenolide (**3aq**) and ε‐caprolactone (**3ar**), following a reaction in which the carboxylic acid served as a nucleophile. In addition to electron‐rich aliphatic alkynes, electron‐deficient propionic acid was also amenable to the reaction, albeit with a lower yield. The conversion afforded the decarboxylative product (**3o**), the formal difunctionalization of acetylene.

**Figure 3:**
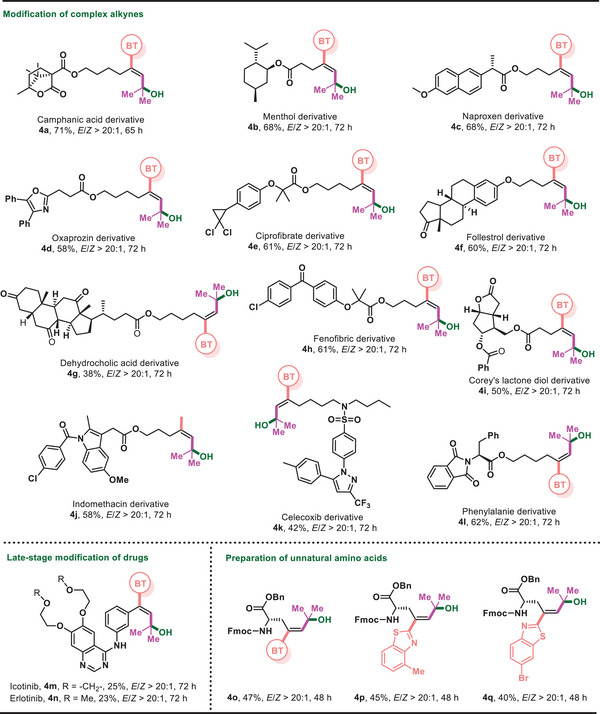
Modification of complex structures of natural products and drug derivatives. Reaction conditions: 1 (0.4 mmol), 2a (0.2 mmol), *fac*‐Ir(ppy)_3_ (1 mol%), and NaOAc (0.1 mmol) in acetone/H_2_O (2/0.6 mL), irradiated with 30 W 510 nm green LED (5 cm away from the light) at rt under N_2_.

The utility of this approach was further illustrated by the modification of complex alkynes (**Figure** **3**). A portfolio of complex molecules based on diverse structural features, such as *N*‐heteroaryl moieties (**4d**, **4j**, **4k**), lactones (**4a**, **4i**), dichlorocyclopropane (**4e**), sulfonamide (**4k**), *α*‐hydroxy acid (**4e**), and *α*‐amino acid (**4l**), were readily converted to the corresponding products with excellent stereocontrol. The chiral centers adjacent to the carbonyl group are sensitive to strong basic or acidic conditions but remain intact under the mild reaction conditions (**4c**, **4l**). The method could be directly applied to the late‐stage modification of Icotinib and Erlotinib (**4m**, **4n**), two commercially available drug molecules. Moreover, a set of unnatural amino acids containing multi‐substituted alkene was also furnished by this method (**4o**‐**4q**).

**Figure 4:**
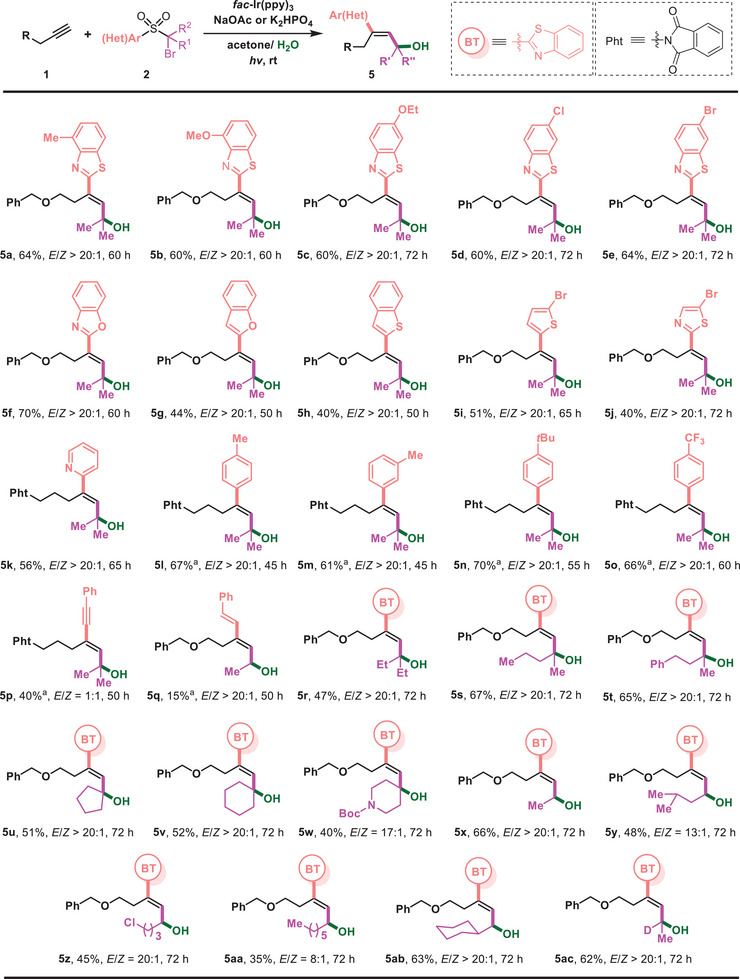
Variation of bifunctional sulfone reagents. Reaction conditions: 1 (0.4 mmol), 2 (0.2 mmol), *fac*‐Ir(ppy)_3_ (1 mol%) and NaOAc (0.1 mmol) in acetone/H_2_O (2/0.6 mL), irradiated with 30 W 510 nm green LED (5 cm away from the light) at rt under N_2_. ^[a]^K_2_HPO_4_ (0.4 mmol), irradiated with 456 nm Kessil LED light.

The diversity of products from this reaction could be enriched by varying the readily accessible (hetero)aryl sulfones (**2**) (**Figure** **4**). The electronic properties of substituents on the benzothiazolyl group had little impact on the reaction outcomes, and products (**5a‐5e**) were delivered in comparable yields. In addition, compounds with other heteroaryls such as benzoxazolyl (**5f**), benzofuryl (**5** **g**), benzothienyl (**5** **h**), thienyl (**5i**), thiazolyl (**5j**), and pyridyl (**5k**) could be incorporated into an alkyne, leading to the corresponding allylic alcohols in useful yields. Though the migration of aryl groups is much slower than that of heteroaryls and in fact failed on many occasions, cinnamyl alcohols (**5l**‐**5o**) were readily obtained in this reaction via phenyl migration. Notably, the reaction with alkynyl or alkenyl‐substituted sulfones resulted in a conjugated 1,3‐enynyl alcohol (**5p**) and a 1,3‐dienyl alcohol (**5q**) as a result of alkynyl or alkenyl migration. The alkyl substituent of sulfones was subsequently examined and it was found that both linear and cyclic alkyl moieties such as cyclopentyl, cyclohexyl, and piperidyl could be introduced to the alkyne, forming 1,1‐dialkyl‐substituted tertiary allylic alcohols (**5r‐5w**). The method is applicable to the preparation of secondary allylic alcohols (**5x‐5ab**), and notably, the valuable deuterium‐labeled allylic alcohol (**5ac**) could be readily produced using the appropriate deuterated sulfone reagent.

### 2.3 Synthetic Applications

**Figure 5:**
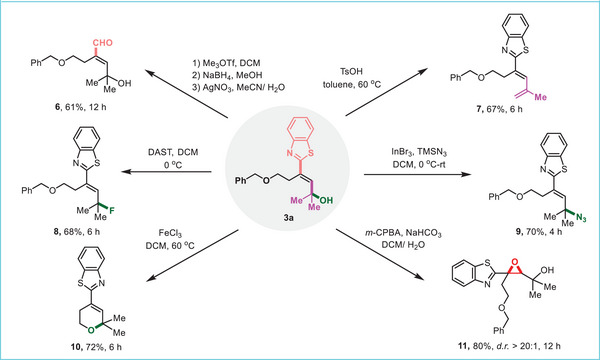
Product transformations.

The products from this reaction could be employed as versatile intermediates, highlighting the synthetic value of the method (**Figure** **5**). The benzothiazolyl group in **3a** serves as the precursor of the carbonyl group, readily releasing a formyl and giving rise to the corresponding conjugated aldehyde (**6**) in a useful yield. The dehydration of **3a** resulted in the conformationally unified s‐*cis* diene (**7**) that could serve in the Diels‐Alder reaction. Treatment of **3a** with diethylaminosulfur trifluoride (DAST) furnished the allylic fluoride (**8**), and with TMSN_3_ afforded allylic azide (**9**). The benzyl group in **3a** was removed in the presence of Lewis acid, and the subsequent spontaneous cyclization led to a 2H‐pyran (**10**). The epoxidation of alkenyl of **3a** smoothly generated the multi‐functionalized oxirane (**11**).

### 2.4 Mechanistic Studies

**Figure 6:**
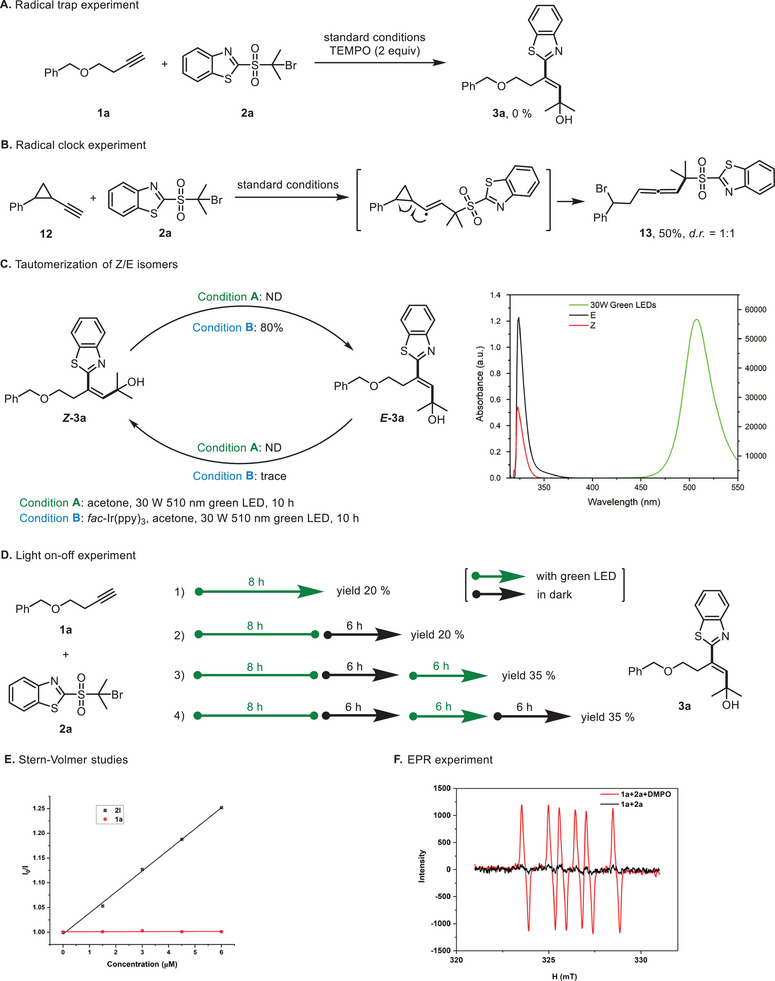
Studies of the mechanism. A) Radical trap experiment. B) Radical clock experiment. C) Tautomerization of Z/E isomers. D) Light on‐off experiment. E) Stern‐Volmer studies. F) EPR experiment.

A set of mechanistic experiments was carried out to elucidate the reaction pathways. The addition of the radical scavenger (2,2,6,6‐tetramethylpiperidin‐1‐yl)oxyl (TEMPO) entirely suppressed the formation of **3a** (**Figure** **6**A). The radical clock reaction of cyclopropylacetylene (**12**) with **2a** furnished the ring‐opened product (**13**), indicating that radical pathways were involved in the reaction which was initiated by homolysis of the C‐Br bond (**2a**) (Figure 6B). The tautomerization of *Z*‐**3a** to *E*‐**3a** took place only in the presence of a photosensitizer under light irradiation. However, this process is irreversible and the conversion of *E*‐**3a** to *Z*‐**3a** failed in the presence or absence of a photosensitizer (Figure 6C). The absorption peaks of *Z‐* **3a** (325 nm) or *E*‐**3a** (329 nm) did not overlap with the emission wavelength of green light (λmax = 510 nm), suggesting that the conversion of *Z*‐ to *E*‐isomer was enabled by the energy transfer (EnT) from the excited photosensitizer to substrate rather than by the direct light excitation of the substrate.^[^ ^14^ ^]^ Light on‐off experiments showed that the reaction proceeded under light irradiation and stopped in its absence (Figure 6D). This result is consistent with the quantum yield of the reaction (Φ < 0.6 for details, see the Supporting Information), illustrating that the reaction is a light‐dependent process. A Stern‐Volmer analysis demonstrated that the luminescence emission of *fac*‐Ir(ppy)_3_ was efficiently quenched by **2a** but not by **1a** (Figure 6E). EPR experiments displayed a conspicuous response signal when 5,5‐dimethyl‐1‐pyrroline *N*‐oxide (DMPO) was added to the reaction as a radical trap (Figure 6F).^[^ ^7b^ ^]^

**Figure 7:**
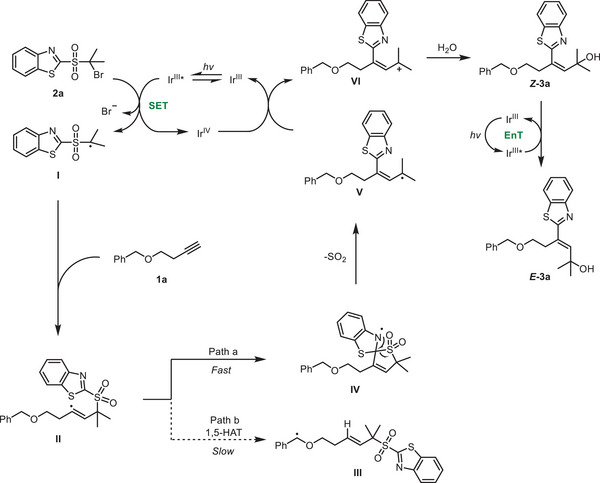
Proposed reaction mechanism.

**Figure 8:**
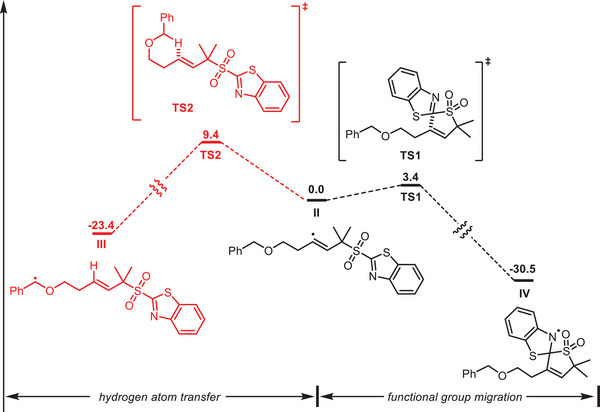
DFT calculations.

A plausible mechanism for the reaction is shown in **Figure** **7**. A single‐electron transfer from the excited *fac*‐Ir(ppy)_3_ to **2a** generates an alkyl radical (**I**) that adds to the alkyne (**1a**) to form an alkenyl radical species (**II**). The reduction potential of **2a** (*E*_p/2_ =−1.1 V vs SCE) determined by cyclic voltammetry (see the SI) supported that the C‐Br bond of **2a** could be readily reduced by the Ir^III*^ species (*E*_1/2_^III*/IV^ =−1.73 V vs SCE). Alkenyl radical **II** is rapidly captured by the heteroaryl moiety, triggering the functional group migration. The competitive alkenyl radical‐mediated 1,5‐HAT is less favorable, and this was supported by DFT calculations (**Figure** **8**) and is not observed in the reaction. As is explicitly shown in Figure 8, the 1,4‐aryl migration proceeds with a lower energetic barrier than a 1,5‐HAT (Table S1 vs Table S2, Supporting Information), leading to a more stable intermediate (**IV**), as opposed to **III**. The ensuing extrusion of SO_2_ affords a radical (**V**) which is then single‐electron oxidized by *in‐situ* generated Ir^IV^ species to form the cation (**VI**) and regenerating the ground‐state Ir^III^ catalyst. Subsequently, the nucleophilic addition of H_2_O to **VI** generates ***Z*‐3a**. With the aid of photosensitizer, the energy transfer causes the conversion of ***Z*‐3a** to the final product (***E*‐3a**) under light irradiation.

## 3 Conclusion

An ingenious photocatalytic protocol proceeding through sequential functional group migration, radical‐polar crossover, and stereoconvergent alkene isomerization has been exploited for the radical di‐functionalization of unactivated alkynes. A hydroxyalkyl and a (hetero)aryl group can be incorporated into alkynes by this reaction, which stereoselectively furnishes densely functionalized *E*‐allyl alcohols. The method can be applied to the modification of complex structures derived from natural products and drug molecules. Comprehensive experimental studies have been conducted to probe the reaction mechanism, and DFT calculations were carried out to rationalize the selectivity of functional group migration beyond an alkenyl radical‐mediated 1,5‐HAT. The protocol features a broad substrate scope and high product diversity, thus opening up a new prospect for free radical‐mediated difunctionalization of unactivated alkynes.

## 4 Experimental Section

### General Procedure for the Preparation of 3, 4 and 5

**1** (0.4 mmol), **2** (0.2 mmol), NaOAc (0.1 mmol), and *fac*‐Ir(ppy)_3_ (0.002 mmol) were loaded into a flask, which had been subjected 3 times to evacuation/flushing with N_2_. Dry acetone (2.0 mL)/H_2_O (0.6 mL) was added by syringe, and the mixture was irradiated by 30 W Green LEDs and stirred at rt until TLC showed that the starting material had been consumed. The mixture was quenched with H_2_O and the aqueous layer was extracted with EtOAc. The organic layer was washed with brine, dried over Na_2_SO_4_, concentrated *in vacuo*, and purified by flash column chromatography on silica gel (eluent: ethyl acetate/petroleum ether) to give the corresponding products.

## Conflict of Interest

The authors declare no conflict of interest.

## Supporting information

Supporting Information

Supporting Information

## Data Availability

The data that support the findings of this study are available from the corresponding author upon reasonable request.
