# Supplementary material for: E‐Selective Radical Difunctionalization of Unactivated Alkynes: Preparation of Functionalized Allyl Alcohols from Aliphatic Alkynes
Source: Adv Sci (Weinh). 2024 Feb 13;11(16):2309022. doi: 10.1002/advs.202309022 (PMC11040374; doi:10.1002/advs.202309022)
Supplement: Supplementary file 1 — Supporting Information [file ADVS-11-2309022-s001.pdf]

ADVANCED SCIENCE
Open Access

Supporting Information

for *Adv. Sci.*, DOI 10.1002/advs.202309022

E-Selective Radical Difunctionalization of Unactivated Alkynes: Preparation of Functionalized Allyl Alcohols from Aliphatic Alkynes

*Jie Wang, Xinxin Wu, Zhu Cao, Xu Zhang, Xinxin Wang, Jie Li and Chen Zhu**

Supporting Information

E-Selective Radical Difunctionalization of Unactivated Alkynes: Preparation of Functionalized Allyl Alcohols from Aliphatic Alkynes

*Jie Wang, Xinxin Wu, Zhu Cao, Xu Zhang, Xinxin Wang, Jie Li and Chen Zhu**

Table of Contents

1. General experimental details S2

2. Reaction optimization S2

3. Preparation of starting materials S6

4. General procedures for difunctionalization of alkynes S15

5. Transformations of **3a** S41

6. Single-crystal X-ray diffraction analysis of **3ai** S45

7. Mechanistic studies S46

8. DFT calculations S53

9. NMR spectra S61

Reference

1. General experimental details

All reactions were maintained under a nitrogen atmosphere unless otherwise stated. Commercially available reagents were used without further purification. DMF was distilled from CaH_2 under reduced pressure, and DCM was distilled from CaH_2, and THF was distilled from sodium. Infrared (FT-IR) spectra were recorded on a BRUKER VERTEX 70, ν_{max} in cm^{-1}. ^{1}H-NMR spectra were recorded on a BRUKER AVANCE III HD (400 MHz) spectrometer. Chemical shifts are reported in ppm from tetramethylsilane with the solvent resonance as internal standard ($CDCl_3$: δ 7.26, $(CD_3)_2SO$: δ 2.50). Data are reported as follows: chemical shift, multiplicity (s = singlet, d = doublet, t = triplet, q = quadruplet, br = broad, m = multiplet), coupling constants (Hz) and integration. ^{13}C-NMR spectra were recorded on a BRUKER AVANCE III HD (100 MHz) spectrometer with complete proton decoupling. Chemical shifts are reported in ppm from tetramethylsilane with the solvent resonance as the internal standard ($CDCl_3$: δ 77.16, $(CD_3)_2SO$: δ 39.52). ^{19}F-NMR spectra were recorded on a BRUKER AVANCE III HD (376 MHz) spectrometer. Mass spectra were measured with an Agilent Technologies 6120 Quadrupole LC/MS. High resolution mass spectrometry (HRMS) were measured with a GCT Premier™ and BRUKER micrOTF-Q III. Melting points were measured using INESA WRR and values are uncorrected. Quantum yield was measured by using Cary 5000 UV-Vis-NIR Spectrophotometer.

2. Reaction optimization

2.1 Reaction setups

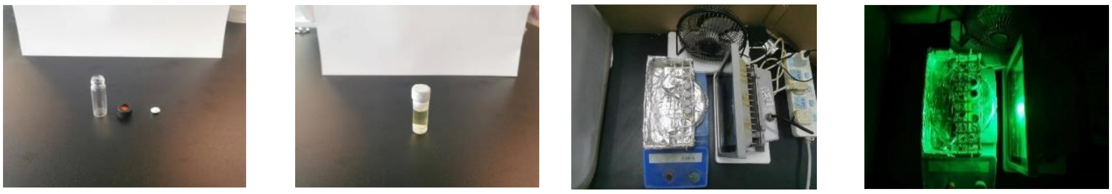

a **b** **c** **d**

Figure S1. Reaction setups. a, Reaction bottle, hollow cap and magnetic stirrer; **b**, 0.2 mmol scale reactor; **c**, Reaction device; **d**, Reaction irradiated by the green LED (510 nm).

2.2 Reaction parameters survey

Table S1. Evaluation of the amount of base[a]

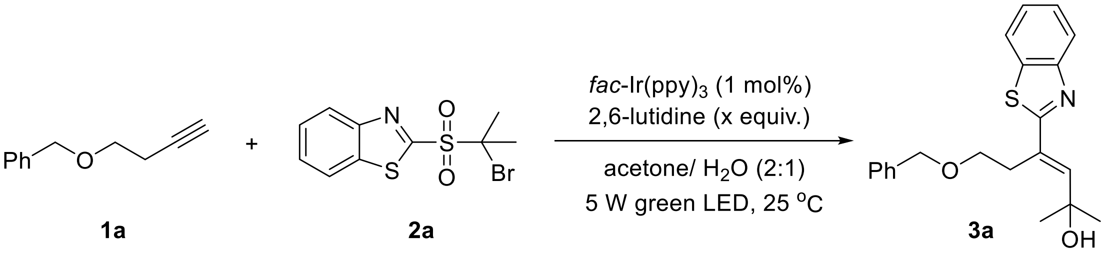

Entry	2,6-Lutidine (x equiv.)	Yield (%)[b]	E/Z
1	-	44	>20:1
2	0.1	46	>20:1
3	0.3	51	>20:1
4	0.5	65	>20:1
5	0.7	49	17:1
6	0.9	50	13:1
7	1.0	50	10:1
8	2.0	52	8:1

[a] Standard reaction conditions: **1a** (0.2 mmol), **2a** (0.1 mmol), *fac*-Ir(ppy)$_3$ (1 mol%), and 2,6-lutidine (x equiv.) in acetone/H_2O (1.0 mL/0.2 mL) under N_2 at rt, irradiated with 5 W green LED. [b] Yields of isolated products.

Table S2. Evaluation of the solvents[a]

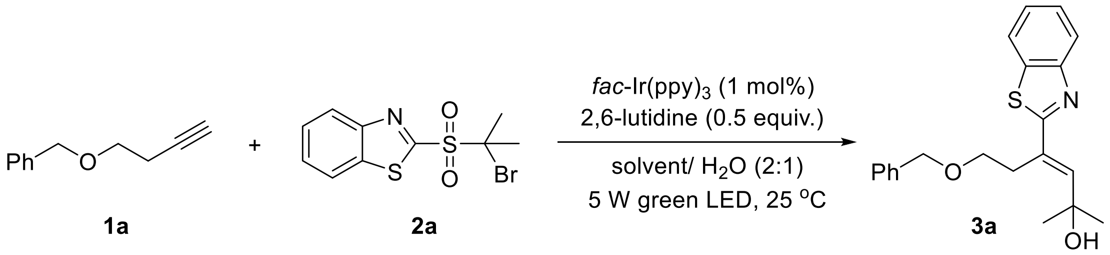

Entry	Solvent (mL)	Yield (%)[b]	E/Z
1	MeCN	37	>20:1
2	EtOAc	43	17:1
3	DCE	35	5:1
4	$PhCF_3$	35	10:1
5	DMF	32	20:1
6	DMSO	28	7:1
7	$MeNO_2$	40	>20:1

8	acetone	65	>20:1

[a] Standard reaction conditions: **1a** (0.2 mmol), **2a** (0.1 mmol), *fac*-Ir(ppy)$_3$ (1 mol%), and 2,6-lutidine (0.5 equiv.) in solvent/H_2O (1.0 mL/0.2 mL) under N_2 at rt, irradiated with 5 W green LED. [b] Yields of isolated products.

Table S3. Evaluation of the base[a]

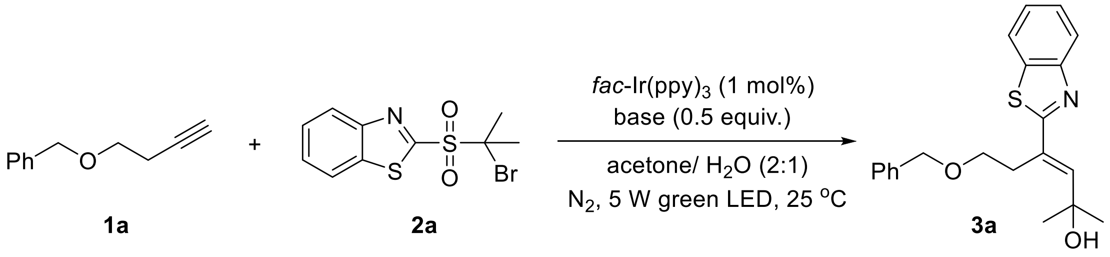

Entry	Additive	Yield (%)[b]	E/Z
1	DBU	52	>20:1
2	DABCO	52	13:1
3	NaOAc	66	>20:1
4	PhCOONa	63	>20:1
5	$NaHCO_3$	54	>20:1
6	$KHCO_3$	46	>20:1
7	KOAc	54	>20:1
8	PhCOOK	57	>20:1
9	NaH_2PO_4	60	>20:1
10	2,6-lutidine	65	>20:1

[a] Standard reaction conditions: **1a** (0.2 mmol), **2a** (0.1 mmol), *fac*-Ir(ppy)$_3$ (1 mol%), and additive (0.5 equiv.) in acetone/H_2O (1.0 mL/0.2 mL) under N_2 at rt, irradiated with 5 W green LED. [b] Yields of isolated products.

Table S4. Evaluation of the photosensitizer[a]

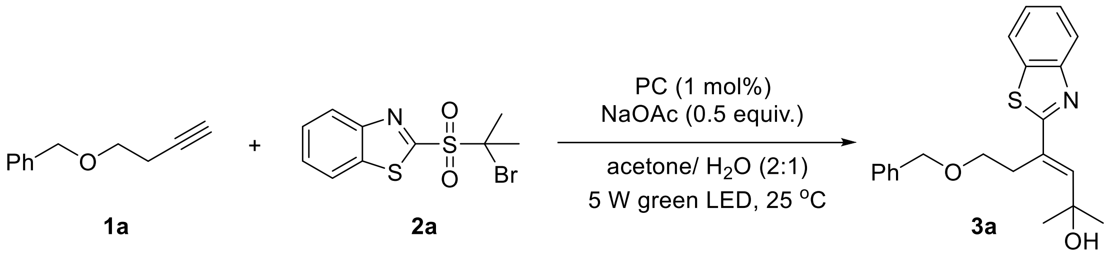

Entry	PC	Yield (%)[c]	E/Z
1	Ir[dF(CF$_3$)ppy]$_2$(dtbbpy)PF$_6$	0	-
2	[Ir(dtbbpy)(ppy)$_2$]PF$_6$	10	>20:1
3	4CzIPN	0	-
4	Ru(bpy)$_3$Cl$_2$·6H$_2$O	0	-
5	Eosin Y[b]	<5	>20:1
6	Eosin B[b]	<5	>20:1
7	PTH[b]	0	-

[a] Standard reaction conditions: **1a** (0.2 mmol), **2a** (0.1 mmol), PC (1 mol%), and additive (0.5 equiv.) in acetone/H_2O (1.0 mL/0.2 mL) under N_2 at rt, irradiated with 5 W green LED. [b] PC (5 mol%). [c] Yields of isolated products.

Table S5. Evaluation of the amount of water[a]

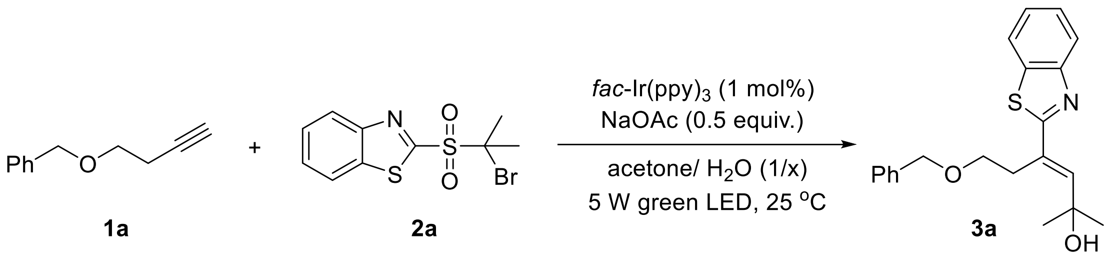

Entry	Water (mL)	Yield (%)[b]	E/Z
1	-	0	-
2	0.1	47	>20:1
3	0.3	70	>20:1

4	0.5	67	>20:1
5	0.7	43	14:1
6	1.0	35	10:1

[a] Standard reaction conditions: **1a** (0.2 mmol), **2a** (0.1 mmol), *fac*-Ir(ppy)$_3$ (1 mol%), and additive (0.5 equiv.) in acetone/H_2O (1.0 mL/x mL) under N_2 at rt, irradiated with 5 W green LED. [b] Yields of isolated products.

Table S6. Evaluation of light source[a]

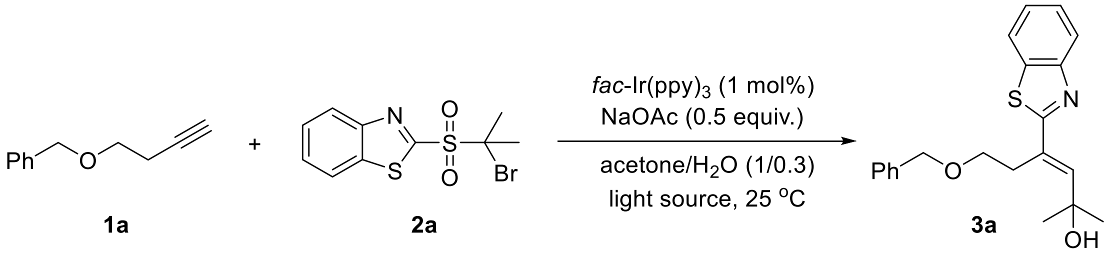

Entry	Light source	Yield (%)[b]	E/Z
1	30 W green LED	78	>20:1
2	5 W blue LED	57	5:1
3	30 W CFL	63	8:1

[a] Standard reaction conditions: **1a** (0.2 mmol), **2a** (0.1 mmol), *fac*-Ir(ppy)$_3$ (1 mol%), and additive (0.5 equiv.) in acetone/H_2O (1.0 mL/0.3 mL) under N_2 at rt, irradiated with visible light. [b] Yields of isolated products.

3. Reaction Preparation of starting materials

3.1 Synthesis of alkyne substrates

Alkynes (**1a**[1], **1j**[2], **1k**[3], **1p**[4], **1x**[5], **1z** and **1au**[6], **1ab**、**1ad**、**1ao**、**1ay** and **1az**[7], **1ac**[8], **1ag**[9], **1ap**[10], **1at**[11], **1av**[12]) were prepared following reported procedures, and characterization data are in agreement with the corresponding literatures. The unreported alkynes are prepared according to the following procedures. Other alkynes, which are not mentioned herein, are commercially available.

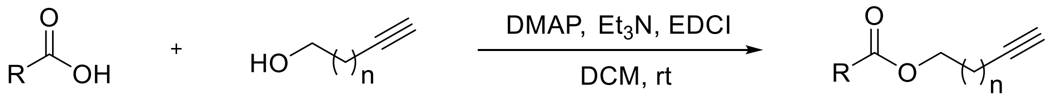

Figure S2. Synthesis of alkyne ester substrates

To a solution of alkyl/aryl carboxylic acid (1.0 equiv), DMAP (0.20 equiv) and Et_3N (3.0 equiv) in CH_2Cl_2 (5.0 mL) at room temperature was added EDCI (2.0 equiv) and alkynol (1.2 equiv). The reaction mixture was stirred at room temperature for 6 h before being quenched with H_2O (10.0 mL), and was extracted 3 times with CH_2Cl_2 (10.0 mL). The combined organic layer was dried over $MgSO_4$. The filtrate was concentrated in vacuo and the residue was purified by flash column chromatography on silica gel (eluent: EtOAc/Petroleum ether).

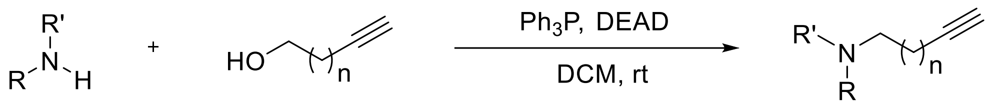

Figure S3. Synthesis of alkyne amine substrates

To a stirred solution of alkynol (1.0 equiv), amide (1.3 equiv), and Ph_3P (1.3 equiv) was added DEAD (1.3 equiv) in THF (10.0 mL) at 0 °C under an argon atmosphere. The resulting mixture was slowly warmed to room temperature and stirred for 2.5 h. Then the reaction was quenched with H_2O and extracted with EtOAc (20 mL × 3). The combined organic extracts were washed with brine (40 mL), dried over Na_2SO_4, and concentrated under vacuum. The filtrate was concentrated in vacuo and the residue was purified by flash column chromatography on silica gel (eluent: EtOAc/Petroleum ether).

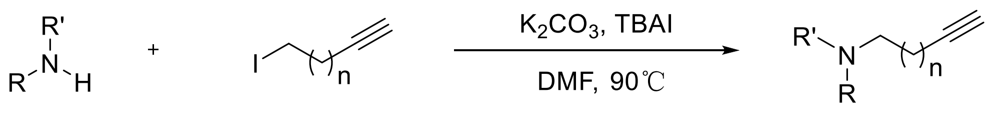

Figure S4. Synthesis of alkyne amine substrates

To a round-bottom flask were added amide (1.0 equiv), K_2CO_3 (1.2 equiv), TBAI (1.0 equiv) and DMF (10.0 mL). To this solution was added alkynyl iodine (1.5 equiv), and the mixture was stired at 90 °C for 16 h. The reaction was cooled to room temperature, quenched with 2M HCl (aq) (10 mL), and extracted with EtOAc (20 mL) for 3 times. The combined organic layers were dried over $MgSO_4$, concentrated, and purified by flash column chromatography on silica gel (eluent: EtOAc/Petroleum ether).

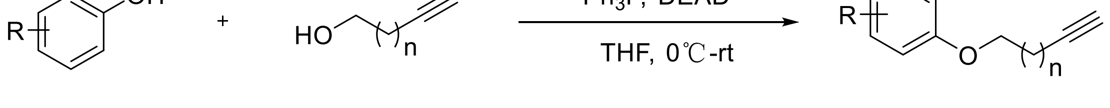

Figure S5. Synthesis of alkyne ether substrates

To a solution of phenol derivative (1.0 equiv) in dry THF (10 mL) under argon, Ph_3P (1.2 equiv) and alkynol (1.2 equiv) were added. The mixture was cooled to 0 °C and DEAD (1.25 equiv) was

added dropwise. The solution was allowed to stirred at room temperature for 18 h. The solvent was evaporated and the residue was extracted twice with CH_2Cl_2 (30 mL). The resulting suspension was filtrated and washed with CH_2Cl_2. The filtrate was concentrated in vacuo and the residue was purified by flash column chromatography on silica gel (eluent: EtOAc/Petroleum ether).

Compounds **1o**, **1q**, **1s-1t**, **1v-1w**, **1y**, **1aa**, **1ad**, **1ae-1af**, **1ao**, **1aq-1as**, **1aw-1ax**, **1az** are newly synthesize.

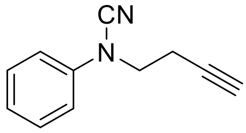

***N*-(but-3-yn-1-yl)-*N*-phenylcyanamide (1o)**: Colorless oil. ^{1}H NMR (400 MHz, $CDCl_3$) δ 7.41-7.35 (m, 2H), 7.16-7.10 (m, 3H), 3.80 (t, *J* = 7.2 Hz, 2H), 2.71-2.65 (m, 2H), 2.09 (t, *J* = 2.8 Hz, 1H); ^{13}C NMR (100 MHz, $CDCl_3$) δ 139.4, 129.9, 124.1, 116.2, 113.3, 79.2, 71.6, 48.4, 17.9. FT-IR: ν (cm^{-1}) 3279, 2897, 2218, 1598, 1205, 752, 687. HRMS [ESI] calcd for $C_{11}H_{10}N_2Na$ $[M+Na]^+$ 193.0736, found 193.0742.

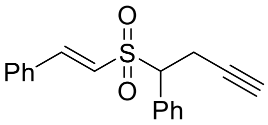

(*E*)-(2-((1-phenylbut-3-yn-1-yl)sulfonyl)vinyl)benzene (1q): Colorless oil. ^{1}H NMR (400 MHz, $CDCl_3$) δ 7.45-7.37 (m, 11H), 6.54 (d, *J* = 15.6 Hz, 1H), 4.28 (dd, *J* = 10.4, 4.8 Hz, 1H), 3.36-3.28 (m, 1H), 3.14-3.04 (m, 1H), 1.93 (t, *J* = 2.8 Hz, 1H); ^{13}C NMR (100 MHz, $CDCl_3$) δ 146.4, 132.3, 131.6, 131.5, 130.0, 129.5, 129.3, 129.0, 128.7, 123.1, 79.2, 71.5, 69.0, 19.1. FT-IR: ν (cm^{-1}) 3067, 2865, 1921, 1735, 1376, 1232, 1016, 854. HRMS [ESI] calcd for $C_{18}H_{16}O_2SNa$ $[M+Na]^+$ 319.0763, found 319.0759.

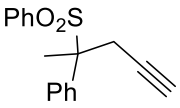

((2-phenylpent-4-yn-2-yl)sulfonyl)benzene (1s): Colorless oil. ^{1}H NMR (400 MHz, $CDCl_3$) δ 7.57-7.51 (m, 1H), 7.36-7.24 (m, 9H), 3.41 (dd, *J* = 16.8, 2.0 Hz, 1H), 3.11 (dd, *J* = 16.8, 2.8 Hz, 1H), 1.92 (s, 3H), 1.87 (t, *J* = 2.8 Hz, 1H); ^{13}C NMR (100 MHz, $CDCl_3$) δ 134.7, 134.3, 133.8, 130.5, 129.1, 128.8, 128.5, 128.2, 78.4, 72.2, 68.4, 25.1, 19.6. FT-IR: ν (cm^{-1}) 3299, 2973, 2900, 1380, 1147, 1048, 880, 670. HRMS [ESI] calcd for $C_{17}H_{16}O_2SNa$ $[M+Na]^+$ 307.0763, found 307.0769.

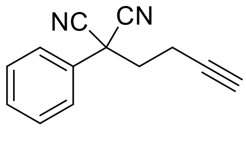

2-(but-3-yn-1-yl)-2-phenylmalononitrile (1t): White solid, m.p. 66-67 °C. ^{1}H NMR (400 MHz, $CDCl_3$) δ 7.59-7.55 (m, 2H), 7.54-7.48 (m, 3H), 2.50-2.49 (m, 4H), 2.05-2.03 (m, 1H); ^{13}C NMR (100 MHz, $CDCl_3$) δ 131.3, 130.4, 130.0, 125.9, 114.4, 79.9, 71.0, 41.6, 41.0, 15.5. FT-IR: ν (cm^{-1}) 3285, 2922, 2250, 1493, 1278, 1041, 767, 671. HRMS [ESI] calcd for $C_{13}H_{10}N_2Na$ $[M+Na]^+$ 217.0736, found 217.0742.

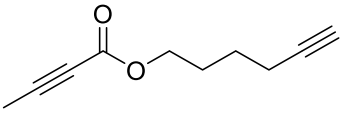

hex-5-yn-1-yl but-2-ynoate (1v): Colorless oil. ^{1}H NMR (400 MHz, $CDCl_3$) δ 4.13 (t, *J* = 6.4 Hz, 2H), 2.22-2.17 (m, 2H), 1.95 (s, 3H), 1.93 (t, *J* = 2.4 Hz, 1H), 1.80-1.69 (m, 2H), 1.62-1.53 (m, 2H); ^{13}C NMR (100 MHz, $CDCl_3$) δ 153.8, 85.6, 83.7, 72.4, 68.9, 65.2, 27.5, 24.8, 18.0, 3.8. FT-IR: ν (cm^{-1}) 3294, 2954, 2241, 1703, 1247, 1065, 751, 636. HRMS [ESI] calcd for $C_{10}H_{12}O_2K$ $[M+K]^+$ 203.0469, found 203.0472.

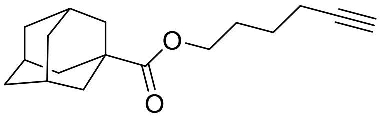

hex-5-yn-1-yl (3r,5r,7r)-adamantane-1-carboxylate (1w): Colorless oil. ^{1}H NMR (400 MHz, $CDCl_3$) δ 4.06 (t, *J* = 6.4 Hz, 2H), 2.26-2.18 (m, 2H), 2.03-1.97 (m, 3H), 1.95 (t, *J* = 2.4 Hz, 1H), 1.89-1.85 (m, 6H), 1.78-1.66 (m, 8H), 1.63-1.55 (m, 2H); ^{13}C NMR (100 MHz, $CDCl_3$) δ 177.8, 84.1, 68.8, 63.6, 40.8, 39.0, 36.6, 28.1, 27.9, 25.1, 18.2. FT-IR: ν (cm^{-1}) 3308, 2905, 2851, 1723, 1453, 1231, 1073, 627. HRMS [ESI] calcd for $C_{17}H_{24}O_2Na$ $[M+Na]^+$ 283.1669, found 283.1672.

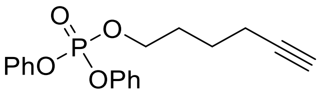

hex-5-yn-1-yl diphenyl phosphate (1y): Colorless oil. ^{1}H NMR (400 MHz, $CDCl_3$) δ 7.37-7.31 (m, 4H), 7.25-7.15 (m, 6H), 4.31-4.25 (m, 2H), 2.23-2.15 (m, 2H), 1.95 (t, *J* = 2.8 Hz, 1H), 1.88-1.78 (m, 2H), 1.64-1.53 (m, 2H); ^{13}C NMR (100 MHz, $CDCl_3$) δ 150.6 (d, J_{C-P} = 7.1 Hz), 129.9, 125.4 (d, J_{C-P} = 1.0 Hz), 120.1 (d, J_{C-P} = 4.8 Hz), 83.7, 69.0, 68.8 (d, J_{C-P} = 6.4 Hz), 29.1 (d, J_{C-P} = 6.9 Hz), 24.3, 17.9; ^{31}P NMR (202 MHz, $CDCl_3$) δ -11.8. FT-IR: ν (cm^{-1}) 3445, 3064, 2870, 1756, 1432, 1153, 1016, 949. HRMS [ESI] calcd for $C_{18}H_{21}O_4P$ $[M+H]^+$ 331.1094, found 331.1092.

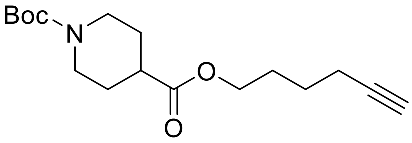

1-(tert-butyl) 4-(hex-5-yn-1-yl) piperidine-1,4-dicarboxylate (1aa): Colorless oil. ^{1}H NMR (400 MHz, $CDCl_3$) δ 4.08 (t, *J* = 6.4 Hz, 2H), 4.02-3.92 (m, 2H), 2.80 (t, *J* = 11.6 Hz, 2H), 2.47-2.35 (m, 1H), 2.25-2.15 (m, 2H), 1.96-1.90 (m, 1H), 1.89-1.79 (m, 2H), 1.78-1.68 (m, 2H), 1.65-1.51 (m, 4H), 1.42 (s, 9H); ^{13}C NMR (100 MHz, $CDCl_3$) δ 174.6, 154.7, 83.8, 79.6, 77.4, 68.9, 64.0, 41.2, 28.4, 28.0, 27.7, 24.9, 18.1. FT-IR: ν (cm^{-1}) 3299, 2953, 1729, 1687, 1451, 1392, 1158, 1036, 755. HRMS [ESI] calcd for $C_{17}H_{27}NO_4Na$ $[M+Na]^+$ 332.1832, found 332.1830.

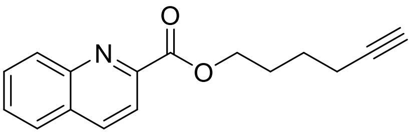

hex-5-yn-1-yl quinoline-2-carboxylate (1ad): White solid, m.p. 53-54 °C. ^{1}H NMR (400 MHz, $CDCl_3$) δ 8.33-8.26 (m, 2H), 8.16 (d, *J* = 8.8 Hz, 1H), 7.87 (d, *J* = 8.4 Hz, 1H), 7.81-7.75 (m, 1H), 7.67-7.60 (m, 1H), 4.51 (t, *J* = 6.4 Hz, 2H), 2.35-2.25 (m, 2H), 2.05-1.95 (m, 3H), 1.77-1.67 (m, 2H); ^{13}C NMR (100 MHz, $CDCl_3$) δ 165.5, 148.2, 147.8, 137.3, 130.9, 130.3, 129.4, 128.7, 127.6, 121.1, 84.0, 69.0, 65.7, 27.9, 25.1, 18.3. FT-IR: ν (cm^{-1}) 3223, 2861, 2145, 1721, 1389, 1243, 1089, 877. HRMS [ESI] calcd for $C_{16}H_{15}NO_2Na$ $[M+Na]^+$ 276.0995, found 276.0988.

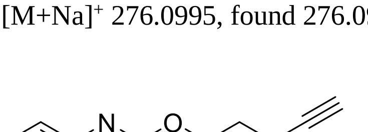

2-(pent-4-yn-1-yloxy)quinoxaline (1ae): Colorless oil. ^{1}H NMR (400 MHz, $CDCl_3$) δ 8.46 (s, 1H), 8.01 (d, *J* = 8.0 Hz, 1H), 7.83 (d, *J* = 8.4 Hz, 1H), 7.70-7.63 (m, 1H), 7.59-7.53 (m, 1H), 4.59 (t, *J* = 6.2 Hz, 2H), 2.47-2.42 (m, 2H), 2.14-2.06 (m, 2H), 1.99 (t, *J* = 2.4 Hz, 1H); ^{13}C NMR (100 MHz, $CDCl_3$) δ 157.4, 140.5, 139.8, 130.3, 129.1, 127.4, 126.7, 83.4, 69.2, 65.0, 27.9, 15.5. FT-IR: ν (cm^{-1}) 3242, 2959, 1653, 1472, 1394, 1277, 1126, 761, 667. HRMS [ESI] calcd for $C_{13}H_{13}N_2O$ $[M+H]^+$ 213.1022, found 213.1027.

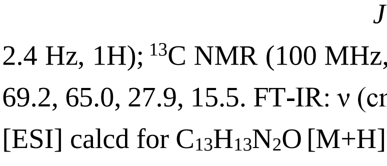

2-(hex-5-yn-1-yloxy)-4*H*-benzo[*d*][1,3]oxazin-4-one (1af): Colorless oil. ^{1}H NMR (400 MHz, $CDCl_3$) δ 8.12 (dd, *J* = 8.0, 1.2 Hz, 1H), 7.76-7.69 (m, 1H), 7.42 (d, *J* = 8.0 Hz, 1H), 7.35

(t, *J* = 7.6 Hz, 1H), 4.49 (t, *J* = 6.4 Hz, 2H), 2.33-2.25 (m, 2H), 2.01-1.91 (m, 3H), 1.77-1.67 (m, 2H); ^{13}C NMR (100 MHz, $CDCl_3$) δ 164.3, 159.7, 146.3, 137.0, 129.2, 126.0, 125.5, 83.8, 69.6, 69.1, 27.5, 24.8, 18.2. FT-IR: ν (cm^{-1}) 3224, 2925, 1732, 1456, 1245, 1172, 1021, 865. HRMS [ESI] calcd for $C_{14}H_{13}NO_3Na$ $[M+Na]^+$ 266.0788, found 266.0793.

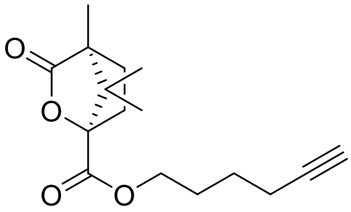

hex-5-yn-1-yl (1*R*,4*S*)-4,7,7-trimethyl-3-oxo-2-oxabicyclo[2.2.1]heptane-1-carboxylate (1ao): Colorless oil. ^{1}H NMR (400 MHz, $CDCl_3$) δ 4.29-4.22 (m, 2H), 2.46-2.37 (m, 1H), 2.27-2.21 (m, 2H), 2.07-1.98 (m, 1H), 1.95 (t, *J* = 2.4 Hz, 1H), 1.94-1.87 (m, 1H), 1.86-1.78 (m, 2H), 1.72-1.65 (m, 1H), 1.65-1.56 (m, 2H), 1.11 (s, 3H), 1.05 (s, 3H), 0.95 (s, 3H); ^{13}C NMR (100 MHz, $CDCl_3$) δ 178.3, 167.7, 91.3, 83.7, 69.1, 65.2, 54.9, 54.3, 30.8, 29.1, 27.7, 24.9, 18.1, 16.9, 16.9, 9.8. FT-IR: ν (cm^{-1}) 3258, 2973, 1779, 1737, 1361, 1234, 1154, 996, 671. HRMS [ESI] calcd for $C_{16}H_{22}O_4Na$ $[M+Na]^+$ 301.1140, found 301.1151.

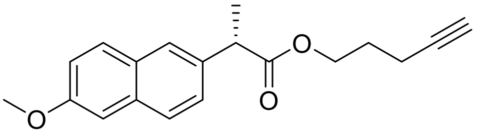

pent-4-yn-1-yl (*S*)-2-(6-methoxynaphthalen-2-yl)propanoate (1aq): White solid, m.p. 52-53 °C. ^{1}H NMR (400 MHz, $CDCl_3$) δ 7.71 (d, *J* = 8.8 Hz, 2H), 7.66 (s, 1H), 7.40 (dd, *J* = 8.4, 1.2 Hz, 1H), 7.17-7.10 (m, 2H), 4.19 (t, *J* = 6.4 Hz, 2H), 3.91 (s, 3H), 3.86 (q, *J* = 7.2 Hz, 1H), 2.20-2.13 (m, 2H), 1.93 (t, *J* = 2.4 Hz, 1H), 1.84-1.75 (m, 2H), 1.58 (d, *J* = 6.8 Hz, 3H); ^{13}C NMR (100 MHz, $CDCl_3$) δ 174.7, 157.8, 135.8, 133.8, 129.4, 129.0, 127.3, 126.3, 126.0, 119.1, 105.7, 83.1, 69.1, 63.4, 55.4, 45.6, 27.6, 18.6, 15.2. FT-IR: ν (cm^{-1}) 3292, 2971, 1728, 1605, 1391, 1263, 1175, 1029, 853. HRMS [ESI] calcd for $C_{19}H_{21}O_3$ $[M+H]^+$ 297.1485, found 297.1494.

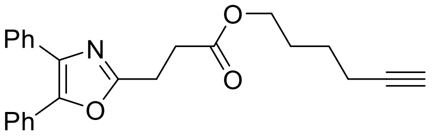

hex-5-yn-1-yl 3-(4,5-diphenyloxazol-2-yl)propanoate (1ar): Colorless oil. ^{1}H NMR (400 MHz, $CDCl_3$) δ 7.65-7.61 (m, 2H), 7.60-7.55 (m, 2H), 7.40-7.29 (m, 6H), 4.16 (t, *J* = 6.4 Hz, 2H), 3.19 (t, *J* = 7.6 Hz, 2H), 2.92 (t, *J* = 7.2 Hz, 2H), 2.23-2.16 (m, 2H), 1.94 (t, *J* = 2.8 Hz, 1H), 1.81-1.72 (m, 2H), 1.63-1.54 (m, 2H); ^{13}C NMR (100 MHz, $CDCl_3$) δ 172.1, 161.8, 145.5, 135.2, 132.6, 129.1, 128.7, 128.6, 128.5, 128.1, 128.0, 126.6, 83.9, 68.9, 64.4, 31.2, 27.7, 24.9, 23.7, 18.1. FT-IR: ν (cm^{-1}) 3296, 3058, 2951, 1732, 1571, 1444, 1165, 1056, 961, 762, 693. HRMS [ESI] calcd for $C_{24}H_{23}NO_3Na$ $[M+Na]^+$ 396.1570, found 396.1575.

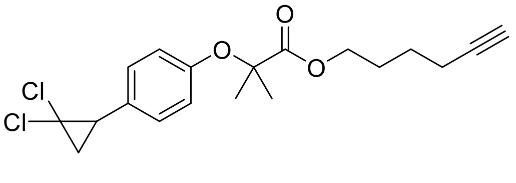

hex-5-yn-1-yl 2-(4-(2,2-dichlorocyclopropyl)phenoxy)-2-methylpropanoate (1as): Colorless oil. ^{1}H NMR (500 MHz, $CDCl_3$) δ 7.12-7.08 (m, 2H), 6.82-6.77 (m, 2H), 4.17 (t, *J* = 6.3 Hz, 2H), 2.82 (dd, *J* = 8.3, 10.8 Hz, 1H), 2.16-2.11 (m, 2H), 1.95-1.90 (m, 2H), 1.79-1.68 (m, 3H), 1.60 (s, 6H), 1.48-1.42 (m, 2H); ^{13}C NMR (125 MHz, $CDCl_3$) δ 174.4, 155.1, 129.8, 128.2, 118.5, 83.9, 79.3, 68.9, 65.0, 61.0, 34.9, 27.6, 25.9, 25.6, 25.6, 24.8, 18.1. FT-IR: ν (cm^{-1}) 3302, 2989, 2251, 1731, 1611, 1510, 1138, 909, 730, 642. HRMS [ESI] calcd for $C_{19}H_{22}Cl_2O_3Na$ $[M+Na]^+$ 391.0838, found 391.0833.

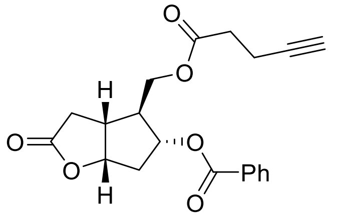

(3a*R*,4*S*,5*R*,6a*S*)-2-oxo-4-((pent-4-ynoyloxy)methyl)hexahydro-2*H*-cyclopenta[*b*]furan-5-yl benzoate (1aw): Colorless oil. ^{1}H NMR (400 MHz, $CDCl_3$) δ 7.99-7.94 (m, 2H), 7.58-7.51 (m, 1H), 7.42 (t, *J* = 7.6 Hz, 2H), 5.36-5.30 (m, 1H), 5.06 (t, *J* = 6.4 Hz, 1H), 4.15 (d, *J* = 6.0 Hz, 2H), 2.95-2.86 (m, 1H), 2.84-2.75 (m, 1H), 2.59-2.52 (m, 3H), 2.51-2.44 (m, 4H), 2.38-2.29 (m, 1H), 1.98 (t, *J* = 2.4 Hz, 1H); ^{13}C NMR (100 MHz, $CDCl_3$) δ 176.3, 171.6, 166.0, 133.5, 129.7, 129.5, 128.6, 84.2, 82.3, 77.3, 69.4, 64.2, 51.7, 40.6, 38.3, 35.9, 33.3, 14.4. FT-IR: ν (cm^{-1}) 3282, 2956, 1768, 1713, 1272, 1159, 1108, 1069, 753, 711. HRMS [ESI] calcd for $C_{20}H_{20}O_6Na$ [M+Na]$^+$ 379.1152, found 379.1160.

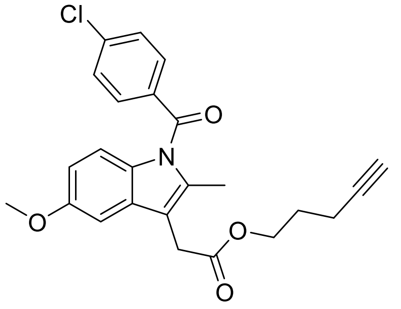

pent-4-yn-1-yl 2-(1-(4-chlorobenzoyl)-5-methoxy-2-methyl-1*H*-indol-3-yl)acetate (1ax): Colorless oil. ^{1}H NMR (400 MHz, $CDCl_3$) δ 7.65 (d, *J* = 8.0 Hz, 2H), 7.46 (d, *J* = 7.6 Hz, 2H), 6.96 (d, *J* = 2.4 Hz, 1H), 6.87 (d, *J* = 9.2 Hz, 1H), 6.67 (dd, *J* = 8.8, 2.4 Hz, 1H), 4.21 (t, *J* = 6.4 Hz, 2H), 3.83 (s, 3H), 3.66 (s, 2H), 2.38 (s, 3H), 2.25-2.18 (m, 2H), 1.96 (t, *J* = 2.4 Hz, 1H), 1.89-1.80 (m, 2H); ^{13}C NMR (100 MHz, $CDCl_3$) δ 170.9, 168.4, 156.2, 139.3, 136.0, 134.0, 131.3, 130.9, 130.7, 129.2, 115.1, 112.6, 111.8, 101.3, 82.9, 69.3, 63.6, 55.8, 30.4, 27.6, 15.2, 13.5. FT-IR: ν (cm^{-1}) 3287, 2963, 1721, 1674, 1476, 1288, 1087, 992, 869, 727. HRMS [ESI] calcd for $C_{24}H_{22}ClNO_4Na$ [M+Na]$^+$ 446.1130, found 446.1138.

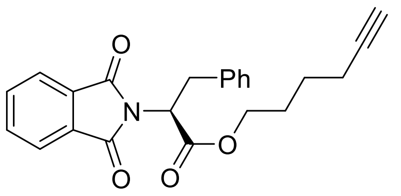

hex-5-yn-1-yl (*S*)-2-(1,3-dioxoisoindolin-2-yl)-3-phenylpropanoate (1az): Colorless oil. ^{1}H NMR (400 MHz, $CDCl_3$) δ 7.79-7.74 (m, 2H), 7.70-7.65 (m, 2H), 7.21-7.09 (m, 5H), 5.15 (dd, *J* = 11.2, 5.2 Hz, 1H), 4.27-4.16 (m, 2H), 3.64-3.47 (m, 2H), 2.20-2.13 (m, 2H), 1.91 (t, *J* = 2.4 Hz, 1H), 1.80-1.69 (m, 2H), 1.57-1.47 (m, 2H); ^{13}C NMR (100 MHz, $CDCl_3$) δ 168.9, 167.6, 136.8, 134.2, 131.7, 128.9, 128.6, 126.9, 123.5, 83.8, 68.9, 65.5, 53.5, 34.8, 27.5, 24.8, 18.0. FT-IR: ν (cm^{-1}) 3286, 2947, 1711, 1385, 1239, 1104, 873, 718. HRMS [ESI] calcd for $C_{23}H_{22}NO_4$ [M+H]$^+$ 376.1543, found 376.1552.

3.1 Synthesis of difunctionalization reagents

Compound **2a-2k, 2r-2z** and **2aa-2ac**[13] were prepared following the reported procedures, and the characterization data are in agreement with the literatures. Other sulfone reagents are new and prepared according to the following procedures:

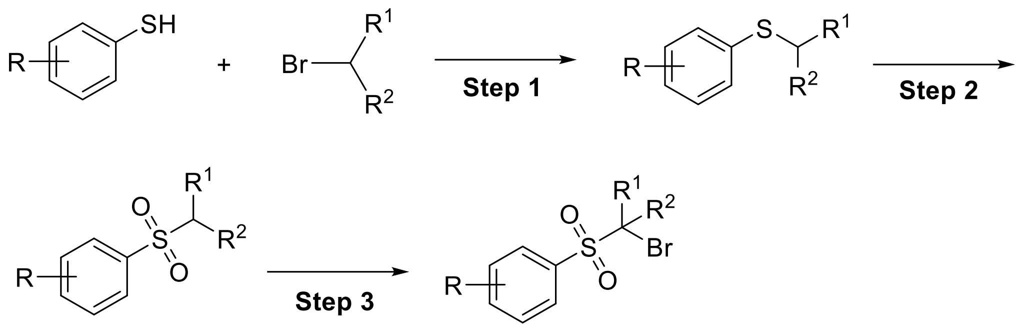

Figure S6. Synthesis of alkyl aryl sulfone difunctionalization reagent

Step 1: To a 50 mL two-necked flask, aryl thiophenol (5.0 mmol, 1.0 equiv.), K_2CO_3 (7.0 mmol, 1.4 equiv.), alkyl bromide (6.0 mmol, 1.2 equiv.) and acetone (15 mL) were added. The mixture was refluxed for 12 h. After cooling to room temperature, water (45 mL) was added and the organic materials were extracted with Et_2O (3 x 150 mL). The combined organic layers were washed with brine (100 mL), dried over Na_2SO_4, concentrated in vacuo, and purified by flash column chromatography on silica gel (eluent: EtOAc/Petroleum ether) to give the corresponding product.

Step 2: To a 100 mL round bottom flask was added alkyl phenyl sulfide (6.57 mmol, 1.0 equiv.) and oxone (19.7 mmol, 3.0 equiv.). The mixture was suspended in 1:1 acetone/water (30 mL) and stirred at room temperature overnight. The reaction mixture was then diluted with DCM and water, and then transferred to a separating funnel. The organic phase was collected, dried over Na_2SO_4, concentrated in vacuo, and purified by flash column chromatography on silica gel (eluent: EtOAc/Petroleum ether) to give the corresponding product.

Step 3: In a sealed flask, a solution of alkyl phenyl sulfone (2.6 mmol) in THF (5 mL) and ^{n}BuLi (2.6 mmol), maintained between -70 °C to -60 °C under argon, was stirred for 1 h. A bromine-hexane solution (2.6 mmol of Br_2 and 4.45 mL hexanes) was added, and the mixture was stirred for 30 min at -65 °C. Then saturated NH_4Cl (20 mL) was added to quench the reaction and the organic materials were extracted with Et_2O (3 x 20 mL). The combined organic layers were washed with brine (20 mL), dried over Na_2SO_4, concentrated in vacuo, and purified by flash column chromatography on silica gel (eluent: EtOAc/Petroleum ether) to give the corresponding product.

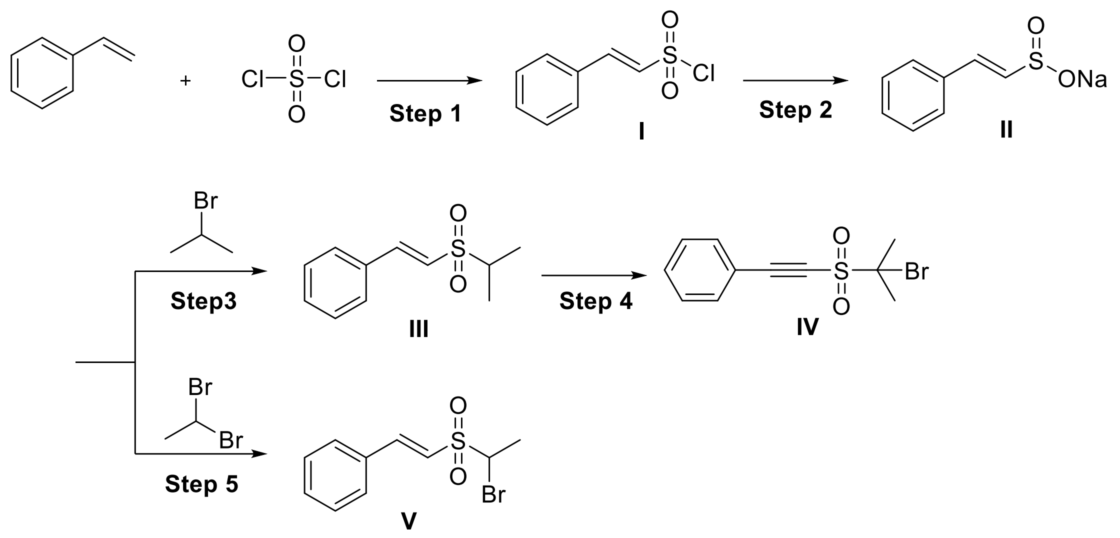

Figure S7. Synthesis of alkyl styryl or phenylethynyl sulfone difunctionalization reagent

Step 1: Sulfuryl chloride (20 mmol, 2.0 equiv.) was added dropwise to stirred anhydrous DMF (4.8 mL) at 0 °C under N_2. After the addition was completed, the mixture was warmed to room temperature and stirred for 0.5 h. Styrene (10 mmol, 1.0 equiv.) was then added and the reaction mixture was gradually heated to 80 °C for 4 h. The reaction mixture was cooled and then poured onto crushed ice. A yellow solid **I** was precipitated, and directly used in the next step without further purification.

Step 2: To a solution of Na_2SO_3 (30 mmol, 3.0 equiv.) in water (30 mL) were added 10 mmol of **I**. The mixture was heated at 100 °C for 10 min. The solvent was removed under reduced pressure. The sodium salt **II** was used in next step without further purification.

Step 3: To a suspension of **II** (7.5 mmol, 1.5 equiv.) in DMF (25 mL) was added 2-bromopropane (5 mmol, 1.0 equiv.). The reaction was stirred at 80 °C for 24 h. The reaction mixture was diluted with EtOAc, washed by brine, dried over Na_2SO_4, filtered, concentrated, and purified by flash column chromatography on silica gel (eluent: EtOAc/Petroleum ether) to give the corresponding product **III**.

Step 4: In a sealed flask, a solution of sulfone **III** (2.6 mmol) in THF (5 mL) and ^{n}BuLi (2.6 mmol), maintained between -70 °C to -60 °C under argon, was stirred for 1 h. A bromine-hexane solution (2.6 mmol of Br_2 and 4.45 mL hexane) was added, and the mixture was stirred for 30 min at -65 °C. Then saturated NH_4Cl (20 mL) was added to quench the reaction and the organic materials were extracted with Et_2O (3 x 20 mL). The combined organic layers were washed with brine (20 mL), dried over Na_2SO_4, concentrated in vacuo, and purified by flash column chromatography on silica gel (eluent: EtOAc/Petroleum ether) to give the corresponding product **IV**.

Step 5: To a suspension of **II** (7.5 mmol, 1.5 equiv.) in DMF (25 mL) were added 1,1-dibromoethane (5 mmol, 1.0 equiv.). The reaction was stirred at 80 °C for 24 h. The reaction mixture was diluted with EtOAc, washed by brine, dried over Na_2SO_4, filtered, concentrated, and purified by flash

column chromatography on silica gel (eluent: EtOAc/Petroleum ether) to give the corresponding product **V**.

Compounds **2l-2q** are newly synthesize.

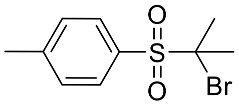

1-((2-bromopropan-2-yl)sulfonyl)-4-methylbenzene (2l): White solid, m.p. 98-99 °C. ^{1}H NMR (400 MHz, $CDCl_3$) δ 7.88 (d, *J* = 8.0 Hz, 2H), 7.36 (d, *J* = 8.0 Hz, 2H), 2.46 (s, 3H), 2.02 (s, 6H); ^{13}C NMR (100 MHz, $CDCl_3$) δ 145.7, 131.7, 130.4, 129.5, 74.2, 28.3, 21.8. FT-IR: ν (cm^{-1}) 2930, 2361, 1593, 1312, 1172, 1075, 816, 712, 690. HRMS [ESI] calcd for $C_{10}H_{13}BrO_2SNa$ [M+Na]$^+$ 298.9712, found 298.9703.

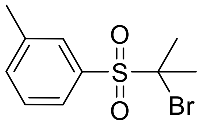

1-((2-bromopropan-2-yl)sulfonyl)-3-methylbenzene (2m): White solid, m.p. 80-81 °C. ^{1}H NMR (400 MHz, $CDCl_3$) δ 7.83-7.78 (m, 2H), 7.52-7.42 (m, 2H), 2.45 (s, 3H), 2.03 (s, 6H); ^{13}C NMR (100 MHz, $CDCl_3$) δ 139.1, 135.3, 133.3, 131.9, 128.9, 128.7, 74.1, 28.3, 21.5. FT-IR: ν (cm^{-1}) 3675, 2985, 2360, 1475, 1313, 1135, 1094, 800, 711, 689. HRMS [ESI] calcd for $C_{10}H_{13}BrO_2SNa$ [M+Na]$^+$ 298.9712, found 298.9711.

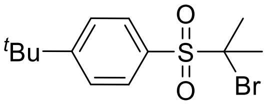

1-((2-bromopropan-2-yl)sulfonyl)-4-(tert-butyl)benzene (2n): White solid, m.p. 155-156 °C. ^{1}H NMR (400 MHz, $CDCl_3$) δ 7.95-7.90 (m, 2H), 7.60-7.55 (m, 2H), 2.04 (s, 6H), 1.36 (s, 9H); ^{13}C NMR (100 MHz, $CDCl_3$) δ 158.6, 131.6, 130.4, 125.9, 74.3, 35.5, 31.2, 28.4. FT-IR: ν (cm^{-1}) 3629, 2972, 2361, 1458, 1320, 1136, 1059, 840, 740. HRMS [ESI] calcd for $C_{13}H_{19}BrO_2SNa$ [M+Na]$^+$ 341.0181, found 341.0192.

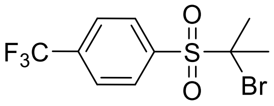

1-((2-bromopropan-2-yl)sulfonyl)-4-(trifluoromethyl)benzene (2o): White solid, m.p. 101-102 °C. ^{1}H NMR (400 MHz, $CDCl_3$) δ 8.17 (d, *J* = 8.0 Hz, 2H), 7.86 (d, *J* = 8.4 Hz, 2H), 2.07 (s, 6H); ^{13}C NMR (100 MHz, $CDCl_3$) δ 137.4, 136.0 (q, J_{C-F} = 33.0 Hz), 132.4, 125.8 (q, J_{C-F} = 3.6 Hz), 124.4 (q, J_{C-F} = 271.6 Hz), 73.6, 28.1; ^{19}F NMR (376 MHz, $CDCl_3$) δ -63.3. FT-IR: ν (cm^{-1}) 3675, 2972, 2362, 1498, 1320, 1136, 1059, 840, 740. HRMS [ESI] calcd for $C_{10}H_{10}BrF_3O_2SNa$ [M+Na]$^+$ 352.9429, found 352.9435.

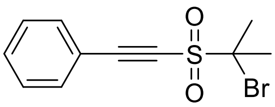

(((2-bromopropan-2-yl)sulfonyl)ethynyl)benzene (2p): White solid, m.p. 81-82 °C. ^{1}H NMR (400 MHz, $CDCl_3$) δ 7.66-7.62 (m, 2H), 7.58-7.52 (m, 1H), 7.47-7.42 (m, 2H), 2.19 (s, 6H); ^{13}C NMR (100 MHz, $CDCl_3$) δ 133.2, 132.2, 129.0, 117.6, 96.6, 79.2, 74.3, 27.6. FT-IR: ν (cm^{-1}) 3554, 2963, 1476, 1345, 1209, 1178, 1012, 908, 789, 767. HRMS [ESI] calcd for $C_{11}H_{11}BrO_2SNa$ [M+Na]$^+$ 308.9555, found 308.9545.

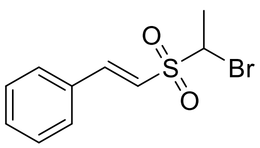

(*E*)-(2-((1-bromoethyl)sulfonyl)vinyl)benzene (2q): White solid, m.p. 70-71 °C. ^{1}H NMR (400 MHz, $CDCl_3$) δ 7.68 (d, *J* = 15.2 Hz, 1H), 7.58-7.54 (m, 2H), 7.51-7.41 (m, 3H), 6.99 (d, *J* = 15.6 Hz, 1H), 4.82 (q, *J* = 6.8 Hz, 1H), 2.04 (d, *J* = 6.8 Hz, 3H); ^{13}C NMR (100 MHz, $CDCl_3$) δ

148.4, 132.1, 132.0, 129.4, 129.0, 120.3, 58.6, 19.2. FT-IR: ν (cm^{-1}) 3545, 2878, 1398, 1211, 1156, 1009, 980, 778, 754. HRMS [ESI] calcd for $C_{10}H_{11}BrO_2SNa$ $[M+Na]^+$ 296.9555, found 296.9558.

4. General procedures for difunctionalization of alkynes

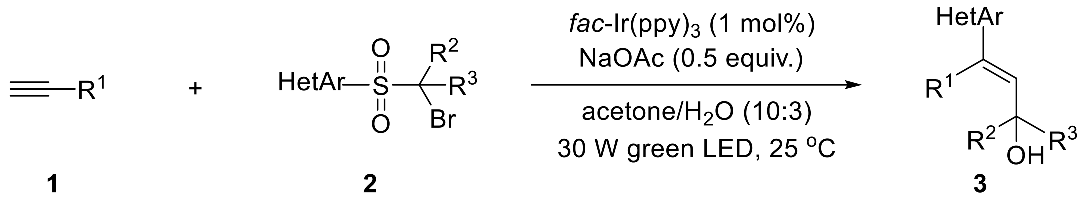

General Procedure A

2 (0.2 mmol), NaOAc (0.1 mmol) and *fac*-Ir(ppy)$_3$ (0.002 mmol) were loaded in a flask, which was subjected to evacuation/ flushing with N_2 for 3 times. Dry acetone (2.0 mL)/ H_2O (0.6 mL) was added to the mixture via syringe. Then alkyne **1** (0.4 mmol) was added to the mixture, which was irradiated by 30 W green LED and stirred at rt until the starting material had been consumed as determined by TLC. The mixture was quenched with H_2O. The aqueous layer was extracted with EtOAc. The combine organic layers were washed with brine, dried over Na_2SO_4, concentrated in vacuo, and purified by flash column chromatography on silica gel (eluent: ethyl acetate/petroleum ether) to give the corresponding product **3**.

General Procedure B

1a (0.4 mmol), **2a** (0.2 mmol), K_2HPO_4 (0.2 mmol), and *fac*-Ir(ppy)$_3$ (0.002 mmol) were loaded in a flask, which was subjected to evacuation/ flushing with N_2 for 3 times. Dry acetone (2.0 mL) /H_2O (0.5 mL) was added to the mixture via syringe, which was irradiated by 456 nm Kessil light and stirred at rt until the starting material had been consumed as determined by TLC. The mixture was quenched with H_2O. The aqueous layer was extracted with EtOAc. The organic layer was washed with brine, dried over Na_2SO_4, concentrated in vacuo, and purified by flash column chromatography on silica gel (eluent: ethyl acetate/petroleum ether) to give the corresponding products **3a**.

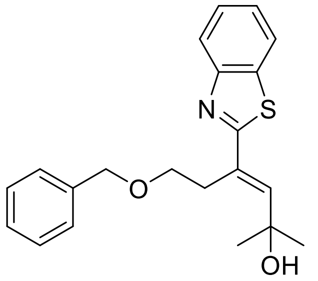

(*E*)-4-(benzo[d]thiazol-2-yl)-6-(benzyloxy)-2-methylhex-3-en-2-ol (3a) was prepared by following **General Procedure A** as a colorless oil (56 mg, 78%, 72 h). ^1H NMR (400 MHz, $CDCl_3$) δ 7.94 (d, J = 8.0 Hz, 1H), 7.81 (d, J = 7.6 Hz, 1H), 7.45-7.40 (m, 1H), 7.36-7.32 (m, 1H), 7.31-7.24 (m, 5H), 6.66 (s, 1H), 4.51 (s, 2H), 4.39 (br, 1H), 3.76 (t, J = 5.2 Hz, 2H), 3.43 (t, J = 5.2 Hz, 2H), 1.36 (s, 6H); ^{13}C NMR (100 MHz, $CDCl_3$) δ 171.4, 154.0, 146.0, 137.5, 134.7, 130.5, 128.6, 128.0, 128.0, 126.1, 125.2, 123.1, 121.4, 73.6, 71.8, 68.7, 31.2, 28.7. FT-IR: ν (cm^{-1}) 3431, 2972, 2341, 1736, 1435, 1359, 1239, 1059, 963, 757, 728. HRMS [ESI] calcd for $C_{21}H_{23}NO_2SNa$ $[M+Na]^+$ 376.1347, found 376.1340.

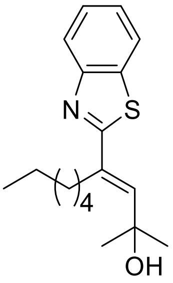

(*E*)-4-(benzo[*d*]thiazol-2-yl)-2-methyldec-3-en-2-ol (3b) was prepared by following **General Procedure A** as a colorless oil (51 mg, 84%, 60 h). ^{1}H NMR (400 MHz, $CDCl_3$) δ 7.99 (d, *J* = 8.0 Hz, 1H), 7.82 (d, *J* = 8.0 Hz, 1H), 7.46-7.40 (m, 1H), 7.36-7.30 (m, 1H), 6.58 (s, 1H), 2.98-2.92 (t, *J* = 8.0 Hz, 2H), 1.82-1.74 (m, 1H), 1.62-1.54 (m, 2H), 1.52 (s, 6H), 1.45-1.37 (m, 2H), 1.31-1.25 (m, 4H), 0.89-0.85 (t, *J* = 6.8 Hz, 3H); ^{13}C NMR (100 MHz, $CDCl_3$) δ 171.6, 153.8, 141.7, 137.0, 134.7, 126.1, 125.1, 123.1, 121.4, 71.8, 31.7, 31.3, 30.0, 29.9, 29.8, 22.7, 14.2. FT-IR: ν (cm^{-1}) 3421, 3063, 2921, 2849, 1435, 1155, 906, 757, 727. HRMS [ESI] calcd for $C_{18}H_{26}NOS$ [M+H]$^+$ 304.1730, found 304.1722.

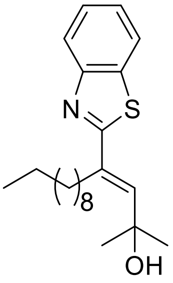

(*E*)-4-(benzo[*d*]thiazol-2-yl)-2-methyltetradec-3-en-2-ol (3c) was prepared by following **General Procedure A** as a colorless oil (50 mg, 70%, 60 h). ^{1}H NMR (400 MHz, CDCl3) δ 7.98 (d, *J* = 8.0 Hz, 1H), 7.82 (d, *J* = 8.0 Hz, 1H), 7.46-7.40 (m, 1H), 7.37-7.31 (m, 1H), 6.58 (s, 1H), 2.98-2.91 (t, *J* = 8.0 Hz, 2H), 1.80-1.71 (m, 1H), 1.61-1.54 (m, 2H), 1.52 (s, 6H), 1.44-1.36 (m, 2H), 1.20-1.33 (m, 12H), 0.87 (t, *J* = 6.8 Hz, 3H). ^{13}C NMR (100 MHz, CDCl3) δ 171.6, 153.9, 141.7, 137.0, 134.8, 126.1, 125.1, 123.1, 121.4, 71.9, 32.0, 31.4, 30.2, 30.0, 29.9, 29.7, 29.6, 29.5, 22.8, 14.3. FT-IR: ν (cm^{-1}) 3455, 2971, 2921, 2849, 1487, 1435, 1155, 1124, 906, 757, 727. HRMS [ESI] calcd for $C_{22}H_{34}NOS$ [M+H]$^+$ 360.2350, found 360.2356.

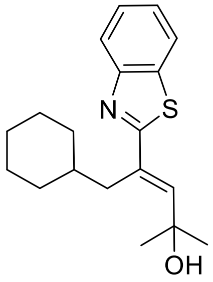

(*E*)-4-(benzo[*d*]thiazol-2-yl)-5-cyclohexyl-2-methylpent-3-en-2-ol (3d) was prepared by following **General Procedure A** as a colorless oil (47 mg, 75%, 56 h). ^{1}H NMR (400 MHz, $CDCl_3$) δ 8.00 (d, *J* = 8.4 Hz, 1H), 7.83 (d, *J* = 8.0 Hz, 1H), 7.47-7.42 (m, 1H), 7.37-7.32 (m, 1H), 6.54 (s, 1H), 2.93 (d, *J* = 7.2 Hz, 2H), 1.76-1.56 (m, 6H), 1.50 (s, 6H), 1.17-1.07 (m, 3H), 1.06-0.94 (m, 2H); ^{13}C NMR (100 MHz, $CDCl_3$) δ 172.2, 153.8, 142.8, 135.0, 134.8, 126.1, 125.0, 123.1, 121.4, 72.1, 37.6, 36.8, 33.6, 31.6, 26.6, 26.5. FT-IR: ν (cm^{-1}) 3409, 2921, 2849, 1487, 1435, 1155, 1124, 906, 757, 727. HRMS [ESI] calcd for $C_{18}H_{26}NOS$ [M+H]$^+$ 316.1730, found 316.1738.

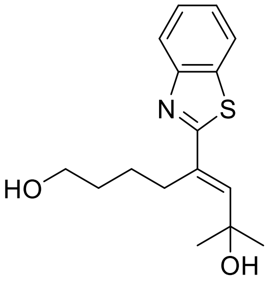

(*E*)-5-(benzo[*d*]thiazol-2-yl)-7-methyloct-5-ene-1,7-diol (3e) was prepared by following **General Procedure A** as a colorless oil (35 mg, 60%, 65 h). ^{1}H NMR (400 MHz, $CDCl_3$) δ 7.96 (d, *J* = 8.0 Hz, 1H), 7.79 (d, *J* = 8.0 Hz, 1H), 7.46-7.39 (m, 1H), 7.37-7.30 (m, 1H), 6.54 (s, 1H), 3.79-3.73 (m, 2H), 3.03-2.97 (m, 2H), 1.76-1.65 (m, 4H), 1.51 (s, 6H); ^{13}C NMR (100 MHz, $CDCl_3$) δ 171.8, 153.5, 142.6, 136.7, 134.4, 126.2, 125.4, 123.0, 121.4, 71.9, 61.7, 31.6, 31.4, 27.7, 25.7. FT-IR: ν (cm^{-1}) 3356, 2930, 2341, 1734, 1456, 1372, 1241, 1152, 1046, 961, 757, 728. HRMS [ESI] calcd for $C_{16}H_{21}NO_2SNa$ [M+Na]$^+$ 314.1185, found 314.1193.

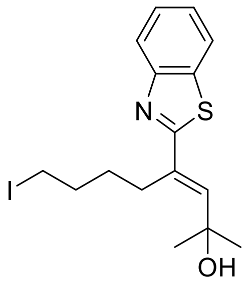

(*E*)-4-(benzo[*d*]thiazol-2-yl)-8-iodo-2-methyloct-3-en-2-ol (3f) was prepared by following **General Procedure A** as a colorless oil (36 mg, 45%, 55 h). ^{1}H NMR (400 MHz, CDCl$_3$) δ 7.98 (d, *J* = 8.0 Hz, 1H), 7.83 (d, *J* = 7.6 Hz, 1H), 7.47-7.42 (m, 1H), 7.37-7.32 (m, 1H), 6.55 (s, 1H), 3.23 (t, *J* = 7.0 Hz, 2H), 3.05-2.98 (m, 2H), 2.00-1.92 (m, 2H), 1.75-1.66 (m, 2H), 1.53 (s, 6H); ^{13}C NMR (100 MHz, CDCl$_3$) δ 171.2, 153.9, 142.2, 136.3, 134.7, 126.2, 125.2, 123.2, 121.5, 72.0, 34.0, 31.5, 30.8, 28.4, 6.9. FT-IR: ν (cm^{-1}) 3300, 2921, 2341, 1636, 1498, 1369, 1275, 994, 771, 727. HRMS [ESI] calcd for $C_{16}H_{21}INOS$ [M+H]$^+$ 402.0383, found 402.0392.

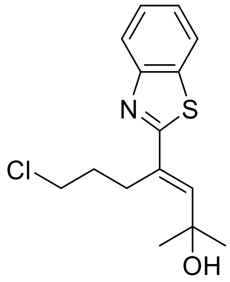

(*E*)-4-(benzo[*d*]thiazol-2-yl)-7-chloro-2-methylhept-3-en-2-ol (3g) was prepared by following **General Procedure A** as a colorless oil (37 mg, 63%, 70 h). ^{1}H NMR (400 MHz, CDCl$_3$) δ 7.97 (d, *J* = 8.0 Hz, 1H), 7.82 (d, *J* = 8.0 Hz, 1H), 7.47-7.42 (m, 1H), 7.37-7.33 (m, 1H), 6.59 (s, 1H), 3.64 (t, *J* = 6.8 Hz, 2H), 3.15 (t, *J* = 8.0 Hz, 2H), 2.16-2.07 (m, 2H), 1.53 (s, 6H); ^{13}C NMR (100 MHz, CDCl$_3$) δ 171.0, 153.9, 143.0, 135.2, 134.6, 126.2, 125.3, 123.2, 121.5, 72.0, 45.4, 32.6, 31.4, 27.0. FT-IR: ν (cm^{-1}) 3265, 2971, 2341, 2187, 1653, 1508, 1457, 1311, 929, 753, 721. HRMS [ESI] calcd for $C_{15}H_{18}ClNOSNa$ [M+Na]$^+$ 318.0690, found 318.0698.

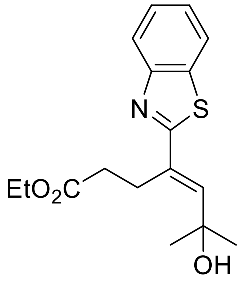

ethyl (*E*)-4-(benzo[*d*]thiazol-2-yl)-6-hydroxy-6-methylhept-4-enoate (3h) was prepared by following **General Procedure A** as a colorless oil (42 mg, 65%, 72 h). ^{1}H NMR (400 MHz, CDCl$_3$) δ 7.96 (d, *J* = 8.0 Hz, 1H), 7.81 (d, *J* = 7.6 Hz, 1H), 7.46-7.41 (m, 1H), 7.36-7.31 (m, 1H), 6.54 (s, 1H), 4.24 (br, 1H), 4.09 (q, *J* = 7.2 Hz, 2H), 3.43 (t, *J* = 6.4 Hz, 2H), 2.79 (t, *J* = 6.4 Hz, 2H), 1.47 (s, 6H), 1.20 (t, *J* = 7.2 Hz, 3H); ^{13}C NMR (100 MHz, CDCl$_3$) δ 175.0, 170.8, 153.9, 144.8, 134.5, 132.7, 126.1, 125.3, 123.2, 121.4, 72.1, 60.9, 32.9, 31.1, 22.8, 14.2. FT-IR: ν (cm^{-1}) 3422, 2975, 2360, 1712, 1435, 1374, 1153, 907, 758, 727. HRMS [ESI] calcd for $C_{17}H_{21}NO_3SNa$ [M+Na]$^+$ 342.1134, found 342.1127.

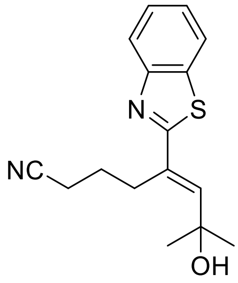

(*E*)-5-(benzo[*d*]thiazol-2-yl)-7-hydroxy-7-methyloct-5-enenitrile (3i) was prepared by following **General Procedure A** as a colorless oil (36 mg, 62%, 72 h). ^{1}H NMR (400 MHz, CDCl$_3$) δ 7.96 (d, *J* = 8.4 Hz, 1H), 7.82 (d, *J* = 7.6 Hz, 1H), 7.47-7.42 (m, 1H), 7.38-7.33 (m, 1H), 6.54 (s, 1H), 3.16 (t, *J* = 7.6 Hz, 2H), 2.44 (t, *J* = 7.2 Hz, 2H), 2.06-1.97 (m, 2H), 1.52 (s, 6H); ^{13}C NMR (100 MHz, CDCl$_3$) δ 170.6, 153.8, 143.3, 134.7, 134.5, 126.2, 125.4, 123.2, 121.4, 120.1, 72.0, 31.6, 28.2, 25.4, 17.3. FT-IR: ν (cm^{-1}) 3446, 2971, 2925, 2248, 1434, 1362, 1153, 958, 908, 758, 727. HRMS [ESI] calcd for $C_{16}H_{18}N_2OSNa$ [M+Na]$^+$ 309.1032, found 309.1035.

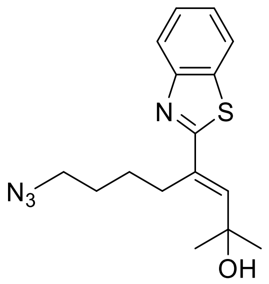

(*E*)-8-azido-4-(benzo[*d*]thiazol-2-yl)-2-methyloct-3-en-2-ol (3j) was prepared by following **General Procedure A** as a colorless oil (44 mg, 70%, 72 h). ^{1}H NMR (400 MHz, CDCl$_3$) δ 7.98 (d, *J* = 8.0 Hz, 1H), 7.82 (d, *J* = 7.6 Hz, 1H), 7.47-7.42 (m, 1H), 7.37-7.32 (m, 1H), 6.55 (s, 1H), 3.30 (t, *J* = 6.4 Hz, 2H), 3.05-2.99 (m, 2H), 1.77-1.65 (m, 4H), 1.52 (s, 6H); ^{13}C NMR (100 MHz, CDCl$_3$) δ 171.2, 153.9, 142.2, 136.4, 134.7, 126.2, 125.2, 123.2, 121.4, 71.9, 51.3, 31.5, 29.1, 28.9, 27.0. FT-IR: ν (cm^{-1}) 3421, 2930, 2341, 2092, 1434, 1245, 1152, 907, 758, 727. HRMS [ESI] calcd for $C_{16}H_{20}N_4OSNa$ [M+Na]$^+$ 339.1250, found 339.1254.

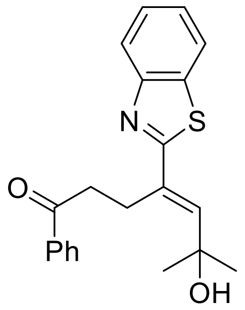

(*E*)-4-(benzo[*d*]thiazol-2-yl)-6-hydroxy-6-methyl-1-phenylhept-4-en-1-one (3k) was prepared by following **General Procedure A** as a colorless oil (52 mg, 74%, 72 h). ^{1}H NMR (400 MHz, CDCl$_3$) δ 7.98 (d, *J* = 8.0 Hz, 1H), 7.96-7.93 (m, 2H), 7.80 (d, *J* = 8.0 Hz, 1H), 7.54-7.38 (m, 4H), 7.37-7.32 (m, 1H), 6.55 (s, 1H), 3.58-3.47 (m, 4H), 1.53 (s, 6H); ^{13}C NMR (100 MHz, CDCl$_3$) δ 201.7, 171.2, 153.9, 144.6, 136.9, 134.5, 133.4, 130.3, 128.7, 128.4, 126.1, 125.3, 123.1, 121.4, 72.2, 37.6, 31.2, 22.6. FT-IR: ν (cm^{-1}) 3420, 2972, 2341, 1674, 1449, 1361, 1206, 1151, 907, 728. HRMS [ESI] calcd for $C_{21}H_{21}NO_2SNa$ [M+Na]$^+$ 374.1185, found 374.1175.

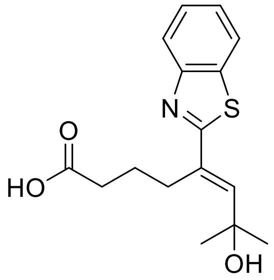

(*E*)-5-(benzo[*d*]thiazol-2-yl)-7-hydroxy-7-methyloct-5-enoic acid (3l) was prepared by following **General Procedure A** as a colorless oil (40 mg, 65%, 65 h). ^{1}H NMR (400 MHz, CDCl$_3$) δ 7.97 (d, *J* = 8.0 Hz, 1H), 7.81 (d, *J* = 8.0 Hz, 1H), 7.47-7.40 (m, 1H), 7.37-7.31 (m, 1H), 6.54 (s, 1H), 3.08 (t, *J* = 7.8 Hz, 2H), 2.49 (t, *J* = 7.0 Hz, 2H), 2.01-1.91 (m, 2H), 1.50 (s, 6H); ^{13}C NMR (100 MHz, CDCl$_3$) δ 177.8, 171.6, 153.5, 143.3, 135.3, 134.5, 126.3, 125.4, 123.1, 121.5, 72.0, 33.9, 31.4, 28.2, 24.7. FT-IR: ν (cm^{-1}) 3318, 2985, 1688, 1489, 1254, 1160, 980, 906, 758, 727. HRMS [ESI] calcd for $C_{16}H_{20}NO_3S$ [M+H]$^+$ 306.1158, found 306.1151.

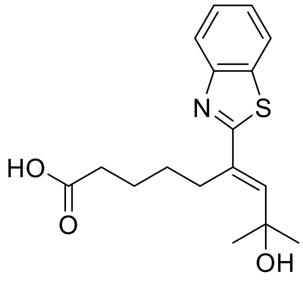

(*E*)-6-(benzo[*d*]thiazol-2-yl)-8-hydroxy-8-methylnon-6-enoic acid (3m) was prepared by following **General Procedure A** as a colorless oil (39 mg, 61%, 72 h). ^{1}H NMR (400 MHz, CDCl$_3$) δ 7.98 (d, *J* = 8.0 Hz, 1H), 7.81 (d, *J* = 8.0 Hz, 1H), 7.46-7.40 (m, 1H), 7.36-7.31 (m, 1H), 6.55 (s, 1H), 3.00 (t, *J* = 8.0 Hz, 2H), 2.39 (t, *J* = 7.2 Hz, 2H), 1.81-1.71 (m, 2H), 1.69-1.59 (m, 2H), 1.50 (s, 6H); ^{13}C NMR (100 MHz, CDCl$_3$) δ 179.1, 171.6, 153.7, 142.3, 136.3, 134.6, 126.2, 125.2, 123.1, 121.4, 72.0, 33.8, 31.4, 29.1, 29.1, 25.0. FT-IR: ν (cm^{-1}) 3245, 2972, 1706, 1456, 1212, 1157, 905, 758, 725. HRMS [ESI] calcd for $C_{17}H_{22}NO_3S$ [M+H]$^+$ 320.1315, found 320.1322.

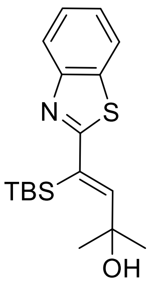

(*Z*)-4-(benzo[*d*]thiazol-2-yl)-4-(tert-butyldimethylsilyl)-2-methylbut-3-en-2-ol (3n) was prepared by following **General Procedure A** as a colorless oil (26 mg, 40%, 50 h). ^{1}H NMR (500 MHz, $CDCl_3$) δ 7.98 (d, *J* = 8.0 Hz, 1H), 7.84 (d, *J* = 8.0 Hz, 1H), 7.48-7.44 (m, 1H), 7.38-7.34 (m, 1H), 6.42 (s, 1H), 4.32 (br, s, 1H), 1.29 (s, 6H), 0.89 (s, 9H), 0.22 (s, 6H); ^{13}C NMR (125 MHz, $CDCl_3$) δ 171.3, 157.0, 152.3, 135.7, 134.0, 126.3, 125.1, 122.8, 121.4, 72.1, 30.3, 27.0, 18.0, 5.0. FT-IR: ν (cm-1) 3385, 2927, 2855, 1435, 1112, 832, 778, 756. HRMS [ESI] calcd for $C_{18}H_{27}NOSSiNa$ $[M+Na]^+$ 356.1475, found 356.1477.

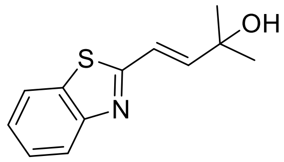

(*E*)-4-(benzo[*d*]thiazol-2-yl)-2-methylbut-3-en-2-ol (3o) was prepared by following **General Procedure A** as a colorless oil (9 mg, 20%, 12 h). 1H NMR (400 MHz, $CDCl_3$) δ 7.98 (d, J = 7.6 Hz, 1H), 7.84 (d, J = 8.0 Hz, 1H), 7.49-7.42 (m, 1H), 7.40-7.32 (m, 1H), 6.96 (d, J = 16.0 Hz, 1H), 6.85 (d, J = 16.0 Hz, 1H), 1.47 (s, 6H); 13C NMR (100 MHz, CDCl3) δ 166.9, 153.8, 147.4, 134.5, 126.4, 125.5, 123.1, 121.7, 121.3, 71.3, 29.7. FT-IR: ν (cm-1) 3348, 2970, 2924, 1708, 1434, 1231, 1153, 962, 757, 727. HRMS [ESI] calcd for $C_{12}H_{13}NOSNa$ $[M+Na]^+$ 242.0610, found 242.0614.

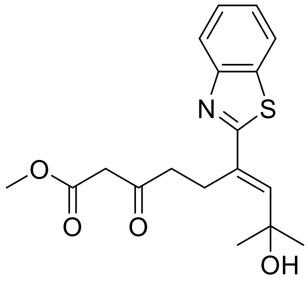

methyl (*E*)-6-(benzo[*d*]thiazol-2-yl)-8-hydroxy-8-methyl-3-oxonon-6-enoate (3p) was prepared by following **General Procedure A** as a colorless oil (43 mg, 62%, 65 h). ^{1}H NMR (400 MHz, $CDCl_3$) δ 7.95 (d, *J* = 8.0 Hz, 1H), 7.81 (d, *J* = 8.0 Hz, 1H), 7.47-7.41 (m, 1H), 7.38-7.32 (m, 1H), 6.51 (s, 1H), 3.64 (s, 3H), 3.59 (br, 1H), 3.43 (s, 2H), 3.38 (t, *J* = 6.8 Hz, 2H), 3.03 (t, *J* = 6.8 Hz, 2H), 1.48 (s, 6H); ^{13}C NMR (100 MHz, $CDCl_3$) δ 204.4, 170.8, 167.5, 153.9, 144.5, 134.4, 133.1, 126.2, 125.4, 123.1, 121.4, 72.1, 52.5, 49.3, 42.2, 31.2, 22.3. FT-IR: ν (cm^{-1}) 2921, 2852, 1743, 1459, 1376, 1258, 1015, 796, 722. HRMS [ESI] calcd for $C_{18}H_{21}NO_4SNa$ $[M+Na]^+$ 370.1083, found 370.1076.

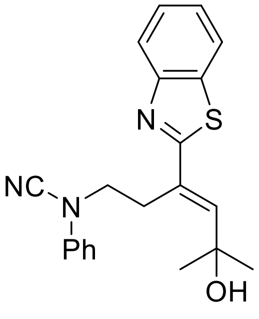

(*E*)-*N*-(3-(benzo[*d*]thiazol-2-yl)-5-hydroxy-5-methylhex-3-en-1-yl)-N-phenylcyanamide (3q) was prepared by following **General Procedure A** as a colorless oil (48 mg, 67%, 72 h). ^{1}H NMR (400 MHz, $CDCl_3$) δ 7.99 (d, *J* = 8.0 Hz, 1H), 7.83 (d, *J* = 8.0 Hz, 1H), 7.50-7.45 (m, 1H), 7.41-7.35 (m, 5H), 7.12-7.06 (m, 1H), 6.59 (s, 1H), 4.03-3.98 (m, 2H), 3.56 (t, *J* = 8.0 Hz, 2H), 1.53 (s, 6H); ^{13}C NMR (100 MHz, $CDCl_3$) δ 170.4, 153.8, 144.9, 140.0, 134.6, 131.8, 129.7, 126.3, 125.6, 123.3, 123.3, 121.5, 115.9, 114.1, 72.2, 49.5, 31.7, 27.7. FT-IR: ν (cm^{-1}) 3475, 2966, 2927, 2341, 2221, 1599, 1557, 1394, 1311, 1057, 687, 673. HRMS [ESI] calcd for $C_{21}H_{21}N_3OSNa$ $[M+Na]^+$ 386.1298, found 386.1289.

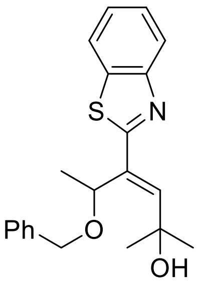

(*E*)-4-(benzo[*d*]thiazol-2-yl)-5-(benzyloxy)-2-methylhex-3-en-2-ol (3r) was prepared by following **General Procedure A** as a colorless oil (35 mg, 50%, 45 h). ^{1}H NMR (400 MHz, $CDCl_3$) δ 7.97 (d, *J* = 7.6 Hz, 1H), 7.83 (d, *J* = 7.2 Hz, 1H), 7.47-7.42 (m, 1H), 7.40-7.30 (m, 6H), 6.68 (s, 1H), 5.44 (q, *J* = 6.8 Hz, 1H), 4.94 (br, 1H), 4.75 (d, *J* = 12.0 Hz, 1H), 4.65 (d, *J* = 12.0 Hz, 1H), 1.66 (d, *J* = 6.4 Hz, 3H), 1.47 (s, 3H), 1.42 (s, 3H); ^{13}C NMR (100 MHz, $CDCl_3$) δ 169.6, 153.5, 145.8, 137.5, 135.0, 134.7, 128.7, 128.0, 127.9, 126.2, 125.3, 123.2, 121.4, 74.8, 71.7, 71.5, 31.4, 30.9, 21.6. FT-IR: ν (cm^{-1}) 3394, 2974, 2927, 1454, 1369, 1314, 1158, 1093, 907, 758, 728, 696. HRMS [ESI] calcd for $C_{21}H_{23}NO_2SNa$ [M+Na]$^+$ 376.1347, found 376.1341.

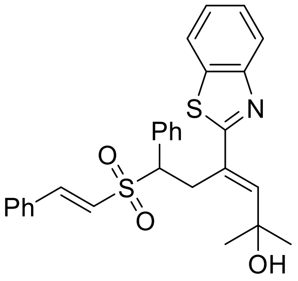

(*E*)-4-(benzo[*d*]thiazol-2-yl)-2-methyl-6-phenyl-6-(((*E*)-styryl)sulfonyl)hex-3-en-2-ol (3s) was prepared by following **General Procedure A** as a colorless oil (44 mg, 45%, 50 h). ^{1}H NMR (400 MHz, $CDCl_3$) δ 7.87 (d, *J* = 8.0 Hz, 1H), 7.79 (d, *J* = 8.0 Hz, 1H), 7.50-7.27 (m, 13H), 6.52 (d, *J* = 15.6 Hz, 1H), 6.47 (s, 1H), 4.92 (dd, *J* = 7.6, 6.8 Hz, 1H), 4.55 (dd, *J* = 14.4, 7.6 Hz, 1H), 3.73 (dd, *J* = 14.4, 6.8 Hz, 1H), 3.06 (br, 1H), 1.52 (s, 3H), 1.35 (s, 3H); ^{13}C NMR (100 MHz, $CDCl_3$) δ 170.8, 153.8, 145.7, 145.0, 134.5, 133.8, 132.6, 131.2, 130.4, 130.2, 129.1, 129.1, 128.9, 128.6, 126.1, 125.4, 124.5, 123.2, 121.5, 72.6, 69.3, 31.6, 30.4, 29.0. FT-IR: ν (cm^{-1}) 3506, 2924, 2341, 1683, 1494, 1361, 1220, 970, 769, 732, 699. HRMS [ESI] calcd for $C_{28}H_{27}NO_3S_2Na$ [M+Na]$^+$ 512.1325, found 512.1326.

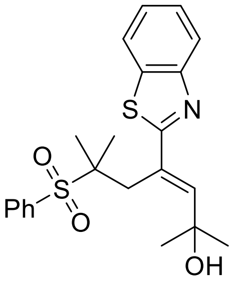

(*E*)-4-(benzo[*d*]thiazol-2-yl)-2,6-dimethyl-6-(phenylsulfonyl)hept-3-en-2-ol (3t) was prepared by following **General Procedure A** as a colorless oil (49 mg, 60%, 55 h). ^{1}H NMR (400 MHz, $CDCl_3$) δ 7.91-7.87 (m, 3H), 7.82 (d, *J* = 8.0 Hz, 1H), 7.68-7.62 (m, 1H), 7.57-7.52 (m, 2H), 7.45-7.40 (m, 1H), 7.37-7.32 (m, 1H), 6.35 (s, 1H), 3.82 (s, 2H), 3.14 (br, 1H), 1.47 (s, 6H), 1.27 (s, 6H); ^{13}C NMR (100 MHz, $CDCl_3$) δ 172.7, 153.4, 146.9, 135.6, 134.8, 133.8, 130.8, 129.4, 128.8, 126.2, 125.3, 123.1, 121.5, 72.8, 64.2, 32.3, 31.2, 23.3. FT-IR: ν (cm^{-1}) 3381, 2924, 2331, 1719, 1464, 1362, 1159, 1123, 1076, 762, 729, 692. HRMS [ESI] calcd for $C_{22}H_{25}NO_3S_2Na$ [M+Na]$^+$ 438.1174, found 438.1169.

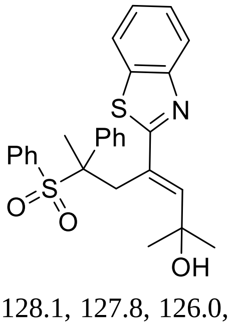

(*E*)-4-(benzo[*d*]thiazol-2-yl)-2-methyl-6-phenyl-6-(phenylsulfonyl)hept-3-en-2-ol (3u) was prepared by following **General Procedure A** as a colorless oil (47 mg, 50%, 55 h). ^{1}H NMR (400 MHz, $CDCl_3$) δ 7.74 (d, *J* = 8.0 Hz, 1H), 7.71 (d, *J* = 8.0 Hz, 1H), 7.46-7.20 (m, 9H), 7.17-7.09 (m, 3H), 6.36 (s, 1H), 5.10 (d, *J* = 14.4 Hz, 1H), 3.85 (d, *J* = 14.8 Hz, 1H), 3.63 (br, 1H), 1.61 (s, 3H), 1.59 (s, 3H), 1.45 (s, 3H); ^{13}C NMR (100 MHz, $CDCl_3$) δ 172.4, 153.2, 147.5, 137.2, 135.4, 134.8, 133.4, 130.4, 129.4, 129.0, 128.4, 128.1, 127.8, 126.0, 125.1, 123.0, 121.4, 72.9, 70.2, 33.3, 31.3, 30.9, 20.3. FT-IR: ν (cm^{-1}) 3515, 2976, 2341, 1750, 1489, 1376, 1244, 1140, 903, 727, 689. HRMS [ESI] calcd for $C_{27}H_{28}NO_3S_2$ [M+H]$^+$ 478.1505, found 478.1515.

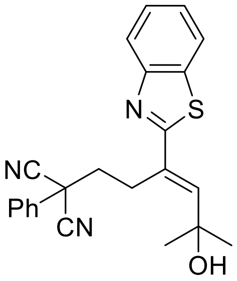

(*E*)-2-(3-(benzo[*d*]thiazol-2-yl)-5-hydroxy-5-methylhex-3-en-1-yl)-2-phenylmalononitrile (3v) was prepared by following **General Procedure A** as a colorless oil (52 mg, 67%, 65 h). ^{1}H NMR (400 MHz, $CDCl_3$) δ 7.94 (d, *J* = 8.0 Hz, 1H), 7.81 (d, *J* = 8.0 Hz, 1H), 7.66-7.62 (m, 2H), 7.53-7.42 (m, 4H), 7.38-7.33 (m, 1H), 6.54 (s, 1H), 3.34-3.29 (m, 2H), 2.68-2.62 (m, 2H), 1.50 (s, 6H); ^{13}C NMR (100 MHz, $CDCl_3$) δ 169.8, 153.9, 143.4, 134.6, 133.1, 132.2, 130.0, 129.7, 126.3, 126.2, 125.6, 123.5, 121.5, 115.2, 72.1, 42.2, 41.6, 31.7, 25.8. FT-IR: ν (cm^{-1}) 3564, 3309, 2976, 2341, 1492, 1435, 1280, 980, 759, 727, 691. HRMS [ESI] calcd for $C_{23}H_{21}N_3OSNa$ [M+Na]$^+$ 410.1298, found 410.1306.

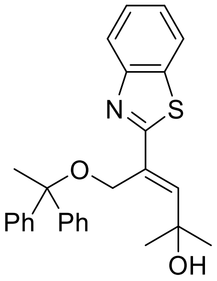

(*E*)-4-(benzo[*d*]thiazol-2-yl)-5-(1,1-diphenylethoxy)-2-methylpent-3-en-2-ol (3w) was prepared by following **General Procedure A** as a colorless oil (43 mg, 50%, 60 h). ^{1}H NMR (400 MHz, $CDCl_3$) δ 7.88 (d, *J* = 7.6 Hz, 1H), 7.82 (d, *J* = 8.0 Hz, 1H), 7.46-7.41 (m, 1H), 7.39-7.34 (m, 5H), 7.33-7.26 (m, 4H), 7.25-7.20 (m, 2H), 6.80 (s, 1H), 4.73 (s, 2H), 4.05 (br, 1H), 2.04 (s, 3H), 1.50 (s, 6H); ^{13}C NMR (100 MHz, $CDCl_3$) δ 170.0, 153.6, 147.0, 145.7, 135.0, 130.8, 128.2, 127.3, 127.1, 126.1, 125.2, 123.1, 121.4, 82.8, 72.2, 60.0, 30.9, 26.1. FT-IR: ν (cm^{-1}) 3234, 2878, 1765, 1323, 1234, 1145, 995, 756, 736. HRMS [ESI] calcd for $C_{27}H_{27}NO_2SNa$ [M+Na]$^+$ 452.1655, found 452.1648.

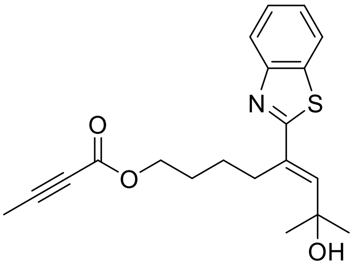

(*E*)-5-(benzo[*d*]thiazol-2-yl)-7-hydroxy-7-methyloct-5-en-1-yl but-2-ynoate (3x) was prepared by following **General Procedure A** as a colorless oil (50 mg, 70%, 72 h). ^{1}H NMR (400 MHz, $CDCl_3$) δ 7.98 (d, *J* = 8.0 Hz, 1H), 7.82 (d, *J* = 8.0 Hz, 1H), 7.46-7.41 (m, 1H), 7.37-7.31 (m, 1H), 6.54 (s, 1H), 4.19 (t, *J* = 6.8 Hz, 2H), 3.03 (t, *J* = 7.6 Hz, 2H), 1.95 (s, 3H), 1.83-1.75 (m, 2H), 1.72-1.65 (m, 2H), 1.51 (s, 6H); ^{13}C NMR (100 MHz, $CDCl_3$) δ 171.2, 154.0, 153.9, 142.3, 136.4, 134.7, 126.1, 125.2, 123.2, 121.4, 85.5, 72.6, 71.9, 65.8, 31.5, 28.9, 28.6, 26.0, 3.9. FT-IR: ν (cm^{-1}) 3423, 2969, 2360, 2242, 1701, 1434, 1254, 1066, 908, 756, 727. HRMS [ESI] calcd for $C_{20}H_{23}NO_3SNa$ [M+Na]$^+$ 380.1291, found 380.1299.

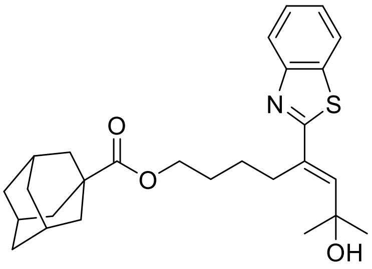

(*E*)-5-(benzo[*d*]thiazol-2-yl)-7-hydroxy-7-methyloct-5-en-1-yl (1s,3s)-adamantane-1-carboxylate (3y) was prepared by following **General Procedure A** as a colorless oil (54 mg, 60%, 72 h). ^{1}H NMR (400 MHz, $CDCl_3$) δ 7.96 (d, *J* = 8.0 Hz, 1H), 7.81 (d, *J* = 8.0 Hz, 1H), 7.46-7.40 (m, 1H), 7.36-7.30 (m, 1H), 6.56 (s, 1H), 4.08 (t, *J* = 6.0 Hz, 2H), 3.06-2.99 (m, 2H), 1.94-1.90 (m, 3H), 1.82-1.79 (m, 6H), 1.75-1.58 (m, 10H), 1.51 (s, 6H); ^{13}C NMR (100 MHz, $CDCl_3$) δ 177.9, 171.3, 153.9, 142.3, 136.5, 134.7, 126.1, 125.2, 123.1, 121.4, 71.8, 63.8, 40.8, 38.9, 36.6, 31.4, 29.0, 29.0, 28.0, 26.0. FT-IR: ν (cm^{-1}) 3454, 2906, 2360, 1704, 1454, 1238, 1077, 907, 727. HRMS [ESI] calcd for $C_{27}H_{36}NO_3S$ [M+H]$^+$ 454.2410, found 454.2420.

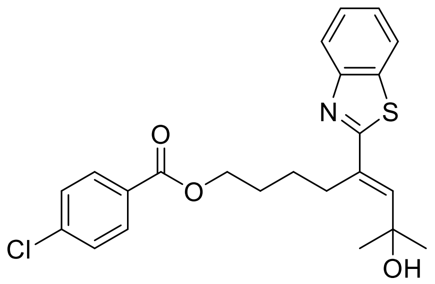

(*E*)-5-(benzo[*d*]thiazol-2-yl)-7-hydroxy-7-methyloct-5-en-1-yl 4-chlorobenzoate (3z) was prepared by following **General Procedure A** as a colorless oil (66 mg, 77%, 72 h). ^{1}H NMR (400 MHz, $CDCl_3$) δ 7.94 (d, *J* = 8.0 Hz, 1H), 7.92-7.88 (m, 2H), 7.80 (d, *J* = 8.0 Hz, 1H), 7.46-7.41 (m, 1H), 7.37-7.30 (m, 3H), 6.56 (s, 1H), 4.35 (t, *J* = 6.4 Hz, 2H), 3.08 (t, *J* = 7.6 Hz, 2H), 1.93-1.84 (m, 2H), 1.80-1.72 (m, 2H), 1.52 (s, 6H); ^{13}C NMR (100 MHz, $CDCl_3$) δ 171.2, 165.9, 153.8, 142.3, 139.3, 136.5, 134.6, 131.0, 129.0, 128.7, 126.2, 125.2, 123.1, 121.4, 71.9, 65.0, 31.5, 28.9, 28.9, 26.0. FT-IR: ν (cm^{-1}) 3503, 2972, 2341, 1716, 1487, 1270, 1090, 1014, 757, 727. HRMS [ESI] calcd for $C_{23}H_{24}ClNO_3SNa$ $[M+Na]^+$ 452.1058, found 452.1051.

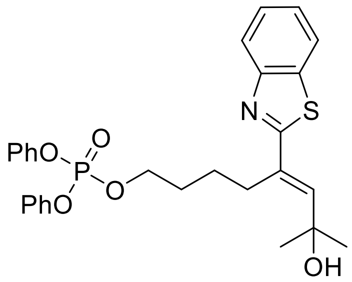

(*E*)-5-(benzo[*d*]thiazol-2-yl)-7-hydroxy-7-methyloct-5-en-1-yl diphenyl phosphate (3aa) was prepared by following **General Procedure A** as a colorless oil (73 mg, 70%, 72 h). ^{1}H NMR (400 MHz, $CDCl_3$) δ 7.96 (d, *J* = 8.0 Hz, 1H), 7.81 (d, *J* = 7.6 Hz, 1H), 7.45-7.40 (m, 1H), 7.36-7.31 (m, 1H), 7.30-7.25 (m, 4H), 7.20-7.17 (m, 4H), 7.14 (t, *J* = 7.6 Hz, 2H), 6.52 (s, 1H), 4.30 (dd, *J* = 14.4, 6.4 Hz, 2H), 3.06-3.00 (m, 2H), 2.18 (br, 1 H), 1.87-1.78 (m, 2H), 1.73-1.64 (m, 2H), 1.47 (s, 6H); ^{13}C NMR (100 MHz, $CDCl_3$) δ 171.4, 153.9, 150.6 (d, $J_{C\text{-}P}$ = 7.1 Hz), 142.5 (d, $J_{C\text{-}P}$ = 3.3 Hz), 136.0, 134.6, 129.8, 126.1, 125.4, 125.2, 123.1, 121.4, 120.1 (d, $J_{C\text{-}P}$ = 5.0 Hz), 71.7, 69.2 (d, $J_{C\text{-}P}$ = 6.5 Hz), 31.4, 30.3 (d, $J_{C\text{-}P}$ = 6.5 Hz), 28.6, 25.4; ^{31}P NMR (162 MHz, $CDCl_3$) δ -11.8. FT-IR: ν (cm^{-1}) 3445, 2921, 1487, 1435, 1155, 1124, 906, 757, 727. HRMS [ESI] calcd for $C_{28}H_{30}NO_5PSNa$ $[M+Na]^+$ 546.1475, found 546.1480.

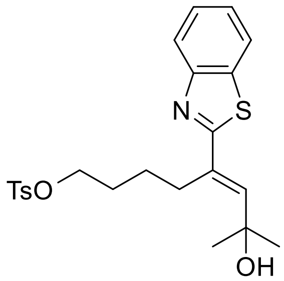

(*E*)-5-(benzo[*d*]thiazol-2-yl)-7-hydroxy-7-methyloct-5-en-1-yl 4-methylbenzenesulfonate (3ab) was prepared by following **General Procedure A** as a colorless oil (65 mg, 73%, 72 h). ^{1}H NMR (400 MHz, $CDCl_3$) δ 7.95 (d, *J* = 8.0 Hz, 1H), 7.82 (d, *J* = 7.2 Hz, 1H), 7.76 (d, *J* = 8.4 Hz, 2H), 7.46-7.41 (m, 1H), 7.37-7.32 (m, 1H), 7.28 (d, *J* = 8.0 Hz, 2H), 6.51 (s, 1H), 4.06 (t, *J* = 6.4 Hz, 2H), 2.97 (t, *J* = 8.0 Hz, 2H), 2.40 (s, 3H), 1.80-1.72 (m, 2H), 1.66-1.58 (m, 2H), 1.49 (s, 6H); ^{13}C NMR (100 MHz, $CDCl_3$) δ 171.2, 153.8, 144.7, 142.4, 136.1, 134.6, 133.3, 129.9, 128.0, 126.2, 125.3, 123.2, 121.4, 71.9, 70.6, 31.5, 29.0, 28.6, 25.6, 21.7. FT-IR: ν (cm^{-1}) 3332, 3069, 2965, 1738, 1491, 1374, 1230, 1191, 1029, 822, 775, 678. HRMS [ESI] calcd for $C_{23}H_{27}NO_4S_2Na$ $[M+Na]^+$ 468.1274, found 468.1289.

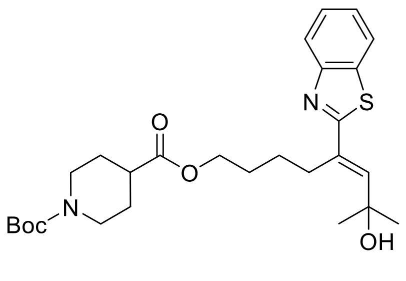

(*E*)-4-(5-(benzo[*d*]thiazol-2-yl)-7-hydroxy-7-methyloct-5-en-1-yl) 1-(tert-butyl) piperidine-1,4-dicarboxylate (3ac) was prepared by following **General Procedure A** as a colorless oil (60 mg, 60%, 66 h). ^{1}H NMR (400 MHz, $CDCl_3$) δ 7.96 (d, *J* = 8.0 Hz, 1H), 7.82 (d, *J* = 8.0 Hz, 1H), 7.47-7.42 (m, 1H), 7.37-7.32 (m, 1H), 6.55 (s, 1H), 4.12 (t, *J* = 6.4 Hz, 2H), 4.03-3.90 (m, 2H), 3.06-2.99 (m, 2H), 2.77 (t, *J* = 12.0

Hz, 2H), 2.42-2.33 (m, 1H), 1.83-1.62 (m, 8H), 1.51 (s, 6H), 1.45 (s, 9H); ^{13}C NMR (100 MHz, $CDCl_3$) δ 174.8, 171.3, 154.8, 153.9, 142.3, 136.5, 134.7, 126.2, 125.3, 123.1, 121.4, 79.7, 71.9, 64.5, 41.3, 31.5, 29.8, 29.0, 28.6, 28.1, 26.0. FT-IR: ν (cm^{-1}) 3446, 2972, 2341, 1729, 1673, 1428, 1365, 1158, 909, 759, 728. HRMS [ESI] calcd for $C_{27}H_{38}N_2O_5SNa$ [M+Na]$^+$ 525.2394, found 525.2381.

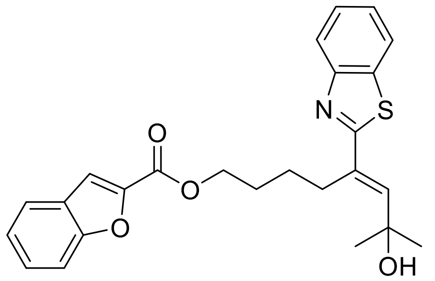

(*E*)-5-(benzo[*d*]thiazol-2-yl)-7-hydroxy-7-methyloct-5-en-1-yl benzofuran-2-carboxylate (3ad) was prepared by following **General Procedure A** as a colorless oil (59 mg, 68%, 72 h). ^{1}H NMR (400 MHz, $CDCl_3$) δ 7.95 (d, *J* = 8.0 Hz, 1H), 7.78 (d, *J* = 8.0 Hz, 1H), 7.63 (d, *J* = 7.6 Hz, 1H), 7.56 (d, *J* = 8.4 Hz, 1H), 7.45-7.38 (m, 3H), 7.34-7.26 (m, 2H), 6.56 (s, 1H), 4.42 (t, *J* = 6.4 Hz, 2H), 3.10 (t, *J* = 7.6 Hz, 2H), 1.96-1.88 (m, 2H), 1.82-1.75 (m, 2H), 1.52 (s, 6H); ^{13}C NMR (100 MHz, $CDCl_3$) δ 171.3, 159.8, 155.8, 153.8, 145.7, 142.4, 136.4, 134.6, 127.6, 127.1, 126.1, 125.2, 123.8, 123.1, 122.9, 121.4, 113.9, 112.5, 71.9, 65.3, 31.5, 28.9, 28.8, 26.0. FT-IR: ν (cm^{-1}) 3420, 2969, 2341, 1717, 1562, 1295, 1177, 907, 726. HRMS [ESI] calcd for $C_{25}H_{25}NO_4SNa$ [M+Na]$^+$ 458.1397, found 458.1407.

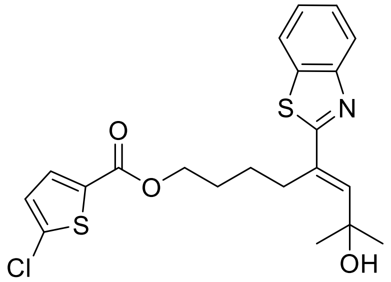

(*E*)-5-(benzo[*d*]thiazol-2-yl)-7-hydroxy-7-methyloct-5-en-1-yl 5-chlorothiophene-2-carboxylate (3ae) was prepared by following **General Procedure A** as a colorless oil (65 mg, 75%, 72 h). ^{1}H NMR (400 MHz, $CDCl_3$) δ 7.95 (d, *J* = 8.4 Hz, 1H), 7.80 (d, *J* = 7.6 Hz, 1H), 7.51 (d, *J* = 4.0 Hz, 1H), 7.46-7.40 (m, 1H), 7.36-7.30 (m, 1H), 6.86 (d, *J* = 4.0 Hz, 1H), 6.56 (s, 1H), 4.31 (t, *J* = 6.4 Hz, 2H), 3.07 (t, *J* = 7.6 Hz, 2H), 1.89-1.81 (m, 2H), 1.78-1.69 (m, 2H), 1.52 (s, 6H); ^{13}C NMR (100 MHz, $CDCl_3$) δ 171.2, 161.4, 153.9, 142.3, 137.3, 136.4, 134.6, 133.0, 132.2, 127.3, 126.1, 125.2, 123.2, 121.4, 71.9, 65.2, 31.5, 28.9, 28.8, 26.0. FT-IR: ν (cm^{-1}) 3395, 2969, 2360, 1705, 1423, 1252, 1090, 959, 728. HRMS [ESI] calcd for $C_{21}H_{22}ClNO_3S_2Na$ [M+Na]$^+$ 458.0622, found 458.0635.

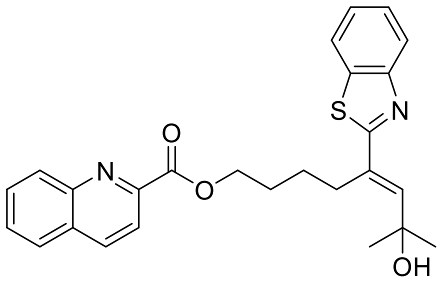

(*E*)-5-(benzo[*d*]thiazol-2-yl)-7-hydroxy-7-methyloct-5-en-1-yl quinoline-2-carboxylate (3af) was prepared by following **General Procedure A** as a colorless oil (69 mg, 78%, 72 h). ^{1}H NMR (400 MHz, $CDCl_3$) δ 8.28 (d, *J* = 8.4 Hz, 1H), 8.20 (d, *J* = 8.8 Hz, 1H), 8.09 (d, *J* = 8.8 Hz, 1H), 7.94 (d, *J* = 8.0 Hz, 1H), 7.84 (d, *J* = 8.4 Hz, 1H), 7.79-7.73 (m, 2H), 7.64-7.60 (m, 1H), 7.42-7.37 (m, 1H), 7.32-7.27 (m, 1H), 6.55 (s, 1H), 4.53 (t, *J* = 6.8 Hz, 2H), 3.11 (t, *J* = 7.6 Hz, 2H), 2.12 (br, 1H), 2.03-1.95 (m, 2H), 1.85-1.76 (m, 2H), 1.51 (s, 6H); ^{13}C NMR (100 MHz, $CDCl_3$) δ 171.3, 165.5, 153.8, 148.3, 147.7, 142.4, 137.3, 136.3, 134.6, 130.8, 130.3, 129.4, 128.6, 127.6, 126.1, 125.1, 123.1, 121.4, 121.1, 71.8, 66.0, 31.5, 28.9, 28.8, 26.1. FT-IR: ν (cm^{-1}) 3421, 2969, 2360, 1717, 1313, 1243, 1136, 907, 758, 726. HRMS [ESI] calcd for $C_{26}H_{26}N_2O_3SK$ [M+K]$^+$ 485.1301, found 485.1304.

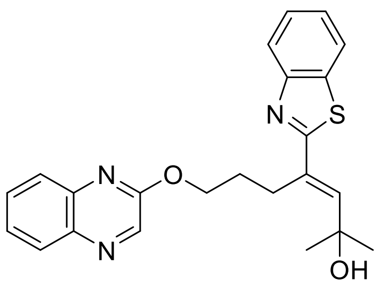

(*E*)-4-(benzo[*d*]thiazol-2-yl)-2-methyl-7-(quinoxalin-2-yloxy)hept-3-en-2-ol (3ag) was prepared by following **General Procedure A** as a colorless oil (47 mg, 58%, 55 h). ^{1}H NMR (400 MHz, $CDCl_3$) δ 8.46 (s, 1H), 8.00 (dd, *J* = 8.4, 1.6 Hz, 1H), 7.94 (d, *J* = 8.4 Hz, 1H), 7.82-7.76 (m, 2H), 7.66-7.62 (m, 1H), 7.57-7.52 (m, 1H), 7.44-7.39 (m, 1H), 7.35-7.30 (m, 1H), 6.57 (s, 1H), 4.60 (t, *J* = 6.4 Hz, 2H), 3.25 (t, *J* = 7.6 Hz, 2H), 2.26-2.18 (m, 2H), 1.51 (s, 6H); ^{13}C NMR (100 MHz, $CDCl_3$) δ 171.1, 157.5, 153.9, 142.9, 140.4, 139.9, 138.9, 135.8, 134.6, 130.2, 129.1, 127.3, 126.6, 126.2, 125.3, 123.2, 121.4, 72.0, 66.5, 31.4, 28.7, 26.0. FT-IR: ν (cm^{-1}) 3411, 2969, 2341, 1571, 1414, 1305, 1220, 1003, 910, 757, 728. HRMS [ESI] calcd for $C_{24}H_{25}N_3O_2SNa$ [M+Na]$^+$ 428.1403, found 428.1412.

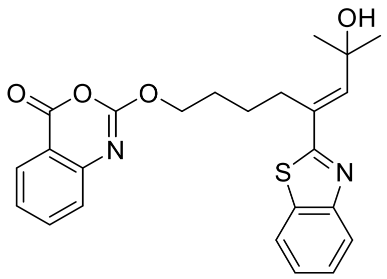

(*E*)-2-((5-(benzo[*d*]thiazol-2-yl)-7-hydroxy-7-methyloct-5-en-1-yl)oxy)-4H-benzo[d][1,3]oxazin-4-one (3ah) was prepared by following **General Procedure A** as a colorless oil (53 mg, 61%, 72 h). ^{1}H NMR (400 MHz, $CDCl_3$) δ 8.09 (d, *J* = 8.0 Hz, 1H), 7.96 (d, *J* = 8.0 Hz, 1H), 7.80 (d, *J* = 7.6 Hz, 1H), 7.72-7.67 (m, 1H), 7.44-7.37 (m, 2H), 7.36-7.29 (m, 2H), 6.56 (s, 1H), 4.49 (t, *J* = 6.4 Hz, 2H), 3.08 (t, *J* = 7.6 Hz, 2H), 1.98-1.90 (m, 2H), 1.82-1.75 (m, 2H), 1.52 (s, 6H); ^{13}C NMR (100 MHz, $CDCl_3$) δ 171.2, 159.7, 154.9, 153.8, 148.5, 142.3, 136.9, 136.4, 134.6, 129.1, 126.1, 125.9, 125.4, 125.2, 123.1, 121.4, 114.6, 71.9, 69.9, 31.5, 28.9, 28.6, 26.0. FT-IR: ν (cm^{-1}) 3387, 2925, 2341, 1769, 1633, 1476, 1247, 1004, 907, 727. HRMS [ESI] calcd for $C_{24}H_{24}N_2O_4SNa$ [M+Na]$^+$ 459.1349, found 459.1357.

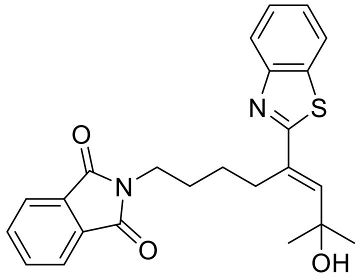

(*E*)-2-(5-(benzo[*d*]thiazol-2-yl)-7-hydroxy-7-methyloct-5-en-1-yl)isoindoline-1,3-dione (3ai) was prepared by following **General Procedure A** as a white solid (58 mg, 70%, 56 h). m.p. 105-106 °C. ^{1}H NMR (400 MHz, $CDCl_3$) δ 7.93 (d, *J* = 8.4 Hz, 1H), 7.82-7.77 (m, 3H), 7.69-7.66 (m, 2H), 7.43-7.39 (m, 1H), 7.34-7.29 (m, 1H), 6.55 (s, 1H), 3.72 (t, *J* = 7.2 Hz, 2H), 3.10 (t, *J* = 7.6 Hz, 2H), 2.43 (br, 1H), 1.82-1.74 (m, 2H), 1.72-1.64 (m, 2H), 1.51 (s, 6H); ^{13}C NMR (100 MHz, $CDCl_3$) δ 171.3, 168.7, 153.8, 142.8, 136.2, 134.6, 134.0, 132.2, 126.1, 125.1, 123.3, 123.1, 121.4, 71.8, 37.9, 31.4, 28.5, 28.4, 26.4. FT-IR: ν (cm^{-1}) 3463, 2865, 2360, 1770, 1703, 1395, 1156, 1036, 758, 718. HRMS [ESI] calcd for $C_{24}H_{24}N_2O_3SK$ [M+K]$^+$ 459.1139, found 459.1145.

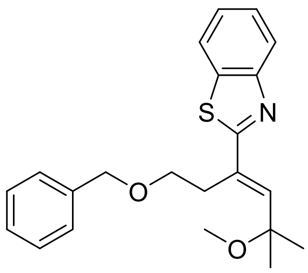

(*E*)-2-(1-(benzyloxy)-5-methoxy-5-methylhex-3-en-3-yl)benzo[*d*]thiazole (3aj) was prepared by following **General Procedure A** as a colorless oil (47 mg, 65%, 50 h). ^{1}H NMR (400 MHz, $CDCl_3$) δ 7.97 (d, *J* = 7.6 Hz, 1H), 7.82 (d, *J* = 8.0 Hz, 1H), 7.48-7.42 (m, 1H), 7.39-7.33 (m, 1H), 7.30-7.19 (m, 5H), 6.46 (s, 1H), 4.51 (s, 2H), 3.74 (t, *J* = 7.2 Hz, 2H), 3.34 (t, *J* = 7.2 Hz, 2H), 3.23 (s, 3H), 1.46 (s, 6H); ^{13}C NMR (100 MHz, $CDCl_3$) δ 170.8, 153.9, 142.1, 138.7, 134.7, 134.6, 128.4, 127.7, 127.5, 126.2, 125.3, 123.3, 121.4, 75.9, 72.9, 69.1, 50.8, 29.6, 27.8. FT-IR: ν (cm^{-1}) 3234, 2935, 1565, 1289, 1210, 1034, 976, 745, 728. HRMS [ESI] calcd for $C_{22}H_{25}NO_2SNa$ [M+Na]$^+$ 390.1498, found 390.1491.

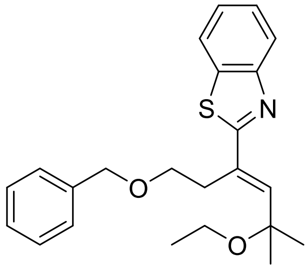

(*E*)-2-(1-(benzyloxy)-5-ethoxy-5-methylhex-3-en-3-yl)benzo[*d*]thiazole (3ak) was prepared by following **General Procedure A** as a colorless oil (45 mg, 60%, 55 h). ^{1}H NMR (400 MHz, $CDCl_3$) δ 7.97 (d, *J* = 8.4 Hz, 1H), 7.82 (d, *J* = 8.0 Hz, 1H), 7.47-7.42 (m, 1H), 7.38-7.33 (m, 1H), 7.30-7.19 (m, 5H), 6.48 (s, 1H), 4.51 (s, 2H), 3.74 (t, *J* = 7.2 Hz, 2H), 3.45 (dd, *J* = 14.0, 7.2 Hz, 2H), 3.36 (t, *J* = 7.2 Hz, 2H), 1.47 (s, 6H), 1.20 (t, *J* = 7.2 Hz, 3H); ^{13}C NMR (100 MHz, $CDCl_3$) δ 170.9, 153.9, 142.9, 138.7, 134.7, 134.4, 128.3, 127.7, 127.5, 126.1, 125.3, 123.2, 121.4, 75.4, 73.0, 69.3, 58.3, 29.7, 28.2, 16.1. FT-IR: ν (cm^{-1}) 3062, 2973, 1588, 1359, 1233, 1067, 964, 758, 728. HRMS [ESI] calcd for $C_{23}H_{28}NO_2S$ [M+H]$^+$ 382.1835, found 382.1844.

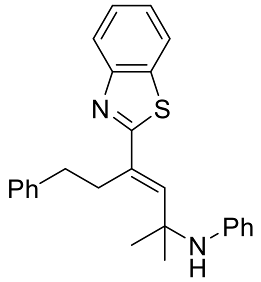

(*E*)-*N*-(4-(benzo[*d*]thiazol-2-yl)-2-methyl-6-phenylhex-3-en-2-yl)aniline (3al) was prepared by following **General Procedure A** as a colorless oil (35 mg, 45%, 45 h). ^{1}H NMR (500 MHz, $CDCl_3$) δ 8.03 (d, *J* = 8.0 Hz, 1H), 7.86 (d, *J* = 8.0 Hz, 1H), 7.49-7.45 (m, 1H), 7.39-7.35 (m, 1H), 7.28-7.23 (m, 2H), 7.19-7.11 (m, 5H), 6.75-6.68 (m, 4H), 3.84 (br, s, 1H), 3.28-3.22 (m, 2H), 2.70-2.65 (m, 2H), 1.54 (s, 6H); ^{13}C NMR (125 MHz, $CDCl_3$) δ 171.1, 154.0, 146.0, 142.5, 141.8, 136.4, 134.7, 129.2, 128.7, 128.4, 126.2, 125.9, 125.2, 123.2, 121.5, 117.7, 115.3, 54.0, 34.7, 31.5, 30.1. FT-IR: ν (cm-1) 3058, 2973, 1731, 1599, 1495, 1313, 1240, 908, 747, 727. HRMS [ESI] calcd for $C_{26}H_{26}N_2SNa$ [M+Na]$^+$ 421.1709, found 421.1711.

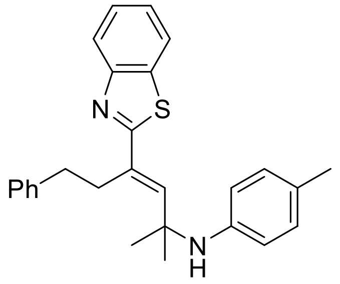

(*E*)-*N*-(4-(benzo[*d*]thiazol-2-yl)-2-methyl-6-phenylhex-3-en-2-yl)-4-methylaniline (3am) was prepared by following **General Procedure A** as a colorless oil (41 mg, 50%, 50 h). ^{1}H NMR (500 MHz, $CDCl_3$) δ 8.03 (d, *J* = 8.0 Hz, 1H), 7.86 (d, *J* = 8.0 Hz, 1H), 7.49-7.44 (m, 1H), 7.39-7.34 (m, 1H), 7.28-7.23 (m, 2H), 7.18-7.12 (m, 3H), 6.95 (d, *J* = 8.0 Hz, 2H), 6.71 (s, 1H), 6.63 (d, *J* = 7.5 Hz, 2H), 3.69 (br, s, 1H), 3.30-3.20 (m, 2H), 2.73-2.67 (m, 2H), 2.21 (s, 3H), 1.52 (s, 6H); ^{13}C NMR (125 MHz, $CDCl_3$) δ 171.3, 154.1, 143.7, 142.6, 142.2, 136.3, 134.7, 129.7, 128.7, 128.4, 127.0, 126.2, 125.9, 125.2, 123.2, 121.5, 115.7, 54.2, 34.8, 31.5, 30.1, 20.5. FT-IR: ν (cm-1) 3398, 3024, 2921, 1733, 1241, 1044, 807, 757, 727. HRMS [ESI] calcd for $C_{27}H_{28}N_2SNa$ [M+Na]$^+$ 435.1865, found 435.1871.

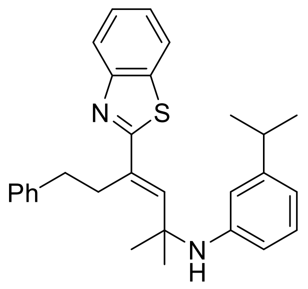

(*E*)-*N*-(4-(benzo[*d*]thiazol-2-yl)-2-methyl-6-phenylhex-3-en-2-yl)-3-isopropylaniline (3an) was prepared by following **General Procedure A** as a colorless oil (48 mg, 55%, 50 h). ^{1}H NMR (500 MHz, $CDCl_3$) δ 8.03 (d, *J* = 8.0 Hz, 1H), 7.87 (d, *J* = 8.0 Hz, 1H), 7.49-7.45 (m, 1H), 7.39-7.35 (m, 1H), 7.26-7.22 (m, 2H), 7.18-7.14 (m, 1H), 7.12-7.05 (m, 3H), 6.71 (s, 1H), 6.60-6.52 (m, 3H), 3.82 (br, s, 1H), 3.27-3.22 (m, 2H), 2.84-2.76 (m, 1H), 2.65-2.60 (m, 2H), 1.55 (s, 6H), 1.21 (dd, *J* = 6.5, 0.5 Hz, 6H); ^{13}C NMR (125 MHz, $CDCl_3$) δ 171.2, 154.1, 149.9, 146.0, 142.6, 142.0, 136.4, 134.7, 129.0, 128.7, 128.4, 126.2, 125.9, 125.2, 123.2, 121.5, 116.1, 113.6, 112.7, 54.0, 34.6, 34.3, 31.6, 30.1, 24.1. FT-IR: ν (cm-1) 3399, 3025, 2966, 1732, 1454, 1166, 777, 757, 727. HRMS [ESI] calcd for $C_{29}H_{32}N_2SNa$ $[M+Na]^+$ 463.2178, found 463.2182.

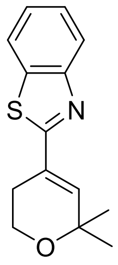

2-(6,6-dimethyl-3,6-dihydro-2*H*-pyran-4-yl)benzo[*d*]thiazole (3ao) was prepared by following **General Procedure A** as a colorless oil (30 mg, 61%, 60 h). ^{1}H NMR (400 MHz, $CDCl_3$) δ 7.99 (d, *J* = 8.4 Hz, 1H), 7.84 (d, *J* = 8.0 Hz, 1H), 7.48-7.43 (m, 1H), 7.38-7.33 (m, 1H), 6.63 (dd, *J* = 1.6, 1.6 Hz, 1H), 3.96 (dd, *J* = 5.2, 5.2 Hz, 2H), 2.75-2.70 (m, 2H), 1.39 (s, 6H); ^{13}C NMR (100 MHz, $CDCl_3$) δ 168.8, 153.8, 138.9, 134.3, 129.7, 126.3, 125.4, 123.2, 121.6, 72.3, 59.6, 27.3, 26.6. FT-IR: ν (cm^{-1}) 3305, 2973, 2244, 1731, 1483, 1433, 1308, 1070, 964, 754, 725. HRMS [ESI] calcd for $C_{14}H_{16}NOS$ $[M+H]^+$ 246.0947, found 246.0939.

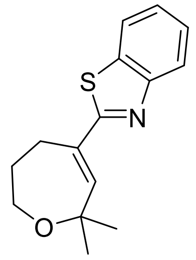

2-(2,2-dimethyl-2,5,6,7-tetrahydrooxepin-4-yl)benzo[*d*]thiazole (3ap) was prepared by following **General Procedure A** as a colorless oil (33 mg, 64%, 60 h). ^{1}H NMR (400 MHz, $CDCl_3$) δ 8.01-7.96 (m, 1H), 7.88-7.84 (m, 1H), 7.47-7.41 (m, 1H), 7.37-7.32 (m, 1H), 5.71-5.68 (m, 1H), 4.11-4.05 (m, 2H), 2.82-2.74 (m, 1H), 2.33-2.24 (m, 1H), 2.08-1.92 (m, 2H), 1.74 (d, *J* = 1.6 Hz, 3H), 1.72 (d, *J* = 1.2 Hz, 3H); ^{13}C NMR (100 MHz, $CDCl_3$) δ 180.0, 154.0, 138.1, 135.5, 127.6, 125.8, 124.8, 123.1, 121.8, 86.4, 68.7, 40.3, 27.0, 25.8, 19.4. FT-IR: ν (cm^{-1}) 3464, 2927, 1711, 1455, 1277, 1051, 907, 758, 727. HRMS [ESI] calcd for $C_{15}H_{18}NOS$ $[M+H]^+$ 260.1104, found 260.1101.

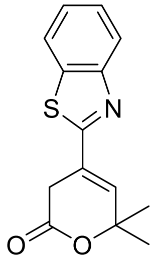

4-(benzo[*d*]thiazol-2-yl)-6,6-dimethyl-3,6-dihydro-2*H*-pyran-2-one (3aq) was prepared by following **General Procedure A** as a colorless oil (51 mg, 73%, 66 h). ^{1}H NMR (400 MHz, $CDCl_3$) δ 8.02 (d, *J* = 8.0 Hz, 1H), 7.88 (d, *J* = 8.0 Hz, 1H), 7.53-7.47 (m, 1H), 7.45-7.39 (m, 1H), 6.72 (s, 1H), 3.73 (s, 2H), 1.62 (s, 6H); ^{13}C NMR (100 MHz, $CDCl_3$) δ 168.3, 165.0, 153.6, 134.4, 133.7, 127.1, 126.7, 126.1, 123.7, 121.7, 82.4, 30.2, 28.9. FT-IR: ν (cm^{-1}) 3295, 2981, 1968, 1718, 1460, 1297, 1218, 1131, 1001, 845, 772, 728. HRMS [ESI] calcd for $C_{14}H_{13}NO_2SNa$ $[M+Na]^+$ 282.0559, found 282.0566.

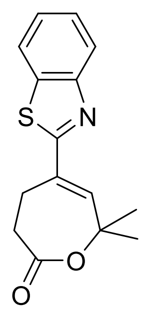

5-(benzo[*d*]thiazol-2-yl)-7,7-dimethyl-4,7-dihydrooxepin-2(3*H*)-one (3ar) was prepared by following **General Procedure A** as a colorless oil (32 mg, 60%, 66 h). ^{1}H NMR (400 MHz, $CDCl_3$) δ 8.02 (d, *J* = 8.0 Hz, 1H), 7.89 (d, *J* = 7.2 Hz, 1H), 7.52-7.47 (m, 1H), 7.43-7.38 (m, 1H), 5.79-5.76 (m, 1H), 3.18-3.10 (m, 1H), 2.83-2.73 (m, 1H), 2.72-2.65 (m, 1H), 2.64-2.55 (m, 1H), 1.81 (d, *J* = 1.6 Hz, 3H), 1.70 (d, *J* = 1.2 Hz, 3H); ^{13}C NMR (100 MHz, $CDCl_3$) δ 176.1, 173.6, 153.2, 141.7, 135.6, 126.4, 125.6, 125.4, 123.6, 121.9, 86.8, 36.1, 28.9, 26.9, 20.0. FT-IR: ν (cm^{-1}) 3392, 2968, 1769, 1498, 1308, 1205, 994, 764. 683. HRMS [ESI] calcd for $C_{15}H_{15}NO_2SNa$ $[M+Na]^+$ 296.0716, found 296.0712.

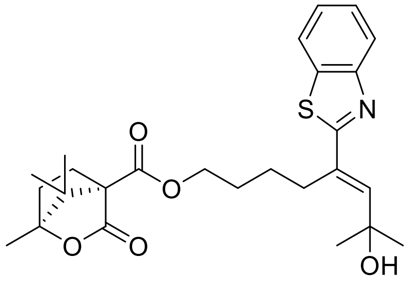

(*E*)-5-(benzo[*d*]thiazol-2-yl)-7-hydroxy-7-methyloct-5-en-1-yl (1*S*,4*R*)-1,7,7-trimethyl-3-oxo-2-oxabicyclo[2.2.1]heptane-4-carboxylate (4a) was prepared by following **General Procedure A** as a colorless oil (66 mg, 71%, 65 h). ^{1}H NMR (400 MHz, $CDCl_3$) δ 7.95 (d, *J* = 8.4 Hz, 1H), 7.81 (d, *J* = 8.0 Hz, 1H), 7.45-7.39 (m, 1H), 7.36-7.30 (m, 1H), 6.52 (s, 1H), 4.32-4.21 (m, 2H), 3.07-3.01 (m, 2H), 2.37-2.27 (m, 1H), 1.98-1.90 (m, 1H), 1.85-1.75 (m, 3H), 1.72-1.56 (m, 3H), 1.50 (s, 6H), 1.05 (s, 3H), 0.94 (s, 3H), 0.86 (s, 3H); ^{13}C NMR (100 MHz, $CDCl_3$) δ 178.3, 171.3, 167.6, 153.8, 142.4, 136.2, 134.6, 126.1, 125.2, 123.1, 121.4, 91.3, 71.2, 65.5, 54.8, 54.1, 31.5, 30.7, 29.0, 28.8, 26.0, 16.8, 16.7, 9.8. FT-IR: ν (cm^{-1}) 3456, 2968, 1786, 1456, 1264, 1105, 1061, 931, 760, 729. HRMS [ESI] calcd for $C_{26}H_{33}NO_5SNa$ $[M+Na]^+$ 494.1977, found 494.1971.

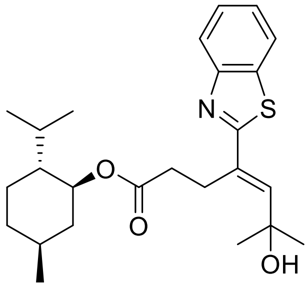

(1*S*,2*R*,5*S*)-2-isopropyl-5-methylcyclohexyl (*E*)-4-(benzo[*d*]thiazol-2-yl)-6-hydroxy-6-methylhept-4-enoate (4b) was prepared by following **General Procedure A** as a colorless oil (58 mg, 68%, 72 h). ^{1}H NMR (400 MHz, $CDCl_3$) δ 7.96 (d, *J* = 8.4 Hz, 1H), 7.81 (d, *J* = 7.6 Hz, 1H), 7.46-7.41 (m, 1H), 7.37-7.32 (m, 1H), 6.51 (s, 1H), 4.72-4.63 (m, 1H), 4.50 (s, 1H), 3.57-3.48 (m, 1H), 3.41-3.33 (m, 1H), 2.85-2.69 (m, 2H), 1.94-1.87 (m, 1H), 1.69-1.57 (m, 3H), 1.47-1.41 (m, 7H), 1.29-1.20 (m, 1H), 1.06-0.95 (m, 1H), 0.95-0.78 (m, 5H), 0.76 (d, *J* = 7.2 Hz, 3H), 0.68 (d, *J* = 7.2 Hz, 3H); ^{13}C NMR (100 MHz, $CDCl_3$) δ 174.8, 170.8, 154.0, 145.0, 134.5, 132.5, 126.1, 125.3, 123.2, 121.4, 74.9, 72.1, 47.0, 41.0, 34.3, 33.1, 31.5, 31.3, 31.0, 26.3, 23.5, 22.7, 22.1, 20.7, 16.5. FT-IR: ν (cm^{-1}) 3445, 2955, 2341, 1704, 1487, 1327, 1204, 959, 758, 728. HRMS [ESI] calcd for $C_{25}H_{35}NO_3SNa$ $[M+Na]^+$ 452.2230, found 452.2219.

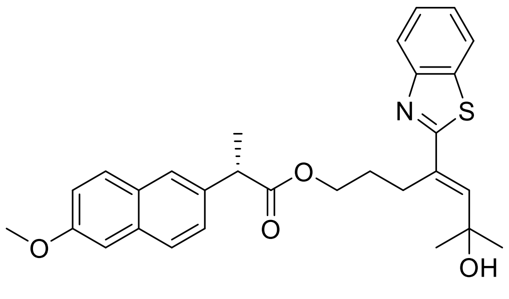

(*E*)-4-(benzo[*d*]thiazol-2-yl)-6-hydroxy-6-methylhept-4-en-1-yl (*S*)-2-(6-methoxynaphthalen-2-yl)propanoate (4c) was prepared by following **General Procedure A** as a colorless oil (66 mg, 68%, 72 h). ^{1}H NMR (400 MHz, $CDCl_3$) δ 7.95 (d, *J* = 8.4 Hz, 1H), 7.78 (d, *J* = 8.0 Hz, 1H), 7.71-7.67 (m, 3H), 7.46-7.40 (m, 2H),

7.36-7.31 (m, 1H), 7.16-7.08 (m, 2H), 6.52 (s, 1H), 4.2-4.13 (m, 2H), 3.90 (s, 3H), 3.89-3.84 (m, 1H), 3.08-2.93 (m, 2H), 1.97-1.88 (m, 2H), 1.59 (d, *J* = 7.2 Hz, 3H), 1.39 (s, 3H), 1.38 (s, 3H); ^{13}C NMR (100 MHz, $CDCl_3$) δ 174.9, 171.0, 157.7, 153.8, 142.7, 135.9, 135.4, 134.6, 133.8, 129.4, 129.0, 127.3, 126.5, 126.2, 126.1, 125.2, 123.1, 121.3, 119.1, 105.7, 71.7, 64.9, 55.4, 45.7, 31.3, 31.2, 28.7, 26.0, 18.7. FT-IR: ν (cm^{-1}) 3254, 2945, 2360, 1713, 1448, 1392, 1177, 1060, 979, 736. HRMS [ESI] calcd for $C_{29}H_{31}NO_4SNa$ $[M+Na]^+$ 512.1866, found 512.1867.

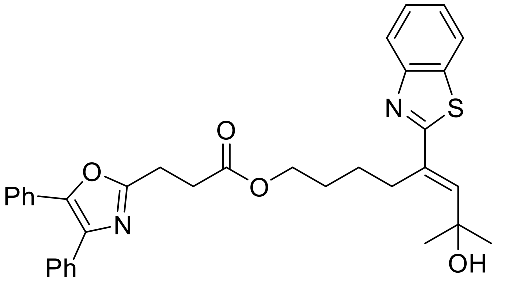

(*E*)-5-(benzo[*d*]thiazol-2-yl)-7-hydroxy-7-methyloct-5-en-1-yl 3-(4,5-diphenyloxazol-2-yl)propanoate (4d) was prepared by following **General Procedure A** as a colorless oil (65 mg, 58%, 72 h). ^{1}H NMR (400 MHz, $CDCl_3$) δ 7.97 (d, *J* = 8.0 Hz, 1H), 7.81 (d, *J* = 8.0 Hz, 1H), 7.63-7.59 (m, 2H), 7.57-7.53 (m, 2H), 7.44-7.39 (m, 1H), 7.37-7.29 (m, 7H), 6.54 (s, 1H), 4.18 (t, *J* = 6.4 Hz, 2H), 3.16 (t, *J* = 7.6 Hz, 2H), 3.02 (t, *J* = 7.6 Hz, 2H), 2.88 (t, *J* = 7.6 Hz, 2H), 2.05 (br, 1 H), 1.82-1.73 (m, 2H), 1.73-1.65 (m, 2H), 1.48 (s, 6H); ^{13}C NMR (100 MHz, $CDCl_3$) δ 172.2, 171.3, 161.9, 153.9, 145.5, 142.4, 136.3, 135.2, 134.7, 132.6, 129.1, 128.7, 128.7, 128.5, 128.2, 128.1, 126.6, 126.1, 125.2, 123.1, 121.4, 71.8, 64.7, 31.4, 31.4, 29.0, 28.8, 26.1, 23.7. FT-IR: ν (cm^{-1}) 3432, 2974, 2360, 1734, 1371, 1238, 1157, 1045, 938, 760, 694. HRMS [ESI] calcd for $C_{34}H_{34}N_2O_4SNa$ $[M+Na]^+$ 589.2131, found 589.2145.

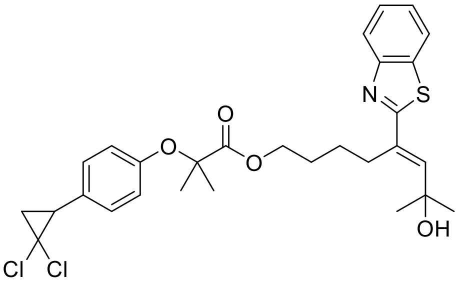

(*E*)-5-(benzo[*d*]thiazol-2-yl)-7-hydroxy-7-methyloct-5-en-1-yl 2-(4-(2,2-dichlorocyclopropyl)phenoxy)-2-methylpropanoate (4e) was prepared by following **General Procedure A** as a colorless oil (68 mg, 61%, 72 h). ^{1}H NMR (500 MHz, $CDCl_3$) δ 7.96 (dd, *J* = 8.0 Hz, 1H), 7.81 (d, *J* = 8.0 Hz, 1H), 7.46-7.41 (m, 1H), 7.37-7.32 (m, 1H), 7.06-7.02 (m, 2H), 6.78-6.74 (m, 2H), 6.53 (s, 1H), 4.20 (t, *J* = 6.4 Hz, 2H), 3.02-2.96 (m, 2H), 2.80-2.74 (m, 1H), 1.92-1.86 (m, 1H), 1.76-1.70 (m, 3H), 1.62-1.56 (m, 2H), 1.55 (s, 6H), 1.49 (s, 6H); ^{13}C NMR (100 MHz, $CDCl_3$) δ 174.4, 171.2, 155.0, 153.8, 142.3, 136.3, 134.6, 129.7, 128.2, 126.2, 125.2, 123.1, 121.4, 118.8, 79.3, 71.8, 65.4, 61.0, 34.9, 31.4, 28.9, 28.7, 26.0 25.9, 25.5. FT-IR: ν (cm^{-1}) 3649, 2972, 2360, 1731, 1509, 1241, 1139, 907, 758, 728. HRMS [ESI] calcd for $C_{29}H_{33}Cl_2NO_4SNa$ $[M+Na]^+$ 562.1580, found 562.1582.

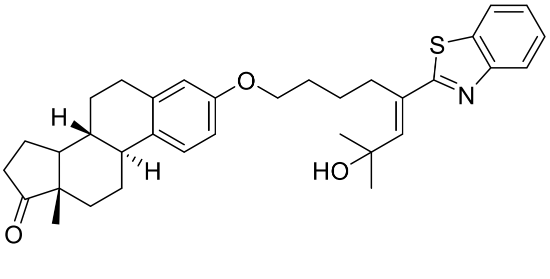

(8*R*,9*S*,13*S*)-3-(((*E*)-5-(benzo[*d*]thiazol-2-yl)-7-hydroxy-7-methyloct-5-en-1-yl)oxy)-13-methyl-6,7,8,9,11,12,13,14,15,16-decahydro-17H-cyclopenta[*a*]phenanthren-17-one (4f) was prepared by following **General Procedure A** as a colorless oil (65 mg, 60%, 72 h). ^{1}H NMR (400 MHz, $CDCl_3$) δ 7.97 (d, *J* = 8.0 Hz, 1H), 7.82 (d, *J* = 8.0 Hz, 1H), 7.47-7.42 (m, 1H), 7.37-7.32 (m, 1H), 7.17 (d, *J* = 8.4 Hz, 1H), 6.72 (dd, *J* = 8.4, 2.4 Hz, 1H), 6.65 (d, *J* = 2.8 Hz, 1H), 6.60 (s, 1H), 4.03 (t, *J* = 6.0 Hz, 2H), 3.26 (t, *J*

= 7.2 Hz, 2H), 2.90-2.83 (m, 2H), 2.70 (br, 1H), 2.54-2.45 (m, 1H), 2.42-2.34 (m, 1H), 2.27-1.92 (m, 8H), 1.68-1.36 (m, 13H), 0.90 (s, 3H); ^{13}C NMR (100 MHz, $CDCl_3$) δ 171.2, 156.6, 153.9, 143.5, 137.9, 134.6, 134.5, 132.4, 126.4, 126.1, 125.2, 123.1, 121.4, 114.8, 112.3, 72.0, 67.3, 50.5, 48.1, 44.1, 38.4, 36.0, 31.7, 31.2, 31.2, 29.7, 28.4, 26.6, 26.0, 25.2, 21.7, 14.0. FT-IR: ν (cm^{-1}) 3452, 2928, 1733, 1497, 1233, 1156, 1055, 907, 759, 727. HRMS [ESI] calcd for $C_{33}H_{42}NO_3S$ [M+H]$^+$ 544.2880, found 544.2886.

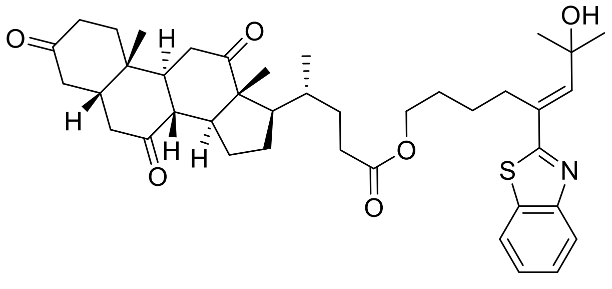

(*E*)-5-(benzo[*d*]thiazol-2-yl)-7-hydroxy-7-methyloct-5-en-1-yl (*R*)-4-((5*S*,8*R*,9*S*,10*S*,13*R*,14*S*,17*R*)-10,13-dimethyl-3,7,12-trioxohexadecahydro-1*H*-cyclopenta[a]phenanthren-17-yl)pentanoate (4g) was prepared by following **General Procedure A** as a colorless oil (51 mg, 38%, 72 h). ^{1}H NMR (400 MHz, $CDCl_3$) δ 7.97 (d, *J* = 8.0 Hz, 1H), 7.82 (d, *J* = 8.0 Hz, 1H), 7.46-7.41 (m, 1H), 7.37-7.31 (m, 1H), 6.55 (s, 1H), 4.10 (t, *J* = 6.4 Hz, 2H), 3.02 (t, *J* = 7.6 Hz, 2H), 2.94-2.77 (m, 3H), 2.39-1.56 (m, 25H), 1.51 (s, 6H), 1.38 (s, 3H), 1.02 (s, 3H), 0.81 (d, *J* = 6.8 Hz, 3H); ^{13}C NMR (100 MHz, $CDCl_3$) δ 212.1, 209.2, 208.8, 174.4, 171.3, 153.9, 142.3, 136.4, 134.7, 126.2, 125.2, 123.2, 121.5, 71.9, 64.3, 57.0, 51.9, 49.1, 47.0, 45.8, 45.7, 45.1, 42.9, 38.8, 36.6, 36.1, 35.6, 35.4, 31.7, 31.5, 30.6, 29.0, 28.9, 27.7, 26.2, 25.3, 22.0, 18.8, 12.0. FT-IR: ν (cm^{-1}) 3456, 2926, 2360, 1704, 1457, 1297, 1156, 908, 758, 728. HRMS [ESI] calcd for $C_{40}H_{53}NO_6SNa$ [M+Na]$^+$ 698.3486, found 698.3493.

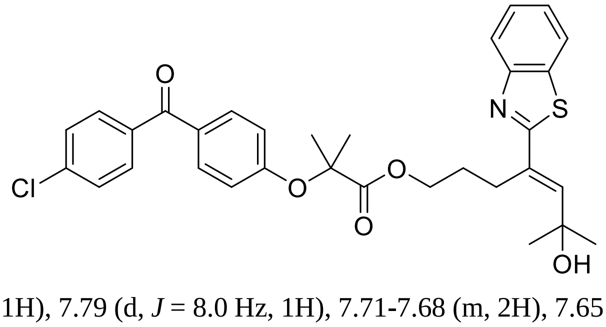

(*E*)-4-(benzo[*d*]thiazol-2-yl)-6-hydroxy-6-methylhept-4-en-1-yl 2-(4-(4-chlorobenzoyl)phenoxy)-2-methylpropanoate (4h) was prepared by following **General Procedure A** as a colorless oil (70 mg, 61%, 72 h). ^{1}H NMR (400 MHz, $CDCl_3$) δ 7.92 (d, *J* = 8.0 Hz, 1H), 7.79 (d, *J* = 8.0 Hz, 1H), 7.71-7.68 (m, 2H), 7.65 (d, *J* = 8.0 Hz, 2H), 7.43-7.37 (m, 3H), 7.35-7.29 (m, 1H), 6.90-6.85 (m, 2H), 6.53 (s, 1H), 4.28 (t, *J* = 6.4 Hz, 2H), 2.99 (t, *J* = 8.0 Hz, 2H), 1.98-1.90 (m, 2H), 1.69 (s, 6H), 1.46 (s, 6H); ^{13}C NMR (100 MHz, $CDCl_3$) δ 194.4, 173.8, 170.8, 159.8, 153.8, 142.9, 138.4, 136.4, 135.4, 134.5, 132.1, 131.3, 130.4, 128.6, 126.1, 125.3, 123.1, 121.4, 117.5, 79.6, 71.8, 65.9, 31.4, 28.6, 25.8, 25.6. FT-IR: ν (cm^{-1}) 3465, 2971, 2360, 1733, 1597, 1249, 1138, 926, 760, 727. HRMS [ESI] calcd for $C_{32}H_{33}ClNO_5S$ [M+H]$^+$ 578.1762, found 578.1766.

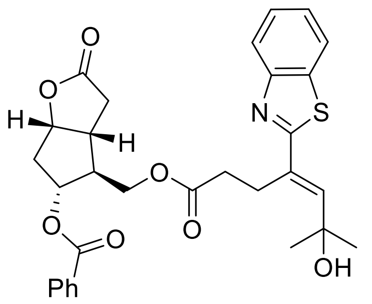

(3a*R*,4*S*,5*R*,6a*S*)-4-((((*E*)-4-(benzo[*d*]thiazol-2-yl)-6-hydroxy-6-methylhept-4-enoyl)oxy)methyl)-2-oxohexahydro-2*H*-cyclopenta[*b*]furan-5-yl benzoate (4i) was prepared by following **General Procedure A** as a colorless oil (55 mg, 50%, 72 h). ^{1}H NMR (400 MHz, $CDCl_3$) δ 7.97-7.93 (m, 3H), 7.81 (d, *J* = 8.0 Hz, 1H), 7.54 (dd, *J* = 7.6, 7.6 Hz, 1H), 7.46-7.39 (m, 3H), 7.37-7.32 (m, 1H), 6.54 (s, 1H), 5.30-5.25 (m, 1H), 5.01-4.95 (m, 1H), 4.10 (d, *J* = 6.0 Hz, 2H), 3.65 (br, 1H), 3.49-3.36 (m, 2H), 2.87-2.78 (m, 3H), 2.73-2.65 (m, 1H), 2.53-2.46 (m, 1H), 2.45-2.37 (m, 2H), 2.32-2.25 (m, 1H), 1.48 (s, 6H); ^{13}C NMR (100 MHz, $CDCl_3$) δ 176.3, 174.4, 170.6, 166.0, 153.9, 144.6, 134.4, 133.5, 133.0, 129.8, 129.5, 128.6, 126.2, 125.5, 123.2, 121.4, 84.3, 72.0, 64.2, 51.7, 40.6, 38.3, 35.9, 33.2, 31.3, 31.2, 23.2. FT-IR: ν (cm^{-1}) 3443, 2973, 2360, 1770, 1716, 1271, 1157, 907, 727. HRMS [ESI] calcd for $C_{30}H_{31}NO_7SNa$ [M+Na]$^+$ 572.1713, found 572.1718.

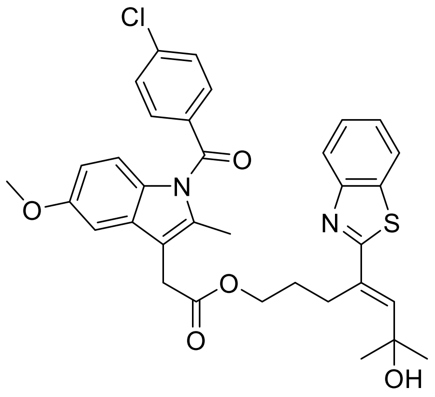

(*E*)-4-(benzo[*d*]thiazol-2-yl)-6-hydroxy-6-methylhept-4-en-1-yl 2-(1-(4-chlorobenzoyl)-5-methoxy-2-methyl-1*H*-indol-3-yl)acetate (4j) was prepared by following **General Procedure A** as a colorless oil (71 mg, 58%, 72 h). ^{1}H NMR (400 MHz, $CDCl_3$) δ 7.94 (d, *J* = 8.0 Hz, 1H), 7.80 (d, *J* = 7.6 Hz, 1H), 7.67-7.62 (m, 2H), 7.45-7.40 (m, 3H), 7.36-7.30 (m, 1H), 7.00 (d, *J* = 2.4 Hz, 1H), 6.88 (d, *J* = 9.2 Hz, 1H), 6.66 (dd, *J* = 8.8, 2.4 Hz, 1H), 6.55 (s, 1H), 4.22 (t, *J* = 6.0 Hz, 2H), 3.79 (s, 3H), 3.68 (s, 2H), 3.09-3.02 (m, 2H), 2.38 (s, 3H), 2.02-1.92 (m, 2H), 1.45 (s, 6H); ^{13}C NMR (100 MHz, $CDCl_3$) δ 171.1, 171.0, 168.5, 156.1, 153.8, 142.7, 139.3, 136.1, 135.4, 134.6, 134.0, 131.3, 131.0, 130.8, 129.2, 126.2, 125.2, 123.1, 121.4, 115.0, 112.8, 111.7, 101.6, 71.7, 65.3, 55.8, 31.4, 30.6, 28.7, 25.9, 13.5. FT-IR: ν (cm^{-1}) 3477, 2967, 2341, 1731, 1682, 1477, 1314, 1221, 1067, 907, 728. HRMS [ESI] calcd for $C_{34}H_{33}ClN_2O_5SNa$ [M+Na]$^+$ 639.1691, found 639.1700.

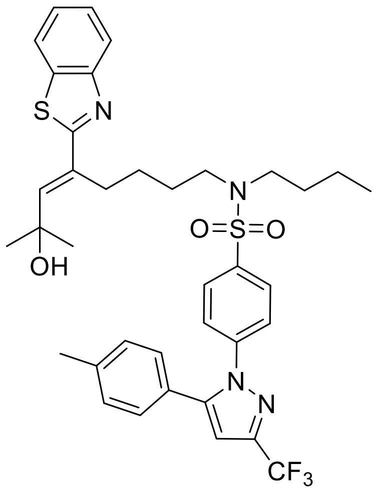

(*E*)-*N*-(5-(benzo[*d*]thiazol-2-yl)-7-hydroxy-7-methyloct-5-en-1-yl)-*N*-butyl-4-(5-(p-tolyl)-3-(trifluoromethyl)-1*H*-pyrazol-1-yl)benzenesulfonamide (4k) was prepared by following **General Procedure A** as a colorless oil (59 mg, 42%, 72 h). ^{1}H NMR (400 MHz, $CDCl_3$) δ 7.94 (d, *J* = 7.6 Hz, 1H), 7.81 (d, *J* = 7.2 Hz, 1H), 7.78-7.74 (m, 2H), 7.45-7.39 (m, 3H), 7.36-7.31 (m, 1H), 7.15 (d, *J* = 8.0 Hz, 2H), 7.08 (d, *J* = 8.0 Hz, 2H), 6.74 (s, 1H), 6.50 (s, 1H), 3.15 (t, *J* = 7.2 Hz, 2H), 3.08 (t, *J* = 7.6 Hz, 2H), 2.99 (t, *J* = 7.2 Hz, 2H), 2.36 (s, 3H), 1.60-1.53 (m, 3H), 1.49 (s, 6H), 1.48-1.42 (m, 2H), 1.31-1.20 (m, 3H), 0.87 (t, *J* = 7.3 Hz, 4H); ^{13}C NMR (100 MHz, $CDCl_3$) δ 171.4, 153.9, 145.4, 144.1 (q, J_{C-F} = 38.3 Hz), 142.4, 142.2, 139.9, 139.9, 136.2, 134.6, 129.8, 128.8, 128.1, 126.1, 125.8, 125.7, 125.2, 123.1, 122.4 (q, J_{C-F} = 267.4 Hz), 121.4, 106.2 (q, J_{C-F} = 1.3 Hz), 71.9, 47.9, 47.9, 31.5, 30.8, 28.8, 28.5, 26.7, 21.4, 20.0, 13.8;

^{19}F NMR (377 MHz, $CDCl_3$) δ -62.32. FT-IR: ν (cm^{-1}) 3612, 2987, 1819, 1756, 1389, 1212, 1176, 1109, 967, 785, 727. HRMS [ESI] calcd for $C_{37}H_{42}F_3N_4O_3S_2$ $[M+H]^+$ 711.2645, found 711.2655.

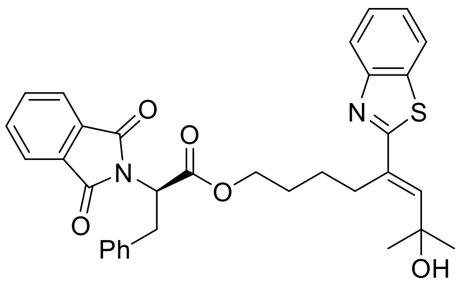

(*E*)-5-(benzo[*d*]thiazol-2-yl)-7-hydroxy-7-methyloct-5-en-1-yl (*R*)-2-(1,3-dioxoisoindolin-2-yl)-3-phenylpropanoate (4l) was prepared by following **General Procedure A** as a colorless oil (70 mg, 62%, 72 h). ^1H NMR (400 MHz, $CDCl_3$) δ 7.95 (d, *J* = 8.0 Hz, 1H), 7.81 (d, *J* = 8.0 Hz, 1H), 7.73-7.69 (m, 2H), 7.63-7.59 (m, 2H), 7.44-7.39 (m, 1H), 7.33 (dd, *J* = 7.6, 7.6 Hz, 1H), 7.19-7.08 (m, 5H), 6.55 (s, 1H), 5.13 (q, *J* = 5.6 Hz, 1H), 4.32-4.18 (m, 2H), 3.61-3.47 (m, 2H), 3.02-2.95 (m, 2H), 2.29-2.22 (m, 1H), 1.79-1.71 (m, 2H), 1.68-1.58 (m, 1H), 1.51 (s, 6H); ^{13}C NMR (100 MHz, $CDCl_3$) δ 171.4, 169.0, 167.7, 153.9, 142.2, 136.9, 136.2, 134.6, 134.2, 131.6, 128.9, 128.6, 126.9, 126.1, 125.1, 123.5, 123.1, 121.4, 71.8, 65.9, 53.6, 34.7, 31.5, 31.4, 28.9, 28.8, 26.1. FT-IR: ν (cm^{-1}) 3514, 2920, 1713, 1386, 1243, 1104, 908, 718. HRMS [ESI] calcd for $C_{33}H_{32}N_2O_5SNa$ $[M+Na]^+$ 591.1924, found 591.1928.

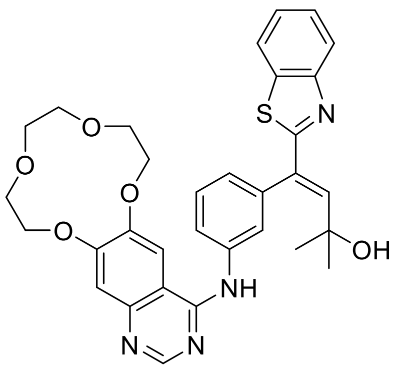

(E)-4-(benzo[d]thiazol-2-yl)-4-(3-((7,8,10,11,13,14-hexahydro-[1,4,7,10]tetraoxacyclododecino[2,3-g]quinazolin-4-yl)amino)phenyl)-2-methylbut-3-en-2-ol (4m) was prepared by following **General Procedure A** as a white solid (30 mg, 25%, 72 h). ^1H NMR (500 MHz, *d*-DMSO) δ 9.53 (s, 1H), 8.48 (s, 1H), 8.17 (s, 1H), 8.02 (d, *J* = 8.8 Hz, 1H), 7.98 (d, *J* = 8.0 Hz, 1H), 7.95 (d, *J* = 8.4 Hz, 1H), 7.85 (s, 1H), 7.50-7.45 (m, 2H), 7.38-7.35 (m, 1H), 7.29 (s, 1H), 7.24 (s, 1H), 7.08 (d, *J* = 7.6 Hz, 1H), 4.92 (s, 1H), 4.30-4.26 (m, 4H), 3.81-3.72 (m, 4H), 3.64-3.60 (m, 4H), 1.21 (s, 6H); ^{13}C NMR (100 MHz, *d*-DMSO) δ 171.3, 156.5, 156.0, 153.3, 153.3, 149.7, 147.7, 144.7, 139.4, 137.6, 134.9, 132.0, 128.2, 126.5, 125.0, 124.8, 123.0, 122.6, 122.1, 121.4, 111.6, 110.5, 109.7, 73.1, 70.5, 70.2, 70.1, 69.9, 68.8, 68.4, 30.3. FT-IR: ν (cm^{-1}) 3369, 2922, 2854, 1622, 1502, 1433, 1242, 1051, 918, 761. HRMS [ESI] calcd for $C_{32}H_{33}N_4O_5S$ $[M+H]^+$ 585.2166, found 585.2174.

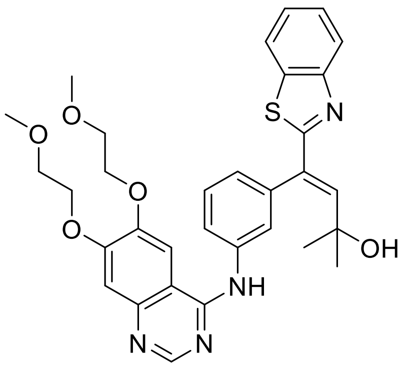

(E)-4-(benzo[d]thiazol-2-yl)-4-(3-((6,7-bis(2-methoxyethoxy)quinazolin-4-yl)amino)phenyl)-2-methylbut-3-en-2-ol (4n) was prepared by following **General Procedure A** as a white solid (27 mg, 23%, 72 h). ^1H NMR (400 MHz, $CDCl_3$) δ 8.55 (s, 1H), 7.94 (d, *J* = 8.0 Hz, 2H), 7.76 (d, *J* = 8.0 Hz, 1H), 7.71 (s, 1H), 7.57 (d, *J* = 8.8 Hz, 1H), 7.43-7.37 (m, 1H), 7.35-7.29 (m, 2H), 7.25 (s, 1H), 7.18 (s, 1H), 7.15-7.13 (m, 2H), 4.22 (t, *J* = 4.8 Hz, 2H), 4.04 (t, *J* = 4.8 Hz, 2H), 3.80 (t, *J* = 4.8 Hz, 2H), 3.71 (t, *J* = 4.8 Hz, 2H), 3.43 (s, 3H), 3.41 (s, 3H), 1.43 (s, 6H); ^{13}C NMR (100 MHz, *d*-DMSO) δ 171.3, 156.3, 153.6, 153.3, 152.8, 148.1, 147.0, 144.7, 139.4, 137.6, 134.9, 132.0, 128.3, 126.5, 125.0, 124.9, 123.2, 122.6, 122.1, 121.6, 109.0, 108.2, 103.3, 70.1, 70.1, 68.4, 68.0, 58.4, 58.4, 30.3. FT-IR: ν

(cm^{-1}) 3278, 2924, 1622, 1531, 1331, 1124, 864, 759, 729. HRMS [ESI] calcd for $C_{32}H_{35}N_4O_5S$ $[M+H]^+$ 587.2323, found 587.2336.

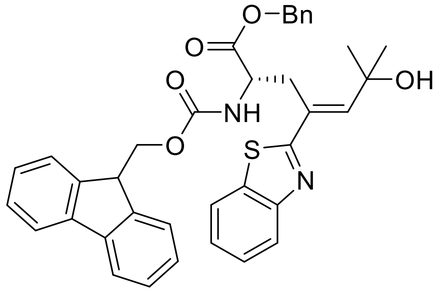

benzyl (S,E)-2-((((9H-fluoren-9-yl)methoxy)carbonyl)amino)-4-(benzo[d]thiazol-2-yl)-6-hydroxy-6-methylhept-4-enoate (4o) was prepared by following **General Procedure A** as a yellow oil (58 mg, 47%, 48 h). ^{1}H NMR (500 MHz, $CDCl_3$) δ 7.98 (d, *J* = 8.0 Hz, 1H), 7.82 (d, *J* = 8.0 Hz, 1H), 7.74-7.70 (m, 2H), 7.51-7.45 (m, 3H), 7.41-7.34 (m, 3H), 7.31-7.27 (m, 4H), 7.20-7.13 (m, 2H), 6.99 (d, *J* = 7.6 Hz, 1H), 6.54 (s, 1H), 5.19 (d, *J* = 12.5 Hz, 1H), 5.16 (d, *J* = 12.0 Hz, 1H), 4.81-4.75 (m, 1H), 4.32-4.24 (m, 2H), 4.17-4.12 (m, 1H), 3.69-3.63 (m, 1H), 3.60-3.55 (m, 1H), 2.81 (s, 1H), 1.50 (s, 3H), 1.48 (s, 3H); ^{13}C NMR (100 MHz, $CDCl_3$) δ 172.8, 171.2, 156.6, 153.6, 145.4, 144.2, 144.0, 141.4, 135.7, 134.5, 130.8, 128.6, 128.3, 128.0, 127.7, 127.7, 127.1, 126.4, 125.7, 125.4, 125.3, 123.4, 121.5, 120.0, 72.5, 67.1, 67.1, 53.9, 47.2, 31.6, 31.4, 30.2. FT-IR: ν (cm^{-1}) 3292, 2970, 1722, 1531, 1209, 1041, 758, 738, 696. HRMS [ESI] calcd for $C_{37}H_{35}N_2O_5S$ $[M+H]^+$ 619.2261, found 619.2267.

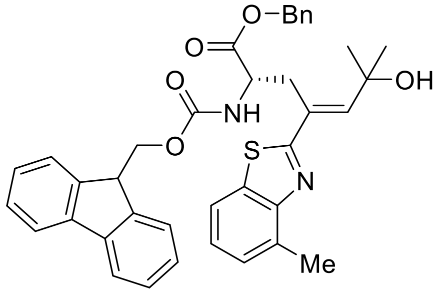

benzyl (S,E)-2-((((9H-fluoren-9-yl)methoxy)carbonyl)amino)-6-hydroxy-6-methyl-4-(4-methylbenzo[d]thiazol-2-yl)hept-4-enoate (4p) was prepared by following **General Procedure A** as a yellow oil (56 mg, 45%, 48 h). ^{1}H NMR (500 MHz, $CDCl_3$) δ 7.72-7.68 (m, 2H), 7.64 (d, *J* = 8.0 Hz, 1H), 7.47 (d, *J* = 7.6 Hz, 1H), 7.44 (d, *J* = 7.6 Hz, 1H), 7.37-7.32 (m, 2H), 7.30-7.22 (m, 7H), 7.18-7.12 (m, 2H), 6.49 (s, 1H), 5.17 (d, *J* = 12.5 Hz, 1H), 5.16 (d, *J* = 12.0 Hz, 1H), 4.82-4.76 (m, 1H), 4.32-4.26 (m, 2H), 4.15 (t, *J* = 7.6 Hz, 1H), 3.69-3.63 (m, 1H), 3.61-3.55 (m, 1H), 2.82 (s, 1H), 2.69 (s, 3H), 1.49 (s, 3H), 1.49 (s, 3H); ^{13}C NMR (100 MHz, $CDCl_3$) δ 173.0, 170.0, 156.6, 152.9, 145.0, 144.2, 144.0, 141.3, 135.7, 134.3, 133.5, 131.1, 128.6, 128.3, 128.0, 127.7, 127.7, 127.1, 126.9, 125.7, 125.4, 125.3, 120.0, 118.8, 72.4, 67.1, 67.0, 54.2, 47.3, 31.8, 31.4, 30.2, 18.5. FT-IR: ν (cm^{-1}) 3292, 2970, 2339, 1760, 1450, 1209, 1043, 738, 696. HRMS [ESI] calcd for $C_{38}H_{37}N_2O_5S$ $[M+H]^+$ 633.2418, found 633.2428.

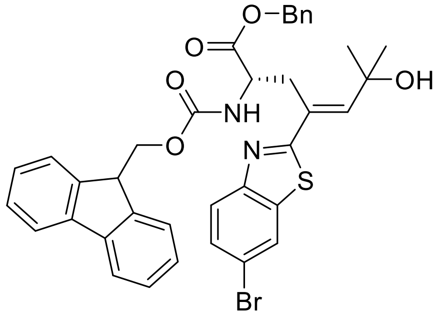

benzyl (S,E)-2-((((9H-fluoren-9-yl)methoxy)carbonyl)amino)-4-(6-bromobenzo[d]thiazol-2-yl)-6-hydroxy-6-methylhept-4-enoate (4q) was prepared by following **General Procedure A** as a yellow oil (55 mg, 40%, 48 h). ^{1}H NMR (500 MHz, $CDCl_3$) δ 7.92 (s, 1H), 7.79 (d, *J* = 8.4 Hz, 1H), 7.74-7.70 (m, 2H), 7.55 (dd, *J* = 8.8, 2.0 Hz, 1H), 7.50 (d, *J* = 8.0 Hz, 1H), 7.46 (d, *J* = 7.6 Hz, 1H), 7.39-7.34 (m, 2H), 7.32-7.28 (m, 4H), 7.21-7.16 (m, 2H), 6.79 (d, *J* = 7.6 Hz, 1H), 6.52 (s, 1H), 5.17 (d, *J* = 12.0 Hz, 1H), 5.15 (d, *J* = 12.0 Hz, 1H), 4.80-4.75 (m, 1H), 4.32-4.26 (m, 2H), 4.14 (t, *J* = 7.6 Hz, 1H),

3.65-3.60 (m, 1H), 3.58-3.52 (m, 1H), 2.82 (s, 1H), 1.49 (s, 3H), 1.48 (s, 3H); ^{13}C NMR (100 MHz, $CDCl_3$) δ 172.7, 171.6, 156.5, 152.5, 145.8, 144.1, 143.9, 141.4, 136.2, 135.7, 130.6, 129.9, 128.6, 128.3, 128.0, 127.7, 127.1, 125.3, 125.2, 124.4, 124.0, 120.0, 119.2, 72.5, 67.2, 67.0, 53.7, 47.2, 31.6, 31.4, 30.3. FT-IR: ν (cm^{-1}) 3304, 2970, 1722, 1531, 1435, 1159, 1043, 813, 738. HRMS [ESI] calcd for $C_{37}H_{34}BrN_2O_5S$ [M+H]$^+$ 697.1366, found 697.1375.

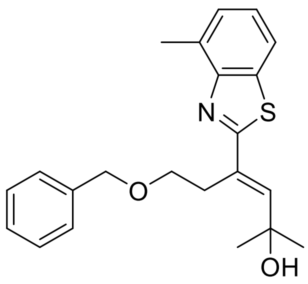

(*E*)-6-(benzyloxy)-2-methyl-4-(4-methylbenzo[*d*]thiazol-2-yl)hex-3-en-2-ol (5a) was prepared by following **General Procedure A** as a colorless oil (47 mg, 64%, 60 h). ^{1}H NMR (400 MHz, $CDCl_3$) δ 7.70-7.65 (m, 1H), 7.36-7.25 (m, 7H), 6.65 (s, 1H), 4.55 (s, 2H), 4.44 (br, 1H), 3.81 (t, *J* = 5.2 Hz, 2H), 3.49 (t, *J* = 5.2 Hz, 2H), 2.72 (s, 3H), 1.40 (s, 6H); ^{13}C NMR (100 MHz, $CDCl_3$) δ 169.8, 153.2, 145.4, 137.6, 134.5, 133.2, 130.9, 128.5, 127.9, 127.9, 126.6, 125.1, 118.7, 73.6, 71.7, 68.8, 31.2, 28.8, 18.4. FT-IR: ν (cm^{-1}) 3444, 2973, 2341, 1771, 1359, 1239, 1154, 1069, 963, 748, 696. HRMS [ESI] calcd for $C_{22}H_{25}NO_2SNa$ [M+Na]$^+$ 390.1504, found 390.1501.

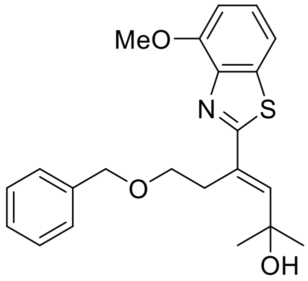

(*E*)-6-(benzyloxy)-4-(4-methoxybenzo[*d*]thiazol-2-yl)-2-methylhex-3-en-2-ol (5b) was prepared by following **General Procedure A** as a colorless oil (46 mg, 60%, 60 h). ^{1}H NMR (400 MHz, $CDCl_3$) δ 7.84 (d, *J* = 8.8 Hz, 1H), 7.35-7.29 (m, 6H), 7.05 (dd, *J* = 8.8, 2.4 Hz, 1H), 6.59 (s, 1H), 4.54 (s, 2H), 4.42 (br, 1H), 3.90 (s, 3H), 3.77 (t, *J* = 5.2 Hz, 2H), 3.43 (t, *J* = 5.2 Hz, 2H), 1.38 (s, 6H); ^{13}C NMR (100 MHz, $CDCl_3$) δ 168.9, 157.9, 145.2, 137.5, 135.9, 130.4, 128.5, 128.0, 128.0, 123.6, 115.2, 104.2, 73.6, 71.8, 68.7, 55.9, 31.3, 28.7. FT-IR: ν (cm^{-1}) 3430, 2970, 2341, 1750, 1600, 1462, 1261, 1224, 1054, 873, 736. HRMS [ESI] calcd for $C_{22}H_{25}NO_3SNa$ [M+Na]$^+$ 406.1453, found 406.1464.

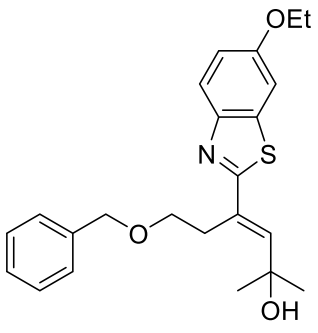

(*E*)-6-(benzyloxy)-4-(6-ethoxybenzo[*d*]thiazol-2-yl)-2-methylhex-3-en-2-ol (5c) was prepared by following **General Procedure A** as a colorless oil (47 mg, 60%, 72 h). ^{1}H NMR (400 MHz, $CDCl_3$) δ 7.80 (d, *J* = 8.8 Hz, 1H), 7.31-7.25 (m, 6H), 7.01 (dd, *J* = 8.8, 2.4 Hz, 1H), 6.56 (s, 1H), 4.51 (s, 2H), 4.38 (br, 1H), 4.08 (q, *J* = 6.8 Hz, 2H), 3.75 (t, *J* = 5.6 Hz, 2H), 3.40 (t, *J* = 5.6 Hz, 2H), 1.44 (t, *J* = 6.8 Hz, 3H), 1.35 (s, 6H). ^{13}C NMR (100 MHz, $CDCl_3$) δ 168.8, 157.2, 148.4, 145.0, 137.5, 136.0, 130.5, 128.6, 128.0, 128.0, 123.6, 115.6, 104.9, 73.6, 71.8, 68.7, 64.2, 31.3, 28.7, 15.0. FT-IR: ν (cm^{-1}) 3431, 2974, 2341, 1735, 1599, 1454, 1258, 1054, 963, 821. HRMS [ESI] calcd for $C_{23}H_{27}NO_3SNa$ [M+Na]$^+$ 420.1609, found 420.1614.

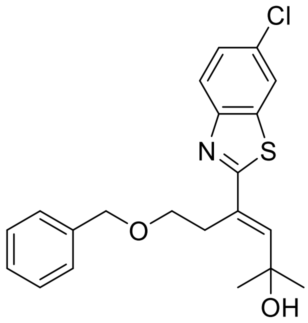

(*E*)-6-(benzyloxy)-4-(6-chlorobenzo[*d*]thiazol-2-yl)-2-methylhex-3-en-2-ol (5d) was prepared by following **General Procedure A** as a colorless oil (46 mg, 60%, 72 h). ^{1}H NMR (400 MHz, $CDCl_3$) δ 7.85 (d, *J* = 8.8 Hz, 1H), 7.81 (d, *J* = 2.0 Hz, 1H), 7.41 (dd, *J* = 8.4, 2.0 Hz, 1H), 7.34-7.28 (m, 5H), 6.66 (s, 1H), 4.54 (s, 2H), 4.42 (br, 1H), 3.77 (t, *J* = 5.2 Hz, 2H), 3.44 (t, *J* = 5.2 Hz, 2H), 1.38 (s, 6H); ^{13}C NMR (100 MHz, $CDCl_3$) δ 171.9, 152.5, 146.5, 137.4, 135.9, 131.1, 130.2, 128.6, 128.0, 128.0, 126.9, 123.8, 121.0, 73.6, 71.9, 68.6, 31.1, 28.6. FT-IR: ν (cm^{-1}) 3431, 2973, 2341, 1736, 1486, 1238, 1093, 964, 815, 697. HRMS [ESI] calcd for $C_{21}H_{22}ClNO_2SNa$ [M+Na]$^+$ 410.0957, found 410.0951.

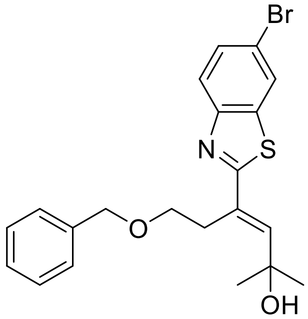

(*E*)-6-(benzyloxy)-4-(6-bromobenzo[*d*]thiazol-2-yl)-2-methylhex-3-en-2-ol (5e) was prepared by following **General Procedure A** as a colorless oil (55 mg, 64%, 72 h). ^{1}H NMR (400 MHz, $CDCl_3$) δ 7.94 (d, *J* = 1.6 Hz, 1H), 7.76 (d, *J* = 8.8 Hz, 1H), 7.52 (dd, *J* = 8.4, 2.0 Hz, 1H), 7.32-7.25 (m, 5H), 6.63 (s, 1H), 4.51 (s, 2H), 4.38 (br, 1H), 3.76-3.72 (m, 2H), 3.41 (t, *J* = 5.2 Hz, 2H), 1.35 (s, 6H); ^{13}C NMR (100 MHz, $CDCl_3$) δ 171.9, 152.8, 146.6, 137.4, 136.4, 130.3, 129.6, 128.6, 128.0, 124.2, 123.9, 118.8, 73.6, 71.9, 68.6, 31.1, 28.6. FT-IR: ν (cm^{-1}) 3421, 2876, 1876, 1732, 1398, 1208, 1056, 987, 856, 704. HRMS [ESI] calcd for $C_{21}H_{22}BrNO_2SNa$ [M+Na]$^+$ 454.0447, found 454.0445.

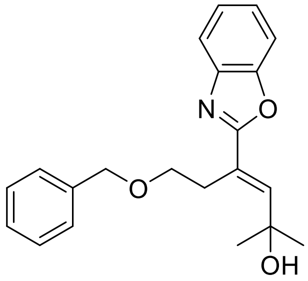

(*E*)-4-(benzo[*d*]oxazol-2-yl)-6-(benzyloxy)-2-methylhex-3-en-2-ol (5f) was prepared by following **General Procedure A** as a colorless oil (47 mg, 70%, 60 h). ^{1}H NMR (400 MHz, $CDCl_3$) δ 7.70-7.66 (m, 1H), 7.50-7.47 (m, 1H), 7.33-7.25 (m, 7H), 7.04 (s, 1H), 4.51 (s, 2H), 4.43 (br, 1H), 3.78 (t, *J* = 5.6 Hz, 2H), 3.37 (t, *J* = 5.6 Hz, 2H), 1.36 (s, 6H); ^{13}C NMR (100 MHz, $CDCl_3$) δ 164.7, 150.6, 147.0, 142.1, 137.3, 128.6, 128.2, 128.0, 125.1, 124.4, 122.7, 120.0, 110.3, 73.7, 72.0, 68.7, 31.0, 27.2. FT-IR: ν (cm^{-1}) 3436, 3031, 1534, 1454, 1358, 1164, 1077, 743, 696. HRMS [ESI] calcd for $C_{21}H_{24}NO_3$ [M+H]$^+$ 338.1751, found 338.1760.

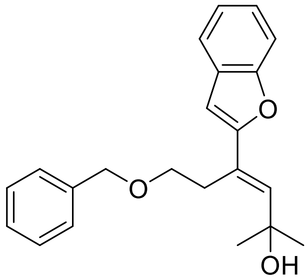

(*E*)-4-(benzofuran-2-yl)-6-(benzyloxy)-2-methylhex-3-en-2-ol (5g) was prepared by following **General Procedure A** as a colorless oil (30 mg, 44%, 50 h). ^{1}H NMR (400 MHz, $CDCl_3$) δ 7.49 (d, *J* = 7.6 Hz, 1H), 7.43 (d, *J* = 8.0 Hz, 1H), 7.34-7.22 (m, 6H), 7.18 (t, *J* = 7.6 Hz, 1H), 6.59 (s, 1H), 6.54 (s, 1H), 4.52 (s, 2H), 4.17 (s, 1H), 3.70 (t, *J* = 5.6 Hz, 2H), 3.15 (t, *J* = 5.6 Hz, 2H), 1.38 (s, 6H); ^{13}C NMR (100 MHz, $CDCl_3$) δ 147.2, 140.5, 140.0, 138.8, 137.6, 129.4, 128.6, 127.9, 127.9, 124.5, 124.5, 123.4, 122.1, 119.5, 73.6, 71.6, 68.3, 31.7, 29.8. FT-IR: ν (cm^{-1}) 3582, 3434, 2924, 1452, 1361, 1254, 1189, 1102, 740, 697. HRMS [ESI] calcd for $C_{22}H_{24}O_3Na$ [M+Na]$^+$ 359.1618, found 359.1617.

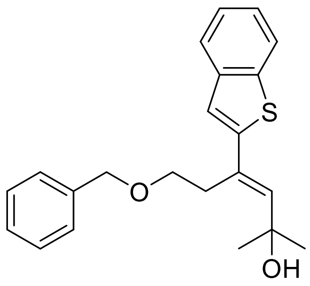

(*E*)-4-(benzo[*b*]thiophen-2-yl)-6-(benzyloxy)-2-methylhex-3-en-2-ol (5h) was prepared by following **General Procedure A** as a colorless oil (28 mg, 40%, 50 h). ^{1}H NMR (400 MHz, $CDCl_3$) δ 7.76-7.72 (m, 1H), 7.67-7.64 (m, 1H), 7.34-7.24 (m, 7H), 7.13 (s, 1H), 6.22 (s, 1H), 4.51 (s, 2H), 4.07 (s, 1H), 3.68 (t, *J* = 5.6 Hz, 2H), 3.23 (t, *J* = 5.6 Hz, 2H), 1.36 (s, 6H); ^{13}C NMR (100 MHz, $CDCl_3$) δ 157.4, 154.7, 138.7, 137.5, 129.2, 128.6, 128.0, 124.3, 124.3, 122.8, 120.7, 110.9, 101.6, 73.6, 71.8, 68.6, 31.6, 27.9. FT-IR: ν (cm^{-1}) 3442, 2925, 2856, 1494, 1361, 1088, 963, 907, 727, 697. HRMS [ESI] calcd for $C_{22}H_{24}O_2SNa$ $[M+Na]^+$ 375.1389, found 375.1388.

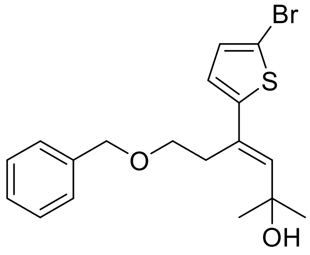

(*E*)-6-(benzyloxy)-4-(5-bromothiophen-2-yl)-2-methylhex-3-en-2-ol (5i) was prepared by following **General Procedure A** as a colorless oil (38 mg, 51%, 65 h). ^{1}H NMR (400 MHz, $CDCl_3$) δ 7.37-7.28 (m, 5H), 6.91 (d, *J* = 3.6 Hz, 1H), 6.60 (d, *J* = 3.6 Hz, 1H), 5.81 (s, 1H), 4.47 (s, 2H), 3.47 (t, *J* = 6.6 Hz, 2H), 2.56-2.50 (m, 2H), 1.30 (s, 6H). ^{13}C NMR (100 MHz, $CDCl_3$) δ 142.8, 141.0, 138.4, 129.7, 128.5, 128.5, 127.8, 127.8, 127.1, 112.1, 73.0, 71.5, 68.2, 41.9, 31.3. FT-IR: ν (cm^{-1}) 3341, 2867, 2245, 1534, 1298, 994, 965, 775, 643. HRMS [ESI] calcd for $C_{18}H_{21}BrO_2SNa$ $[M+Na]^+$ 403.0338, found 403.0332.

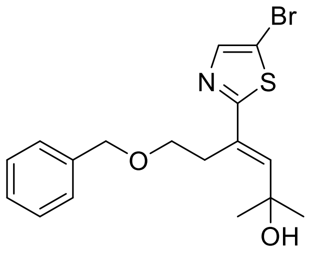

(*E*)-6-(benzyloxy)-4-(5-bromothiazol-2-yl)-2-methylhex-3-en-2-ol (5j) was prepared by following **General Procedure A** as a colorless oil (30 mg, 40%, 72 h). ^{1}H NMR (400 MHz, $CDCl_3$) δ 7.57 (s, 1H), 7.34-7.26 (m, 5H), 6.41 (s, 1H), 4.51 (s, 2H), 4.28 (s, 1H), 3.66 (t, *J* = 5.6 Hz, 2H), 3.27 (t, *J* = 5.6 Hz, 2H), 1.31 (s, 6H); ^{13}C NMR (100 MHz, $CDCl_3$) δ 172.9, 144.1, 143.4, 137.4, 129.5, 128.6, 128.0, 107.5, 100.1, 73.6, 71.7, 68.3, 31.2, 28.5. FT-IR: ν (cm^{-1}) 3431, 2971, 2341, 1485, 1359, 1070, 998, 963, 734, 696. HRMS [ESI] calcd for $C_{17}H_{20}BrNO_2SNa$ $[M+Na]^+$ 404.0296, found 404.0311.

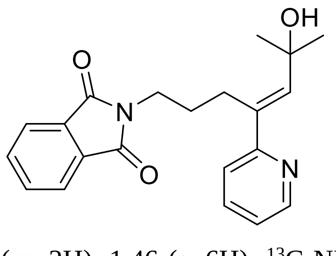

(*E*)-2-(6-hydroxy-6-methyl-4-(pyridin-2-yl)hept-4-en-1-yl)isoindoline-1,3-dione (5k) was prepared by following **General Procedure A** as a colorless oil (39 mg, 56%, 65 h). ^{1}H NMR (400 MHz, $CDCl_3$) δ 8.45-8.40 (m, 1H), 7.81-7.76 (m, 2H), 7.72-7.67 (m, 2H), 7.56-7.49 (m, 1H), 7.21 (d, *J* = 8.0 Hz, 1H), 7.10-7.05 (m, 1H), 6.14 (s, 1H), 3.71 (t, *J* = 7.0 Hz, 2H), 3.13-3.05 (m, 2H), 1.85-1.76 (m, 2H), 1.46 (s, 6H); ^{13}C NMR (100 MHz, $CDCl_3$) δ 168.7, 159.9, 149.0, 140.6, 139.0, 136.4, 134.0, 132.3, 123.2, 121.9, 120.9, 71.5, 38.3, 31.6, 27.5, 25.8. FT-IR: ν (cm^{-1}) 3463, 2968, 1770, 1706, 1434, 1361, 1170, 960, 911, 719. HRMS [ESI] calcd for $C_{19}H_{23}NO_2Na$ $[M+Na]^+$ 373.1523, found 373.1522.

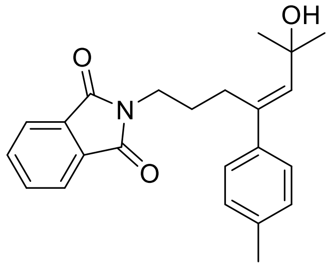

(*E*)-2-(6-hydroxy-6-methyl-4-(p-tolyl)hept-4-en-1-yl)isoindoline-1,3-dione (5l) was prepared by following **General Procedure B** as a colorless oil (48 mg, 67%, 45 h). ^{1}H NMR (400 MHz, $CDCl_3$) δ 7.82-7.76 (m, 2H), 7.72-7.66 (m, 2H), 7.06 (d, *J* = 8.0 Hz, 2H), 7.00 (d, *J* = 8.0 Hz, 2H), 5.63 (s, 1H), 3.67 (t, *J* = 6.8 Hz, 2H), 2.98-2.92 (m, 2H), 2.29 (s, 3H), 1.79-1.70 (m, 2H), 1.41 (s, 6H); ^{13}C NMR (100 MHz, $CDCl_3$) δ 168.6, 141.2, 140.1, 136.7, 136.1, 134.0, 132.3, 129.0, 126.5, 123.2, 71.4, 38.3, 31.8, 27.4, 27.1, 21.2. FT-IR: ν (cm^{-1}) 3465, 2969, 1770, 1704, 1395, 1159, 817, 717. HRMS [ESI] calcd for $C_{23}H_{25}NO_3Na$ [M+Na]$^+$ 386.1727, found 386.1720.

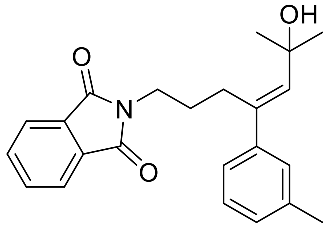

(*E*)-2-(6-hydroxy-6-methyl-4-(m-tolyl)hept-4-en-1-yl)isoindoline-1,3-dione (5m) was prepared by following **General Procedure B** as a colorless oil (44 mg, 61%, 45 h). ^{1}H NMR (400 MHz, $CDCl_3$) δ 7.82-7.77 (m, 2H), 7.72-7.67 (m, 2H), 7.09 (dd, *J* = 7.6, 7.6 Hz, 1H), 7.01-6.94 (m, 2H), 6.91 (s, 1H), 5.63 (s, 1H), 3.68 (t, *J* = 6.8 Hz, 2H), 2.98 (t, *J* = 7.2 Hz, 2H), 2.21 (s, 3H), 1.81-1.72 (m, 2H), 1.42 (s, 6H); ^{13}C NMR (100 MHz, $CDCl_3$) δ 168.7, 143.0, 141.5, 137.8, 136.6, 134.0, 132.3, 128.2, 127.8, 127.4, 123.7, 123.2, 71.4, 38.3, 31.8, 27.2, 27.1, 21.6. FT-IR: ν (cm^{-1}) 3711, 2971, 2362, 1636, 1363, 1066, 959, 781, 718. HRMS [ESI] calcd for $C_{23}H_{25}NO_3Na$ [M+Na]$^+$ 386.1727, found 386.1729.

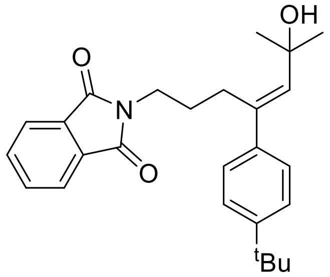

(*E*)-2-(4-(4-(tert-butyl)phenyl)-6-hydroxy-6-methylhept-4-en-1-yl)isoindoline-1,3-dione (5n) was prepared by following **General Procedure B** as a colorless oil (56 mg, 70%, 55 h). ^{1}H NMR (400 MHz, $CDCl_3$) δ 7.80-7.76 (m, 2H), 7.72-7.68 (m, 2H), 7.22-7.17 (m, 2H), 7.11-7.07 (m, 2H), 5.66 (s, 1H), 3.68 (t, *J* = 6.8 Hz, 2H), 2.99-2.93 (m, 2H), 1.82-1.72 (m, 2H), 1.41 (s, 6H), 1.28 (s, 9H); ^{13}C NMR (100 MHz, $CDCl_3$) δ 168.6, 150.0, 141.0, 139.9, 136.2, 134.0, 132.3, 126.2, 125.2, 123.2, 71.4, 38.3, 34.5, 31.8, 31.5, 27.4, 27.1. FT-IR: ν (cm^{-1}) 3466, 2963, 2360, 1704, 1508, 1395, 1362, 1168, 719. HRMS [ESI] calcd for $C_{26}H_{31}NO_3Na$ [M+Na]$^+$ 428.2196, found 428. 2192.

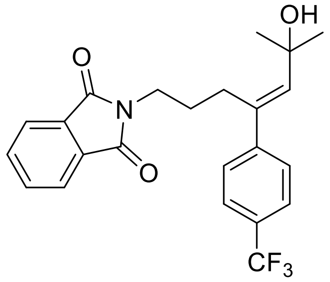

(*E*)-2-(6-hydroxy-6-methyl-4-(4-(trifluoromethyl)phenyl)hept-4-en-1-yl)isoindoline-1,3-dione (5o) was prepared by following **General Procedure B** as a colorless oil (55 mg, 66%, 60 h). ^{1}H NMR (400 MHz, $CDCl_3$) δ 7.81-7.77 (m, 2H), 7.72-7.69 (m, 2H), 7.44 (d, *J* = 8.0 Hz, 2H), 7.27 (d, *J* = 8.4 Hz, 2H), 5.69 (s, 1H), 3.67 (t, *J* = 6.8 Hz, 2H), 3.02-2.97 (m, 2H), 1.78-1.70 (m, 2H), 1.43 (s, 6H); ^{13}C NMR (100 MHz, $CDCl_3$) δ 168.6, 146.7, 140.2, 138.5, 134.2, 134.1, 133.0 (q, $J_{C\text{-}F}$ = 216.6 Hz), 132.1, 126.9, 125.2 (q, $J_{C\text{-}F}$ = 3.7 Hz), 123.3, 71.5, 38.1, 31.7, 27.2, 27.0; ^{19}F NMR (377 MHz, $CDCl_3$) δ -62.4. FT-IR: ν (cm^{-1}) 3065, 2246, 1700, 1488, 1142, 1042, 906, 759, 727. HRMS [ESI] calcd for $C_{23}H_{22}F_3NO_3Na$ [M+Na]$^+$ 440.1444, found 440.1434.

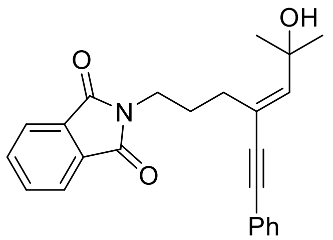

(*E*)-2-(6-hydroxy-6-methyl-4-(phenylethynyl)hept-4-en-1-yl)isoindoline-1,3-dione (*E*-5p) was prepared by following **General Procedure B** as a colorless oil (14 mg, 20%, 50 h). ^{1}H NMR (400 MHz, $CDCl_3$) δ 7.73-7.69 (m, 2H), 7.66-7.62 (m, 2H), 7.34-7.30 (m, 2H), 7.29-7.26 (m, 3H), 5.91 (s, 1H), 3.80 (t, *J* = 6.4 Hz, 2H), 2.75 (t, *J* = 6.8 Hz, 2H), 2.57 (br, 1H), 2.19-2.11 (m, 2H), 1.38 (s, 6H); ^{13}C NMR (100 MHz, $CDCl_3$) δ 169.1, 144.8, 133.9, 132.3, 131.6, 128.3, 128.1, 124.1, 123.4, 123.2, 90.8, 88.3, 71.2, 38.2, 31.3, 28.3, 26.7. FT-IR: ν (cm^{-1}) 3449, 2971, 2362, 1770, 1710, 1507, 1397, 1188, 890, 754. HRMS [ESI] calcd for $C_{24}H_{23}NO_3Na$ $[M+Na]^+$ 396.1570, found 396.1567.

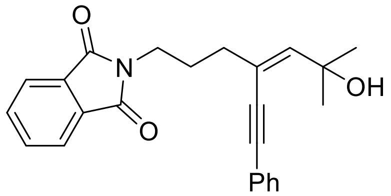

(*Z*)-2-(6-hydroxy-6-methyl-4-(phenylethynyl)hept-4-en-1-yl)isoindoline-1,3-dione (*Z*-5p) was prepared by following **General Procedure B** as a colorless oil (14 mg, 20%, 50 h). ^{1}H NMR (400 MHz, $CDCl_3$) δ 7.85-7.80 (m, 2H), 7.72-7.68 (m, 2H), 7.44-7.40 (m, 2H), 7.34-7.30 (m, 3H), 5.98 (s, 1H), 3.75 (t, *J* = 6.8 Hz, 2H), 3.08 (br, 1H), 2.32-2.26 (m, 2H), 2.06-1.98 (m, 2H), 1.44 (s, 6H); ^{13}C NMR (100 MHz, $CDCl_3$) δ 168.6, 145.8, 134.0, 132.3, 131.5, 128.7, 128.5, 123.3, 122.8, 119.4, 97.9, 86.5, 71.3, 37.5, 35.6, 30.1, 27.2. FT-IR: ν (cm^{-1}) 3232, 2985, 2345, 1870, 1737, 1534, 1409, 1167, 887, 765. HRMS [ESI] calcd for $C_{24}H_{23}NO_3Na$ $[M+Na]^+$ 396.1570, found 396.1575.

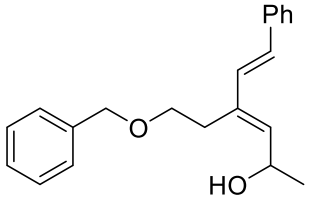

(3*E*,5*E*)-4-(2-(benzyloxy)ethyl)-6-phenylhexa-3,5-dien-2-ol (5q) was prepared by following **General Procedure B** as a colorless oil (9 mg, 15%, 50 h). ^{1}H NMR (400 MHz, $CDCl_3$) δ 7.42-7.38 (m, 2H), 7.36-7.28 (m, 5H), 7.25-7.18 (m, 3H), 6.50 (d, *J* = 12.4 Hz, 1H), 6.03 (d, *J* = 12.4 Hz, 1H), 5.31 (d, *J* = 8.8 Hz, 1H), 4.50 (s, 2H), 4.42-4.34 (m, 1H), 3.66-3.60 (m, 2H), 2.49 (t, *J* = 6.4 Hz, 2H), 1.00 (d, *J* = 6.4 Hz, 3H); ^{13}C NMR (100 MHz, $CDCl_3$) δ 138.5, 137.2, 135.3, 132.8, 131.3, 128.8, 128.6, 128.5, 128.4, 127.8, 127.7, 127.7, 73.0, 68.9, 65.7, 37.3, 22.4. FT-IR: ν (cm^{-1}) 3245, 2934, 1789, 1667, 1433, 1256, 1109, 945, 856, 778. HRMS [ESI] calcd for $C_{21}H_{24}O_2Na$ $[M+Na]^+$ 331.1669, found 331.1675.

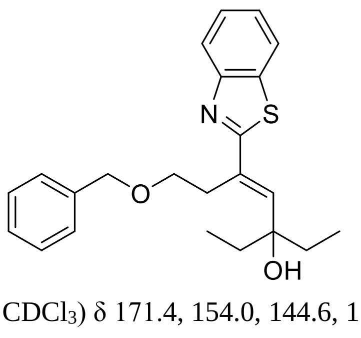

(*E*)-5-(benzo[*d*]thiazol-2-yl)-7-(benzyloxy)-3-ethylhept-4-en-3-ol (5r) was prepared by following **General Procedure A** as a colorless oil (35 mg, 47%, 72 h). ^{1}H NMR (400 MHz, $CDCl_3$) δ 7.93 (d, *J* = 7.6 Hz, 1H), 7.81 (d, *J* = 8.0 Hz, 1H), 7.45-7.41 (m, 1H), 7.36-7.32 (m, 1H), 7.29-7.24 (m, 5H), 6.48 (s, 1H), 4.50 (s, 2H), 4.44 (br, 1H), 3.76-3.72 (m, 2H), 3.42 (t, *J* = 5.2 Hz , 2H), 1.67-1.51 (m, 4H), 0.85 (t, *J* = 7.6 Hz, 6H); ^{13}C NMR (100 MHz, $CDCl_3$) δ 171.4, 154.0, 144.6, 137.4, 134.6, 132.9, 128.6, 128.2, 128.0, 126.1, 125.2, 123.1, 121.4, 77.1, 73.7, 68.6, 34.8, 28.9, 8.3. FT-IR: ν (cm^{-1}) 3439, 2877, 1753, 1480, 1390, 1200, 1140, 932, 744, 696. HRMS [ESI] calcd for $C_{23}H_{27}NO_2SNa$ $[M+Na]^+$ 382.1835, found 382.1830.

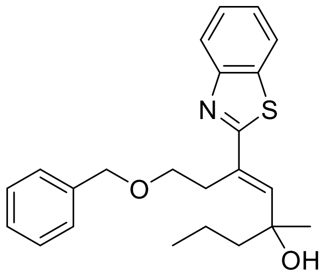

(*E*)-6-(benzo[*d*]thiazol-2-yl)-8-(benzyloxy)-4-methyloct-5-en-4-ol (5s) was prepared by following **General Procedure A** as a colorless oil (51 mg, 67%, 72 h). ^{1}H NMR (400 MHz, $CDCl_3$) δ 7.93 (d, *J* = 8.0 Hz, 1H), 7.81 (d, *J* = 8.0 Hz, 1H), 7.45-7.41 (m, 1H), 7.36-7.32 (m, 1H), 7.30-7.25 (m, 5H), 6.58 (s, 1H), 4.50 (s, 2H), 4.43 (br, 1H), 3.76 (t, *J* = 6.0 Hz, 2H), 3.40-3.45 (m, 2H), 1.58-1.51 (m, 2H), 1.36-1.28 (m, 5H), 0.86 (t, *J* = 7.2 Hz, 3H); ^{13}C NMR (100 MHz, $CDCl_3$) δ 171.4, 154.0, 145.6, 137.4, 134.6, 131.2, 128.5, 128.1, 128.0, 126.1, 125.2, 123.1, 121.4, 74.2, 73.7, 68.7, 46.2, 29.8, 28.7, 17.5, 14.7. FT-IR: ν (cm^{-1}) 3444, 2928, 1738, 1371, 1237, 1045, 937, 758, 697. HRMS [ESI] calcd for $C_{23}H_{27}NO_2SNa$ $[M+Na]^+$ 404.1655, found 404.1650.

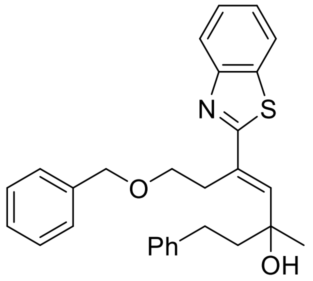

(*E*)-5-(benzo[*d*]thiazol-2-yl)-7-(benzyloxy)-3-methyl-1-phenylhept-4-en-3-ol (5t) was prepared by following **General Procedure A** as a colorless oil (57 mg, 65%, 72 h). ^{1}H NMR (400 MHz, $CDCl_3$) δ 7.98 (d, *J* = 8.0 Hz, 1H), 7.86 (d, *J* = 8.0 Hz, 1H), 7.50-7.45 (m, 1H), 7.41-7.36 (m, 1H), 7.34-7.27 (m, 7H), 7.21-7.13 (m, 3H), 6.66 (s, 1H), 4.67 (s, 1H), 4.55 (s, 2H), 3.87-3.78 (m, 2H), 3.53-3.48 (m, 2H), 2.74-2.56 (m, 2H), 1.99-1.82 (m, 2H), 1.39 (s, 3H); ^{13}C NMR (100 MHz, $CDCl_3$) δ 171.2, 154.0, 145.1, 142.7, 137.3, 134.7, 131.7, 128.6, 128.5, 128.4, 128.2, 128.1, 126.2, 125.8, 125.3, 123.2, 121.4, 74.0, 73.8, 68.8, 45.8, 30.7, 29.9, 28.8. FT-IR: ν (cm^{-1}) 3422, 2923, 1683, 1486, 1158, 1059, 906, 728, 696. HRMS [ESI] calcd for $C_{28}H_{29}NO_2SNa$ $[M+Na]^+$ 466.1811, found 466.1817.

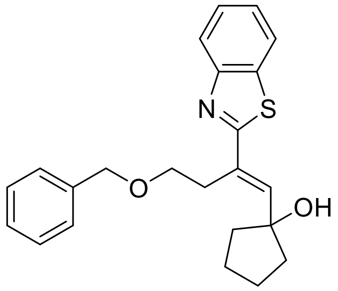

(*E*)-1-(2-(benzo[*d*]thiazol-2-yl)-4-(benzyloxy)but-1-en-1-yl)cyclopentan-1-ol (5u) was prepared by following **General Procedure A** as a colorless oil (38 mg, 51%, 72 h). ^{1}H NMR (400 MHz, $CDCl_3$) δ 7.93 (d, *J* = 8.0 Hz, 1H), 7.81 (d, *J* = 7.6 Hz, 1H), 7.44-7.40 (m, 1H), 7.35-7.31 (m, 1H), 7.30-7.24 (m, 5H), 6.71 (s, 1H), 4.51 (s, 2H), 4.26 (br, 1H), 3.76 (t, *J* = 5.6 Hz, 2H), 3.42 (t, *J* = 5.6 Hz, 2H), 1.88-1.65 (m, 8H); ^{13}C NMR (100 MHz, $CDCl_3$) δ 171.4, 153.9, 145.3, 137.5, 134.7, 131.5, 128.5, 128.0, 127.9, 126.1, 125.2, 123.1, 121.4, 82.1, 73.6, 68.8, 42.1, 29.1, 24.0. FT-IR: ν (cm^{-1}) 3430, 2956, 2341, 1483, 1434, 1058, 906, 757, 727. HRMS [ESI] calcd for $C_{23}H_{25}NO_2SNa$ $[M+Na]^+$ 402.1504, found 402.1499.

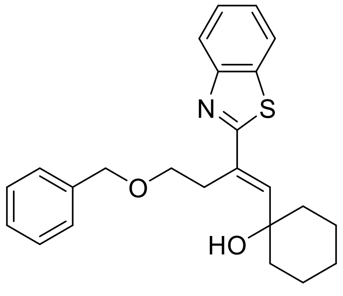

(*E*)-1-(2-(benzo[*d*]thiazol-2-yl)-4-(benzyloxy)but-1-en-1-yl)cyclohexan-1-ol (5v) was prepared by following **General Procedure A** as a colorless oil (40 mg, 52%, 72 h). ^{1}H NMR (400 MHz, $CDCl_3$) δ 7.93 (d, *J* = 8.0 Hz, 1H), 7.83 (d, *J* = 7.2 Hz, 1H), 7.45-7.41 (m, 1H), 7.38-7.33 (m, 1H), 7.30-7.23 (m, 5H), 6.62 (s, 1H), 4.50 (dd, *J* = 13.6, 12.4 Hz, 2H), 3.79-3.74 (m, 1H), 3.71-3.65 (m, 1H), 3.23-3.15 (m, 2H), 3.09-3.00 (m, 1H), 2.02 (d, *J* = 12.8 Hz, 1H), 1.80-1.64 (m, 3H), 1.59-1.50 (m, 1H), 1.33-1.14 (m, 2H), 1.10-0.95 (m, 2H); ^{13}C NMR (100 MHz, $CDCl_3$) δ 170.0, 153.9, 140.1, 137.5, 134.9, 134.6, 128.6, 128.0, 128.0, 126.1, 125.5, 123.2, 121.5, 73.5, 68.4, 43.2, 29.7, 29.1, 29.0, 26.7, 26.2, 26.1. FT-IR:

ν (cm^{-1}) 3420, 2923, 1733, 1486, 1240, 1094, 906, 758, 727. HRMS [ESI] calcd for $C_{24}H_{27}NO_2SNa$ [M+Na]$^+$ 416.1655, found 416.1662.

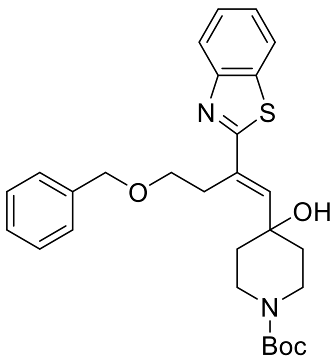

tert-butyl (*E*)-4-(2-(benzo[*d*]thiazol-2-yl)-4-(benzyloxy)but-1-en-1-yl)-4-hydroxypiperidine-1-carboxylate (5w) was prepared by following **General Procedure A** as a colorless oil (39 mg, 40%, 72 h). ^{1}H NMR (400 MHz, $CDCl_3$) δ 7.93 (d, *J* = 8.0 Hz, 1H), 7.82 (d, *J* = 7.6 Hz, 1H), 7.46-7.41 (m, 1H), 7.37-7.32 (m, 1H), 7.31-7.26 (m, 5H), 6.49 (s, 1H), 4.49 (s, 2H), 4.45 (br, 1H), 3.82-3.71 (m, 4H), 3.50-3.40 (m, 2H), 3.16-2.96 (m, 2H), 1.67-1.59 (m, 2H), 1.52-1.47 (m, 2H), 1.46 (s, 9H); ^{13}C NMR (100 MHz, $CDCl_3$) δ 171.1, 154.9, 153.9, 144.4, 137.1, 134.6, 131.5, 128.6, 128.3, 128.2, 126.2, 125.4, 123.1, 121.4, 79.4, 73.8, 71.0, 68.9, 37.8, 28.9, 28.6. FT-IR: ν (cm^{-1}) 3411, 2923, 2341, 1682, 1423, 1246, 1156, 1080, 907, 727. HRMS [ESI] calcd for $C_{28}H_{34}N_2O_4SNa$ [M+Na]$^+$ 517.2131, found 517.2141.

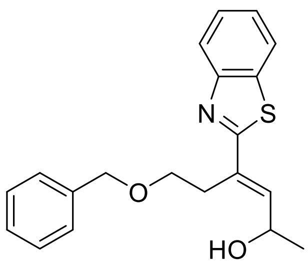

(*E*)-4-(benzo[*d*]thiazol-2-yl)-6-(benzyloxy)hex-3-en-2-ol (5x) was prepared by following **General Procedure A** as a colorless oil (44 mg, 66%, 72 h). ^{1}H NMR (400 MHz, $CDCl_3$) δ 7.95 (d, *J* = 8.0 Hz, 1H), 7.84 (d, *J* = 8.0 Hz, 1H), 7.46-7.42 (m, 1H), 7.38-7.34 (m, 1H), 7.32-7.25 (m, 5H), 6.59 (d, *J* = 8.0 Hz, 1H), 4.74-4.67 (m, 1H), 4.49 (dd, *J* = 14.0, 12.0 Hz, 2H), 3.82-3.77 (m, 1H), 3.74-3.68 (m, 1H), 3.38 (br, 1H), 3.23-3.17 (m, 1H), 3.10-3.02 (m, 1H), 1.37 (d, *J* = 6.4 Hz, 3H); ^{13}C NMR (100 MHz, $CDCl_3$) δ 169.9, 153.8, 141.9, 137.3, 134.6, 134.0, 128.6, 128.1, 128.1, 126.2, 125.5, 123.2, 121.5, 73.6, 68.5, 63.2, 29.7, 22.3. FT-IR: ν (cm^{-1}) 3410, 2970, 2340, 1734, 1435, 1363, 1241, 1060, 872, 758. HRMS [ESI] calcd for $C_{20}H_{21}NO_2SNa$ [M+Na]$^+$ 362.1185, found 362.1177.

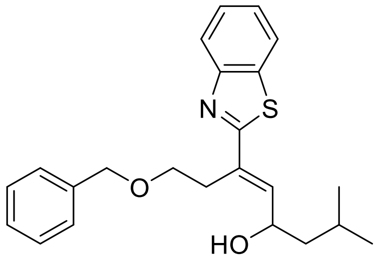

(*E*)-6-(benzo[*d*]thiazol-2-yl)-8-(benzyloxy)-2-methyloct-5-en-4-ol (5y) was prepared by following **General Procedure A** as a colorless oil (36 mg, 48%, 72 h). ^{1}H NMR (400 MHz, $CDCl_3$) δ 7.94 (d, *J* = 8.0 Hz, 1H), 7.83 (d, *J* = 7.2 Hz, 1H), 7.46-7.41 (m, 1H), 7.38-7.33 (m, 1H), 7.31-7.24 (m, 5H), 6.56 (d, *J* = 8.4 Hz, 1H), 4.59 (dd, *J* = 14.8, 7.2 Hz, 1H), 4.49 (t, *J* = 12.4 Hz, 2H), 3.82-3.76 (m, 1H), 3.73-3.66 (m, 1H), 3.29 (br, 1H), 3.25-3.19 (m, 1H), 3.11-3.03 (m, 1H), 1.81-1.71 (m, 1H), 1.70-1.62 (m, 1H), 1.48-1.40 (m, 1H), 0.95 (d, *J* = 6.4 Hz, 3H), 0.93 (d, *J* = 6.8 Hz, 3H); ^{13}C NMR (100 MHz, $CDCl_3$) δ 170.0, 153.8, 141.4, 137.4, 134.6, 134.4, 128.6, 128.1, 128.1, 126.1, 125.5, 123.2, 121.5, 73.6, 68.4, 65.4, 45.4, 29.7, 24.7, 23.2, 22.8. FT-IR: ν (cm^{-1}) 3421, 2954, 2341, 1734, 1435, 1242, 1077, 758, 728. HRMS [ESI] calcd for $C_{23}H_{27}NO_2SNa$ [M+Na]$^+$ 404.1660, found 404.1649.

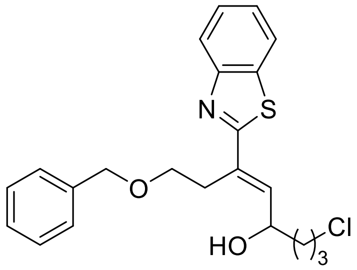

(*E*)-6-(benzo[*d*]thiazol-2-yl)-8-(benzyloxy)-1-chlorooct-5-en-4-ol (5z) was prepared by following **General Procedure A** as a colorless oil (36 mg, 45%, 72 h). ^{1}H NMR (400 MHz, $CDCl_3$) δ 7.94 (d, *J* = 7.6 Hz, 1H), 7.84 (d, *J* = 8.0 Hz, 1H), 7.47-7.41 (m, 1H), 7.39-7.34 (m, 1H), 7.32-7.23 (m, 5H), 6.57 (d, *J* = 8.4 Hz, 1H), 4.57-4.52 (m, 1H), 4.49 (s, 2H), 3.82-3.77 (m, 1H), 3.74-3.67 (m, 1H), 3.63-3.54 (m, 2H), 3.42 (br, 1H), 3.25-3.18 (m, 1H), 3.10-3.01 (m, 1H), 2.02-1.92 (m, 1H), 1.90-1.76 (m, 3H); ^{13}C NMR (100 MHz, $CDCl_3$) δ 169.7, 153.8, 140.3, 137.3, 135.0, 134.6, 128.6, 128.1, 126.2, 125.6, 123.3, 121.5, 73.6, 68.4, 66.5, 45.2, 33.6, 29.8, 28.9. FT-IR: ν (cm^{-1}) 3396, 2927, 1733, 1485, 1371, 1241, 1058, 758, 729, 698. HRMS [ESI] calcd for $C_{22}H_{24}ClNO_2SNa$ [M+Na]$^+$ 424.1114, found 424.1111.

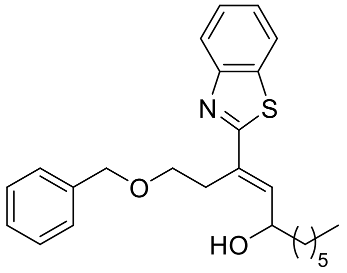

(*E*)-3-(benzo[*d*]thiazol-2-yl)-1-(benzyloxy)undec-3-en-5-ol (5aa) was prepared by following **General Procedure A** as a colorless oil (28 mg, 35%, 72 h). ^{1}H NMR (400 MHz, $CDCl_3$) δ 7.94 (d, *J* = 8.0 Hz, 1H), 7.84 (d, *J* = 7.2 Hz, 1H), 7.47-7.41 (m, 1H), 7.39-7.33 (m, 1H), 7.32-7.23 (m, 5H), 6.57 (d, *J* = 8.0 Hz, 1H), 4.55-4.46 (m, 3H), 3.83-3.75 (m, 1H), 3.74-3.66 (m, 1H), 3.31 (br, 1H), 3.25-3.16 (m, 1H), 3.11-3.01 (m, 1H), 1.79-1.67 (m, 1H), 1.46-1.38 (m, 1H), 1.37-1.24 (m, 8H), 0.88 (t, *J* = 6.8 Hz, 3H); ^{13}C NMR (100 MHz, $CDCl_3$) δ 170.0, 153.9, 141.2, 137.4, 134.7, 134.6, 128.6, 128.1, 128.1, 126.1, 125.5, 123.2, 121.5, 73.6, 68.4, 67.2, 36.5, 32.0, 29.8, 29.4, 25.6, 22.8, 14.2. FT-IR: ν (cm^{-1}) 3321, 2976, 1987, 1745, 1367, 1234, 1056, 987, 775. HRMS [ESI] calcd for $C_{25}H_{31}NO_2SNa$ [M+Na]$^+$ 432.1968, found 432.1975.

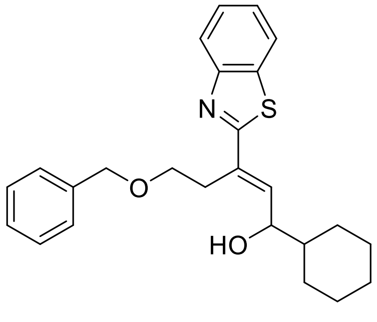

(*E*)-3-(benzo[*d*]thiazol-2-yl)-5-(benzyloxy)-1-cyclohexylpent-2-en-1-ol (5ab) was prepared by following **General Procedure A** as a colorless oil (51 mg, 63%, 72 h). ^{1}H NMR (400 MHz, $CDCl_3$) δ 7.94 (d, *J* = 8.0 Hz, 1H), 7.83 (d, *J* = 8.0 Hz, 1H), 7.46-7.41 (m, 1H), 7.39-7.33 (m, 1H), 7.31-7.21 (m, 5H), 6.63 (d, *J* = 8.4 Hz, 1H), 4.50 (dd, *J* = 13.6, 12.4 Hz, 2H), 4.23 (t, *J* = 8.0 Hz, 1H), 3.80-3.74 (m, 1H), 3.72-3.65 (m, 1H), 3.25-3.16 (m, 2H), 3.12-3.01 (m, 1H), 2.07-2.00 (m, 1H), 1.82-1.63 (m, 4H), 1.61-1.50 (m, 1H), 1.34-1.14 (m, 3H), 1.10-0.94 (m, 2H). ^{13}C NMR (100 MHz, $CDCl_3$) δ 170.0, 153.9, 140.2, 137.5, 134.9, 134.6, 128.6, 128.0, 128.0, 126.1, 125.5, 123.2, 121.5, 73.5, 71.5, 68.4, 43.3, 29.7, 29.1, 29.0, 26.7, 26.2, 26.1. FT-IR: ν (cm^{-1}) 3421, 2921, 1487, 1361, 1188, 1124, 906, 757, 727. HRMS [ESI] calcd for $C_{25}H_{29}NO_2SNa$ [M+Na]$^+$ 430.1811, found 430.1807.

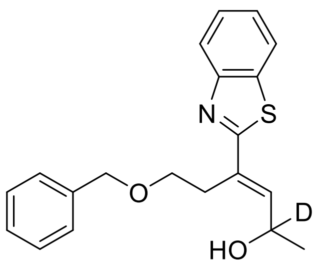

(*E*)-4-(benzo[*d*]thiazol-2-yl)-6-(benzyloxy)hex-3-en-2-d-2-ol (5ac) was prepared by following **General Procedure A** as a colorless oil (42 mg, 62%, 72 h). ^{1}H NMR (400 MHz, $CDCl_3$) δ 7.95 (d, *J* = 7.6 Hz, 1H), 7.84 (d, *J* = 8.0 Hz, 1H), 7.47-7.42 (m, 1H), 7.39-7.33 (m, 1H), 7.32-7.22 (m, 5H), 6.58 (s, 1H), 4.49 (dd, *J* = 14.0, 12.0 Hz, 2H), 3.83-3.78 (m, 1H), 3.75-3.68 (m, 1H), 3.35 (br, 1H), 3.23-3.17 (m,

1H), 3.11-3.02 (m, 1H), 1.36 (s, 3H); ^{13}C NMR (100 MHz, $CDCl_3$) δ 169.9, 153.9, 141.8, 137.4, 134.7, 134.1, 128.6, 128.1, 128.1, 126.1, 125.5, 123.3, 121.5, 73.6, 68.5, 62.8 (t, $J_{C\text{-}D}$ = 21.7 Hz), 29.7, 22.2. FT-IR: ν (cm^{-1}) 3397, 2970, 2244, 1734, 1557, 1362, 1242, 1076, 906, 758, 727. HRMS [ESI] calcd for $C_{20}H_{20}DNO_2SNa$ $[M+Na]^+$ 363.1248, found 363.1239.

5. Transformations of 3a

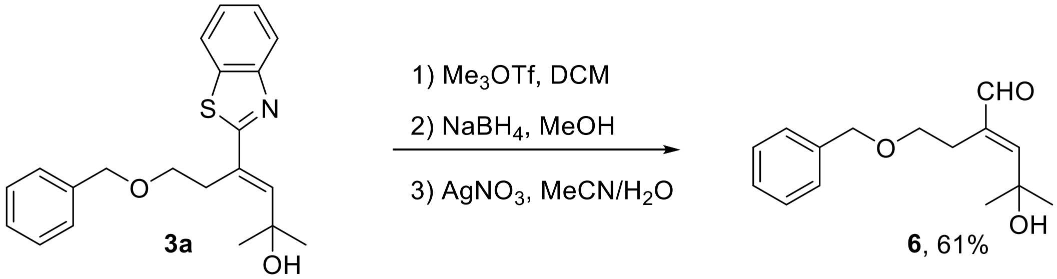

3a (0.2 mmol), activated 4 Å powdered molecular sieves (300 mg), and anhydrous CH_2Cl_2 (2 mL) was stirred at room temperature for 10 min, and then Me_3OTf (1.0 mmol) was added in twice. The suspension was stirred at room temperature for 4 h and then concentrated to dryness without filtering off the molecular sieves. To a cooled (0 °C) stirred suspension of the crude N-methylbenzothiazolium salt in CH_3OH (2 mL) was added $NaBH_4$ (0.5 mmol). The mixture was stirred at room temperature. For an additional 30 min, diluted with acetone, filtered through a pad of Celite, and concentrated. To a vigorously stirred solution of the crude benzothiazoline in CH_2Cl_2 (0.6 mL) and CH_3CN (3.0 mL) were added H_2O (0.36 mL) and then $AgNO_3$ (0.6 mmol). The mixture was stirred at room temperature until the benzothiazoline were completely consumed as determined by TLC, and then diluted with 1 M phosphate buffer at pH 7 (0.1 mL). Stirring was continued for an additional 15 min, and then the reaction mixture was diluted with 1 M phosphate buffer at pH 7 (5 mL) and the suspension was extracted with EtOAc (2 × 10 mL), and the combined organic layers were dried over Na_2SO_4, filtered through a pad of Celite, and concentrated. The residue was eluted from a short column of silica gel (EtOAc/petroleum ether =1:40) to give the corresponding product **6**.

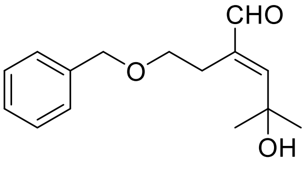

(*E*)-2-(2-(benzyloxy)ethyl)-4-hydroxy-4-methylpent-2-enal (6): Colorless oil. ^{1}H NMR (400 MHz, $CDCl_3$) δ 9.36 (s, 1H), 7.36-7.24 (m, 5H), 6.50 (s, 1H), 4.51 (s, 1H), 4.48 (s, 2H), 3.52 (t, *J* = 5.4 Hz, 2H), 2.92 (t, *J* = 5.4 Hz, 2H), 1.29 (s, 6H). ^{13}C NMR (100 MHz, $CDCl_3$) δ 195.8, 163.6, 137.1, 136.8, 128.6, 128.3, 128.2, 73.7, 72.2, 68.4, 30.2, 23.5. FT-IR: ν (cm^{-1}) 3430, 2974, 2866, 2360, 1684, 1325, 1091, 1055, 739, 698. HRMS [ESI] calcd for $C_{15}H_{20}O_3Na$ $[M+Na]^+$ 271.1305, found 271.1308.

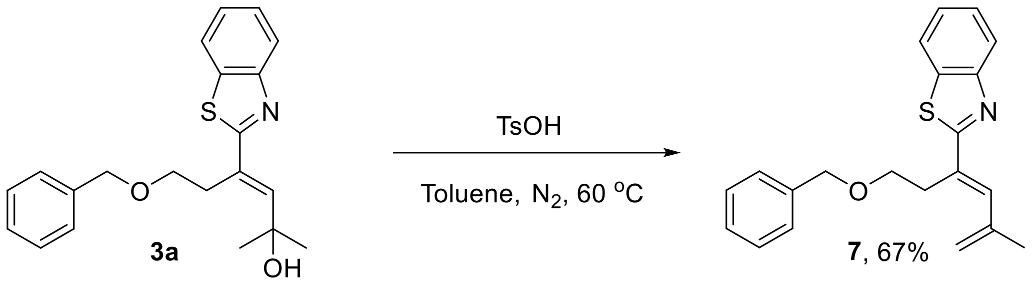

3a (0.2 mmol) and TsOH (0.1 mmol) were loaded in a flask, which was subjected to evacuation/ flushing with N_2 for 3 times. Toluene (2 mL) was added to the mixture via syringe. The resulting mixture was stirred at 60°C for 12 h. Then the mixture was quenched with H_2O. The aqueous layer was extracted with EtOAc. The organic layer was washed with brine, dried over Na_2SO_4, concentrated in vacuo, and purified by flash column chromatography on silica gel to give the corresponding product **7**.

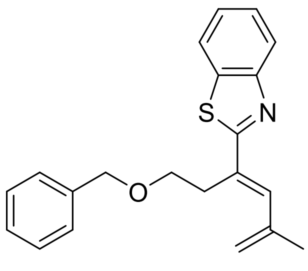

(*E*)-2-(1-(benzyloxy)-5-methylhexa-3,5-dien-3-yl)benzo[*d*]thiazole (7): Colorless oil. ^{1}H NMR (400 MHz, $CDCl_3$) δ 7.98 (d, *J* = 8.0 Hz, 1H), 7.83 (d, *J* = 7.6 Hz, 1H), 7.47-7.42 (m, 1H), 7.37-7.33 (m, 1H), 7.31-7.23 (m, 5H), 7.00 (s, 1H), 5.27-5.24 (m, 1H), 5.23-5.20 (m, 1H), 4.53 (s, 2H), 3.76 (t, *J* = 7.2 Hz, 2H), 3.28 (t, *J* = 7.2 Hz, 2H), 2.03 (s, 3H); ^{13}C NMR (100 MHz, $CDCl_3$) δ 170.9, 154.0, 140.8, 138.7, 138.4, 134.7, 132.5, 128.4, 127.6, 127.5, 126.1, 125.3, 123.2, 121.4, 119.4, 72.9, 69.5, 30.4, 23.1. FT-IR: ν (cm^{-1}) 3234, 2324, 1731, 1556, 1455, 1267, 1125, 934, 775. HRMS [ESI] calcd for $C_{21}H_{21}NOSNa$ $[M+Na]^+$ 358.1236, found 358.1228.

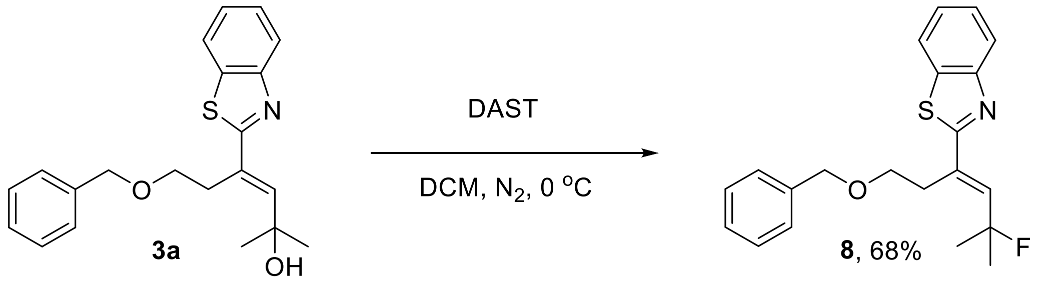

To a flame-dried Schlenk tube was added **3a** (0.2 mmol), which was subjected to evacuation/ flushing with N_2 for 3 times. Dry CH_2Cl_2 (2 mL) was added to the mixture via syringe. The resulting mixture was stirred at 0°C for 10 min. The resulting mixture was added DAST (0.3 mmol) and stirred at 0°C for 30 min. Then the reaction mixture was poured into a cold sat. $NaHCO_3$ solution (30 mL) and extracted with CH_2Cl_2 (3 x 10 mL), washed with brine (10 mL), and dried over anhydrous Na_2SO_4. After filtration and concentration under reduced pressure, the crude product was purified by flash column chromatography on silica gel to afford **8**.

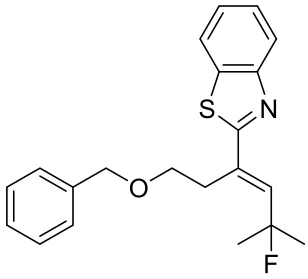

(E)-2-(1-(benzyloxy)-5-fluoro-5-methylhex-3-en-3-yl)benzo[d]thiazole (8): Colorless oil. ^{1}H NMR (400 MHz, $CDCl_3$) δ 7.99 (d, J = 8.4 Hz, 1H), 7.84 (d, J = 8.0 Hz, 1H), 7.49-7.43 (m, 1H), 7.39-7.34 (m, 1H), 7.31-7.21 (m, 5H), 6.58 (d, $J_{F\text{-}H}$ = 22.8 Hz, 1H), 4.54 (s, 2H), 3.75 (t, J = 7.2 Hz, 2H), 3.27 (t, J = 7.2 Hz, 2H), 1.62 (d, $J_{F\text{-}H}$ = 21.2 Hz, 6H); ^{13}C NMR (100 MHz, $CDCl_3$) δ 170.5, 153.8, 139.2 (d, $J_{C\text{-}F}$ = 19.9 Hz), 138.7, 134.8, 133.0 (d, $J_{C\text{-}F}$ = 19.5 Hz), 128.4, 127.6, 127.5, 126.2, 125.4, 123.3, 121.5, 94.8 (d, $J_{C\text{-}F}$ = 170 Hz), 72.8, 69.7, 30.4 (d, $J_{C\text{-}F}$ = 6.7 Hz), 28.5 (d, $J_{C\text{-}F}$ = 25.4 Hz); ^{19}F NMR (377 MHz, $CDCl_3$) δ -137.3. FT-IR: ν (cm^{-1}) 3432, 2921, 1487, 1435, 1312, 1155, 963, 757, 727. HRMS [ESI] calcd for $C_{21}H_{22}FNOSNa$ $[M+Na]^+$ 378.1298, found 378.1295.

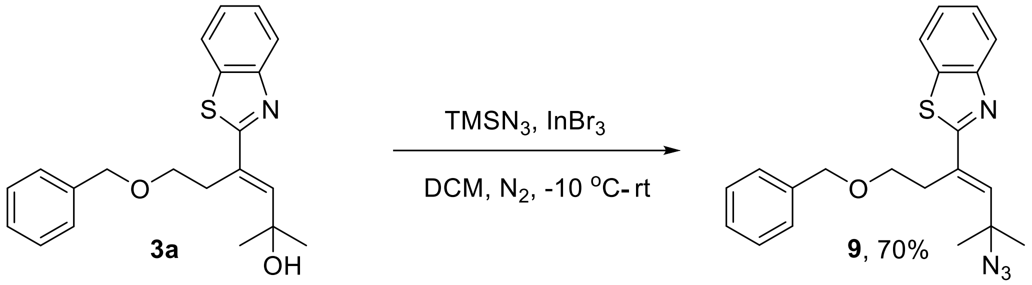

A solution of alcohol **3a** (0.2 mmol) and $TMSN_3$ (3 mmol) in dry CH_2Cl_2 (2 mL) were placed in a 100 mL Schlenk tube, the tube was degassed with N_2 for 3 times. $InBr_3$ (0.2 mmol) was added to this reaction mixture. The mixture was stirred at -10 °C for 10 min. Then the temperature gradually was raised to 25 °C, and the stirring was continued until the reaction finished (a few min in general) as indicated by TLC analysis. The product was extracted with CH_2Cl_2 (3×5 mL). The combined CH_2Cl_2 phases were washed sequentially with 10% aqueous Na_2CO_3 solution (5 mL) and water (10 mL), and dried over anhydrous Na_2SO_4. After filtration and concentration under reduced pressure, the crude product was purified by flash column chromatography on silica gel to afford **9**.

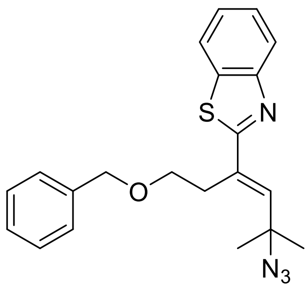

(E)-2-(5-azido-1-(benzyloxy)-5-methylhex-3-en-3-yl)benzo[d]thiazole (9): Colorless oil. ^{1}H NMR (400 MHz, $CDCl_3$) δ 7.98 (d, J = 8.0 Hz, 1H), 7.83 (d, J = 8.0 Hz, 1H), 7.49-7.43 (m, 1H), 7.39-7.34 (m, 1H), 7.31-7.20 (m, 5H), 6.50 (s, 1H), 4.53 (s, 2H), 3.75 (t, J = 7.2 Hz, 2H), 3.35 (t, J = 7.2 Hz, 2H), 1.56 (s, 6H); ^{13}C NMR (100 MHz, $CDCl_3$) δ 170.1, 153.8, 139.7, 138.6, 135.2, 134.7, 128.4, 127.7, 127.6, 126.3, 125.5, 123.4, 121.5, 73.0, 69.1, 61.4, 30.4, 28.4. FT-IR: ν (cm^{-1}) 3323, 2643, 1834, 1654, 1501, 1365, 1178, 958, 785. HRMS [ESI] calcd for $C_{21}H_{22}N_4OSNa$ $[M+Na]^+$ 401.1407, found 401.1416.

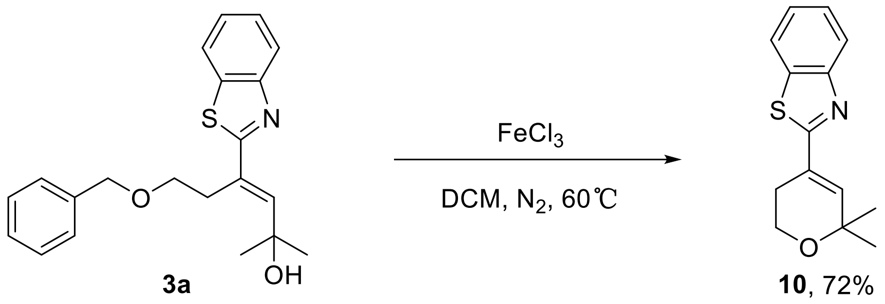

3a (0.2 mmol) and $FeCl_3$ (0.02 mmol) were loaded in a flask, which was subjected to evacuation/ flushing with N_2 for 3 times. CH_2Cl_2 (2 mL) was added to the mixture via syringe. The resulting mixture was stirred at 60°C for 12 h. The then mixture was quenched with H_2O. The aqueous layer was extracted with CH_2Cl_2. The organic layer was washed with brine, dried over Na_2SO_4, concentrated in vacuo, and purified by flash column chromatography on silica gel to give the corresponding product **10**.

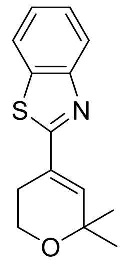

2-(6,6-dimethyl-3,6-dihydro-2*H*-pyran-4-yl)benzo[*d*]thiazole (10): Colorless oil. ^{1}H NMR (400 MHz, $CDCl_3$) δ 7.99 (d, *J* = 8.4 Hz, 1H), 7.84 (d, *J* = 8.0 Hz, 1H), 7.48-7.43 (m, 1H), 7.38-7.33 (m, 1H), 6.63 (t, *J* = 1.6 Hz, 1H), 3.96 (t, *J* = 5.4 Hz, 2H), 2.75-2.70 (m, 2H), 1.39 (s, 6H); ^{13}C NMR (100 MHz, $CDCl_3$) δ 168.8, 153.8, 138.9, 134.3, 129.7, 126.3, 125.4, 123.2, 121.6, 72.3, 59.6, 27.3, 26.6. FT-IR: ν (cm^{-1}) 3305, 2973, 2244, 1731, 1483, 1433, 1308, 1070, 964, 754, 725. HRMS [ESI] calcd for $C_{14}H_{16}NOS$ $[M+H]^+$ 246.0947, found 246.0939.

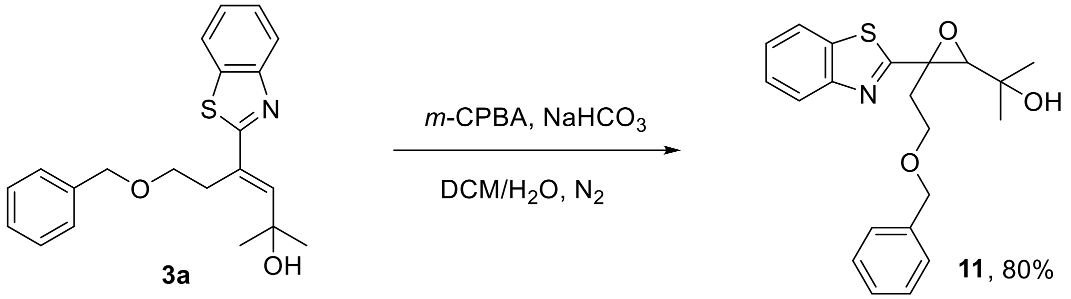

A solution of **3a** (0.2 mmol) and *m*-chloroperoxybenzoic acid (2.2 mmol) in CH_2Cl_2 (2.0 mL) was added aq. $NaHCO_3$ (3 N, 0.1 mL). The resulted mixture was stirred at room temperature for 20 h. Then the reaction mixture was treated with 2 N Na_2CO_3 (5.0 mL). The organic phase was extracted with CH_2Cl_2, and concentrated under reduced pressure. The residue was purified by flash column chromatography on silica gel to give the corresponding product **11**.

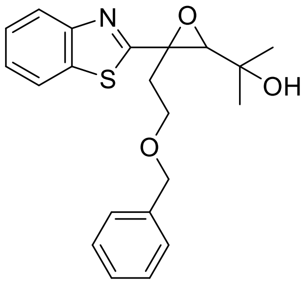

2-(3-(benzo[*d*]thiazol-2-yl)-3-(2-(benzyloxy)ethyl)oxiran-2-yl)propan-2-ol (11): Colorless oil. ^{1}H NMR (400 MHz, $CDCl_3$) δ 7.99 (d, *J* = 8.0 Hz, 1H), 7.86 (d, *J* = 7.6 Hz, 1H), 7.51-7.45 (m, 1H), 7.41-7.36 (m, 1H), 7.25-7.16 (m, 5H), 4.51 (d, *J* = 11.6 Hz, 1H), 4.46 (d, *J* = 11.6 Hz, 1H), 3.95-3.88 (m, 1H), 3.79-3.72 (m, 1H), 3.04 (s, 1H), 3.03-2.96 (m, 1H), 2.95-2.87 (m, 1H), 1.43 (s, 3H), 1.37 (s, 3H); ^{13}C NMR (100 MHz, $CDCl_3$) δ 174.1, 153.8, 138.0, 134.9, 128.4, 127.7, 127.6, 126.2, 125.3, 123.1, 121.9, 73.1, 68.7, 66.8, 63.5, 30.0, 29.2, 25.7. FT-IR: ν (cm^{-1}) 2973, 2145, 1731, 1546, 1237, 1124, 945, 754, 723. HRMS [ESI] calcd for $C_{21}H_{23}NO_3SNa$ $[M+Na]^+$ 392.1291, found 392.1287.

6. Single-crystal X-ray diffraction analysis of 3ai

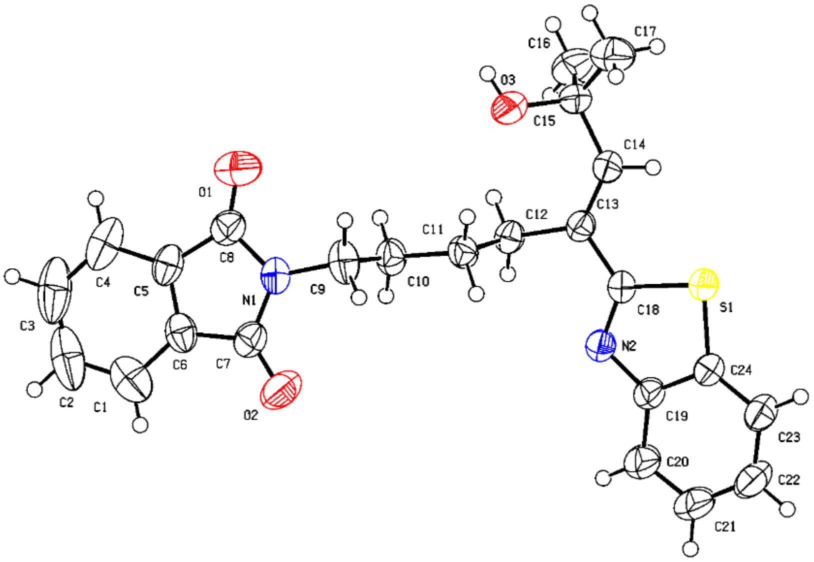

Figure S8. Single-crystal X-ray of 3ai

Bond precision:	C-C = 0.0033 A		Wavelength=0.71073
Cell:	a=8.3959(3)	b=9.9482(4)	c=13.7861(5)
	alpha=85.8973(19)	beta=87.8852(18)	gamma=69.4629(19)
Temperature:	296 K		
	Calculated	Reported	
Volume	1075.44(7)	1075.44(7)	
Space group	P -1	P -1	
Hall group	-P 1	-P 1	
Moiety formula	C24 H24 N2 O3 S	C24 H24 N2 O3 S	
Sum formula	C24 H24 N2 O3 S	C24 H24 N2 O3 S	
Mr	420.51	420.51	
Dx,g cm-3	1.29	1.299	

Z	2	2
Mu (mm-1)	0.178	0.178
F000	444.0	444.0
F000'	444.43	
h,k,lmax	10,12,17	10,12,17
Nref	4973	4965
Tmin,Tmax	0.918,0.965	0.652,0.746
Tmin'	0.899	

Correction method = # Reported T Limits: Tmin=0.652 Tmax=0.746
AbsCorr = MULTI-SCAN

Data completenesss = 0.998	Theta(max) = 27.590
R(reflections) = 0.0562 (3355)	wR2(reflections) = 0.1453 (4965)
S = 1.019	Npar = 262

7. Mechanistic studies

7.1. Radical trap experiment

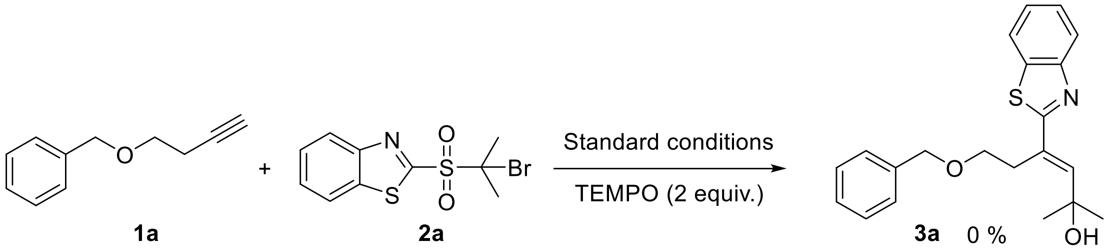

1a 2a 3a 0 %

A flame-dried sealed tube was charged with **1** (0.4 mmol, 2.0 equiv.), **2** (0.2 mmol, 1.0 equiv.), TEMPO (2.0 equiv.), NaOAc (0.1 mmol, 0.5 equiv.) and *fac*-Ir(ppy)$_3$ (0.002 mmol), which was subjected to evacuation/ flushing with N_2 for 3 times. Then acetone (2 mL) and H_2O (0.6 mL) were added to the mixture via syringe under N_2. The sealed tube was placed 5 cm away from the light source and stirred at 25 °C. Subsequently, the reaction was irradiated with 30 W 510 nm green LED. No product was produced as determined by TLC.

7.2. Radical clock experiment

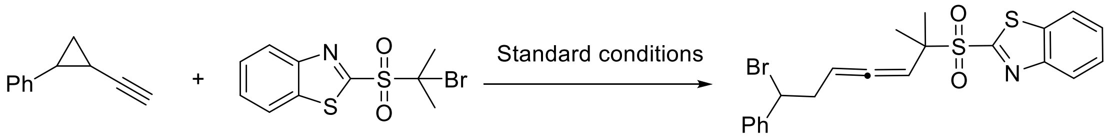

12 2a 13, 70%

A flame-dried sealed tube was charged with 1 (0.4 mmol, 2.0 equiv.), 2 (0.2 mmol, 1.0 equiv.), NaOAc (0.1 mmol, 0.5 equiv.), and *fac*-Ir(ppy)$_3$ (0.002 mmol), which was subjected to evacuation/ flushing with nitrogen for three times. Then acetone (2 mL) and H_2O (0.6 mL) were added to the mixture via syringe under N_2. The sealed tube was placed 5 cm away from the light source, which

was irradiated by 30 W green LED and stirred at room temperature until the starting material had been consumed as determined by TLC. The mixture was quenched with H_2O. The aqueous layer was extracted with EtOAc. The organic layer was washed with brine, dried over Na_2SO_4, concentrated in vacuo, and purified by flash column chromatography on silica gel to give the corresponding product **13**.

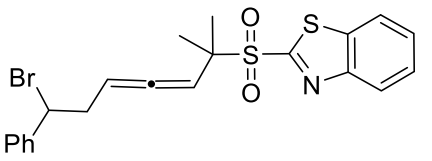

2-((7-bromo-2-methyl-7-phenylhepta-3,4-dien-2-yl)sulfonyl)benzo[*d*]thiazole (13): The ratio of *Z*-allene and *E*-allene could not be determined by NMR analysis. Colorless oil. ^{1}H NMR (400 MHz, $CDCl_3$) δ 8.26-8.21 (m, 1H, two isomers), 8.01-7.95 (m, 1H, two isomers), 7.63-7.52 (m, 2H, two isomers), 7.31-7.23 (m, 3H, two isomers), 7.20-7.13 (m, 2H, two isomers), 5.42-5.36 (m, 1H, two isomers), 5.32-5.23 (m, 1H, two isomers), 4.68-4.60 (m, 1H, two isomers), 2.28-2.16 (m, 2H, two isomers), 1.60-1.56 (m, 6H, two isomers); ^{13}C NMR (100 MHz, $CDCl_3$) δ 207.1 & 207.1 (two isomers), 163.9 & 163.9 (two isomers), 153.0 & 153.0 (two isomers), 143.6 (overlap, two isomers), 137.5 (overlap, two isomers), 128.5 (overlap, two isomers), 128.1 & 128.1 (two isomers), 127.7 & 127.7 (two isomers), 127.6 & 127.6 (two isomers), 125.8 (overlap, two isomers), 125.7 (overlap, two isomers), 122.2 (overlap, two isomers), 92.3 (overlap, two isomers), 91.8 & 91.8 (two isomers), 73.3 & 73.1 (two isomers), 65.3 & 65.1 (two isomers), 38.2 & 38.1 (two isomers), 22.1 & 22.0 (two isomers), 21.3 & 21.2 (two isomers). FT-IR: ν (cm^{-1}) 3525, 2924, 2250, 1963, 1493, 1314, 1159, 908, 760, 728. HRMS [ESI] calcd for $C_{21}H_{21}BrNO_2S_2$ $[M+H]^+$ 462.0192, found 462.0183.

7.3. Tautomerization of *Z/E* isomers

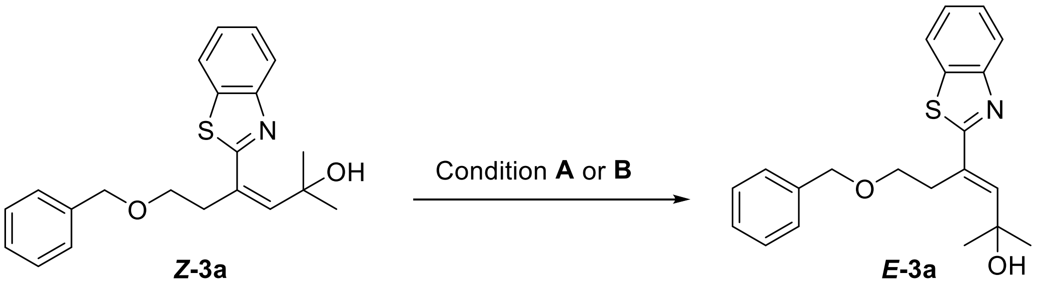

***Z*-3a** ***E*-3a**

Condition A: A flame-dried sealed tube was charged with ***Z*-3a** (0.1 mmol), which was subjected to evacuation/ flushing with N_2 for 3 times. Then acetone (2 mL) was added to the mixture via syringe under N_2. The sealed tube was placed 5 cm away from the light source, which was irradiated by 30 W green LED and stirred at room temperature. No ***E*-3a** was produced as determined by TLC.

Condition B: A flame-dried sealed tube was charged with ***Z*-3a** (0.1 mmol) and *fac*-Ir(ppy)$_3$ (0.002 mmol), which was subjected to evacuation/ flushing with N_2 for 3 times. Then acetone (2 mL) and H_2O (0.6 mL) were added to the mixture via syringe under N_2. The sealed tube was placed 5 cm away from the light source, which was irradiated by 30 W green LED and stirred at room temperature until the starting material had been consumed as determined by TLC. The mixture was quenched with H_2O. The aqueous layer was extracted with EtOAc. The organic layer was washed with brine, dried over Na_2SO_4, concentrated in vacuo, and purified by flash column chromatography on silica gel (eluent: ethyl acetate/petroleum ether) to give the corresponding product ***E*-3a** (80%).

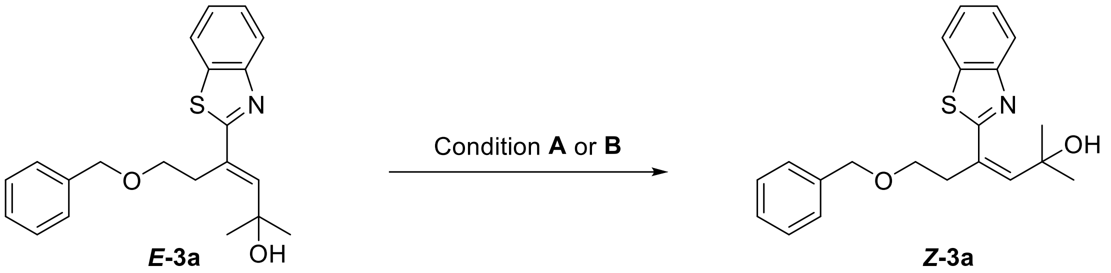

Condition A: A flame-dried sealed tube was charged with ***E*-3a** (0.1 mmol), which was subjected to evacuation/ flushing with N_2 for 3 times. Then acetone (2 mL) was added to the mixture via syringe under N_2. The sealed tube was placed 5 cm away from the light source, which was irradiated by 30 W green LED and stirred at room temperature. No ***Z*-3a** was produced as determined by TLC.
Condition B: A flame-dried sealed tube was charged with ***E*-3a** (0.1 mmol) and *fac*-Ir(ppy)$_3$ (0.002 mmol), which was subjected to evacuation/ flushing with N_2 for 3 times. Then acetone (2 mL) was added to the mixture via syringe under N_2. The sealed tube was placed 5 cm away from the light source, which was irradiated by 30 W green LED and stirred at room temperature. Only trace ***Z*-3a** was produced as determined by TLC.

7.4. Light on/off experiments

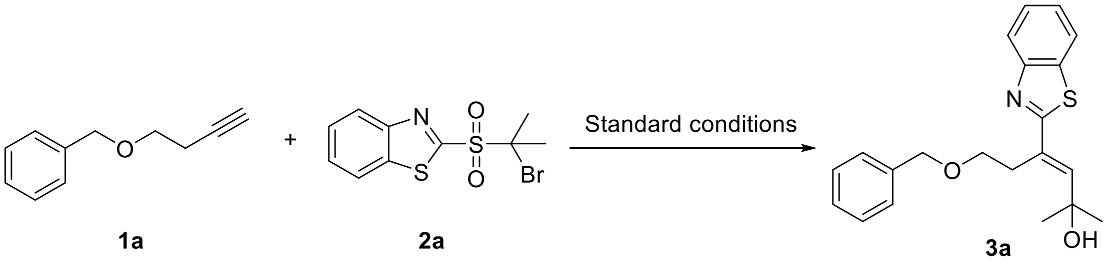

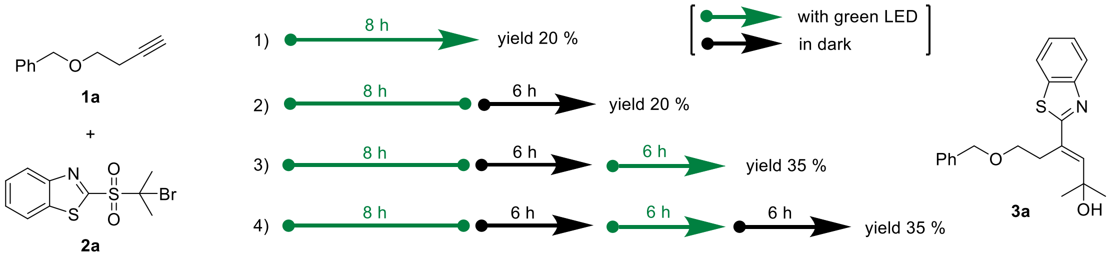

Figure S9. Time picture of the transformation with the light on/off over time.

7.5. Fluorescence quenching experiments (Stern-Volmer study)

Emission intensities were recorded using a FLS980 (Edinburgh Instrument, UK) luminescence spectrophotometer. All *fac*-Ir(ppy)$_3$ solutions were excited at 460 nm and the emission intensity was collected at 520 nm. In a typical experiment, to a $3{\cdot}10^{-6}$ M solution of *fac*-Ir(ppy)$_3$ in acetone was added the appropriate amount of a quencher Unactivated alkynes S1 in a screw-top quartz cuvette. After degassing the sample with a stream of N_2 for 10 minutes, the emission of the sample was collected. Then, another quencher difunctional reagent T36 was test in the same way.

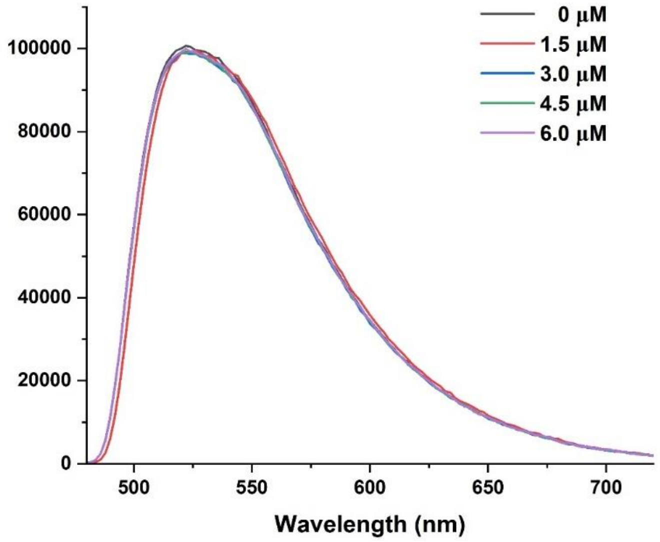

Figure S10. Quenching the emission of *fac*-Ir(ppy)$_3$ by increasing the amount of alkyne 1a.

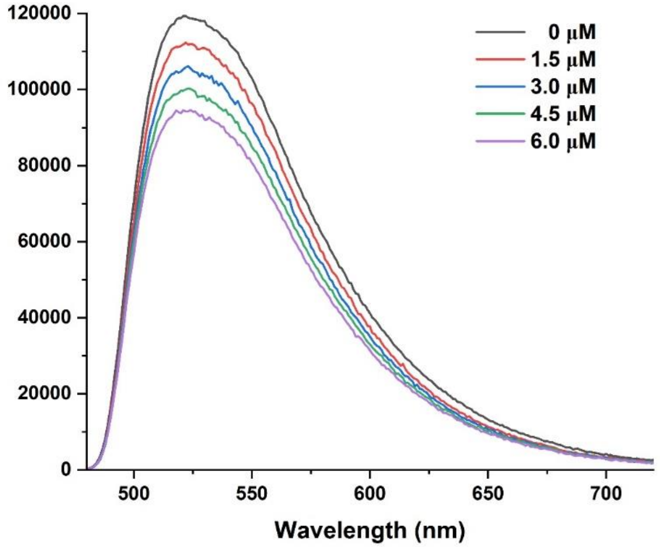

Figure S11. Quenching the emission of *fac*-Ir(ppy)$_3$ by increasing the amount of difunctional reagent 2l.

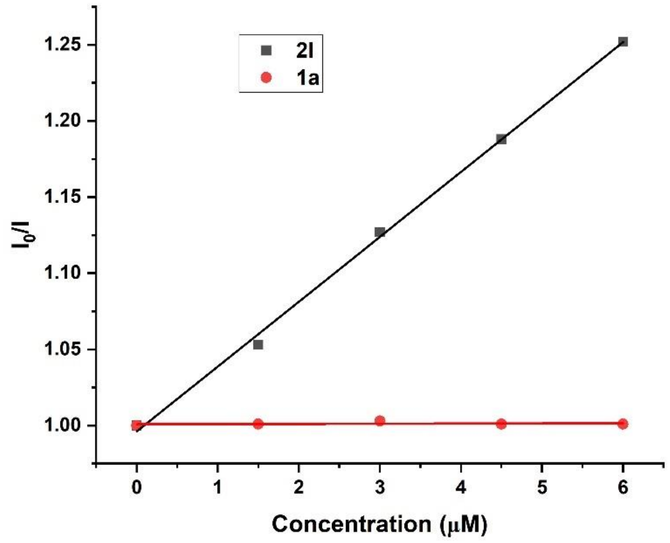

Figure S12. Stern-Volmer quenching plot.

7.5. Quantum yield experiment

7.5.1. Determination of the light intensity at 510 nm[14-15]

The photon flux of the Green LED (30 W, λ max = 510 nm) was determined by standard ferrioxalate actinometry following a modified literature procedure of Yoon[3] and Glorius[4]. Therefore, two solutions were prepared which were stored in the dark. All following steps were carried out in a dark environment aiming to prevent undesired light pollution. A solution of ferrioxalate (0.15 M) was prepared by dissolving potassium ferrioxalate hydrate (1.47 g) in aq. H_2SO_4 (0.05 M, 20 mL). Further, a buffered solution of 1,10-phenanthroline was prepared by dissolving 1,10-Phenanthroline (20.0 mg) and sodium acetate (water-free, 2.71 g) in aq. H_2SO_4 (0.5 M, 25 mL).

To determine the photon flux of the light and therefore the reduction of $[Fe(C_2O_4)_3]^{3-}$ into $[Fe(C_2O_4)_2]^{2-}$, a defined time is measured. The ferrioxalate solution (1.0 mL) was placed in a 4.0 mL vial and irradiated for 60 s at λ max = 510 nm. After irradiation, the phenanthroline solution (175 μL) was added and the mixture stirred in the dark for 1 h. This allows the ferrous ions to be completely coordinated by the phenanthroline. The solution was transferred to a quartz cuvette and the absorption of the solution was measured at 510 nm. The same procedure was repeated two more times. A non-irradiated sample was also prepared and the absorption at 510 nm was measured. The average of the absorption of the irradiated and non-irradiated samples was determined and used to calculate the generated amount of Fe (II) according to the Lambert-Beer law (equation 1),

$$n_{Fe(II)} = \frac{V \cdot \Delta A_{510nm}}{l \cdot \varepsilon} \qquad (1)$$

where V is the total volume ($1.175*10^{-3}$ L), ΔA_{510nm} the difference between absorbance of irradiated samples and the non-irradiated (control) ones (at λ = 510 nm), l is the path length of the cuvette (1.0 cm), and ε is the molar attenuation coefficient of the ferrioxalate actinometer λ = 510 nm (11100 L· mol^{-1}·cm^{-1}). The photonflux (ϕ_q) can be calculated using equation 2,

$$\emptyset_q = \frac{n_{Fe(II)}}{\emptyset_F \cdot t \cdot f} \qquad (2)$$

where Φ_F is the quantum yield of the ferrioxalate actinometer (1.12 at λ = 416 nm) and t is the irradiation time (60 s). The fraction of light absorbed at λ = 510 nm by the actinometer (*f*) is calculated by using equation 3. $A_{427\text{nm}}$ is the absorbance of the ferrioxalate solution at λ = 510 nm.

$$f = 1 - 10^{-A_{427nm}} \qquad (3)$$

The absorbance ($A_{510\text{nm}}$) of the ferrioxalate solution was measured to be > 3 indicating that > 99.9% of the photons are absorbed (f> 0.999). The photon flux Φ_q was therefore calculated to be 7.57*10^{-10} einstein s^{-1} as an average of three experiments.

7.5.2. Determination of the quantum yield of the reaction

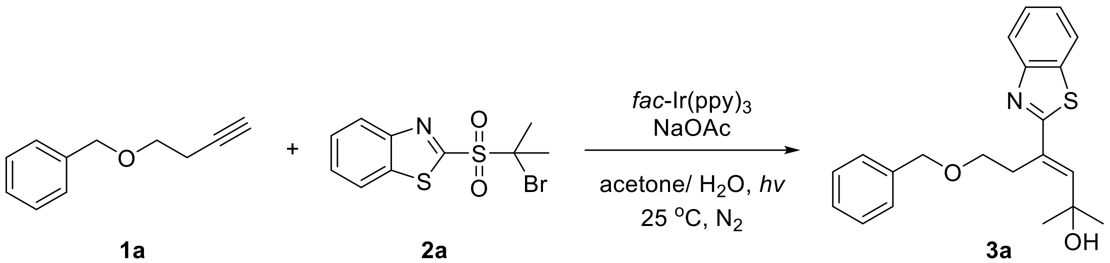

1a **2a** **3a**

A flame-dried sealed tube was charged with **1a** (0.4 mmol, 2.0 equiv.), **2a** (0.2 mmol, 1.0 equiv.) and NaOAc (0.1 mmol, 0.5 equiv.), which was subjected to evacuation/ flushing with N_2 for 3 times. Then acetone (2 mL) and H_2O (0.6 mL) were added to the mixture via syringe under N_2. The sealed tube was placed 5 cm away from the light source and stirred at 25 °C. Subsequently, the reaction was irradiated with 30 W 510 nm green LED for 10 h. The reaction yield after 10 h was determined to be 15% (1.5*10^{-5} mol). The reaction quantum yield (ϕ) was determined using equation 4, where the photon flux is 7.57*10^{-10} einstein s^{-1} (determined by actinometry as mentioned above), u is the reaction time (3 h) and f_R is the fraction of incident light absorbed by the reaction mixture, determined using equation 3. An absorption spectrum of the reaction mixture gave an absorbance value of 2.85 at 510 nm leading to a f_R value of 0.89 indicating that essentially almost all incident light is absorbed by the photocatalyst.

$$\emptyset = \frac{n(product)}{\emptyset_q \cdot t \cdot f_R} \qquad (4)$$

Thus, the reaction quantum yield (Φ) was determined to be ϕ = 0.61.

7.5. EPR experiment

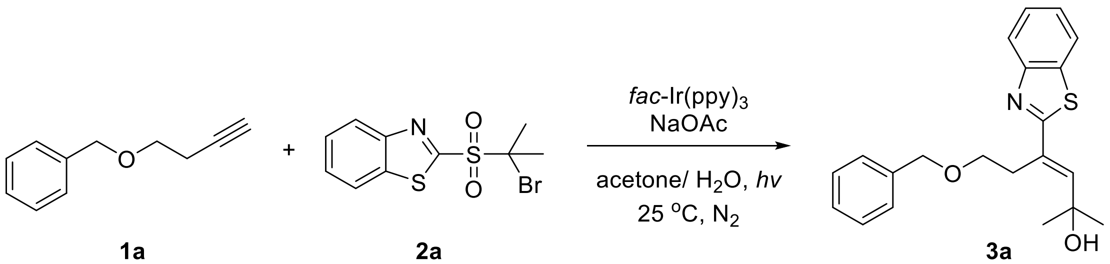

1a **2a** **3a**

Experiment A: A flame-dried sealed tube was charged with **1a** (0.4 mmol, 2.0 equiv.), **2a** (0.2 mmol, 1.0 equiv.) and NaOAc (0.1 mmol, 0.5 equiv.), which was subjected to evacuation/ flushing with N_2 for 3 times. Then acetone (2 mL) and H_2O (0.6 mL) were added to the mixture via syringe under N_2. The sealed tube was placed 5 cm away from the light source and stirred at 25 °C. The reaction was irradiated with 30 W 510 nm green LED for 1.5 h for signal detection.

Experiment B: A flame-dried sealed tube was charged with **1a** (0.4 mmol, 2.0 equiv.), **2a** (0.2 mmol, 1.0 equiv.) and NaOAc (0.1 mmol, 0.5 equiv.), which was subjected to evacuation/ flushing with N_2 for 3 times. Then acetone (2 mL) and H_2O (0.6 mL) were added to the mixture via syringe under N_2. The sealed tube was placed 5 cm away from the light source and stirred at 25 °C. The reaction was irradiated with 30 W 510 nm green LED for 1.0 h. Subsequently, DMPO (2.0 equiv.) was added to the reaction and reacted for another 0.5 h for signal detection.

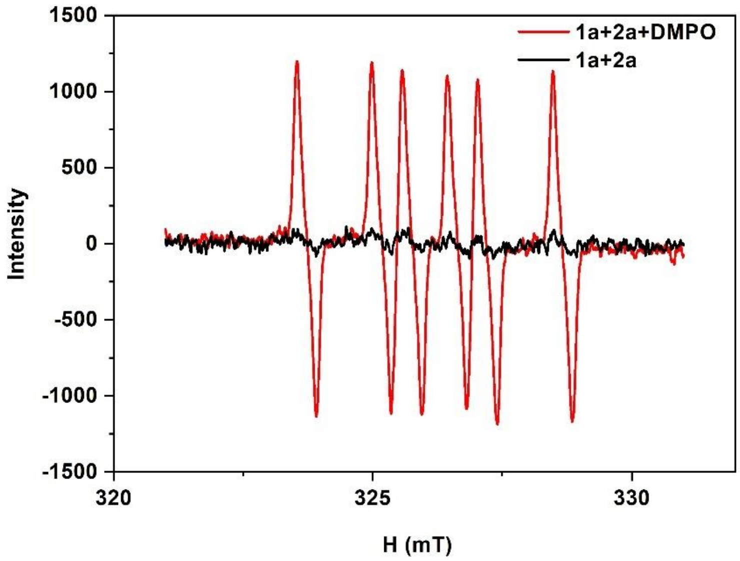

Figure S13. EPR experiment

7.6 Cyclic voltammograms of 2a

All voltammograms were taken at room temperature using a saturated calomel (SCE) reference electrode, a mesh platinum (Pt) counter electrode, and a glassy carbon working electrode. The conditions of the experiments were the following: an acetonitrile solution of 100 mM tetrabutylammonium hexafluorophosphate (NBu_4PF_6) and 3 mM **2a**, a scan rate of 0.1 V/s, and a negative initial scan direction. The reported potentials were averages over segments, and were taken at half-height of the cathodic peaks ($E_{p/2}$) of **2a**, since all reductions were nonreversible. To convert the potentials from SCE to Fc/Fc+ reference, 380 mV were subtracted from the measured values. The positive peaks on the return sweep of most substrates were thought to signify an ECE-type mechanism.

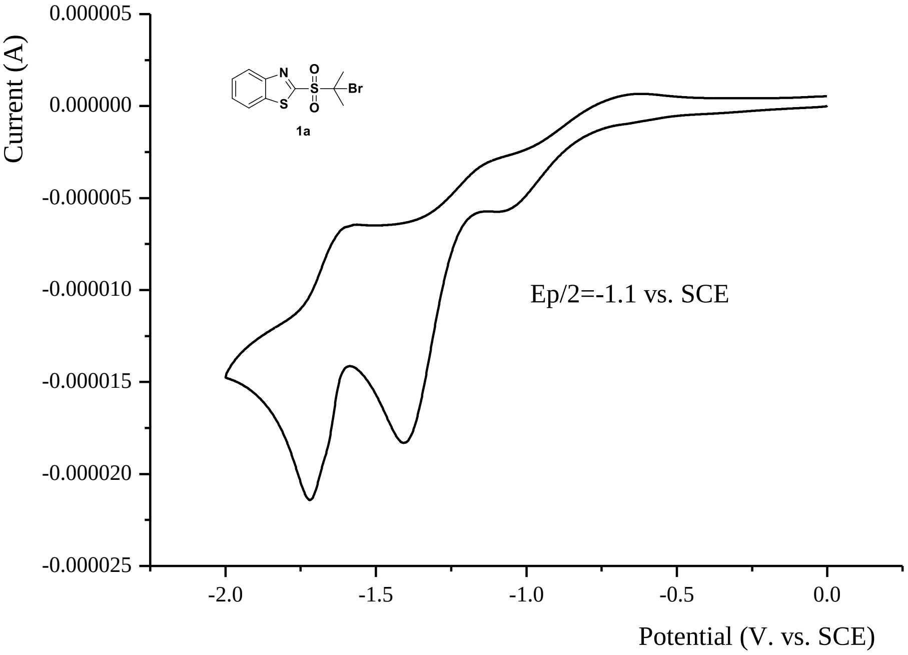

Fig. Difunctionalization reagent 2a

8. DFT calculations

Computational methods

All theoretical calculations were performed with Gaussian 09[16]. Geometry optimizations and frequency computations were calculated by M06[17-19] functional together with the 6-31G(d) basis set. Single-point energies were calculated the SMD-M06/6-311++G(2d,p) method. The solvent effect of acetone was estimated with the SMD model.[20] All the optimized geometries were verified as minima or transition state structures by frequency calculations.

8.1. BDE calculations

Cartesian Coordinates and Energies

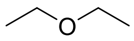

Center Number	Atomic Number	Atomic Type	Coordinates (Angstroms) X	Y	Z
1	6	0	1.182742	-0.521931	0.000118
2	1	0	1.206297	-1.179205	-0.886844
3	1	0	1.206254	-1.178872	0.887329
4	6	0	-1.182742	-0.521931	-0.000112

5	1	0	-1.206230	-1.179152	0.886892
6	1	0	-1.206321	-1.178925	-0.887281
7	6	0	-2.378798	0.416454	0.000072
8	1	0	-2.362376	1.058324	-0.886715
9	1	0	-3.314339	-0.153773	0.000074
10	1	0	-2.362258	1.058133	0.886997
11	6	0	2.378798	0.416454	-0.000032
12	1	0	2.362305	1.058382	0.886713
13	1	0	3.314339	-0.153773	0.000079
14	1	0	2.362328	1.058075	-0.886999
15	8	0	0.000000	0.257064	-0.000066

Zero-point correction= 0.137473 (Hartree/Particle)
Thermal correction to Energy= 0.144273
Thermal correction to Enthalpy= 0.145217
Thermal correction to Gibbs Free Energy= 0.107355
Sum of electronic and zero-point Energies= -233.525976
Sum of electronic and thermal Energies= -233.519176
Sum of electronic and thermal Enthalpies= -233.518232
Sum of electronic and thermal Free Energies= -233.556094
B3LYP/6-311++G(d,p)/SMD// B3LYP/6-31G(d) energy= -233.742882

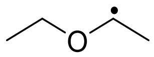

Center Number	Atomic Number	Atomic Type	Coordinates (Angstroms) X	Y	Z
1	6	0	-1.192164	0.486238	-0.058788
2	1	0	-1.150464	1.473987	0.406874
3	6	0	1.168698	0.537041	-0.045641
4	1	0	1.163414	1.237651	0.804256
5	1	0	1.208590	1.133965	-0.968096
6	6	0	2.350738	-0.412473	0.042368
7	1	0	2.351720	-1.105193	-0.805245
8	1	0	3.290719	0.150089	0.032070
9	1	0	2.306999	-0.999435	0.965462
10	6	0	-2.432248	-0.334795	0.014883
11	1	0	-2.558501	-0.832015	0.992981
12	1	0	-3.313426	0.293464	-0.153262
13	1	0	-2.422868	-1.125119	-0.746056
14	8	0	-0.030791	-0.235433	-0.030740

Zero-point correction= 0.123367 (Hartree/Particle)
Thermal correction to Energy= 0.130402
Thermal correction to Enthalpy= 0.131347
Thermal correction to Gibbs Free Energy= 0.092259
Sum of electronic and zero-point Energies= -232.880668
Sum of electronic and thermal Energies= -232.873633
Sum of electronic and thermal Enthalpies= -232.872688
Sum of electronic and thermal Free Energies= -232.911776
UB3LYP/6-311++G(d,p)/SMD// UB3LYP/6-31G(d) energy= -233.083370

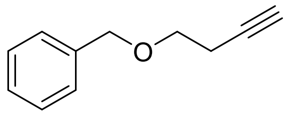

Center Number	Atomic Number	Atomic Type	Coordinates (Angstroms) X	Y	Z
1	6	0	3.367276	-0.645059	-0.528897
2	1	0	4.307784	-1.195352	-0.381994
3	1	0	3.137309	-0.702251	-1.600812
4	6	0	2.255369	-1.400654	0.234982
5	1	0	2.376116	-2.469704	0.029199
6	1	0	2.371698	-1.243258	1.318564
7	8	0	0.943761	-1.085160	-0.183075
8	6	0	0.390273	0.109006	0.359692
9	1	0	0.618055	0.156239	1.439262
10	1	0	0.844123	0.997677	-0.097812
11	6	0	-1.106465	0.117594	0.144151
12	6	0	-1.848490	-1.068794	0.181673
13	6	0	-1.777071	1.329809	-0.052788
14	6	0	-3.234667	-1.040420	0.028393
15	1	0	-1.328395	-2.011779	0.315786
16	6	0	-3.165026	1.361177	-0.196646
17	1	0	-1.208695	2.256677	-0.096907
18	6	0	-3.898343	0.174383	-0.157236
19	1	0	-3.798529	-1.969556	0.053663
20	1	0	-3.670966	2.310987	-0.349698
21	1	0	-4.978506	0.195004	-0.276013
22	6	0	3.569243	0.748358	-0.129753
23	6	0	3.757967	1.890561	0.215663
24	1	0	3.919516	2.900829	0.515962

Zero-point correction= 0.199850 (Hartree/Particle)
Thermal correction to Energy= 0.211455
Thermal correction to Enthalpy= 0.212399
Thermal correction to Gibbs Free Energy= 0.160038
Sum of electronic and zero-point Energies= -501.330571
Sum of electronic and thermal Energies= -501.318966
Sum of electronic and thermal Enthalpies= -501.318022
Sum of electronic and thermal Free Energies= -501.370382

B3LYP/6-311++G(d,p)/SMD// B3LYP/6-31G(d) energy= -501.688123

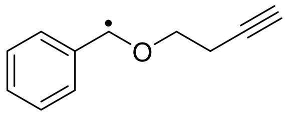

Center Number	Atomic Number	Atomic Type	Coordinates (Angstroms) X	Y	Z
1	6	0	3.321677	-0.531378	-0.442927
2	1	0	4.374337	-0.781569	-0.248462
3	1	0	3.055268	-1.013779	-1.392144
4	6	0	2.475875	-1.171302	0.674369
5	1	0	2.773669	-2.217726	0.787060
6	1	0	2.642860	-0.655917	1.629087
7	8	0	1.088961	-1.202559	0.365508
8	6	0	0.349942	-0.108699	0.697546
9	1	0	0.843383	0.668635	1.272610
10	6	0	-1.003778	-0.035046	0.319077
11	6	0	-1.640189	-1.056476	-0.443479
12	6	0	-1.785085	1.093310	0.701505
13	6	0	-2.977240	-0.943397	-0.794631
14	1	0	-1.062287	-1.923591	-0.745430
15	6	0	-3.119447	1.189187	0.340847
16	1	0	-1.319535	1.885538	1.283679
17	6	0	-3.731221	0.174074	-0.409821
18	1	0	-3.444160	-1.734250	-1.377021
19	1	0	-3.695055	2.060300	0.644244
20	1	0	-4.777726	0.253576	-0.689772
21	6	0	3.180101	0.919624	-0.558802
22	6	0	3.081913	2.120229	-0.643535
23	1	0	2.982263	3.178496	-0.728809

Zero-point correction= 0.186290 (Hartree/Particle)
Thermal correction to Energy= 0.197836
Thermal correction to Enthalpy= 0.198780
Thermal correction to Gibbs Free Energy= 0.146551
Sum of electronic and zero-point Energies= -500.706770
Sum of electronic and thermal Energies= -500.695224
Sum of electronic and thermal Enthalpies= -500.694280
Sum of electronic and thermal Free Energies= -500.746510
UB3LYP/6-311++G(d,p)/SMD// UB3LYP/6-31G(d) energy= -501.051197

8.2. Energy calculations

Cartesian Coordinates and Energies

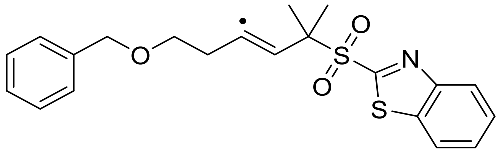

Center Number	Atomic Number	Atomic Type	Coordinates (Angstroms) X	Y	Z
1	6	0	5.751207	2.136471	-1.080931
2	6	0	4.407472	2.078807	-0.740840
3	6	0	3.811997	0.821048	-0.556974
4	6	0	4.581831	-0.358986	-0.718955
5	6	0	5.936146	-0.296047	-1.063622
6	6	0	6.507599	0.959559	-1.240460
7	1	0	6.228206	3.100849	-1.228676
8	1	0	3.808160	2.975147	-0.618015
9	1	0	6.526186	-1.198420	-1.189905
10	1	0	7.557977	1.031946	-1.507707
11	6	0	2.241092	-0.651608	-0.127810
12	6	0	0.104430	-0.687903	1.933672
13	7	0	2.483012	0.610332	-0.222635
14	16	0	3.580645	-1.766651	-0.424183
15	16	0	0.602595	-1.356526	0.231828
16	8	0	0.860381	-2.805046	0.354931
17	8	0	-0.340217	-0.855128	-0.781245
18	6	0	-1.221034	-1.406699	2.230076
19	1	0	-1.562203	-1.099897	3.223936
20	1	0	-1.994696	-1.138444	1.506676
21	1	0	-1.085269	-2.491573	2.223192

22	6	0	1.200526	-1.098769	2.925319
23	1	0	2.150112	-0.594739	2.716865
24	1	0	0.883346	-0.808321	3.932992
25	1	0	1.362881	-2.179872	2.909143
26	6	0	-0.037122	0.813652	1.848460
27	1	0	0.901452	1.379059	1.888310
28	6	0	-1.179178	1.459406	1.723901
29	6	0	-1.716579	2.826461	1.642457
30	1	0	-0.913786	3.556451	1.857540
31	1	0	-2.484746	2.965534	2.415397
32	6	0	-2.347655	3.190303	0.278897
33	1	0	-2.709620	4.224208	0.337611
34	1	0	-1.585482	3.144358	-0.511515
35	8	0	-3.456641	2.372440	-0.033286
36	6	0	-3.152172	1.296642	-0.922216
37	1	0	-2.958200	1.708349	-1.928352
38	1	0	-2.239767	0.774678	-0.609938
39	6	0	-4.307090	0.324524	-0.973105
40	6	0	-5.626889	0.750071	-0.782505
41	6	0	-4.059363	-1.025073	-1.254768
42	6	0	-6.681159	-0.159449	-0.874323
43	1	0	-5.818375	1.792612	-0.549435
44	6	0	-5.115593	-1.932356	-1.354662
45	1	0	-3.034580	-1.363284	-1.391871
46	6	0	-6.429958	-1.502311	-1.164125
47	1	0	-7.701879	0.181634	-0.719202
48	1	0	-4.909246	-2.977158	-1.572747
49	1	0	-7.252624	-2.209183	-1.236308

Zero-point correction= 0.388300 (Hartree/Particle)

Thermal correction to Energy= 0.414580

Thermal correction to Enthalpy= 0.415524

Thermal correction to Gibbs Free Energy= 0.326697

Sum of electronic and zero-point Energies= -1889.731082

Sum of electronic and thermal Energies= -1889.704802

Sum of electronic and thermal Enthalpies= -1889.703858

Sum of electronic and thermal Free Energies= -1889.792685

UM06/6-311++G(d,p)/SMD// UB3LYP/6-31G(d) energy= -1889.653656

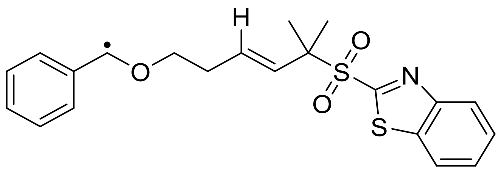

Center Number	Atomic Number	Atomic Type	Coordinates (Angstroms) X	Y	Z
1	6	0	5.438087	2.518257	-0.795984
2	6	0	4.107622	2.300234	-0.469334
3	6	0	3.621544	0.983318	-0.465096
4	6	0	4.485834	-0.092947	-0.790773
5	6	0	5.826407	0.131796	-1.121419
6	6	0	6.288715	1.443464	-1.118657
7	1	0	5.831054	3.530586	-0.805810
8	1	0	3.436933	3.116853	-0.222371
9	1	0	6.488492	-0.690794	-1.373063
10	1	0	7.326423	1.641150	-1.371618
11	6	0	2.184640	-0.662990	-0.249258
12	6	0	0.114808	-1.232979	1.813670
13	7	0	2.318650	0.616074	-0.165337
14	16	0	3.608499	-1.607168	-0.701253
15	16	0	0.613287	-1.544426	0.004965
16	8	0	0.976991	-2.967627	-0.140778
17	8	0	-0.392363	-0.937878	-0.882315
18	6	0	-1.126851	-2.120726	1.989942
19	1	0	-1.506360	-1.991468	3.008941
20	1	0	-1.921361	-1.857644	1.287050
21	1	0	-0.869415	-3.172657	1.842964
22	6	0	1.272965	-1.717344	2.696987
23	1	0	2.166225	-1.095667	2.575686
24	1	0	0.961319	-1.650220	3.745325
25	1	0	1.532490	-2.756595	2.477105
26	6	0	-0.133336	0.241481	1.977996
27	1	0	0.763233	0.857298	2.029852
28	6	0	-1.331994	0.835425	2.020031
29	6	0	-1.526991	2.325052	2.088588
30	1	0	-0.611397	2.814716	2.445737
31	1	0	-2.330965	2.574490	2.792522
32	6	0	-1.873758	2.947422	0.720222
33	1	0	-2.002724	4.028643	0.828647
34	1	0	-1.061017	2.759704	0.006011
35	8	0	-3.105612	2.463451	0.193089
36	6	0	-3.042858	1.294025	-0.502334
37	1	0	-2.061616	0.945392	-0.810478
38	1	0	-2.238782	0.240216	1.954681
39	6	0	-4.226121	0.599629	-0.823014

40	6	0	-5.516764	1.058143	-0.431913
41	6	0	-4.143711	-0.616916	-1.561921
42	6	0	-6.651966	0.335752	-0.770128
43	1	0	-5.597886	1.982008	0.131520
44	6	0	-5.289643	-1.324707	-1.887750
45	1	0	-3.164806	-0.986285	-1.857921
46	6	0	-6.554714	-0.857988	-1.498664
47	1	0	-7.629111	0.703089	-0.464730
48	1	0	-5.204026	-2.251255	-2.450348
49	1	0	-7.449445	-1.416543	-1.758855

Zero-point correction= 0.389053 (Hartree/Particle)

Thermal correction to Energy= 0.414935

Thermal correction to Enthalpy= 0.415879

Thermal correction to Gibbs Free Energy= 0.328586

Sum of electronic and zero-point Energies= -1889.773571

Sum of electronic and thermal Energies= -1889.747689

Sum of electronic and thermal Enthalpies= -1889.746745

Sum of electronic and thermal Free Energies= -1889.834038

UM06/6-311++G(d,p)/SMD// UB3LYP/6-31G(d) energy= -1889.695032

9. NMR spectra

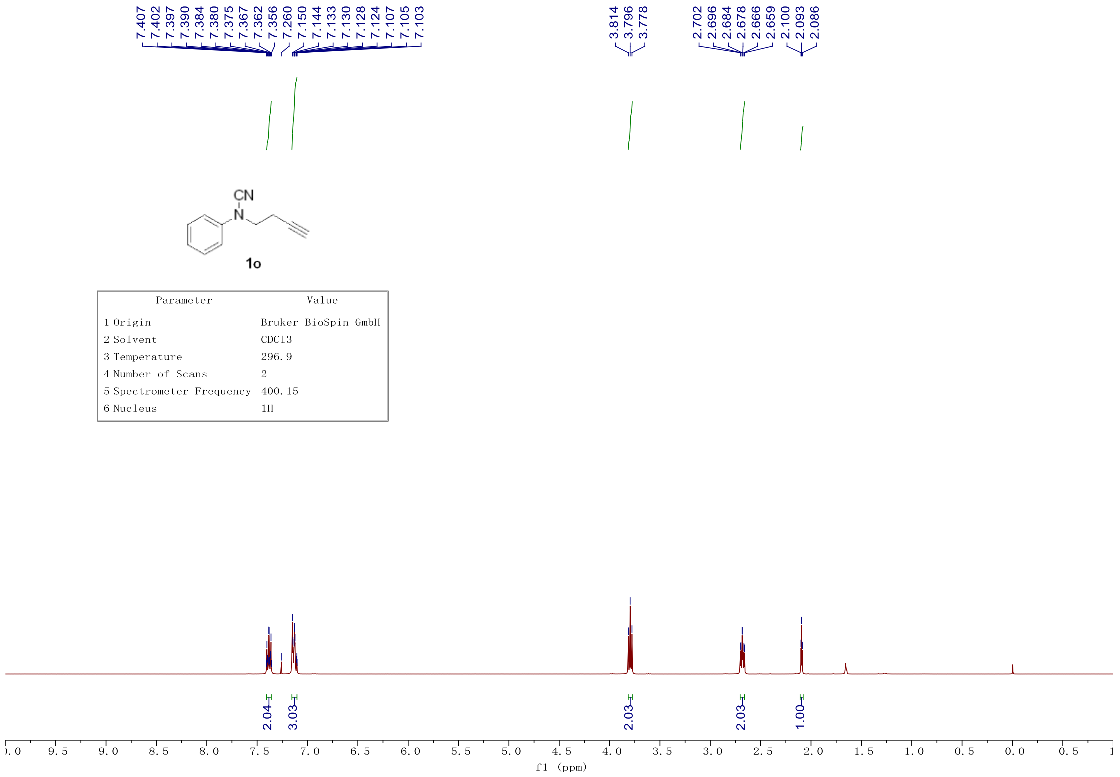

Figure S14. ^{1}H-NMR **of 1o.**

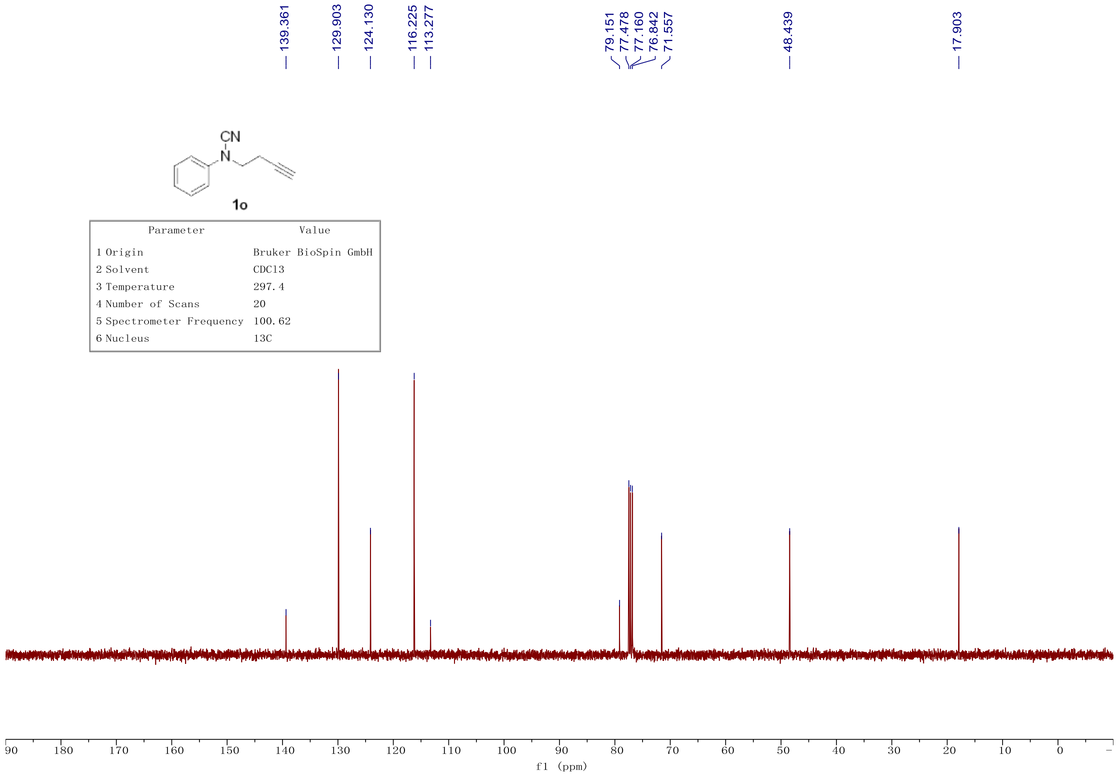

Figure S15. ^{13}C-NMR of **1o.**

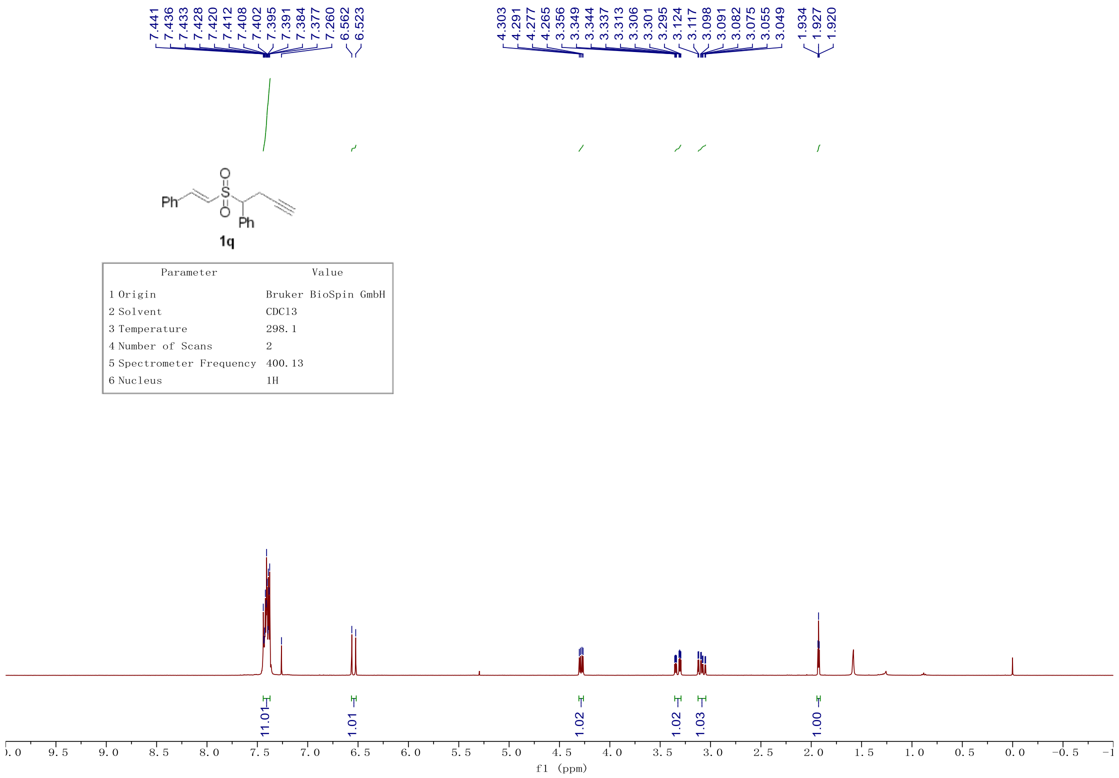

Figure S16. ^{1}H-NMR of **1q.**

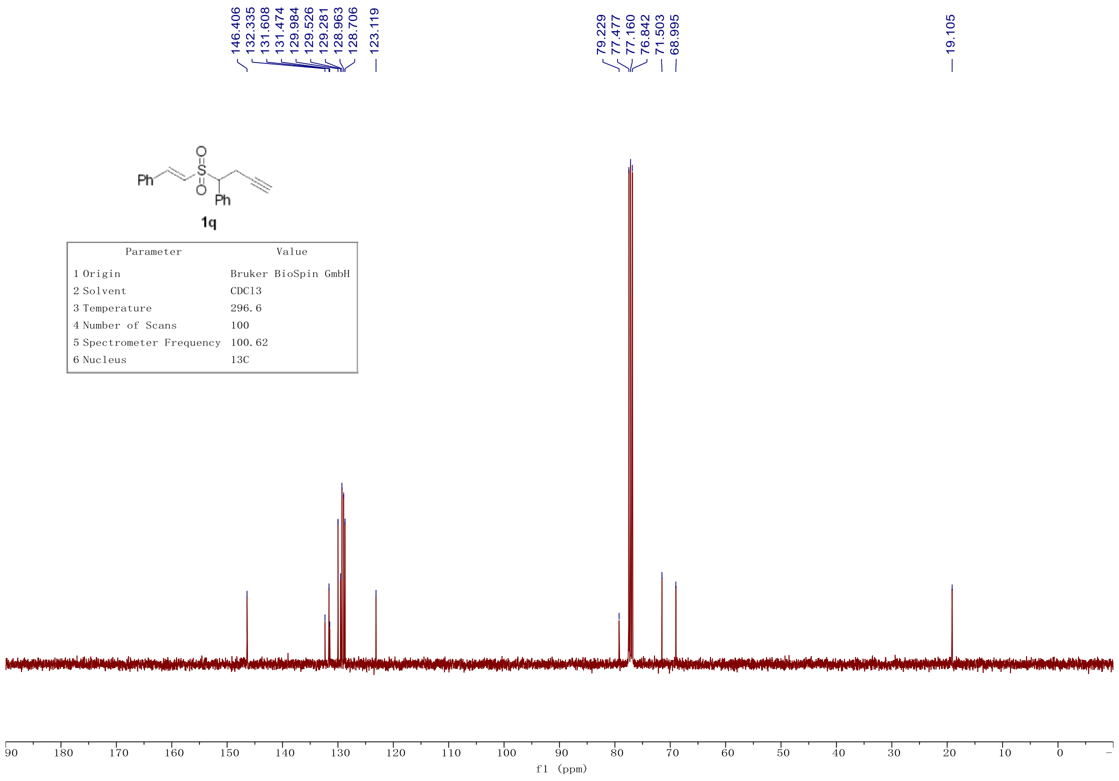

Figure S17. ^{13}C-NMR of **1q.**

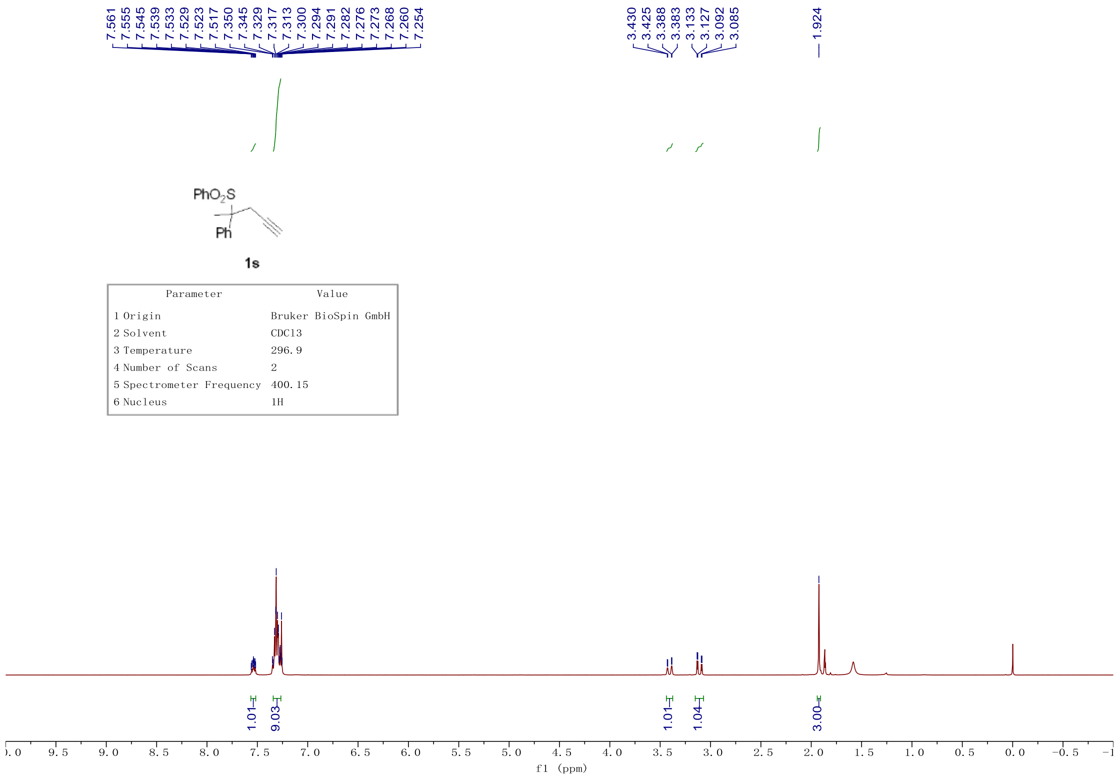

Figure S18. ^{1}H-NMR of **1s.**

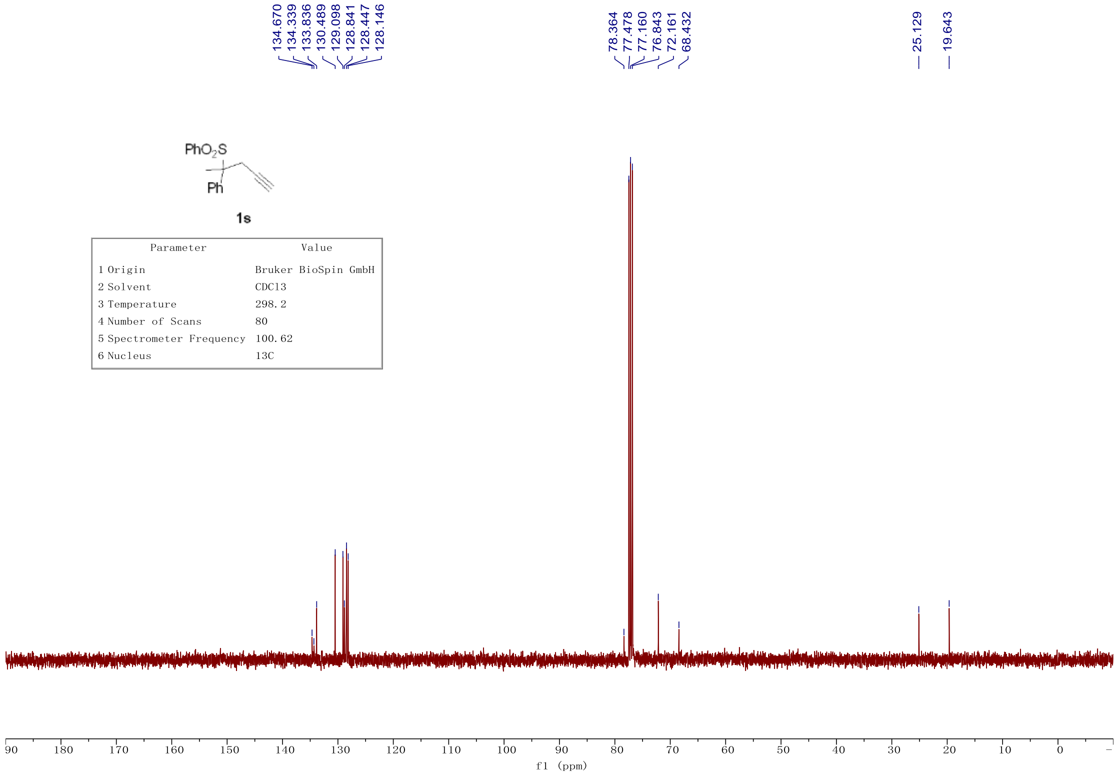

Figure S19. ^{13}C-NMR of **1s.**

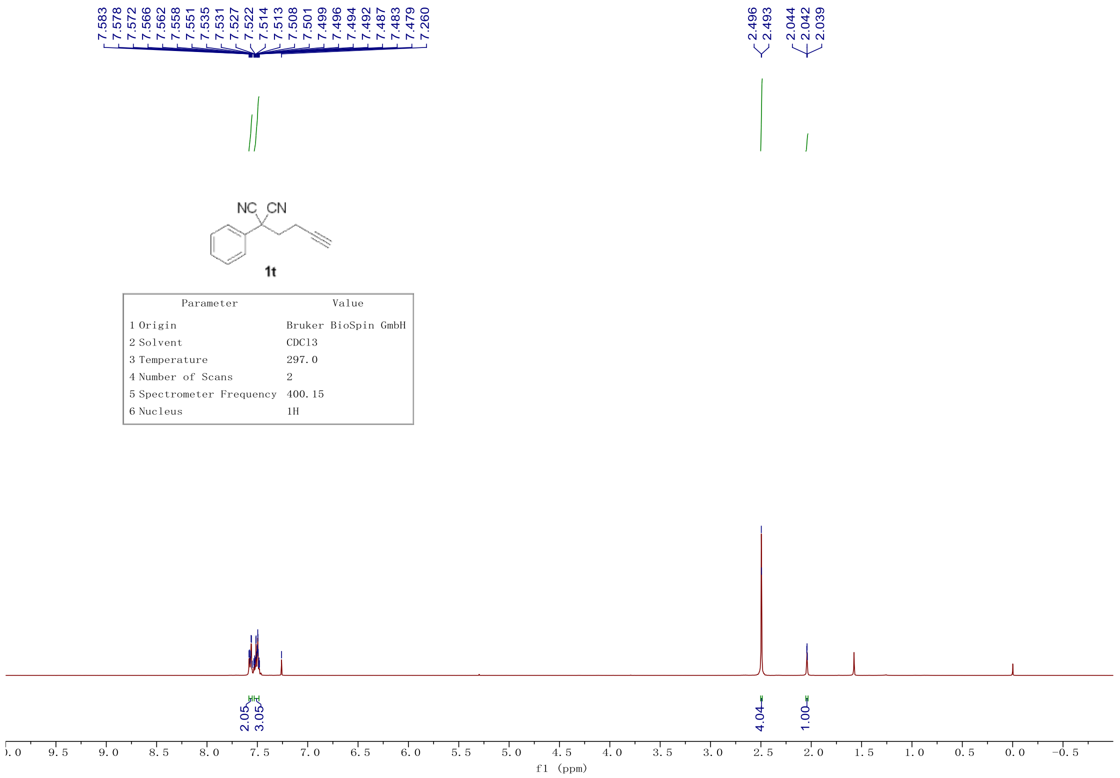

Figure S20. ^{1}H-NMR of **1t.**

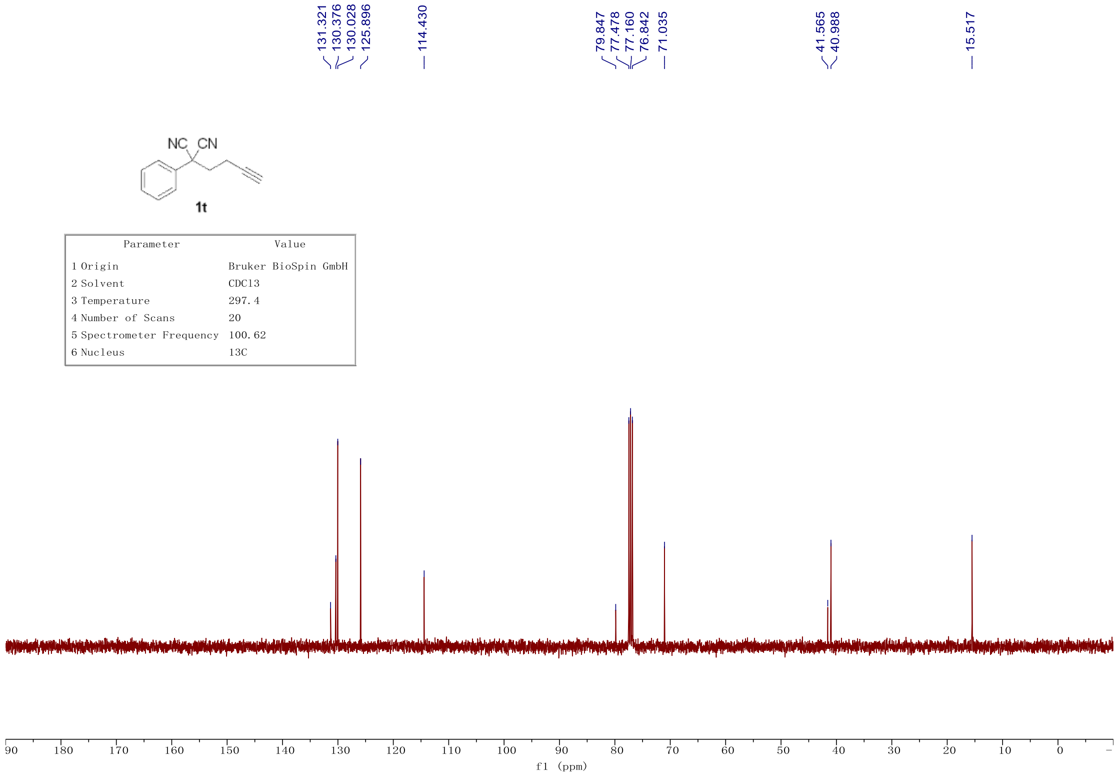

Figure S21. ^{13}C-NMR of **1t.**

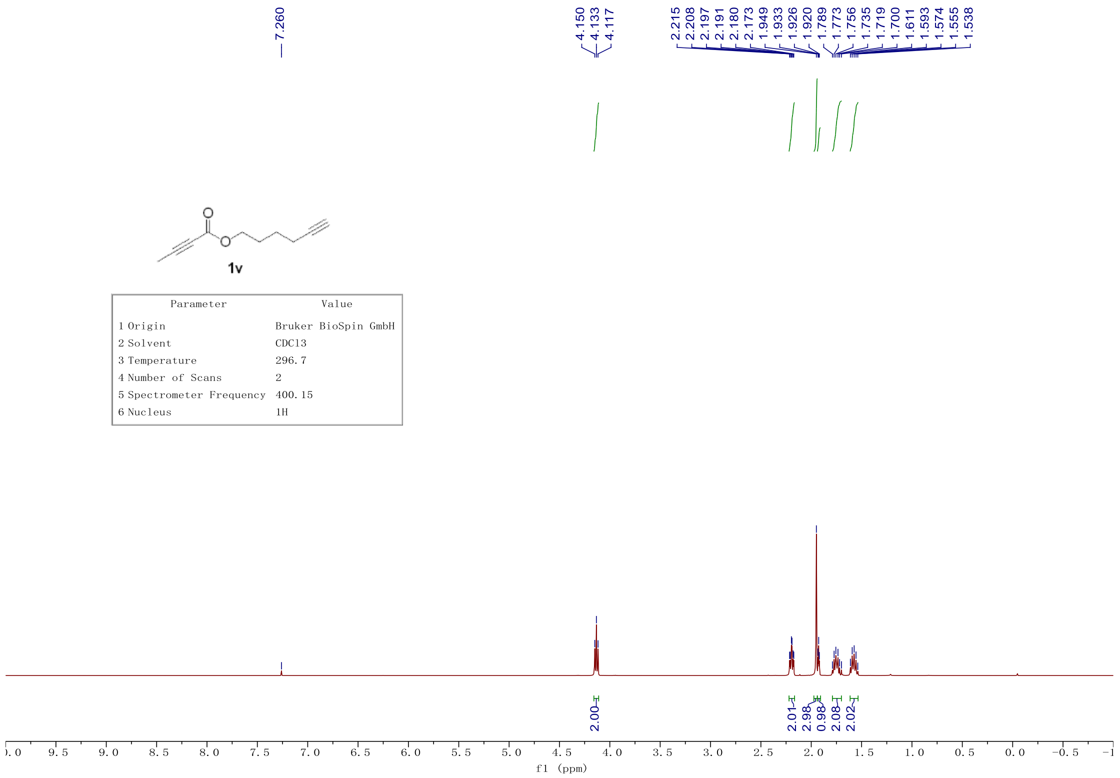

Parameter	Value
1 Origin	Bruker BioSpin GmbH
2 Solvent	CDCl3
3 Temperature	296.7
4 Number of Scans	2
5 Spectrometer Frequency	400.15
6 Nucleus	1H

Figure S22. ^{1}H-NMR of **1v.**

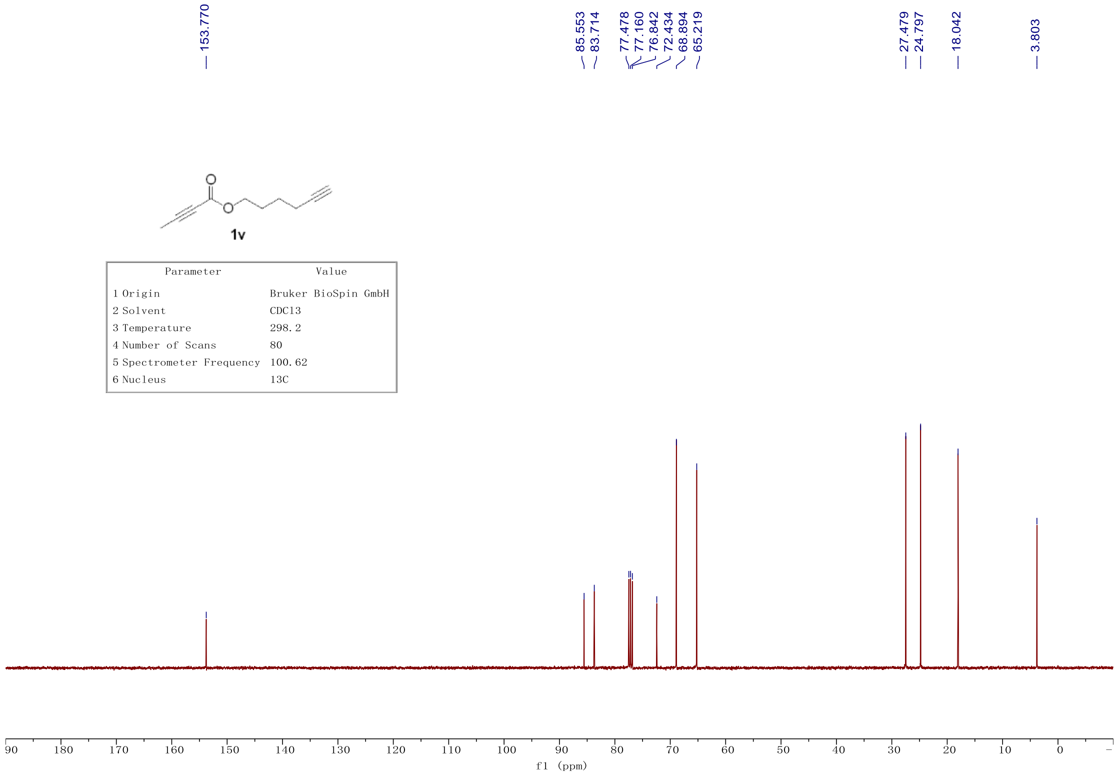

Parameter	Value
1 Origin	Bruker BioSpin GmbH
2 Solvent	CDCl3
3 Temperature	298.2
4 Number of Scans	80
5 Spectrometer Frequency	100.62
6 Nucleus	13C

Figure S23. ^{13}C-NMR of **1v.**

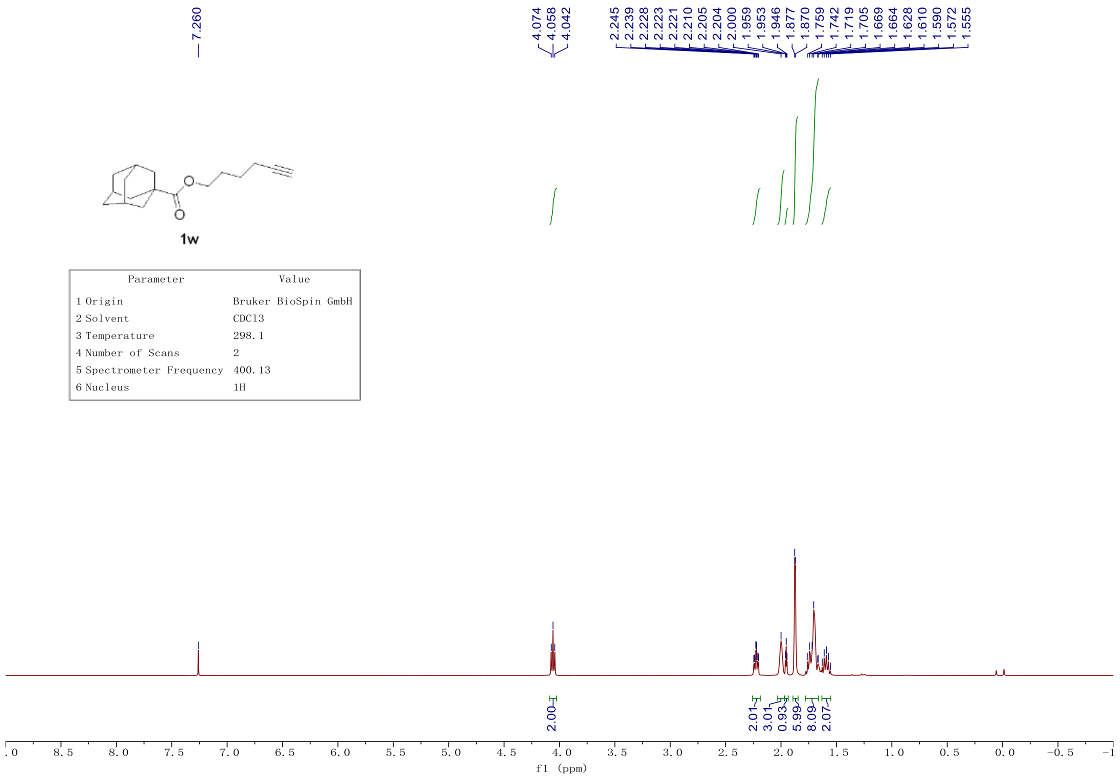

Figure S24. ^{1}H-NMR of **1w.**

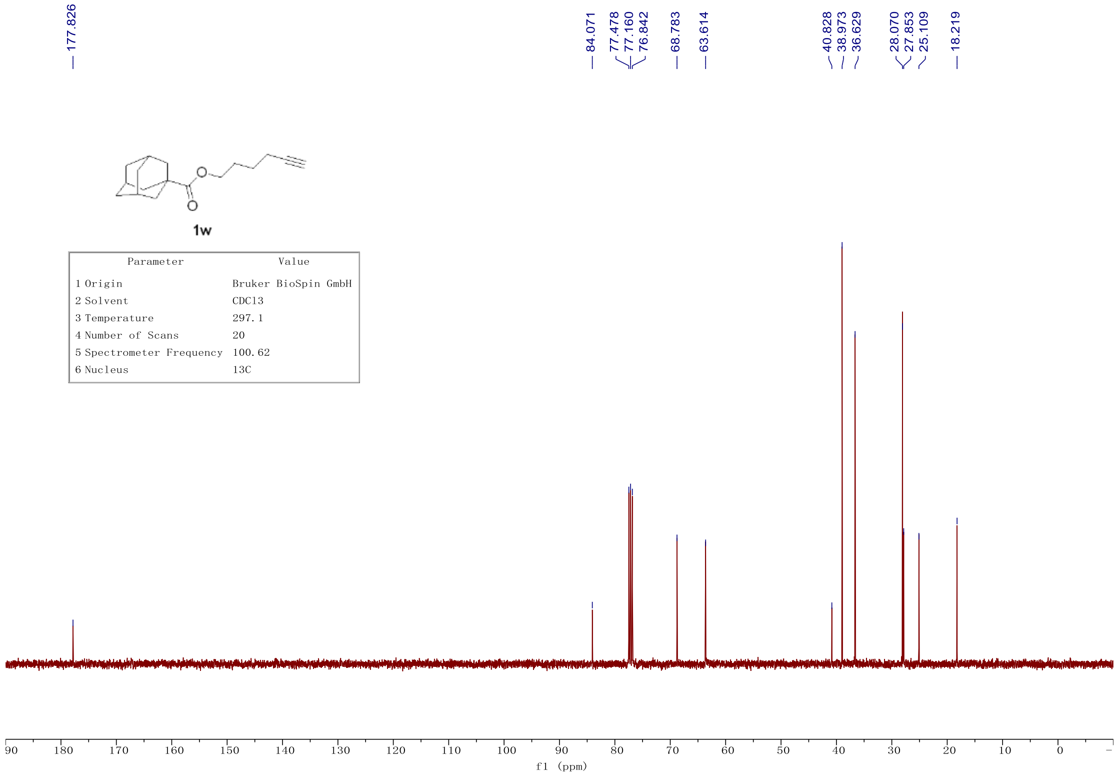

Figure S25. ^{13}C-NMR of **1w.**

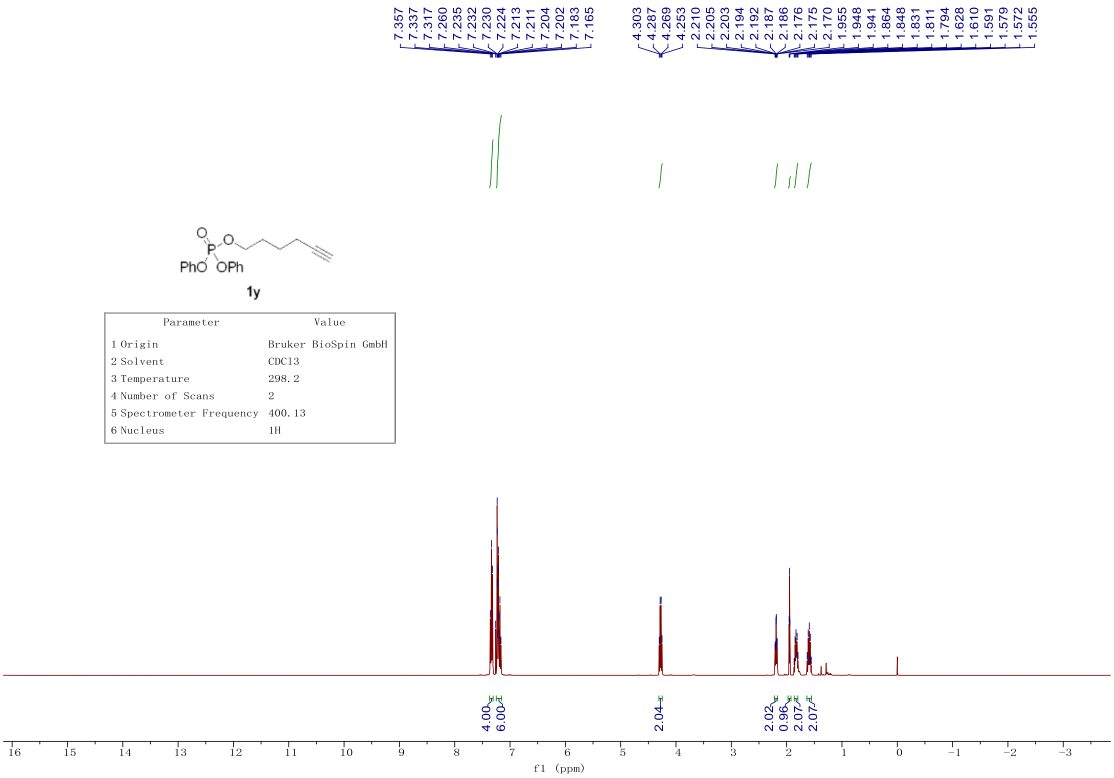

Figure S26. ^{1}H-NMR of **1y.**

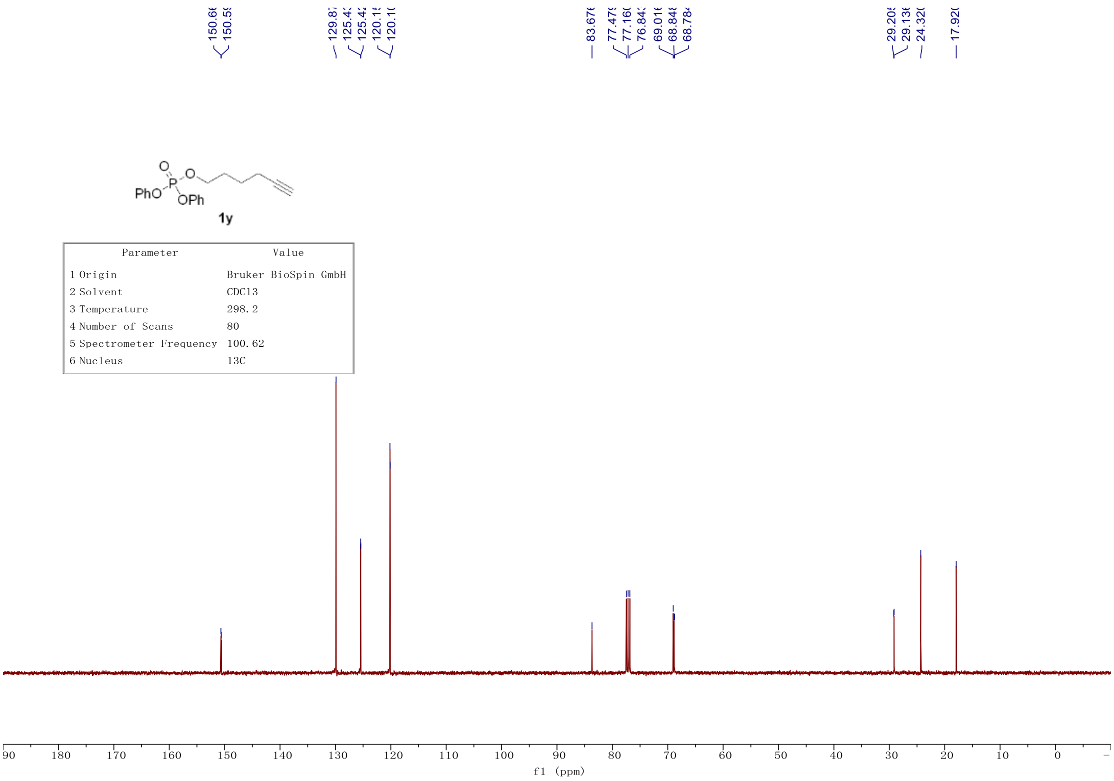

Figure S27. ^{13}C-NMR of **1y.**

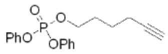

1y

Parameter	值
1 Origin	Bruker BioSpin GmbH
2 Solvent	CDCl3
3 Temperature	294.3
4 Number of Scans	16
5 Spectrometer Frequency	202.50
6 Nucleus	31P

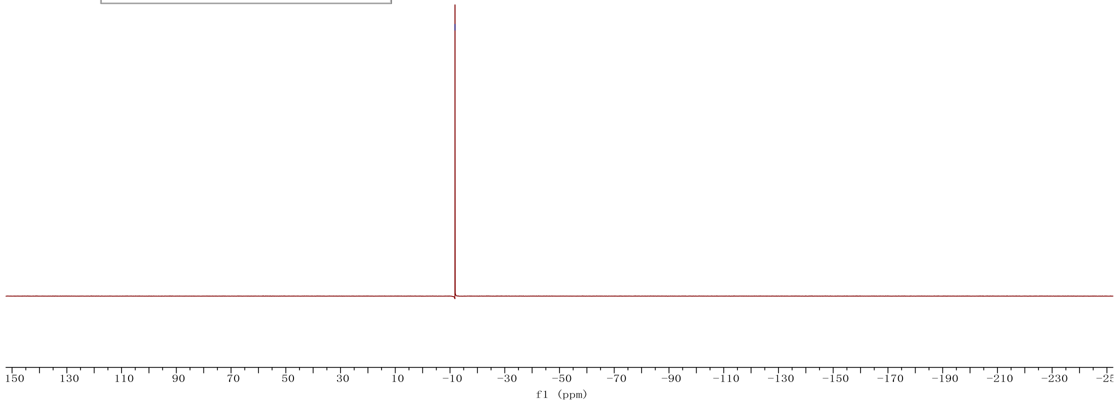

Figure S28. ^{32}P-NMR of **1y.**

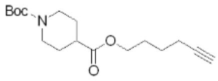

1aa

Parameter	Value
1 Origin	Bruker BioSpin GmbH
2 Solvent	CDCl3
3 Temperature	297.8
4 Number of Scans	2
5 Spectrometer Frequency	400.15
6 Nucleus	1H

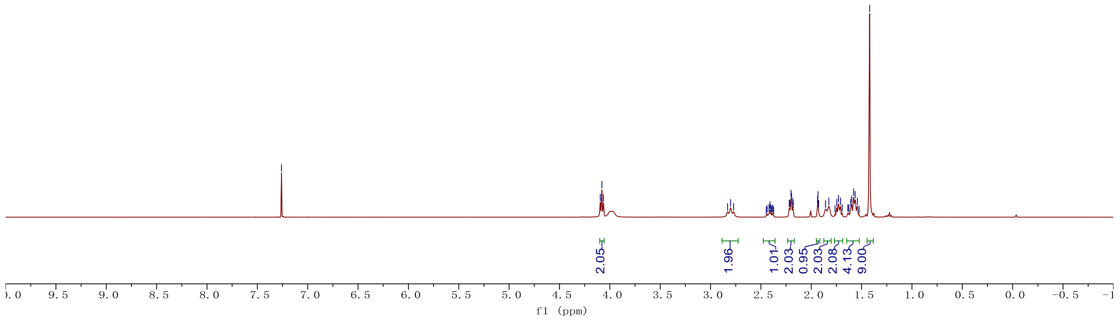

Figure S29. ^{1}H-NMR of **1aa.**

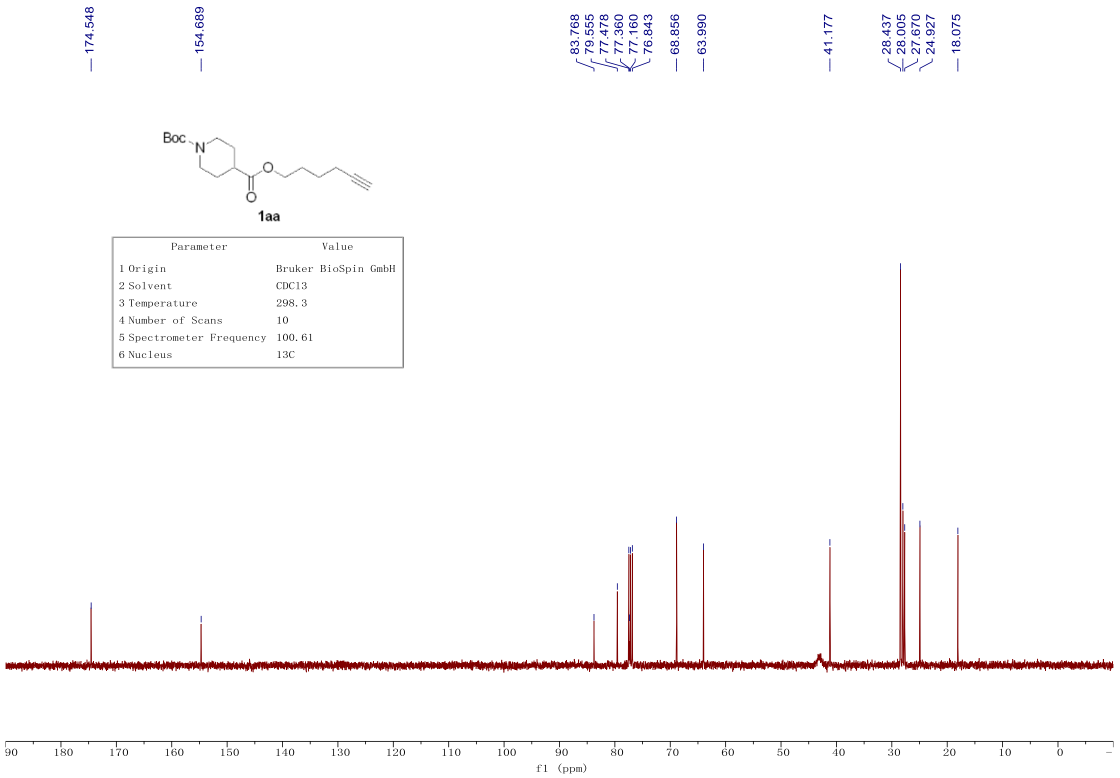

Figure S30. ^{13}C-NMR of **1aa.**

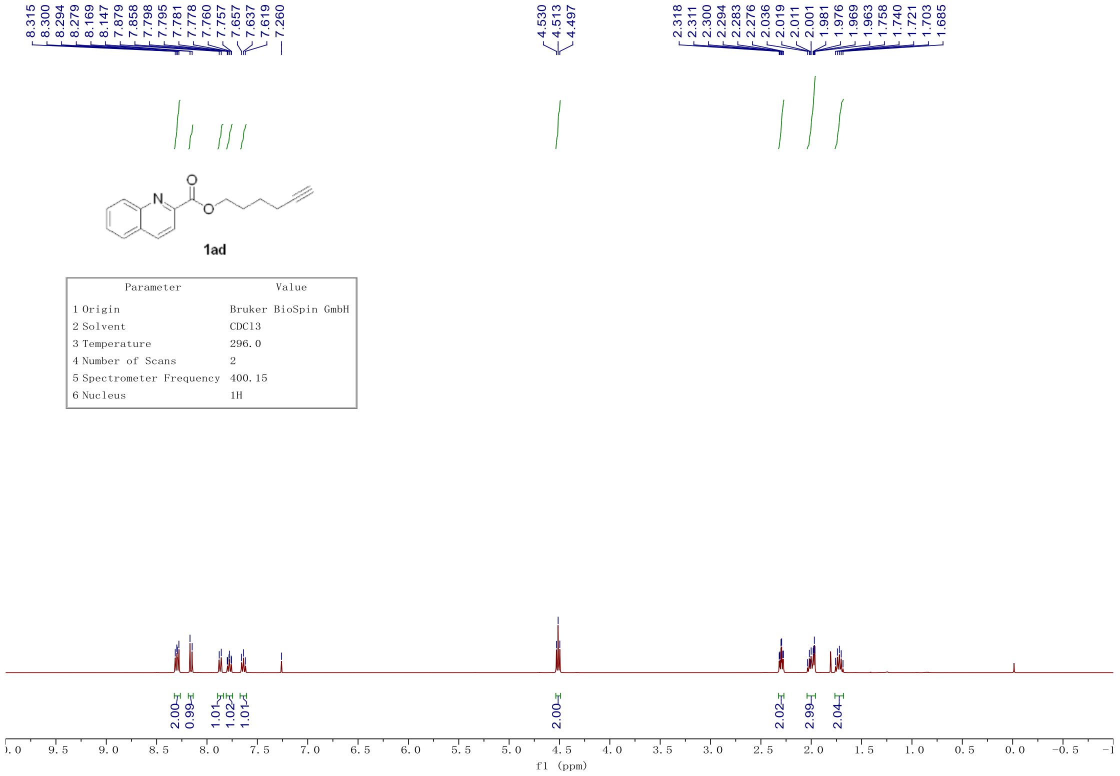

Figure S31. ^{1}H-NMR of **1ad.**

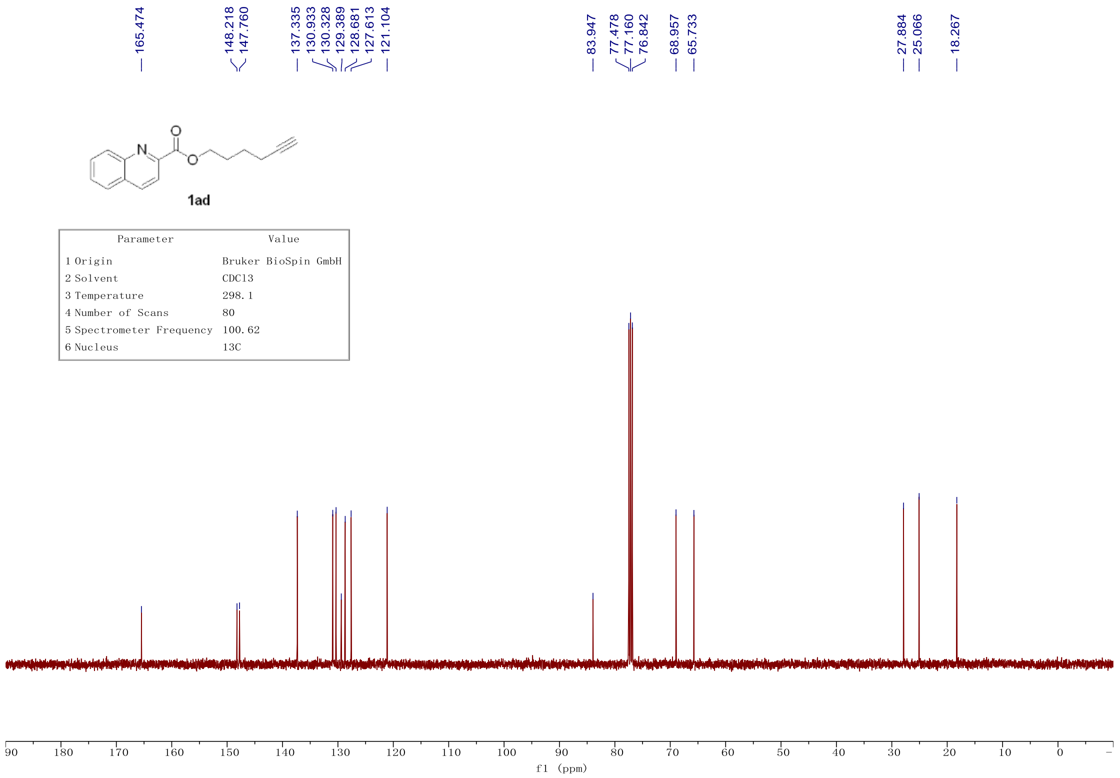

Figure S32. ^{13}C-NMR of **1ad.**

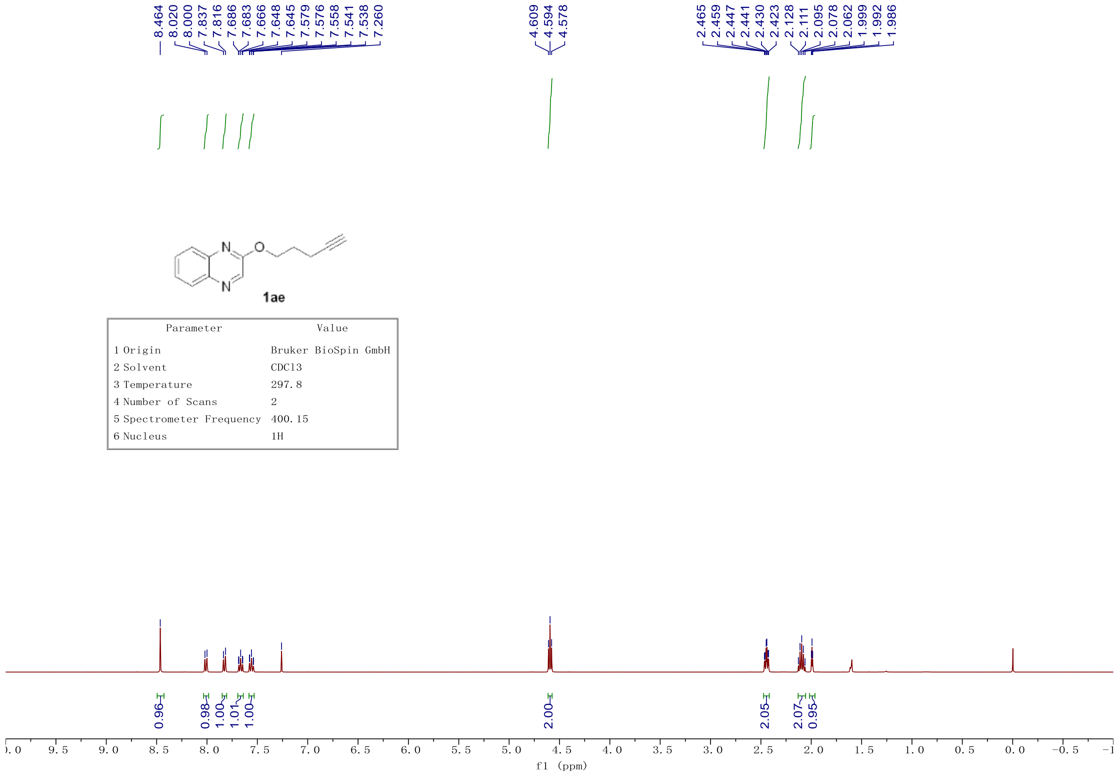

Figure S33. ^{1}H-NMR of **1ae.**

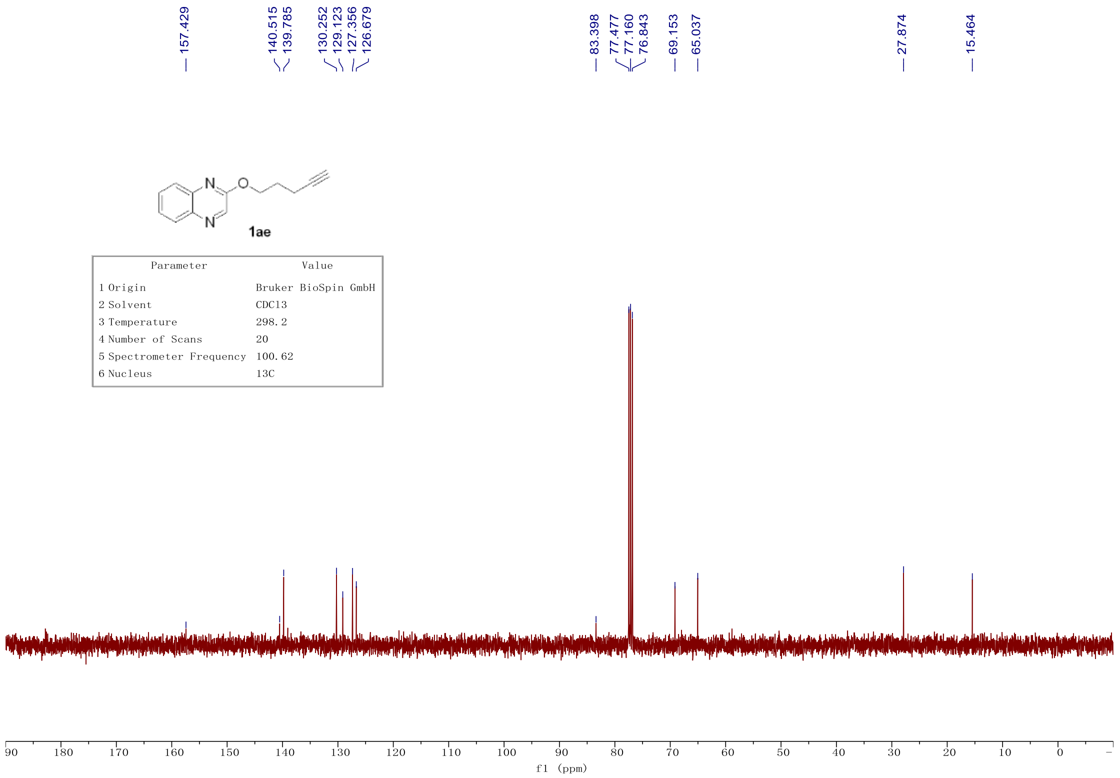

Figure S34. ^{13}C-NMR of **1ae.**

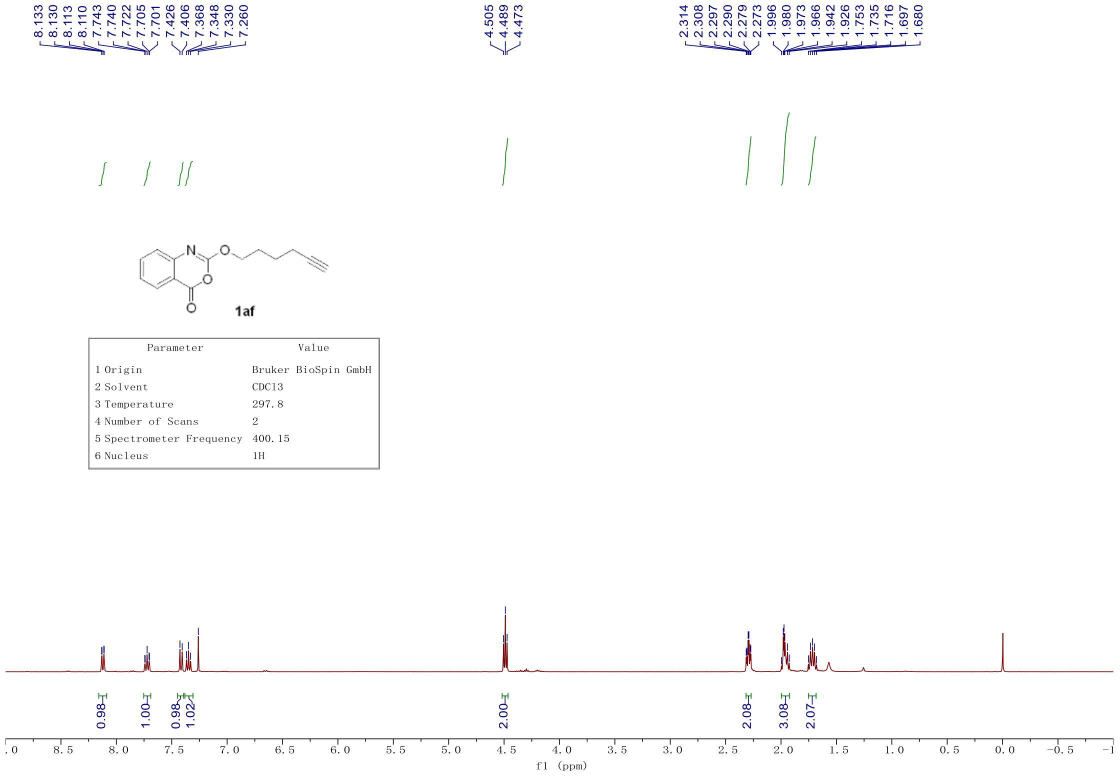

Figure S35. ^{1}H-NMR of **1af.**

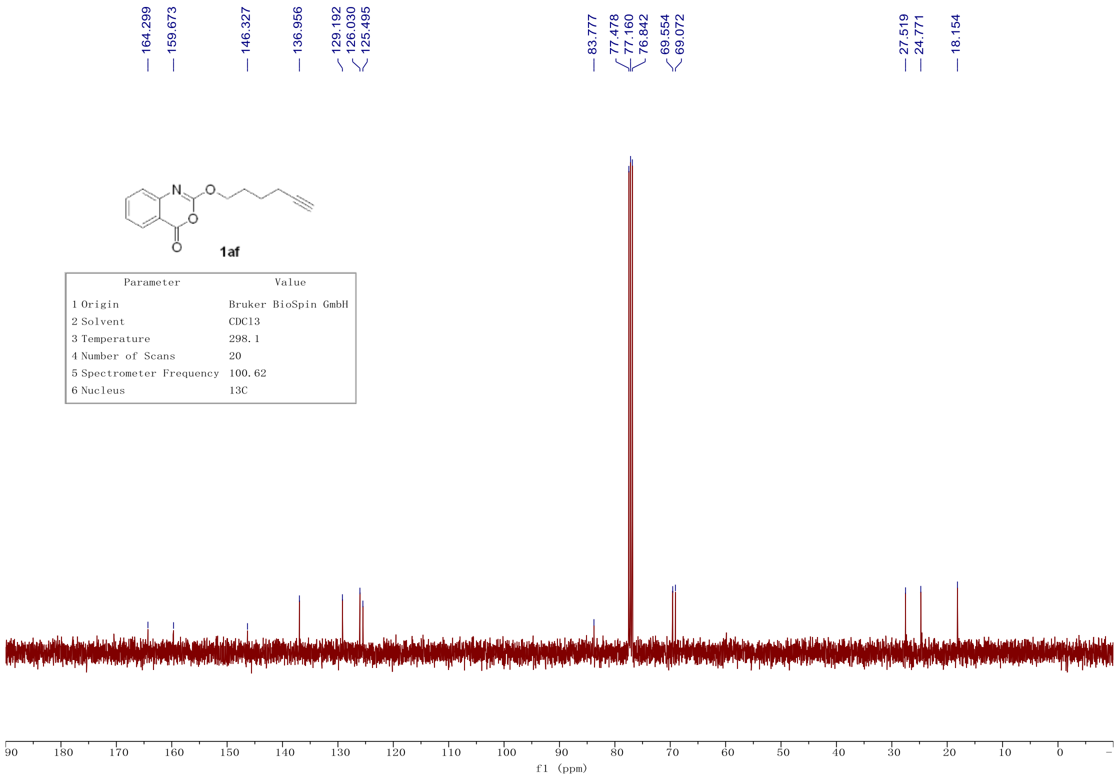

Figure S36. ^{13}C-NMR of **1af.**

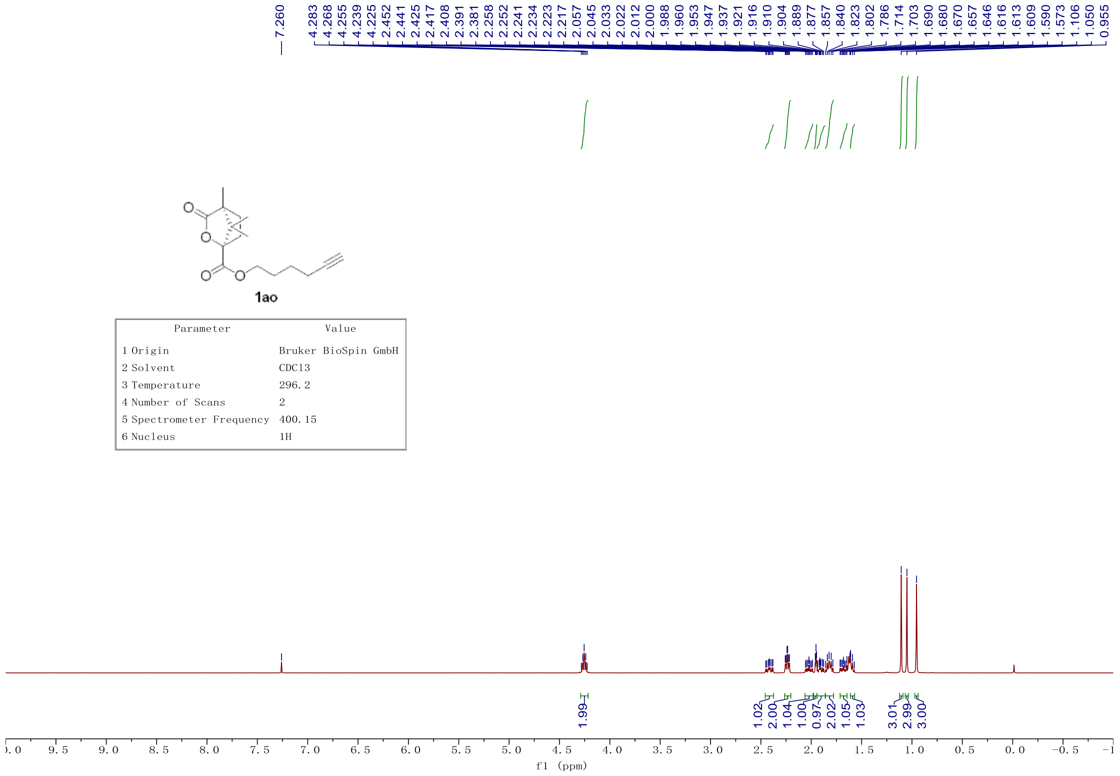

Figure S37. ^{1}H-NMR of **1ao.**

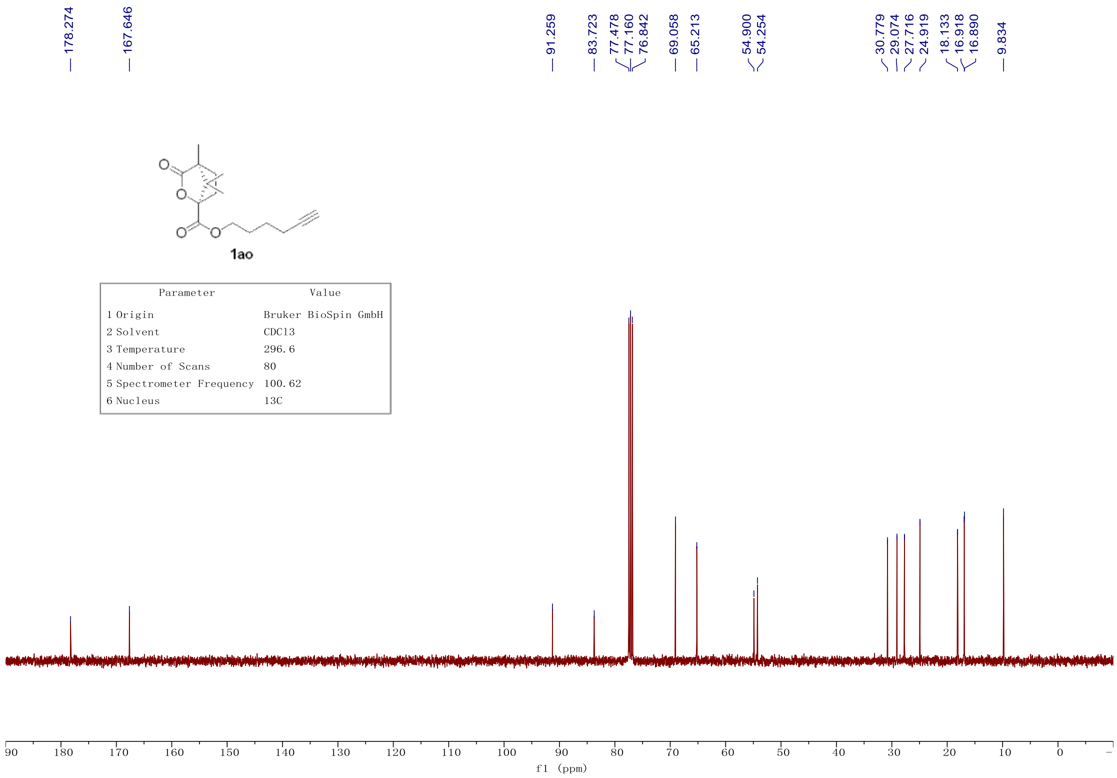

Figure S38. ^{13}C-NMR of **1ao.**

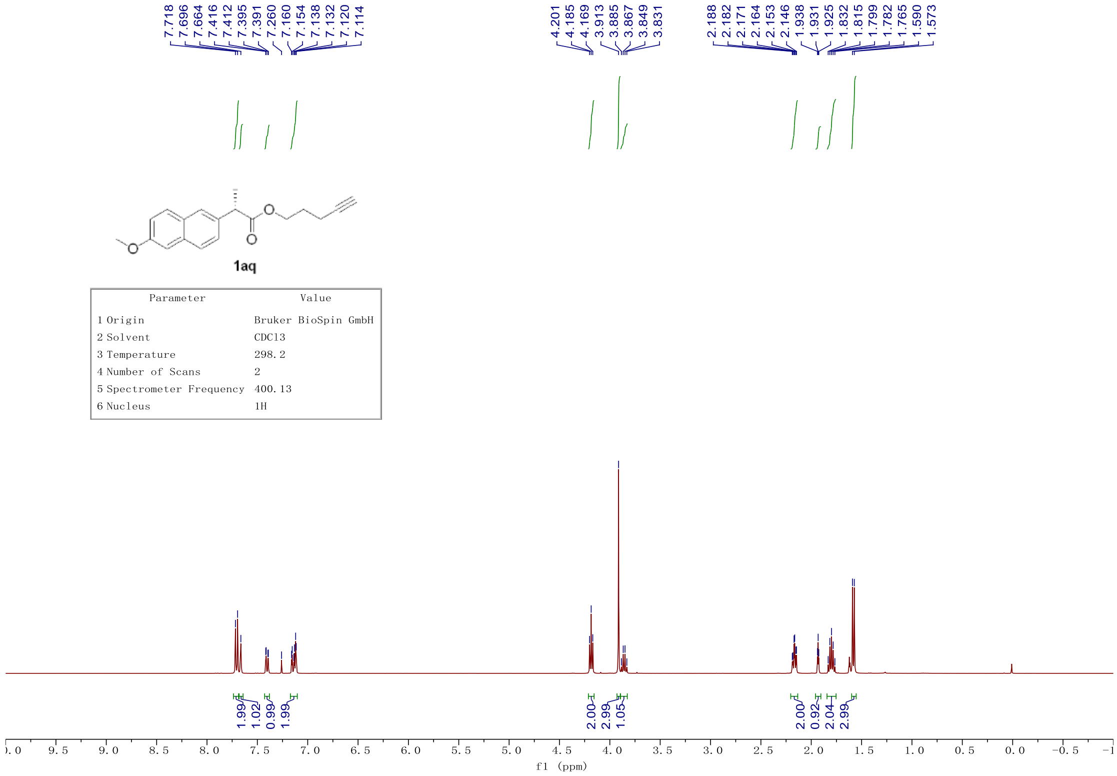

Figure S39. ^{1}H-NMR of **1aq.**

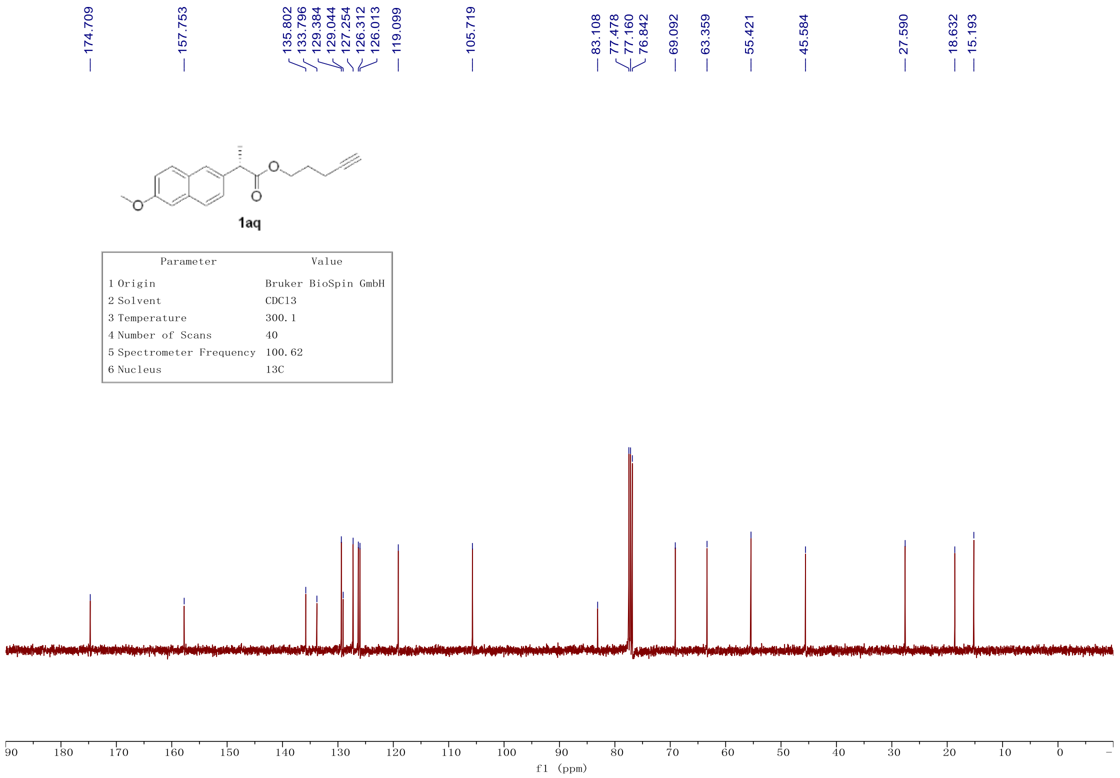

Figure S40. ^{13}C-NMR of **1aq.**

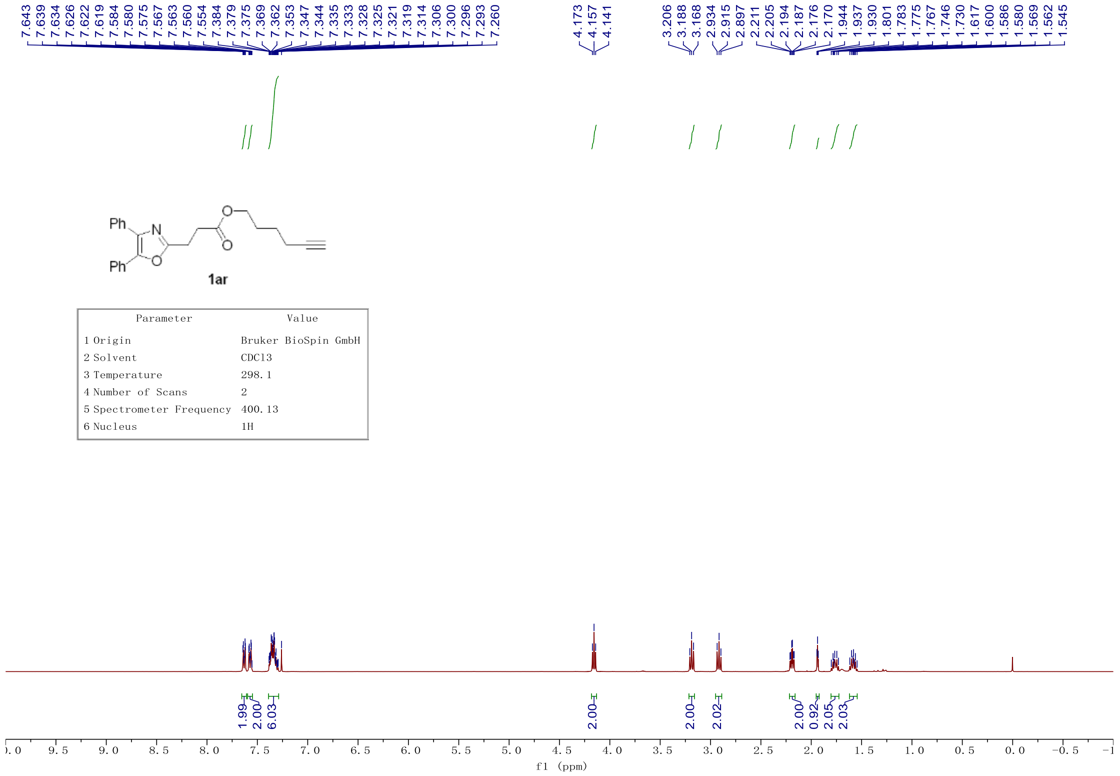

Figure S41. ^{1}H-NMR of **1ar.**

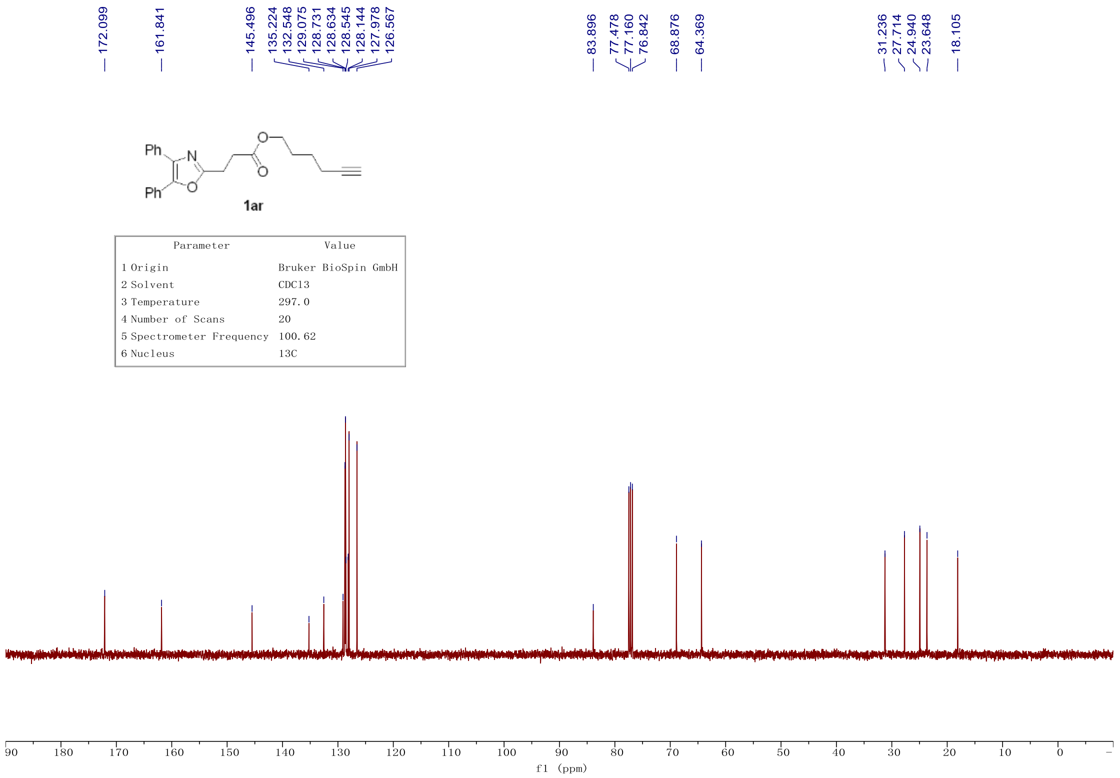

Figure S42. ^{13}C-NMR of **1ar.**

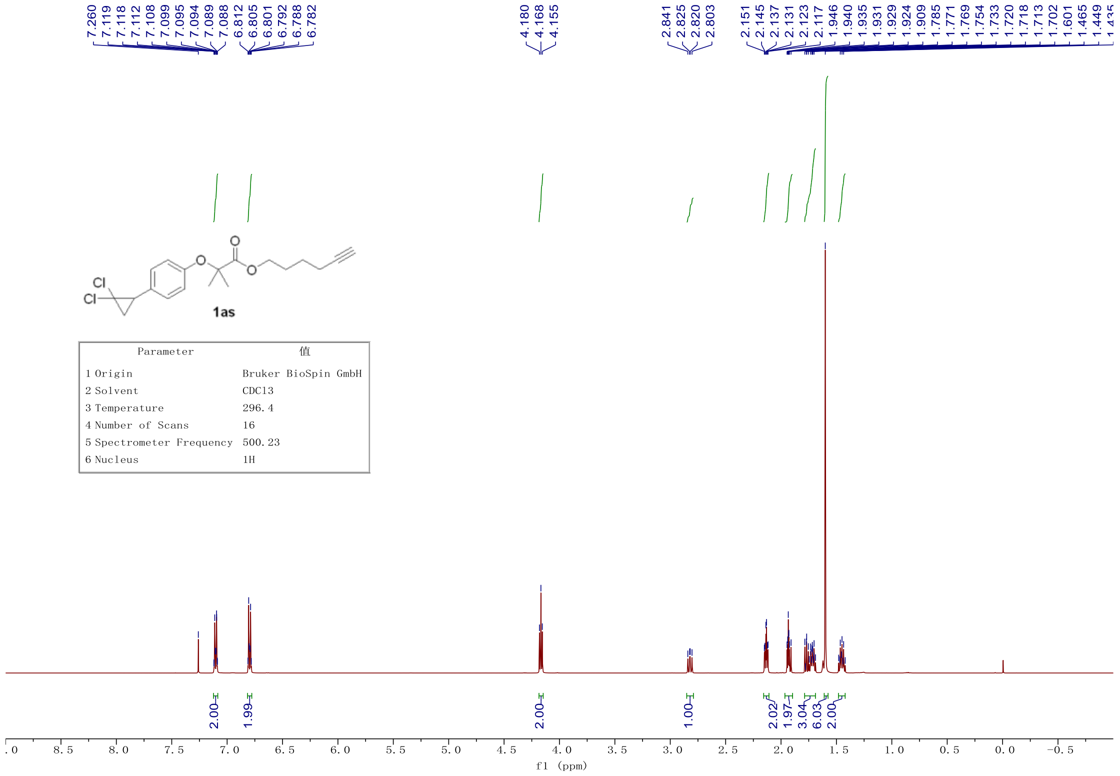

Figure S43. ^{1}H-NMR of **1as.**

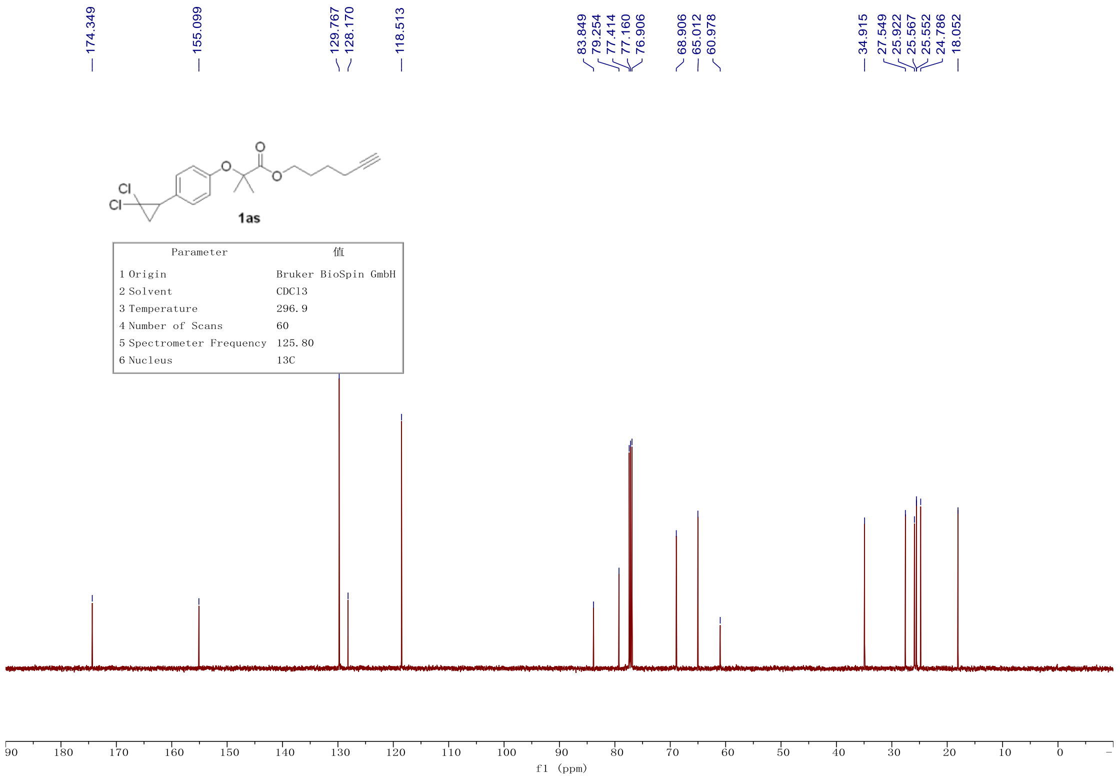

Figure S44. ^{13}C-NMR of **1as.**

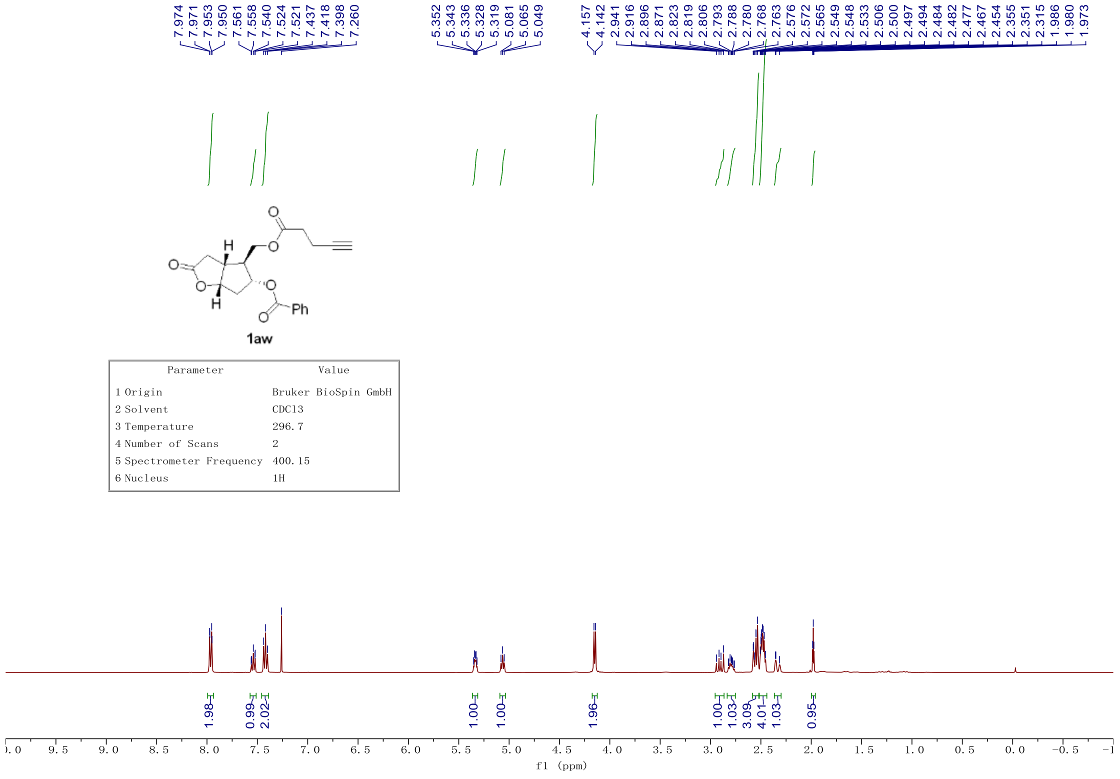

Figure S45. ^{1}H-NMR of **1aw.**

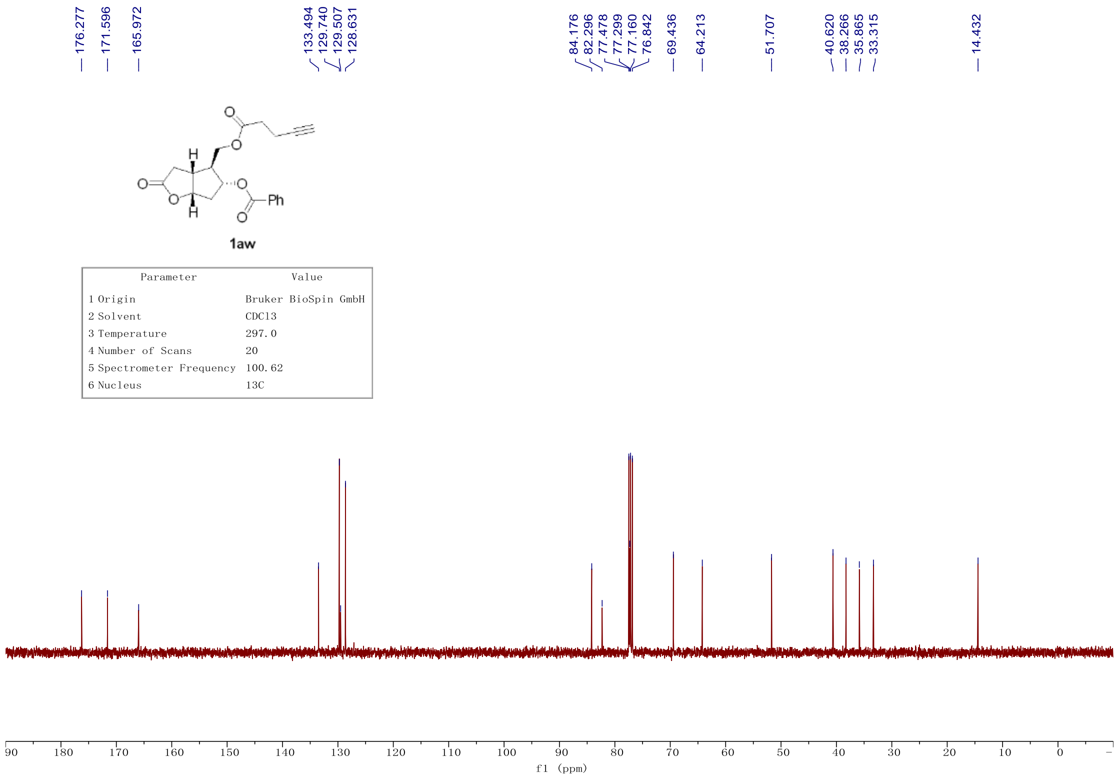

Figure S46. ^{13}C-NMR of **1aw.**

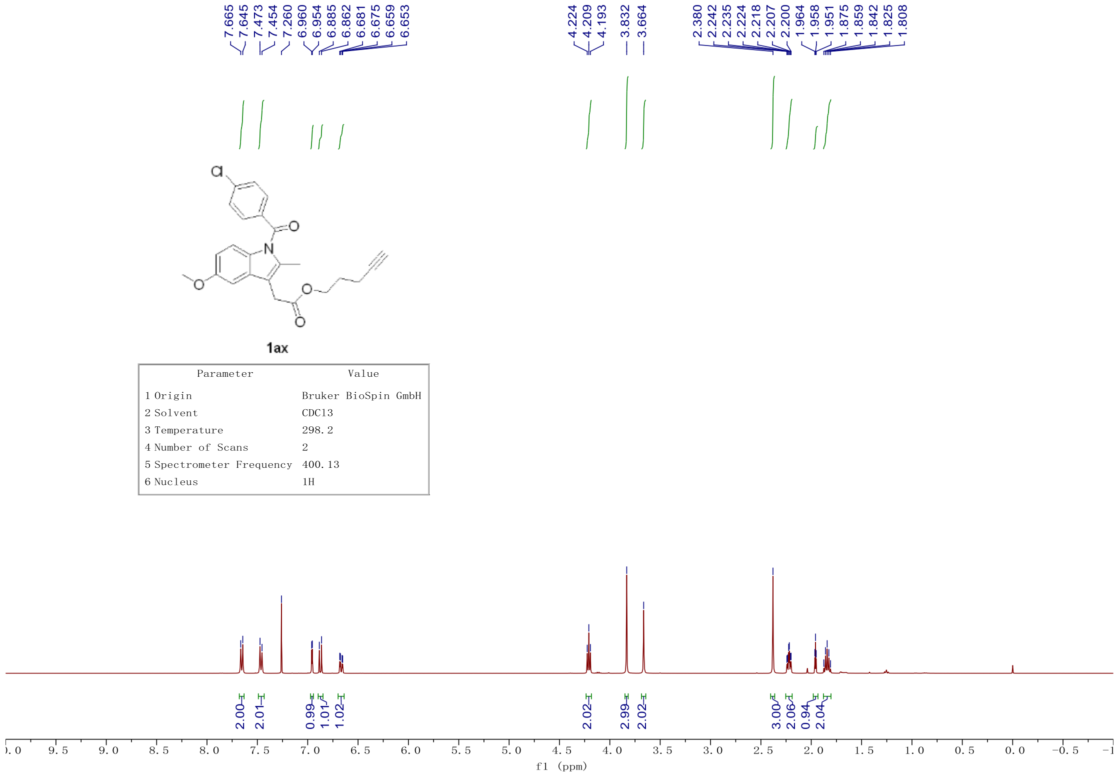

Figure S47. ^{1}H-NMR of **1ax.**

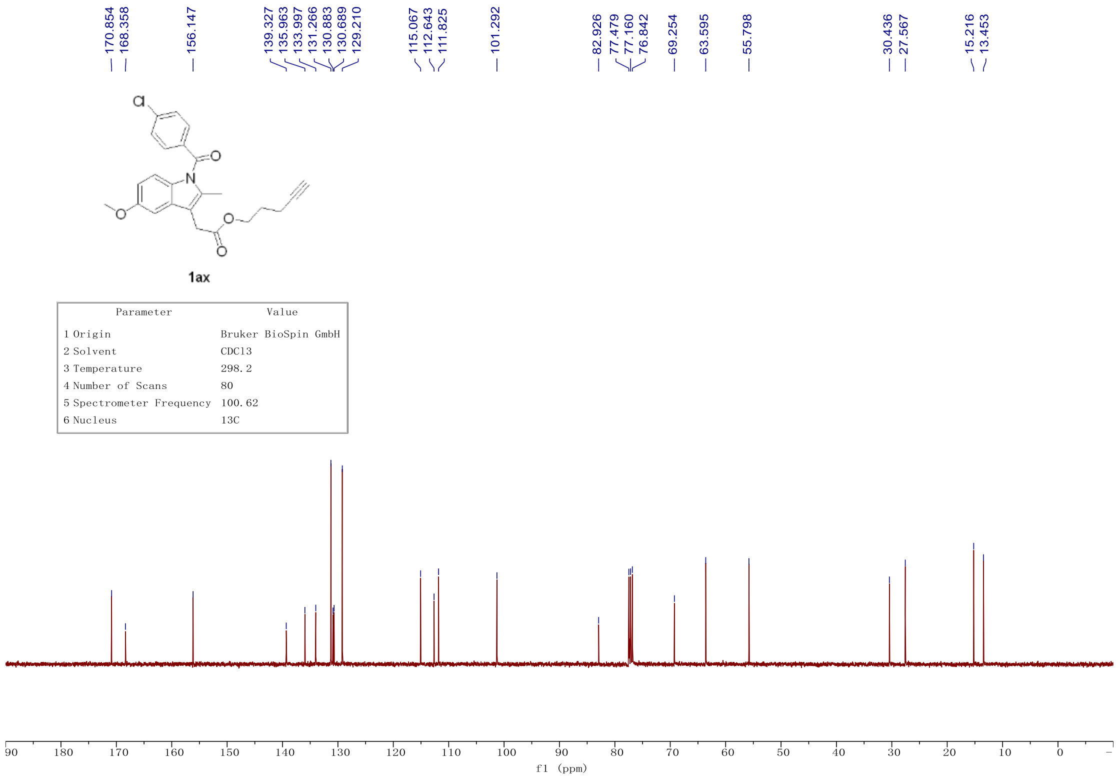

Figure S48. ^{13}C-NMR of **1ax.**

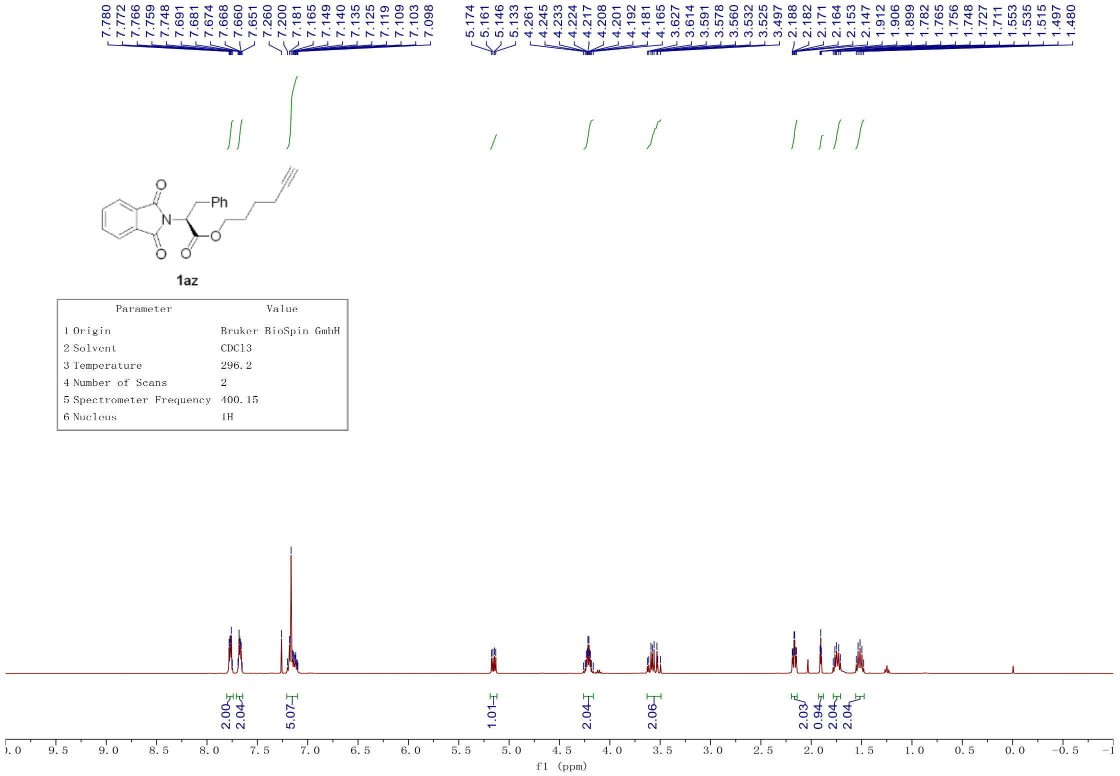

Figure S49. ^{1}H-NMR of **1az.**

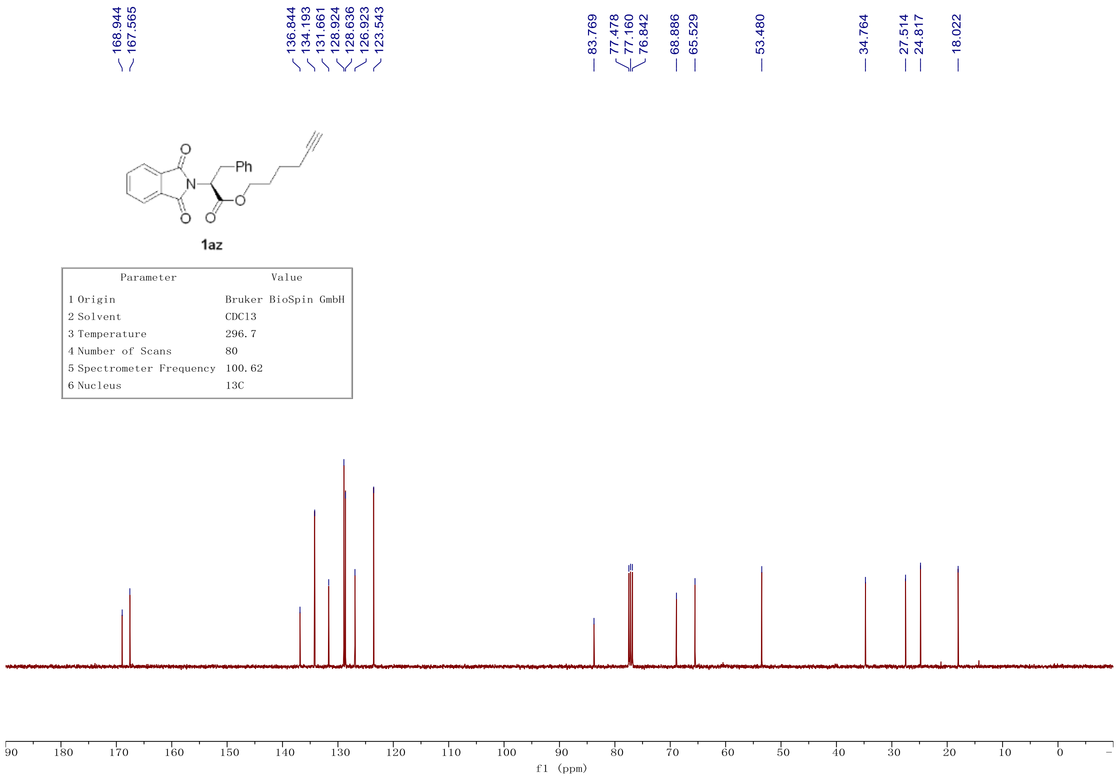

Figure S50. ^{13}C-NMR of **1az.**

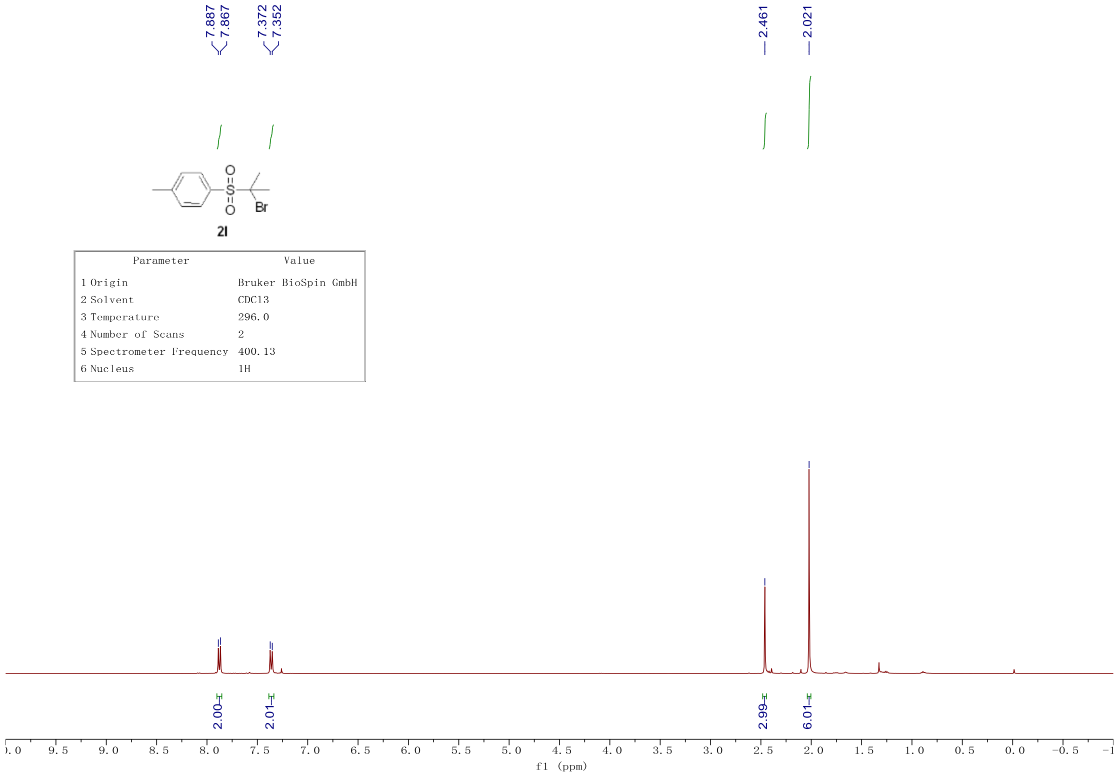

Figure S51. ^{1}H-NMR of **2l.**

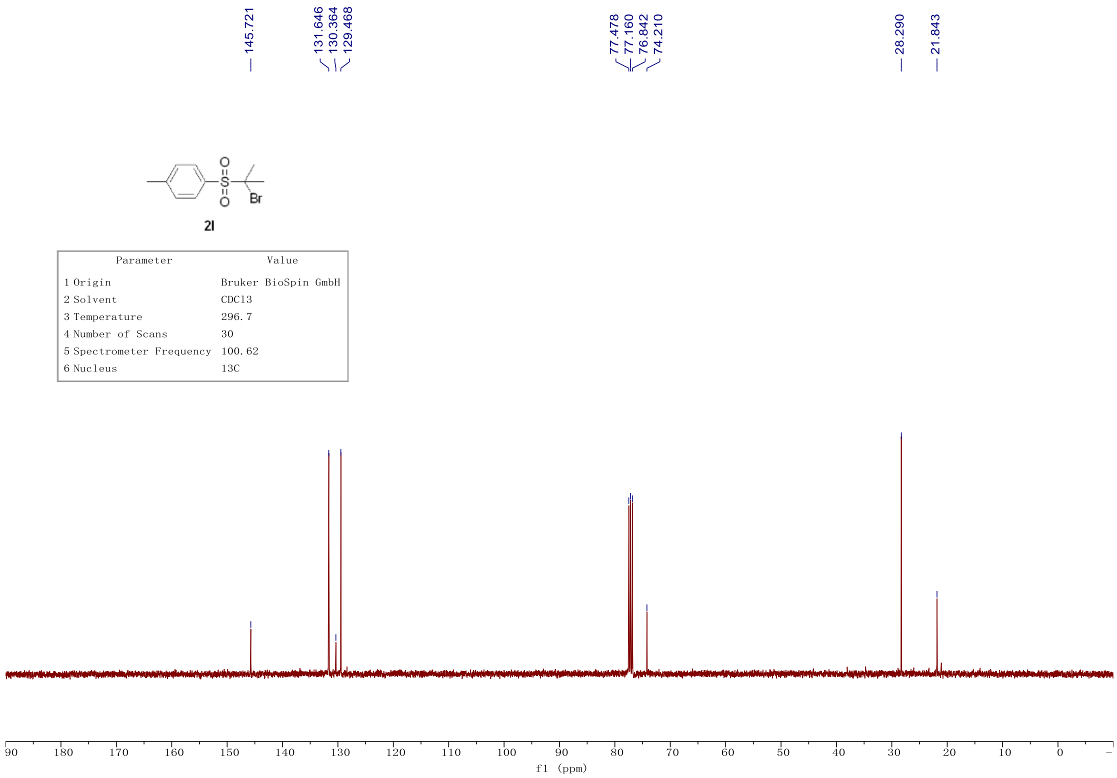

Figure S52. ^{13}C-NMR of **2l.**

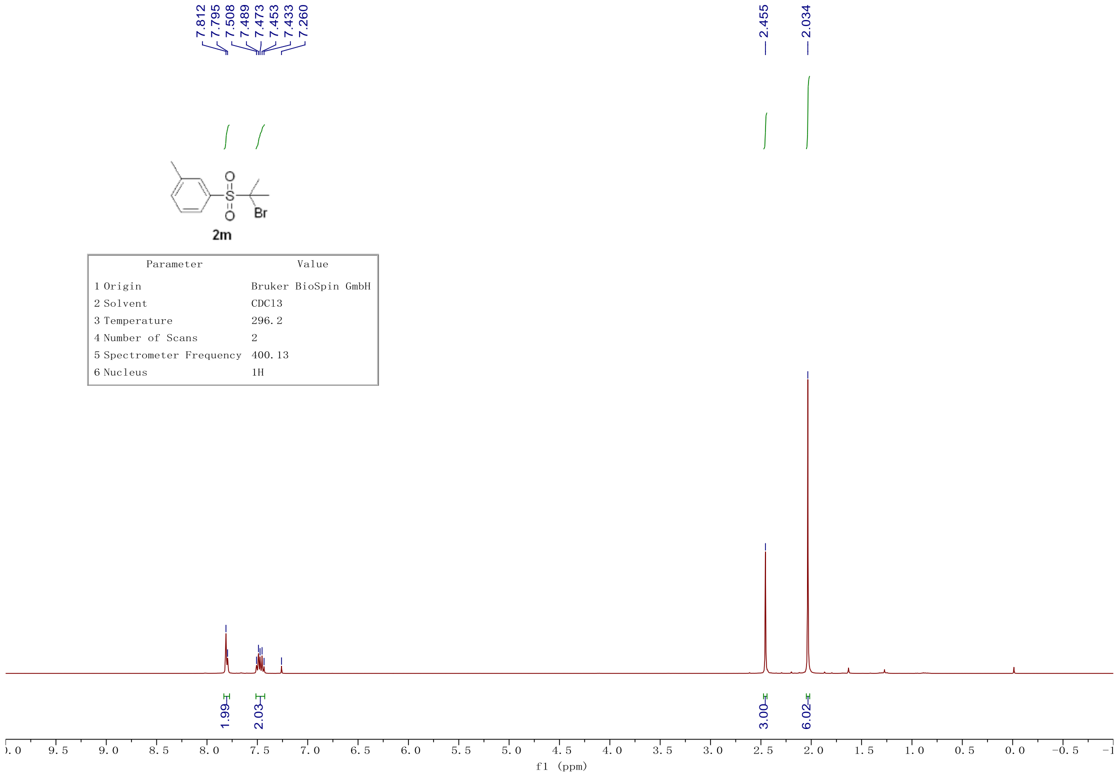

Figure S53. ^{1}H-NMR of **2m.**

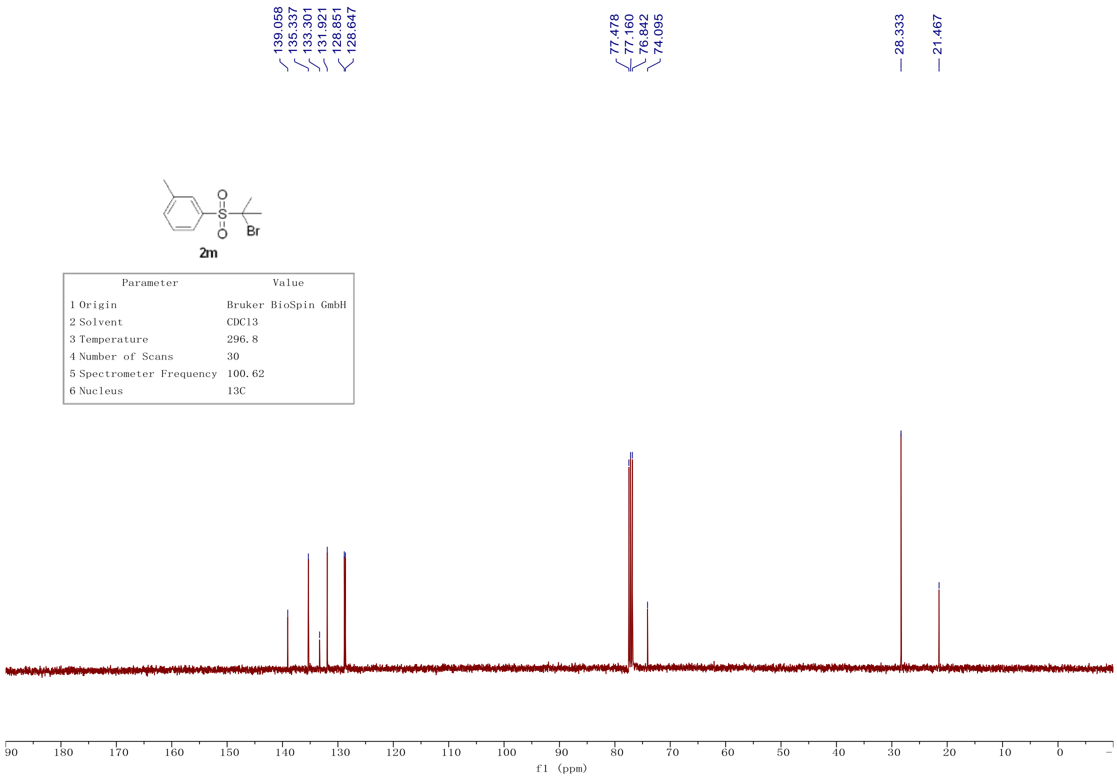

Figure S54. ^{13}C-NMR of **2m.**

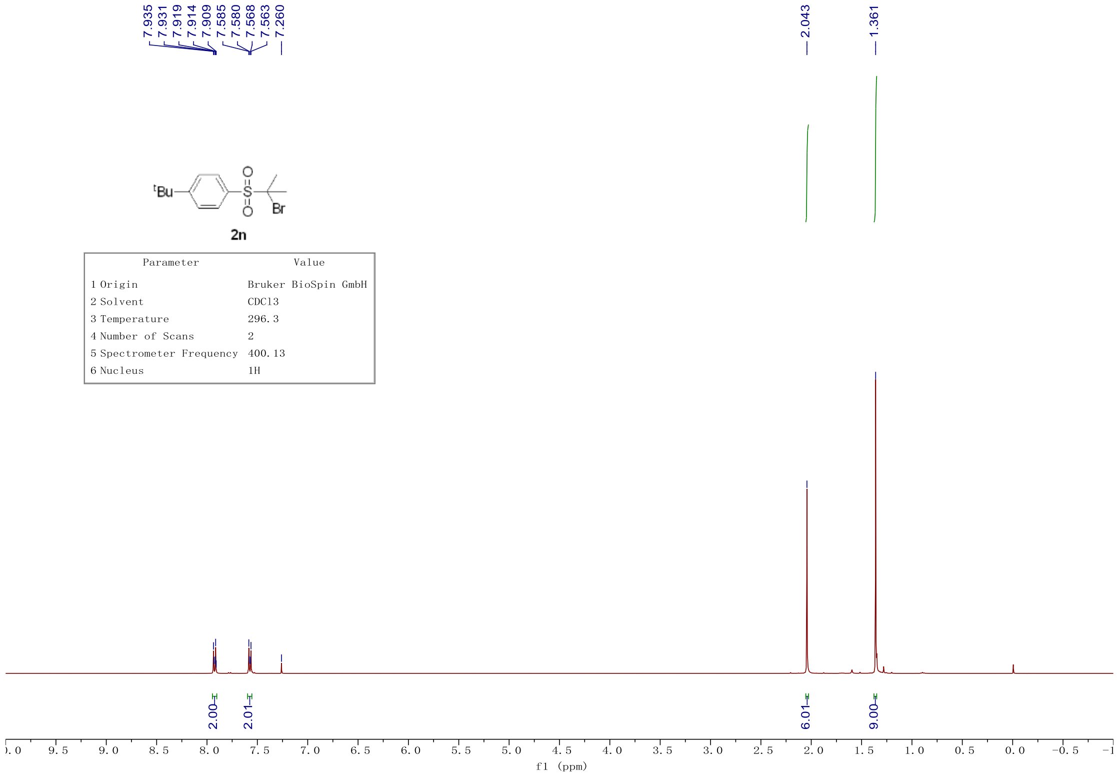

Figure S55. ^{1}H-NMR of **2n.**

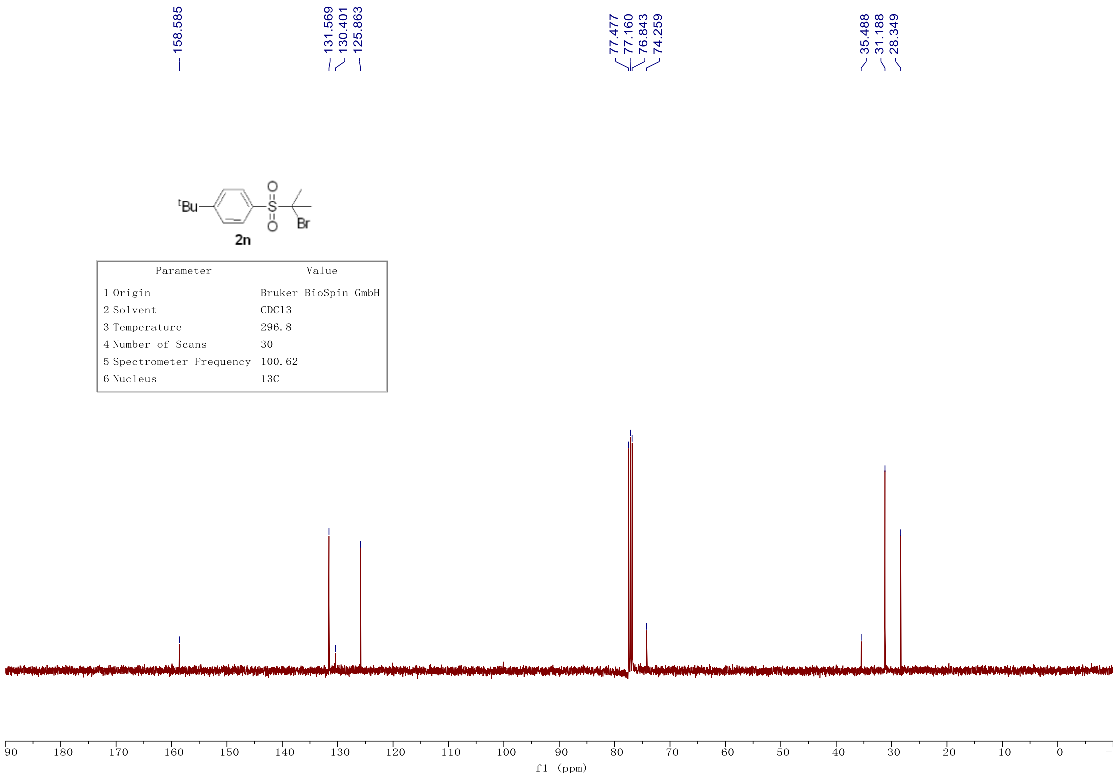

Figure S56. ^{13}C-NMR of **2n.**

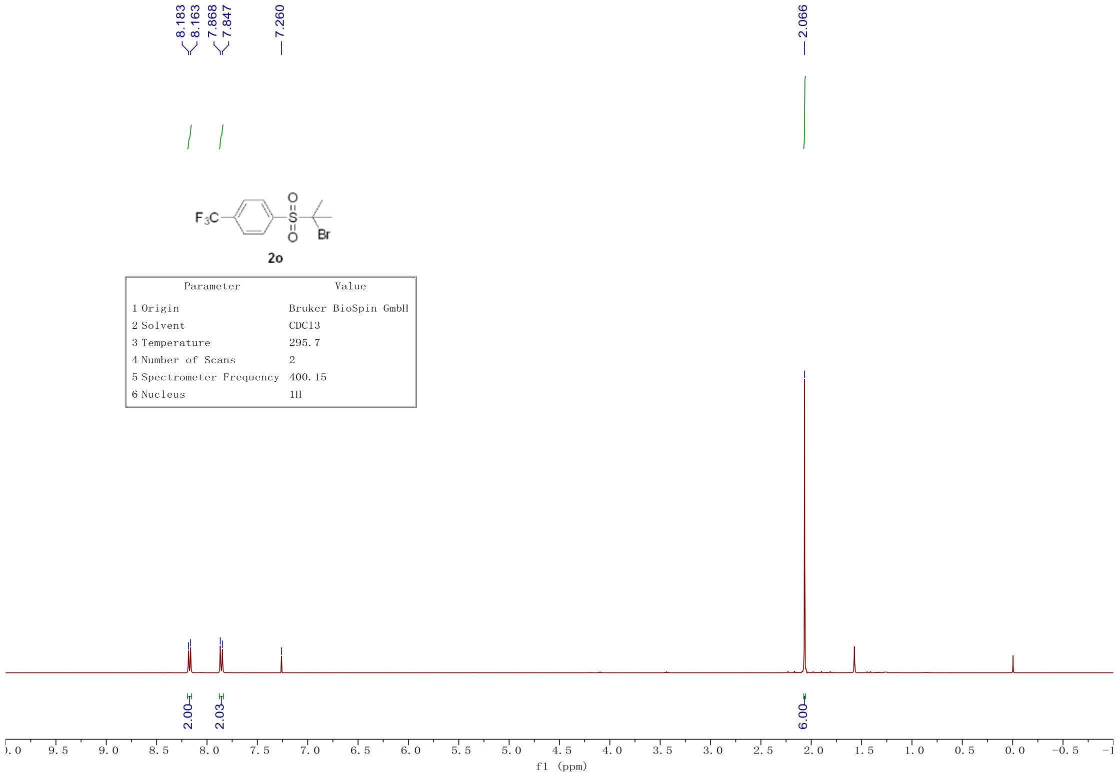

Figure S57. ^{1}H-NMR of **2o.**

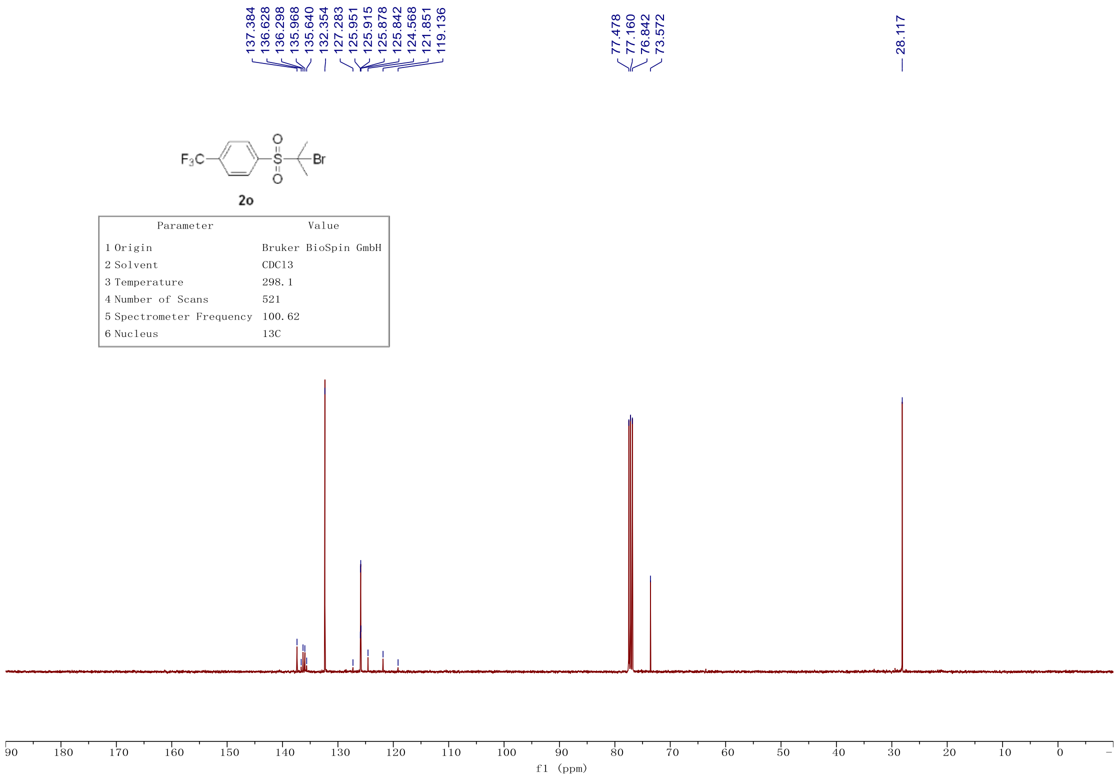

Figure S58. ^{13}C-NMR of **2o.**

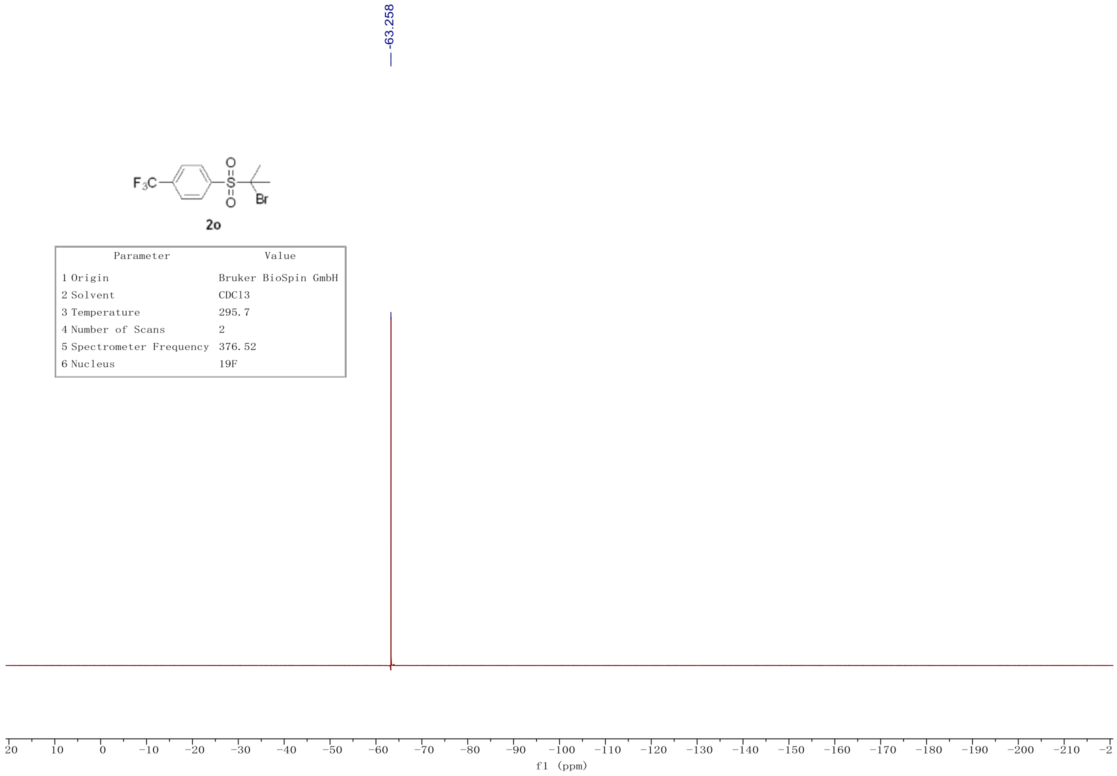

Figure S59. ^{19}F-NMR of **2o.**

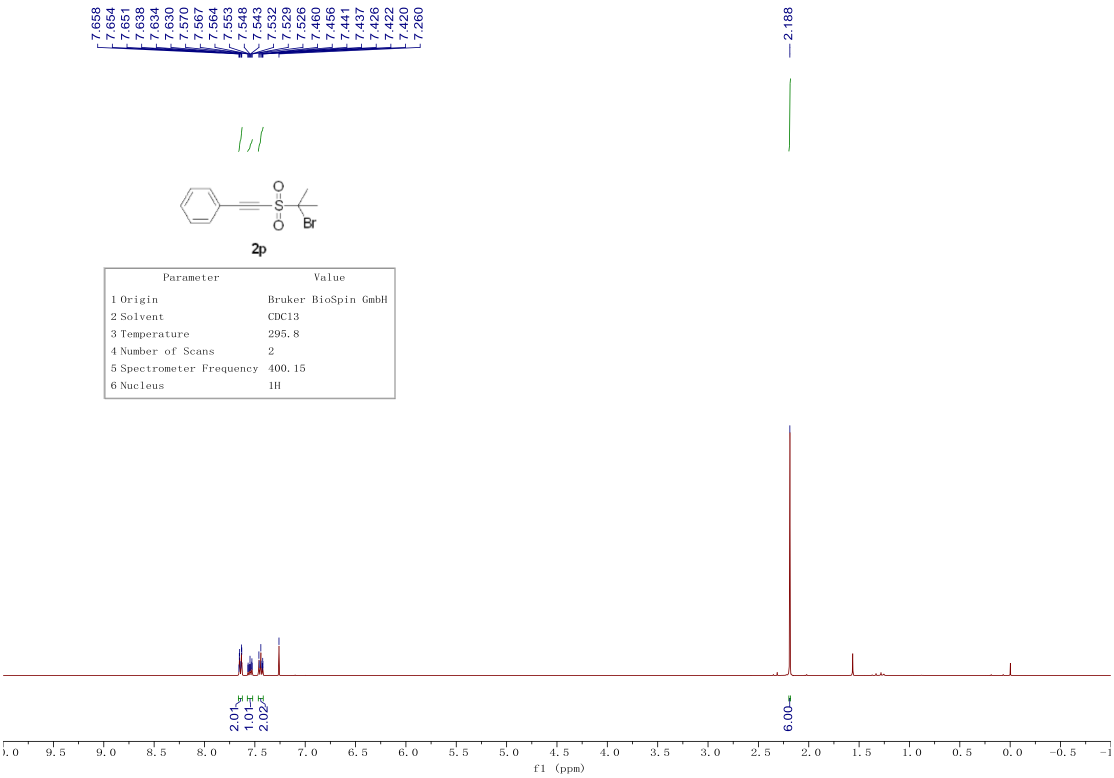

Figure S60. ^{1}H-NMR of **2p.**

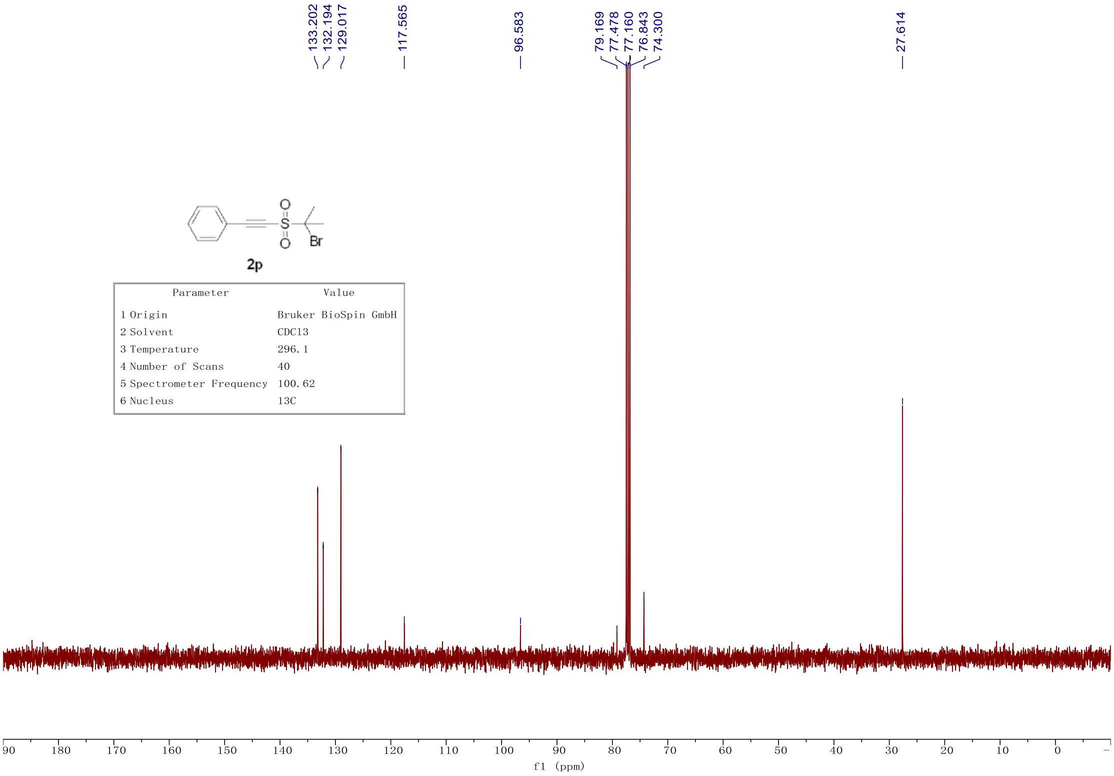

Figure S61. ^{13}C-NMR of **2p.**

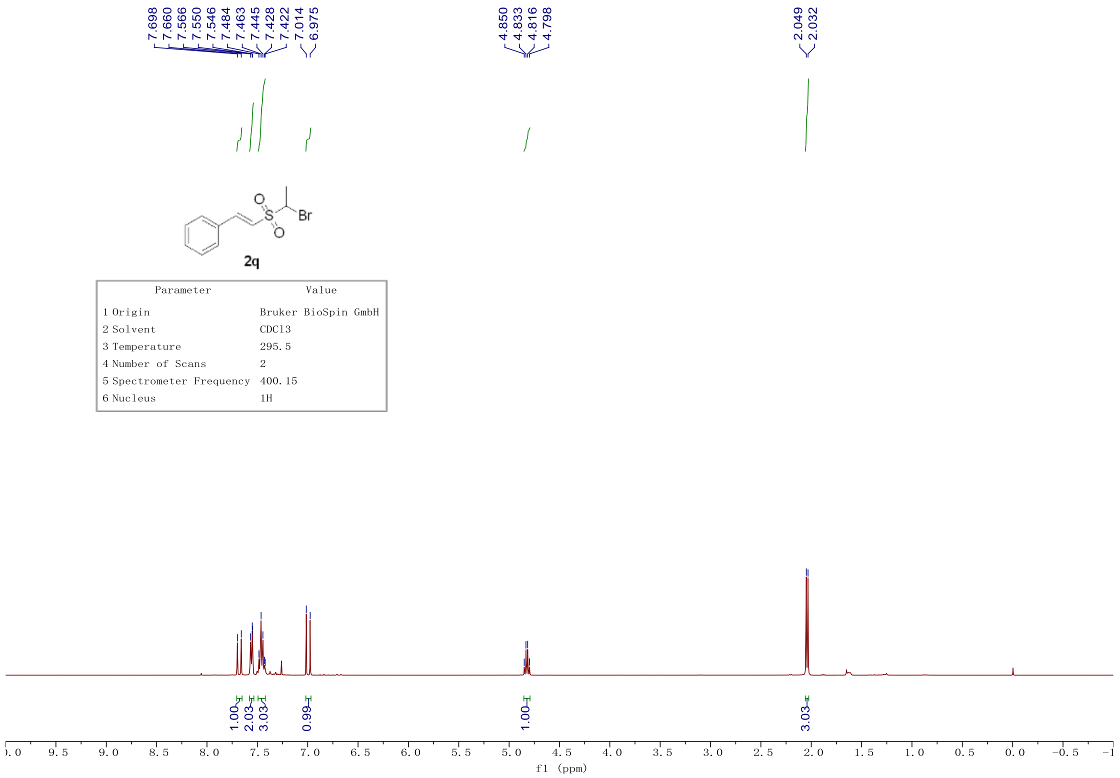

Figure S62. ^{1}H-NMR of **2q.**

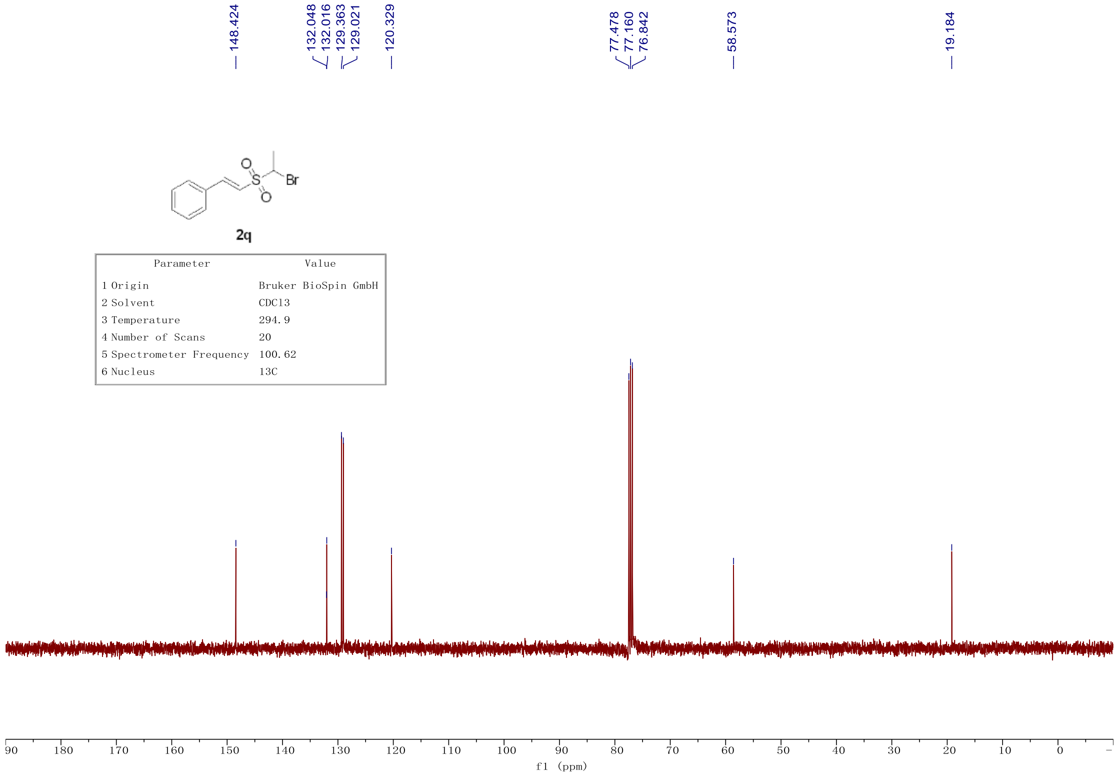

Figure S63. ^{13}C-NMR of **2q.**

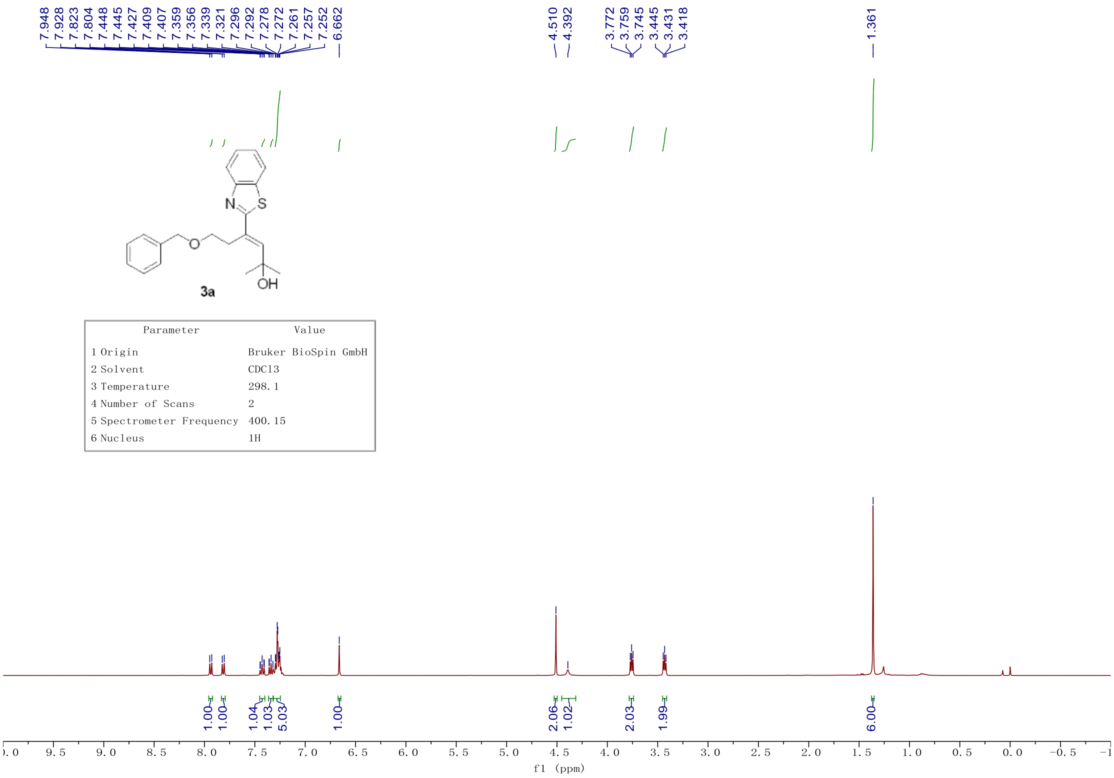

Figure S64. ^{1}H-NMR of **3a.**

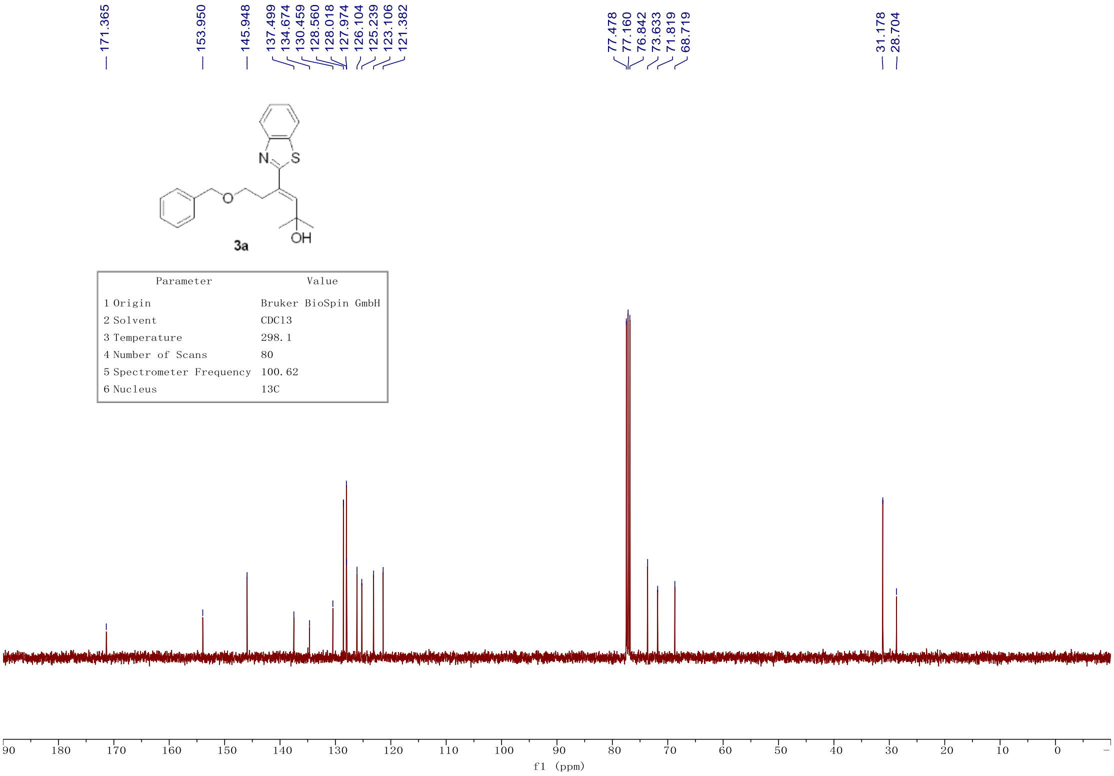

Figure S65. ^{13}C-NMR of **3a.**

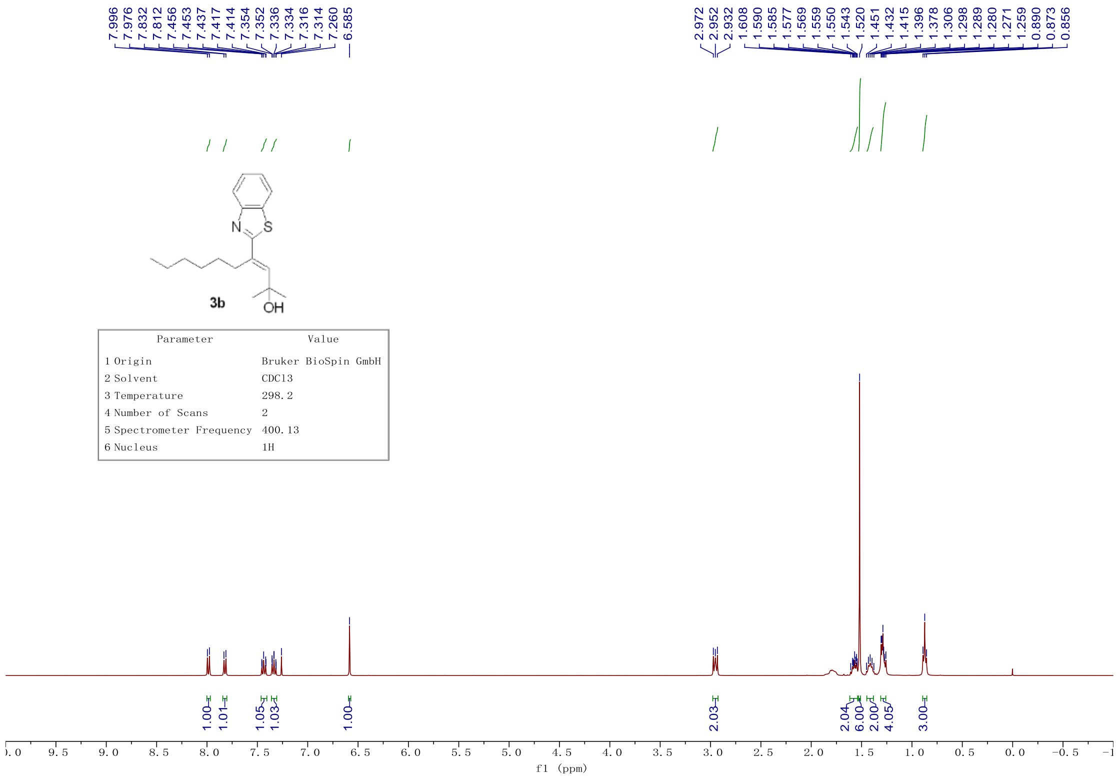

Figure S66. ^{1}H-NMR of **3b.**

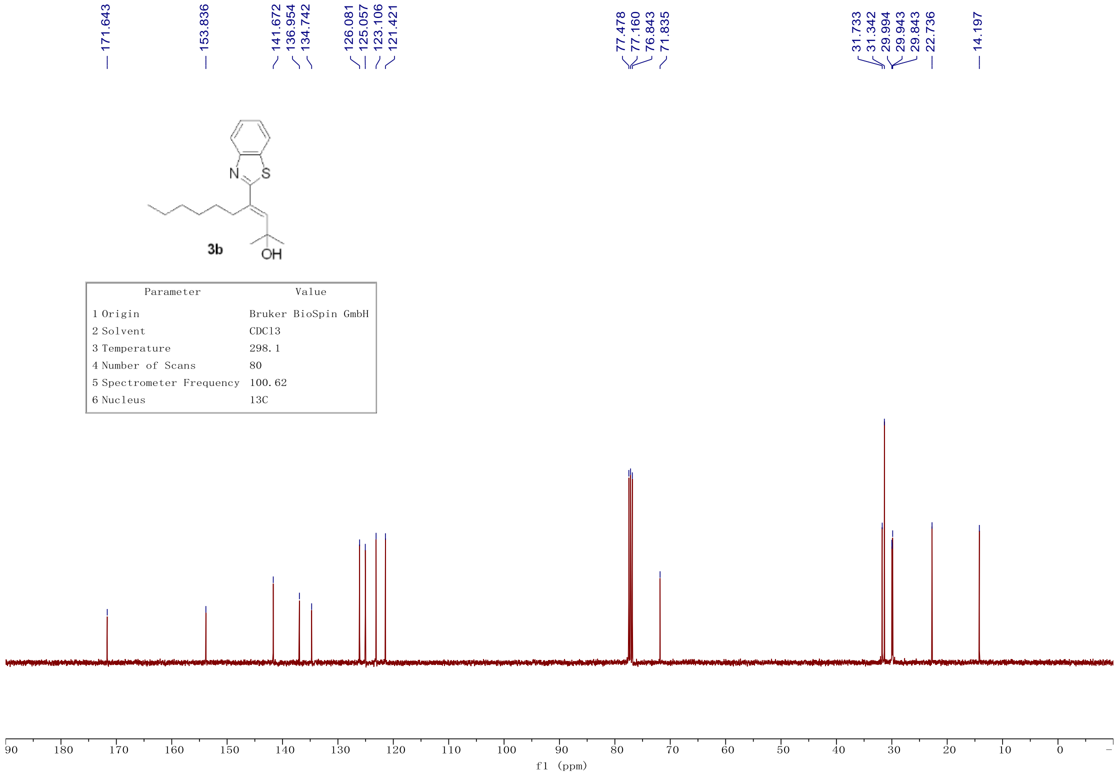

Figure S67. ^{13}C-NMR of **3b.**

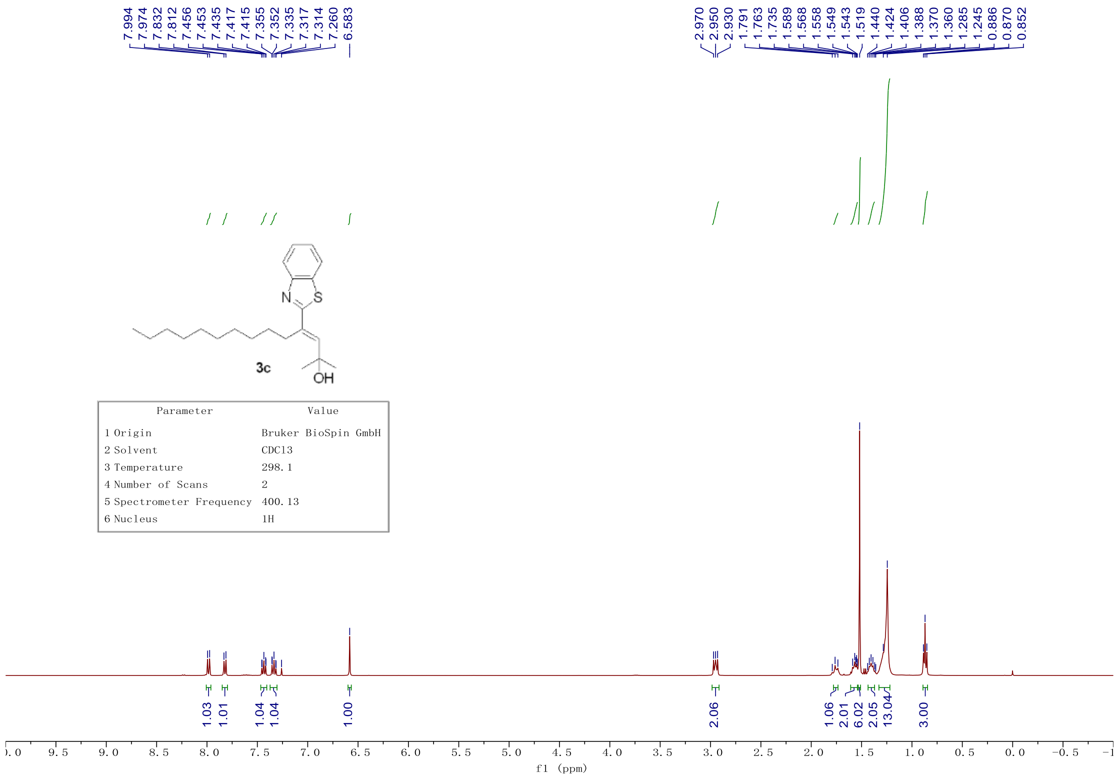

Figure S68. ^{1}H-NMR of **3c.**

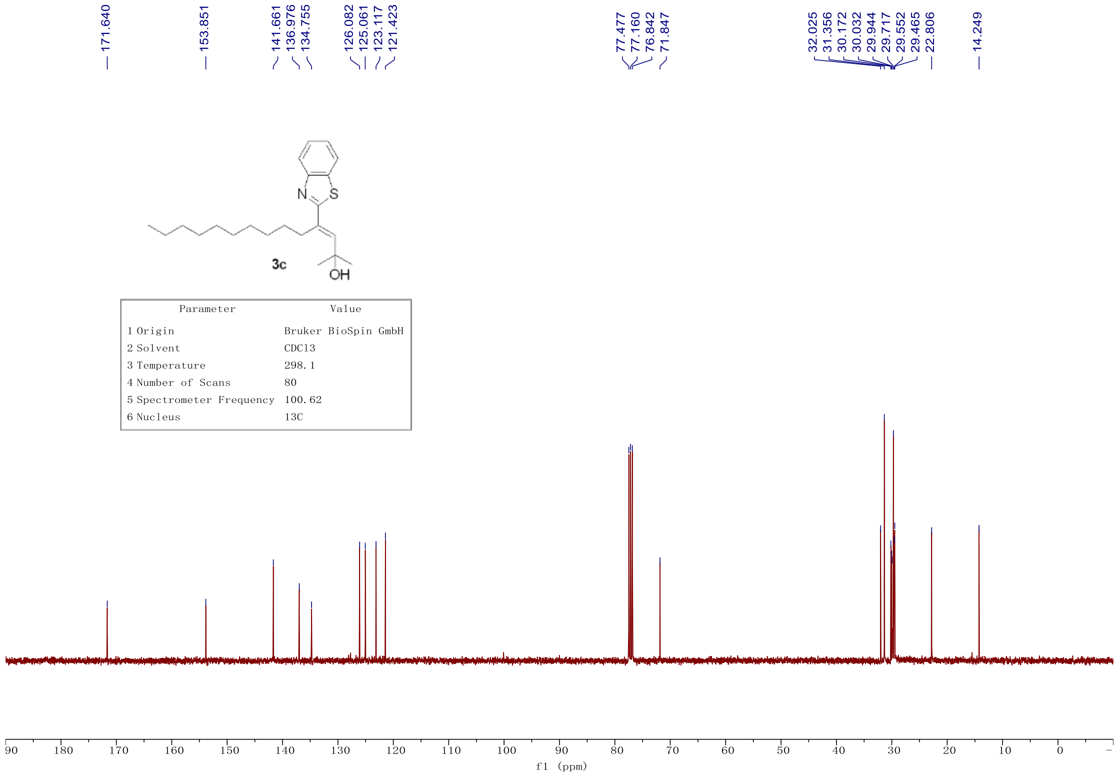

Figure S69. ^{13}C-NMR of **3c.**

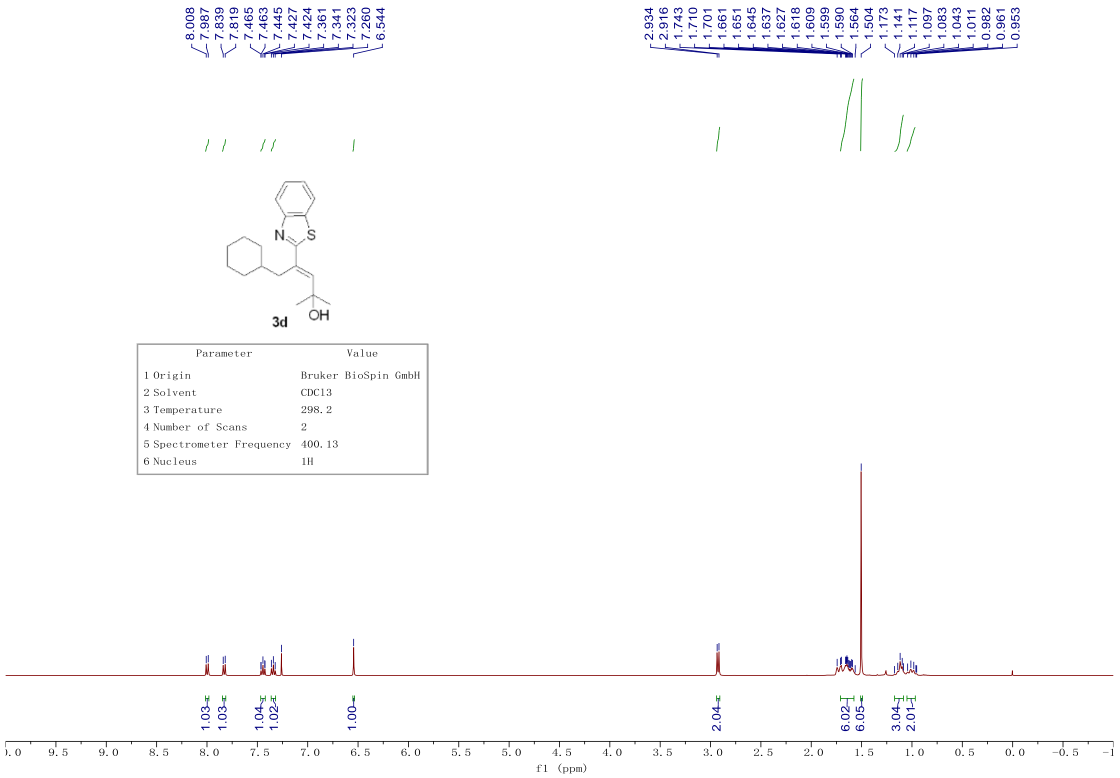

Figure S70. ^{1}H-NMR of **3d.**

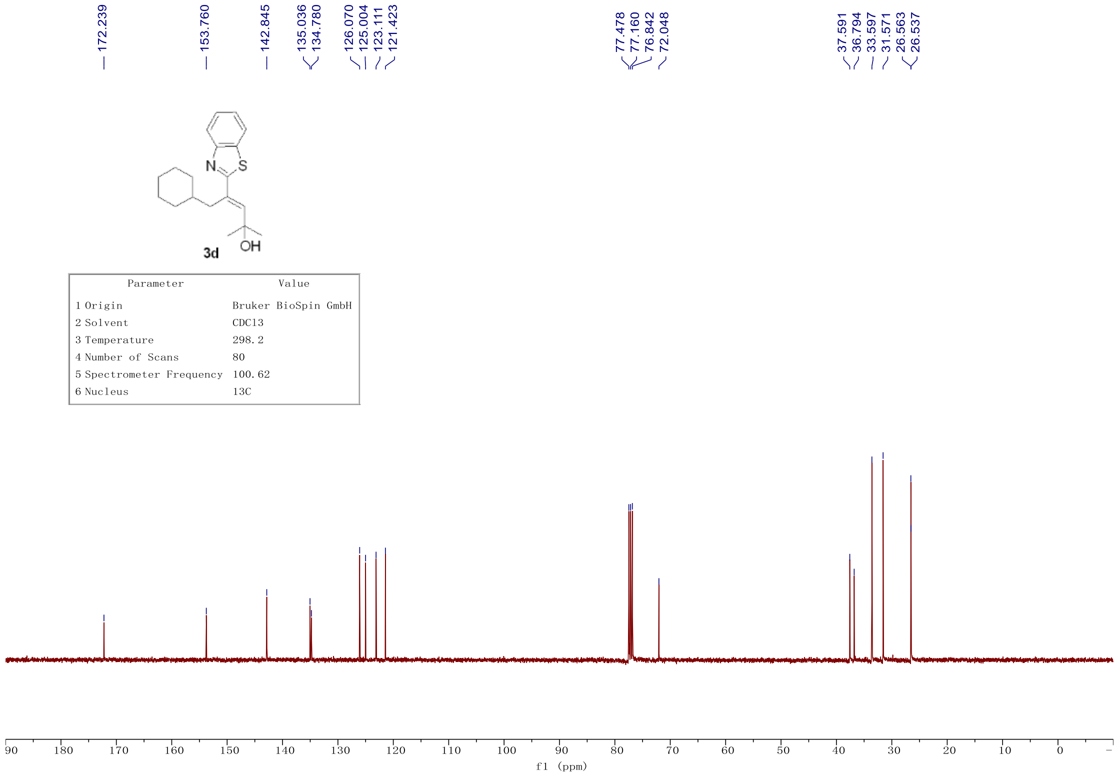

Figure S71. ^{13}C-NMR of **3d.**

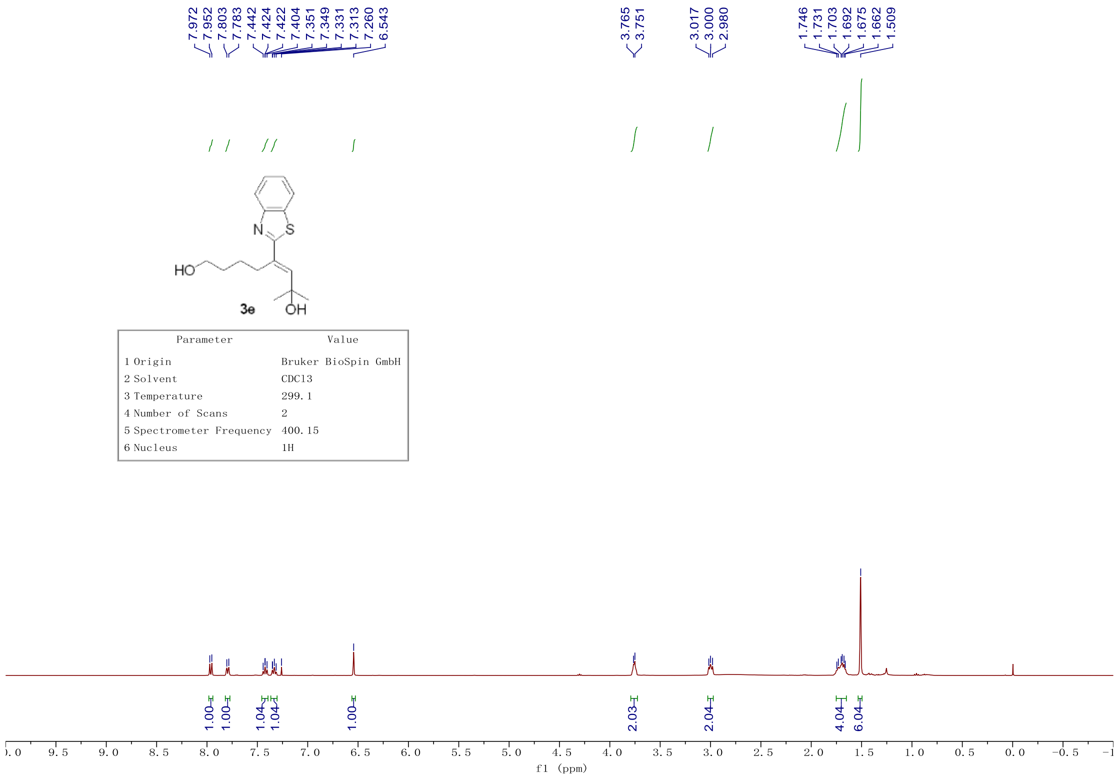

Figure S72. ^{1}H-NMR of **3e.**

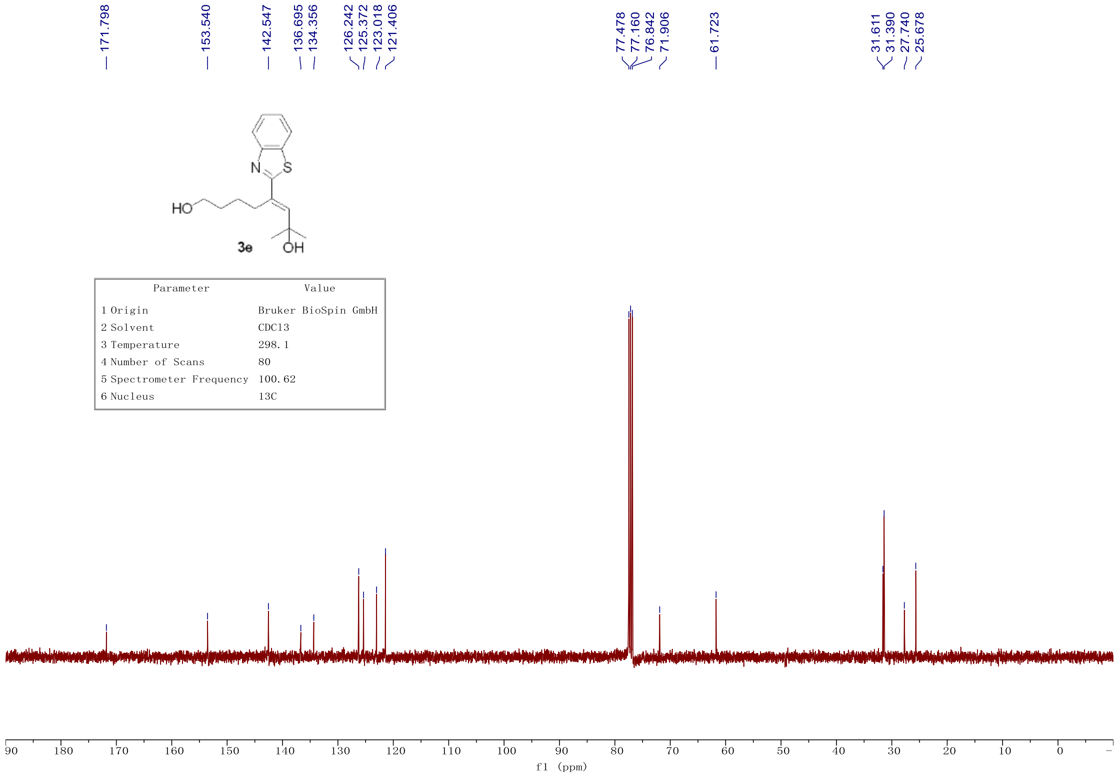

Figure S73. ^{13}C-NMR of **3e.**

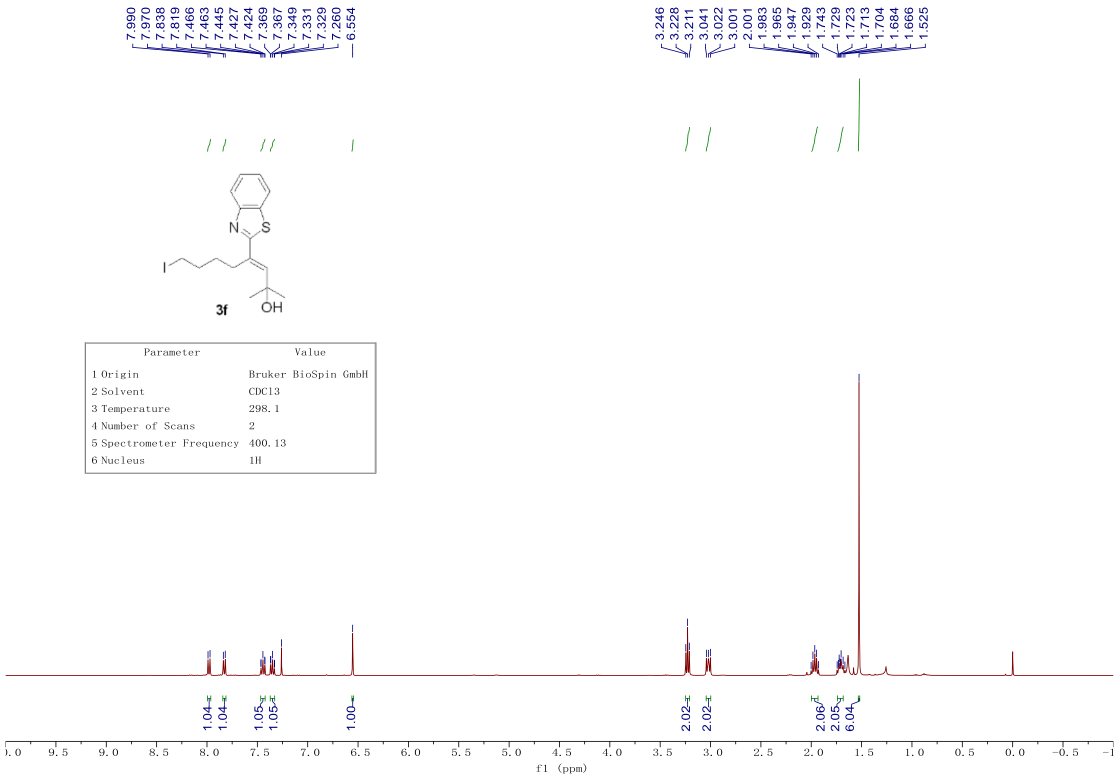

Parameter	Value
1 Origin	Bruker BioSpin GmbH
2 Solvent	CDCl3
3 Temperature	298.1
4 Number of Scans	2
5 Spectrometer Frequency	400.13
6 Nucleus	1H

Figure S74. ^{1}H-NMR of **3f.**

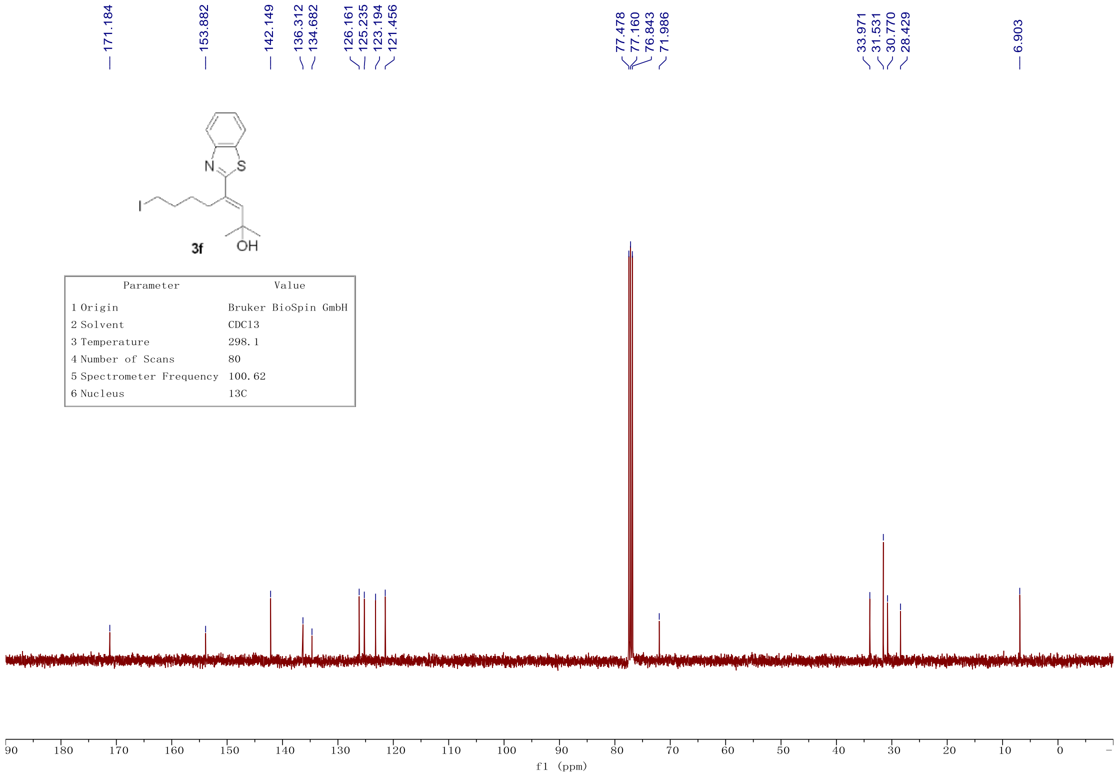

Parameter	Value
1 Origin	Bruker BioSpin GmbH
2 Solvent	CDCl3
3 Temperature	298.1
4 Number of Scans	80
5 Spectrometer Frequency	100.62
6 Nucleus	13C

Figure S75. ^{13}C-NMR of **3f.**

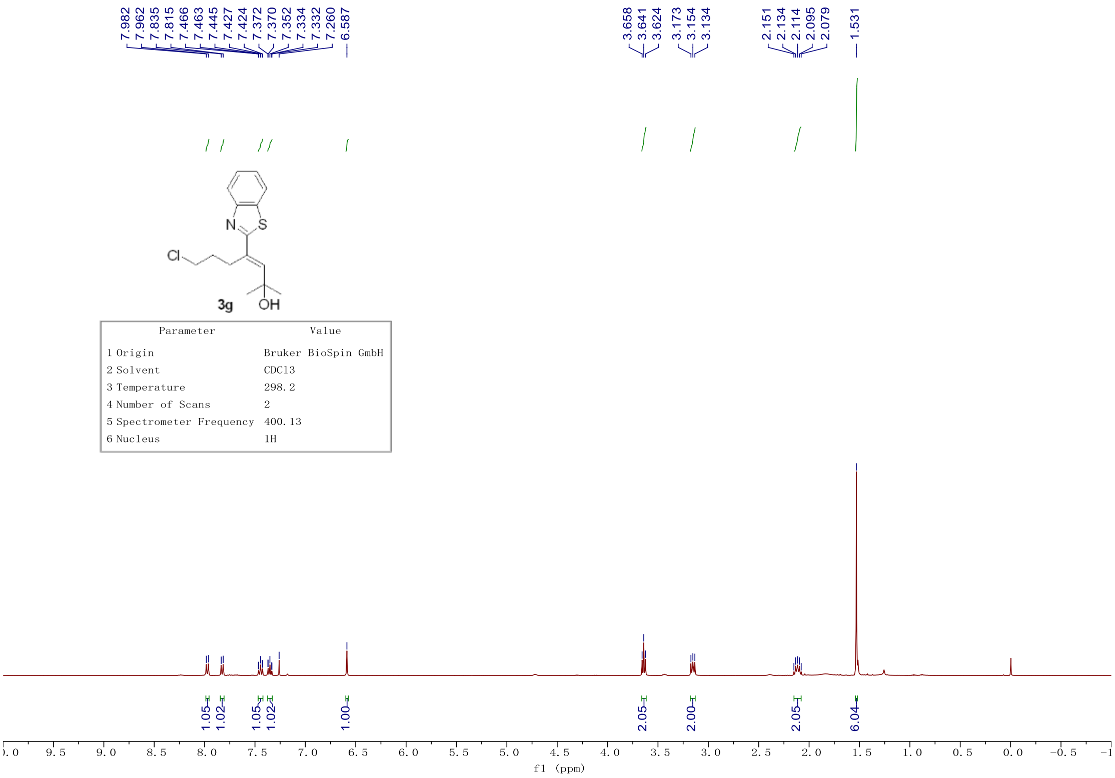

Figure S76. ^{1}H-NMR of **3g.**

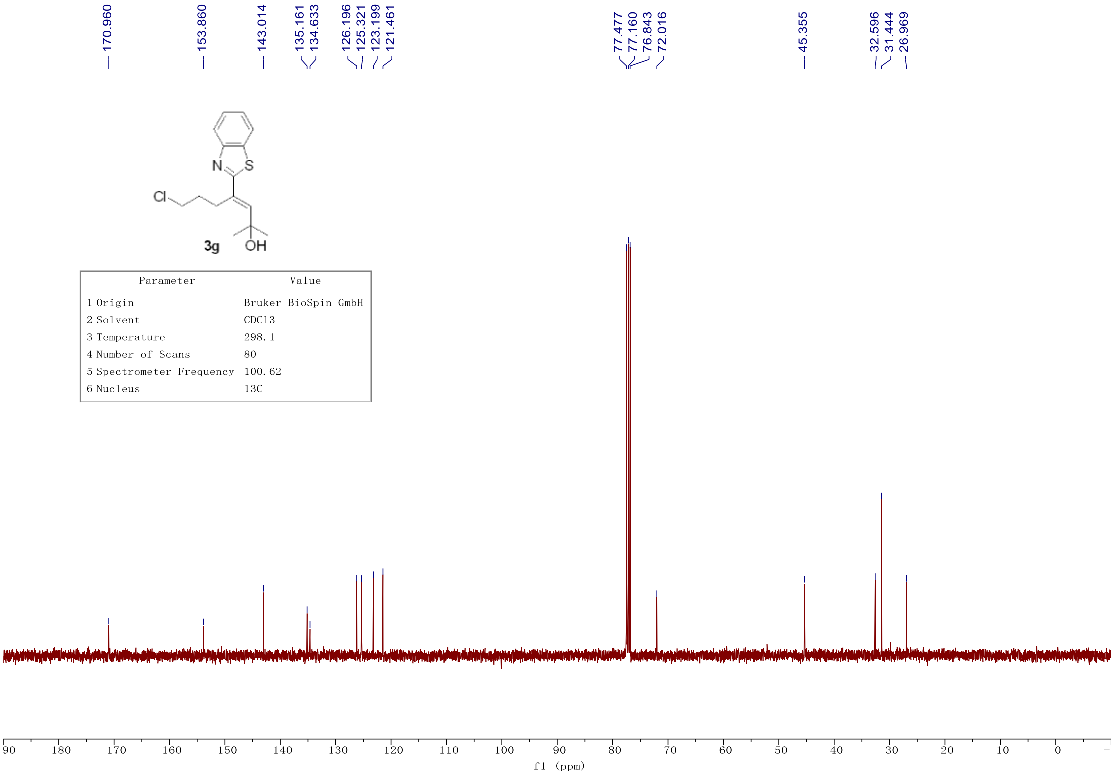

Figure S77. ^{13}C-NMR of **3g.**

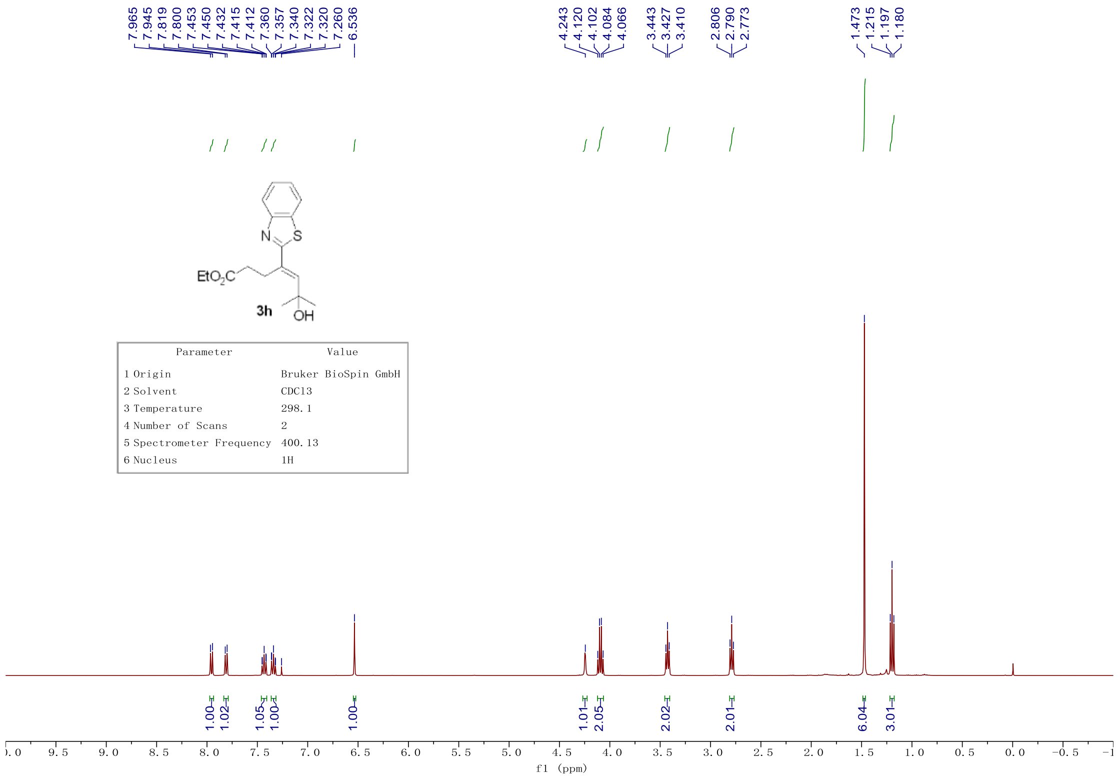

Figure S78. ^{1}H-NMR of **3h.**

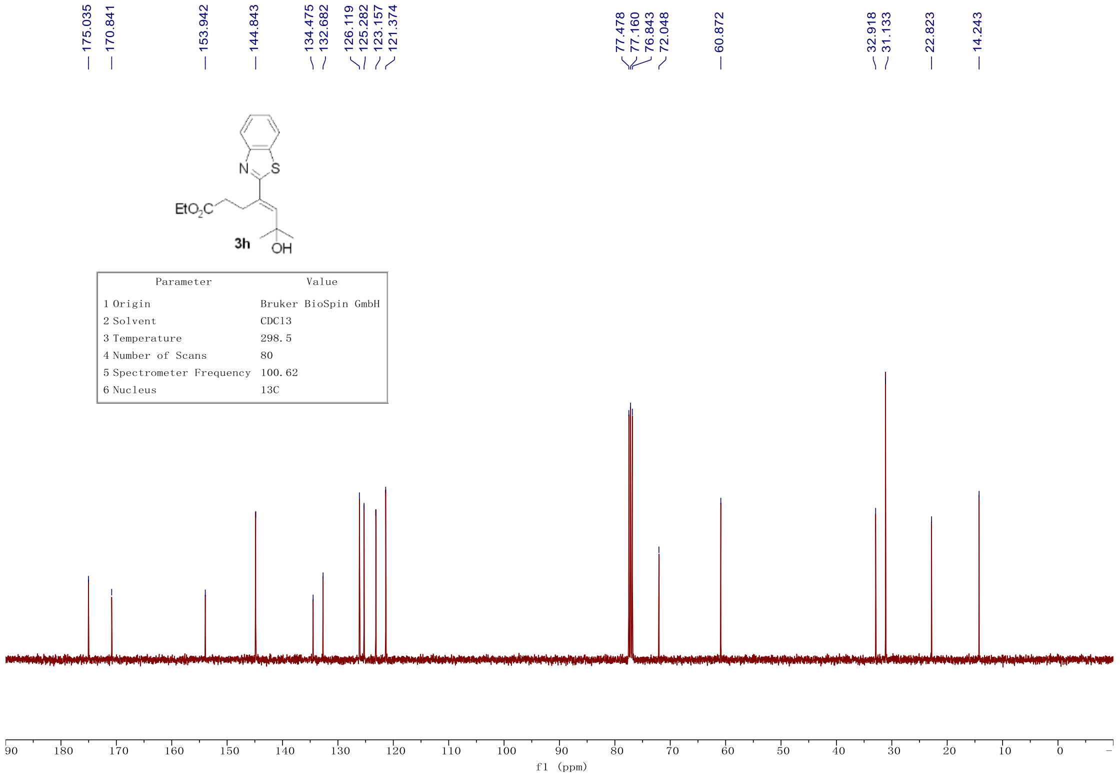

Figure S79. ^{13}C-NMR of **3h.**

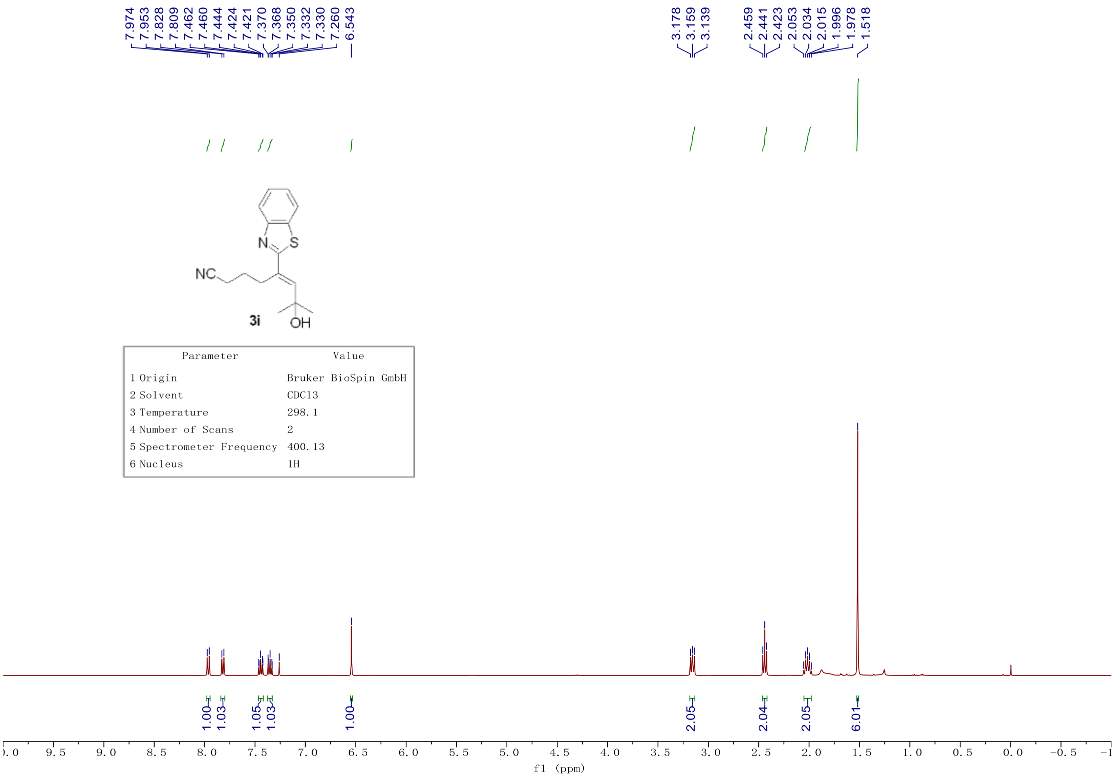

Figure S80. ^{1}H-NMR of **3i.**

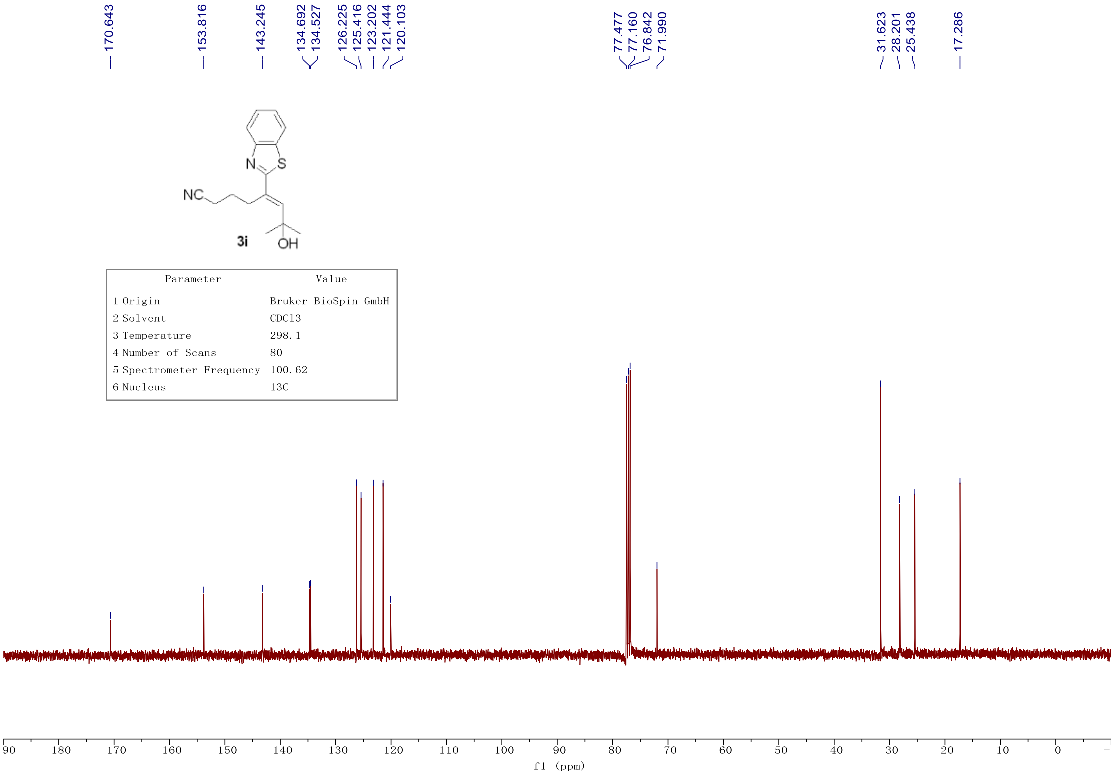

Figure S81. ^{13}C-NMR of **3i.**

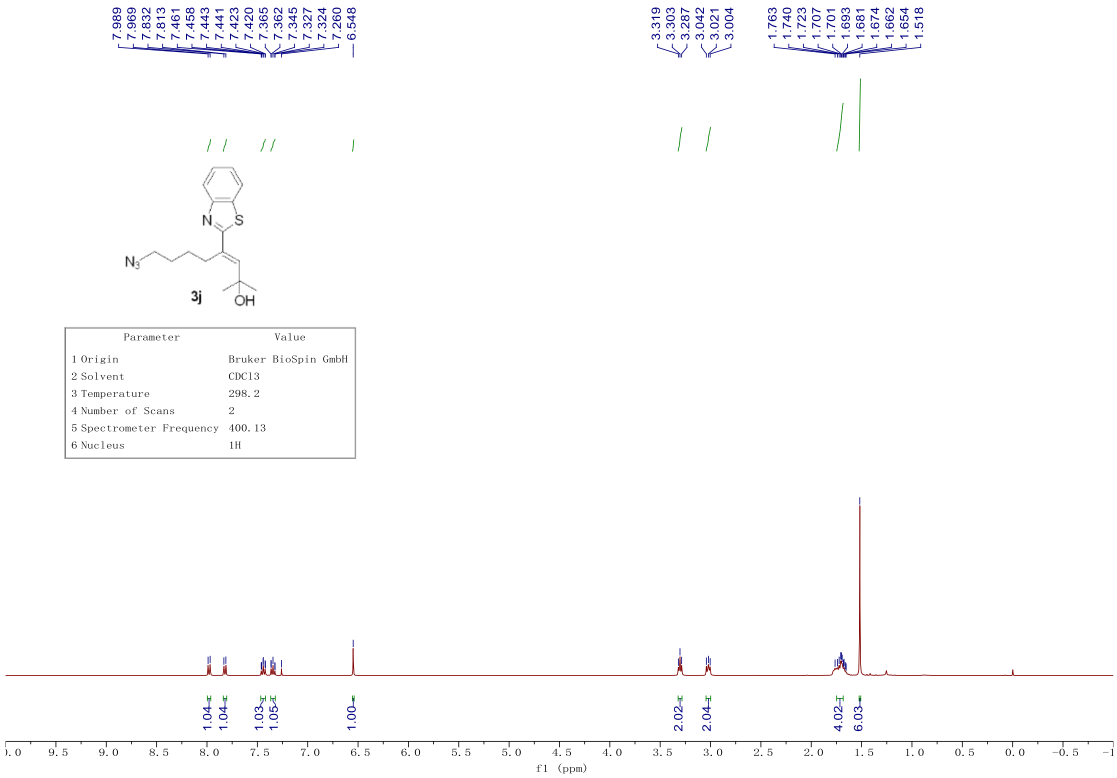

Figure S82. ^{1}H-NMR of **3j.**

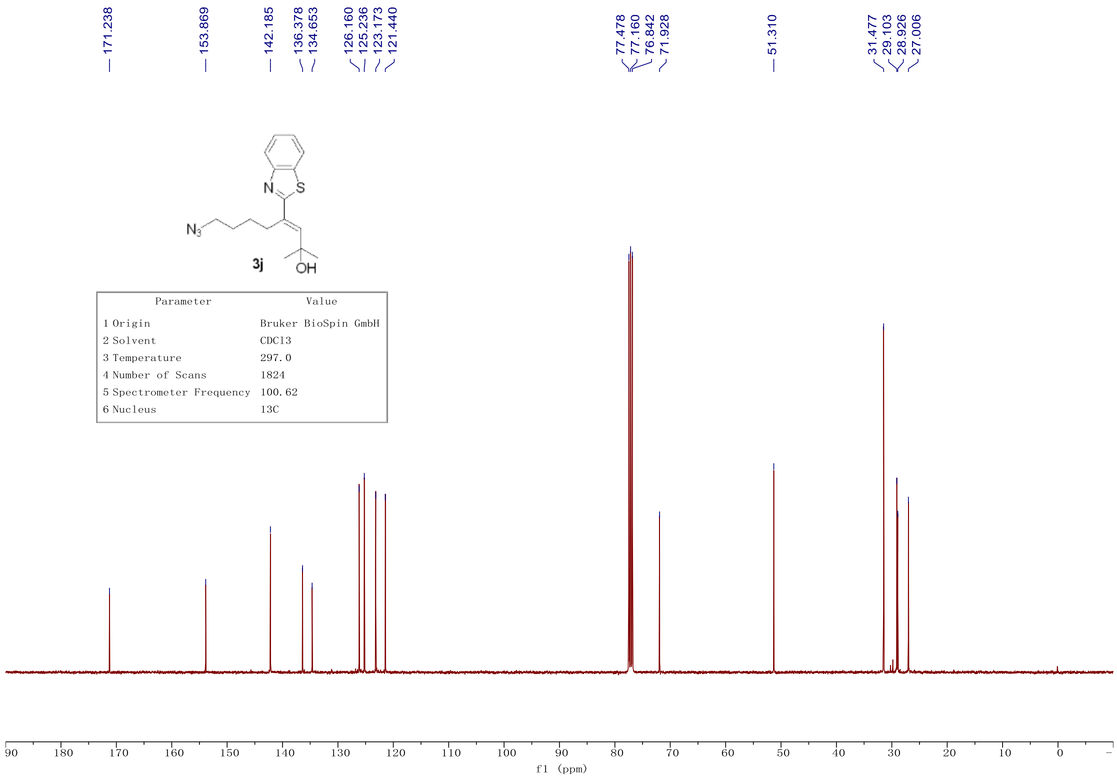

Figure S83. ^{13}C-NMR of **3j.**

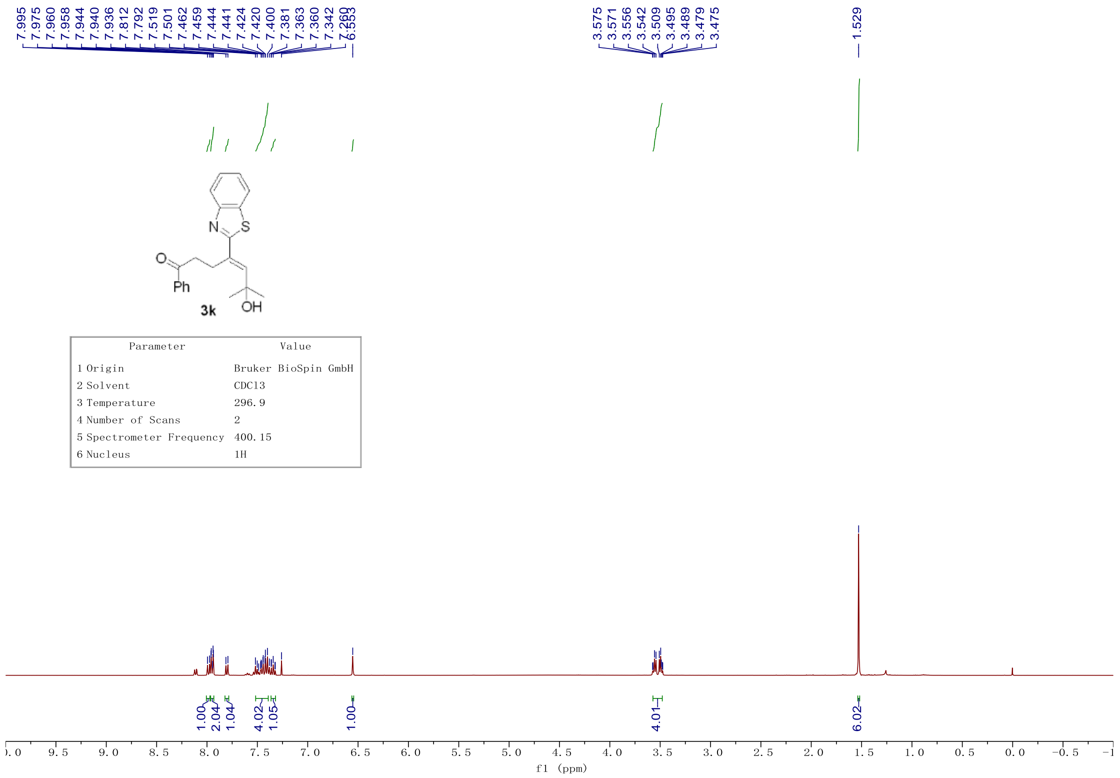

Figure S84. ^{1}H-NMR of **3k.**

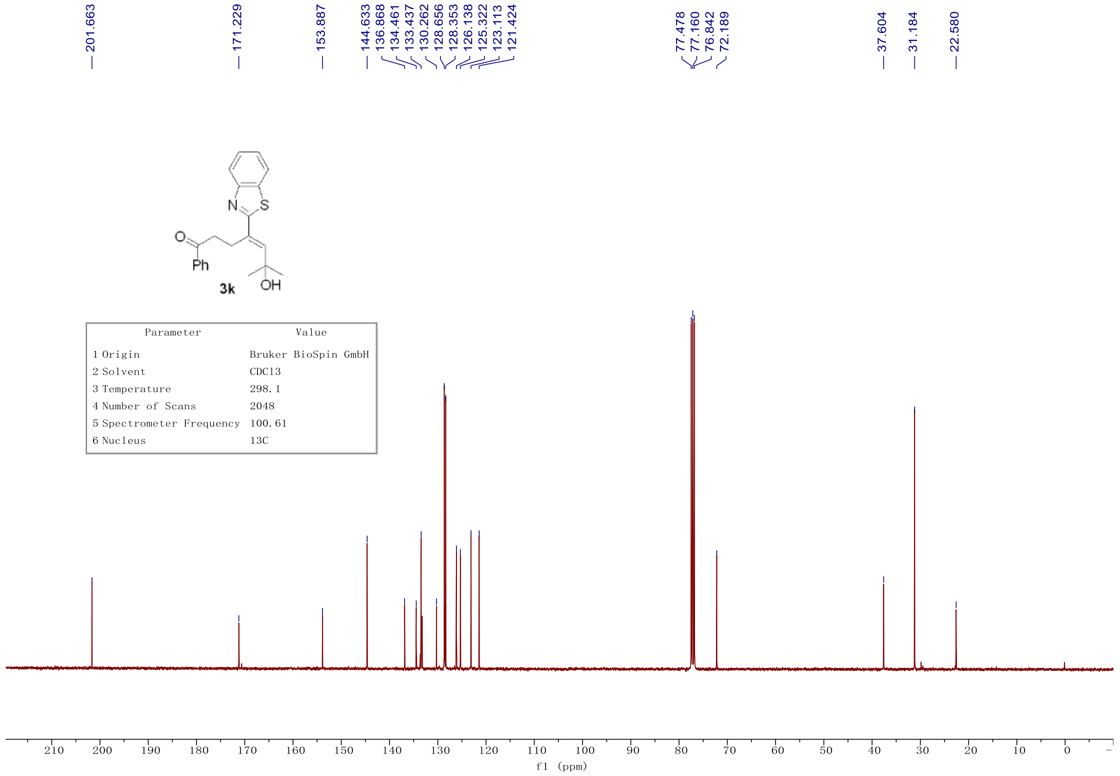

Figure S85. ^{13}C-NMR of **3k.**

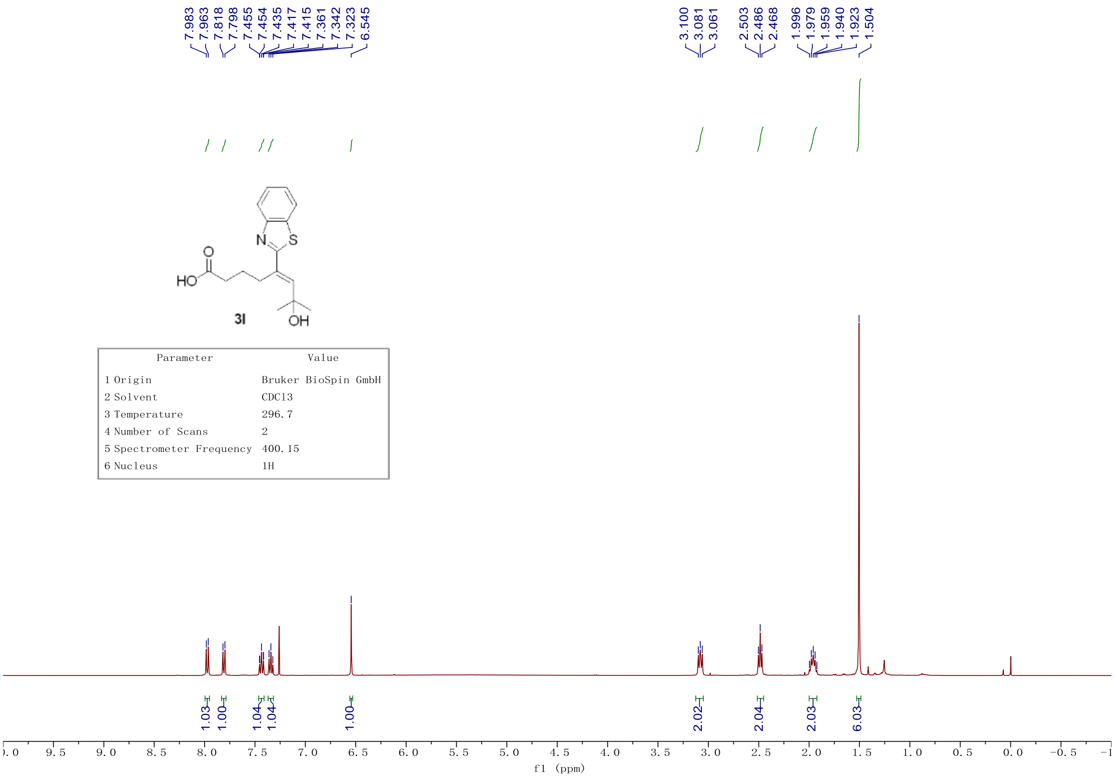

Figure S86. ^{1}H-NMR of **3l.**

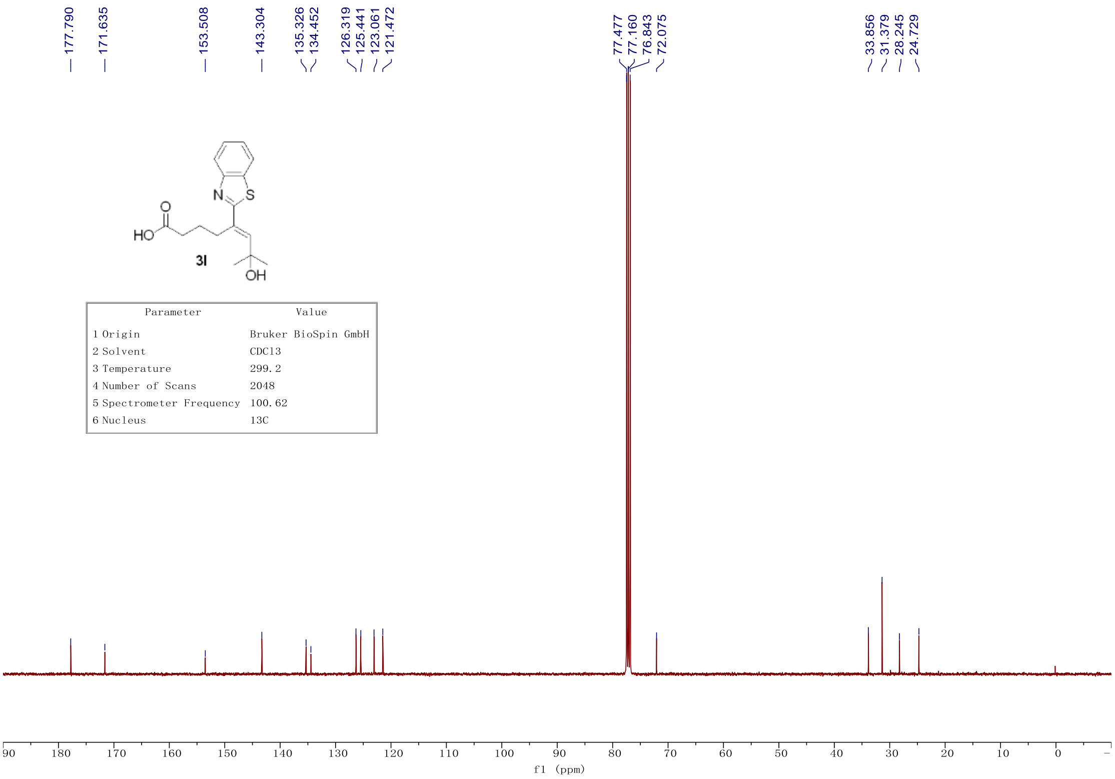

Figure S87. ^{13}C-NMR of **3l.**

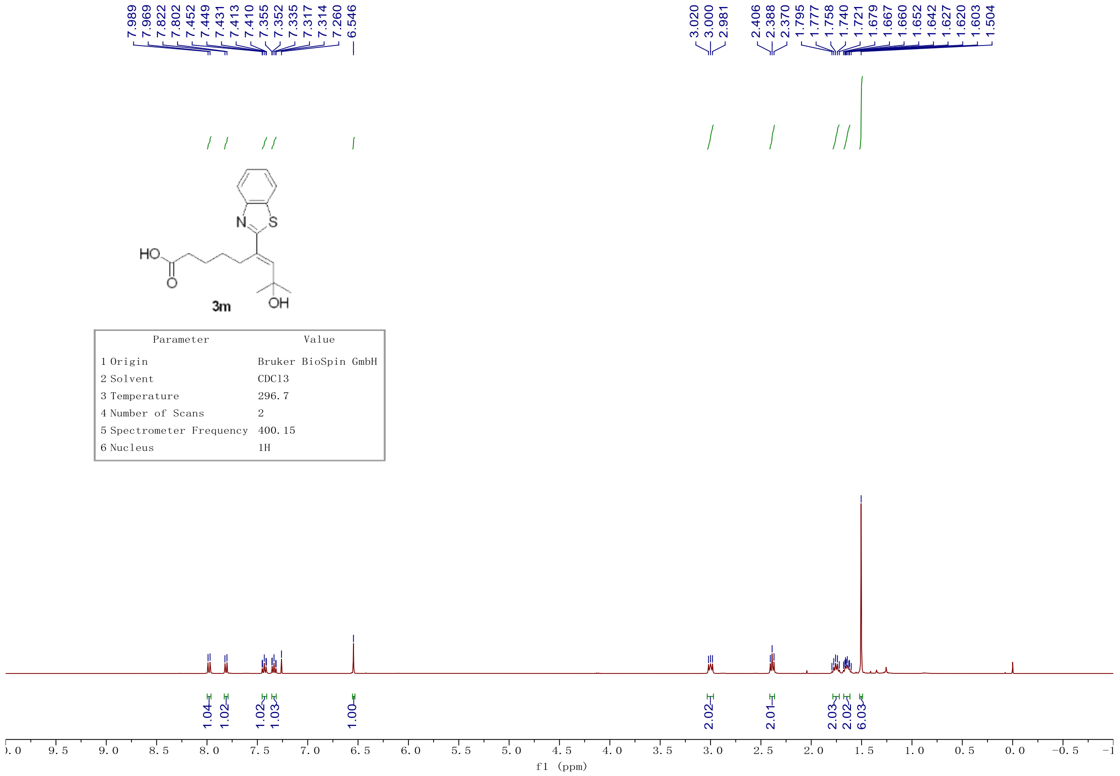

Figure S88. ^{1}H-NMR of **3m.**

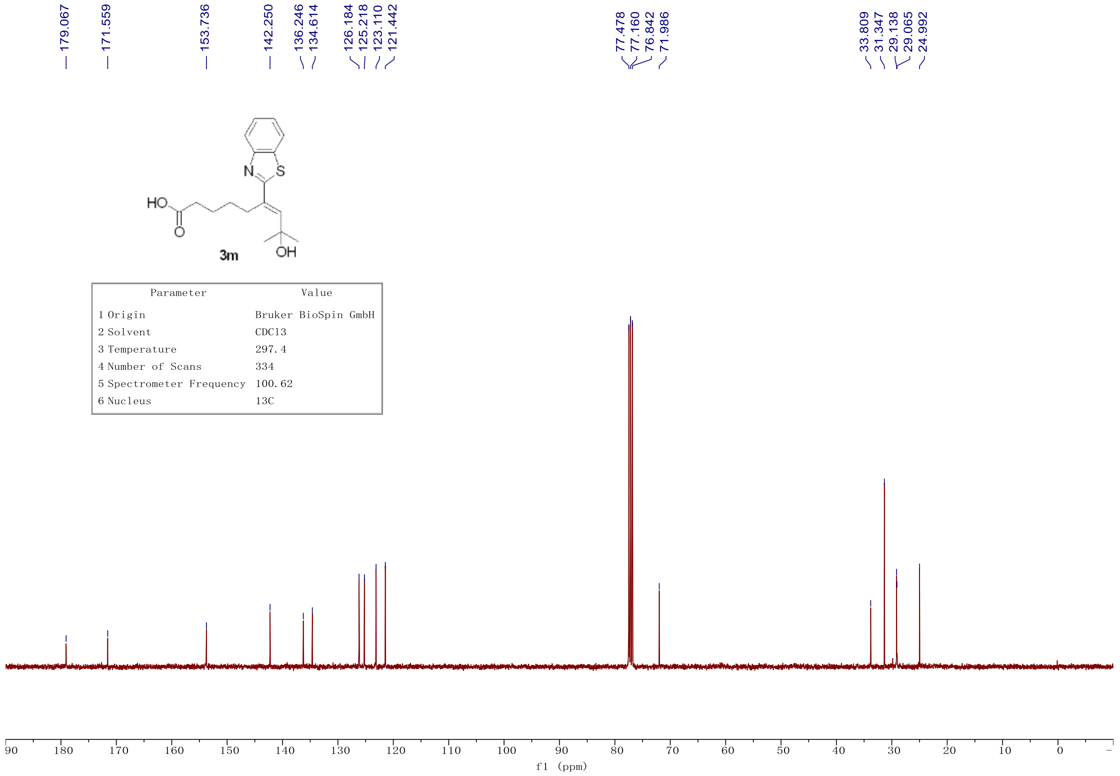

Figure S89. ^{13}C-NMR of **3m.**

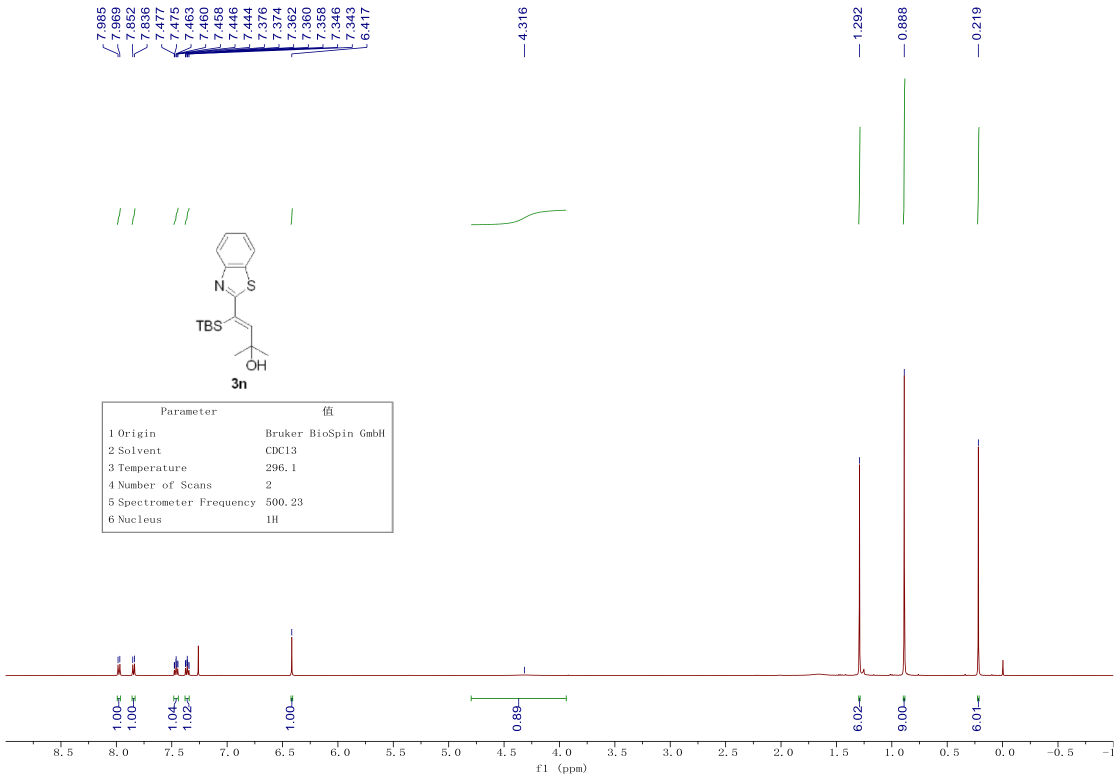

Figure S90. ^{1}H-NMR of **3n.**

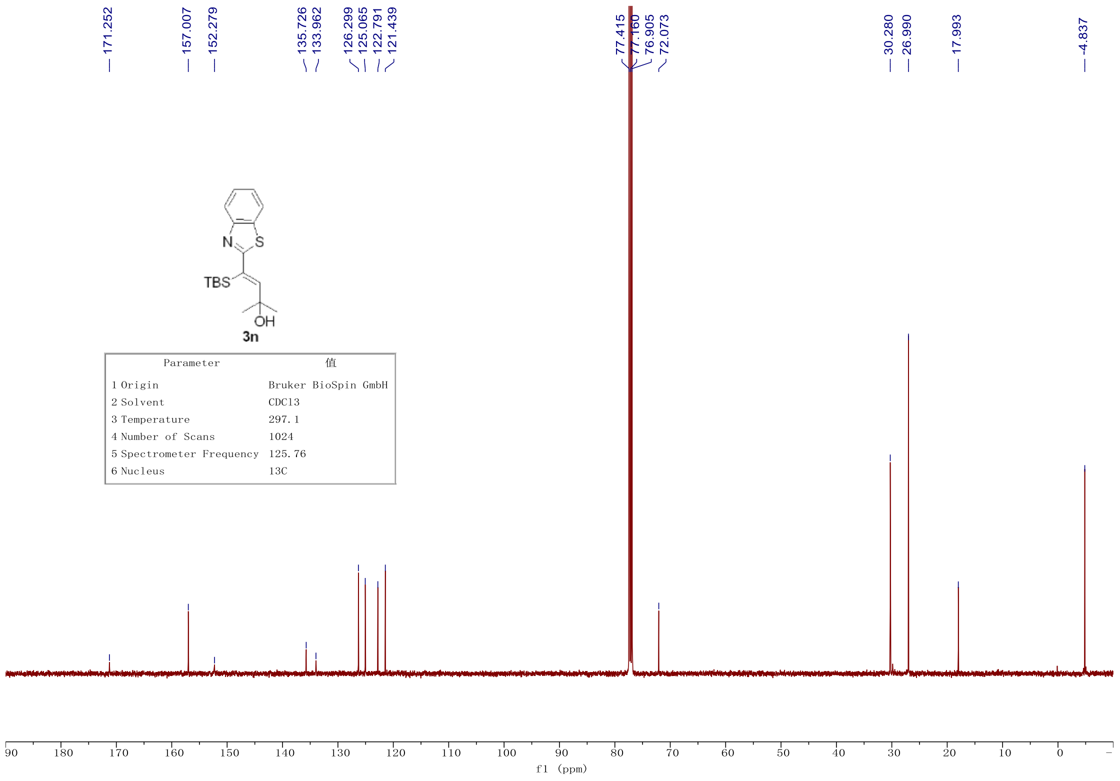

Figure S91. ^{13}C-NMR of **3n.**

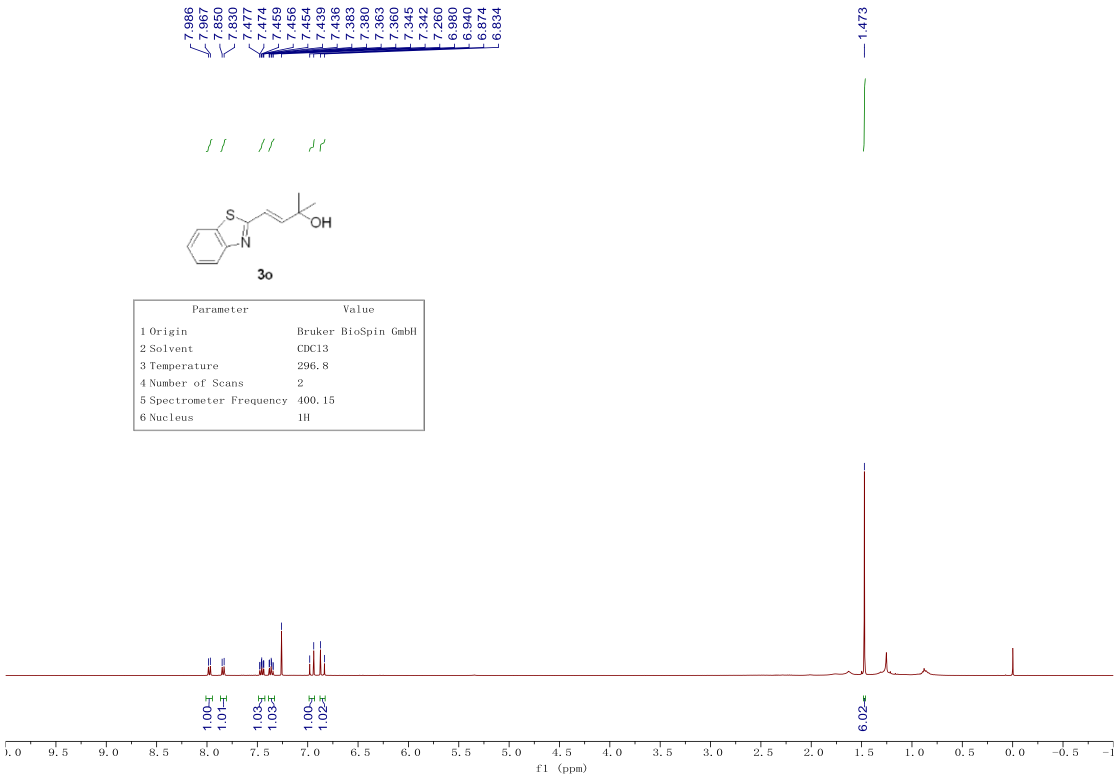

Figure S92. ^{1}H-NMR of **3o.**

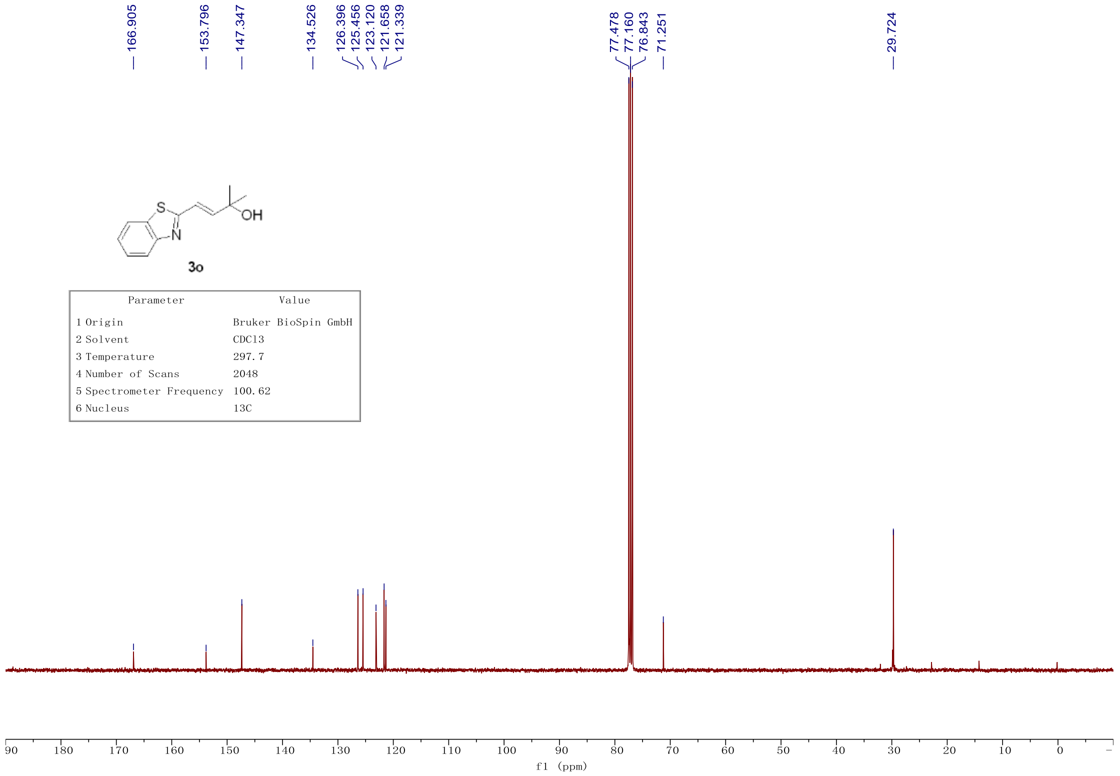

Figure S93. ^{13}C-NMR of **3o.**

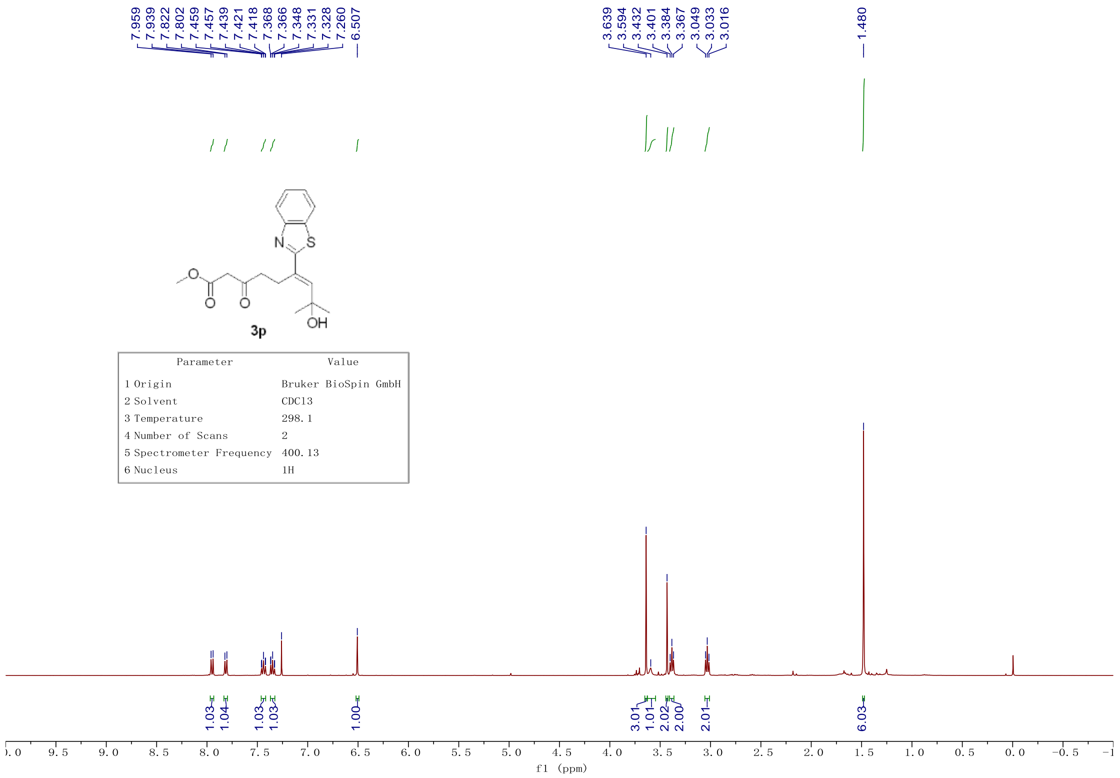

Figure S94. ^{1}H-NMR of **3p.**

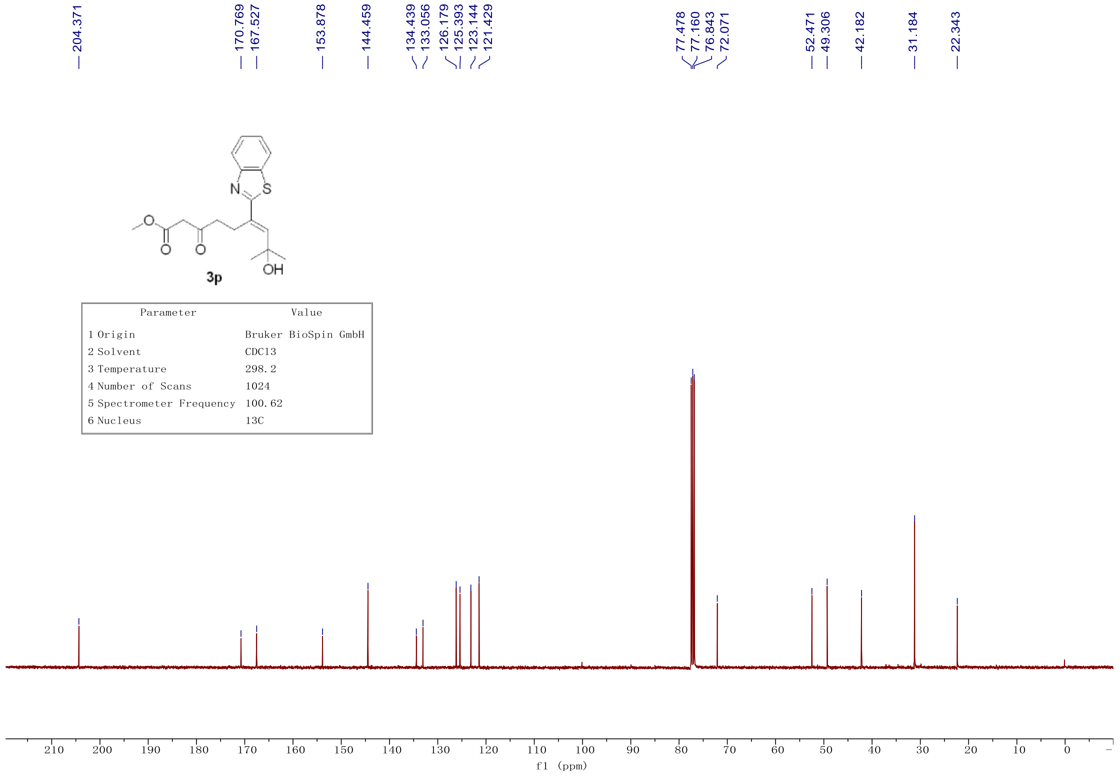

Figure S95. ^{13}C-NMR of **3p.**

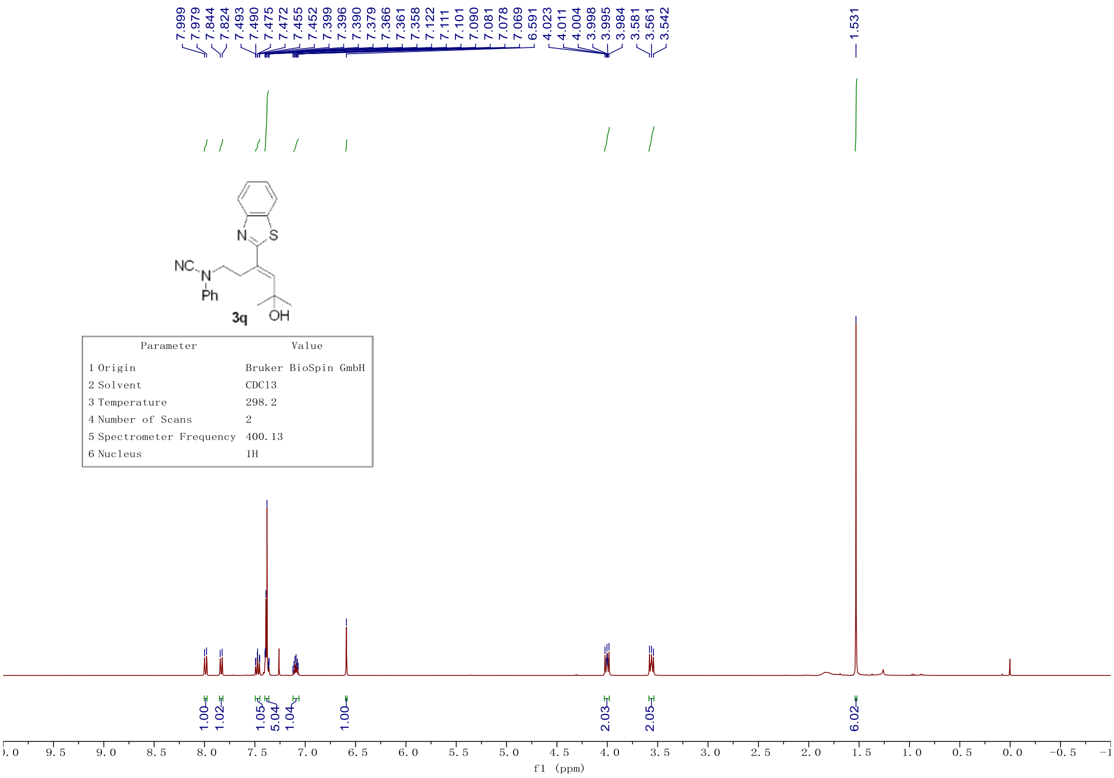

Figure S96. ^{1}H-NMR of **3q.**

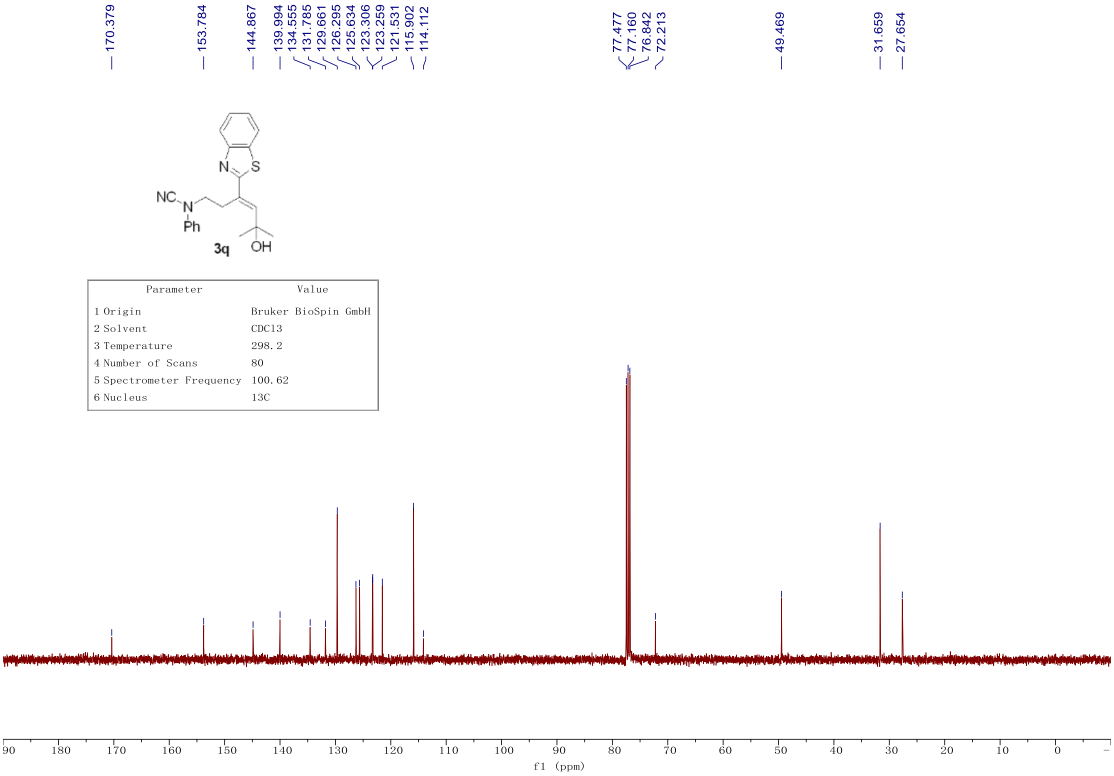

Figure S97. ^{13}C-NMR of **3q.**

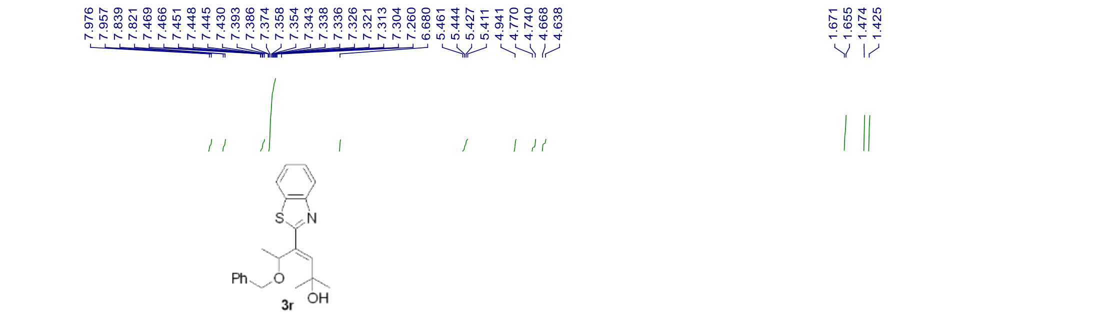

Parameter	Value
1 Origin	Bruker BioSpin GmbH
2 Solvent	CDCl3
3 Temperature	296.9
4 Number of Scans	2
5 Spectrometer Frequency	400.15
6 Nucleus	1H

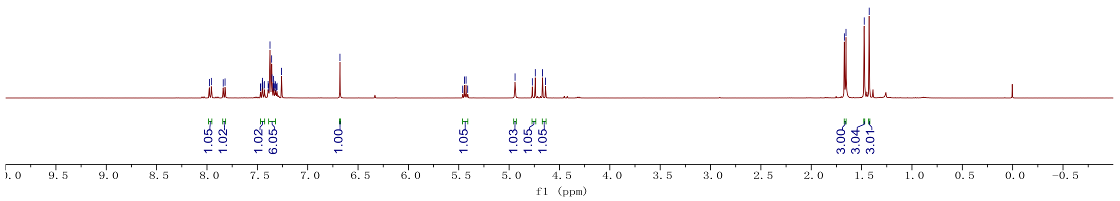

Figure S98. ^{1}H-NMR of **3r.**

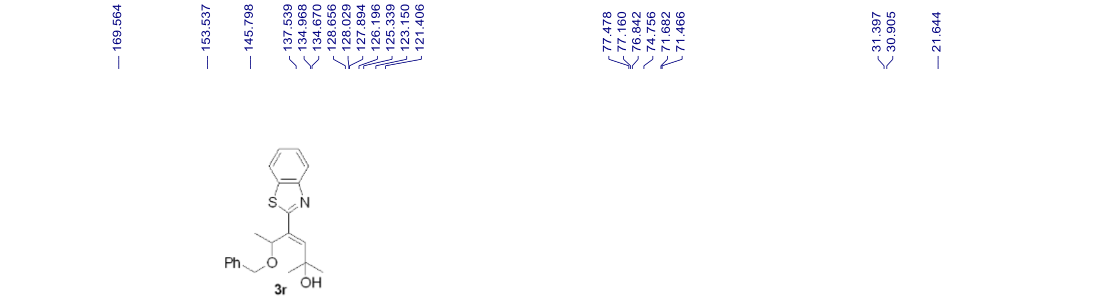

Parameter	Value
1 Origin	Bruker BioSpin GmbH
2 Solvent	CDCl3
3 Temperature	298.2
4 Number of Scans	2048
5 Spectrometer Frequency	100.61
6 Nucleus	13C

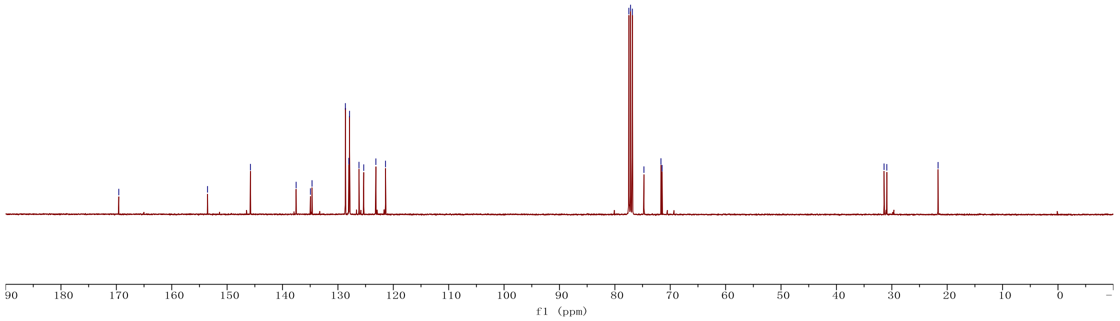

Figure S99. ^{13}C-NMR of **3r.**

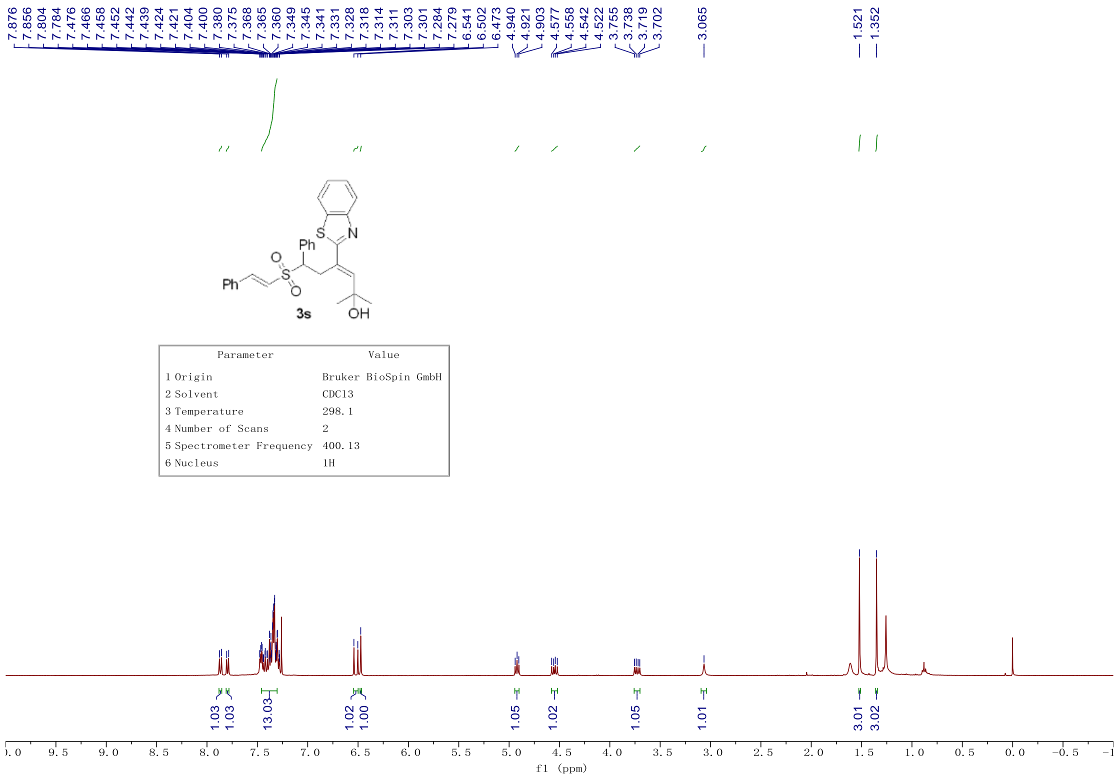

Figure S100. ^{1}H-NMR of **3s.**

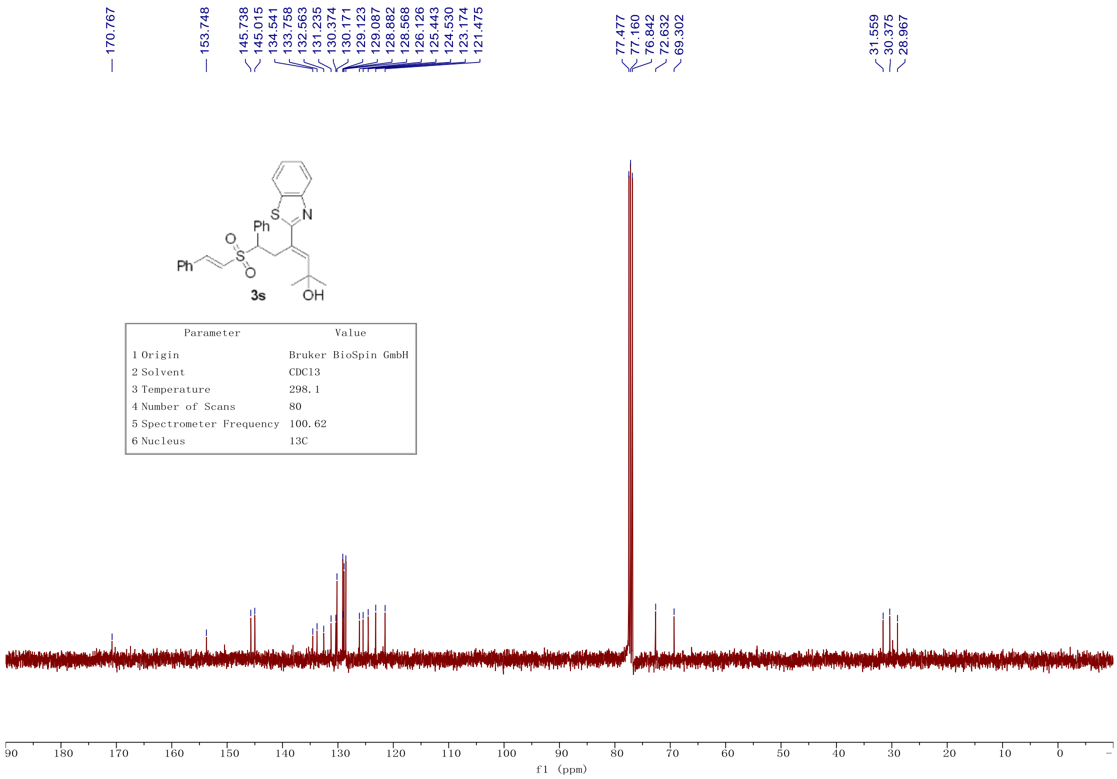

Figure S101. ^{13}C-NMR of **3s.**

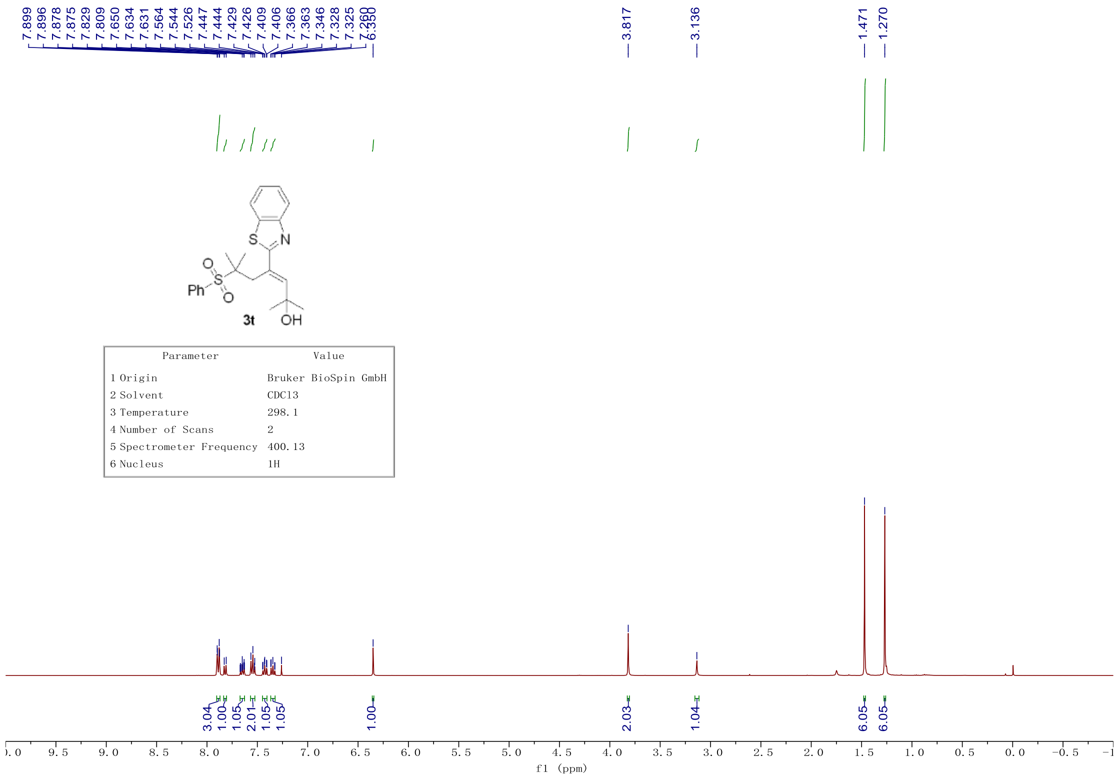

Figure S102. ^{1}H-NMR of **3t.**

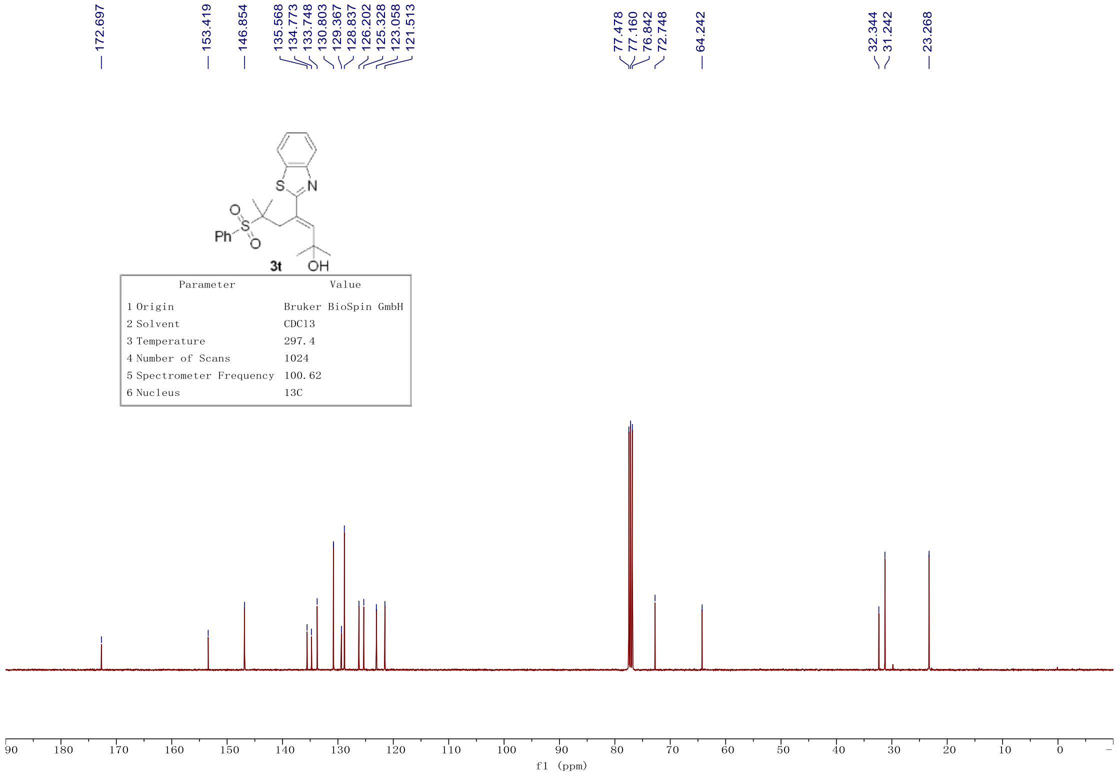

Figure S103. ^{13}C-NMR of **3t.**

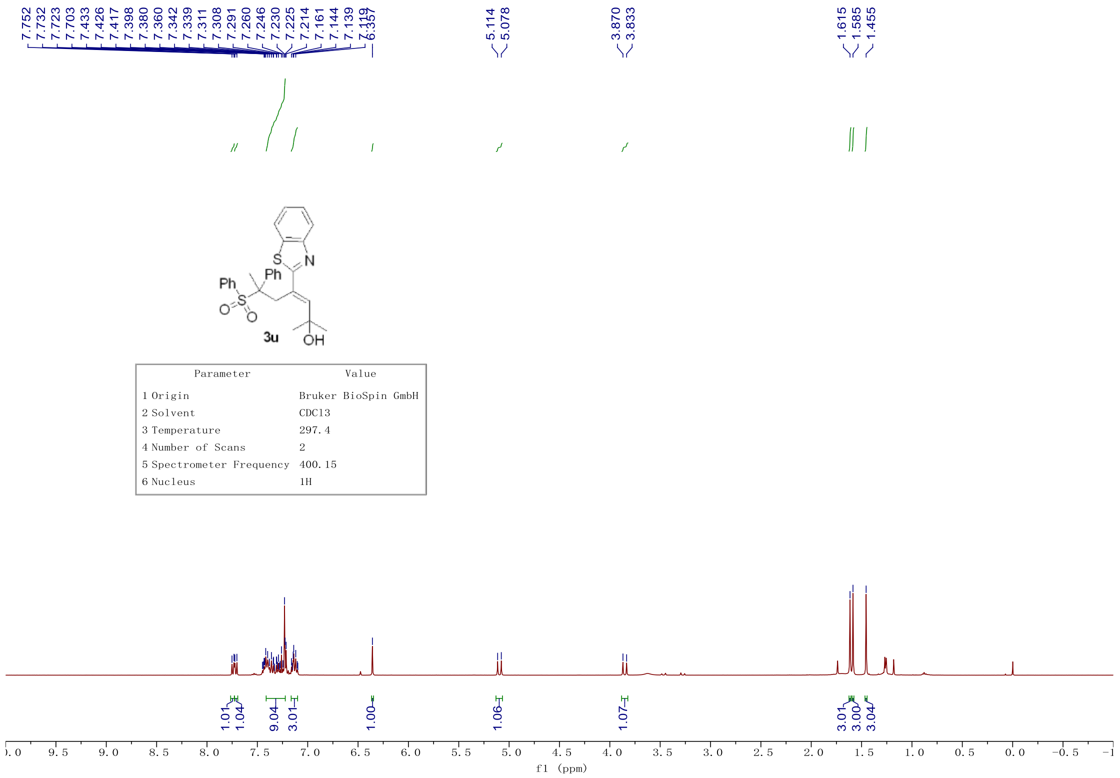

Figure S104. ^{1}H-NMR of **3u.**

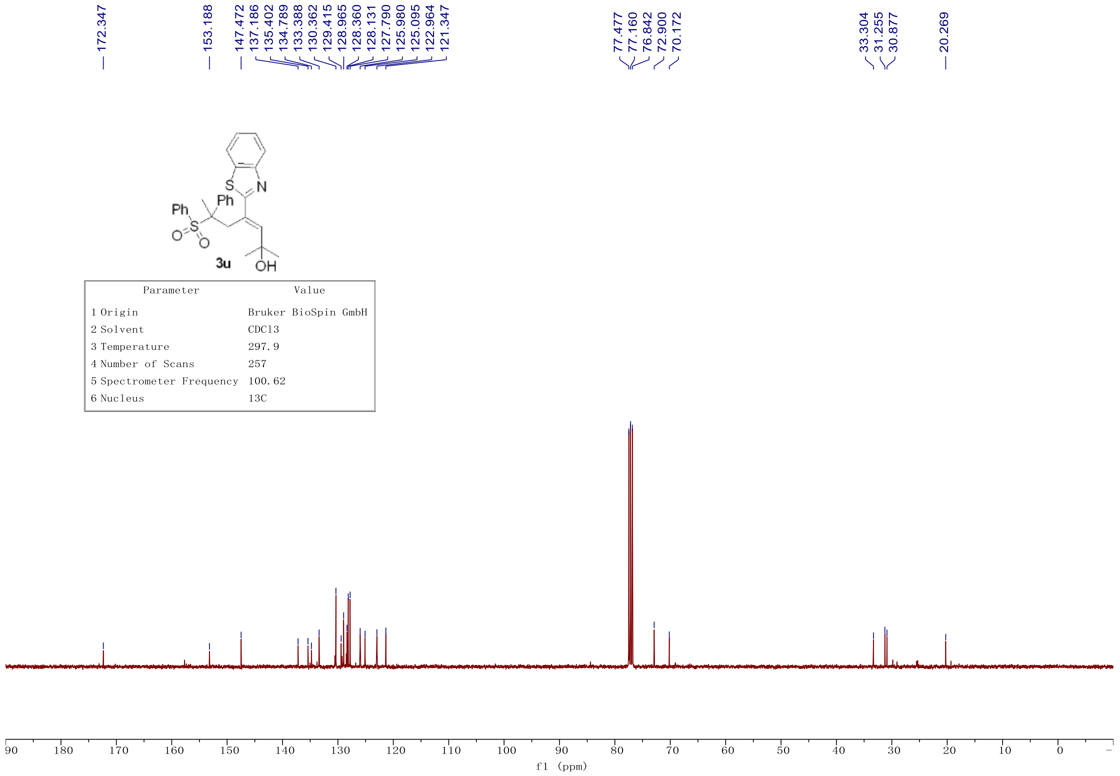

Figure S105. ^{13}C-NMR of **3u.**

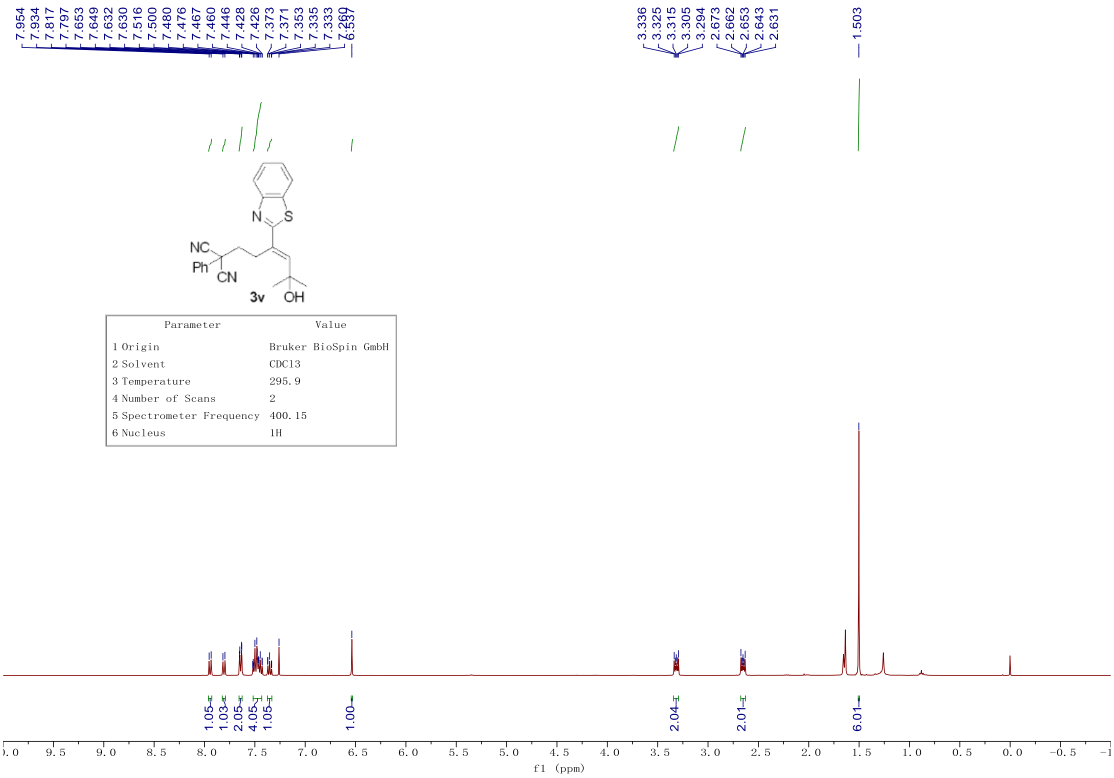

Figure S106. ^{1}H-NMR of **3v.**

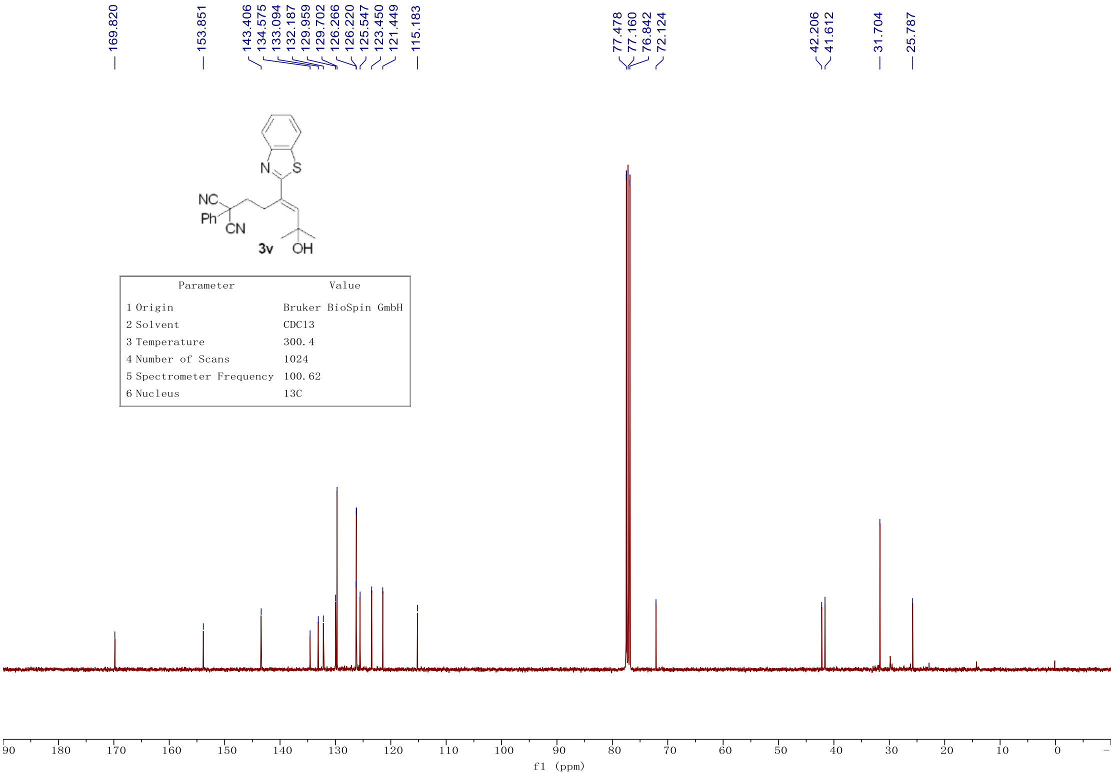

Figure S107. ^{13}C-NMR of **3v.**

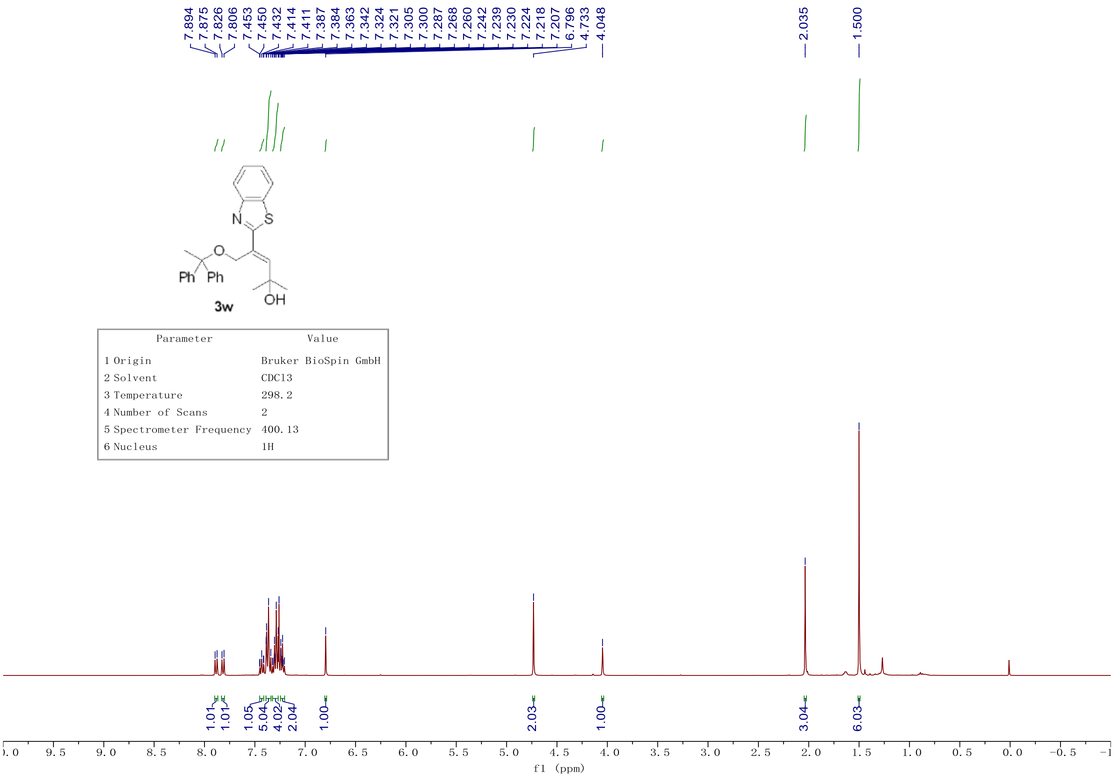

Figure S108. ^{1}H-NMR of **3w.**

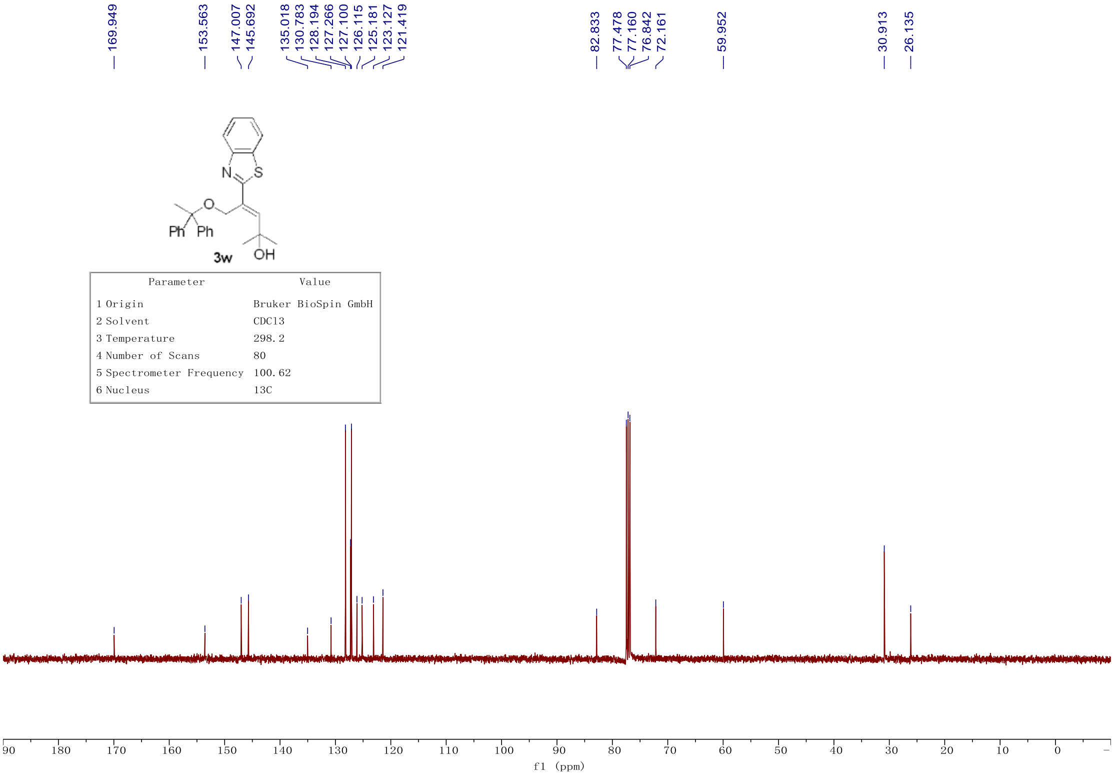

Figure S109. ^{13}C-NMR of **3w.**

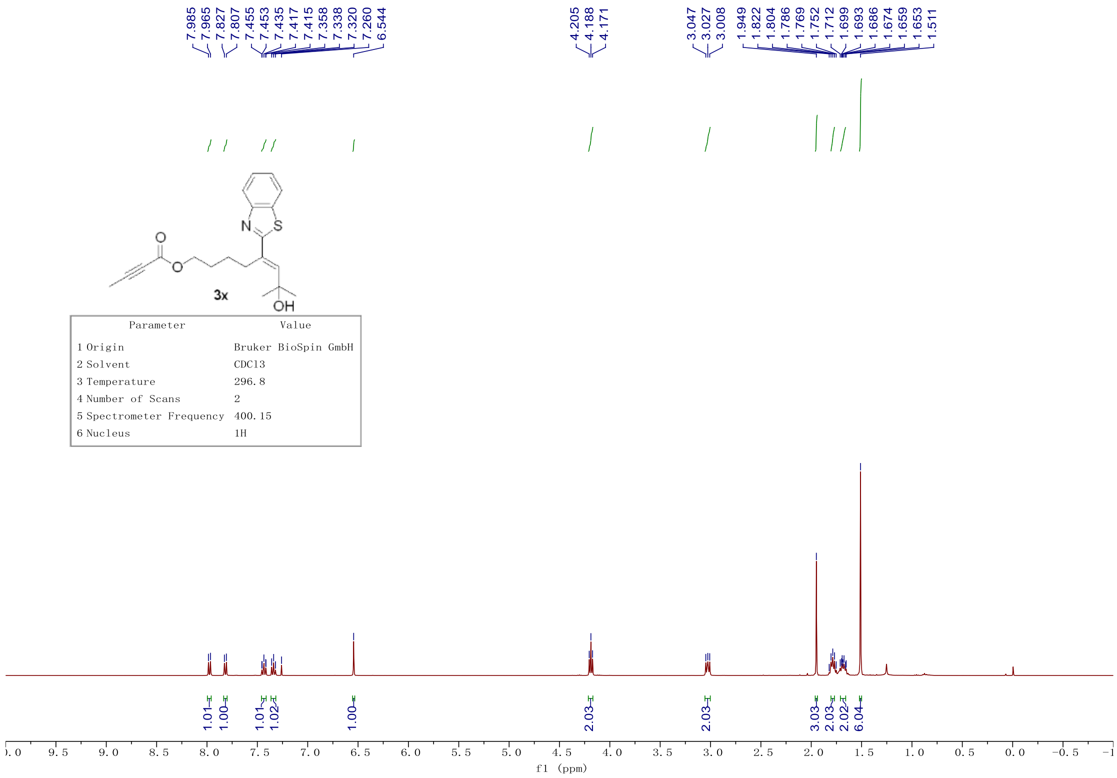

Figure S110. ^{1}H-NMR of **3x.**

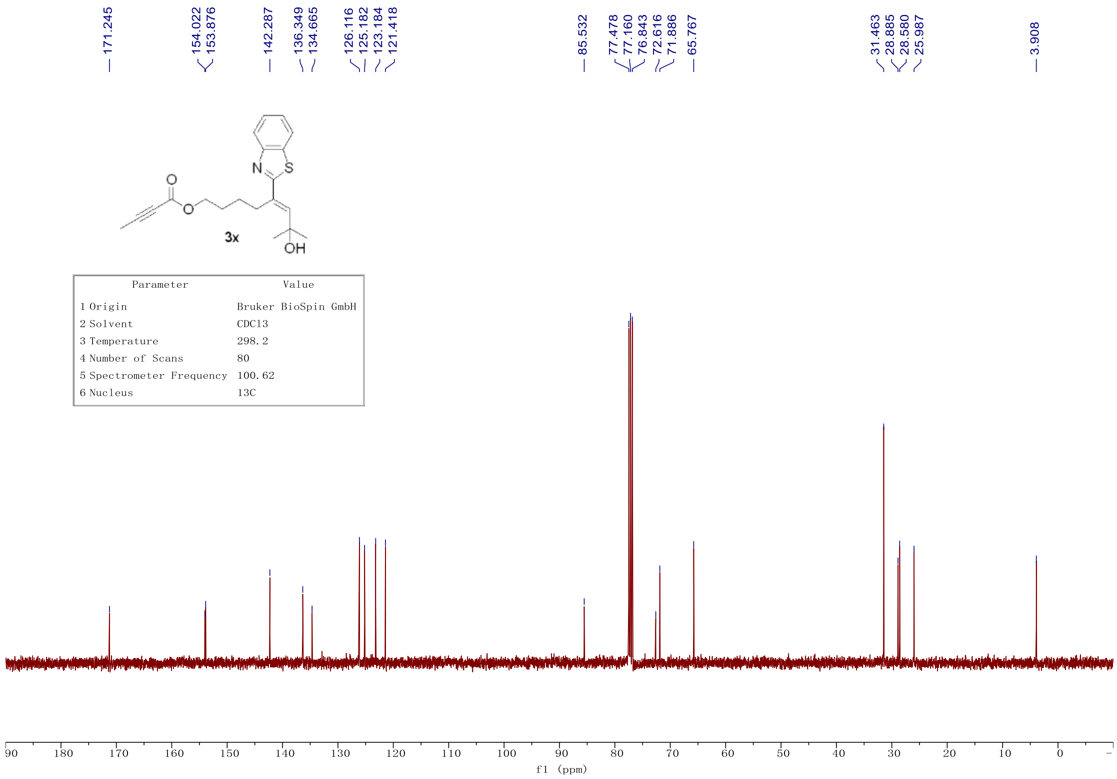

Figure S111. ^{13}C-NMR of **3x.**

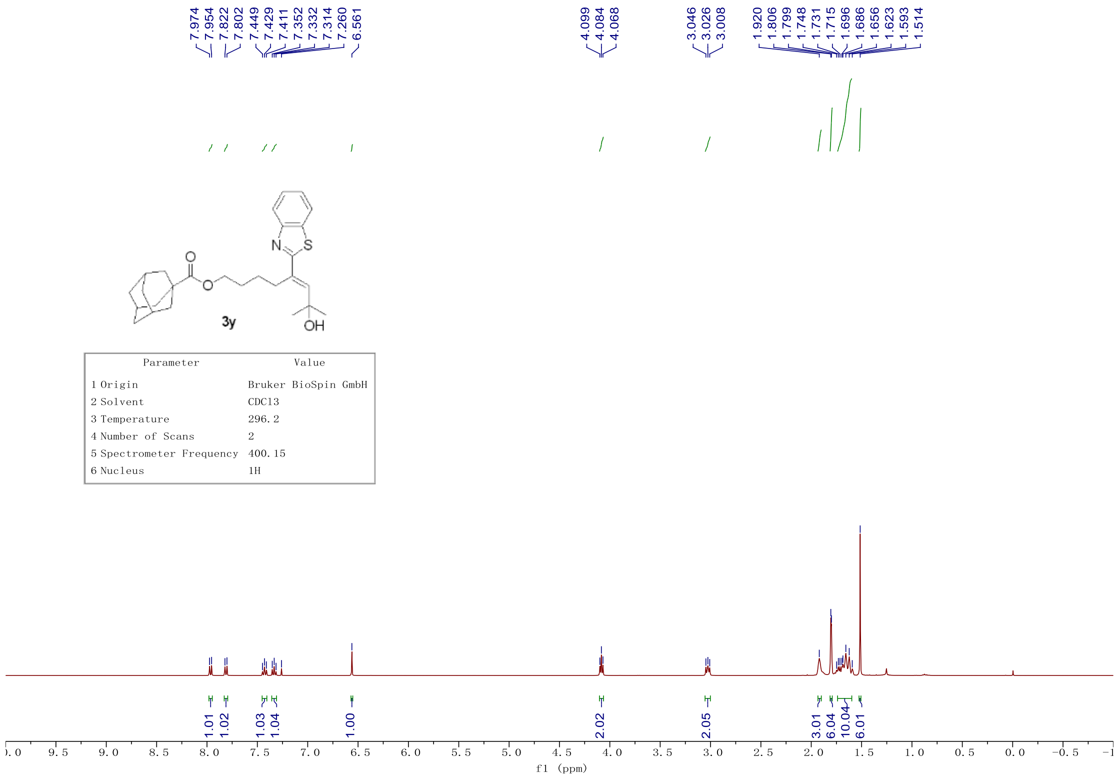

Figure S112. ^{1}H-NMR of **3y.**

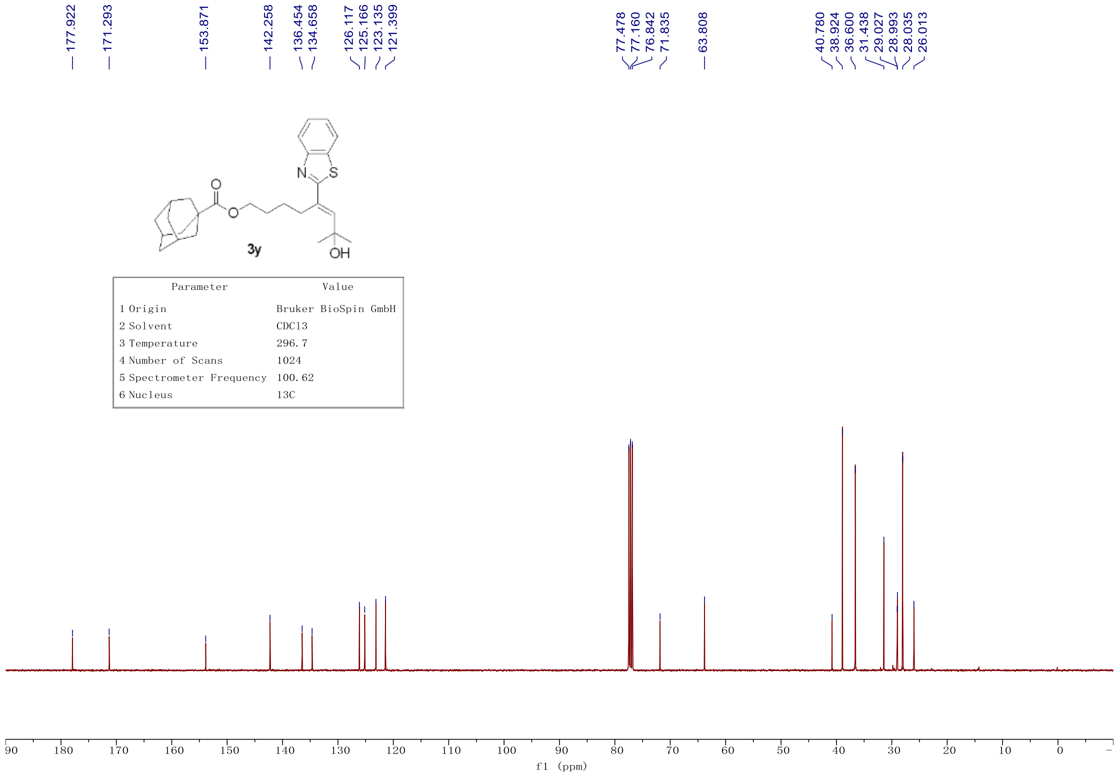

Figure S113. ^{13}C-NMR of **3y.**

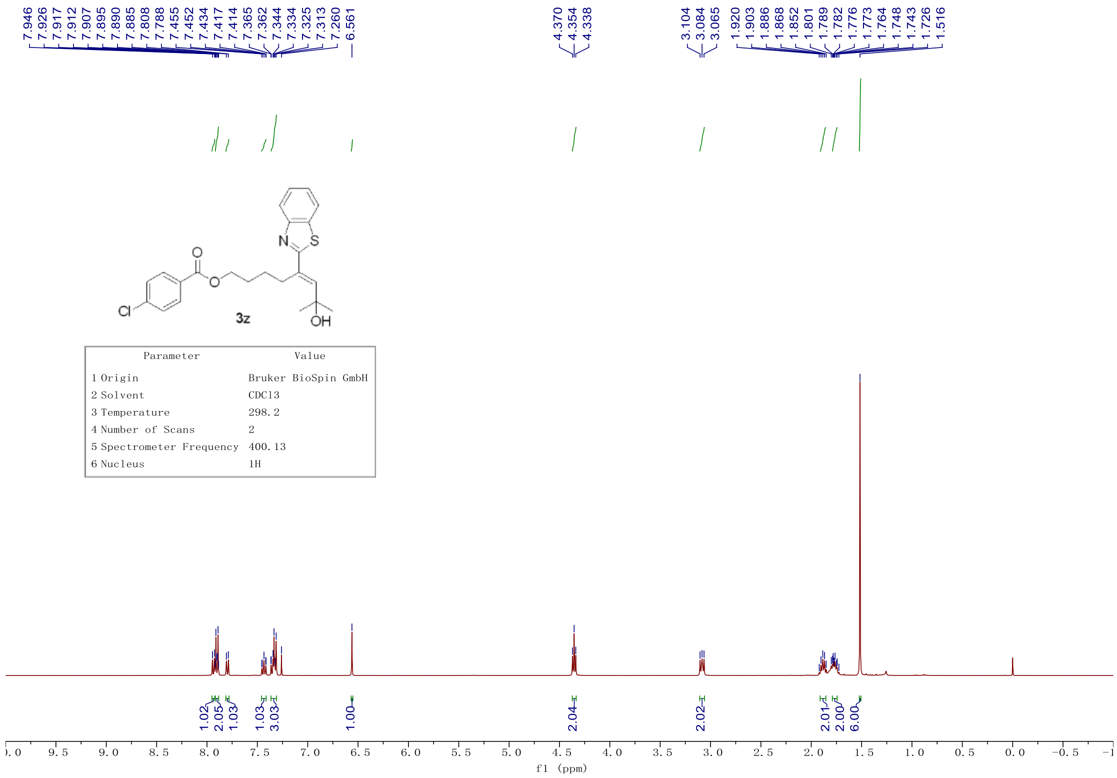

Figure S114. ^{1}H-NMR of **3z.**

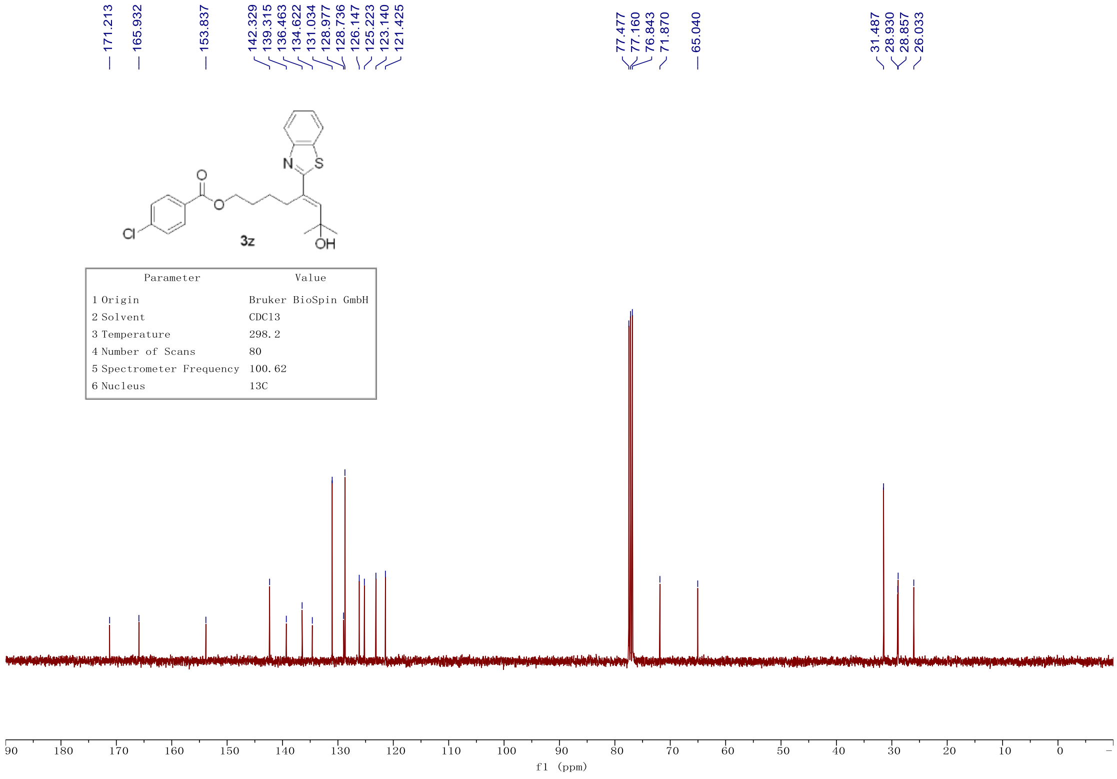

Figure S115. ^{13}C-NMR of **3z.**

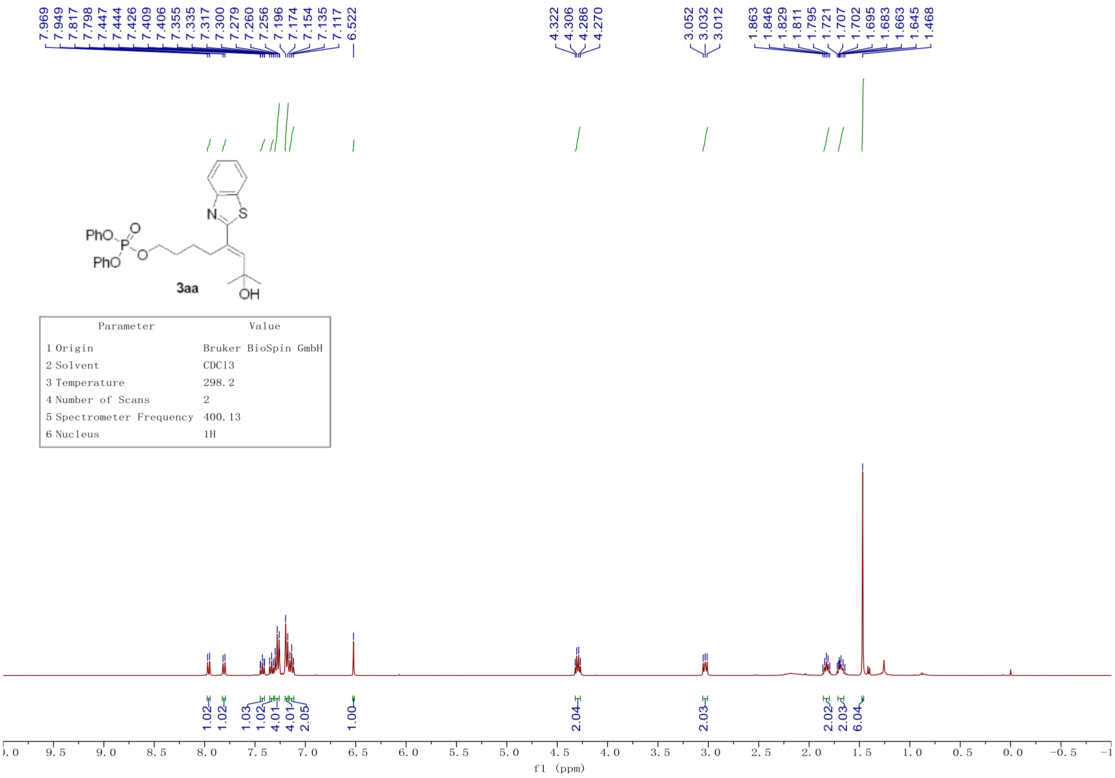

Figure S116. ^{1}H-NMR of **3aa.**

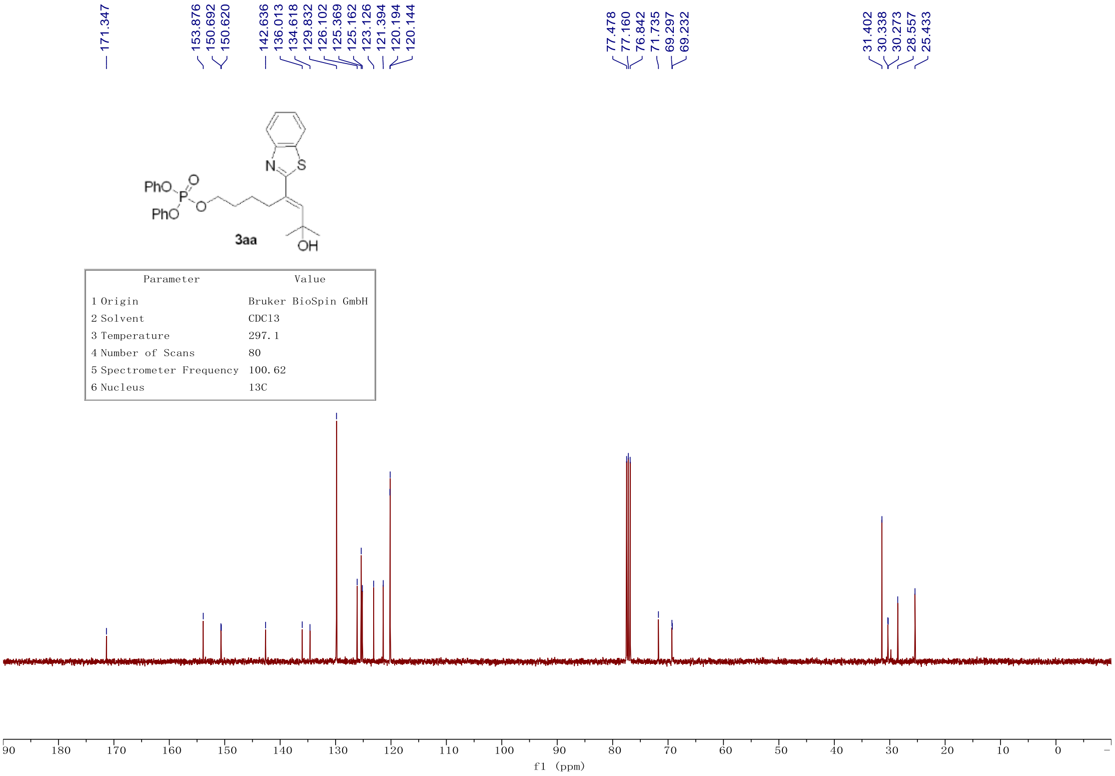

Figure S117. ^{13}C-NMR of **3aa.**

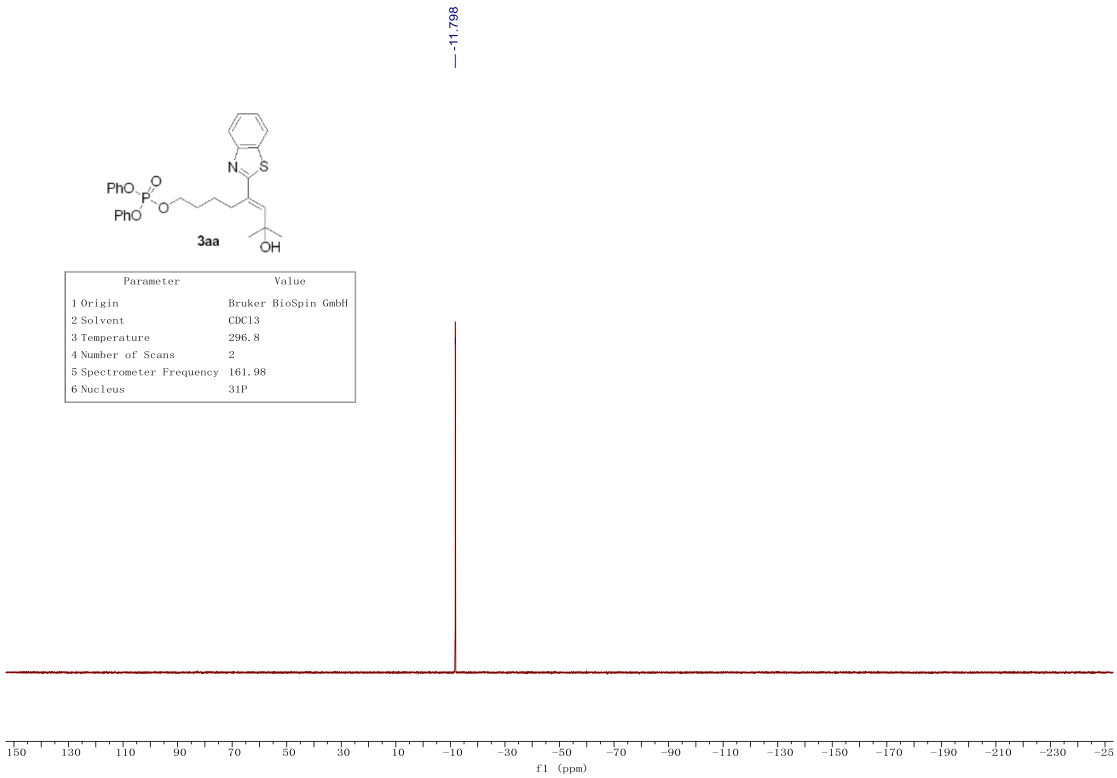

Figure S118. ^{32}P-NMR of **3aa.**

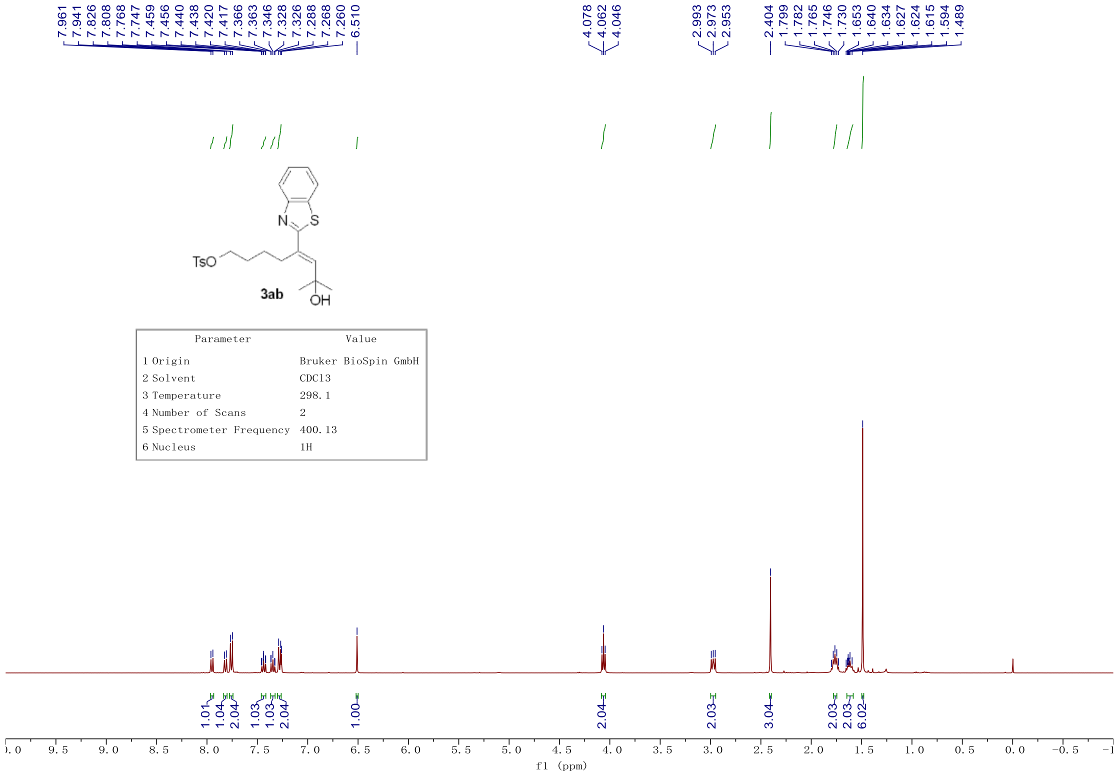

Figure S119. ^{1}H-NMR of **3ab.**

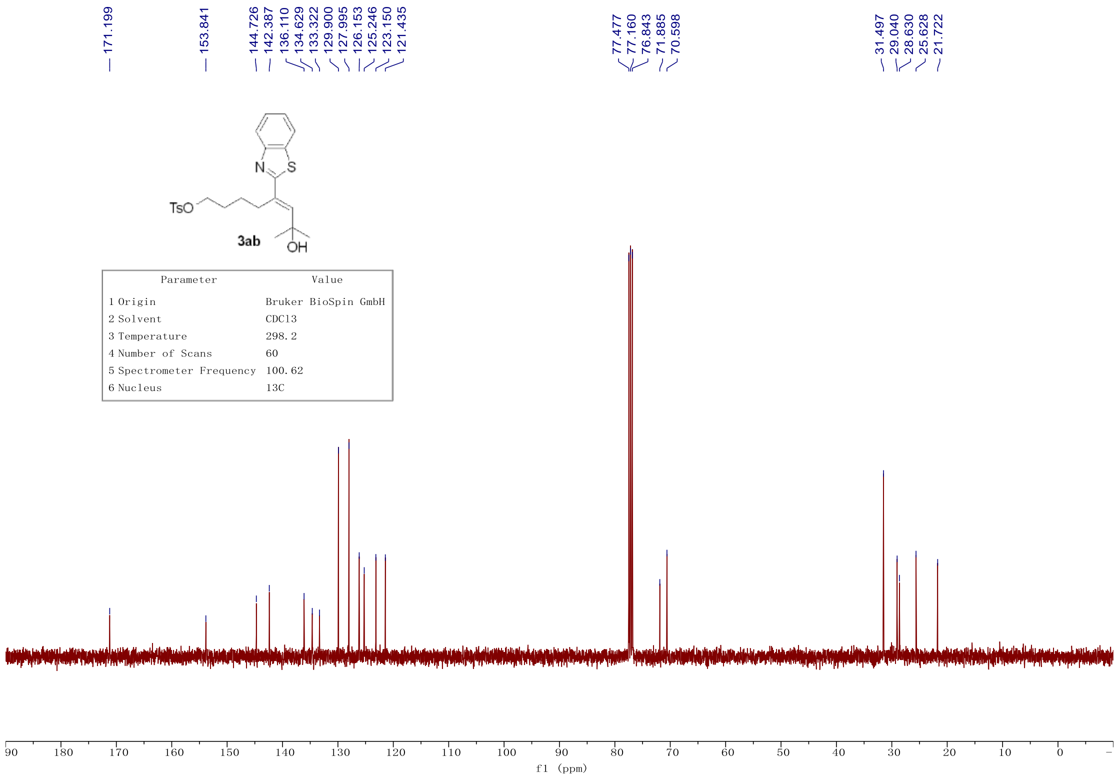

Figure S120. ^{13}C-NMR of **3ab.**

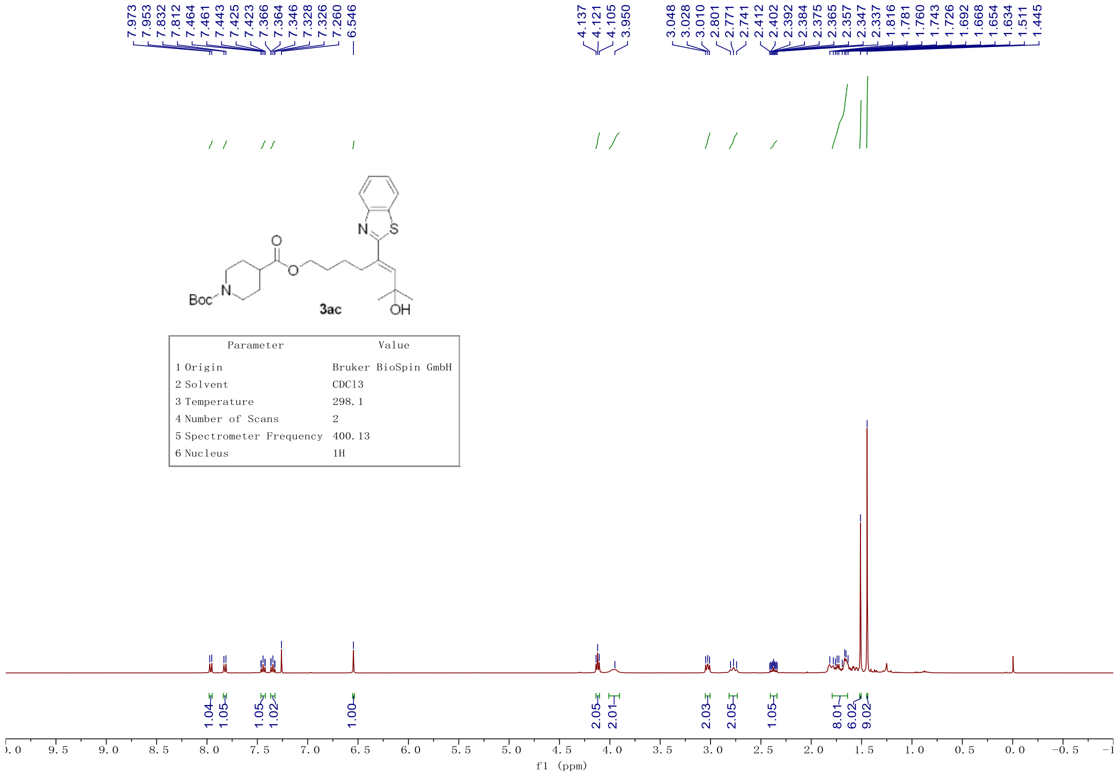

Figure S121. ^{1}H-NMR of **3ac.**

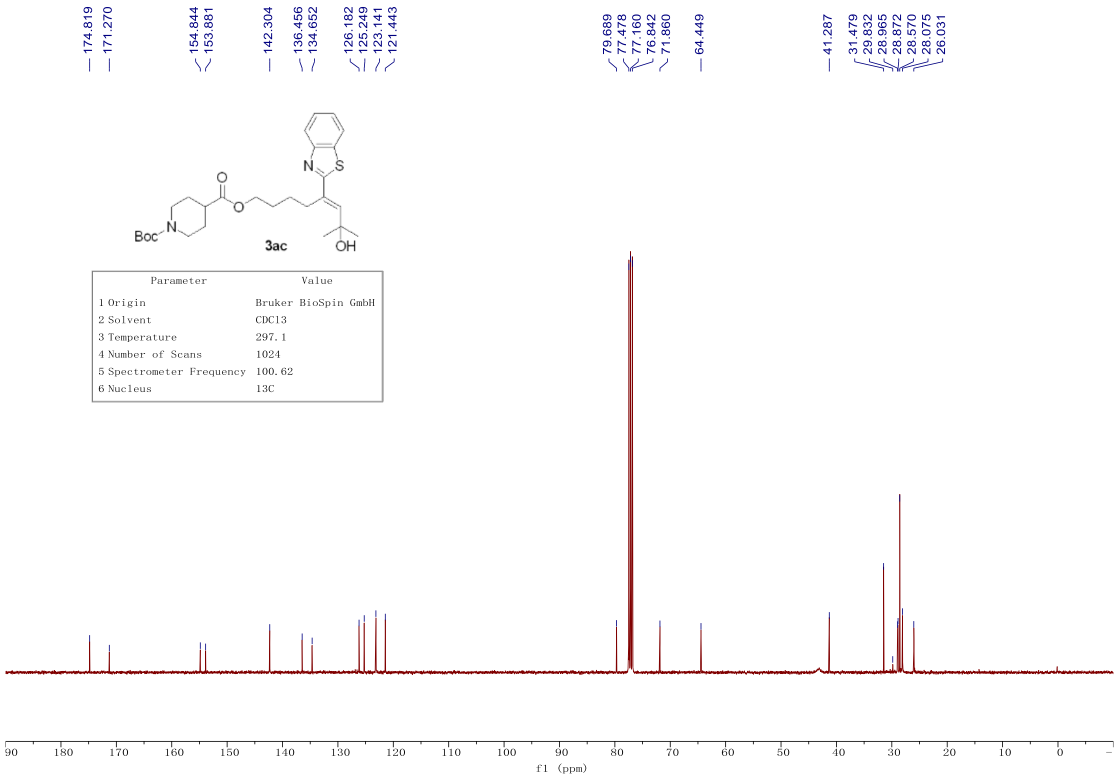

Figure S122. ^{13}C-NMR of **3ac.**

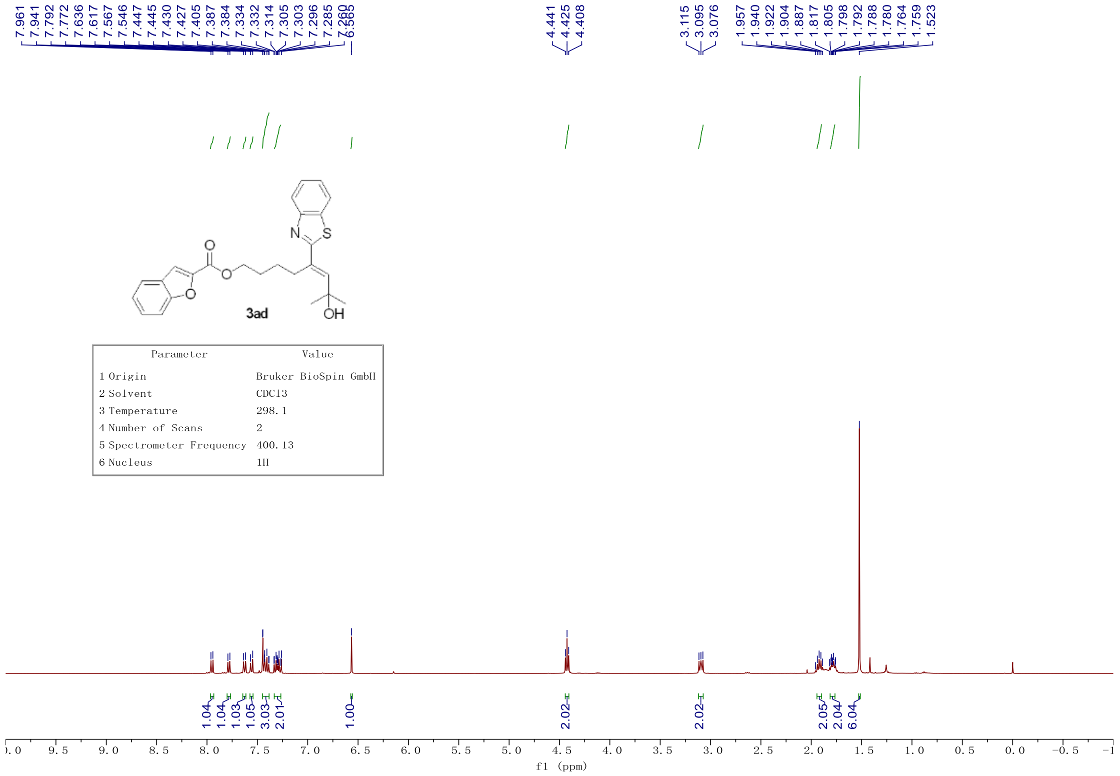

Figure S123. ^{1}H-NMR of **3ad.**

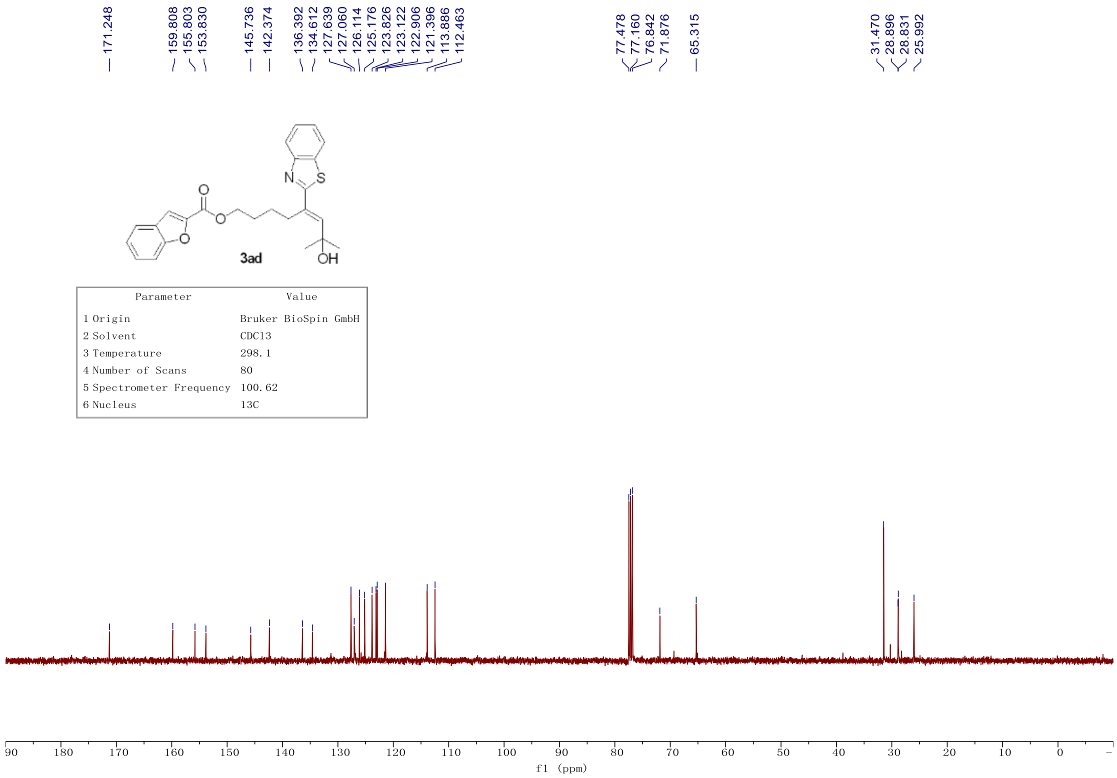

Figure S124. ^{13}C-NMR of **3ad.**

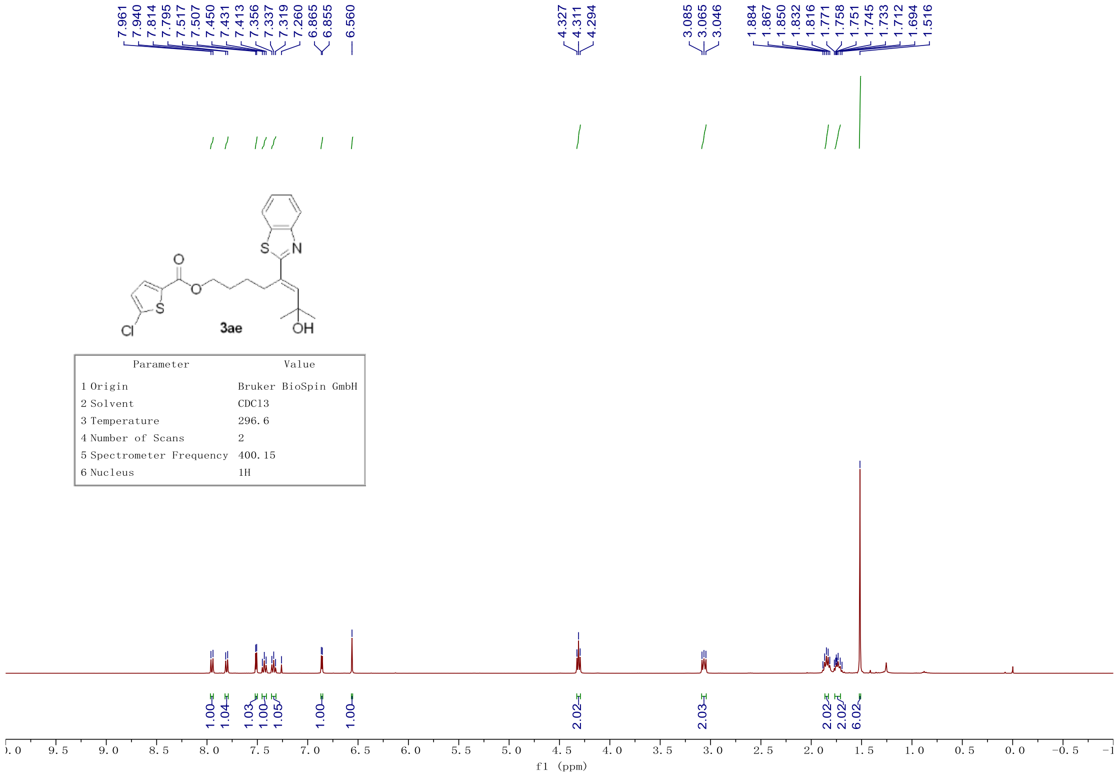

Figure S125. ^{1}H-NMR of **3ae.**

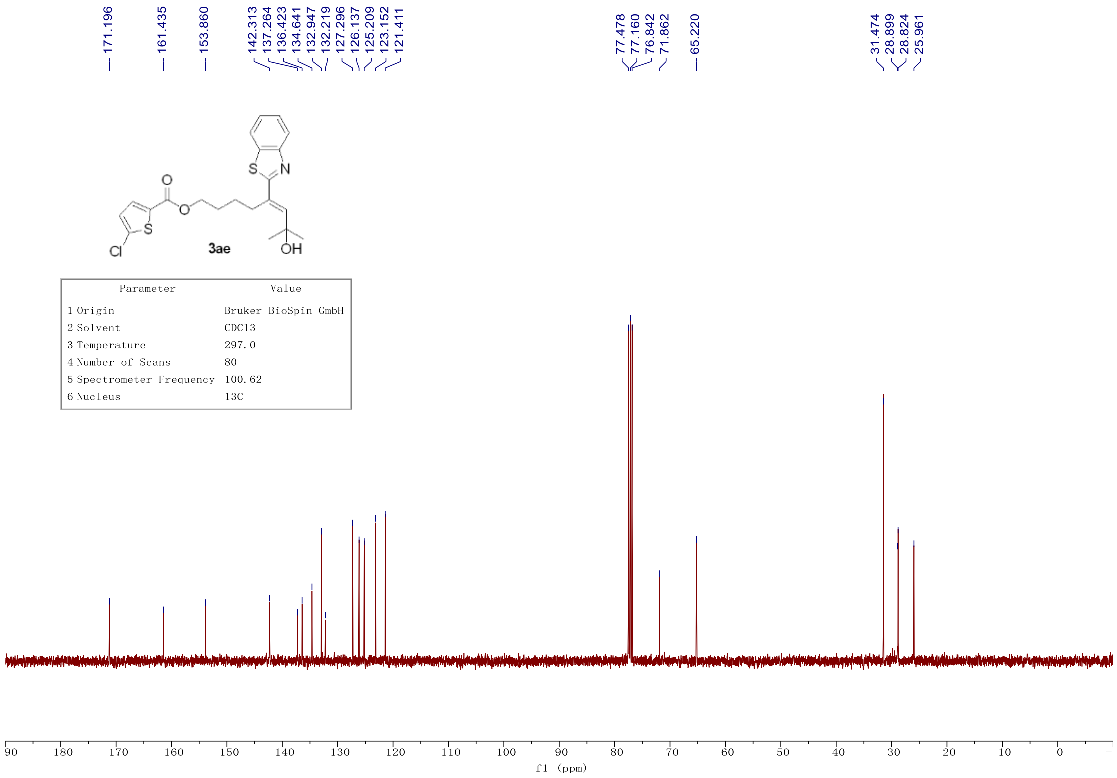

Figure S126. ^{13}C-NMR of **3ae.**

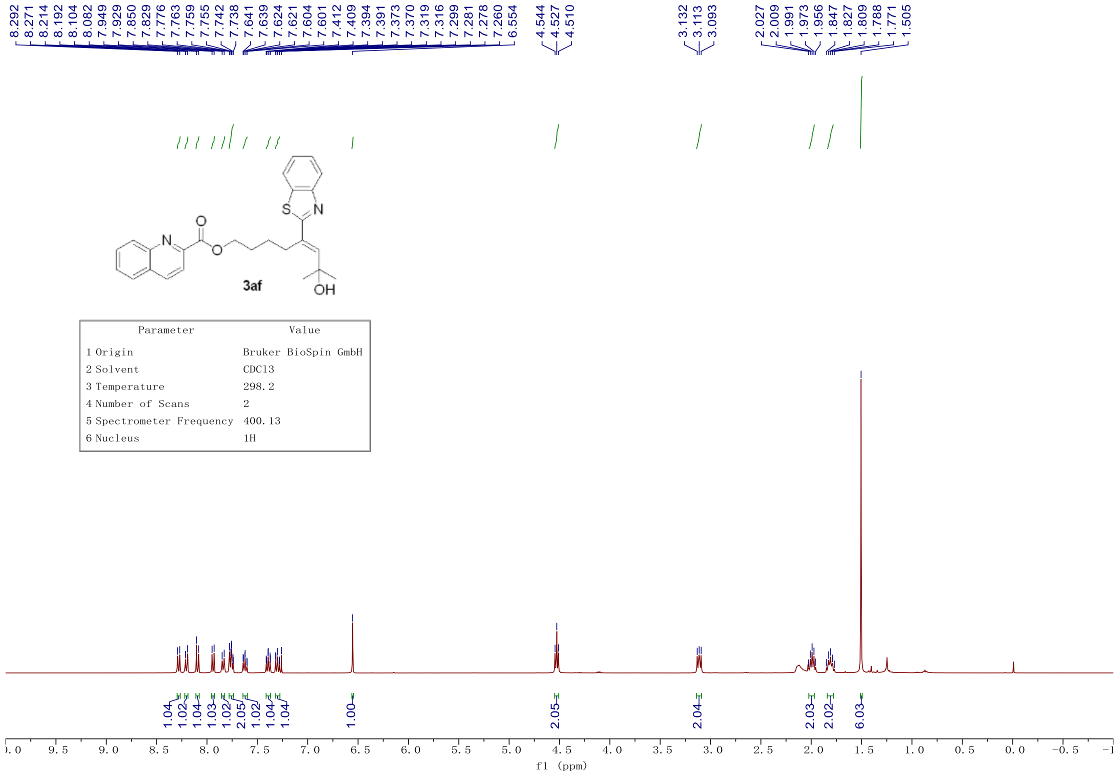

Figure S127. ^{1}H-NMR of **3af.**

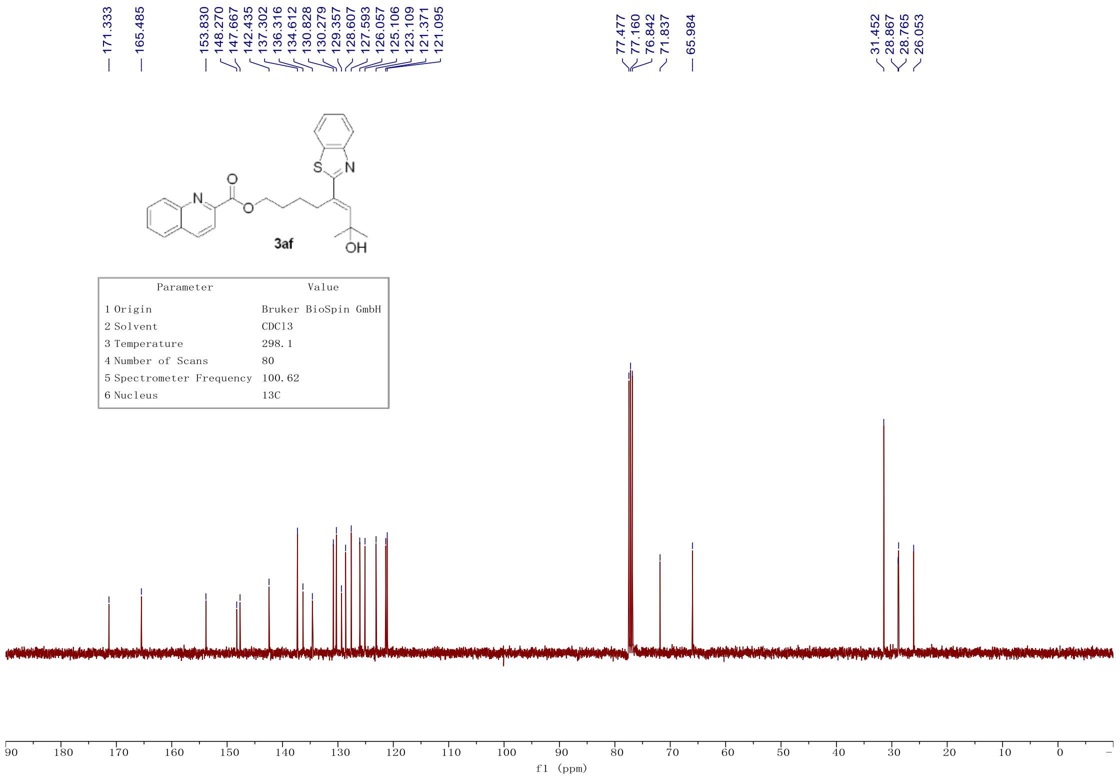

Figure S128. ^{13}C-NMR of **3af.**

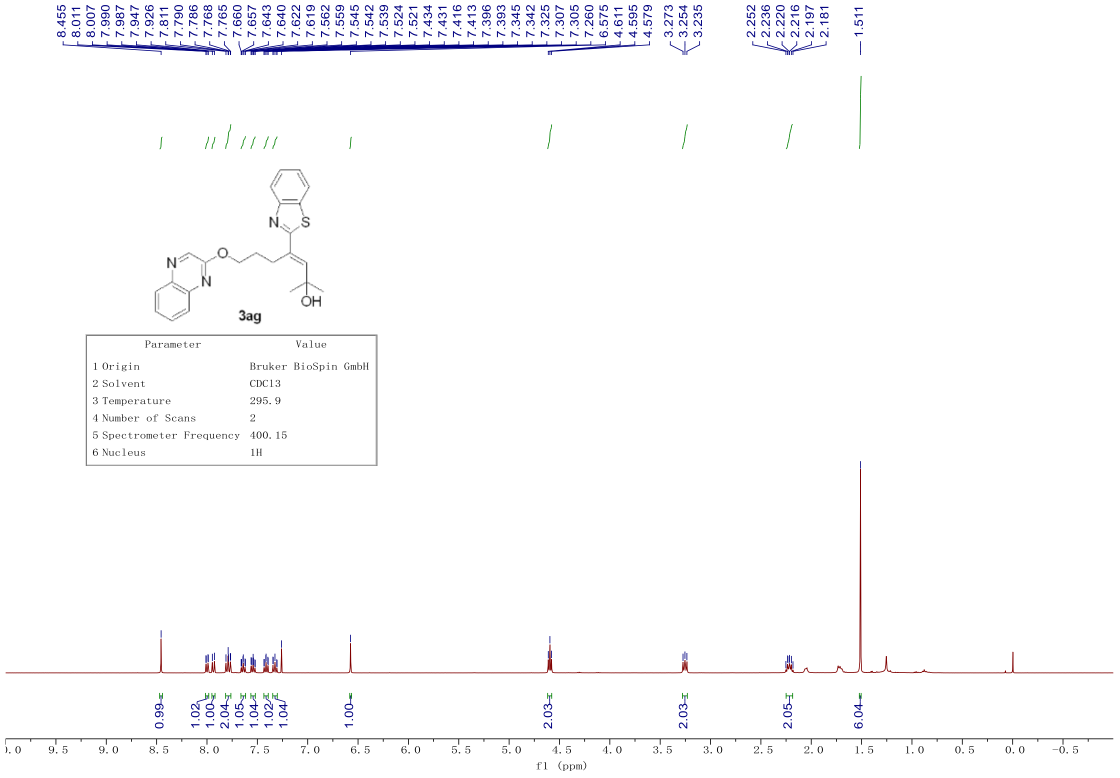

Figure S129. ^{1}H-NMR of **3ag.**

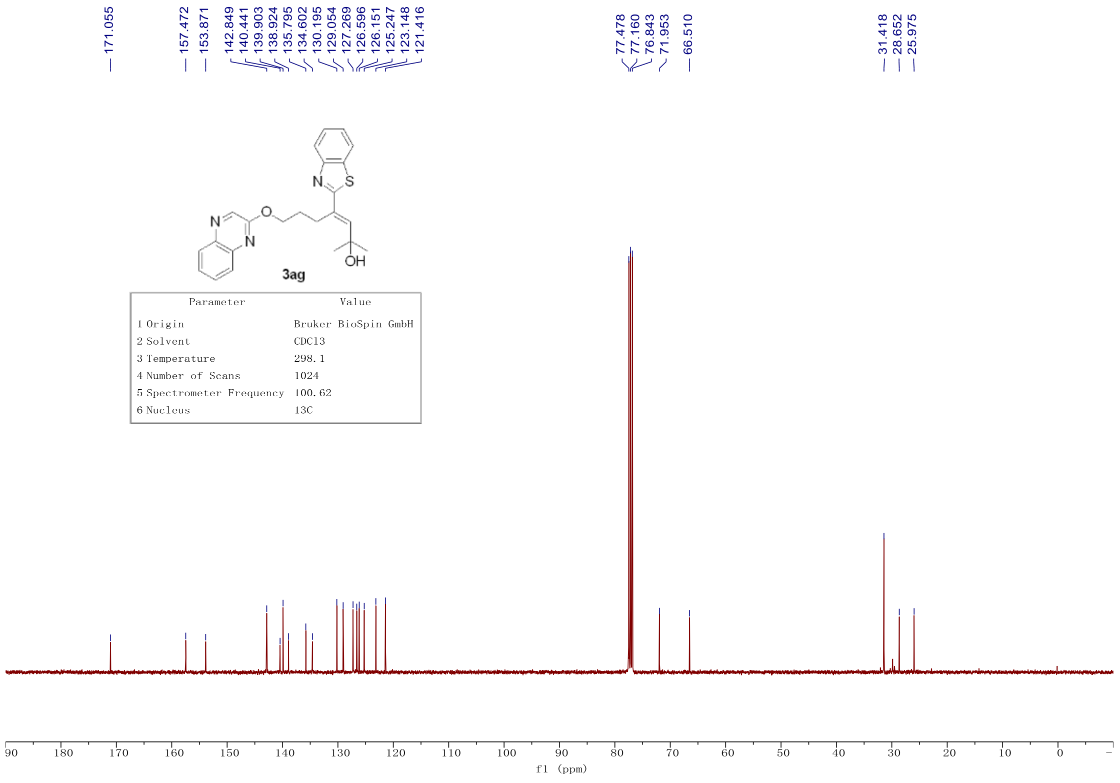

Figure S130. ^{13}C-NMR of **3ag.**

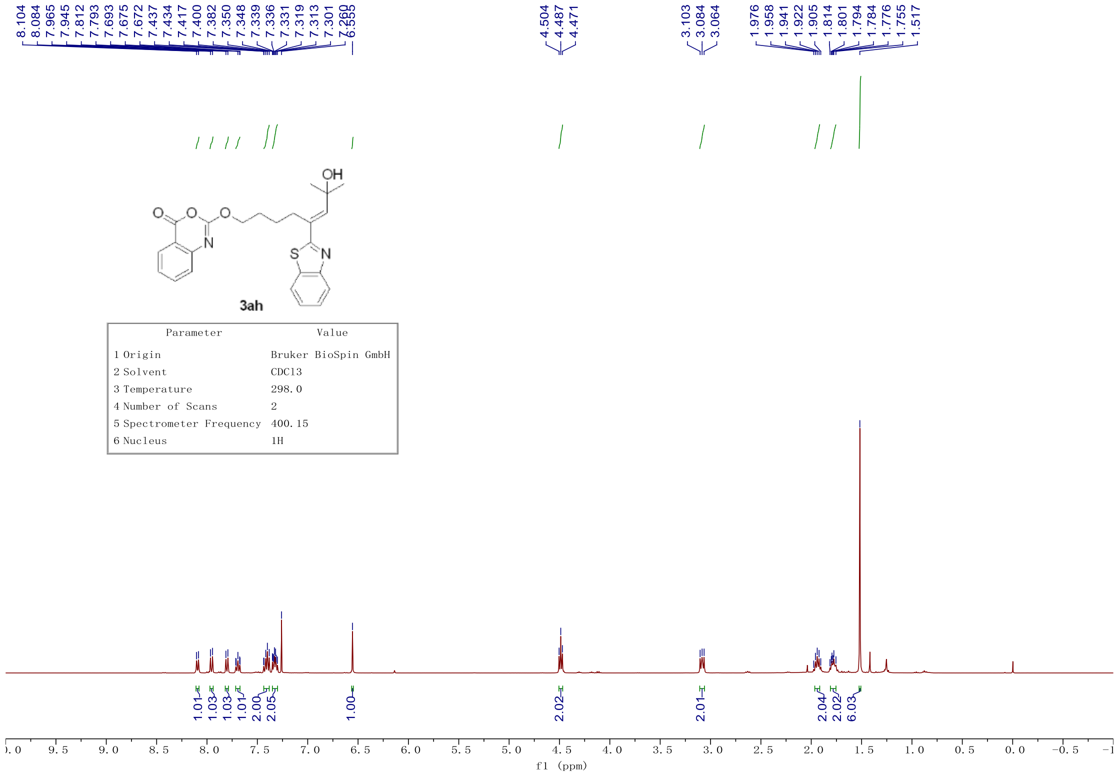

Figure S131. ^{1}H-NMR of **3ah.**

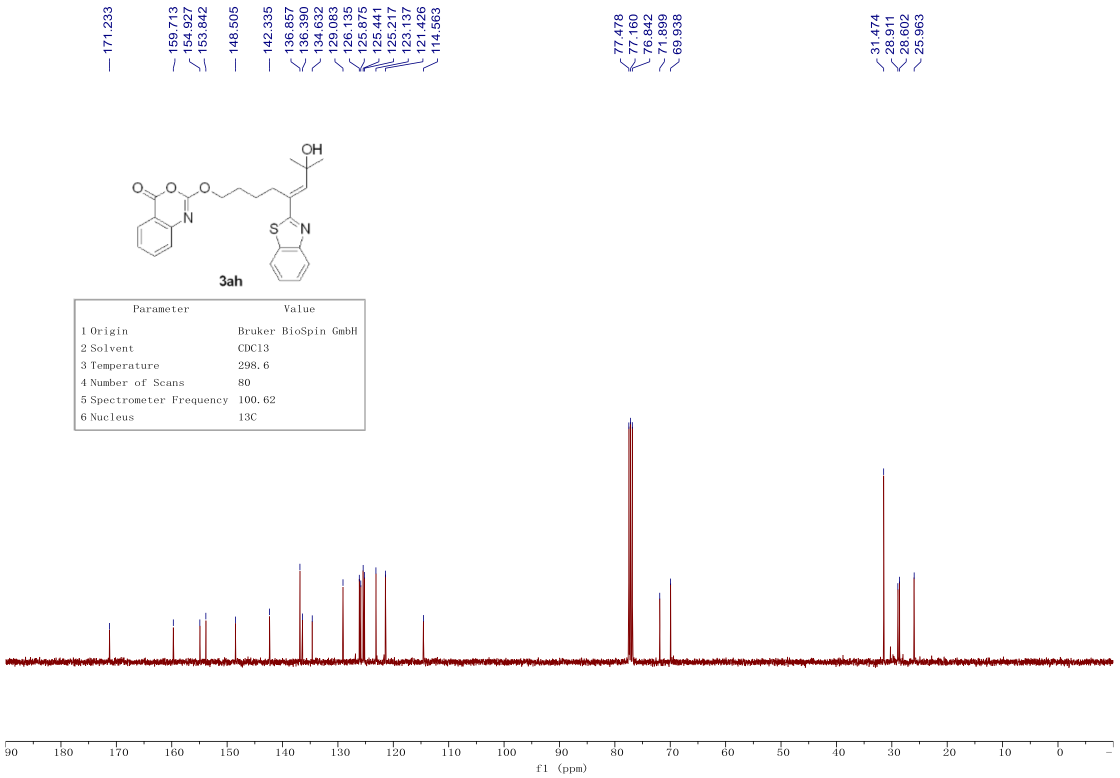

Figure S132. ^{13}C-NMR of **3ah.**

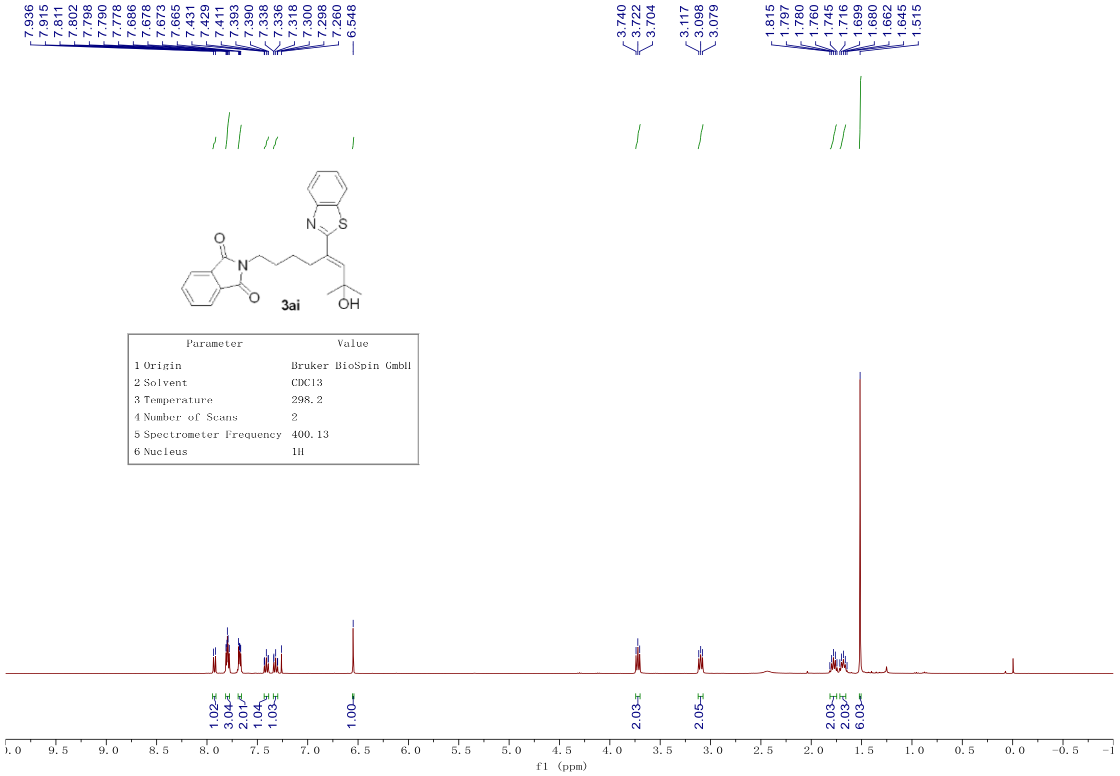

Figure S133. ^{1}H-NMR of **3ai.**

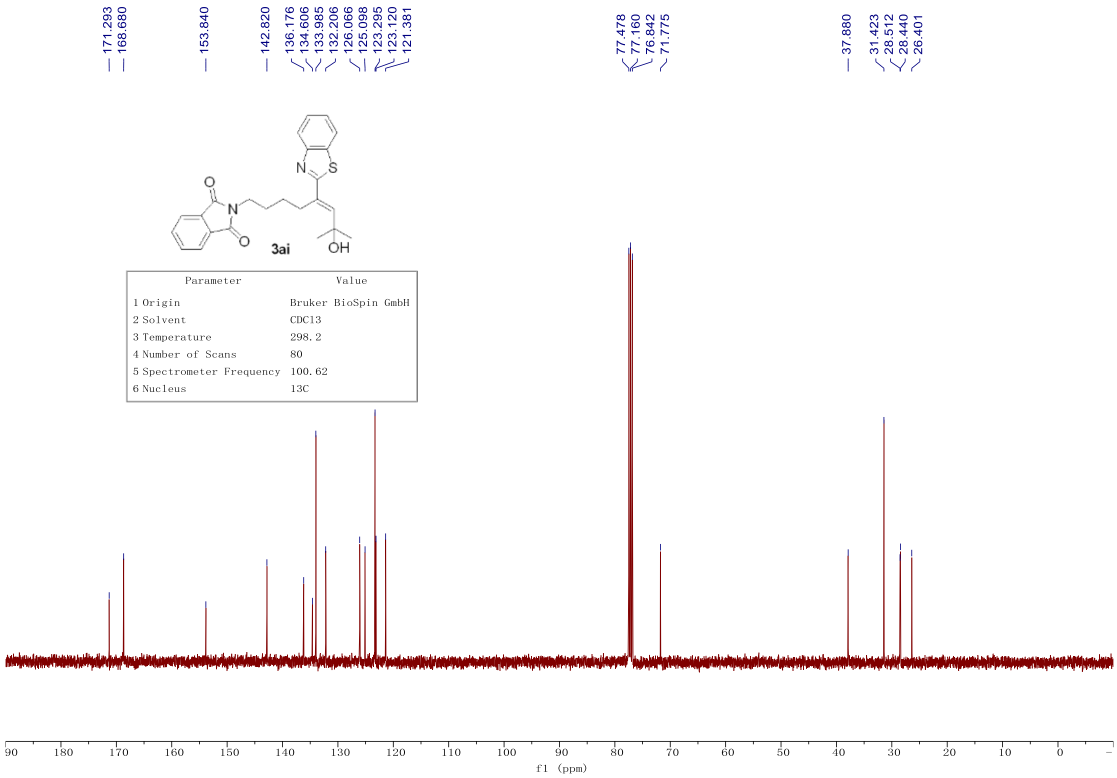

Figure S134. ^{13}C-NMR of **3ai.**

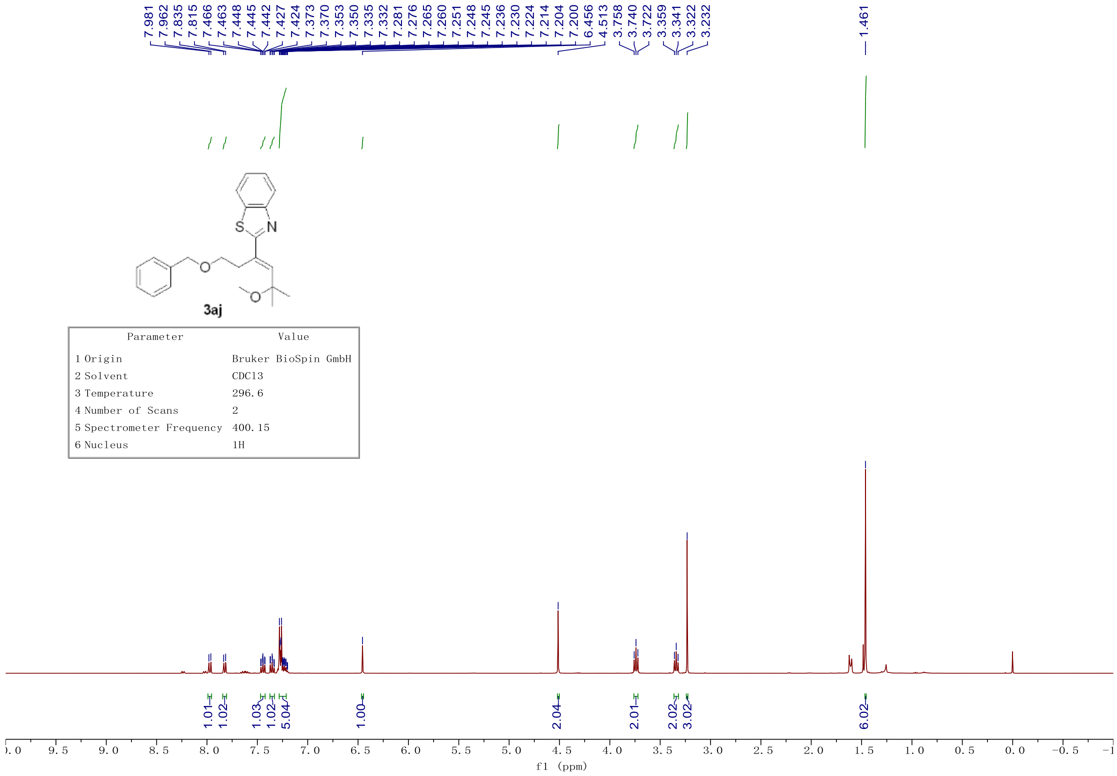

Figure S135. ^{1}H-NMR of **3aj.**

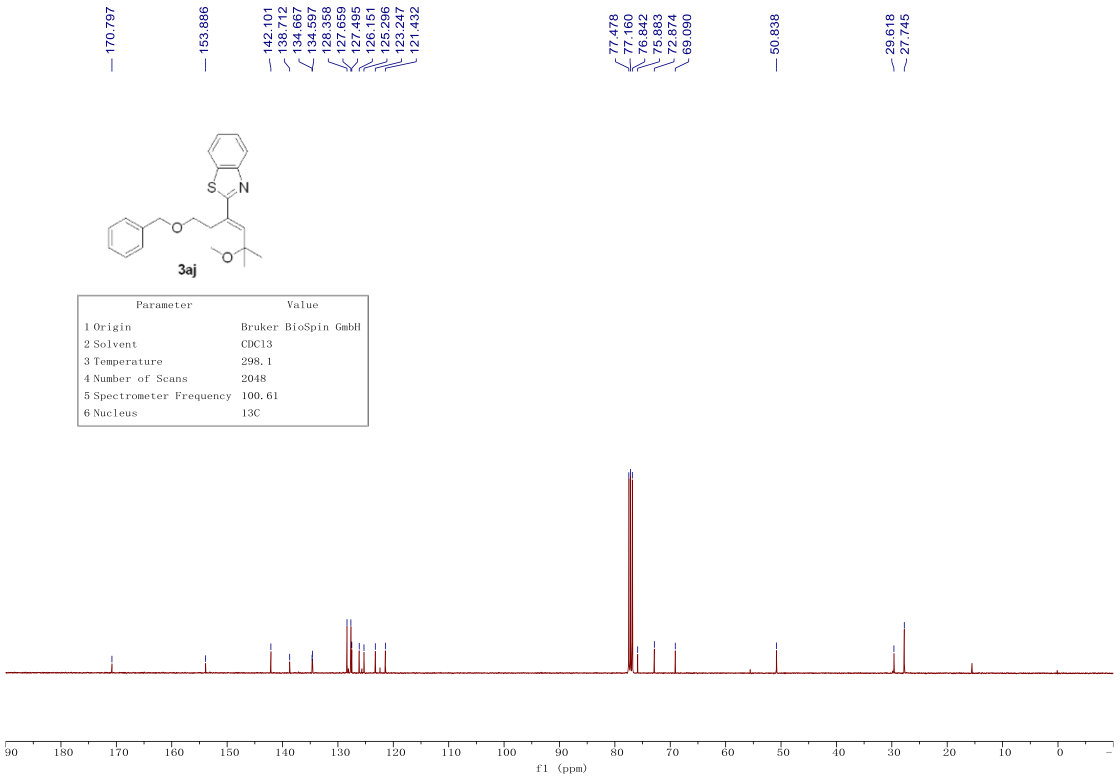

Figure S136. ^{13}C-NMR of **3aj.**

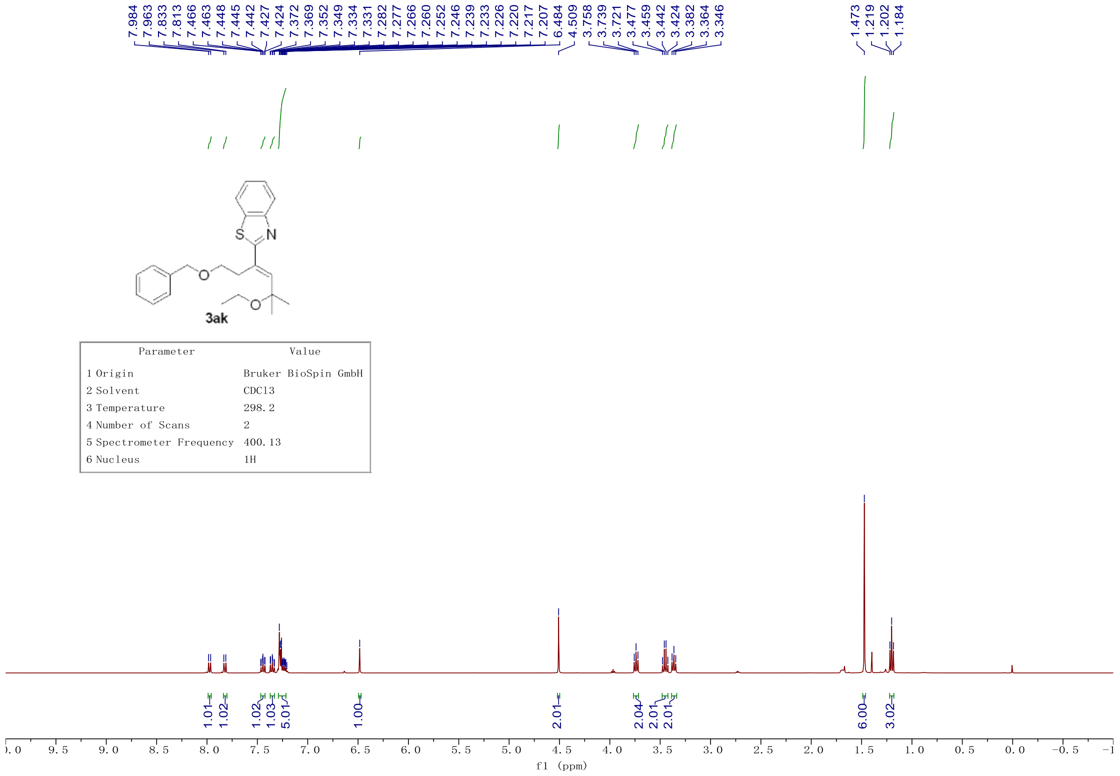

Figure S137. ^{1}H-NMR of **3ak.**

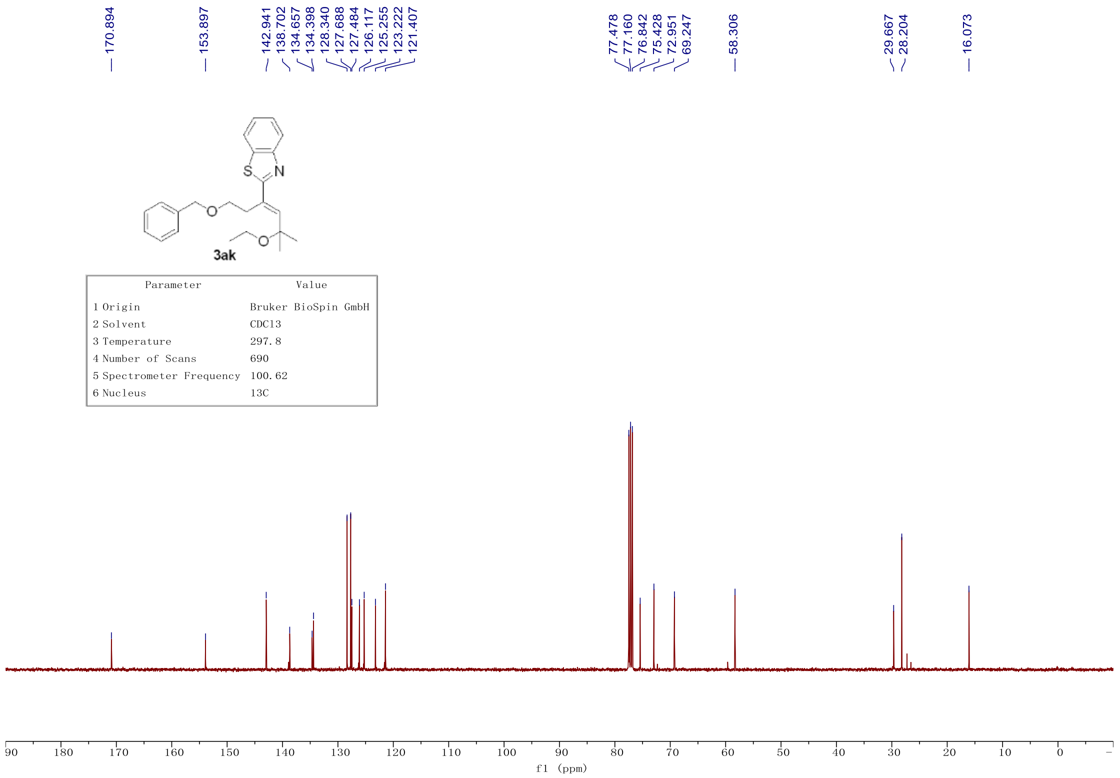

Figure S138. ^{13}C-NMR of **3ak.**

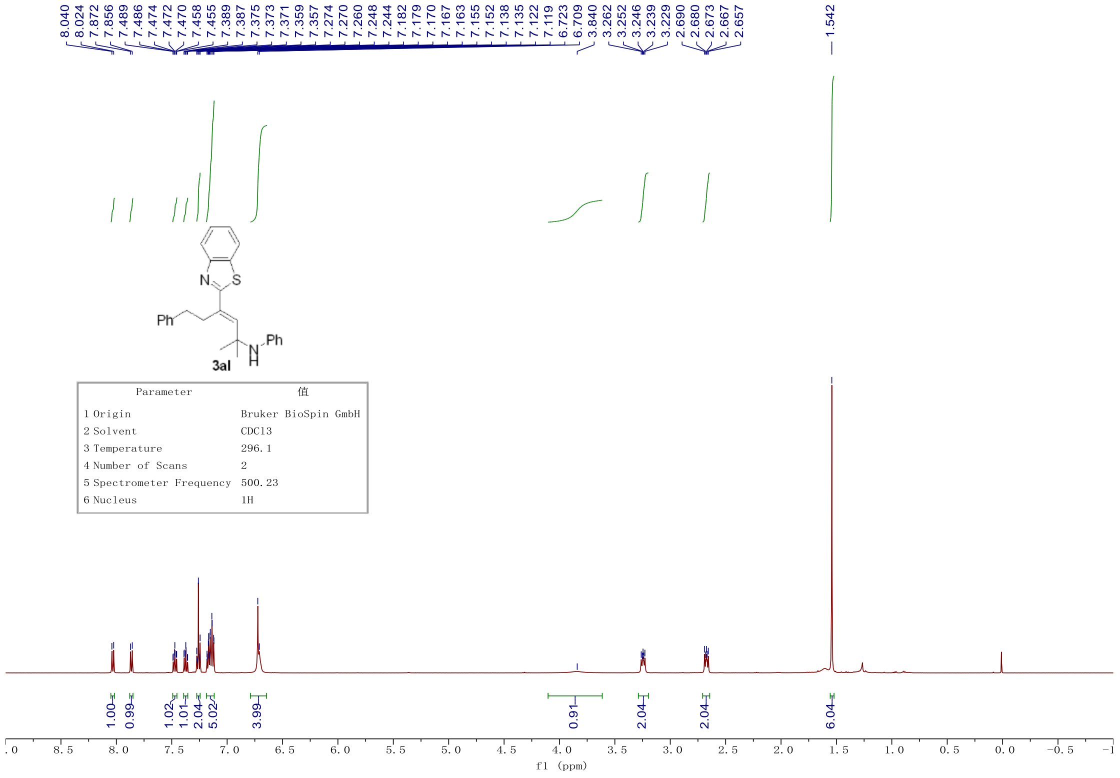

Figure S139. ^{1}H-NMR of **3al.**

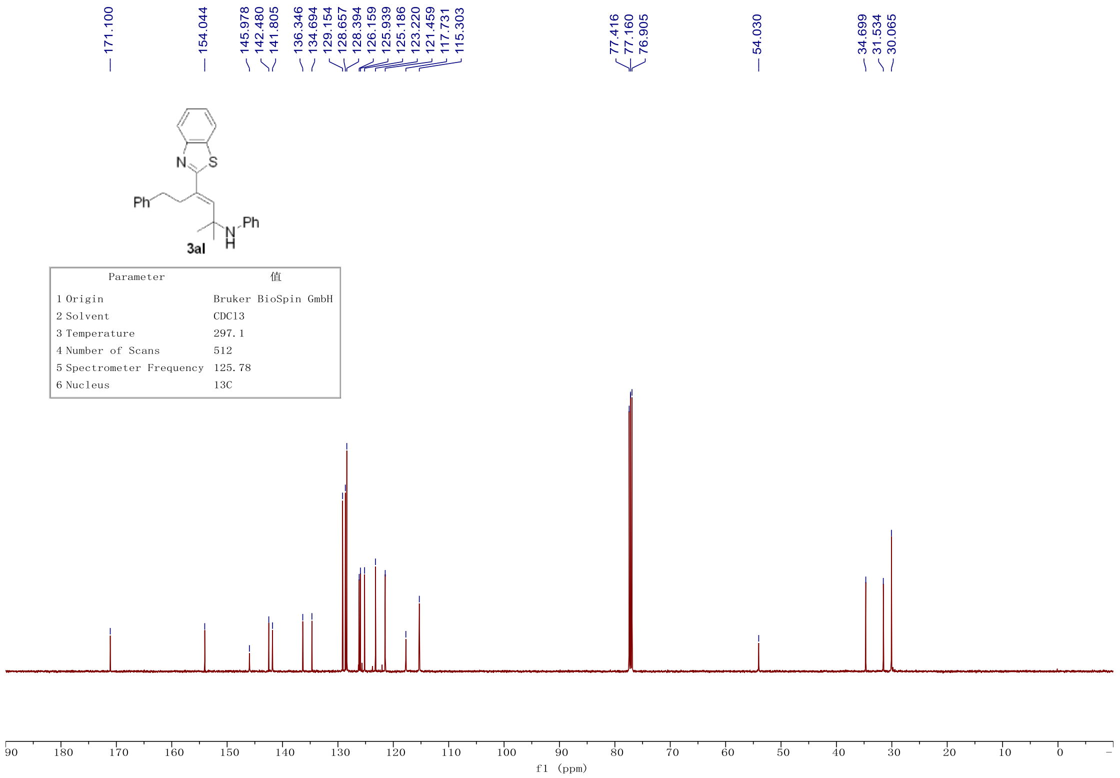

Figure S140. ^{13}C-NMR of **3al.**

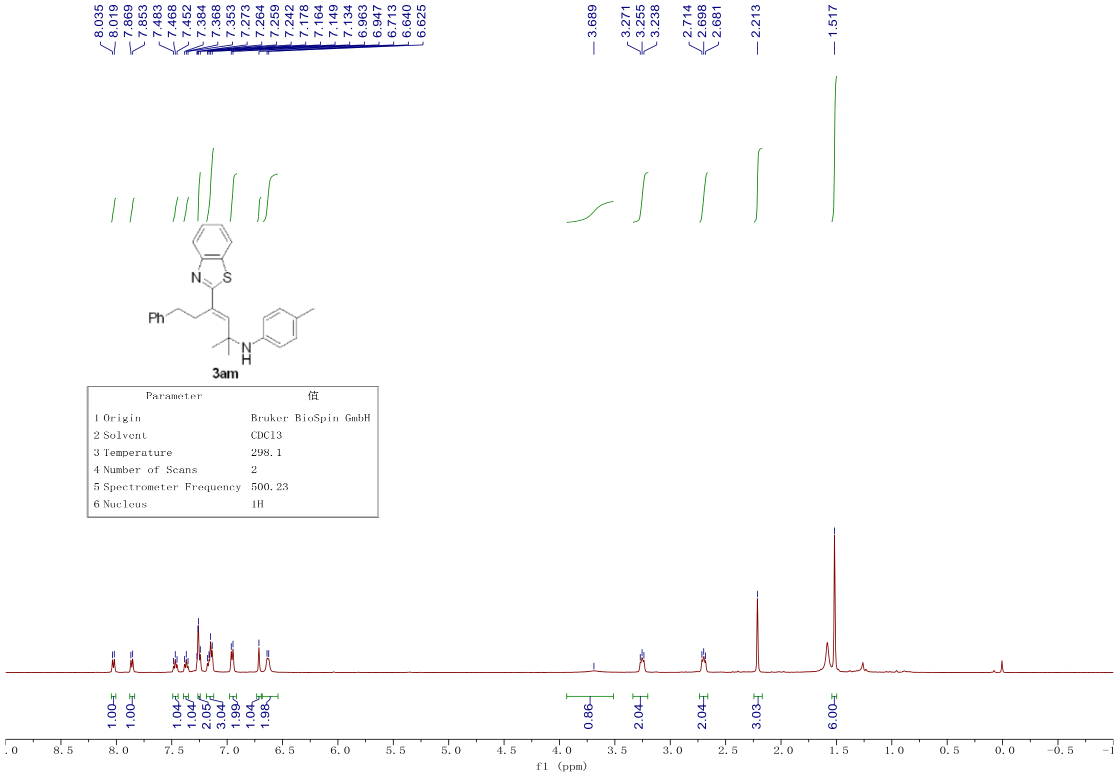

Figure S141. ^{1}H-NMR of **3am.**

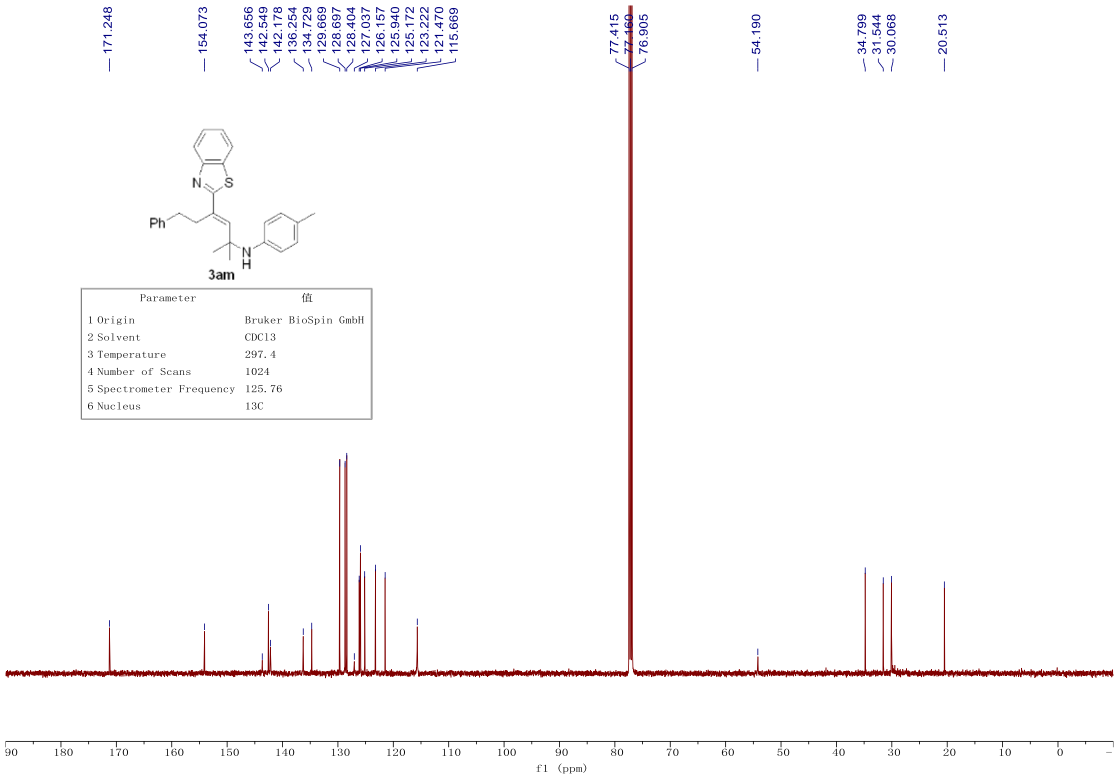

Figure S142. ^{13}C-NMR of **3am.**

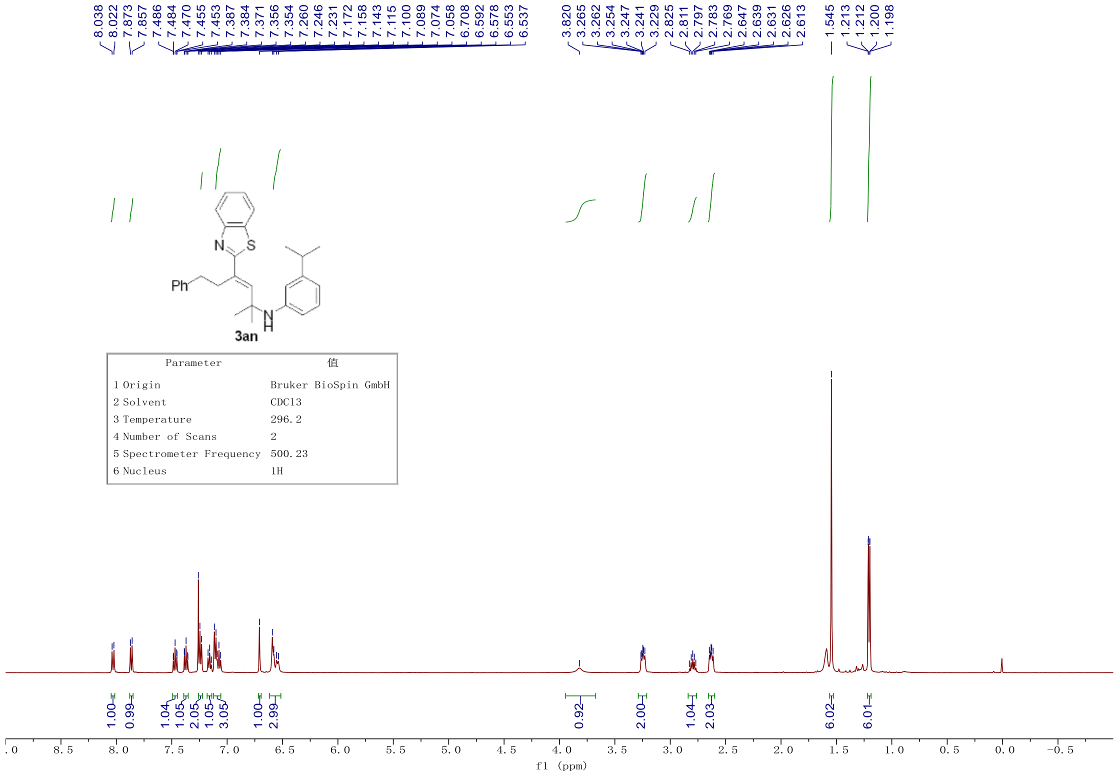

Figure S143. ^{1}H-NMR of **3an.**

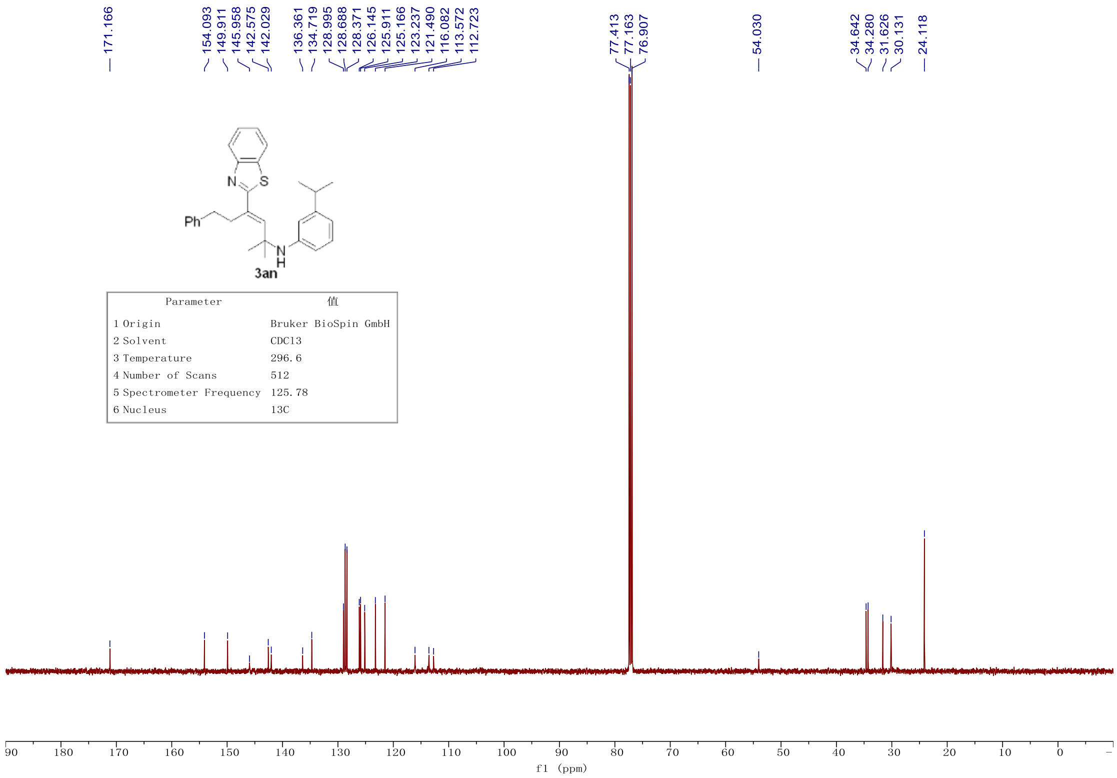

Figure S144. ^{13}C-NMR of **3an.**

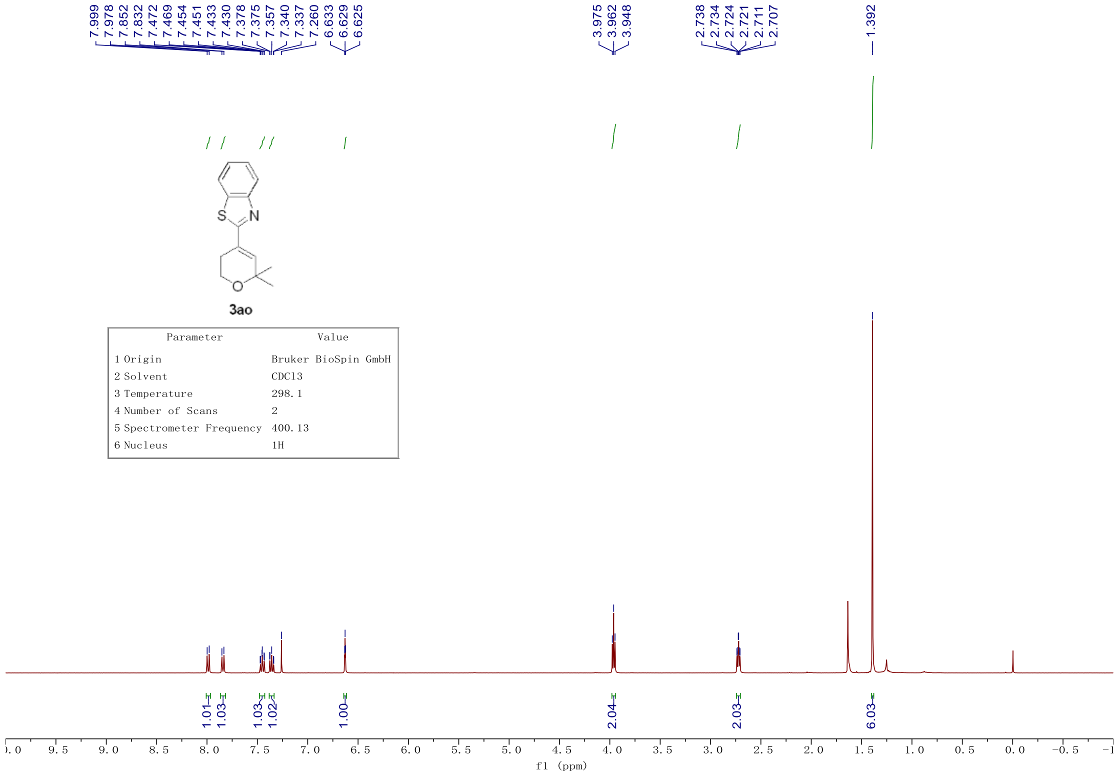

Figure S145. ^{1}H-NMR of **3ao.**

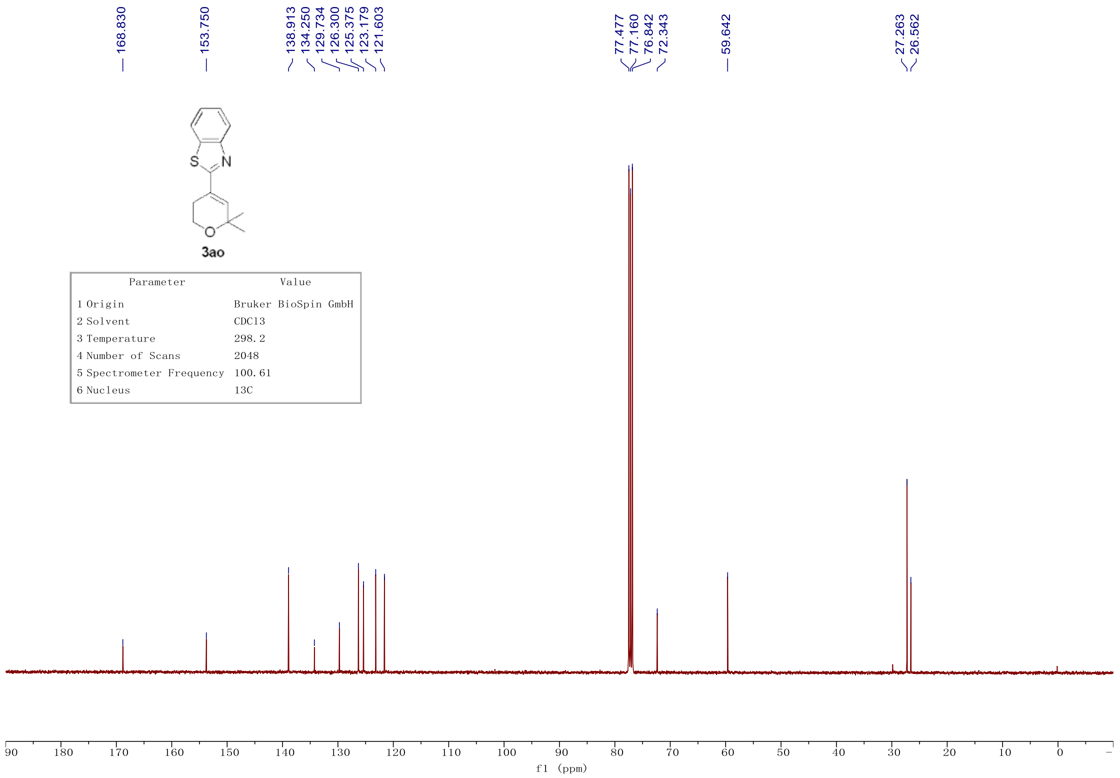

Figure S146. ^{13}C-NMR of **3ao.**

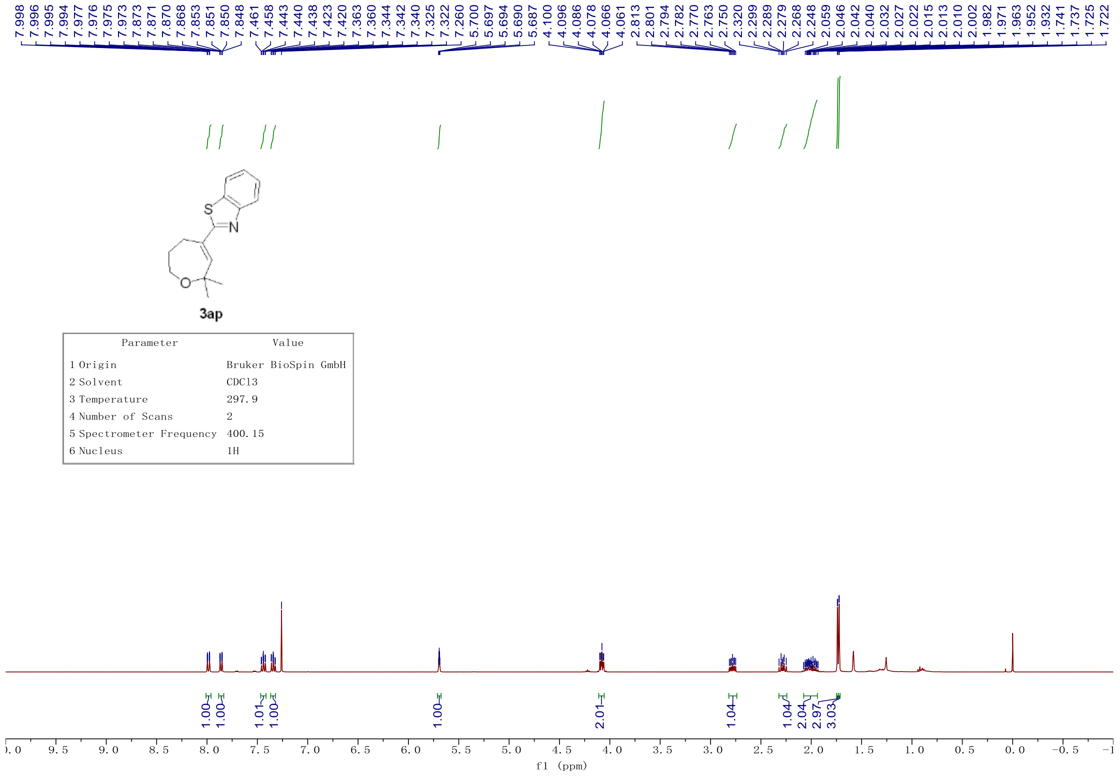

Figure S147. ^{1}H-NMR of **3ap.**

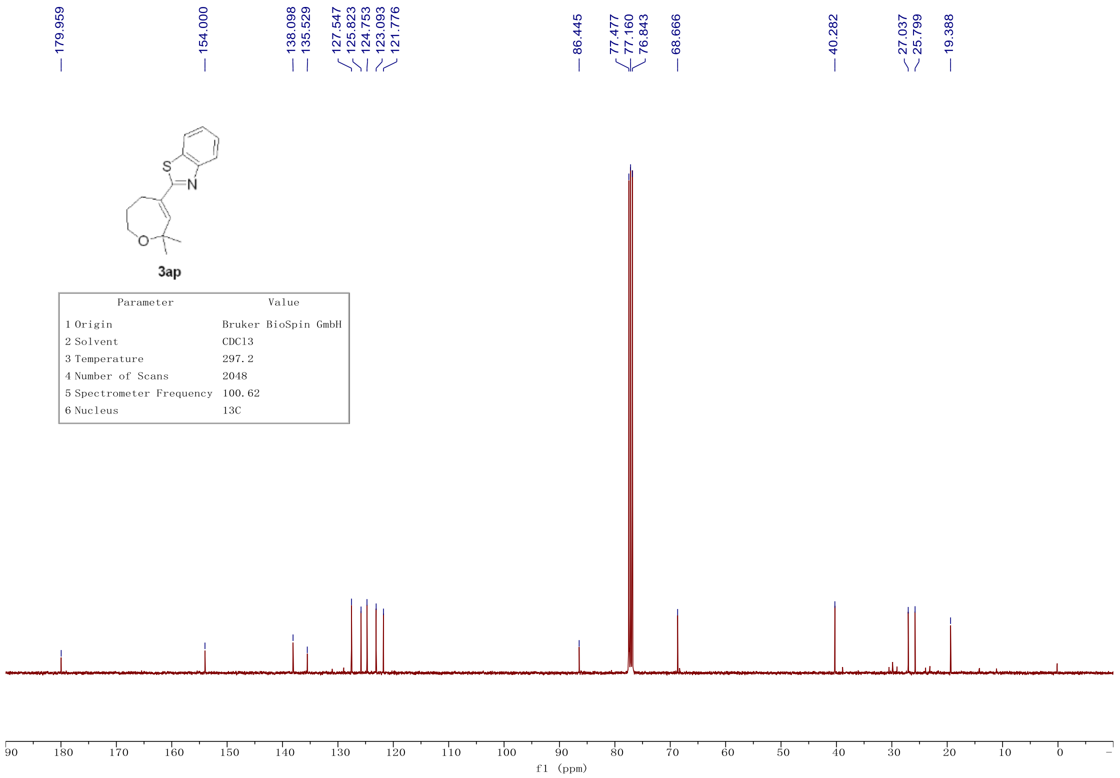

Figure S148. ^{13}C-NMR of **3ap.**

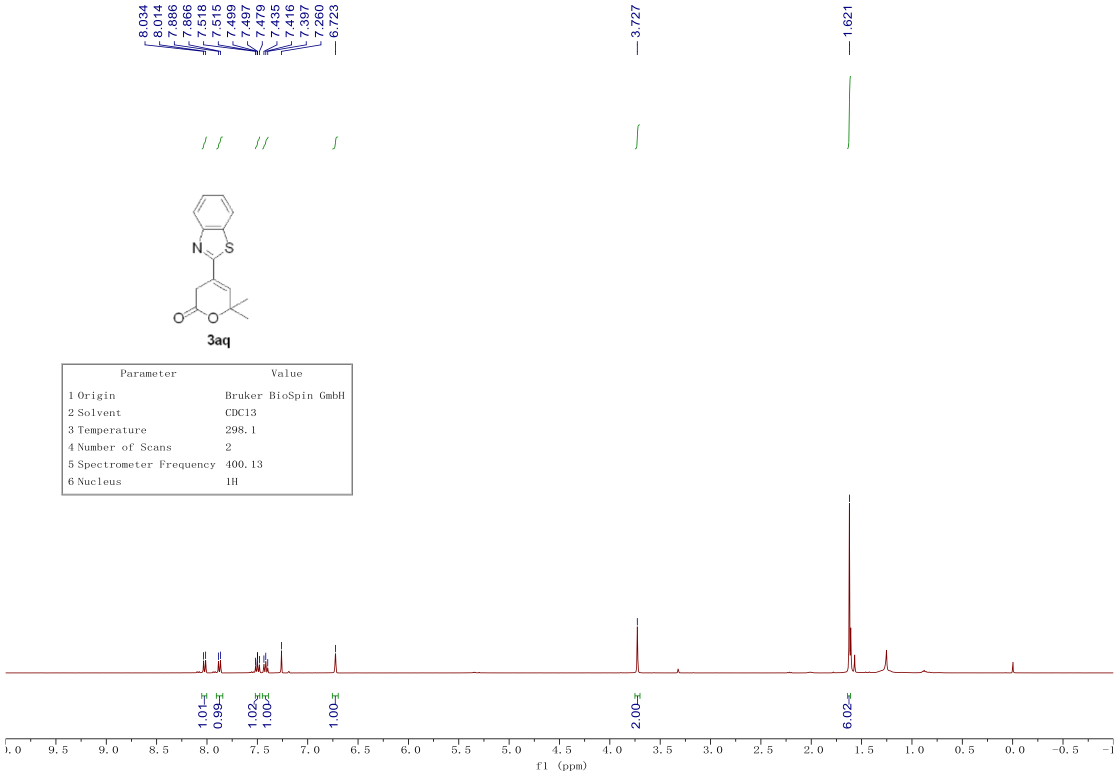

Figure S149. ^{1}H-NMR of **3aq.**

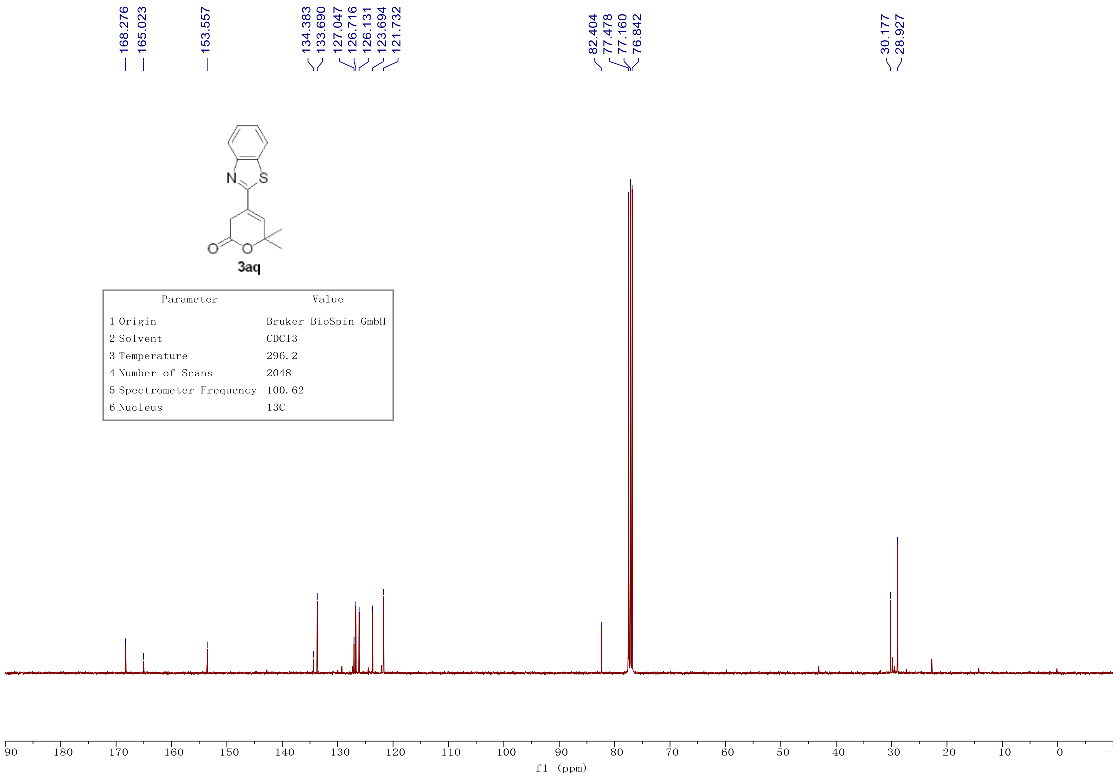

Figure S150. ^{13}C-NMR of **3aq.**

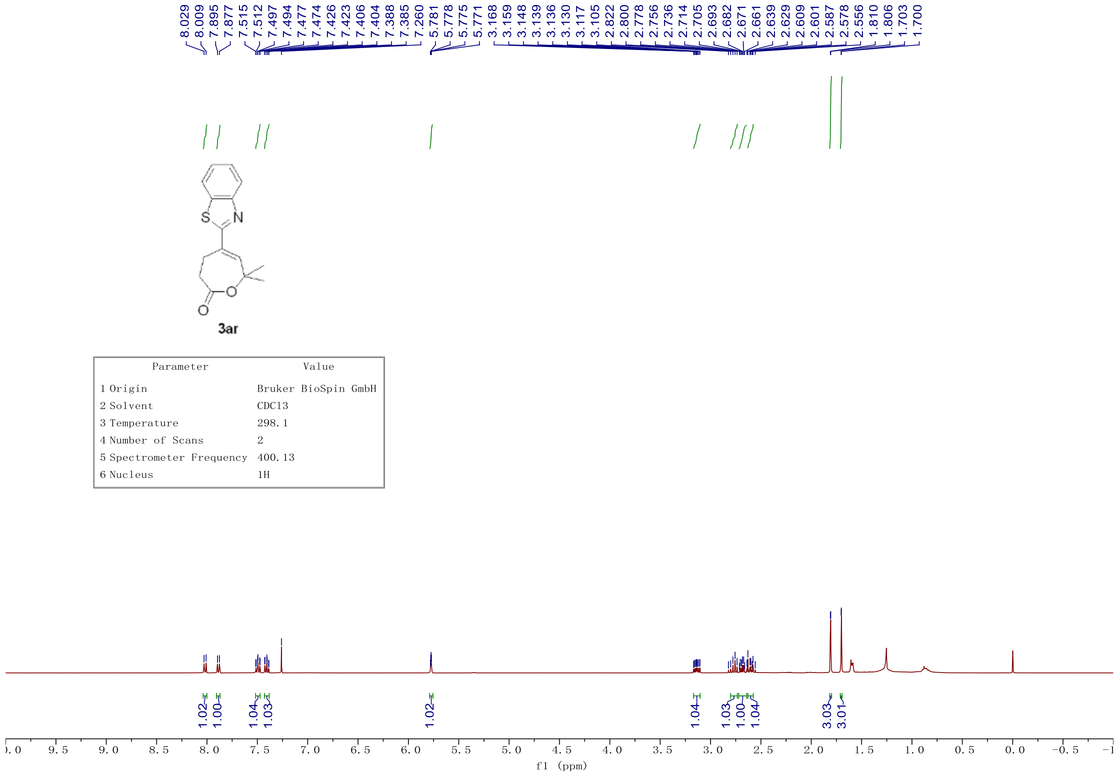

Figure S151. ^{1}H-NMR of **3ar.**

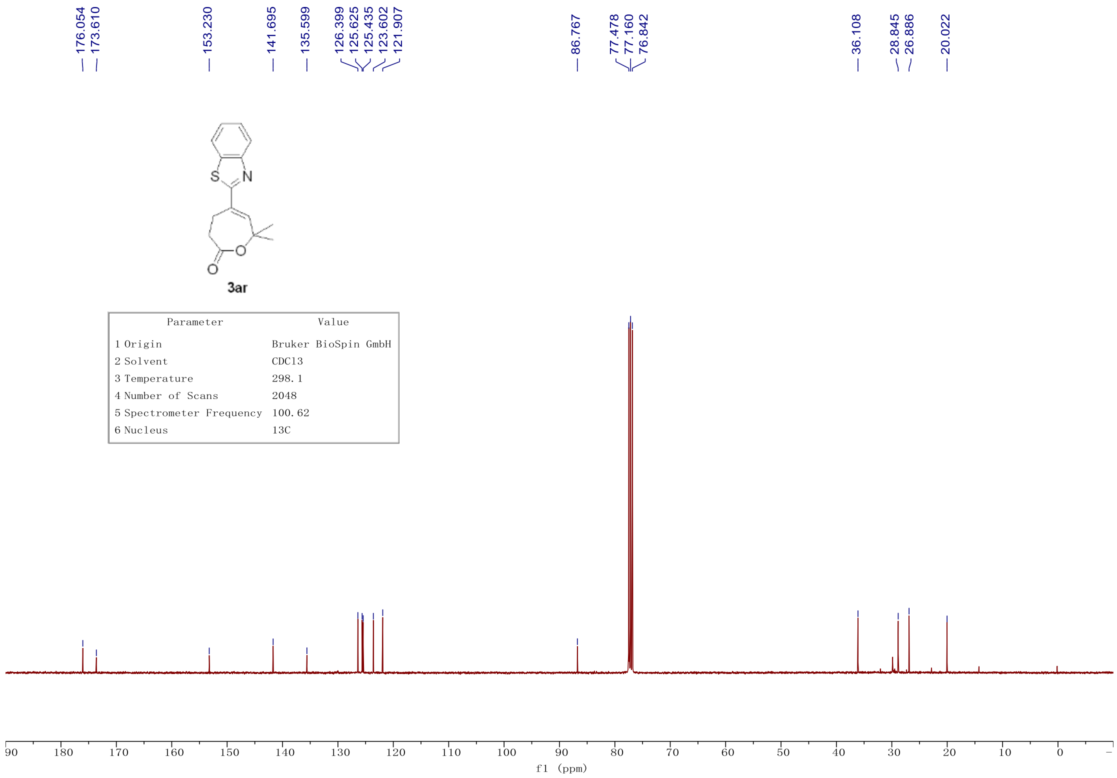

Figure S152. ^{13}C-NMR of **3ar.**

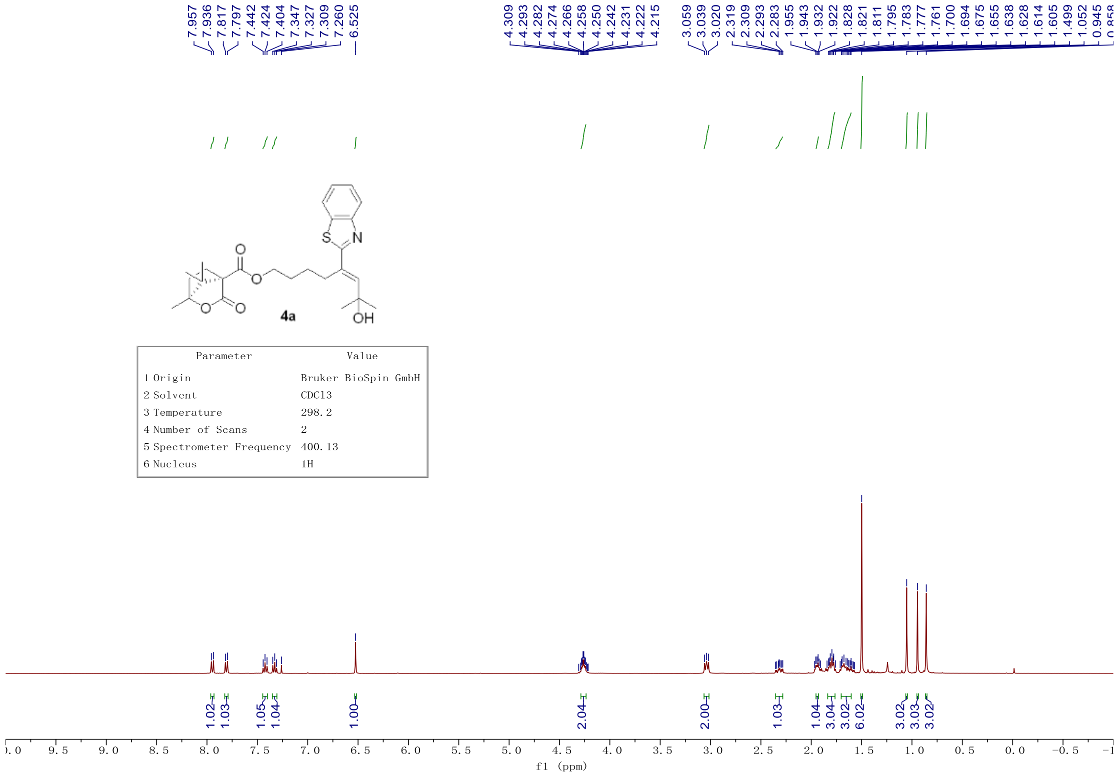

Figure S153. ^{1}H-NMR of **4a.**

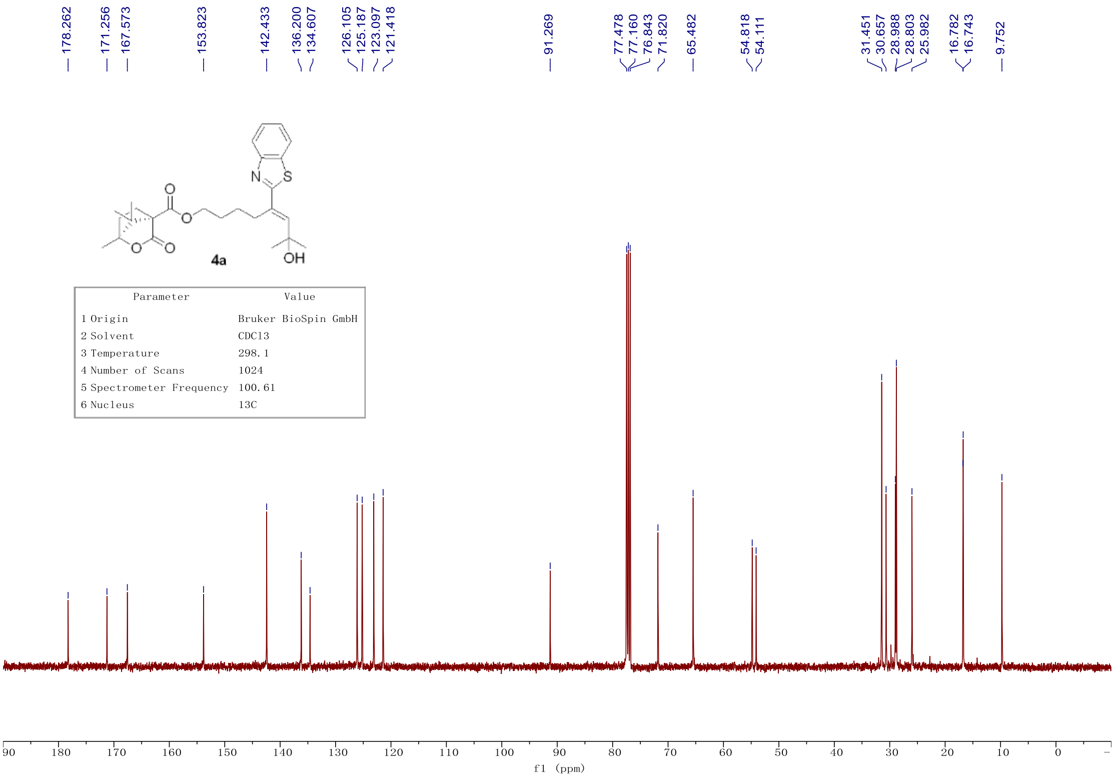

Figure S154. ^{13}C-NMR of **4a.**

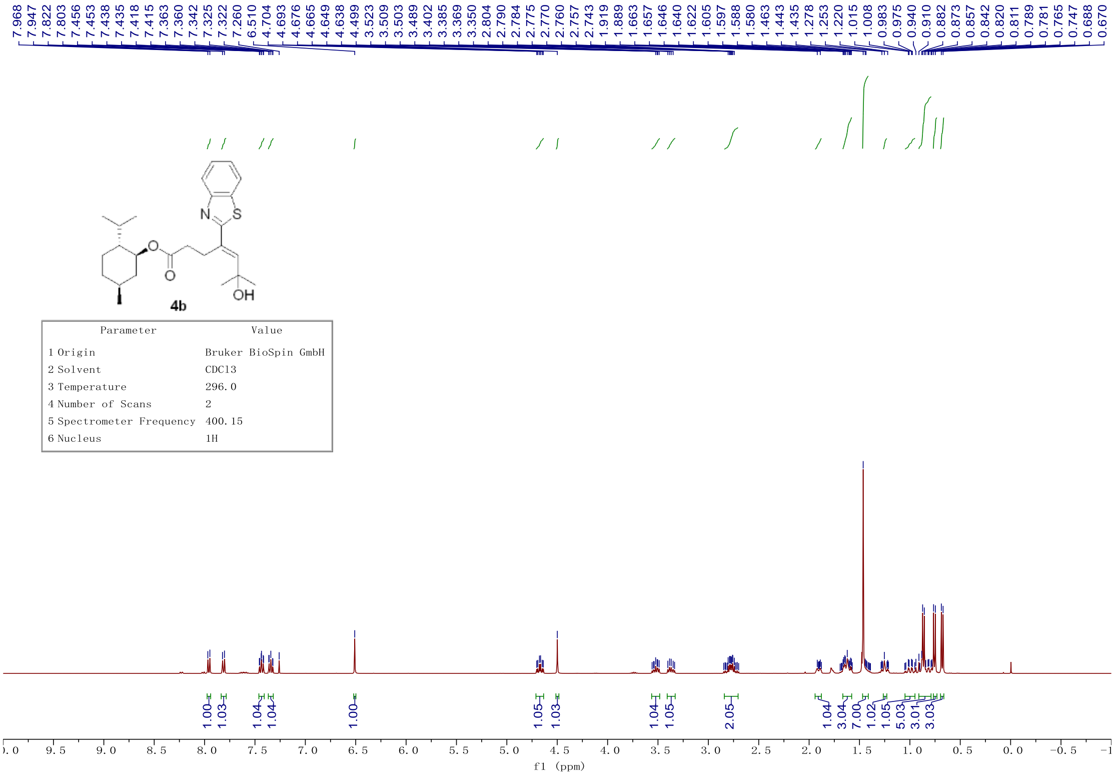

Figure S155. ^{1}H-NMR of **4b.**

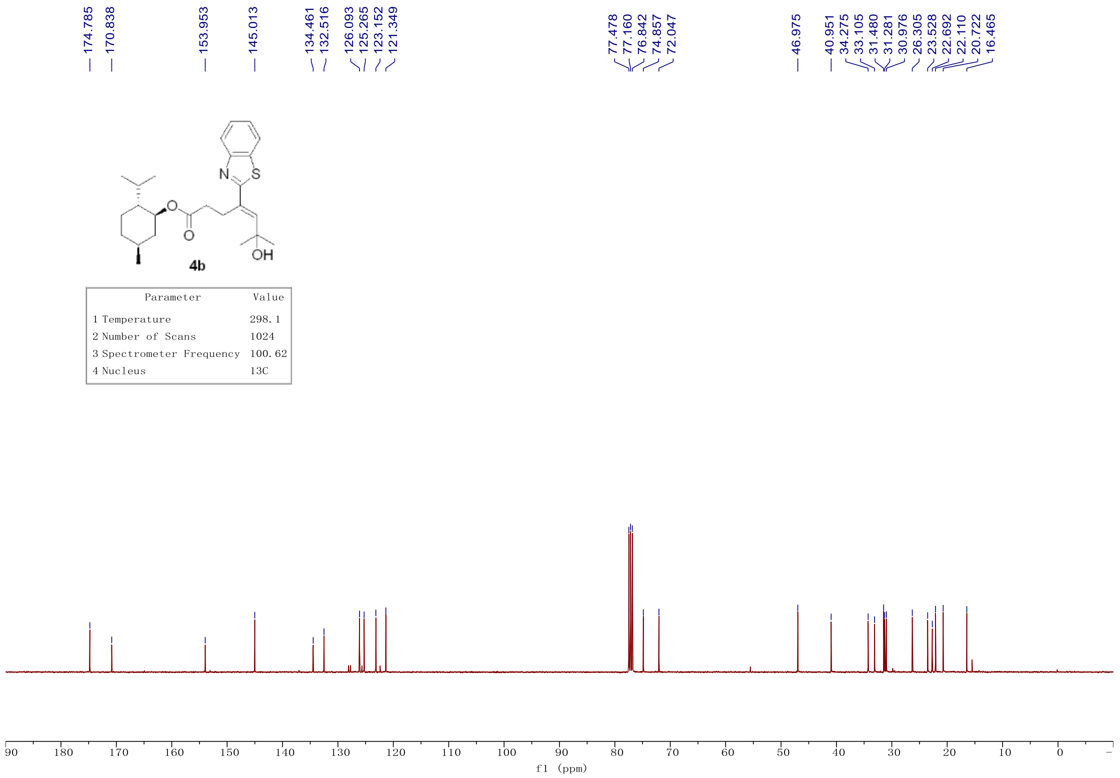

Figure S156. ^{13}C-NMR of **4b.**

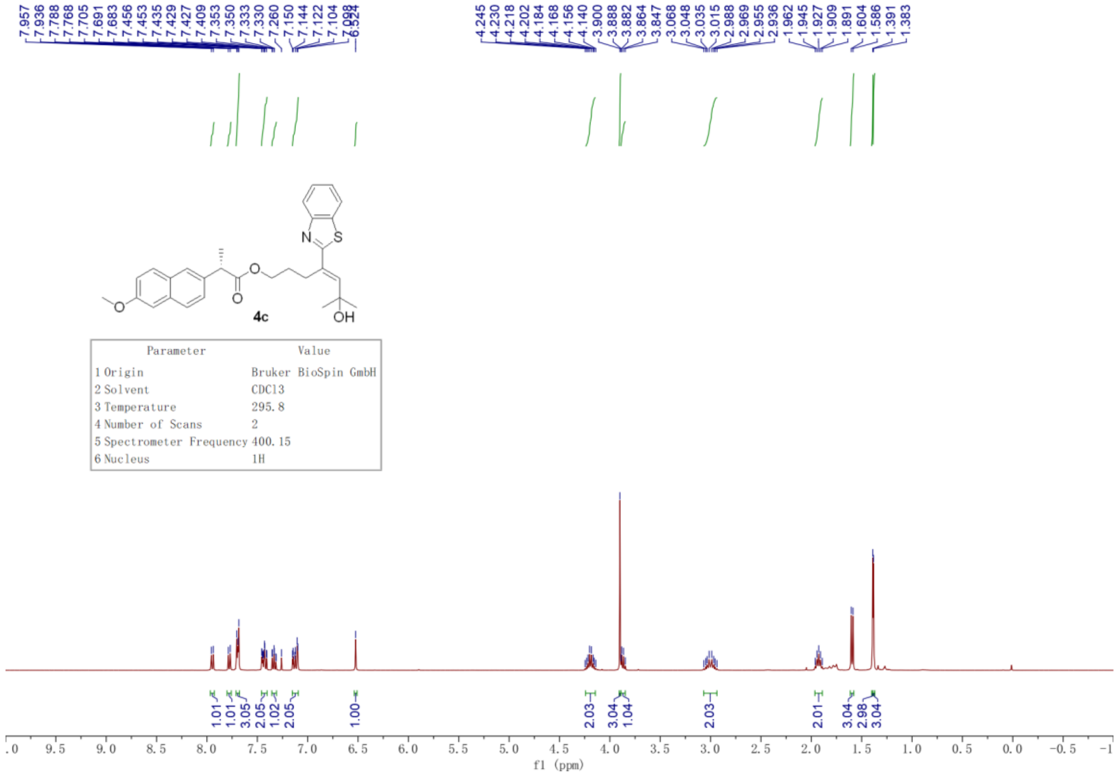

Figure S157. ^{1}H-NMR of **4c.**

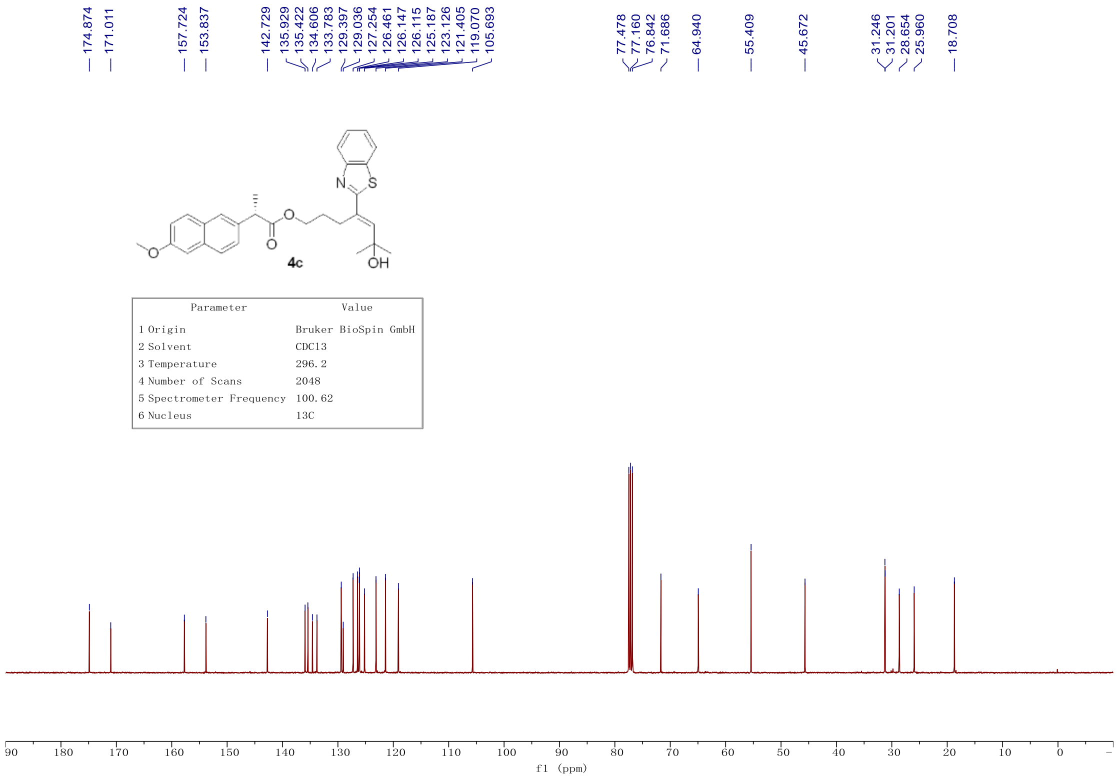

Figure S158. ^{13}C-NMR of **4c.**

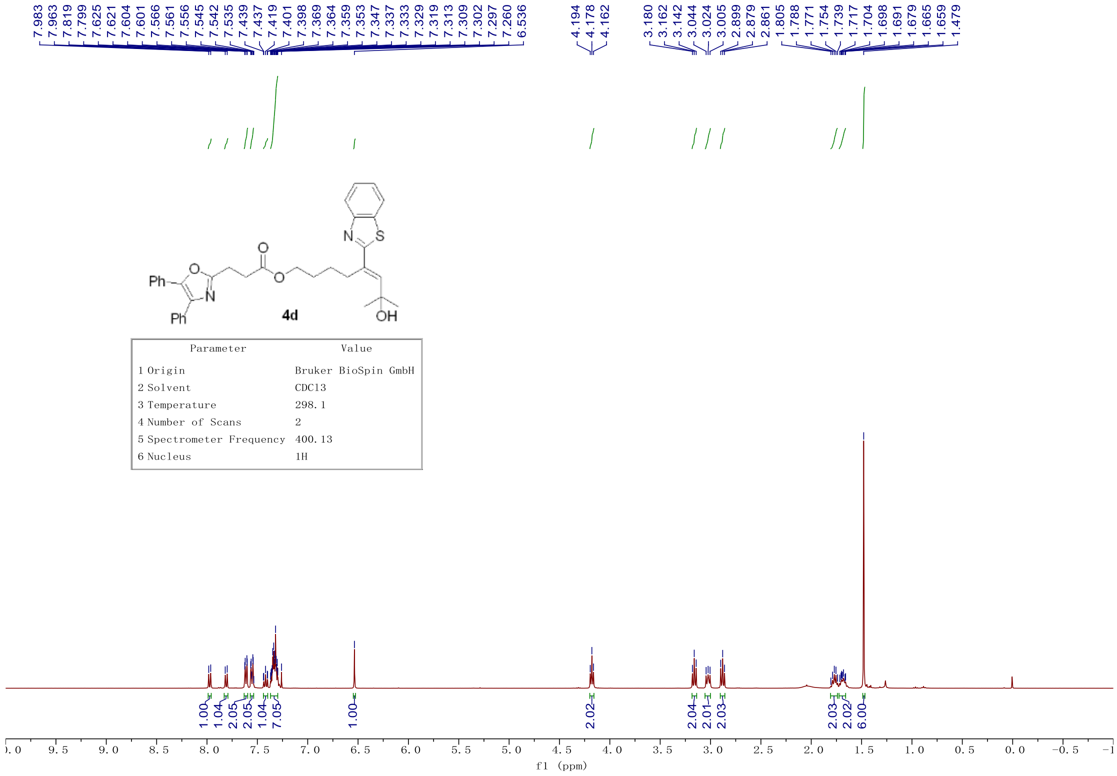

Figure S159. ^{1}H-NMR of **4d.**

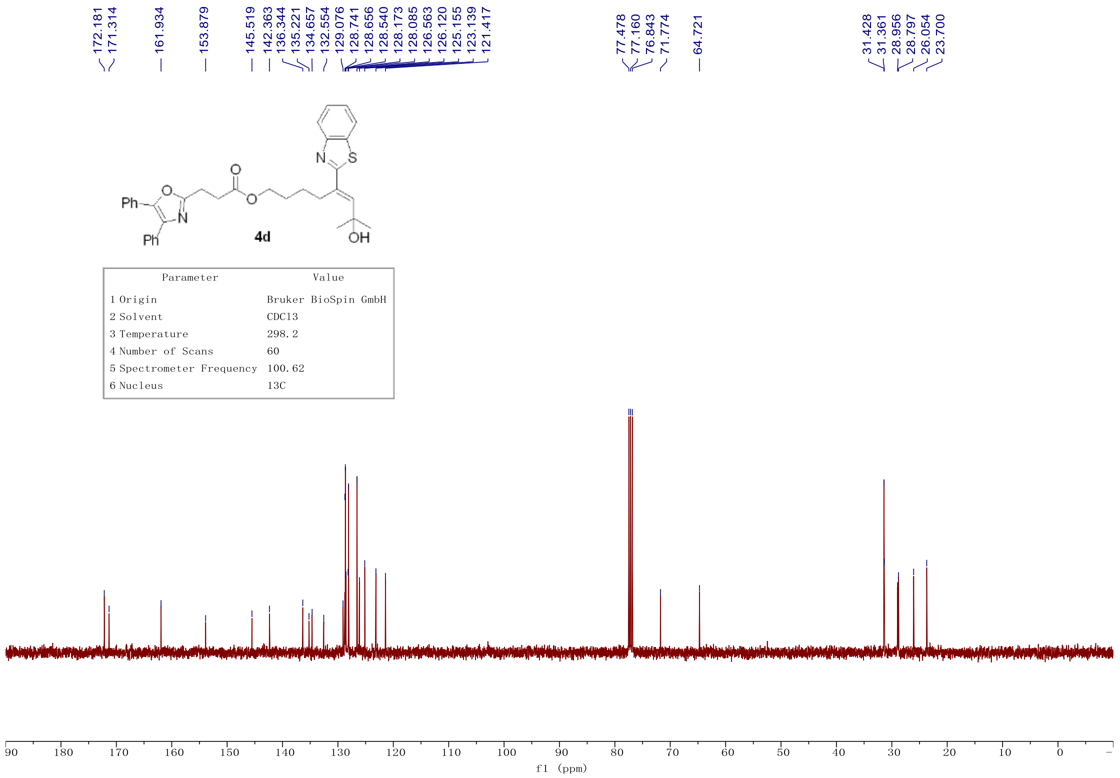

Figure S160. ^{13}C-NMR of **4d.**

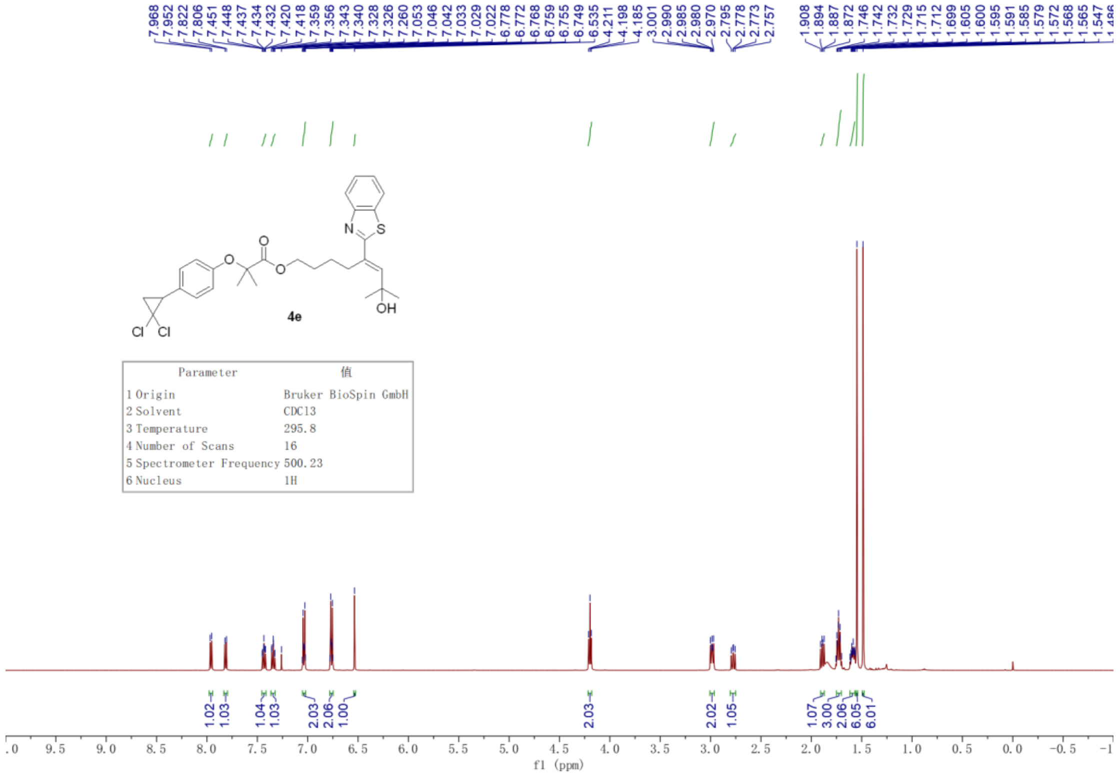

Figure S161. ^{1}H-NMR of **4e.**

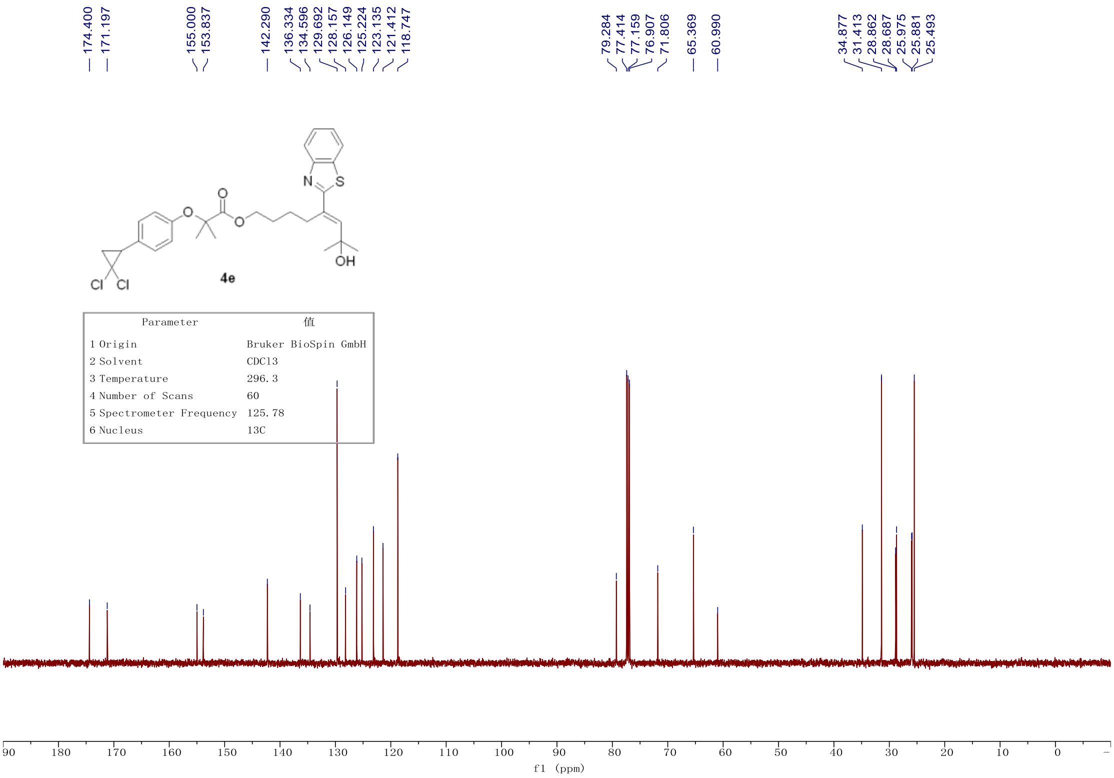

Figure S162. ^{13}C-NMR of **4e.**

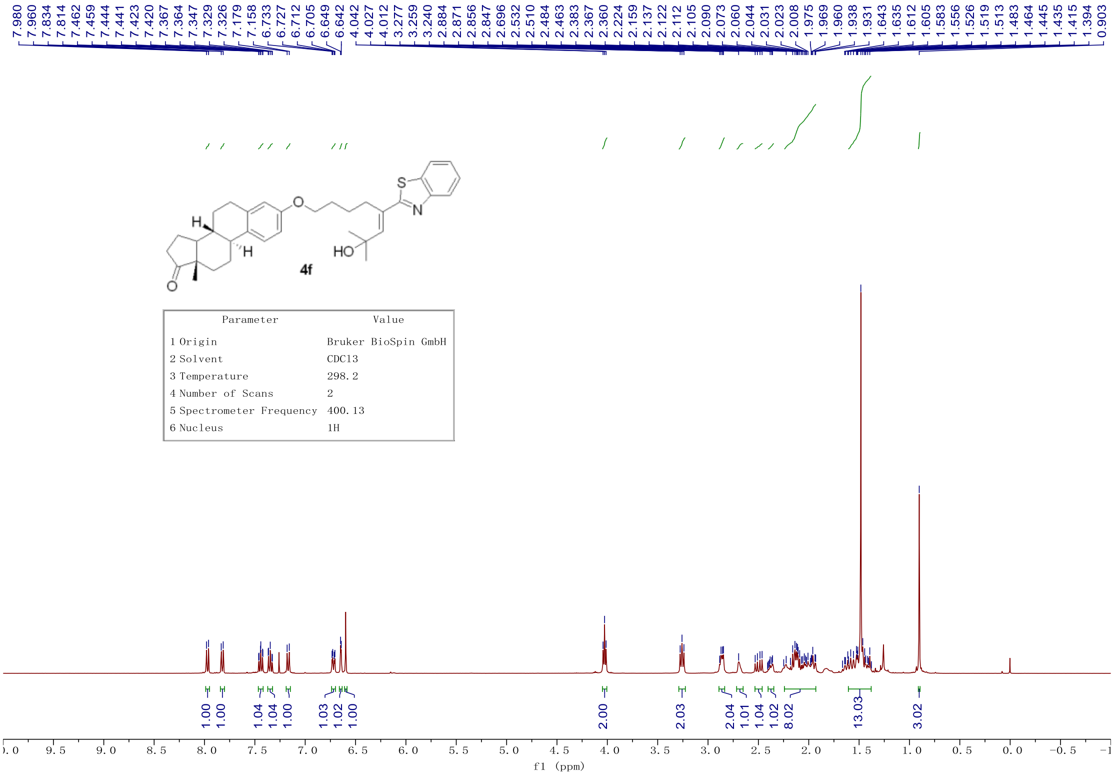

Figure S163. ^{1}H-NMR of **4f.**

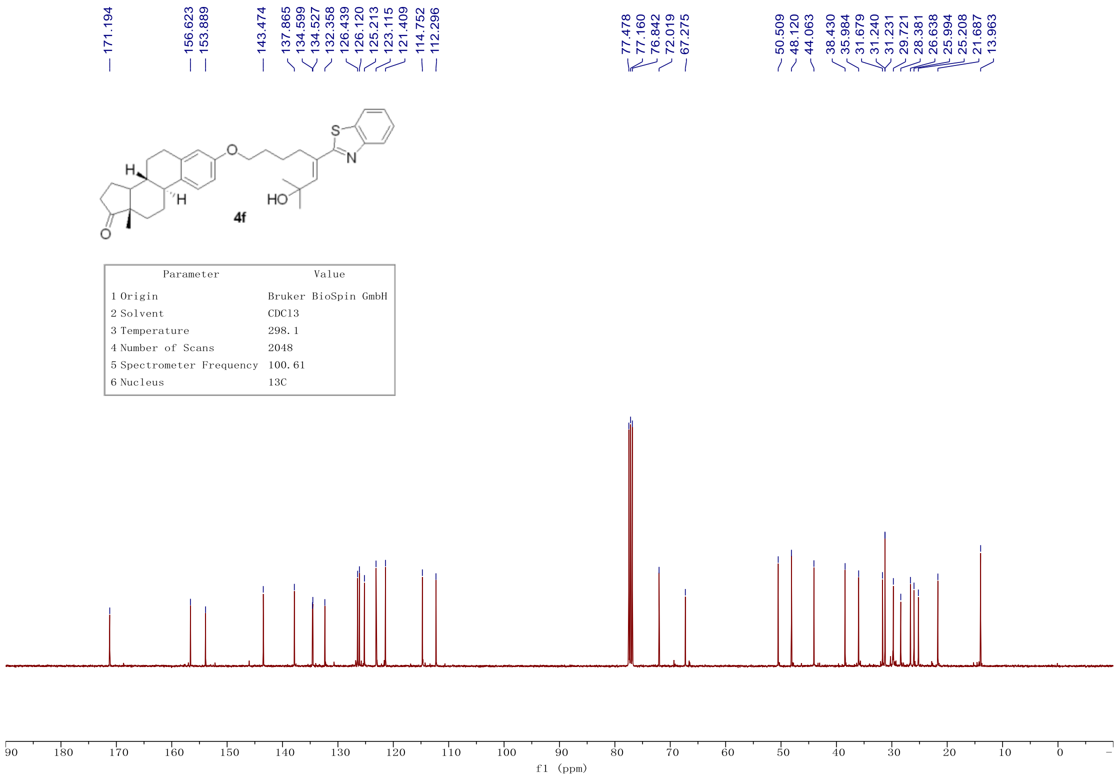

Figure S164. ^{13}C-NMR of **4f.**

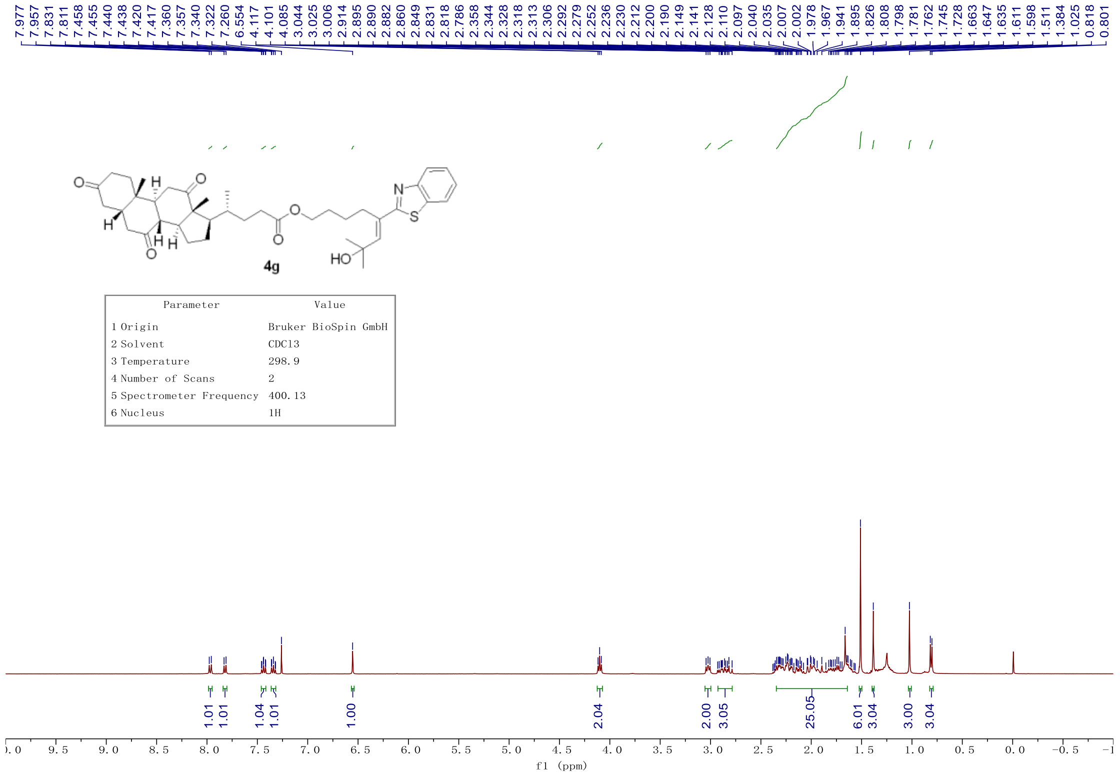

Figure S165. ^{1}H-NMR of **4g.**

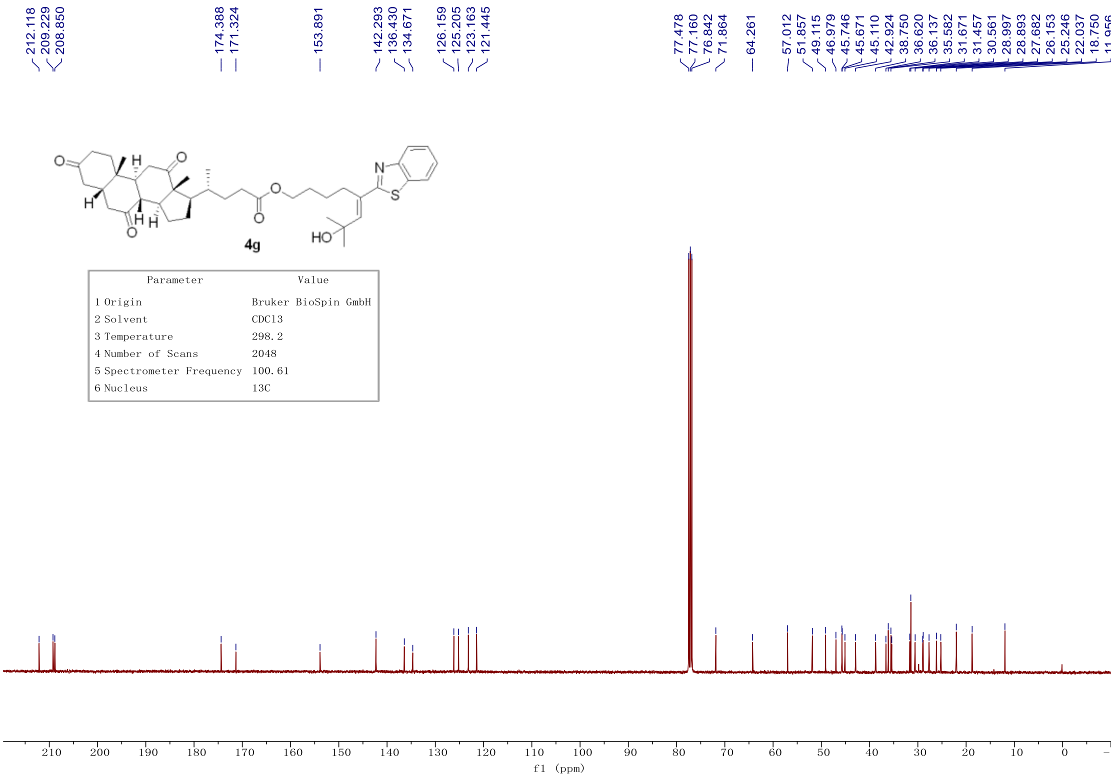

Parameter	Value
1 Origin	Bruker BioSpin GmbH
2 Solvent	CDCl3
3 Temperature	298.2
4 Number of Scans	2048
5 Spectrometer Frequency	100.61
6 Nucleus	13C

Figure S166. ^{13}C-NMR of **4g.**

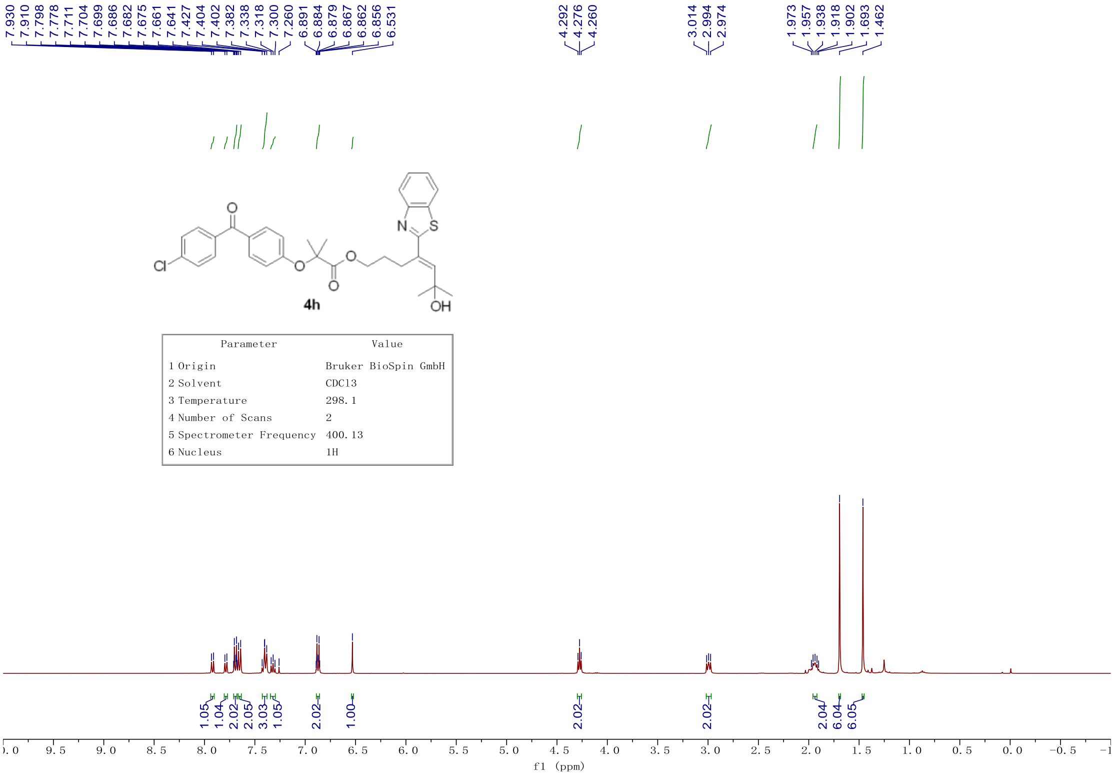

Parameter	Value
1 Origin	Bruker BioSpin GmbH
2 Solvent	CDCl3
3 Temperature	298.1
4 Number of Scans	2
5 Spectrometer Frequency	400.13
6 Nucleus	1H

Figure S167. ^{1}H-NMR of **4h.**

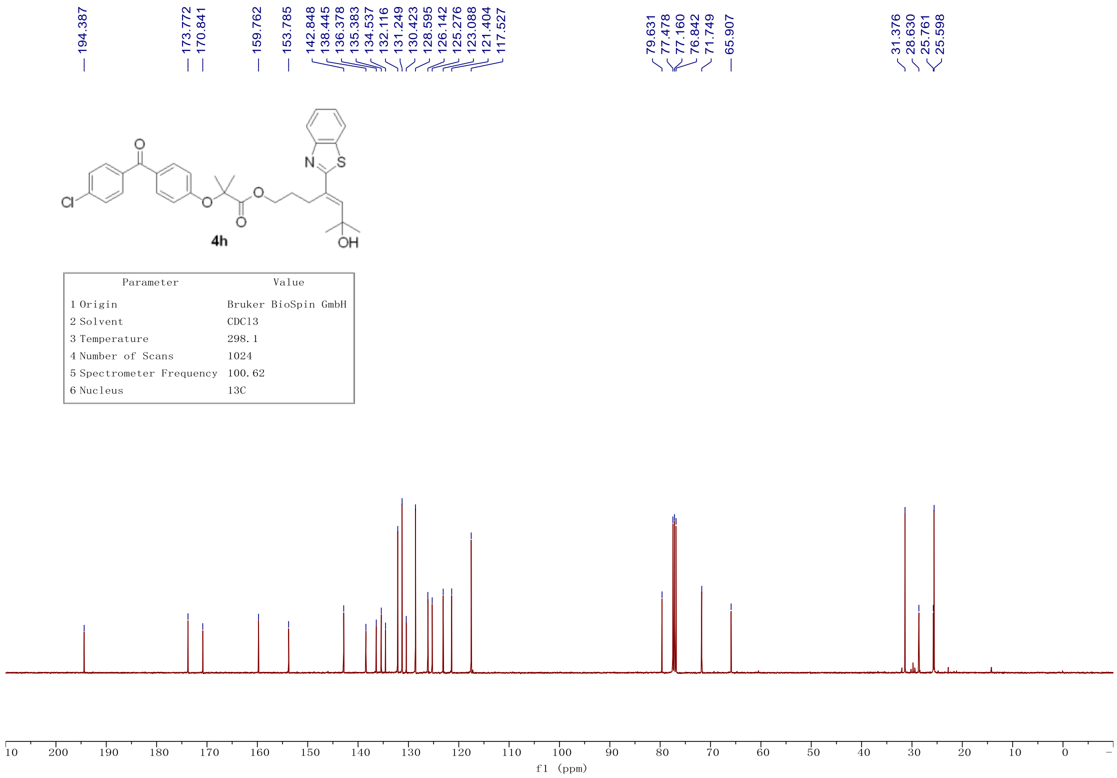

Figure S168. ^{13}C-NMR of **4h.**

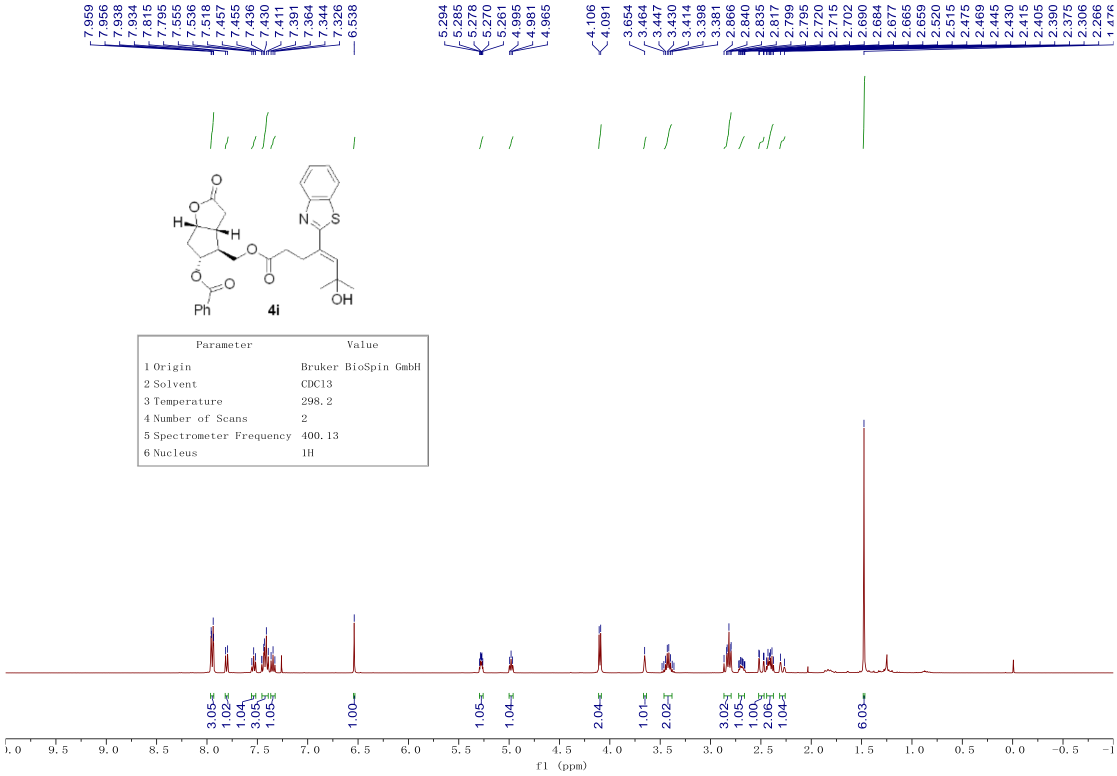

Figure S169. ^{1}H-NMR of **4i.**

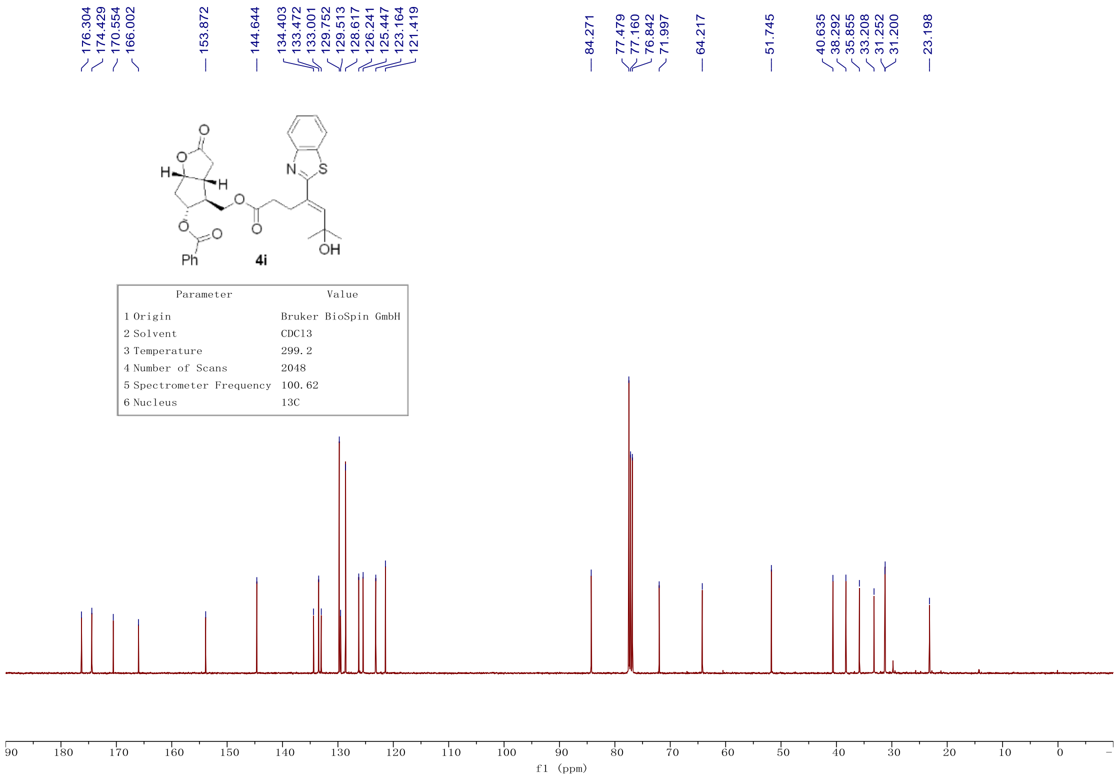

Figure S170. ^{13}C-NMR of **4i.**

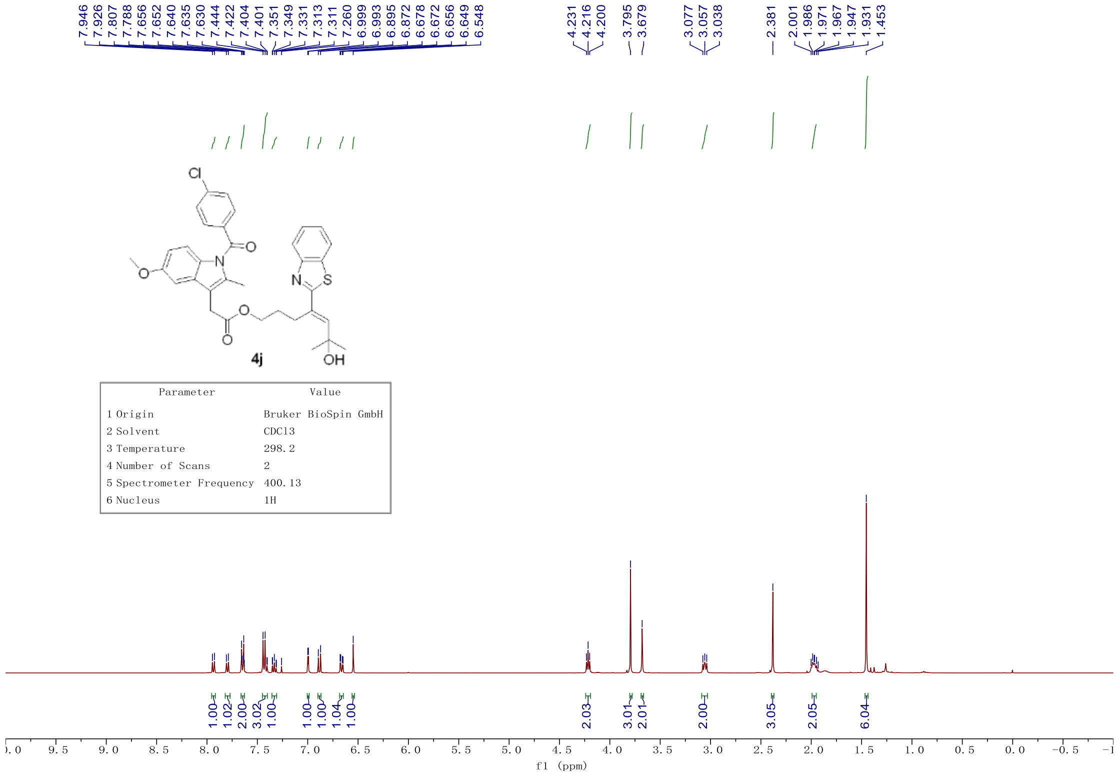

Figure S171. ^{1}H-NMR of **4j.**

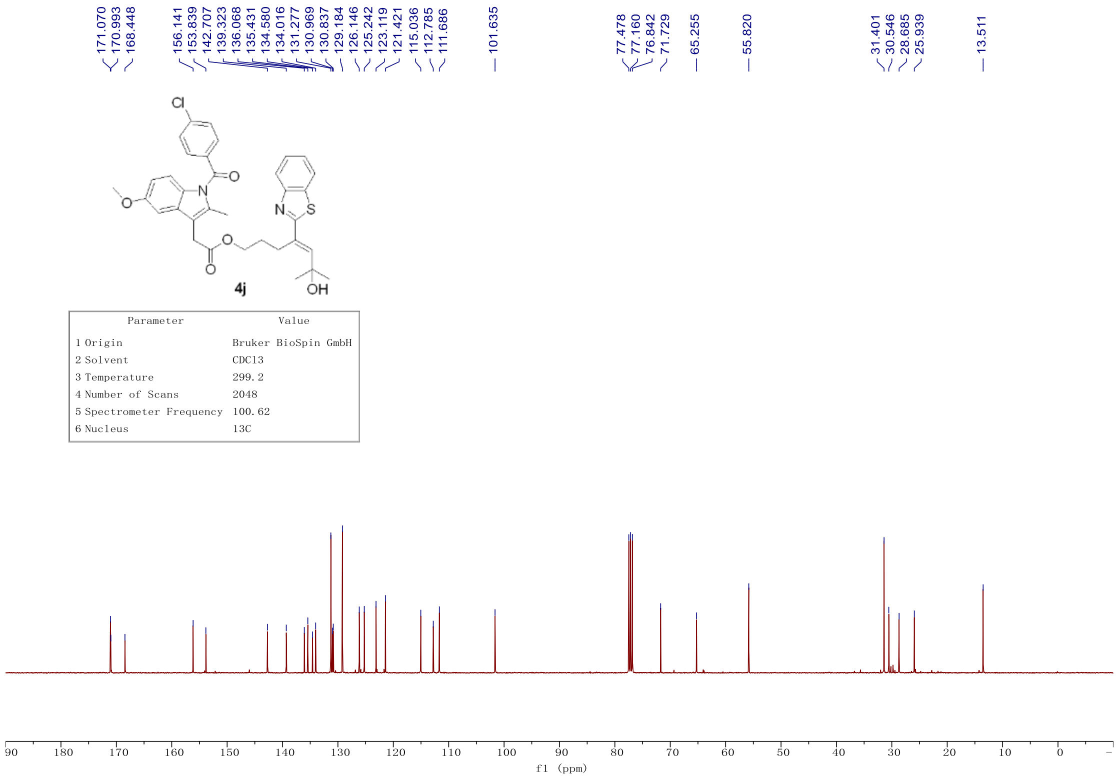

Figure S172. ^{13}C-NMR of **4j.**

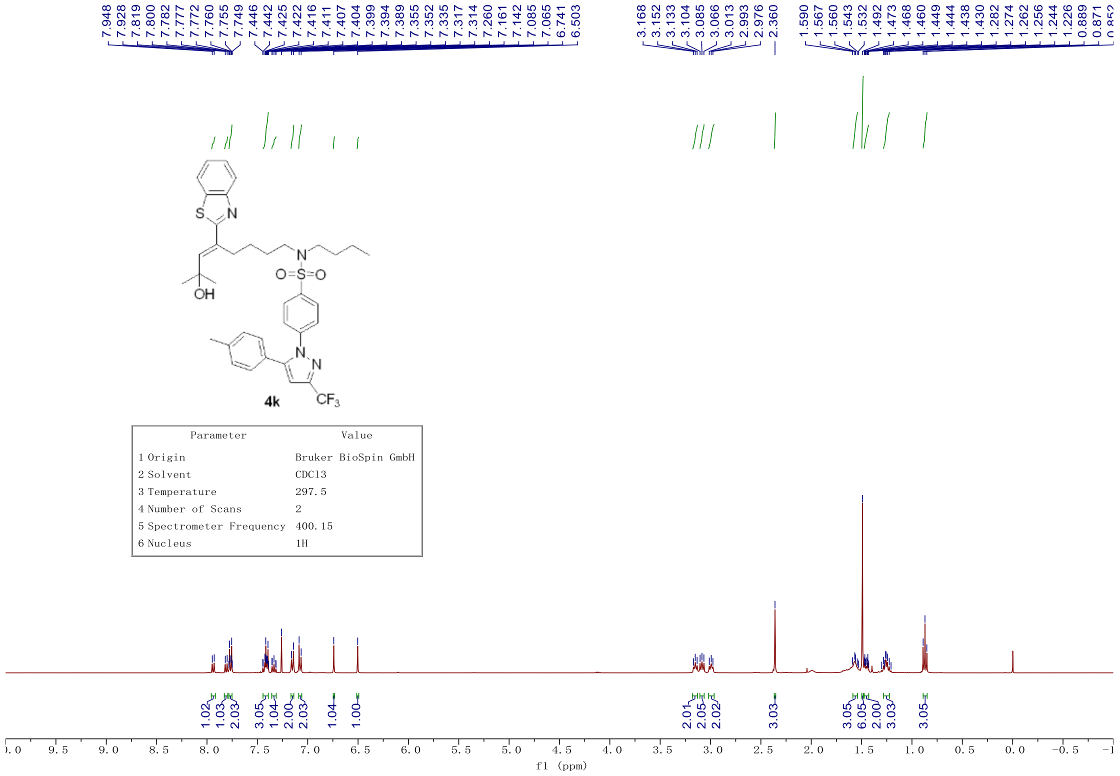

Figure S173. ^{1}H-NMR of **4k.**

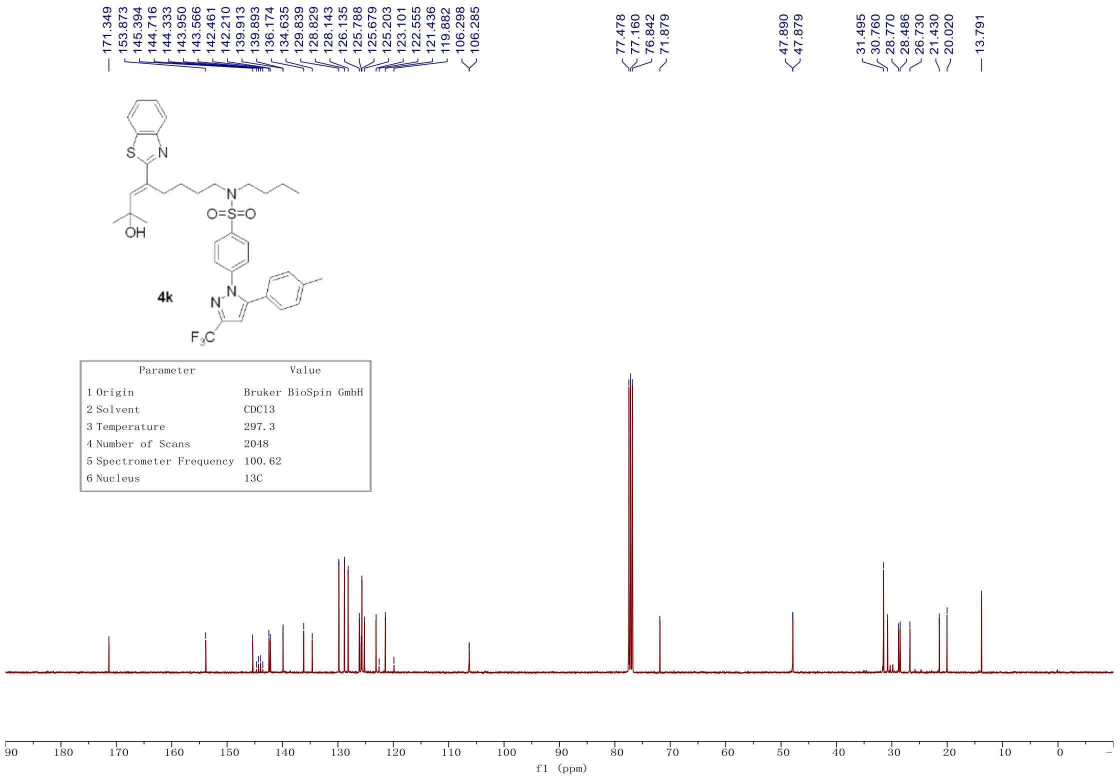

Figure S174. ^{13}C-NMR of **4k.**

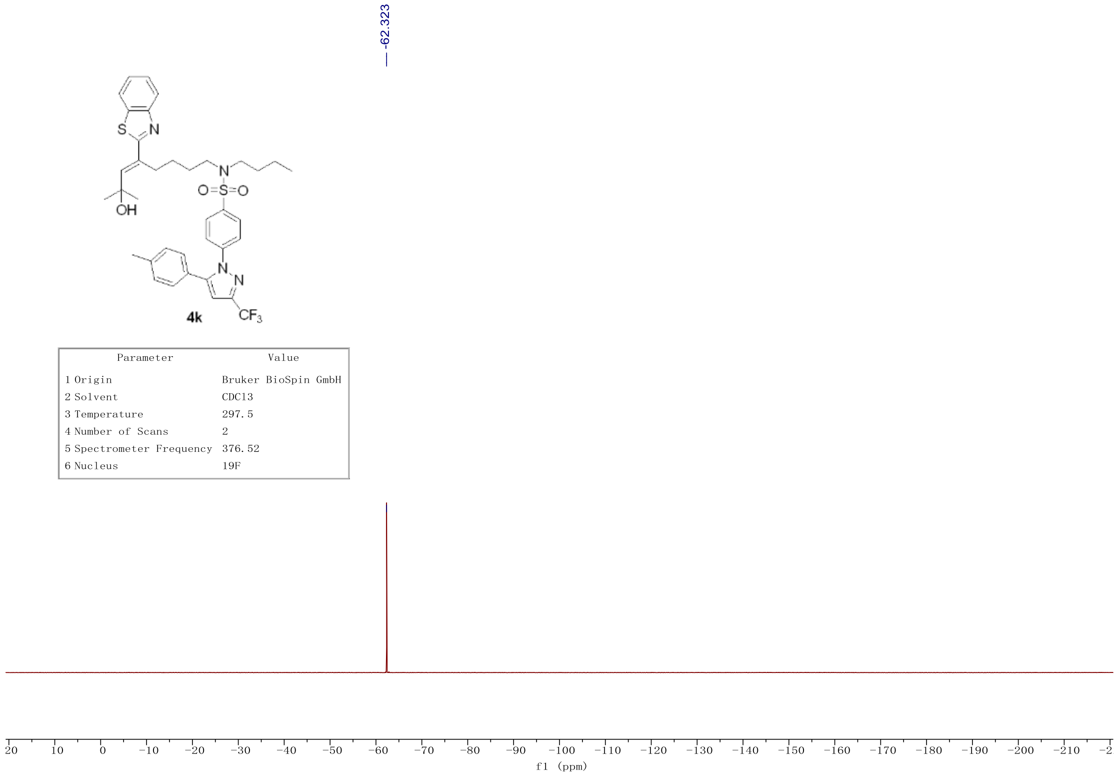

Figure S175. ^{19}F-NMR of **4k.**

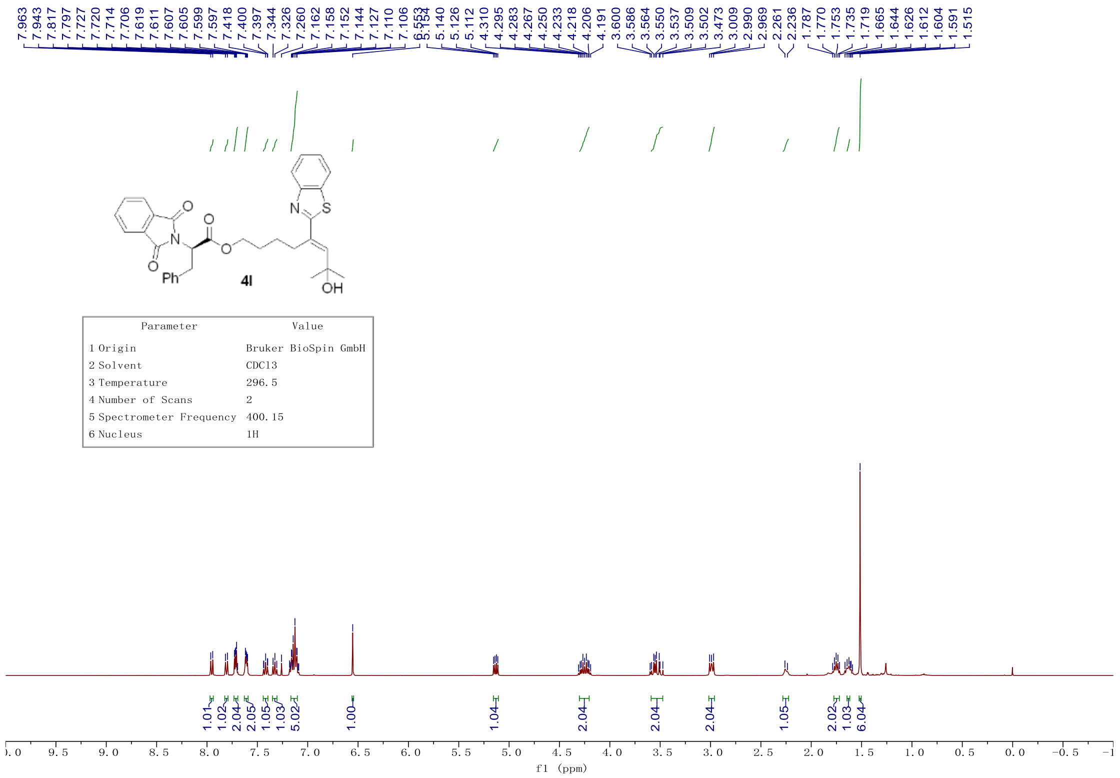

Figure S176. ^{1}H-NMR of **4l.**

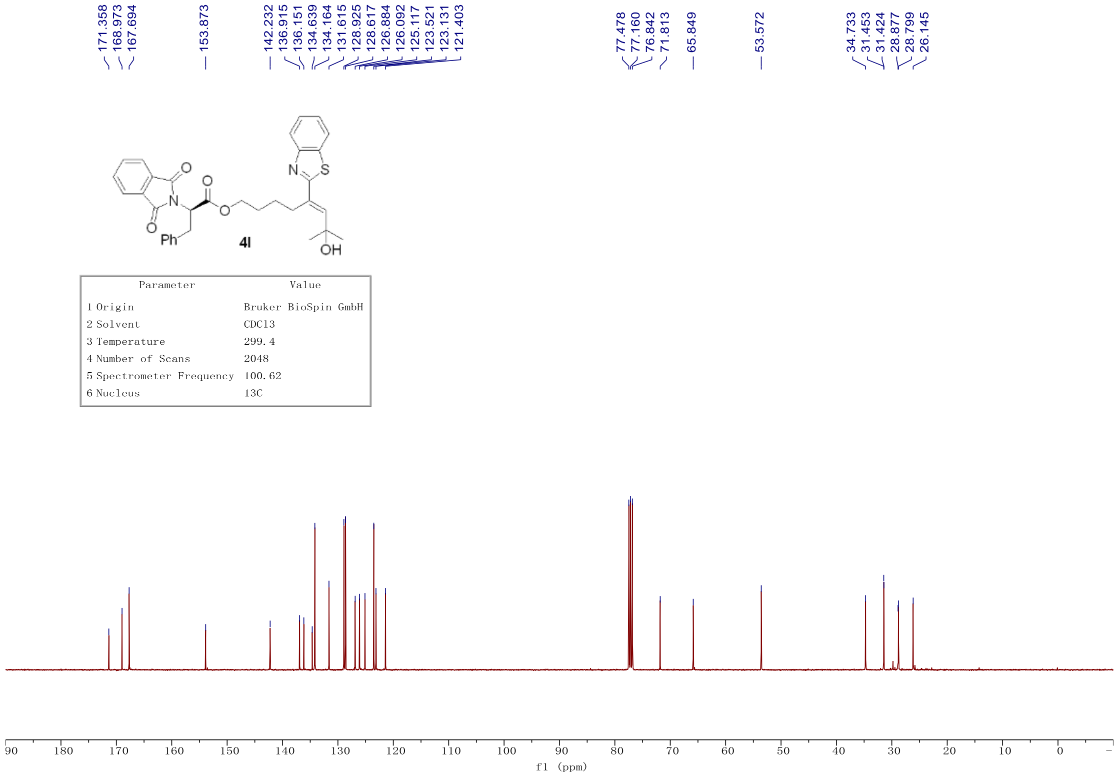

Figure S177. ^{13}C-NMR of **4l.**

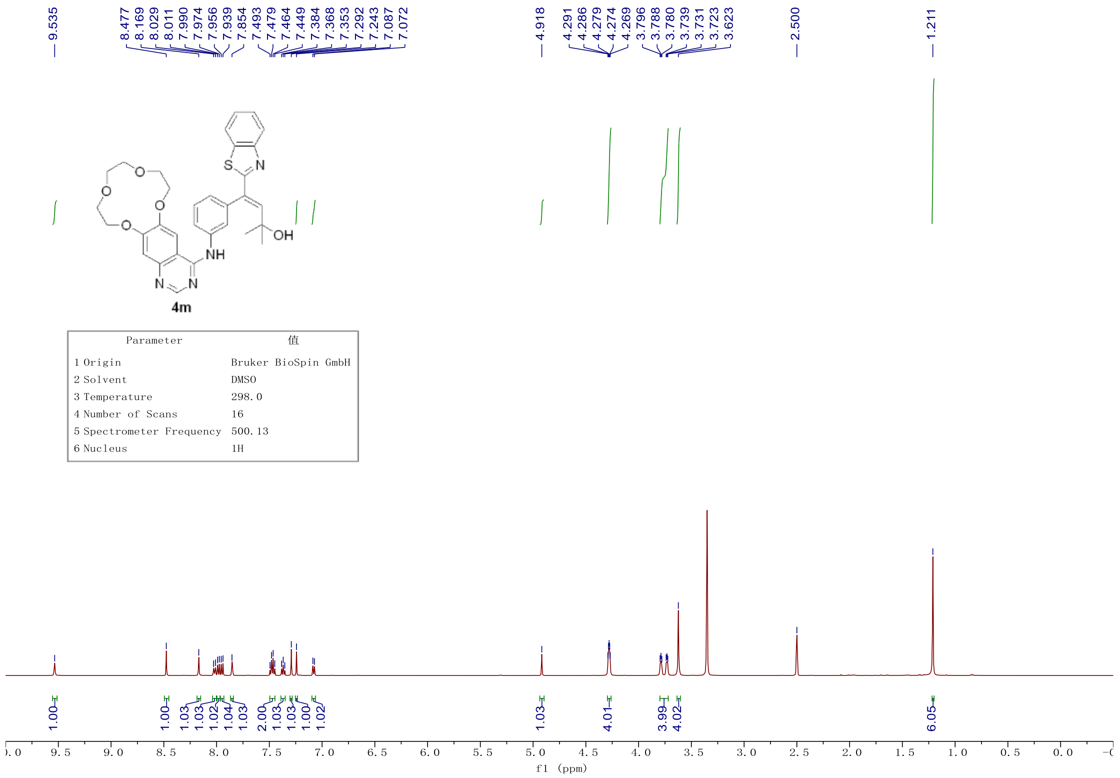

Figure S178. ^{1}H-NMR of **4m.**

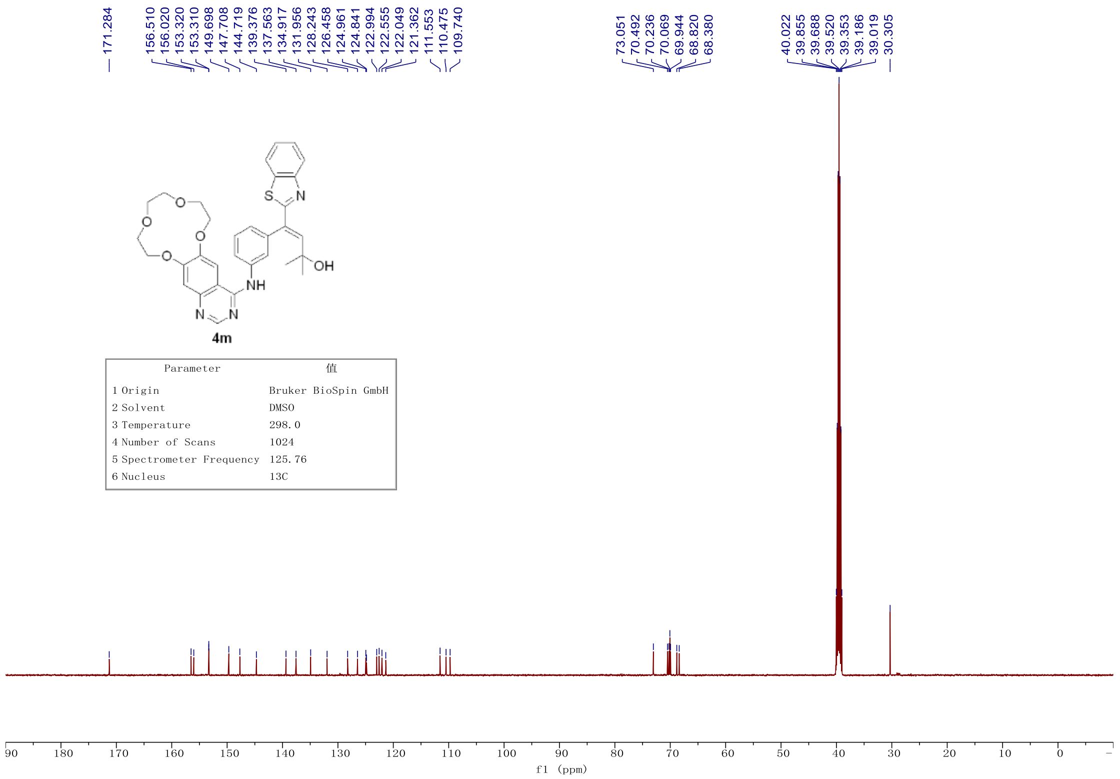

Figure S179. ^{13}C-NMR of **4m.**

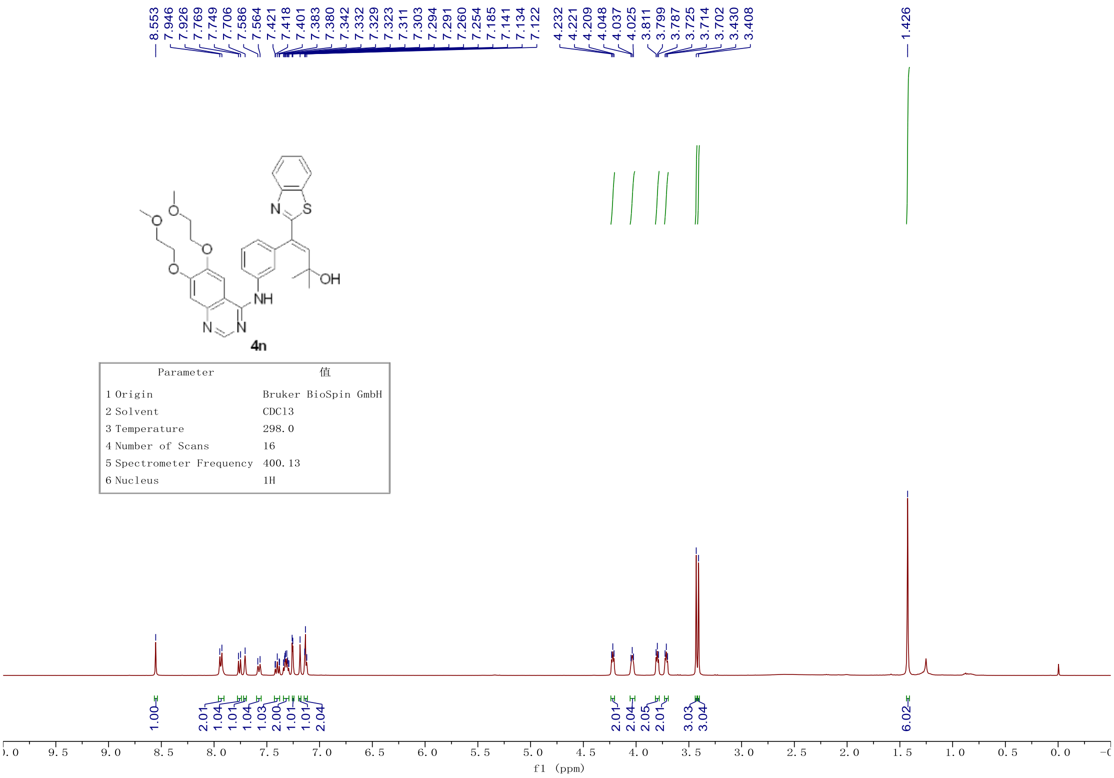

Figure S180. ^{1}H-NMR of **4n.**

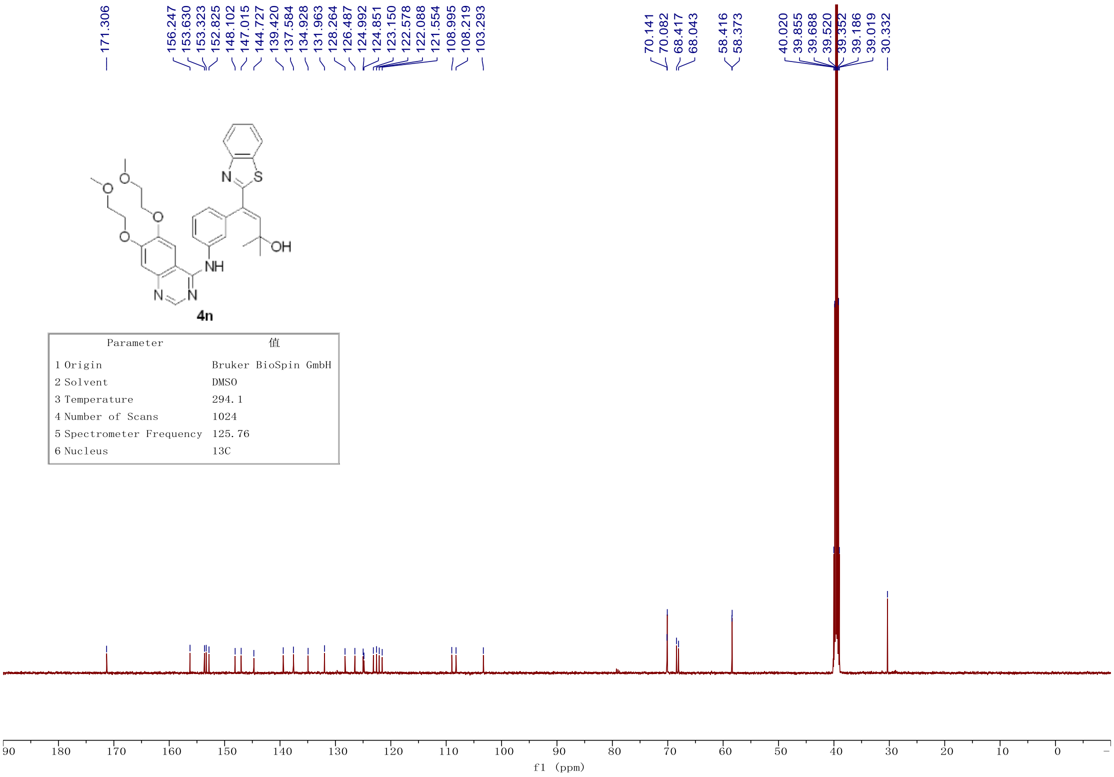

Figure S181. ^{13}C-NMR of **4n.**

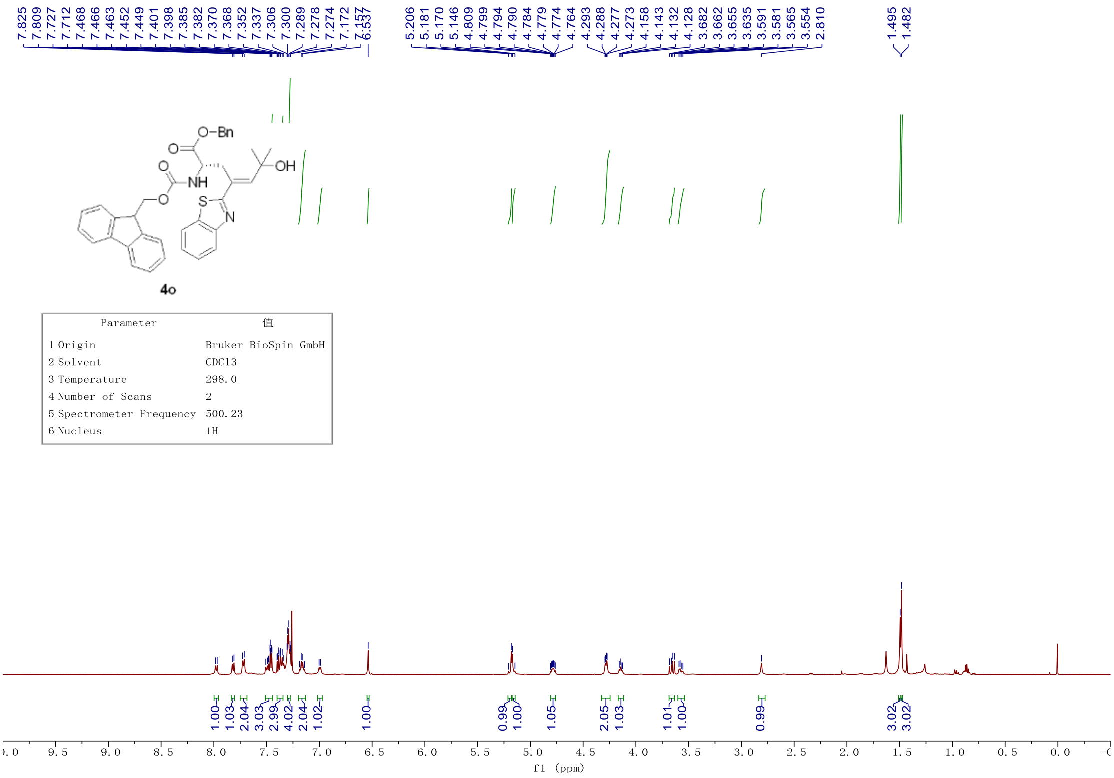

Figure S182. ^{1}H-NMR of **4o.**

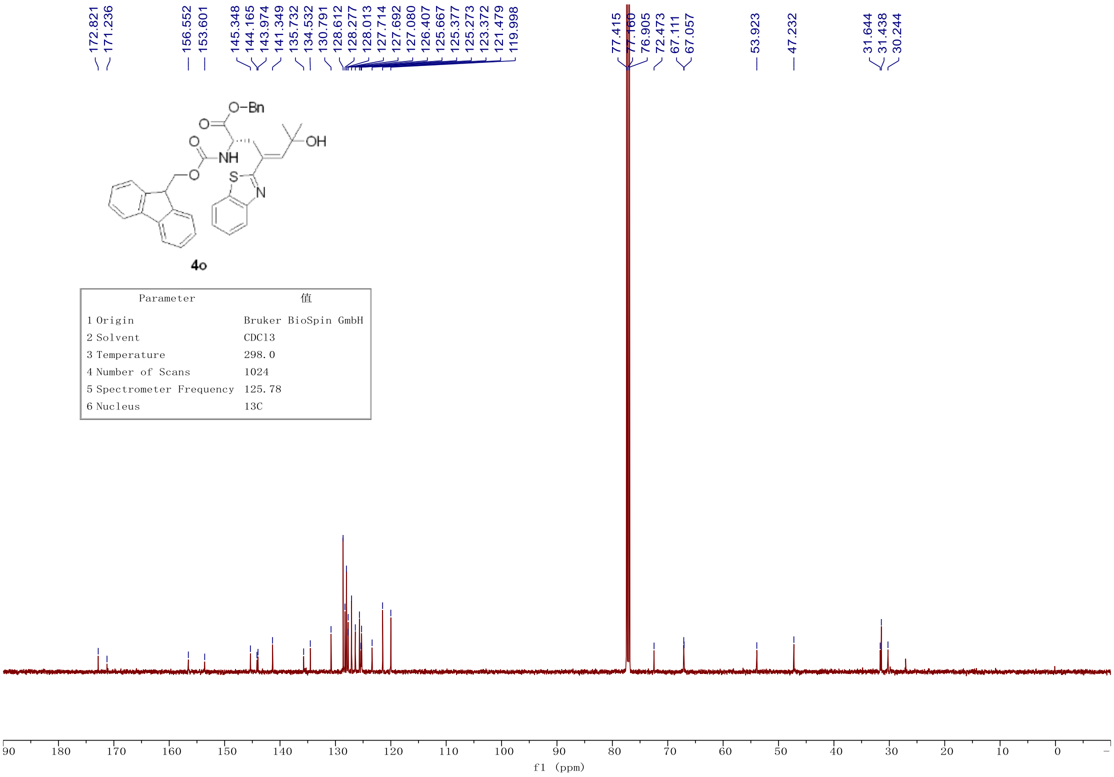

Figure S183. ^{13}C-NMR of **4o.**

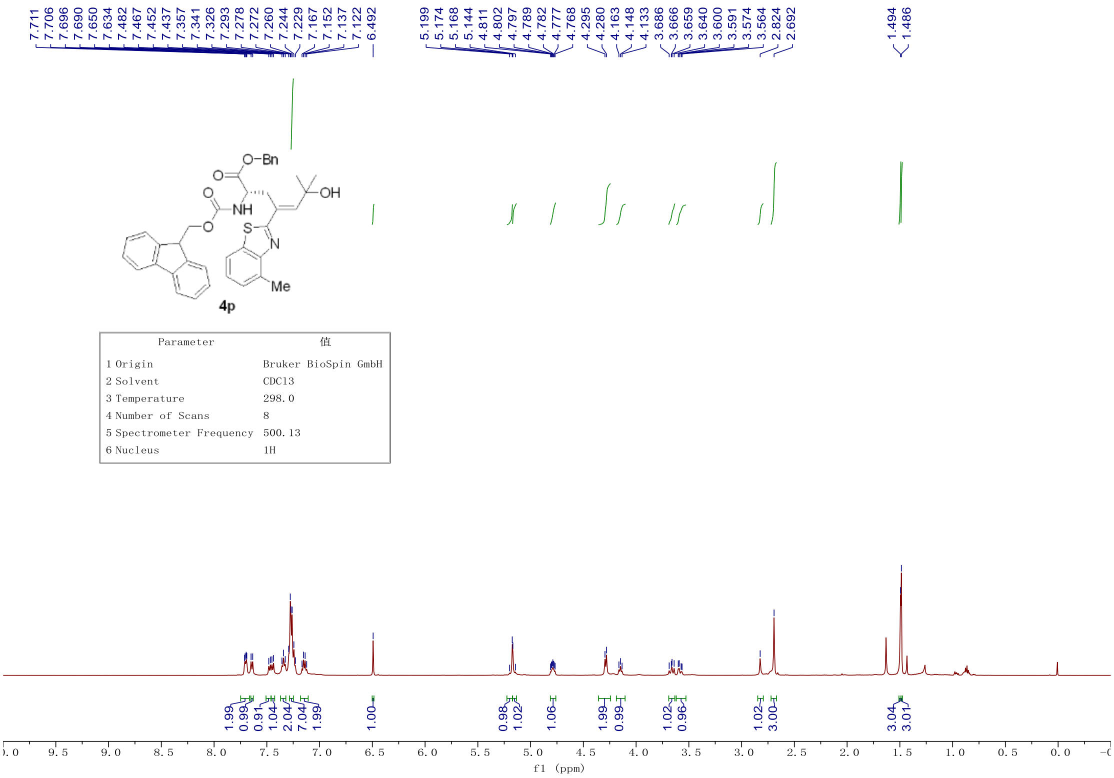

Figure S184. ^{1}H-NMR of **4p.**

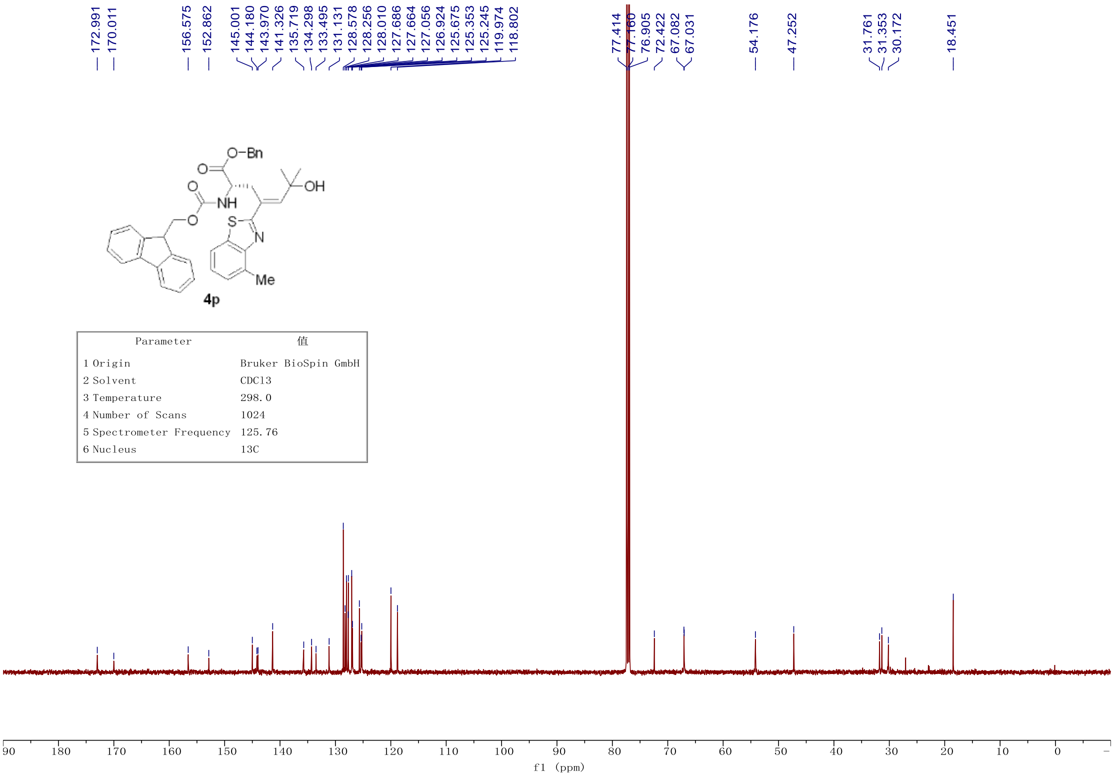

Figure S185. ^{13}C-NMR of **4p.**

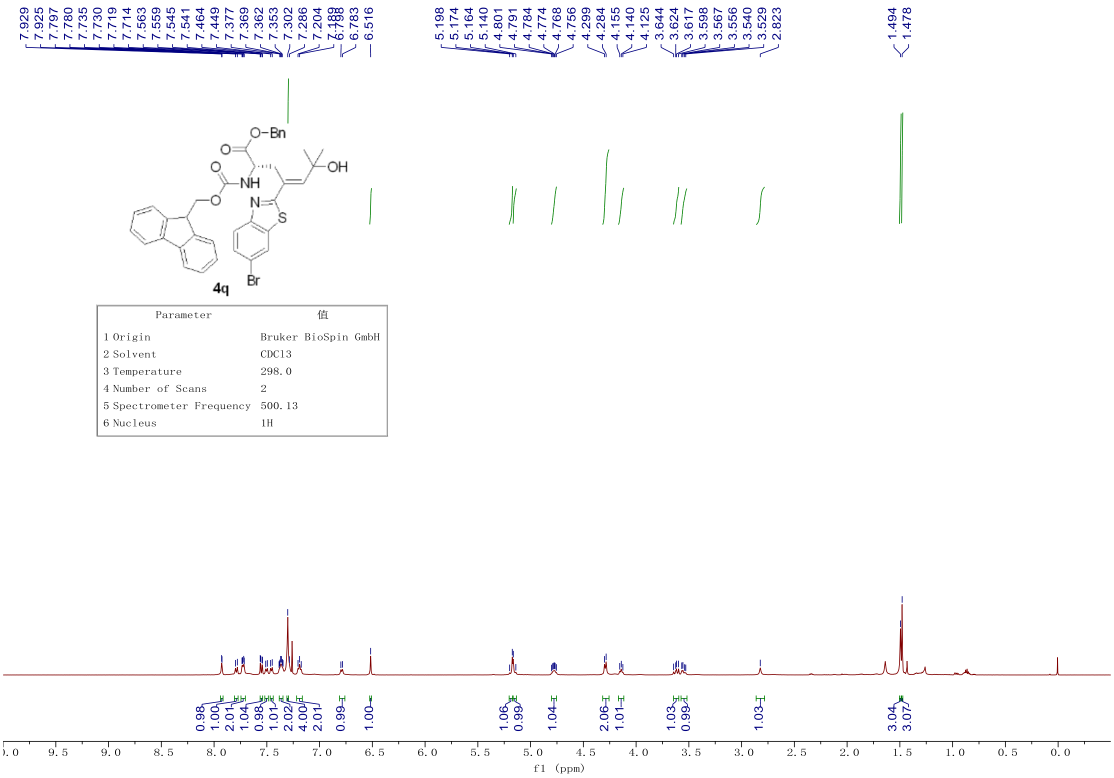

Figure S186. ^{1}H-NMR of **4q.**

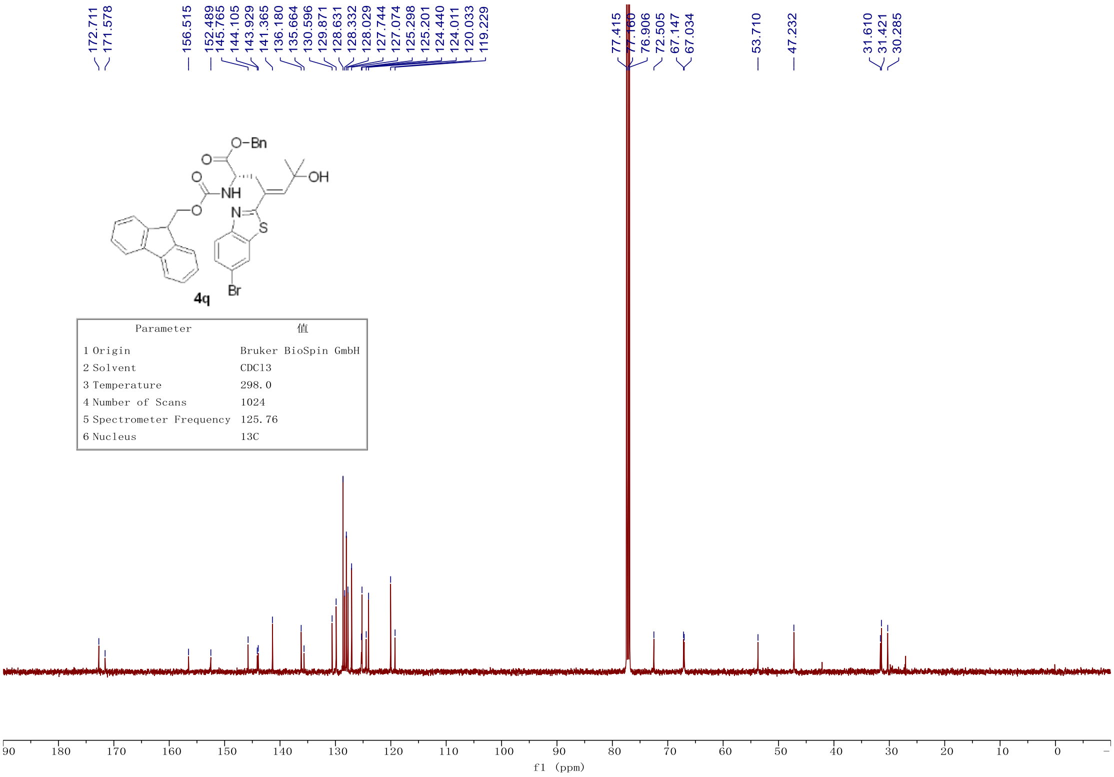

Figure S187. ^{13}C-NMR of **4q.**

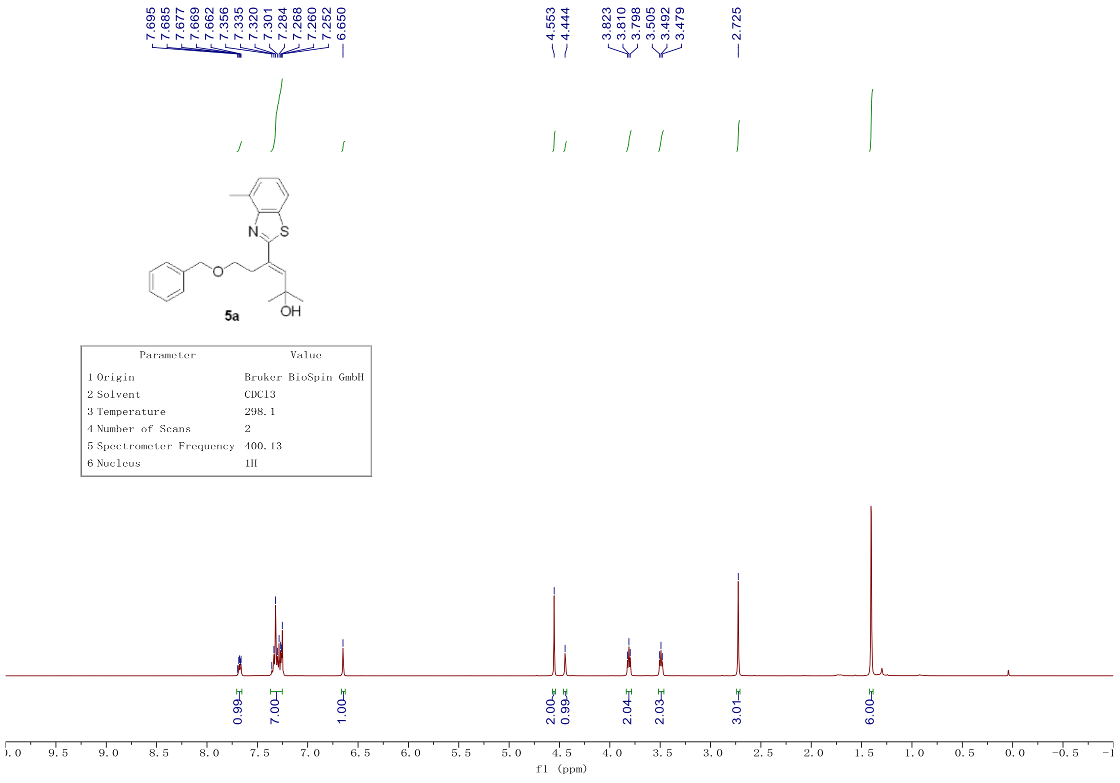

Figure S188. ^{1}H-NMR of **5a.**

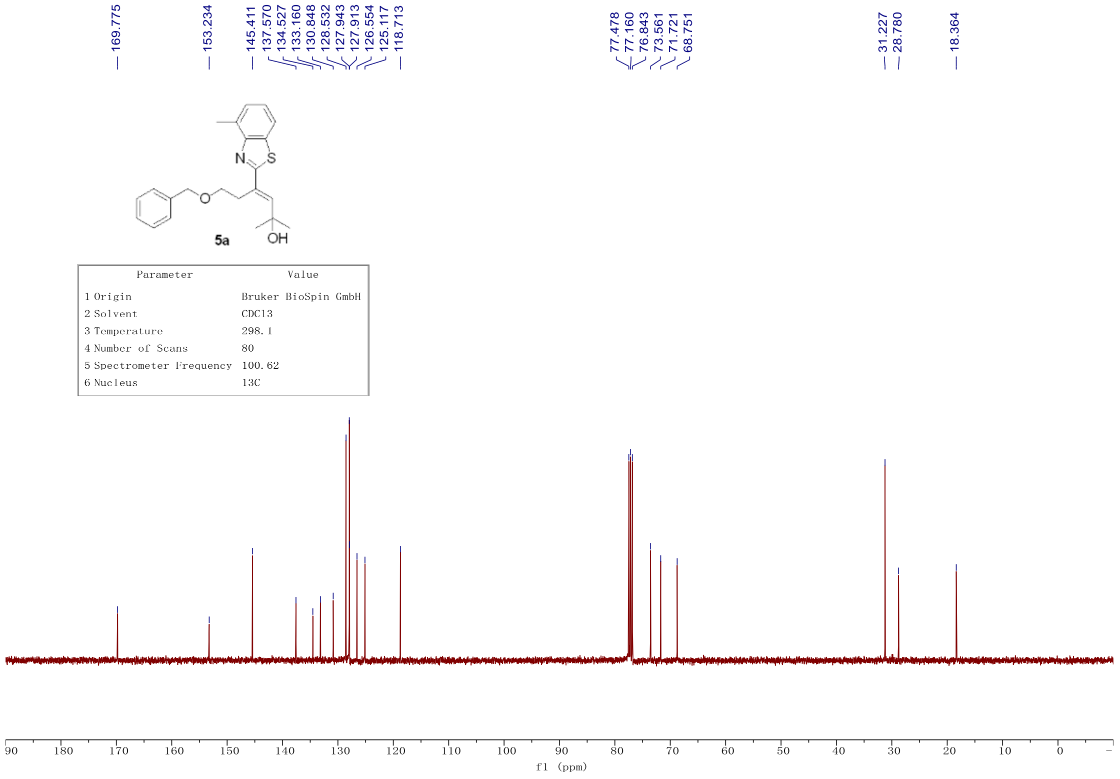

Figure S189. ^{13}C-NMR of **5a.**

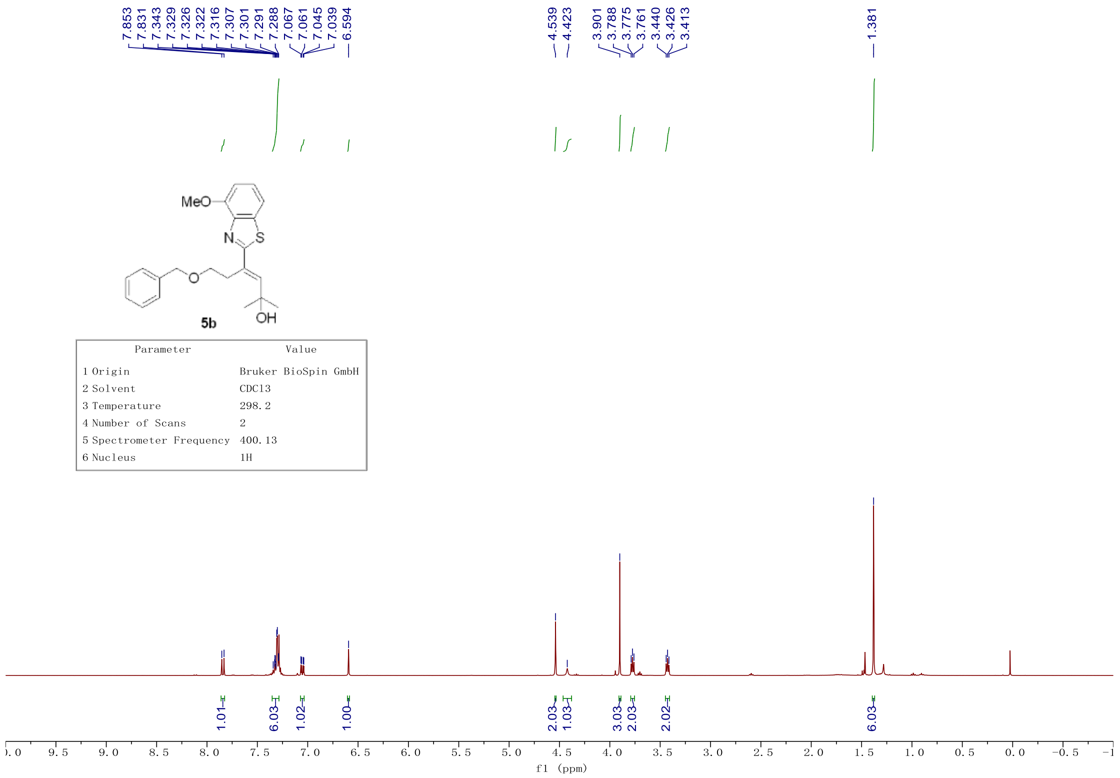

Figure S190. ^{1}H-NMR of **5b.**

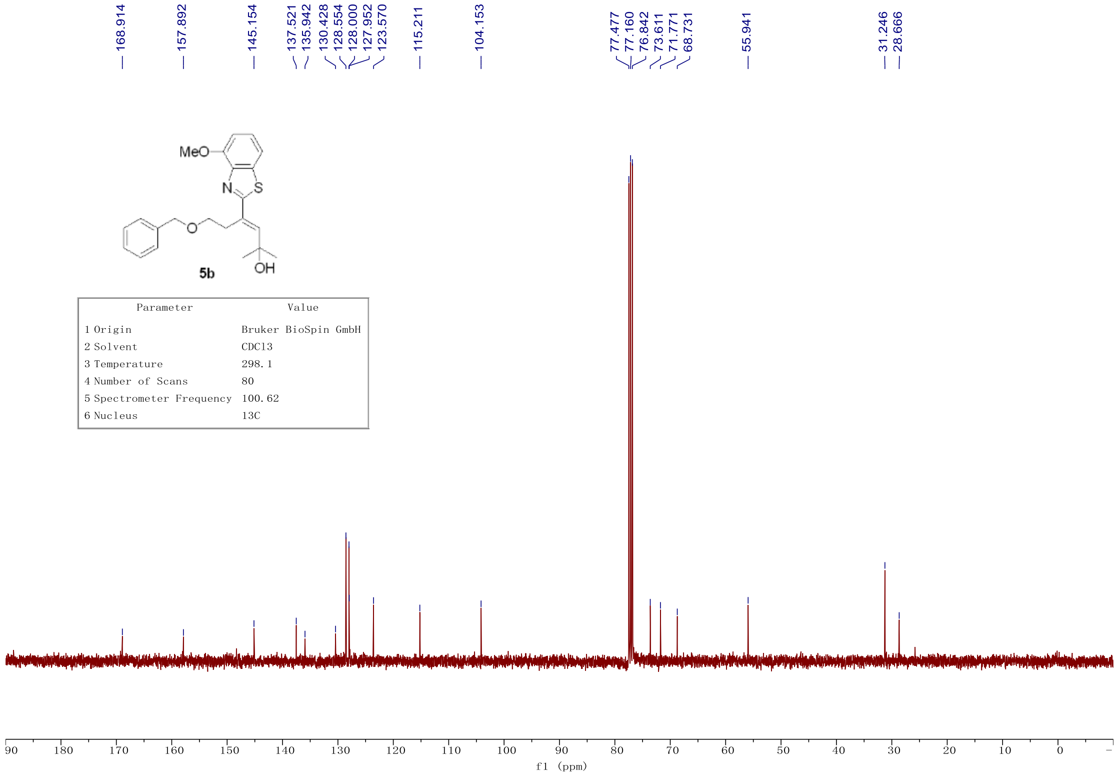

Figure S191. ^{13}C-NMR of **5b.**

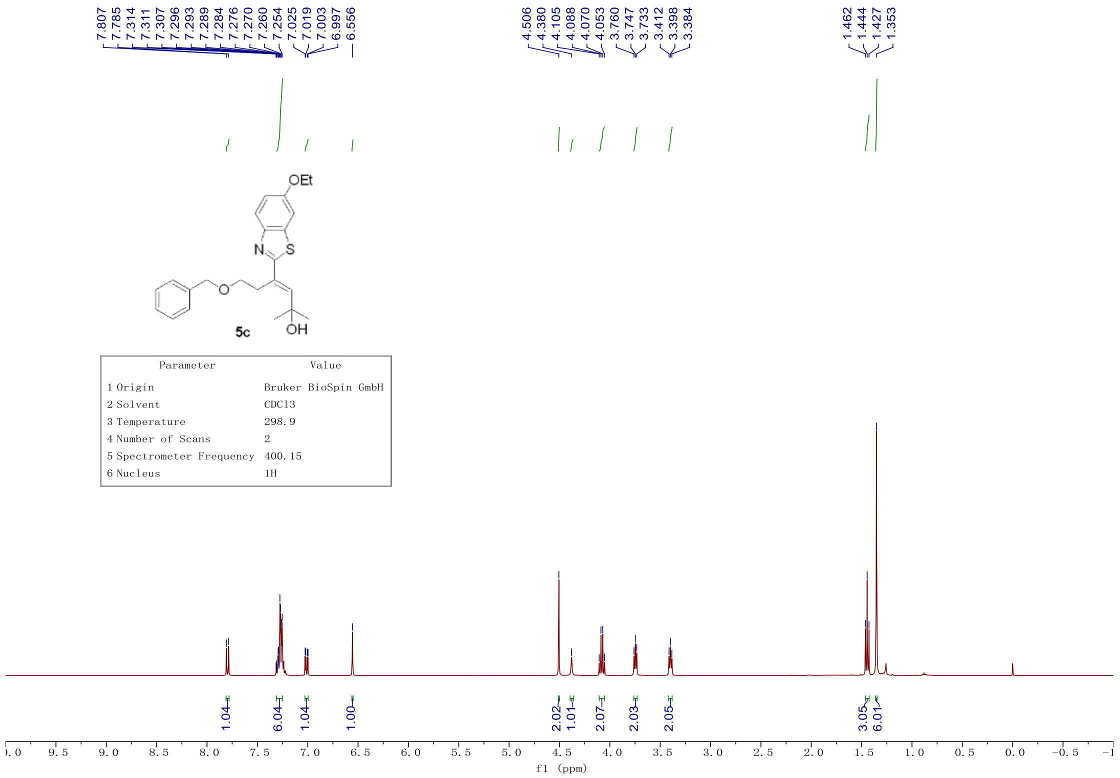

Parameter	Value
1 Origin	Bruker BioSpin GmbH
2 Solvent	CDCl3
3 Temperature	298.9
4 Number of Scans	2
5 Spectrometer Frequency	400.15
6 Nucleus	1H

Figure S192. ^{1}H-NMR of **5c.**

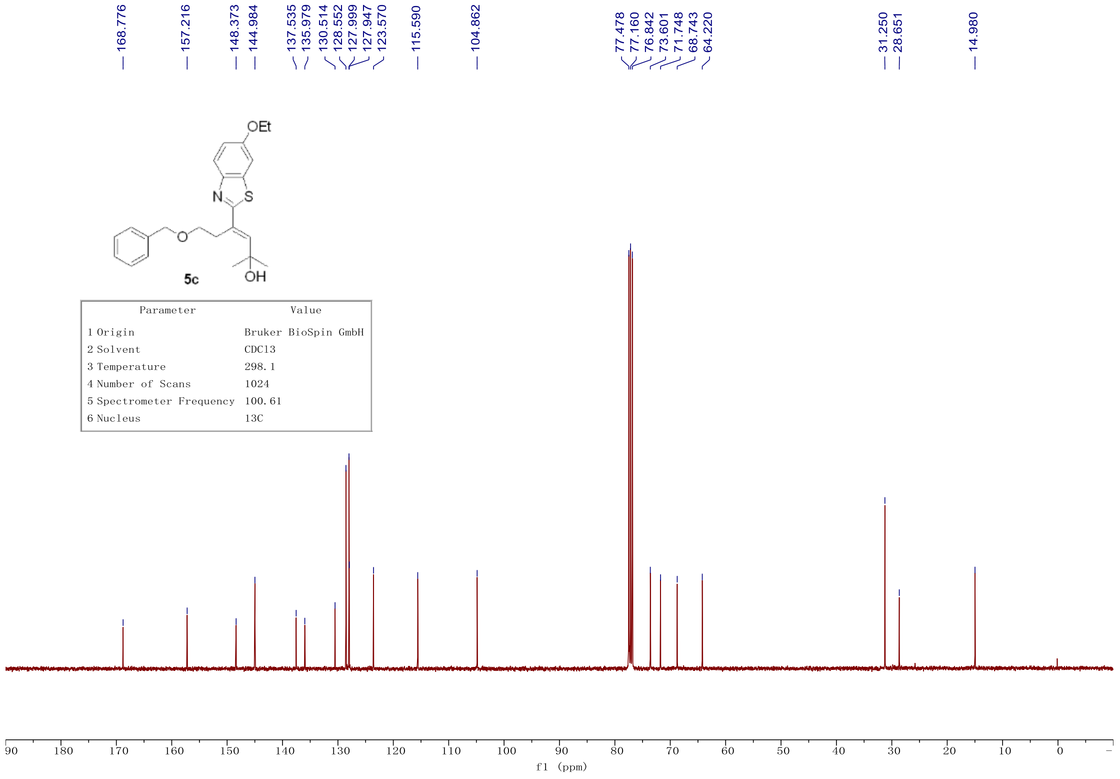

Parameter	Value
1 Origin	Bruker BioSpin GmbH
2 Solvent	CDCl3
3 Temperature	298.1
4 Number of Scans	1024
5 Spectrometer Frequency	100.61
6 Nucleus	13C

Figure S193. ^{13}C-NMR of **5c.**

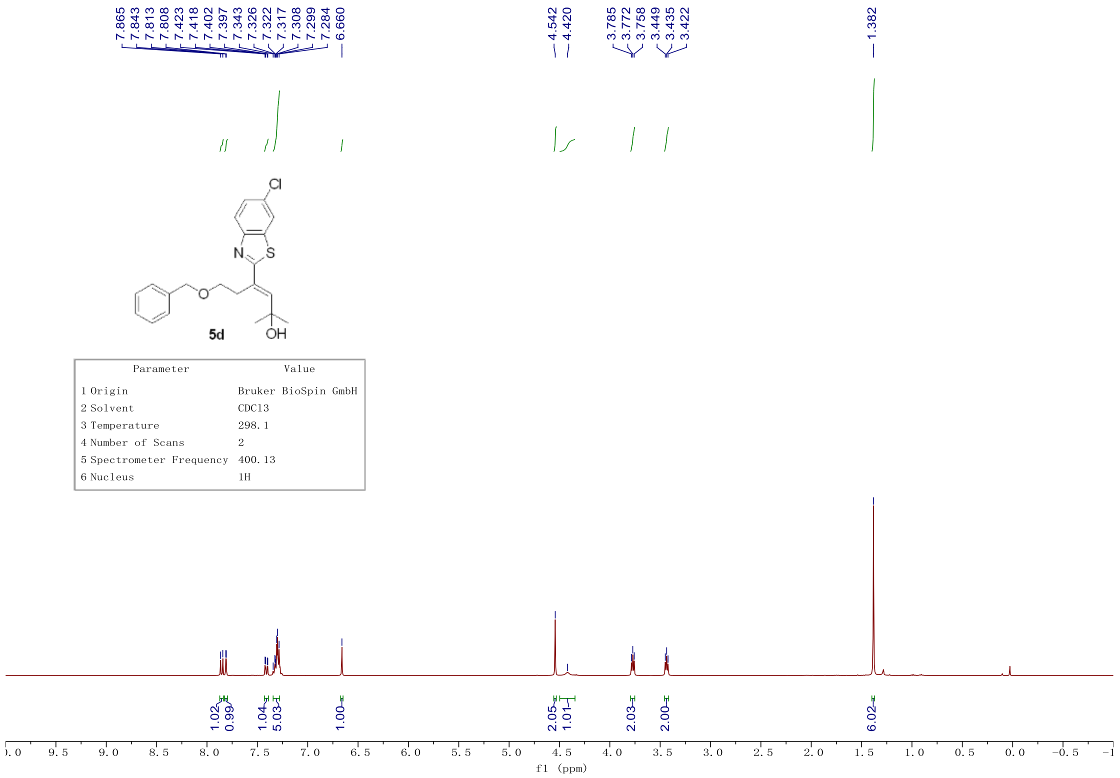

Figure S194. ^{1}H-NMR of **5d.**

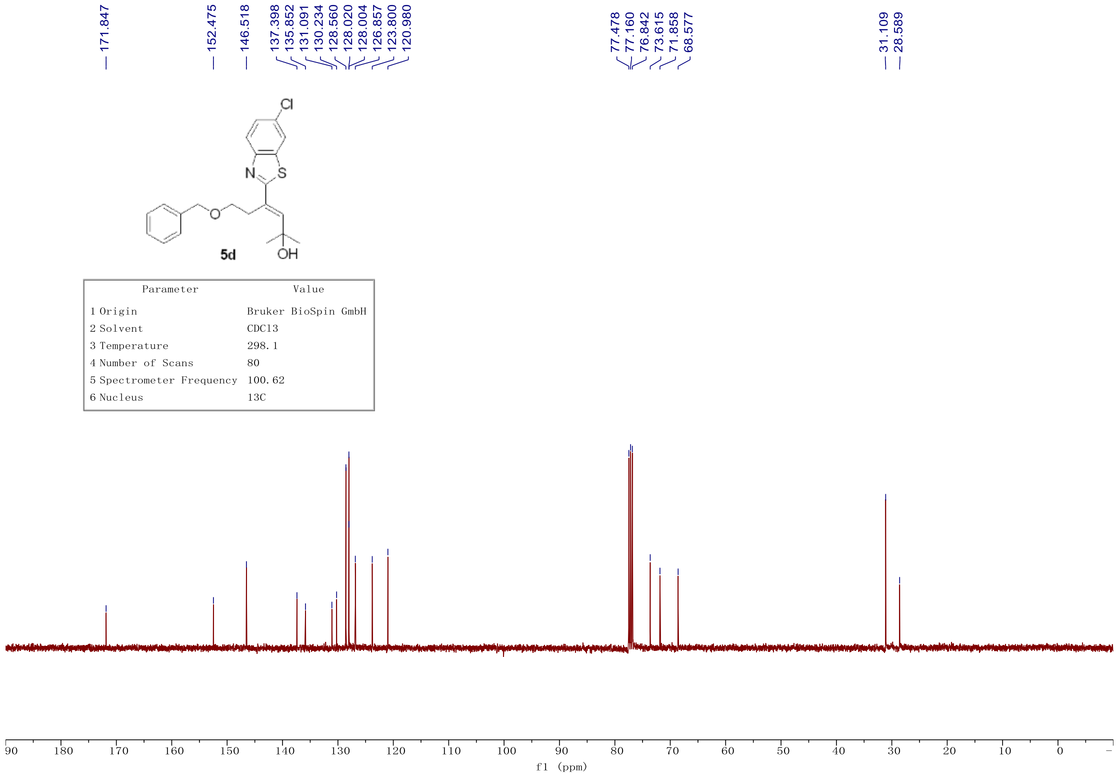

Figure S195. ^{13}C-NMR of **5d.**

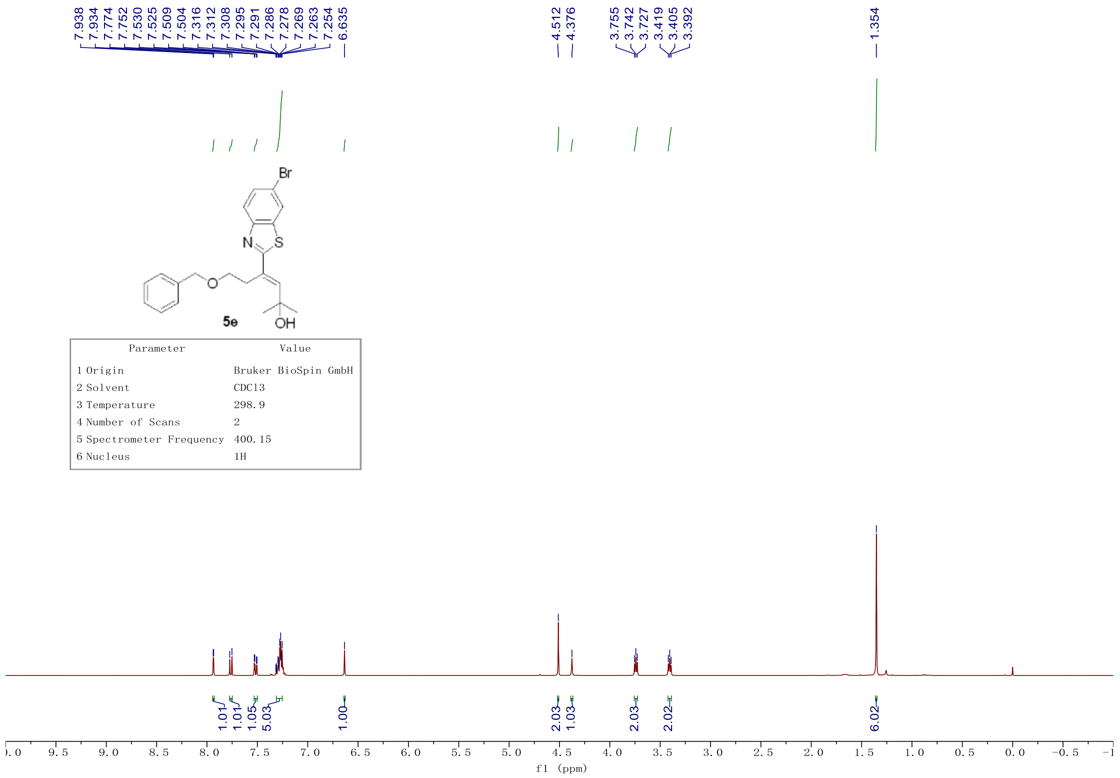

Figure S196. ^{1}H-NMR of **5e.**

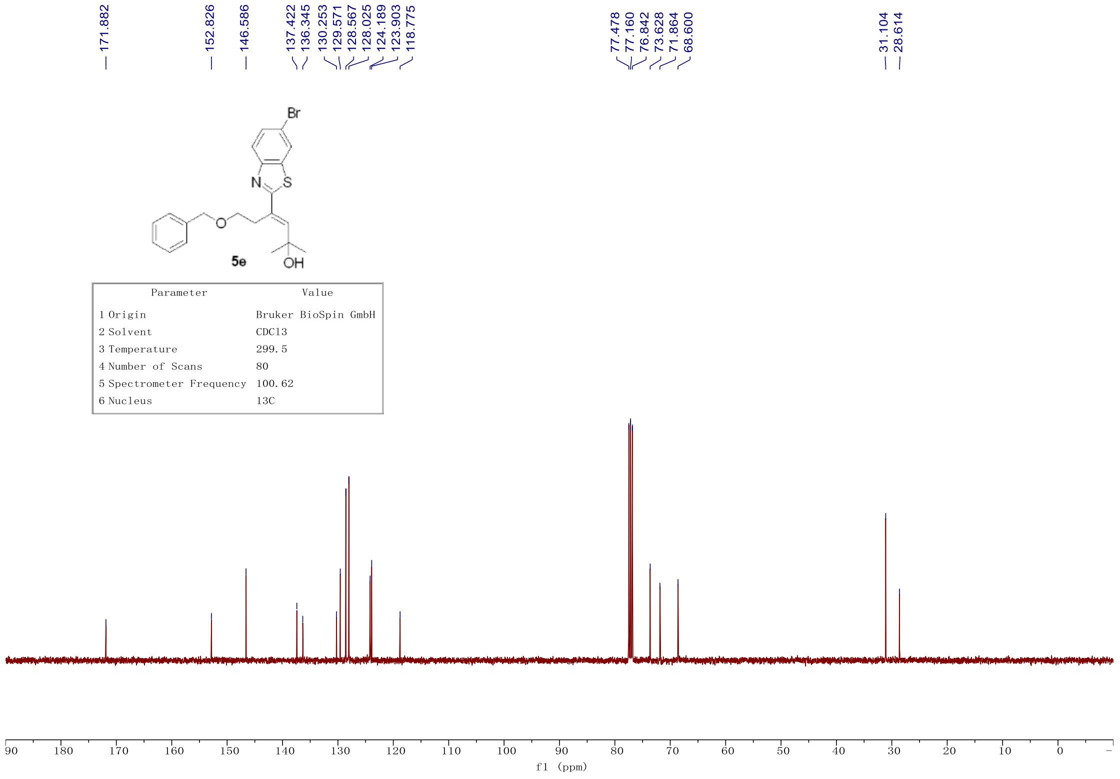

Figure S197. ^{13}C-NMR of **5e.**

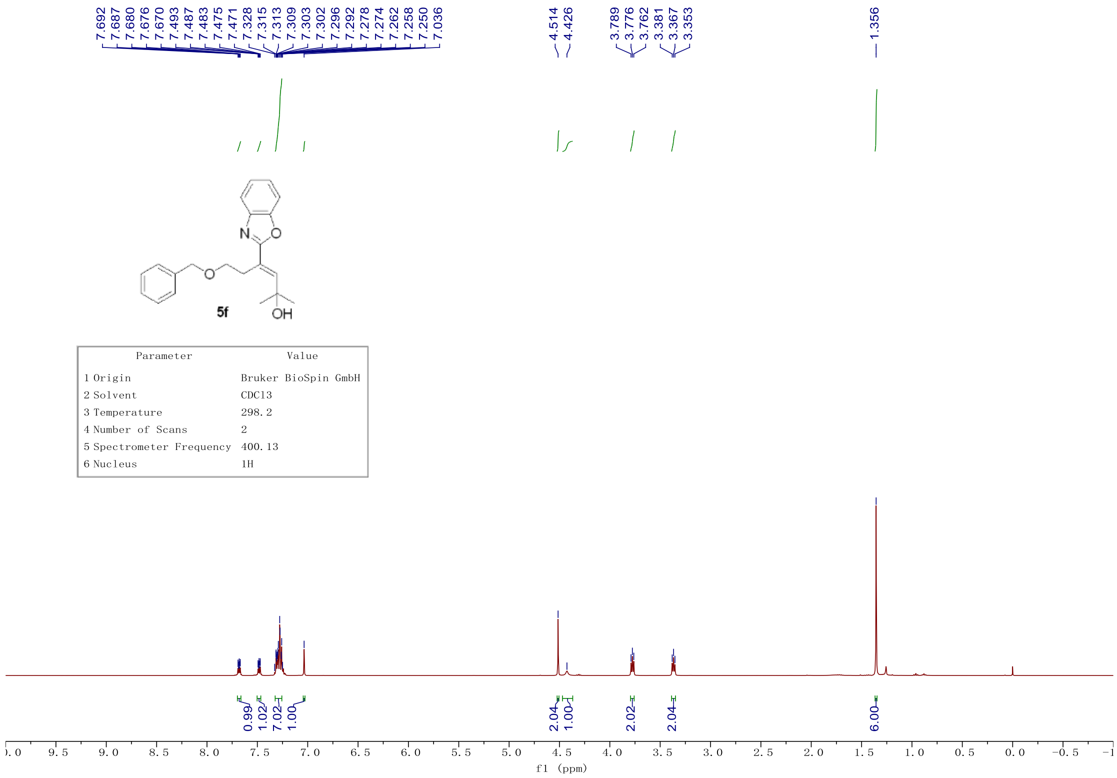

Figure S198. ^{1}H-NMR of **5f.**

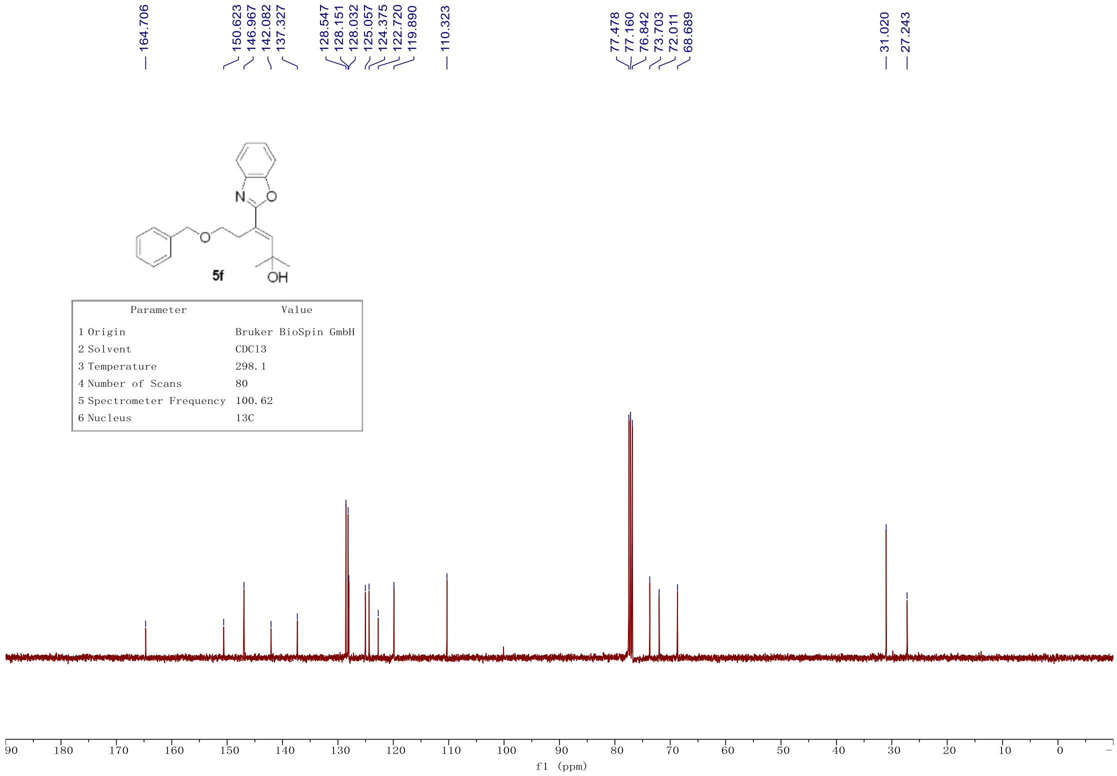

Figure S199. ^{13}C-NMR of **5f.**

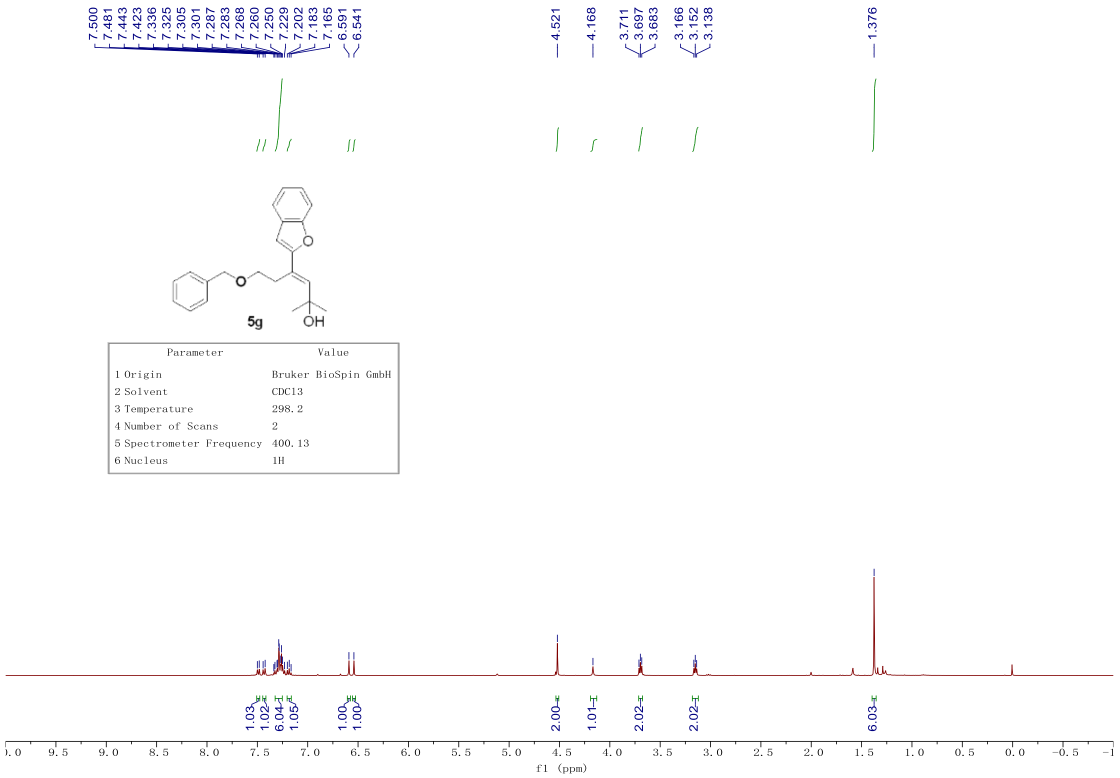

Parameter	Value
1 Origin	Bruker BioSpin GmbH
2 Solvent	CDCl3
3 Temperature	298.2
4 Number of Scans	2
5 Spectrometer Frequency	400.13
6 Nucleus	1H

Figure S200. ^{1}H-NMR of **5g.**

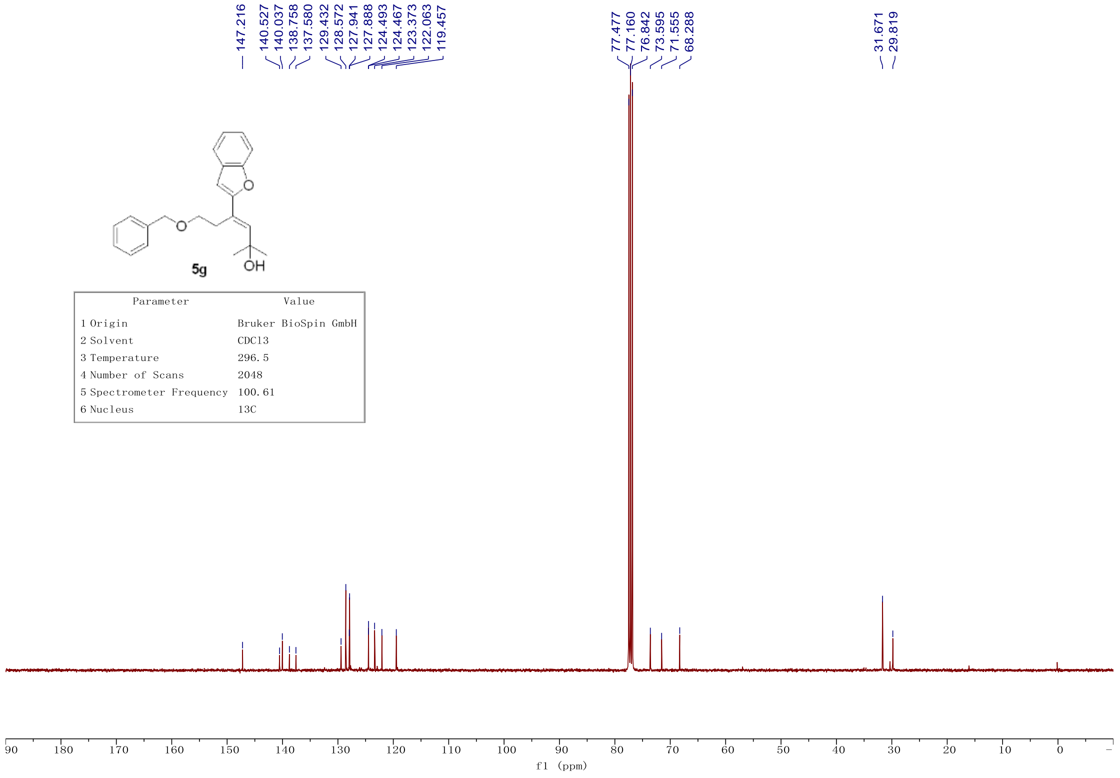

Parameter	Value
1 Origin	Bruker BioSpin GmbH
2 Solvent	CDCl3
3 Temperature	296.5
4 Number of Scans	2048
5 Spectrometer Frequency	100.61
6 Nucleus	13C

Figure S201. ^{13}C-NMR of **5g.**

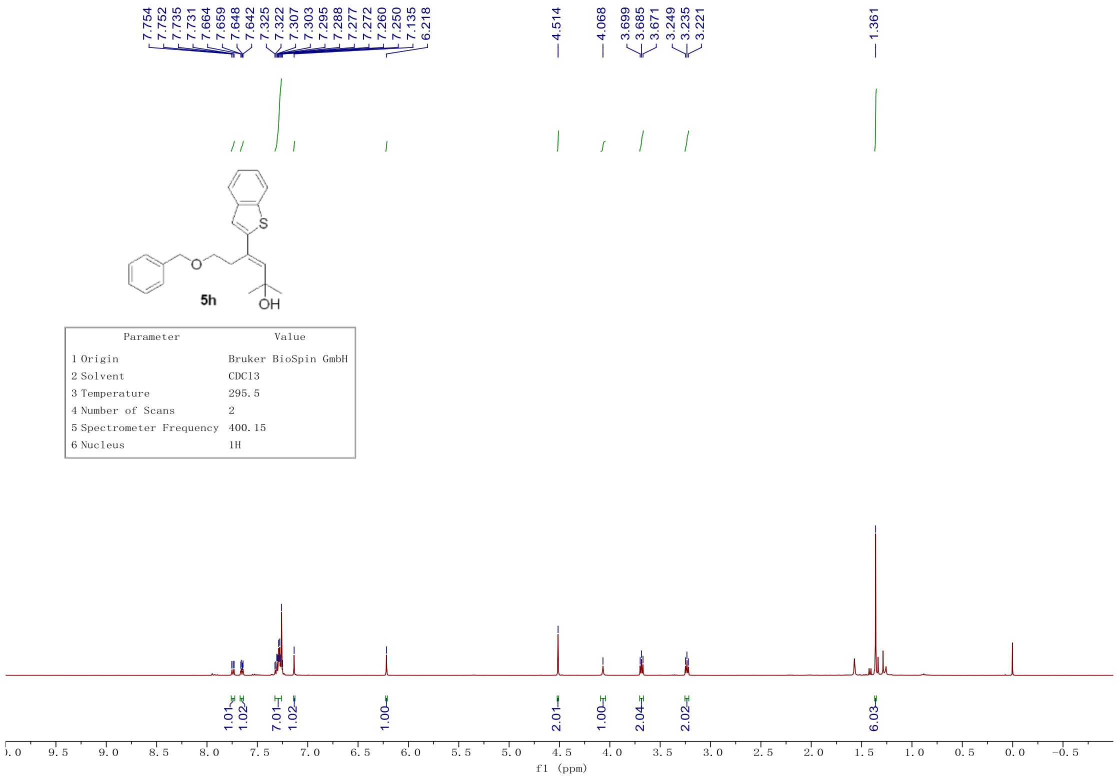

Figure S202. ^{1}H-NMR of **5h.**

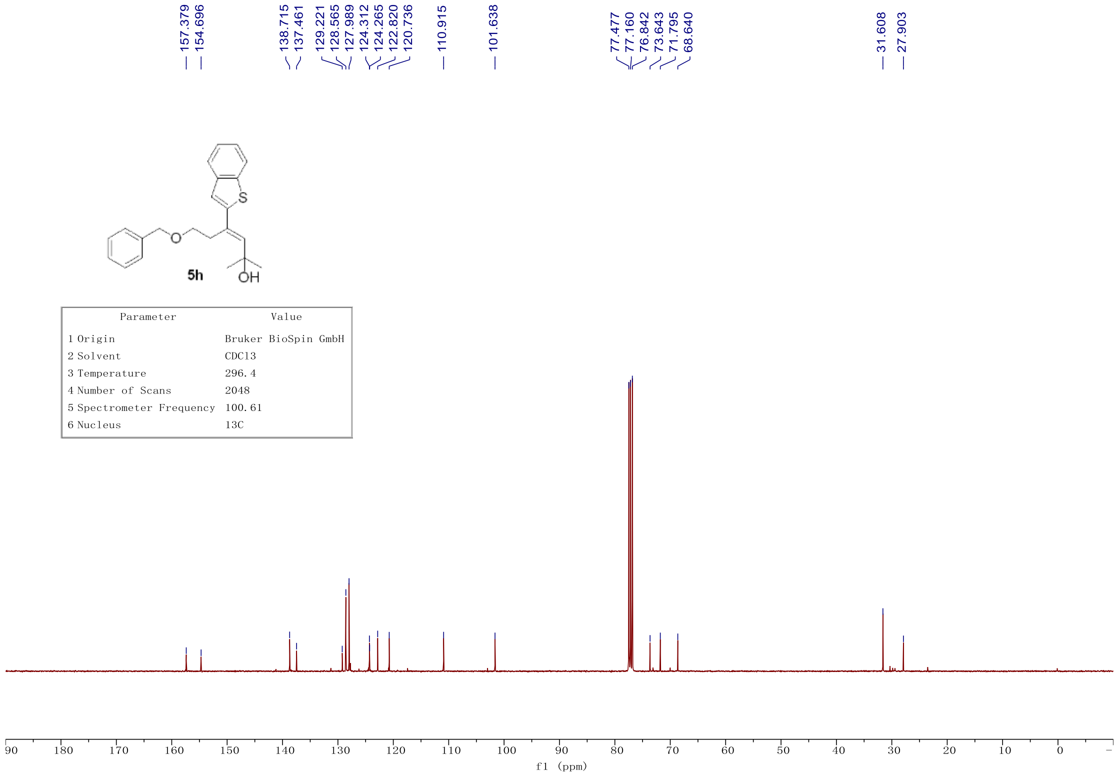

Figure S203. ^{13}C-NMR of **5h.**

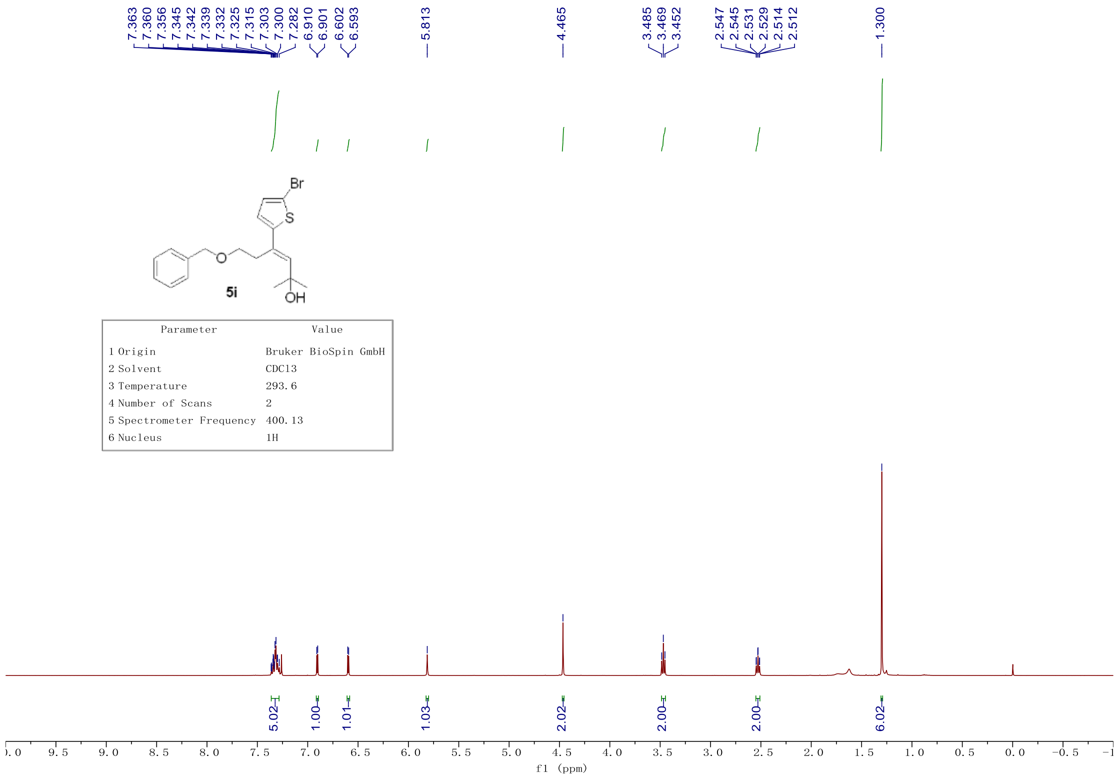

Figure S204. ^{1}H-NMR of **5i.**

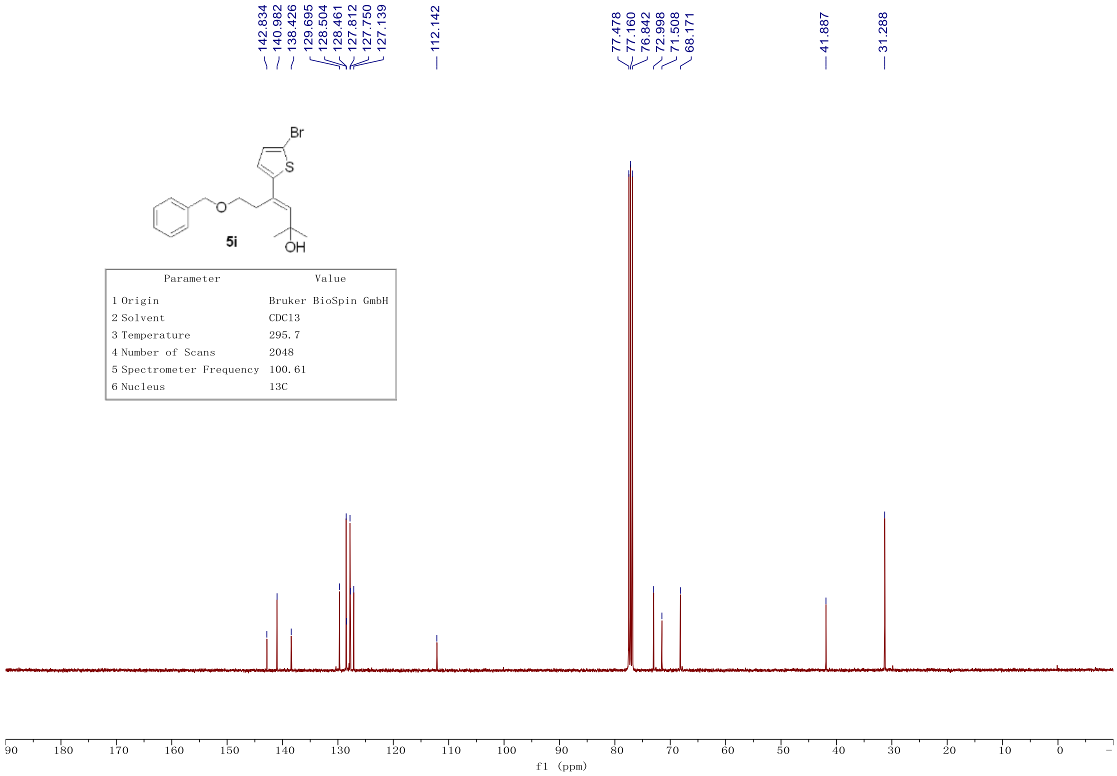

Figure S205. ^{13}C-NMR of **5i.**

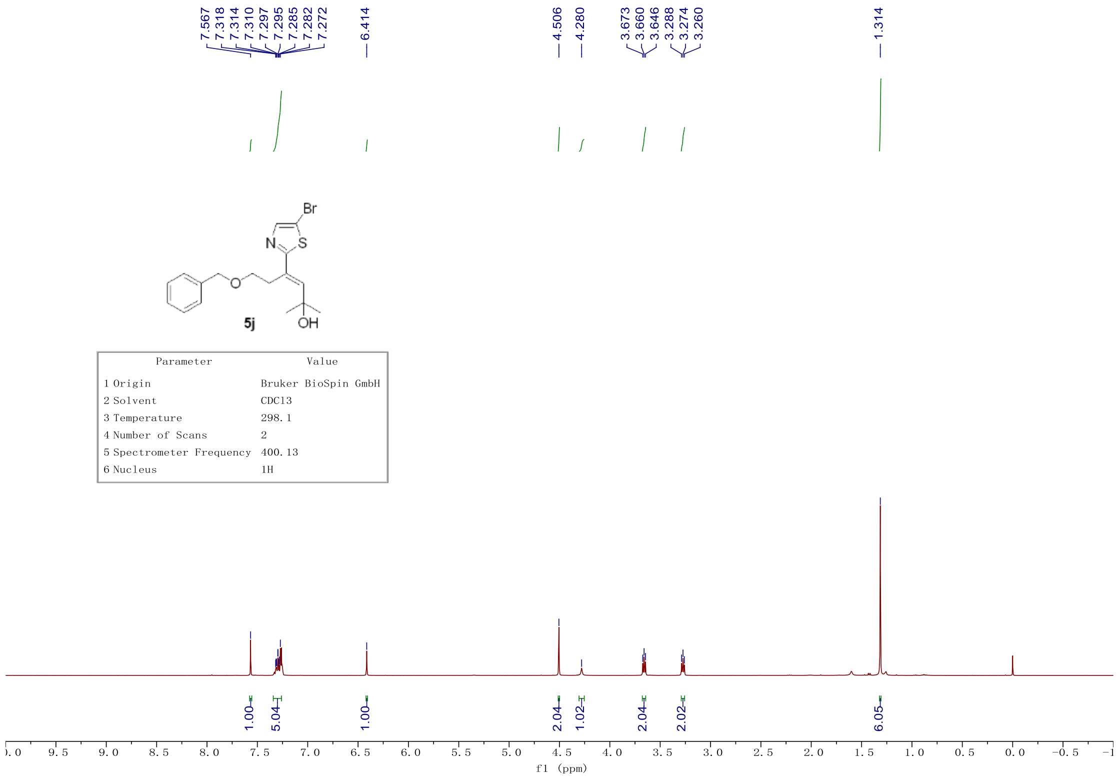

Figure S206. ^{1}H-NMR of **5j.**

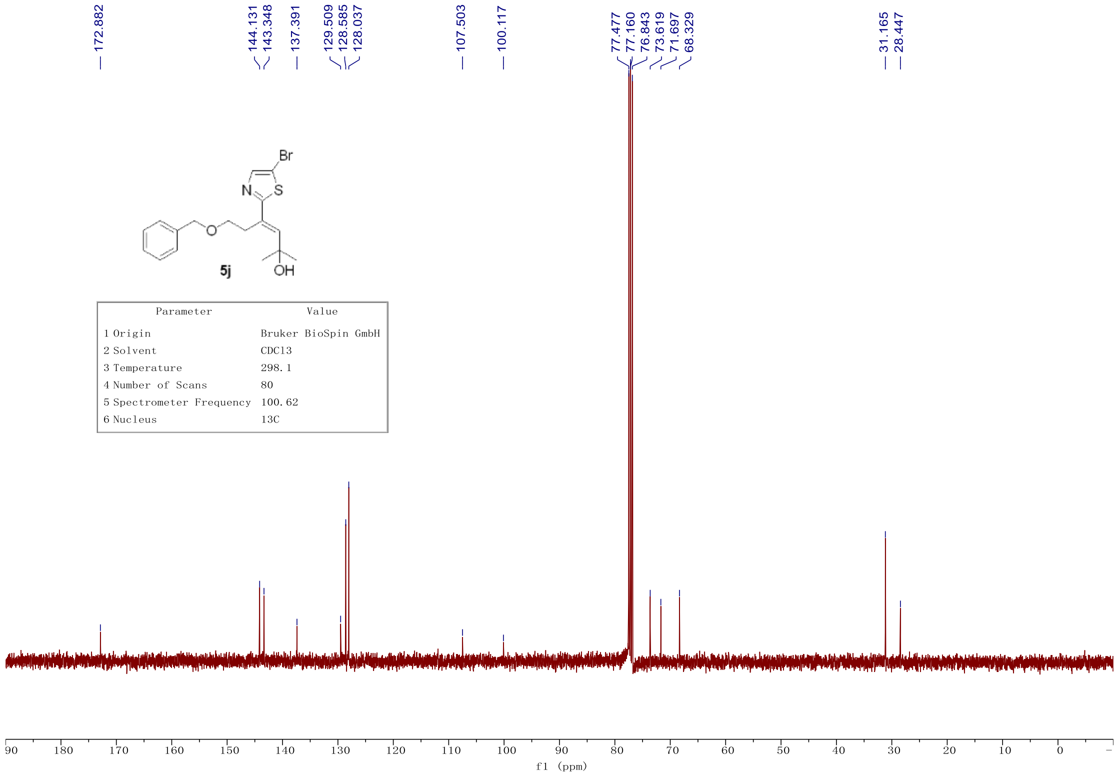

Figure S207. ^{13}C-NMR of **5j.**

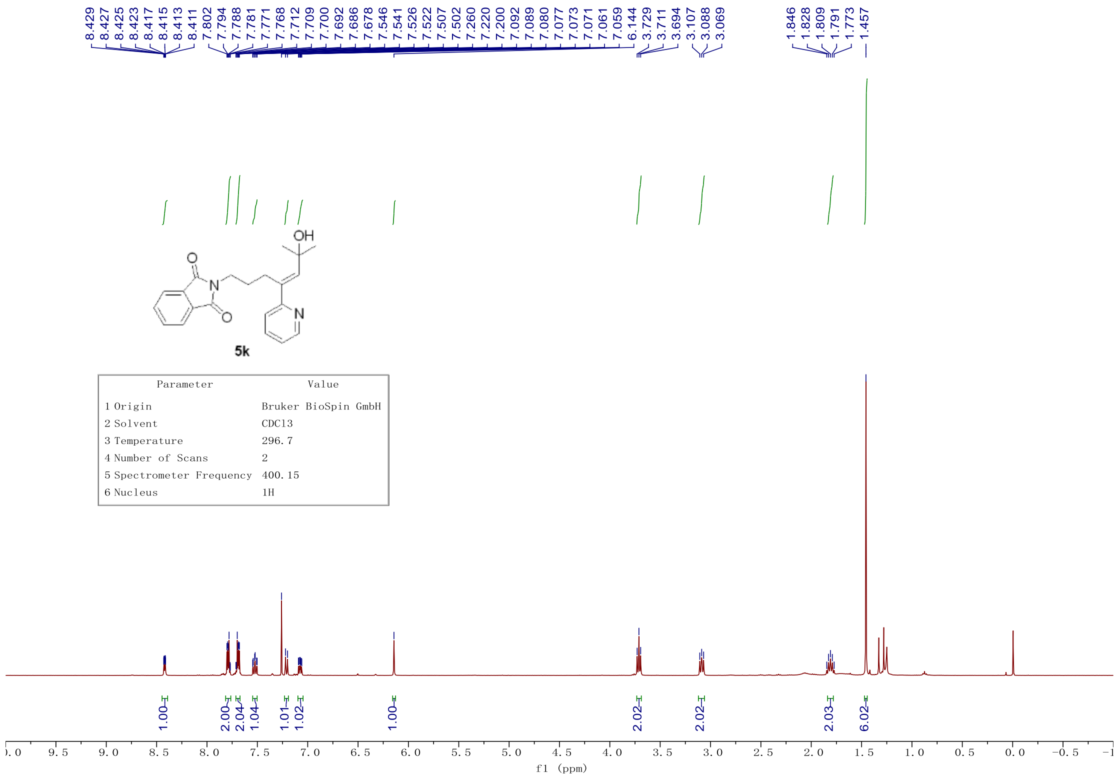

Figure S208. ^{1}H-NMR of **5k.**

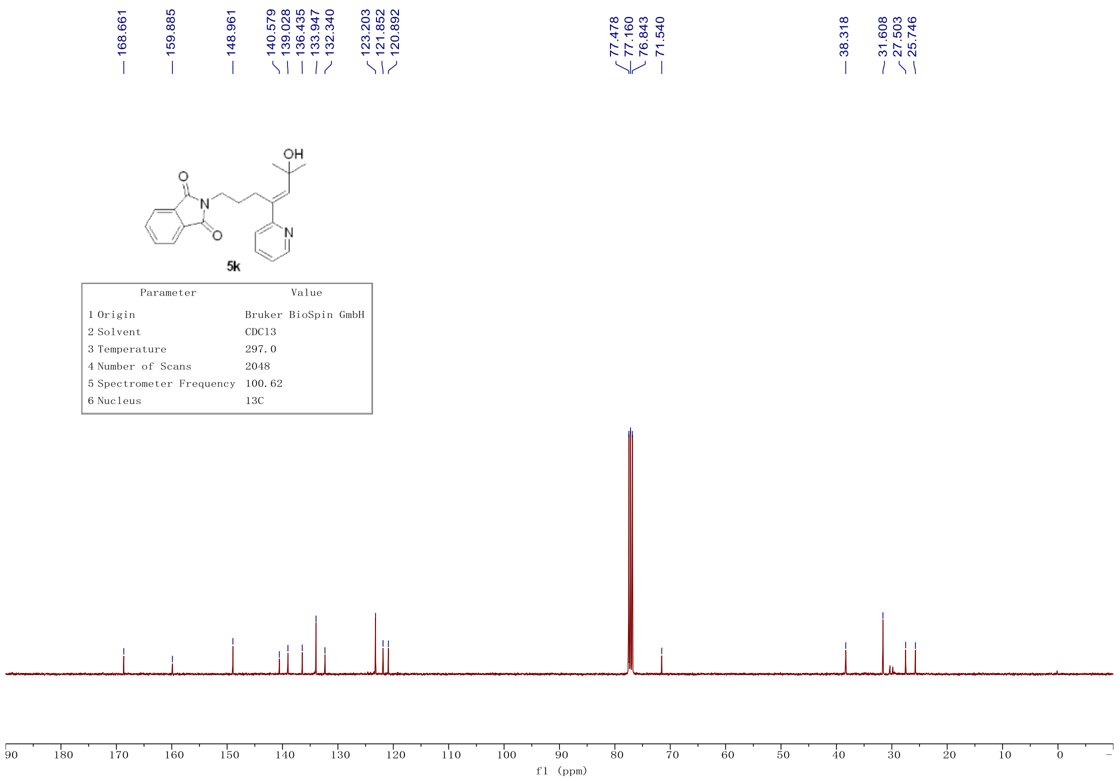

Figure S209. ^{13}C-NMR of **5k.**

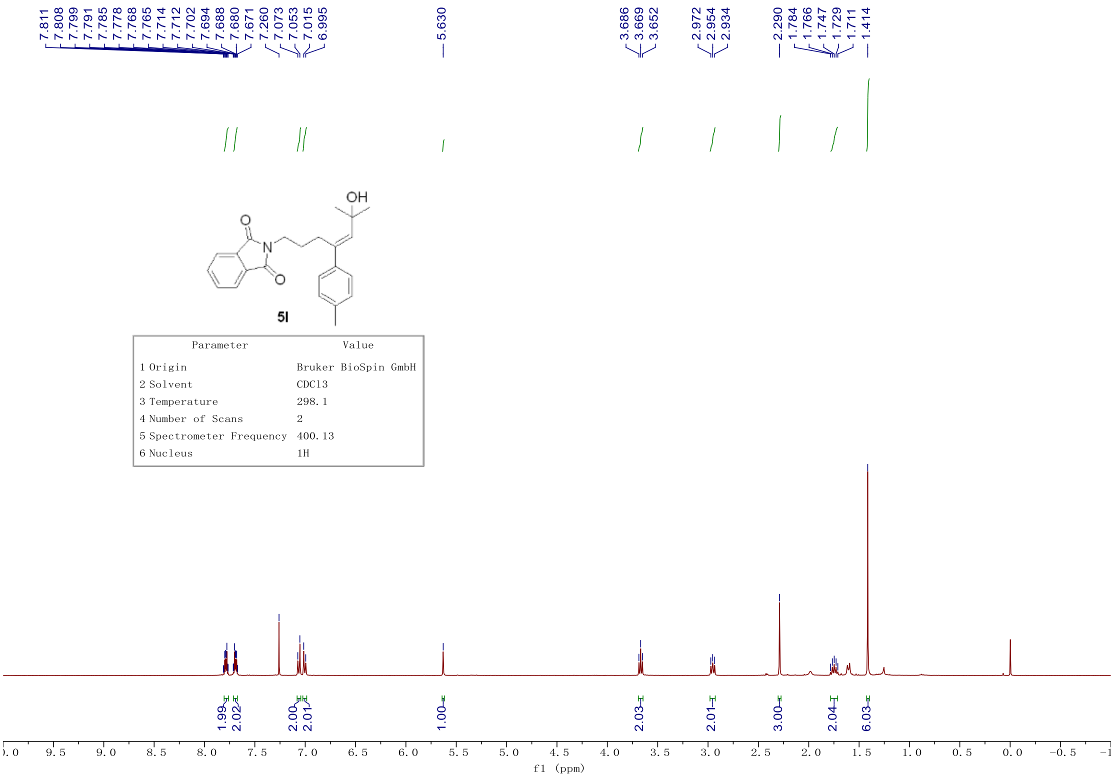

Figure S210. ^{1}H-NMR of **5l.**

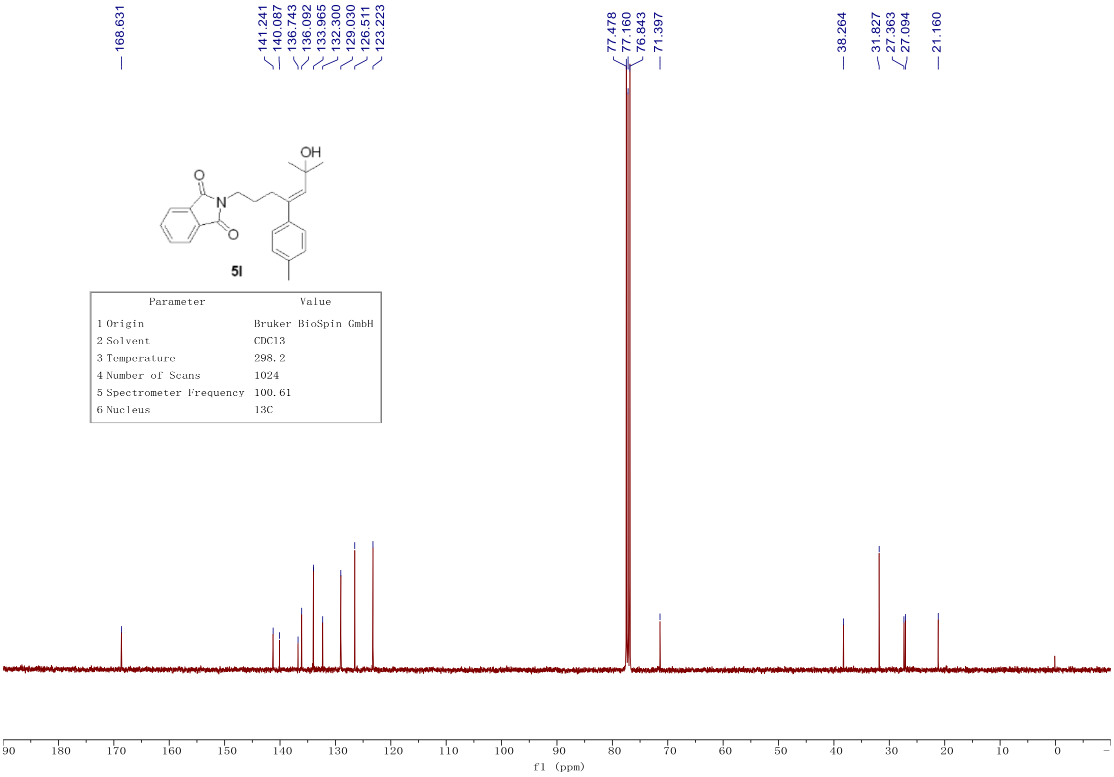

Figure S211. ^{13}C-NMR of **5l.**

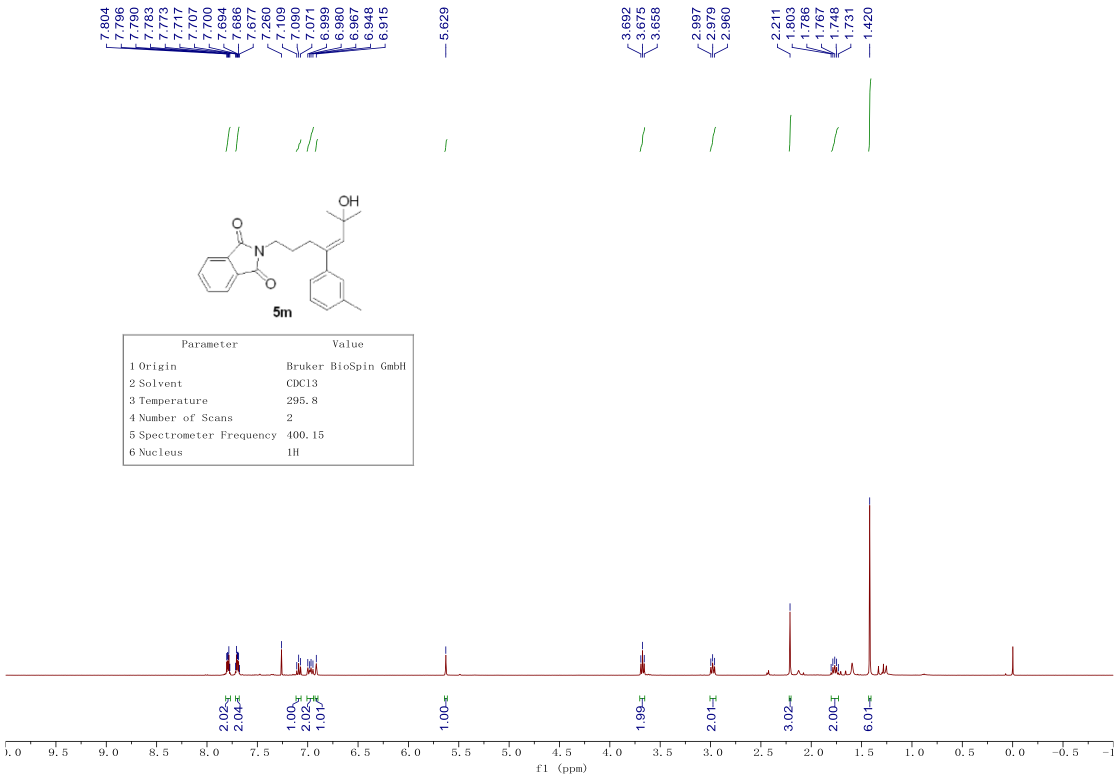

Figure S212. ^{1}H-NMR of **5m.**

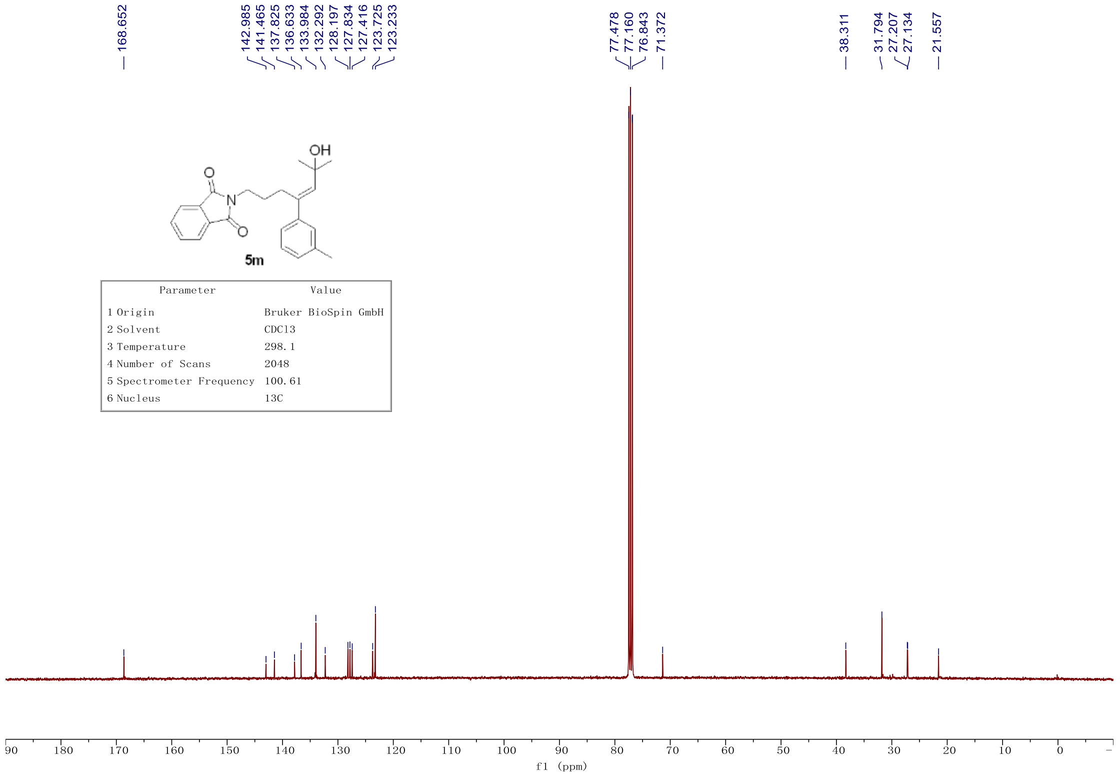

Figure S213. ^{13}C-NMR of **5m.**

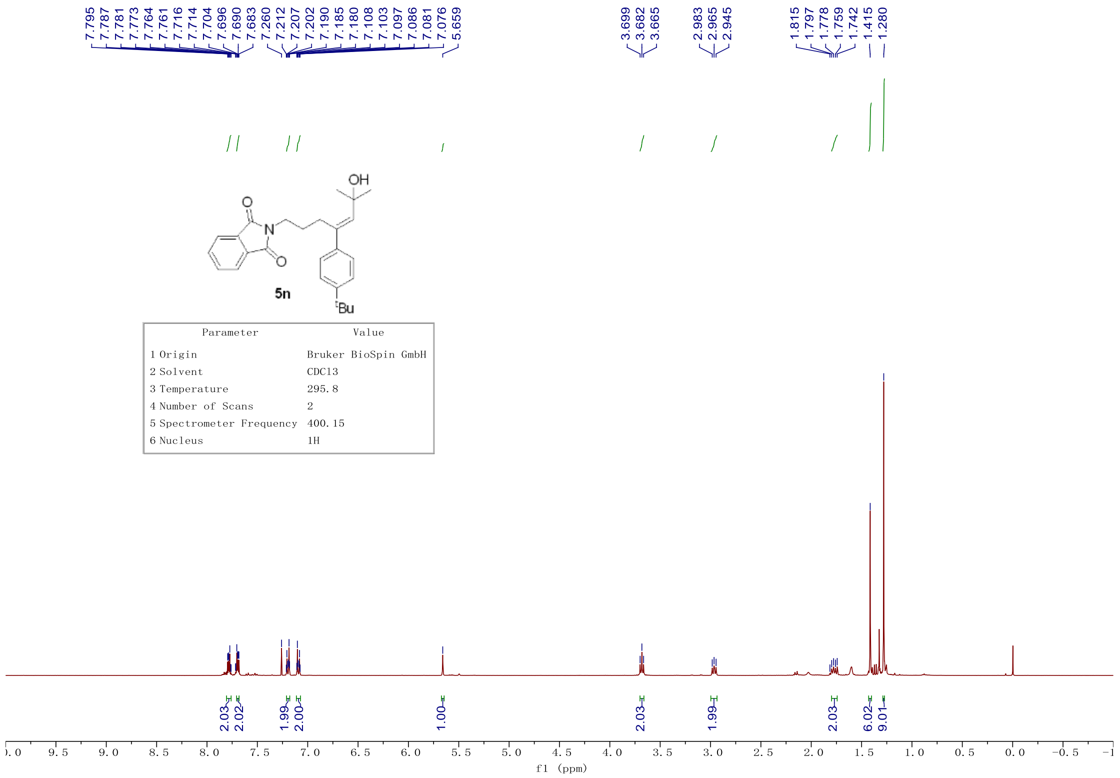

Figure S214. ^{1}H-NMR of **5n.**

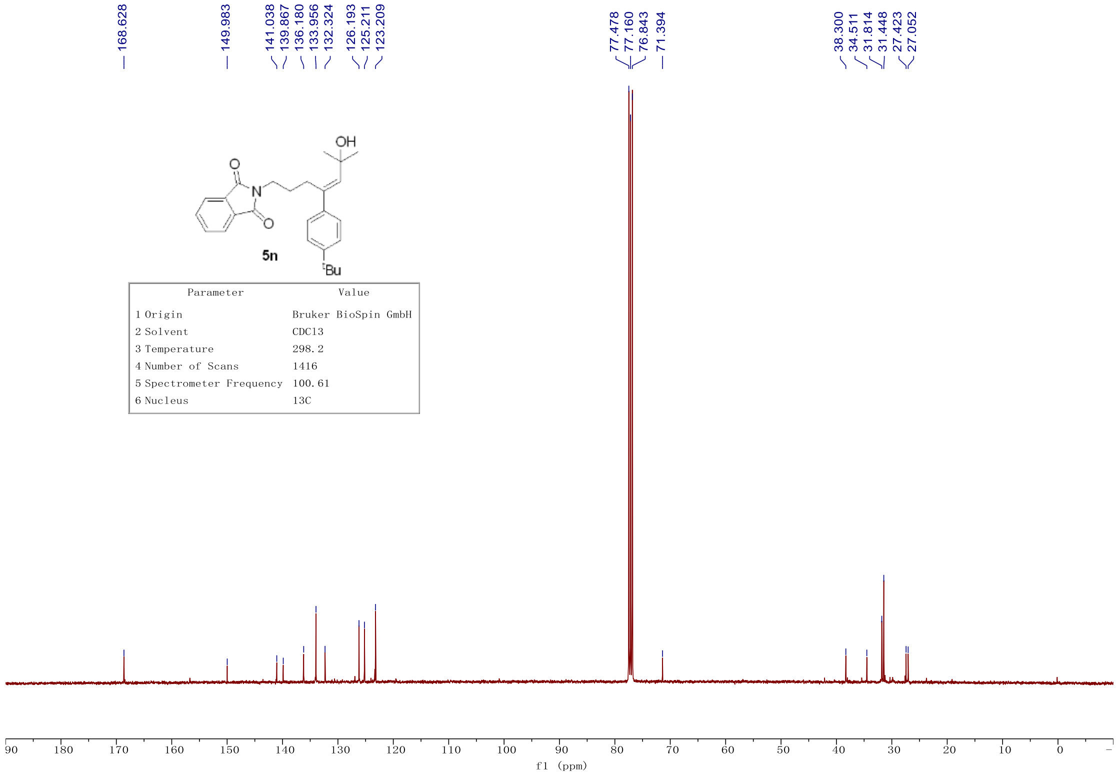

Figure S215. ^{13}C-NMR of **5n.**

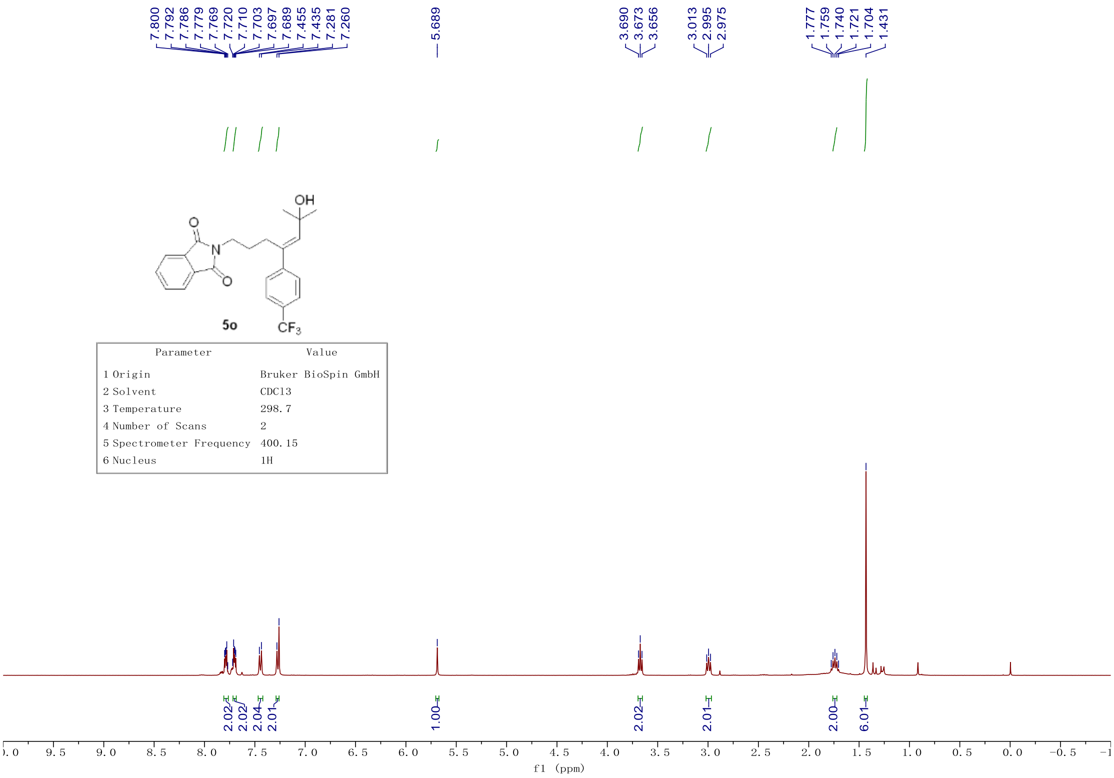

Figure S216. ^{1}H-NMR of **5o.**

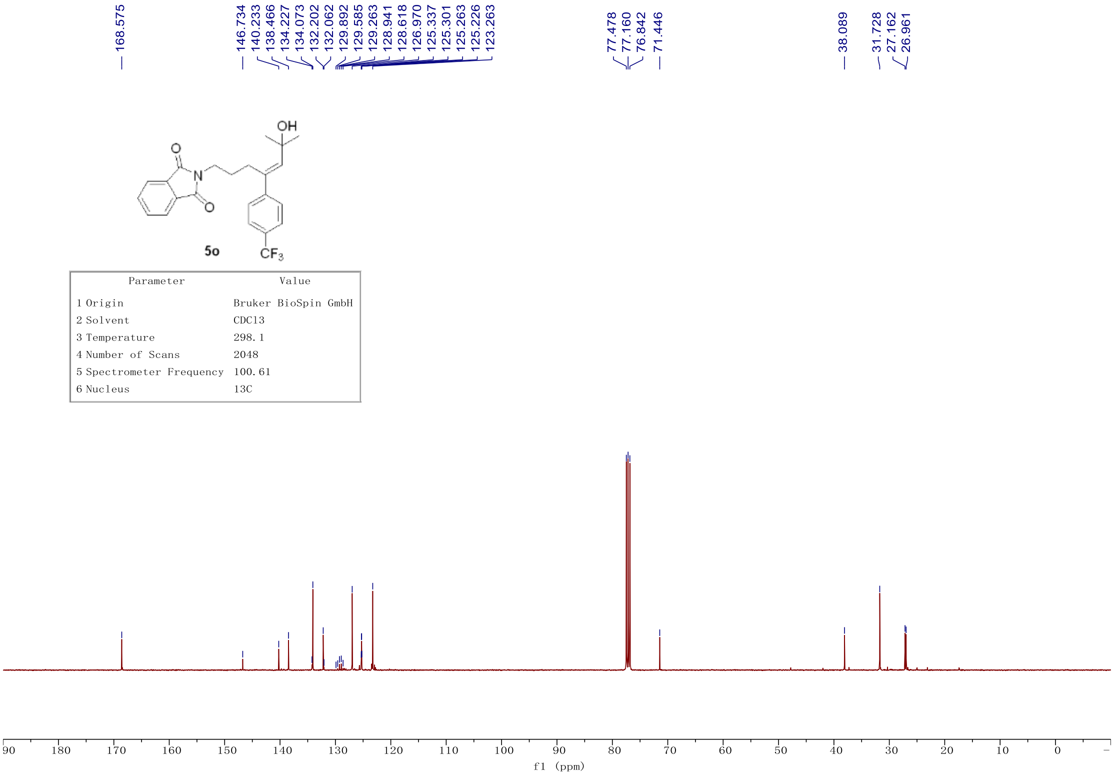

Figure S217. ^{13}C-NMR of **5o.**

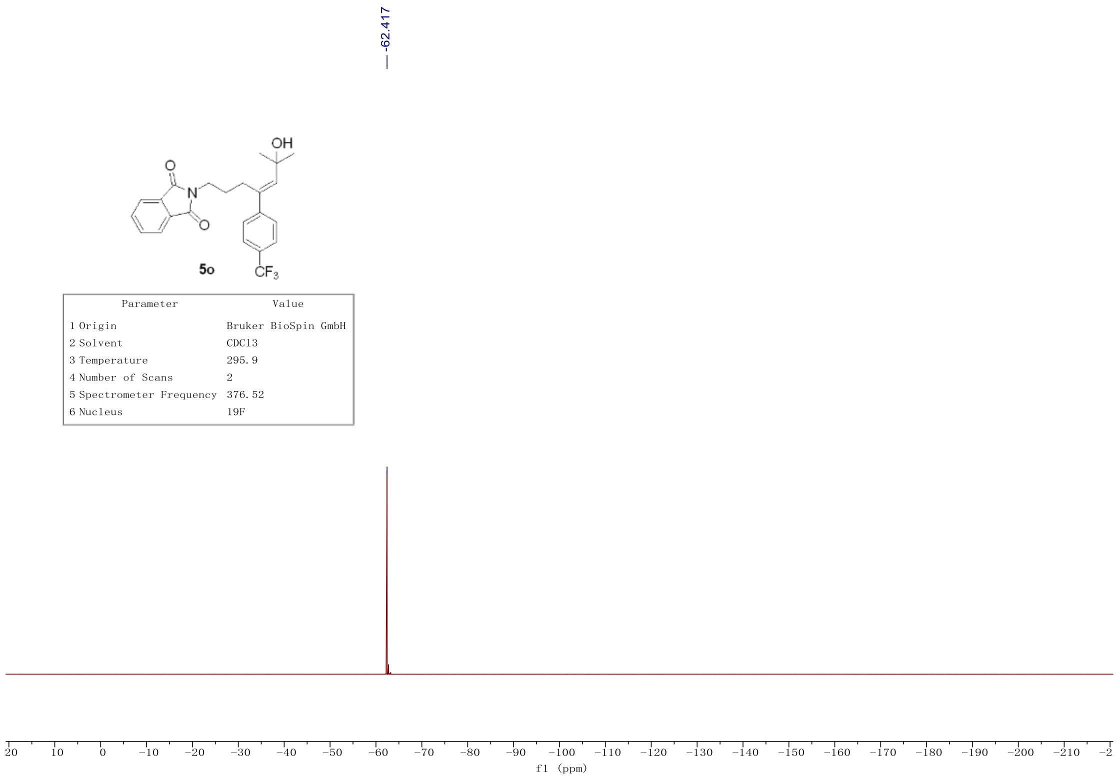

Figure S218. ^{18}F-NMR of **5o.**

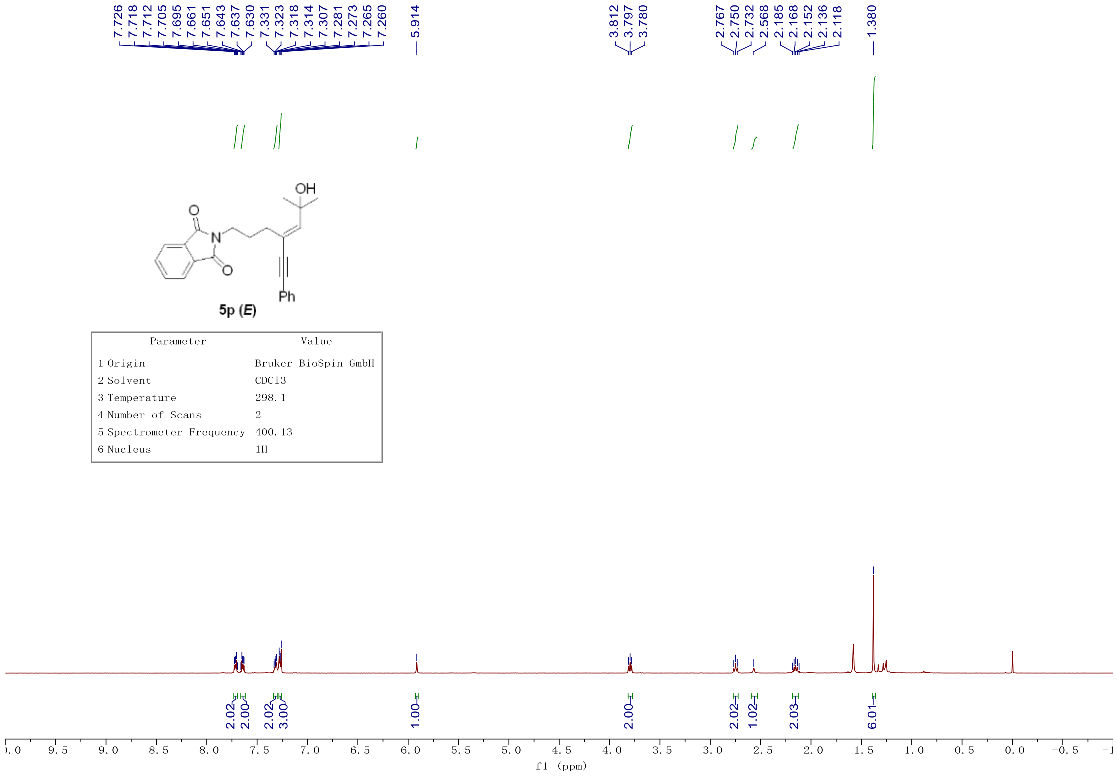

Figure S219. ^{1}H-NMR of **5p (*E*).**

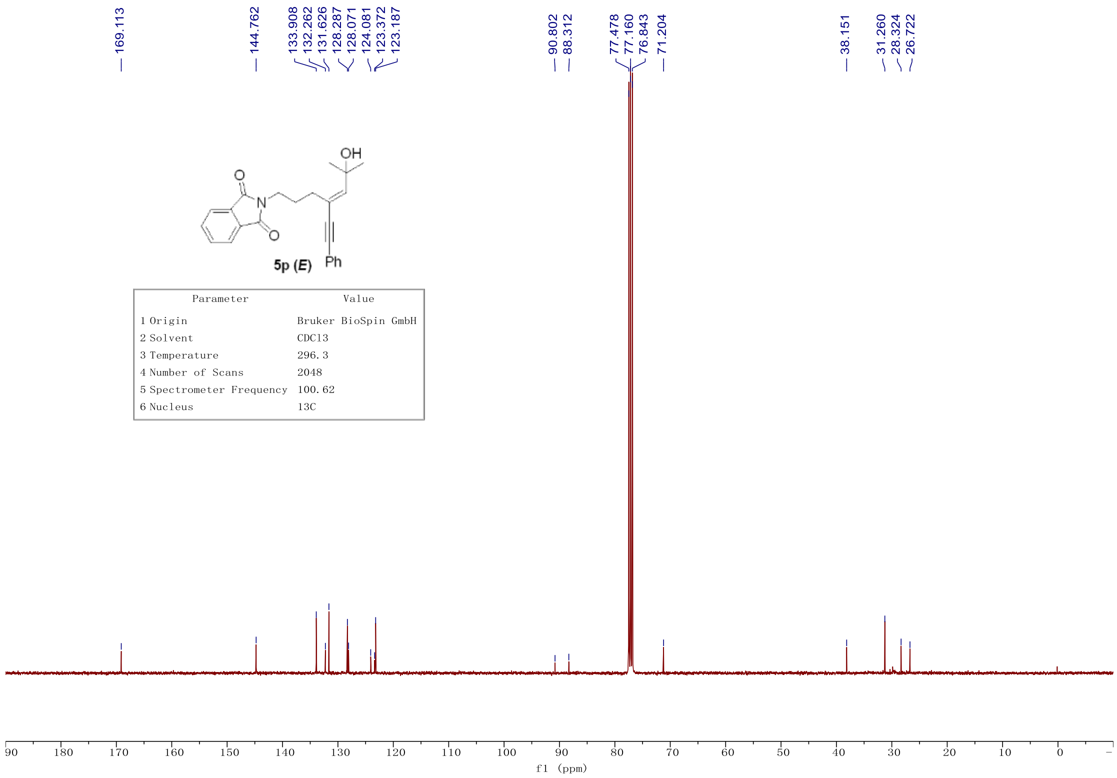

Figure S220. ^{13}C-NMR of **5p** (***E***).

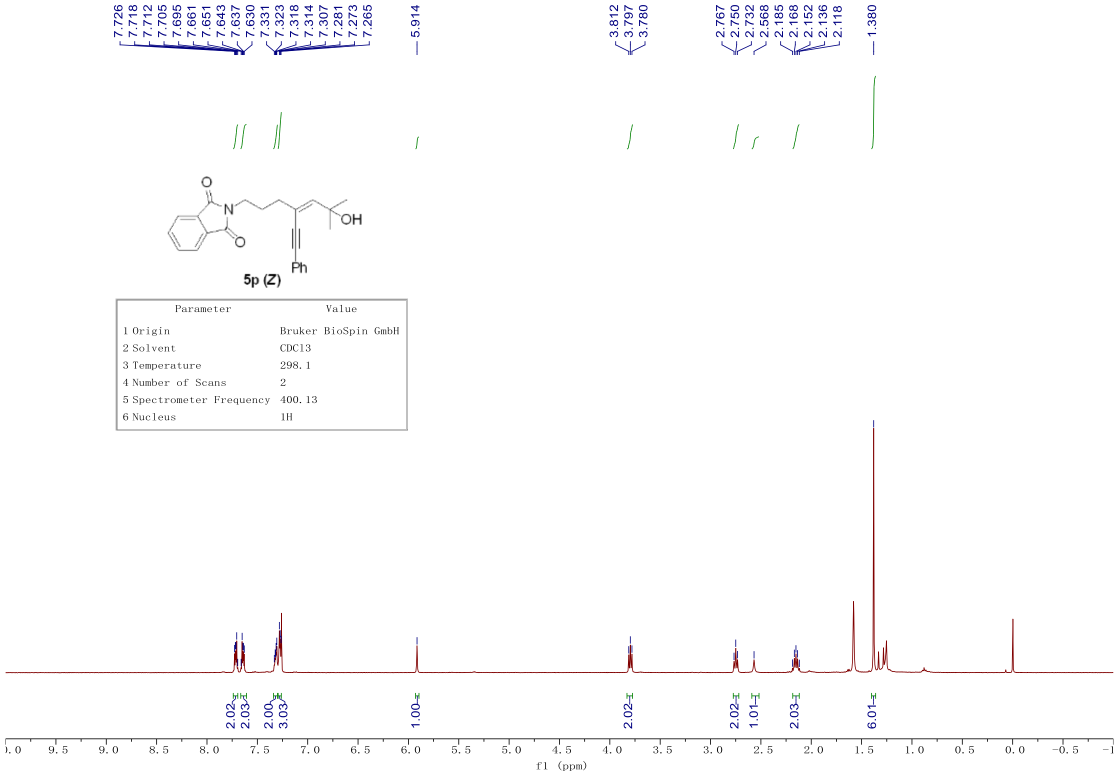

Figure S221. ^{1}H-NMR of **5p** (***Z***).

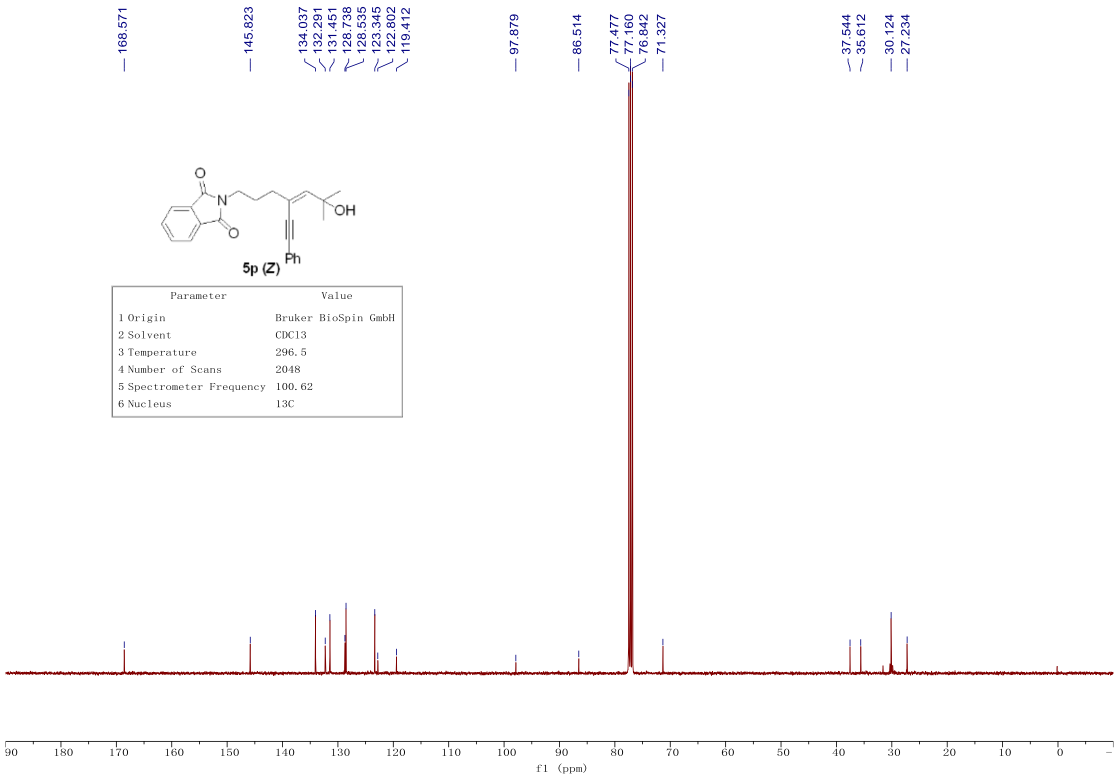

Figure S222. ^{13}C-NMR of **5p (*Z*).**

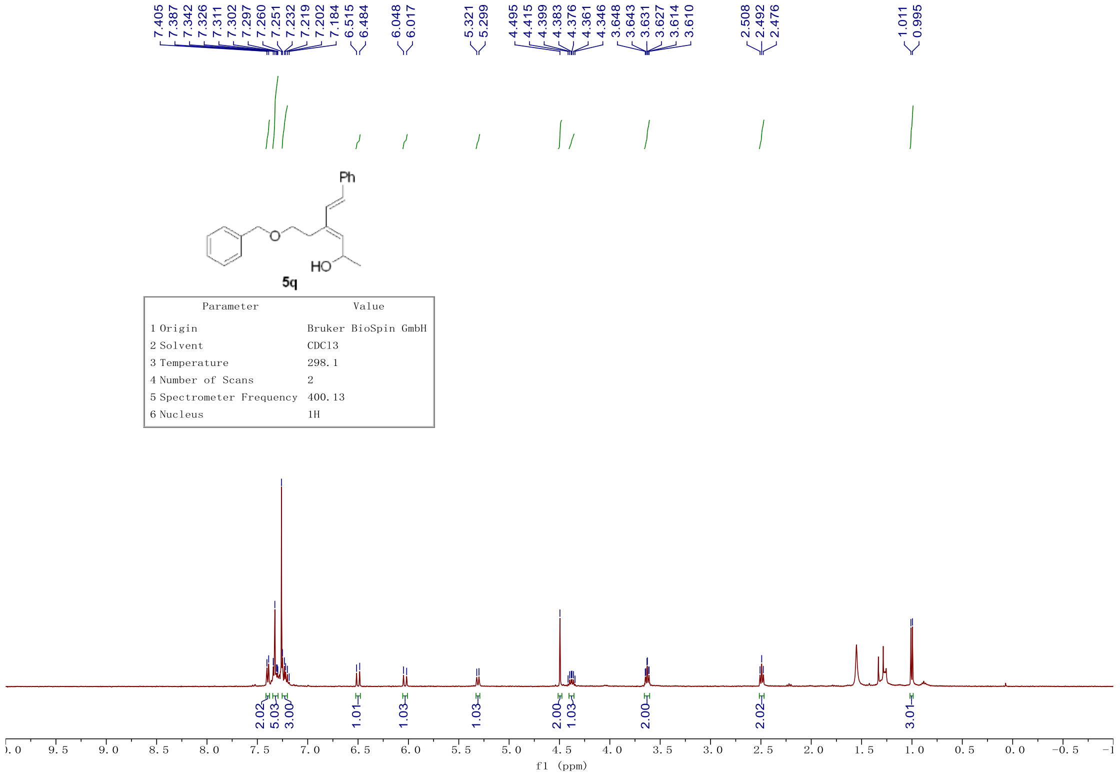

Figure S223. ^{1}H-NMR of **5q.**

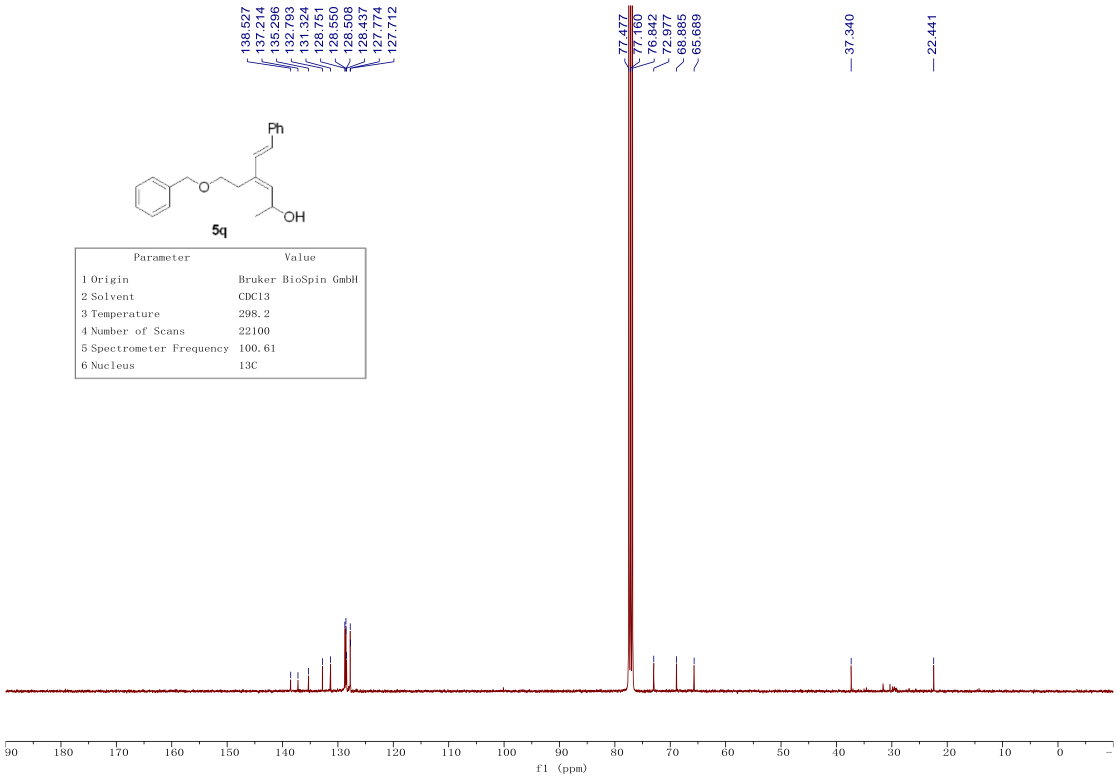

Figure S224. ^{13}C-NMR of **5q.**

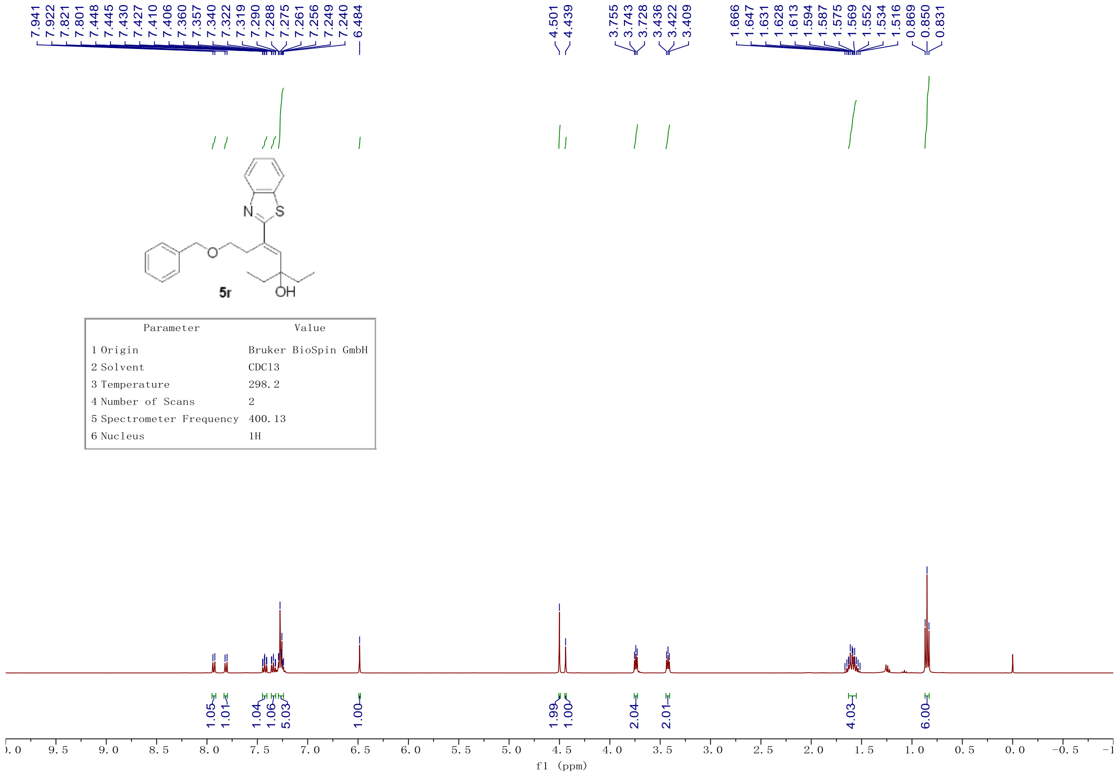

Figure S225. ^{1}H-NMR of **5r.**

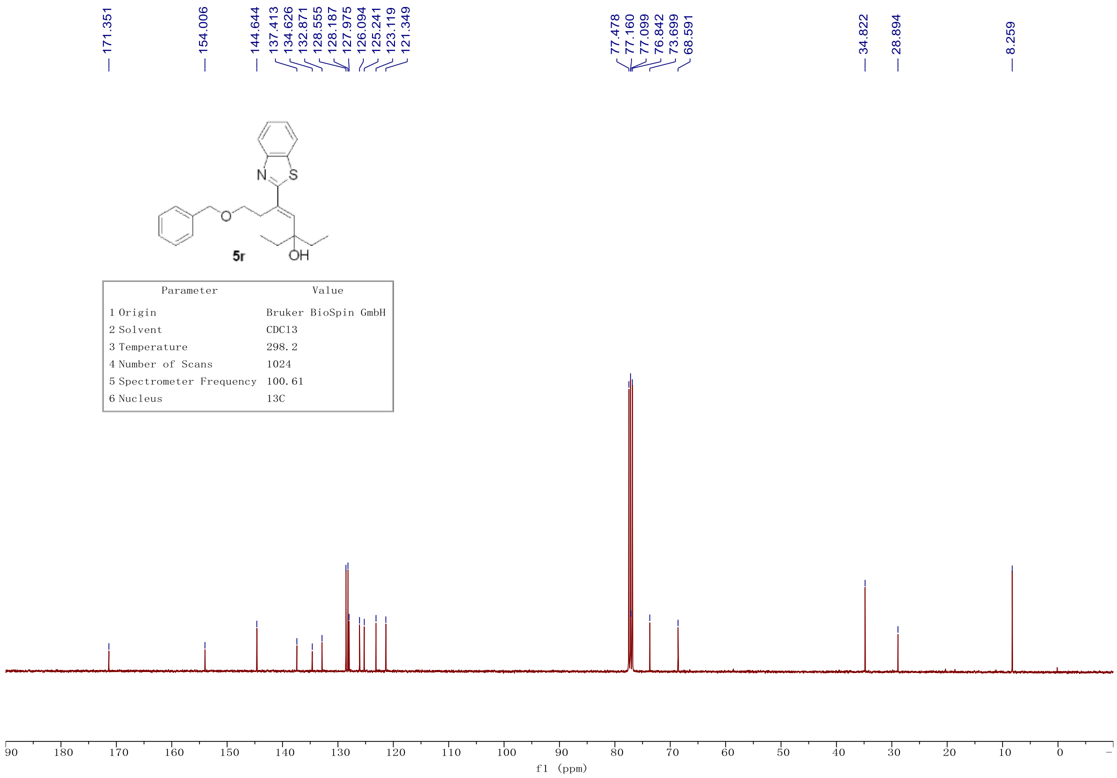

Figure S226. ^{13}C-NMR of **5r.**

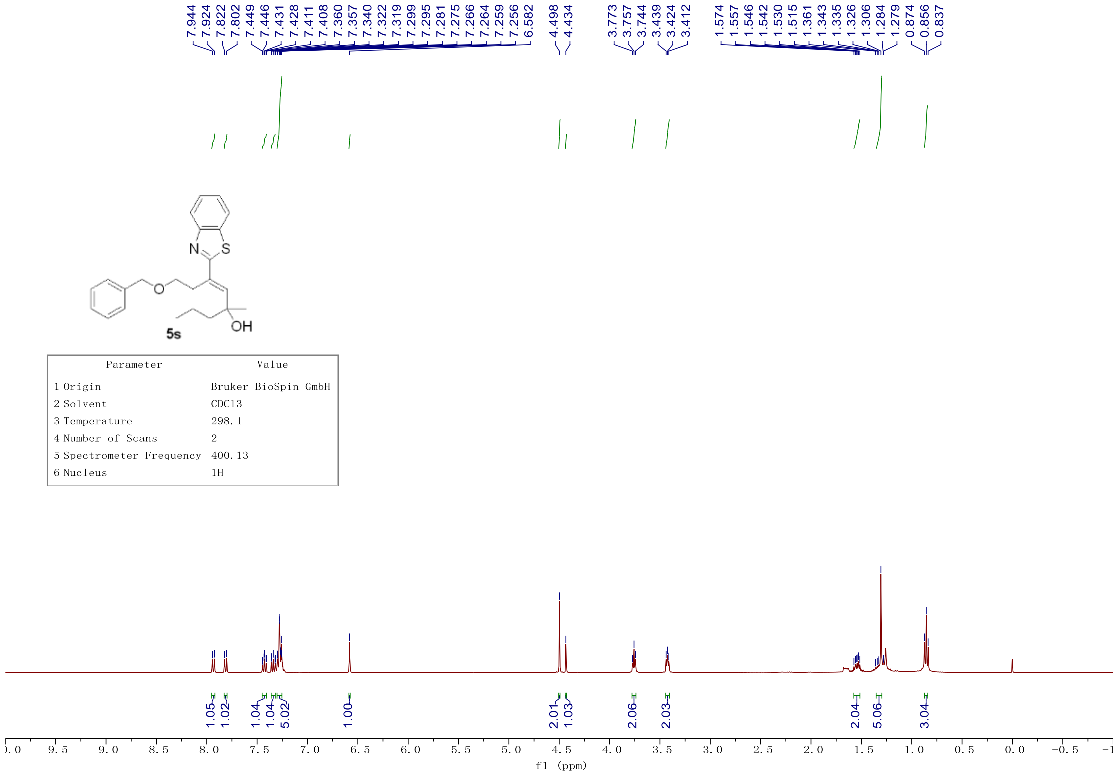

Figure S227. ^{1}H-NMR of **5s.**

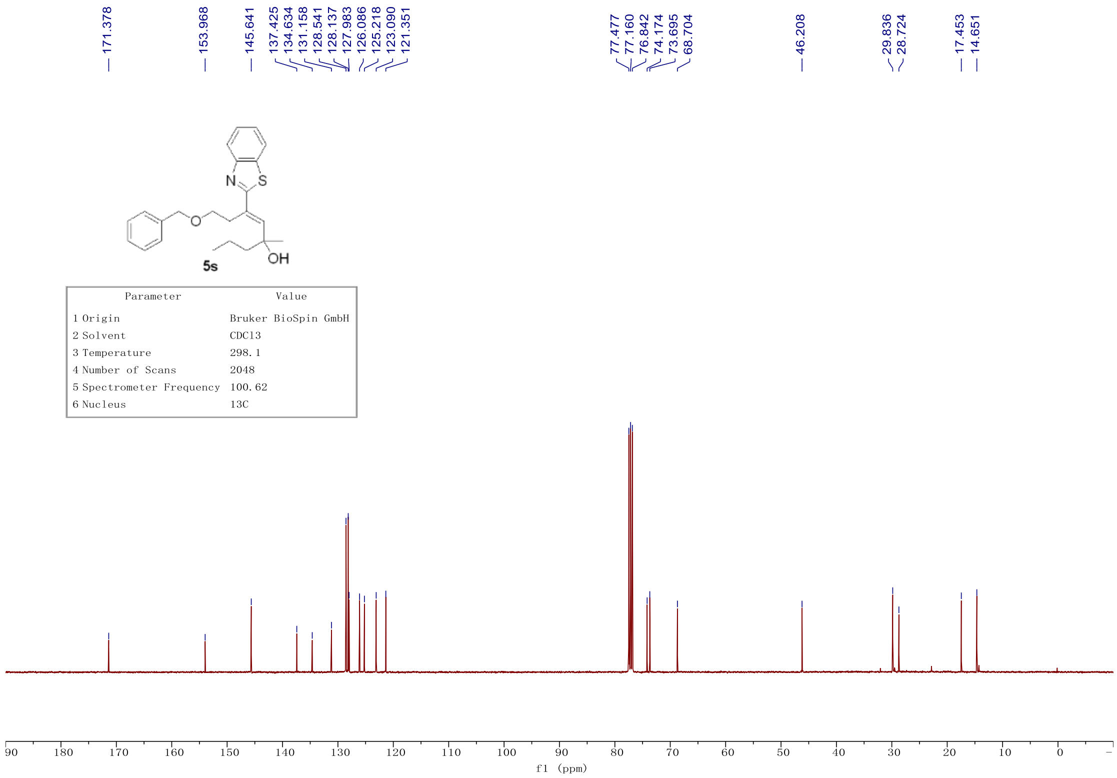

Figure S228. ^{13}C-NMR of **5s.**

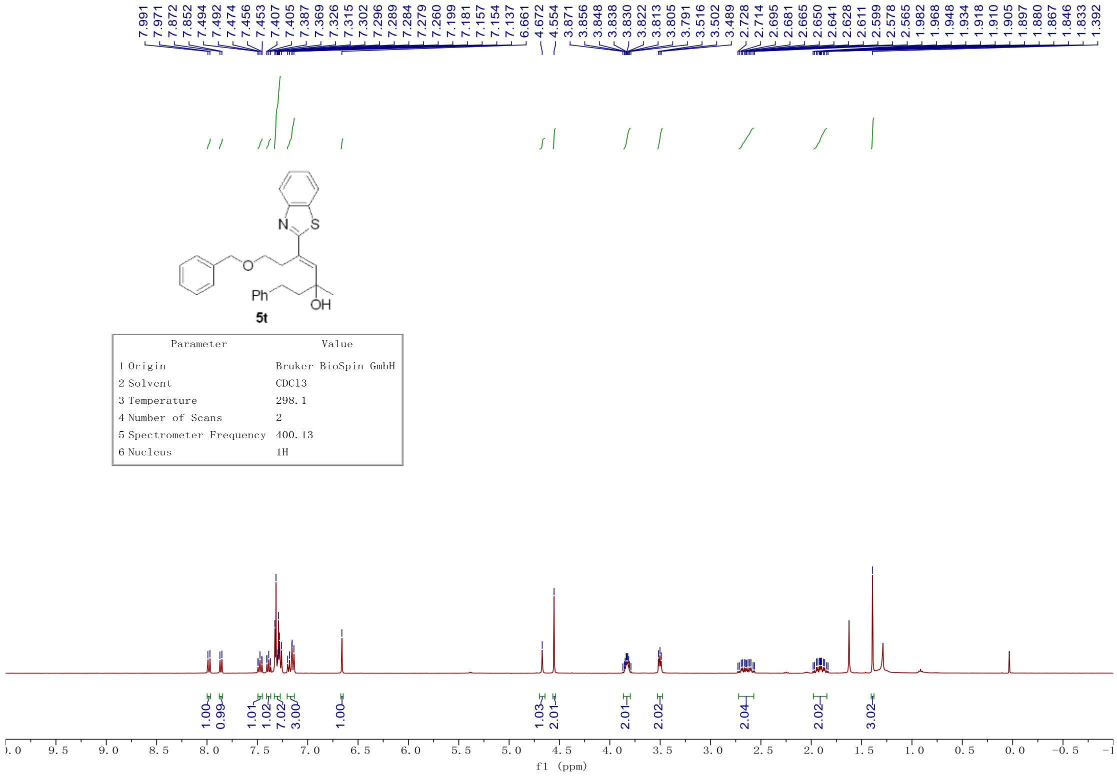

Figure S229. ^{1}H-NMR of **5t.**

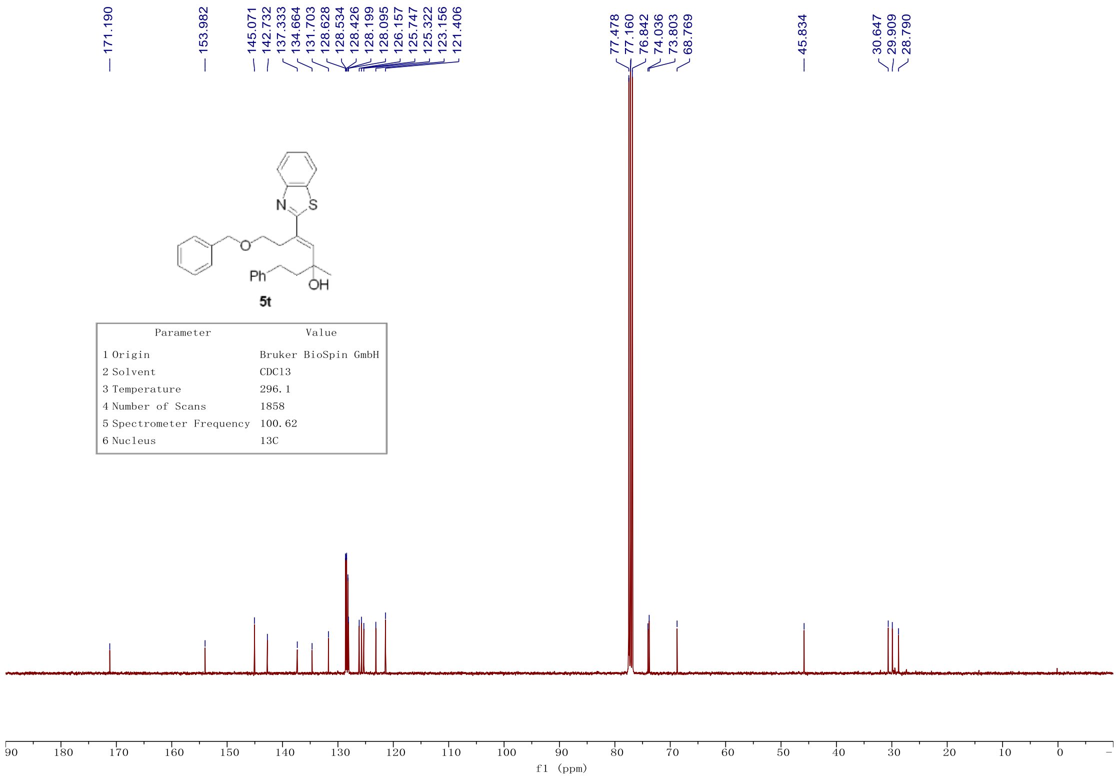

Figure S230. ^{13}C-NMR of **5t.**

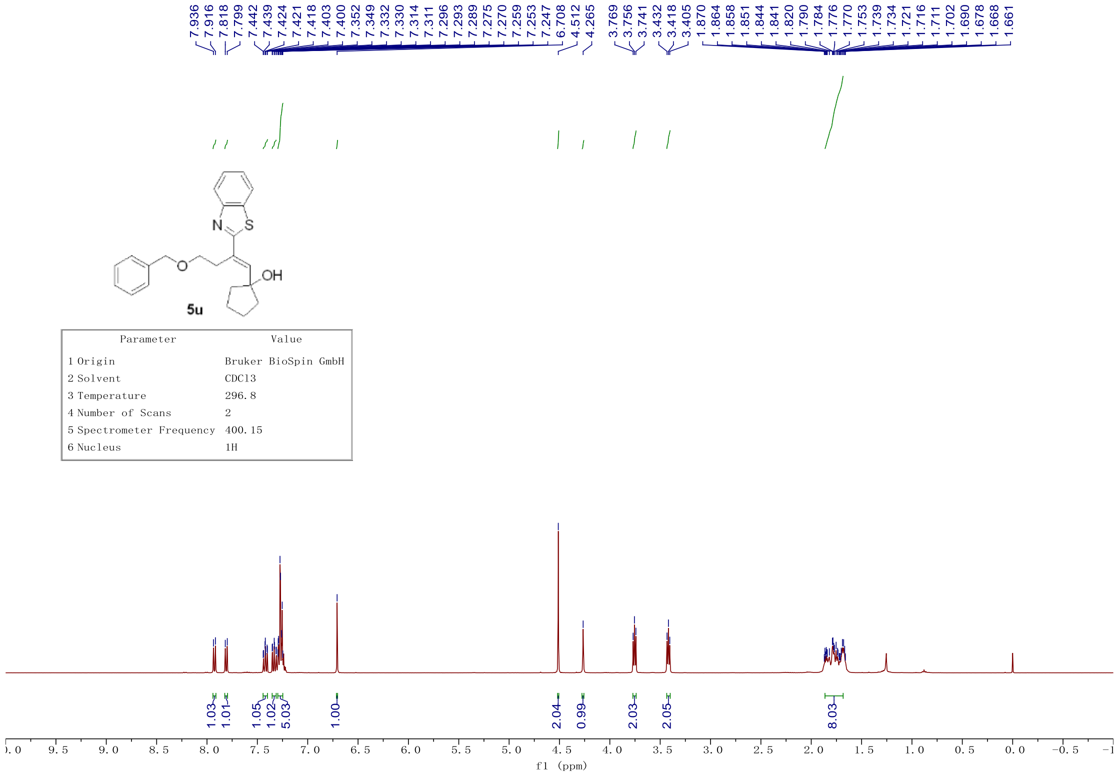

Figure S231. ^{1}H-NMR of **5u.**

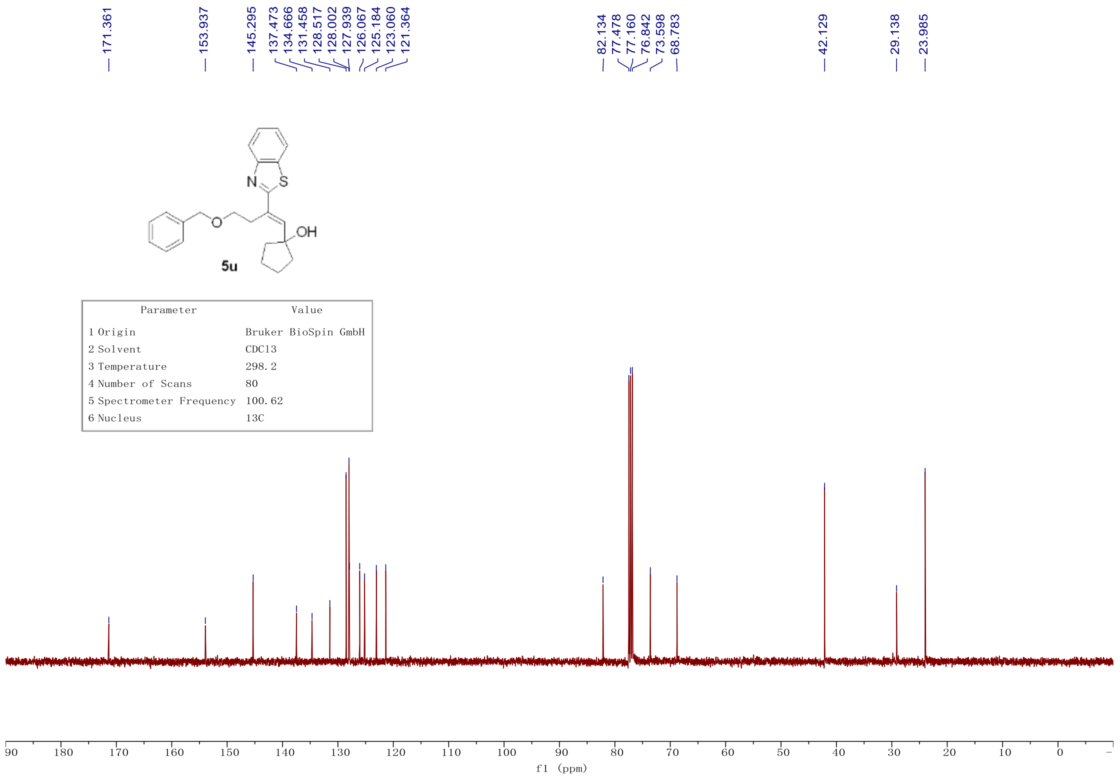

Figure S232. ^{13}C-NMR of **5u.**

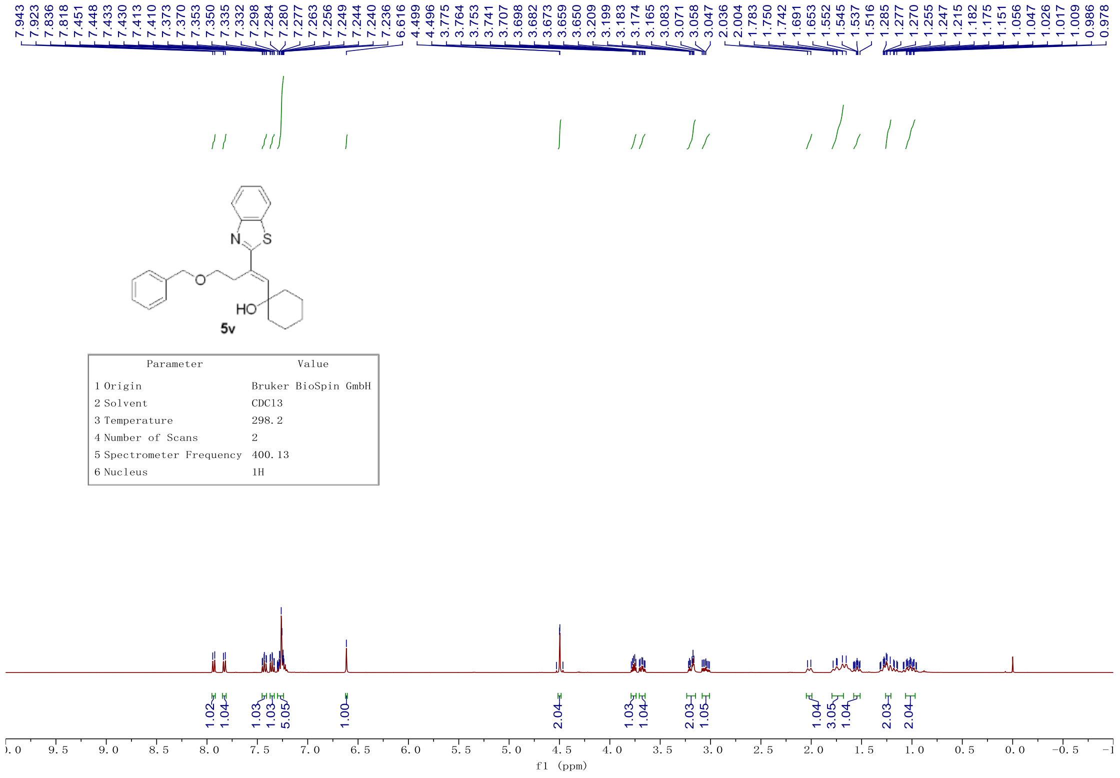

Figure S233. ^{1}H-NMR of **5v.**

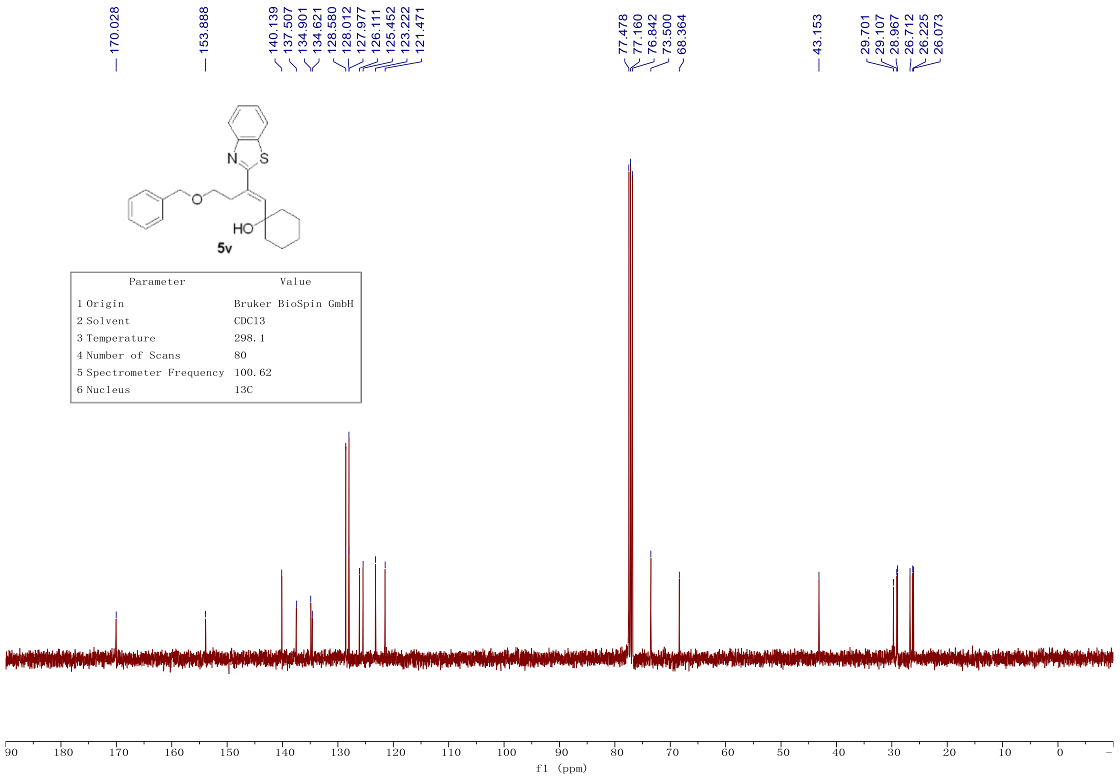

Figure S234. ^{13}C-NMR of **5v.**

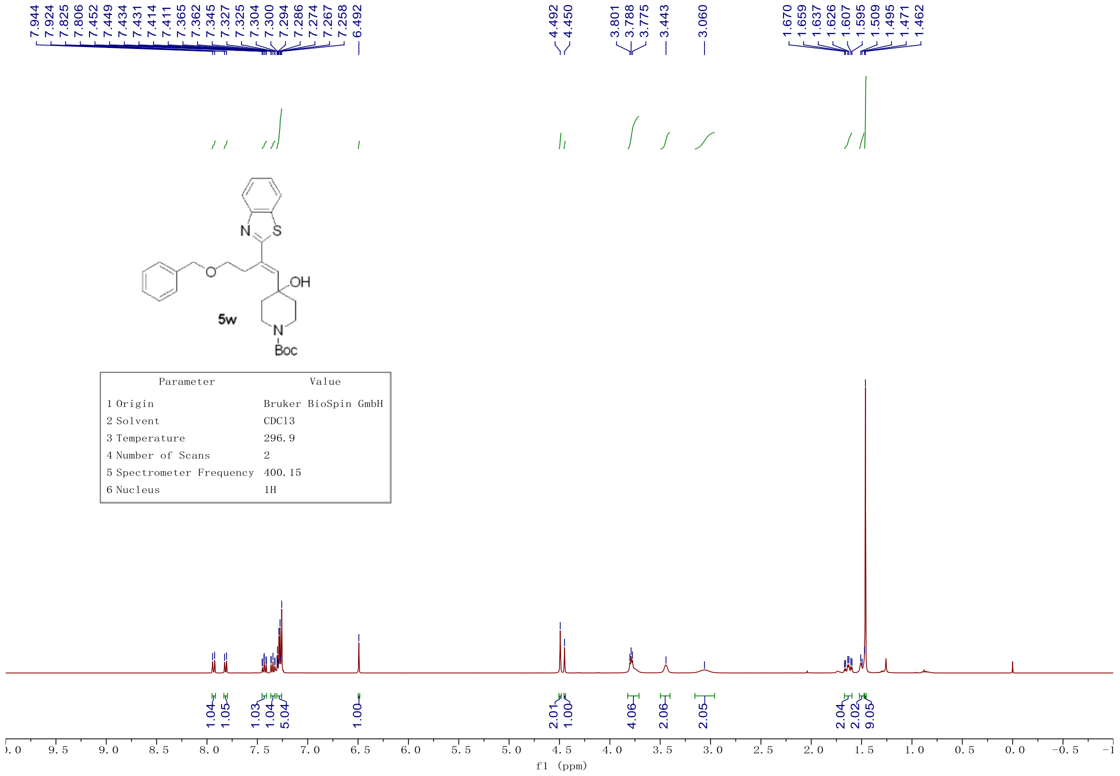

Figure S235. ^{1}H-NMR of **5w.**

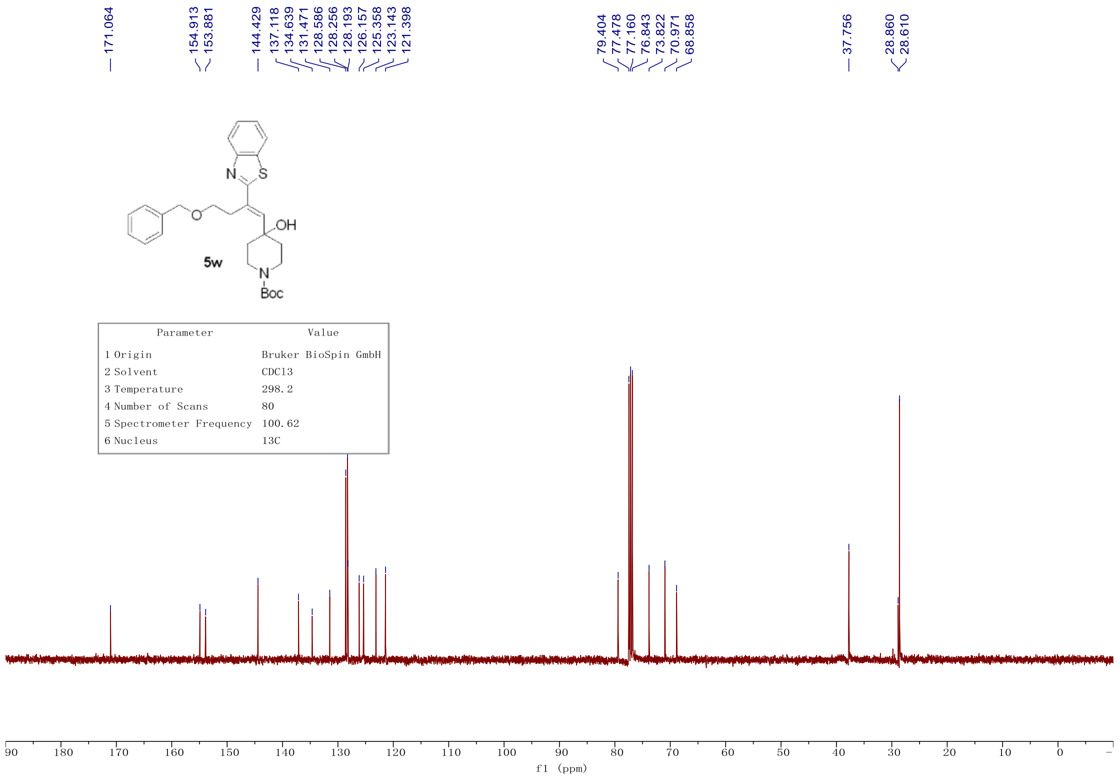

Figure S236. ^{13}C-NMR of **5w.**

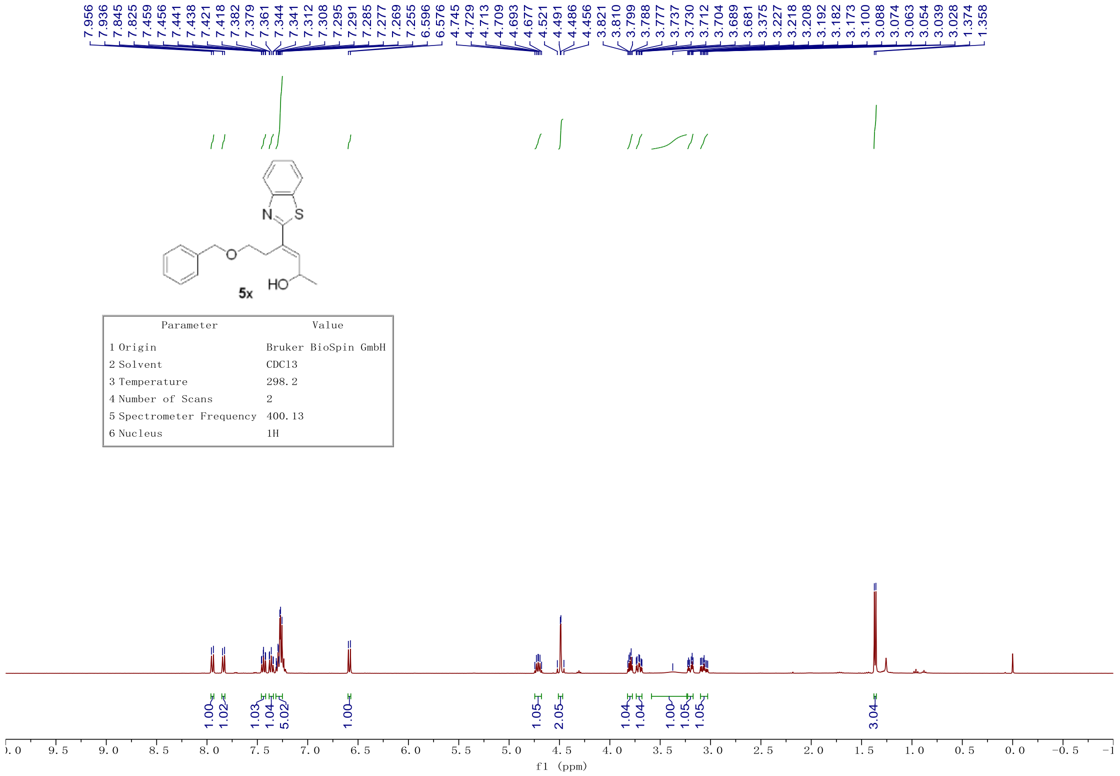

Figure S237. ^{1}H-NMR of **5x.**

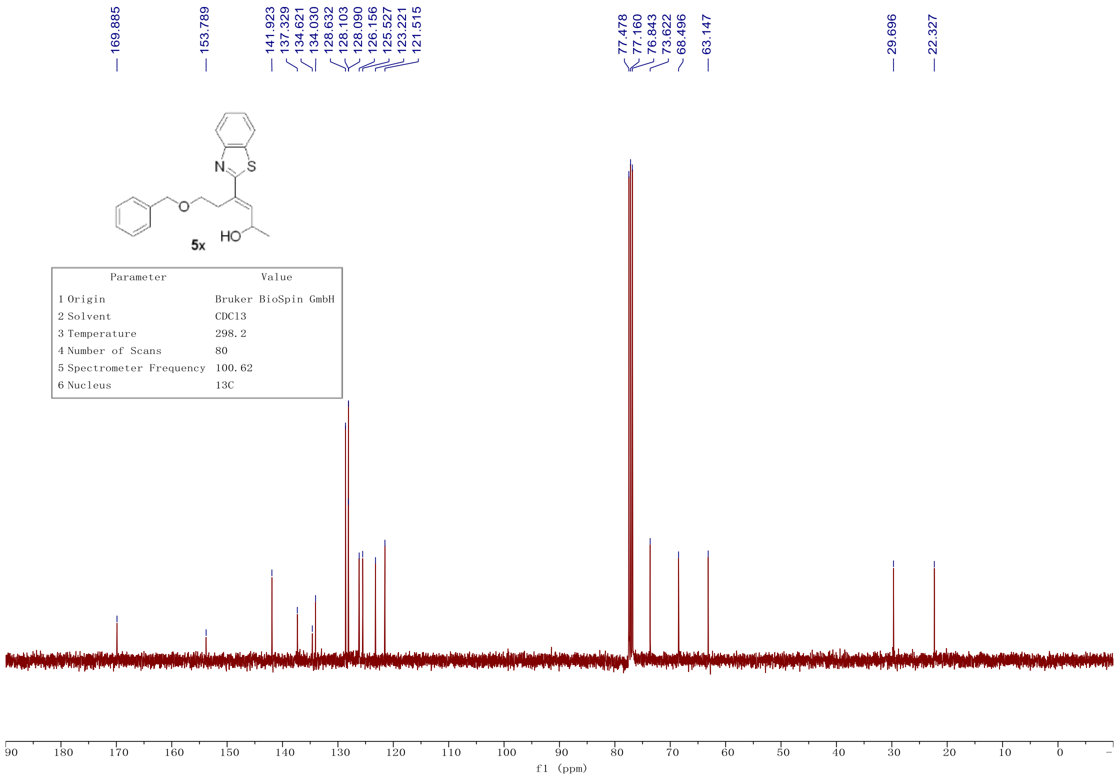

Figure S238. ^{13}C-NMR of **5x.**

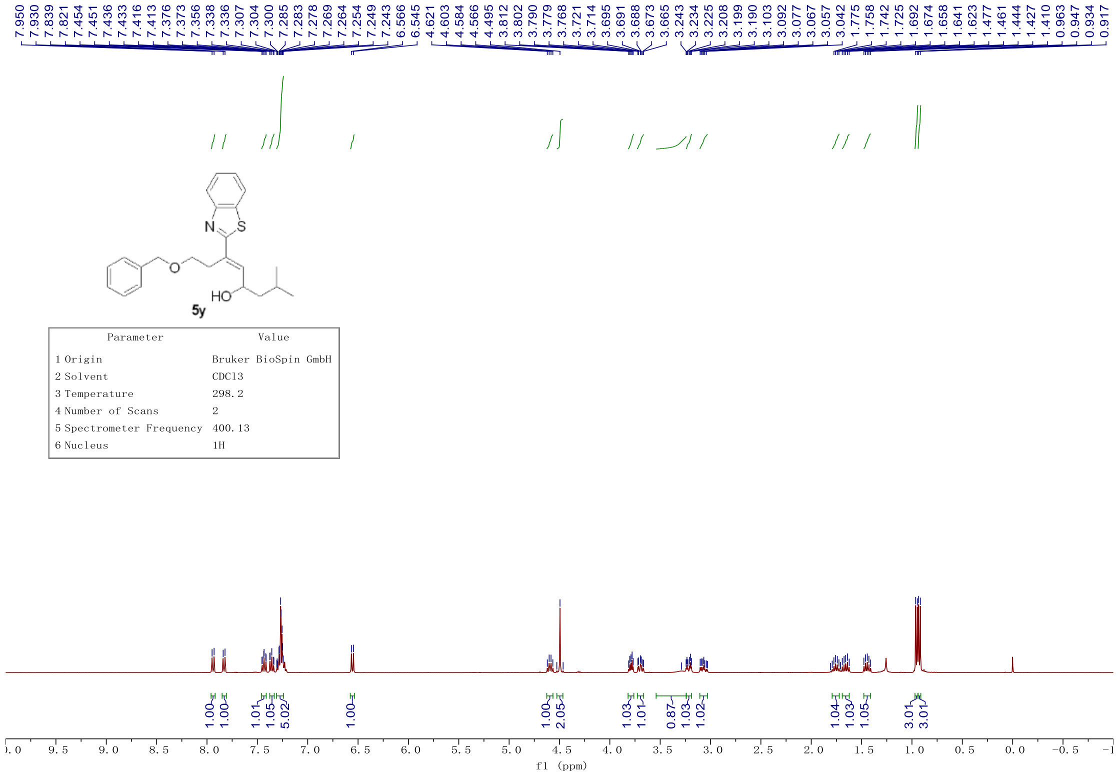

Figure S239. ^{1}H-NMR of **5y.**

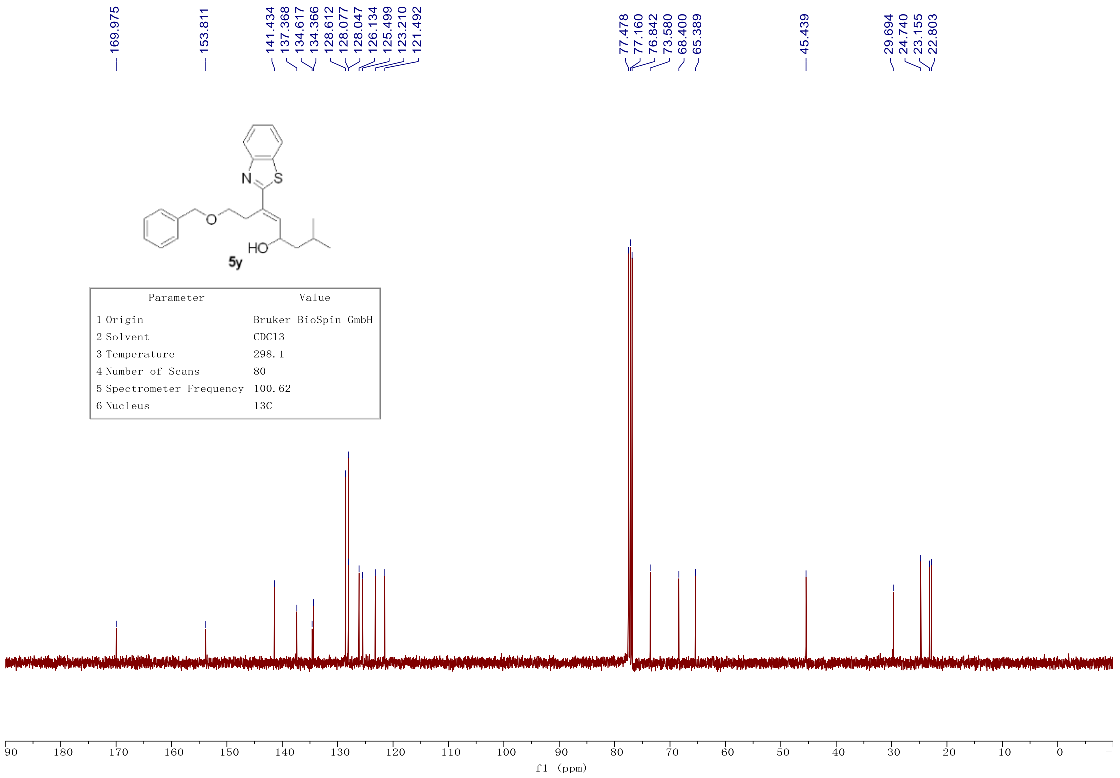

Figure S240. ^{13}C-NMR of **5y.**

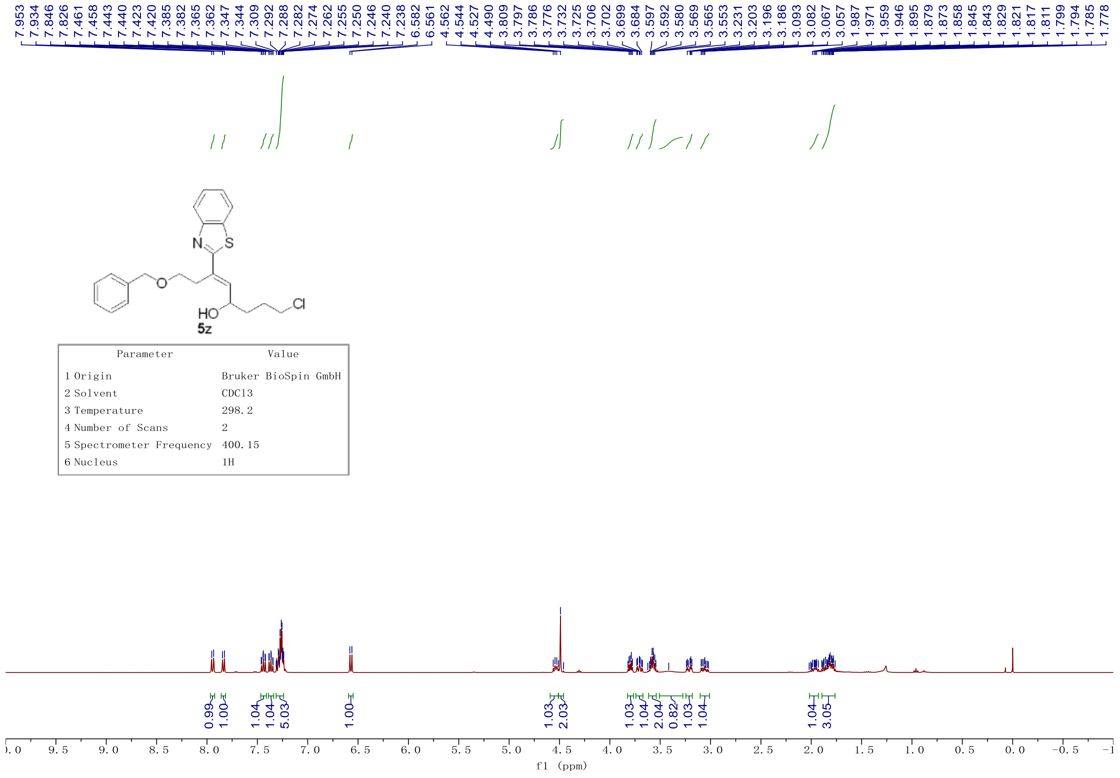

Figure S241. ^{1}H-NMR of **5z.**

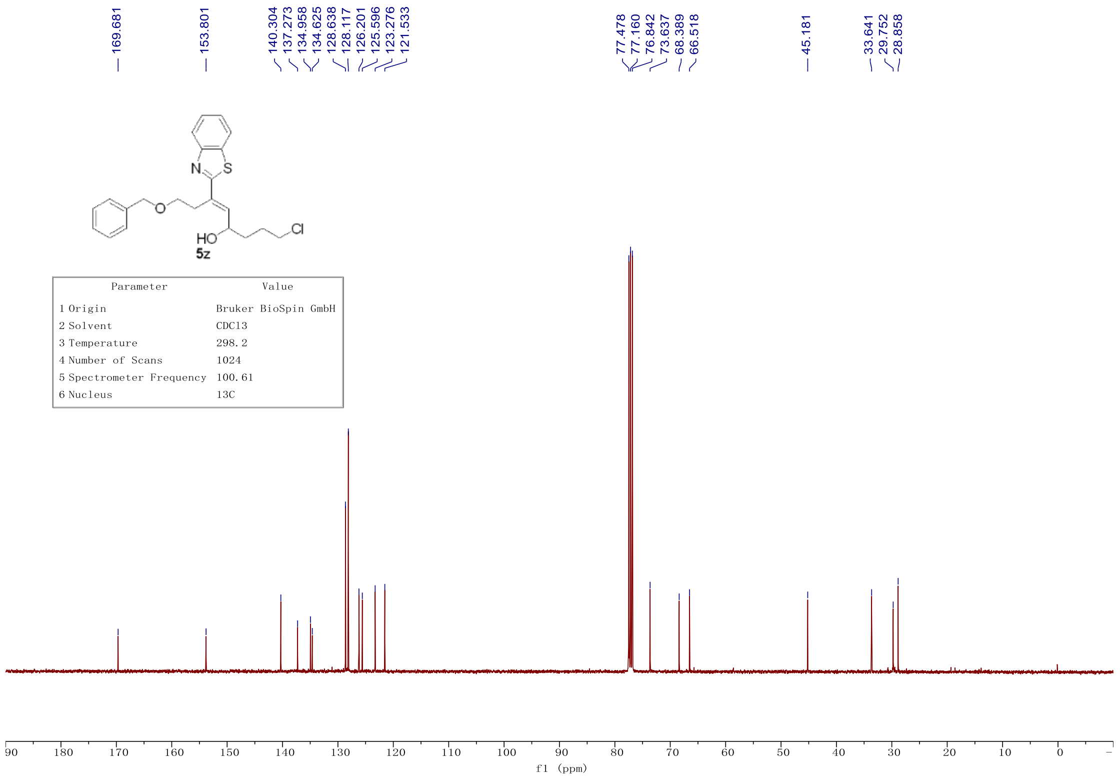

Figure S242. ^{13}C-NMR of **5z.**

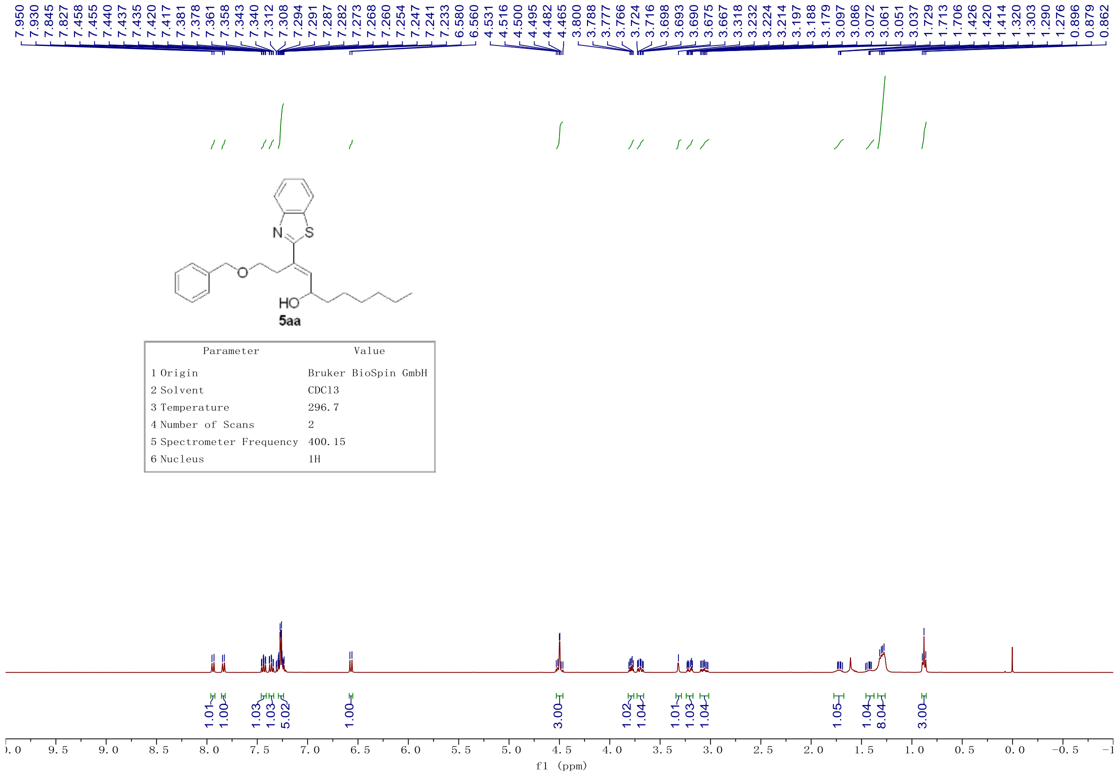

Figure S243. ^{1}H-NMR of **5aa.**

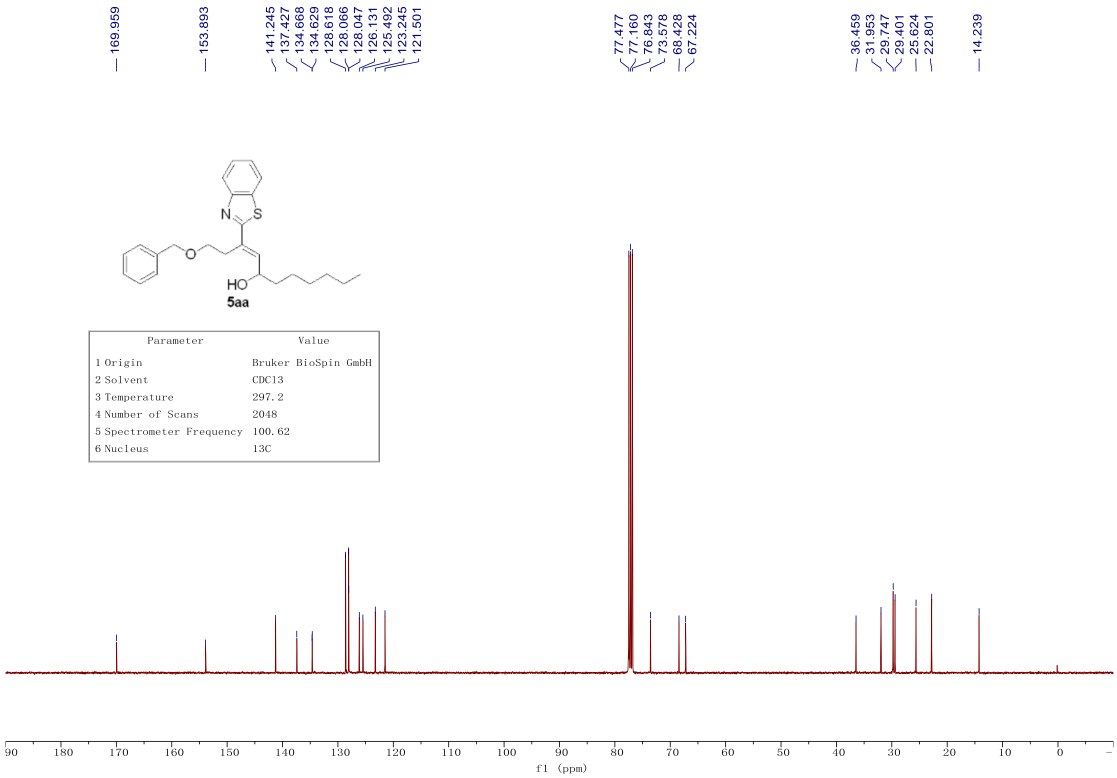

Figure S244. ^{13}C-NMR of **5aa.**

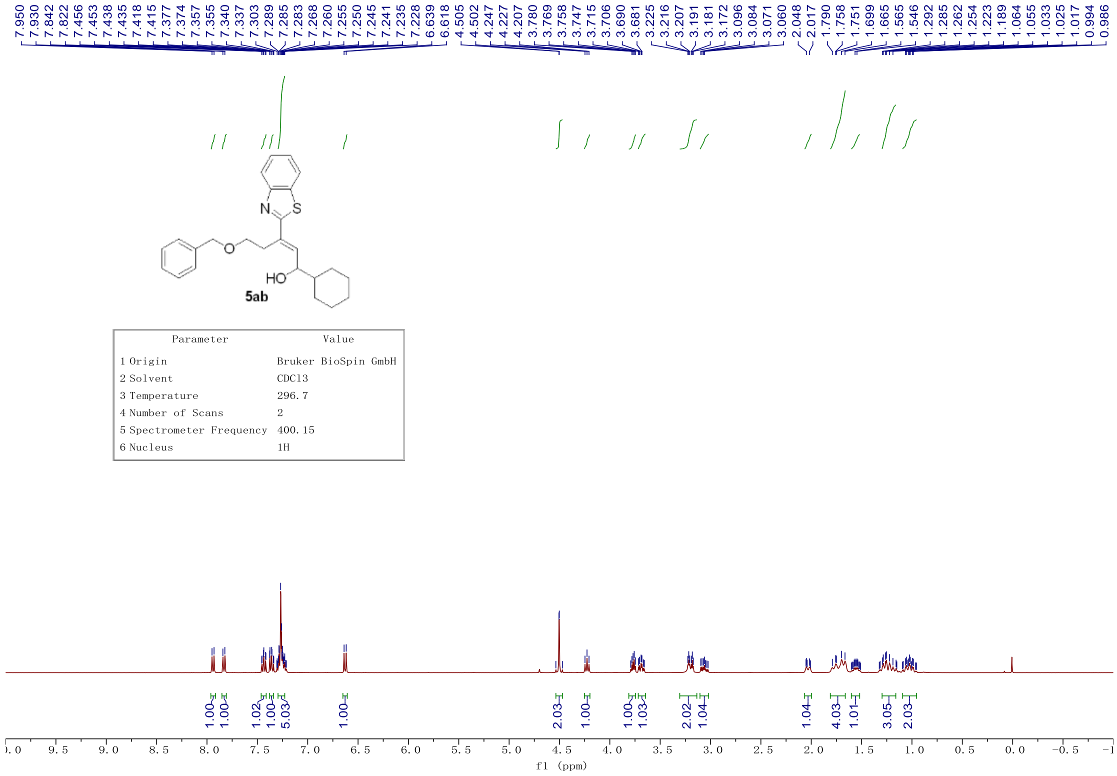

Figure S245. ^{1}H-NMR of **5ab.**

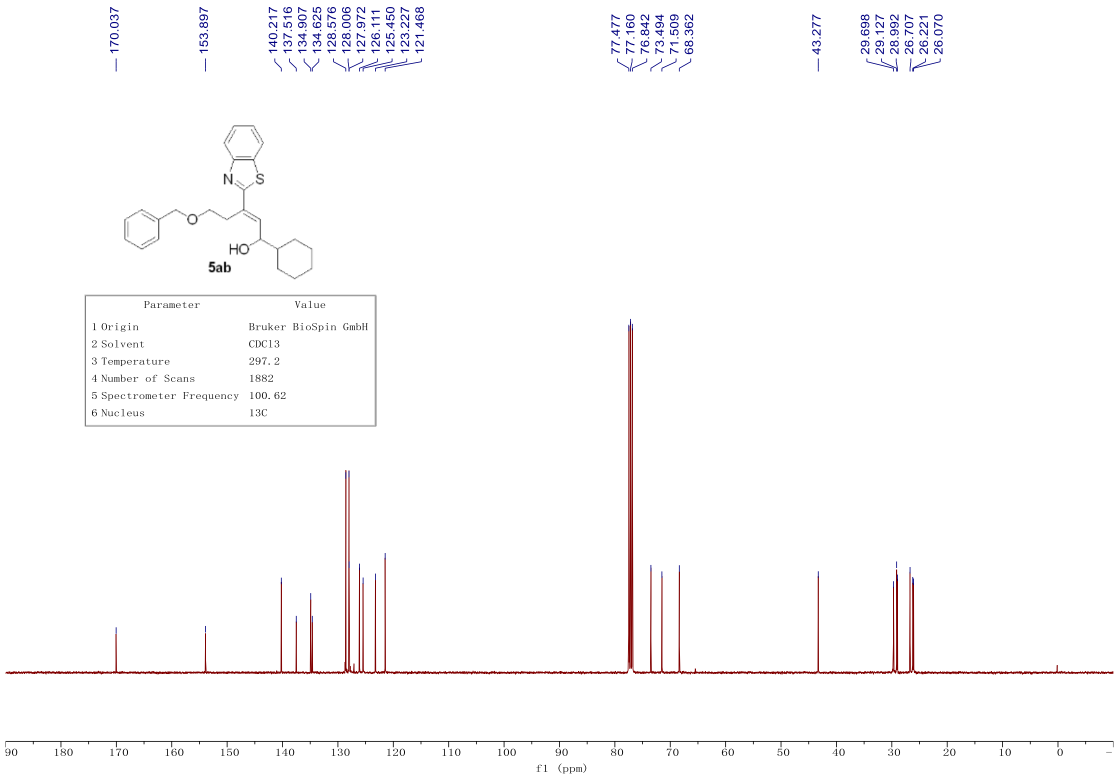

Figure S246. ^{13}C-NMR of **5ab.**

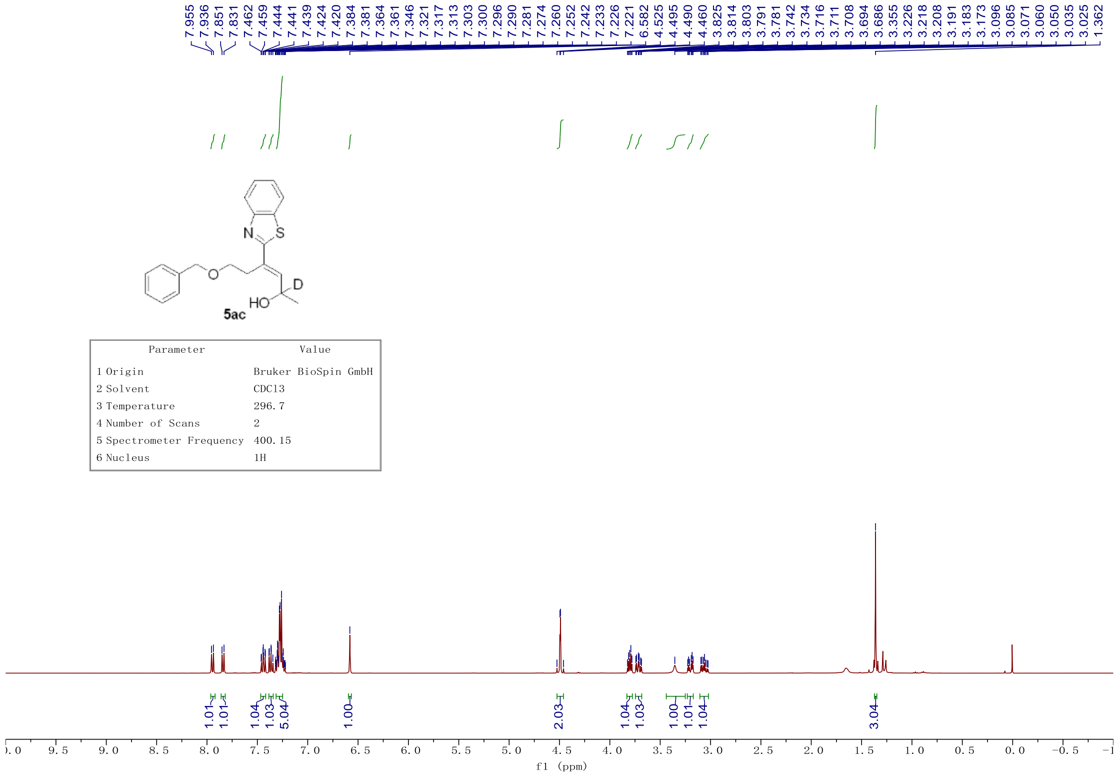

Figure S247. ^{1}H-NMR of **5ac.**

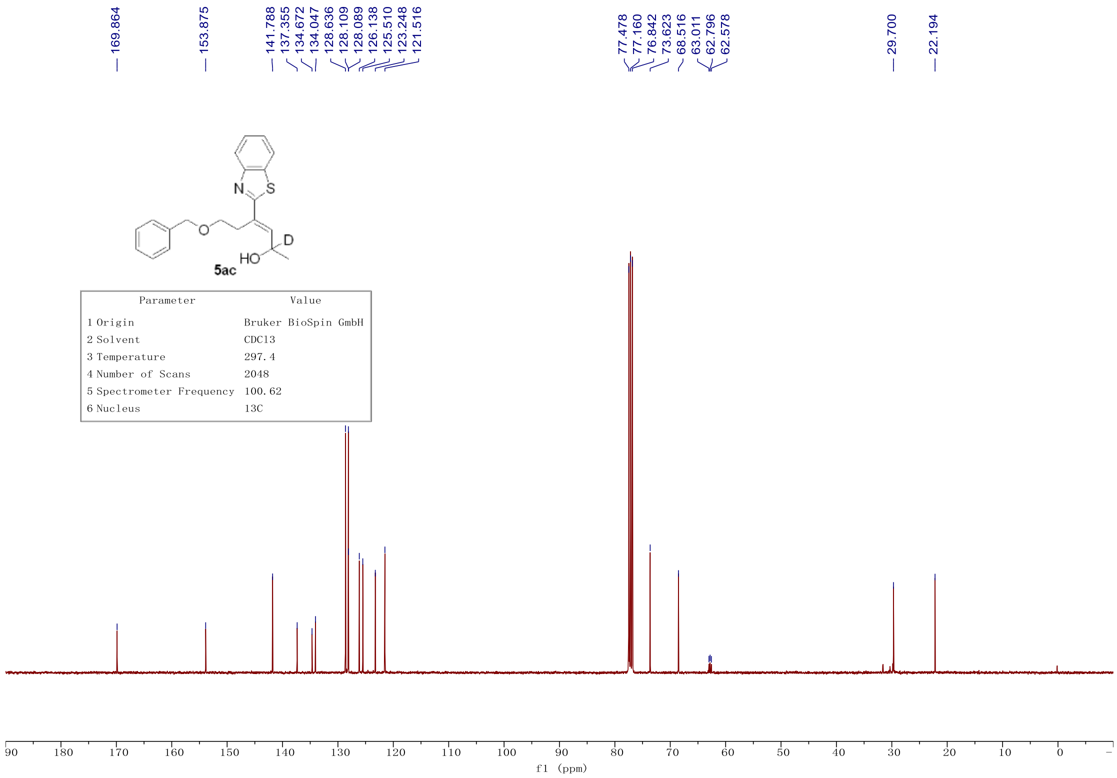

Figure S248. ^{13}C-NMR of **5ac.**

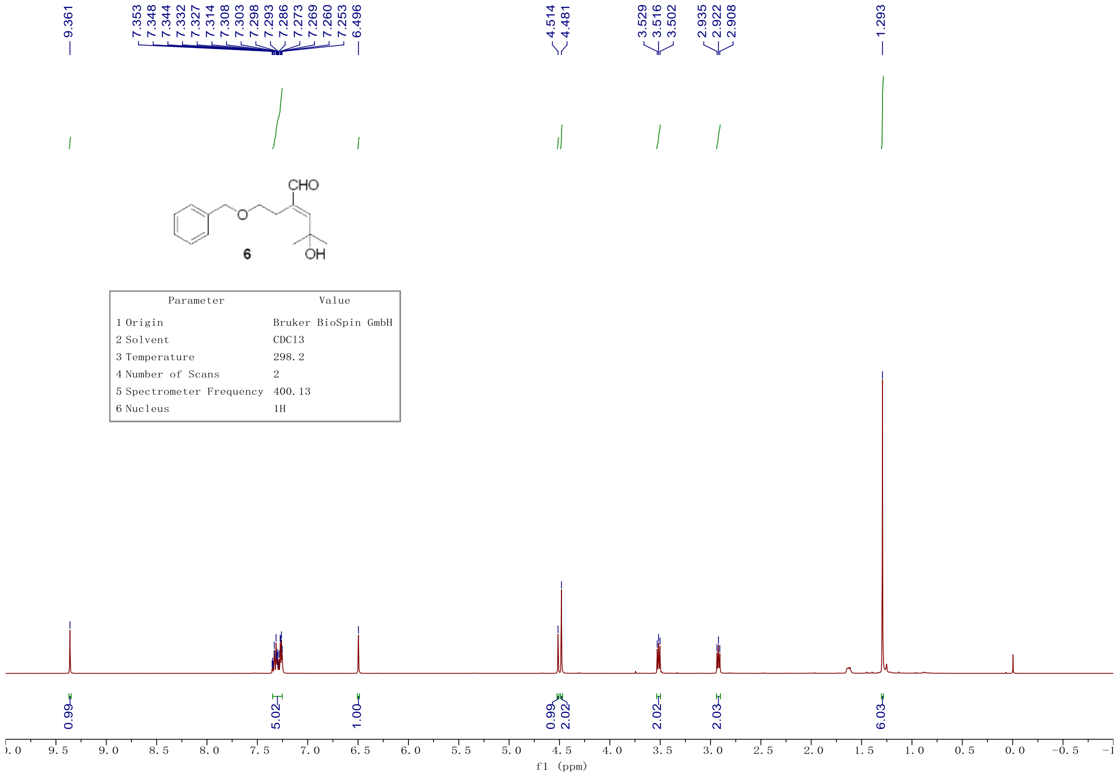

Figure S249. ^{1}H-NMR of **6.**

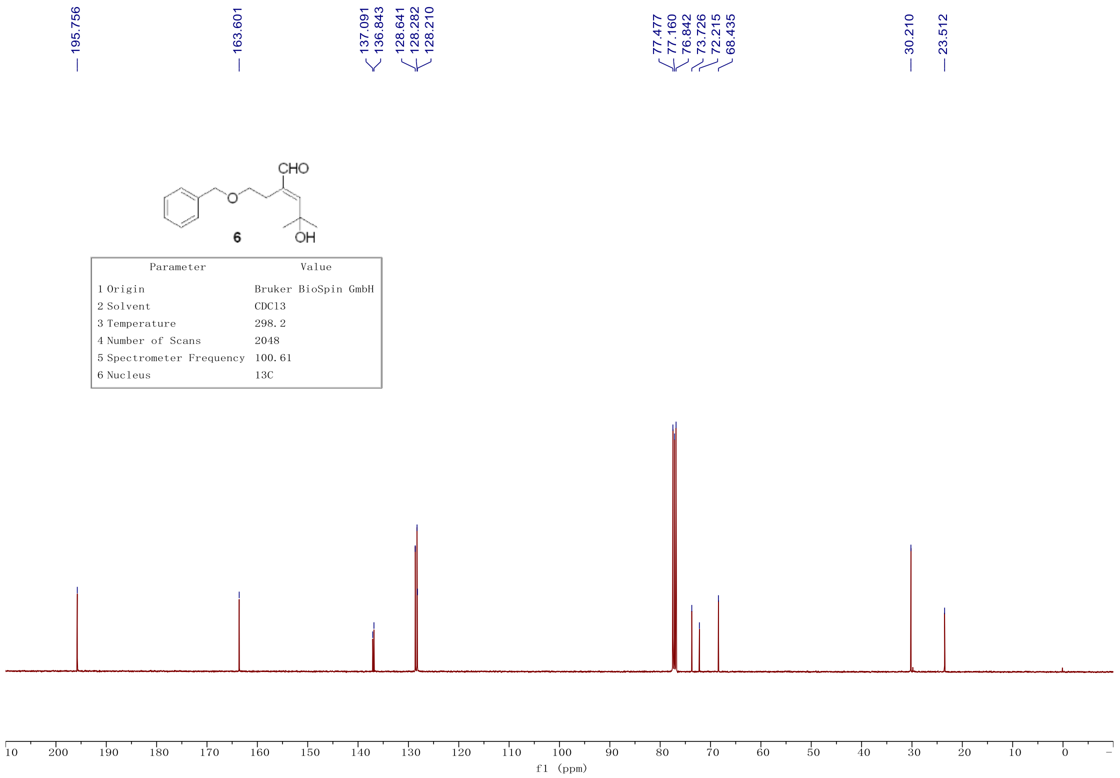

Figure S250. ^{13}C-NMR of **6.**

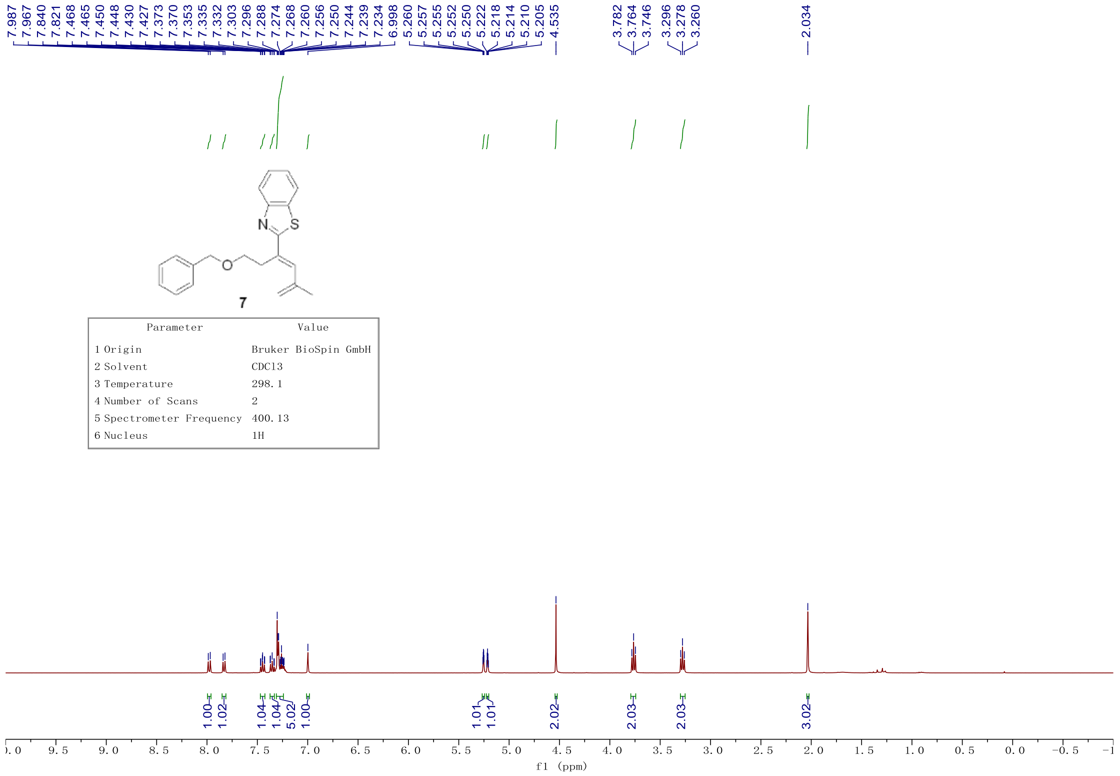

Figure S251. ^{1}H-NMR of **7.**

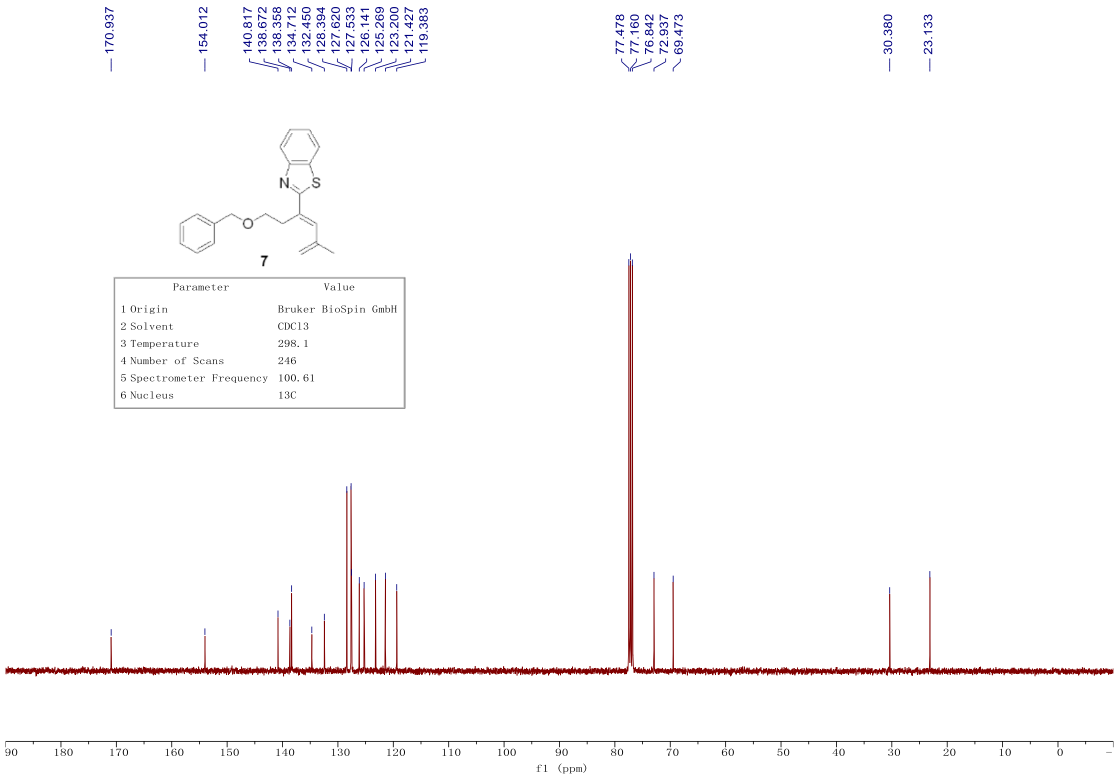

Figure S252. ^{13}C-NMR of **7.**

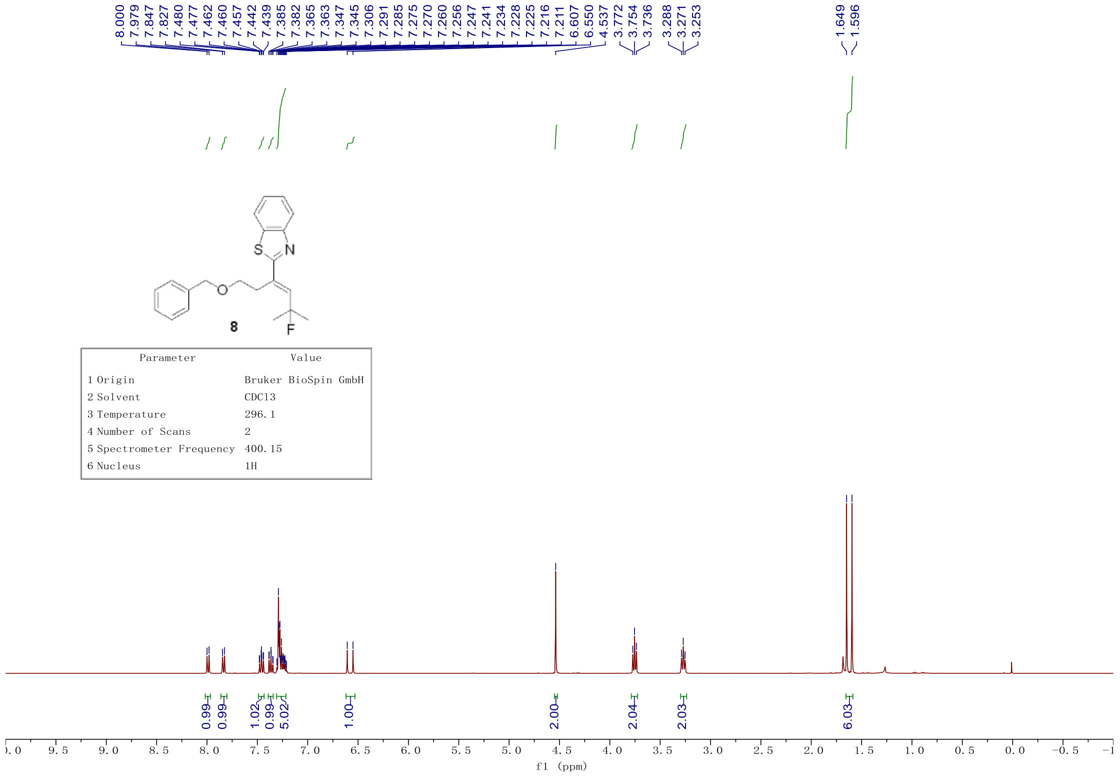

Figure S253. ^{1}H-NMR of **8.**

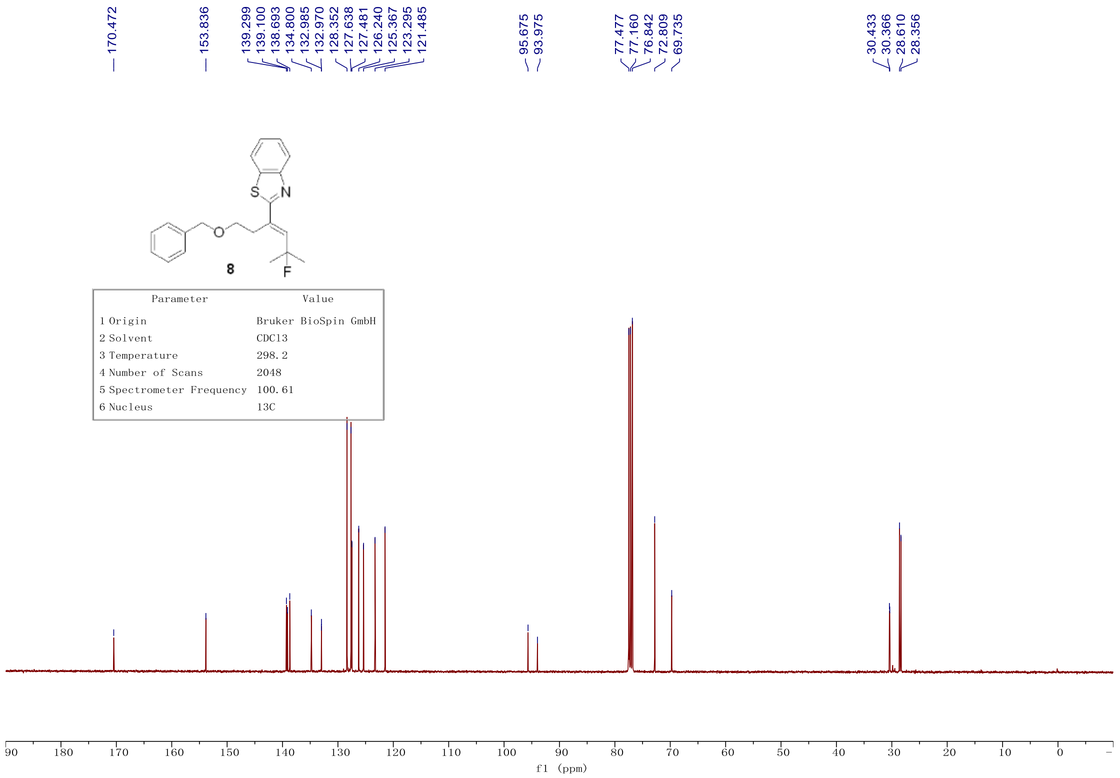

Figure S254. ^{13}C-NMR of **8.**

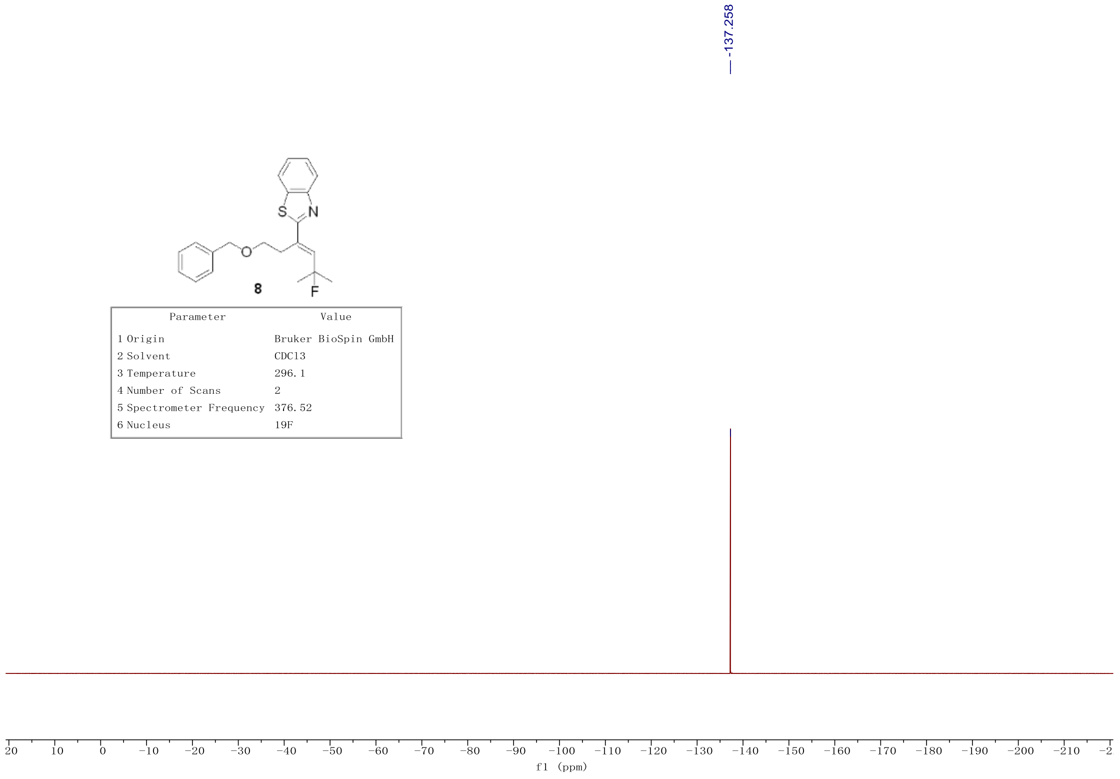

Figure S255. ^{19}F-NMR of **8.**

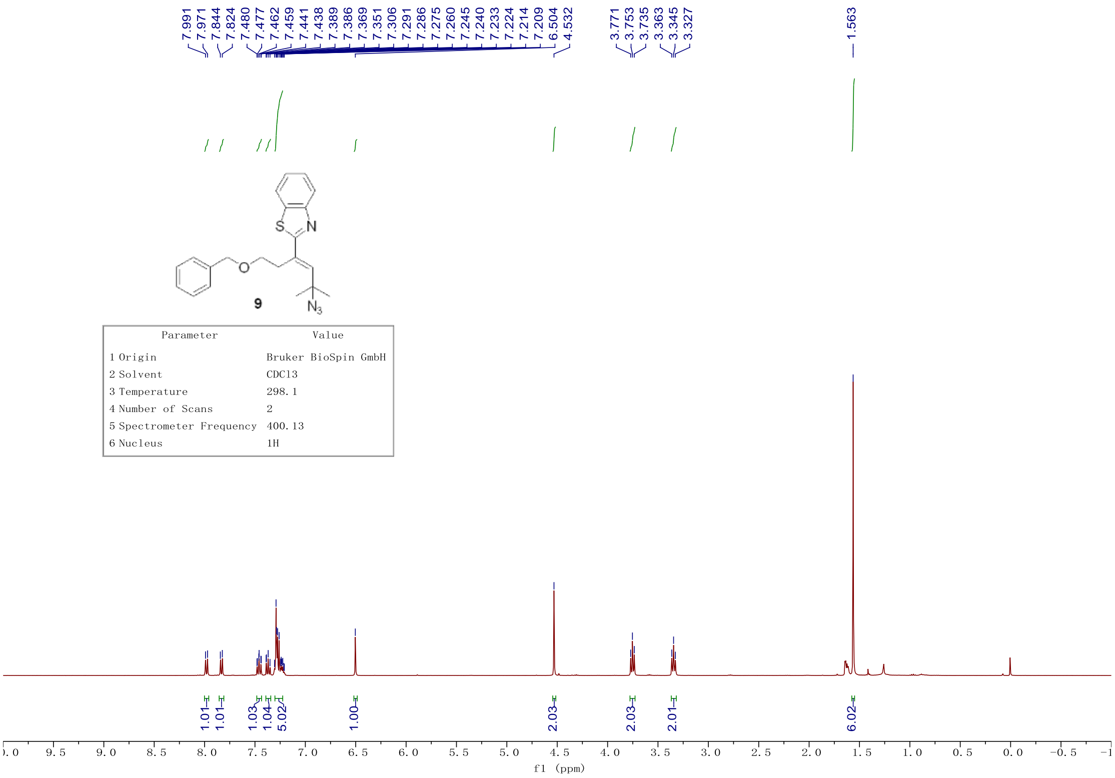

Figure S256. ^{1}H-NMR of **9.**

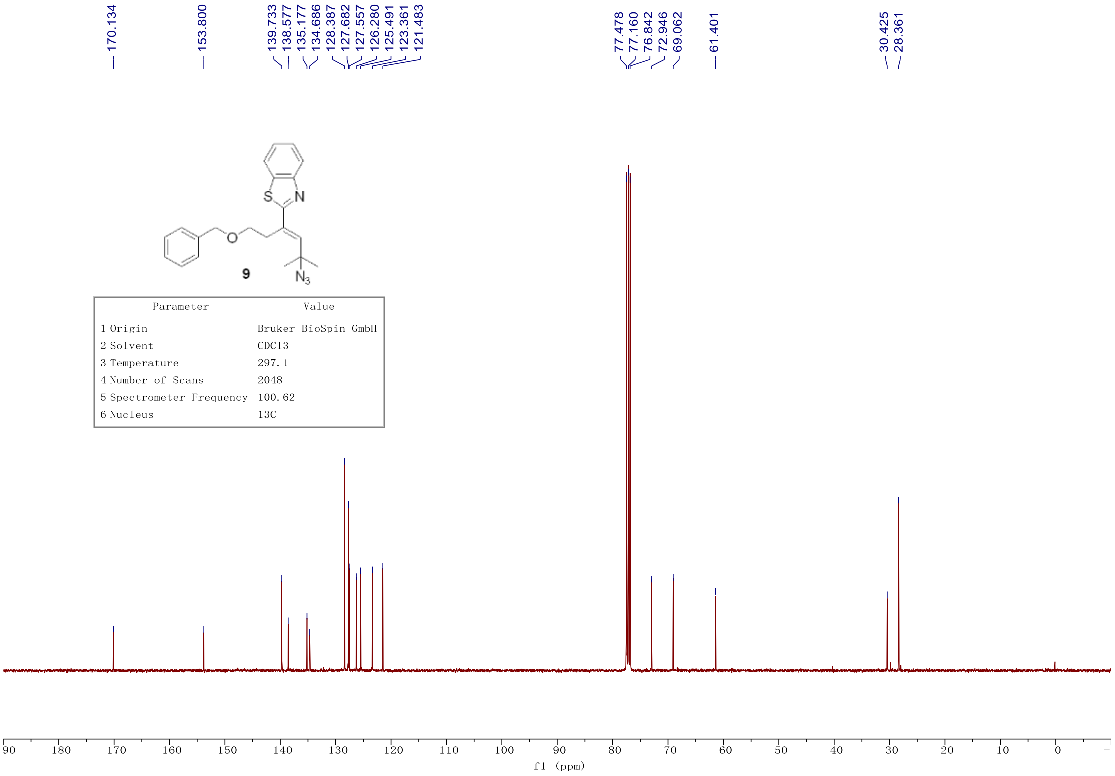

Figure S257. ^{13}C-NMR of **9.**

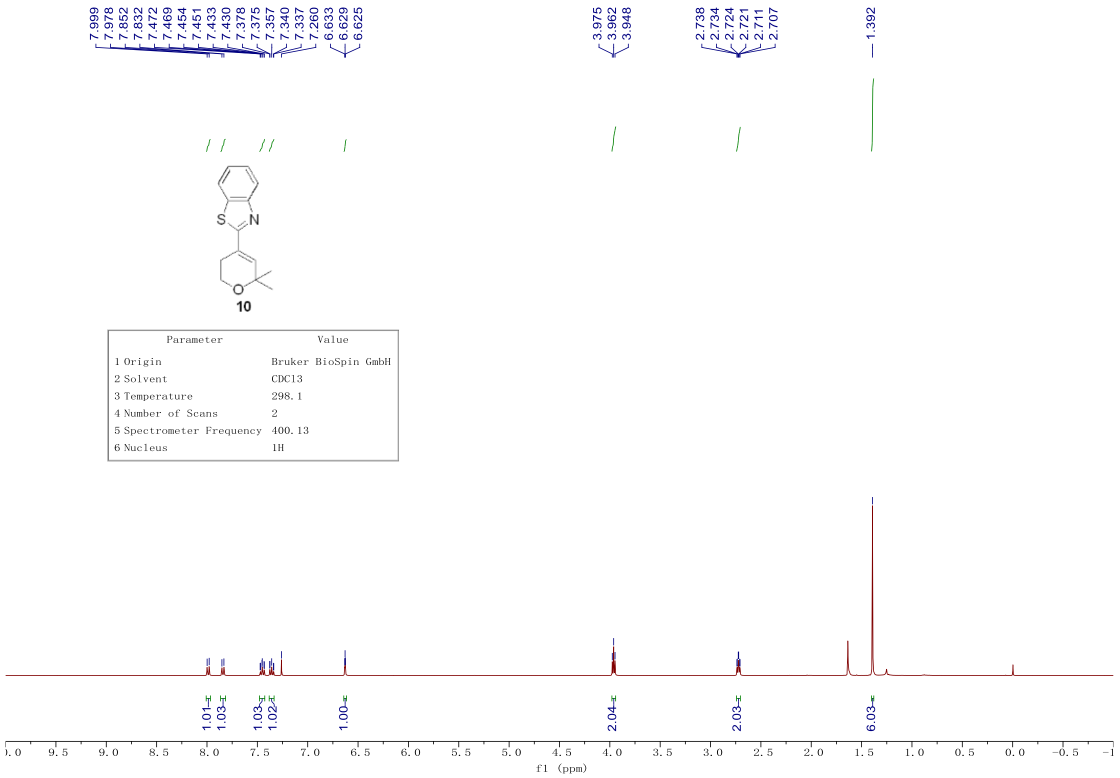

Figure S258. ^{1}H-NMR of **10.**

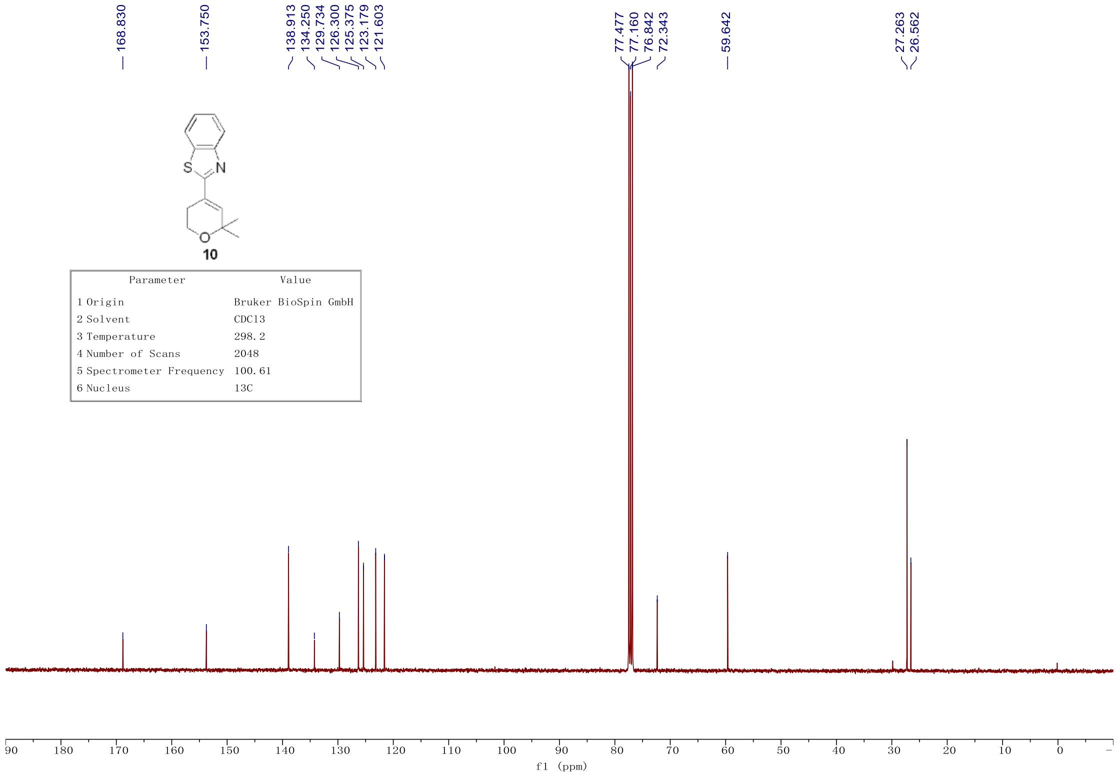

Figure S259. ^{13}C-NMR of **10.**

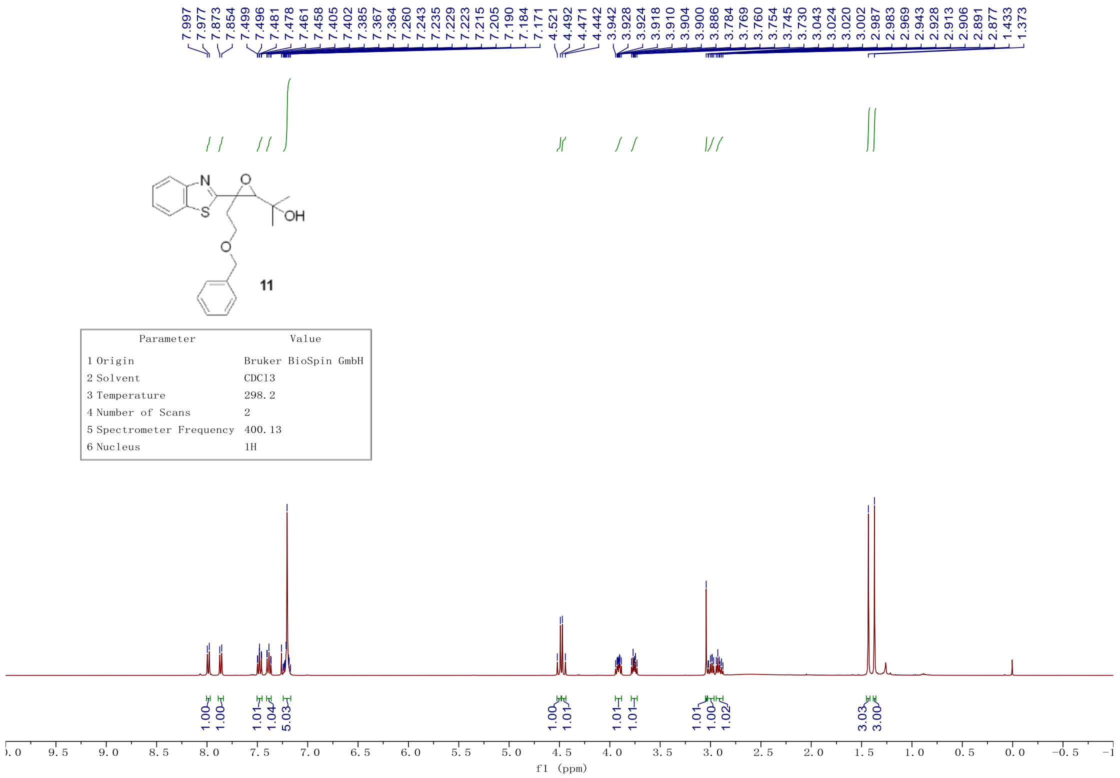

Figure S260. ^{1}H-NMR of **11.**

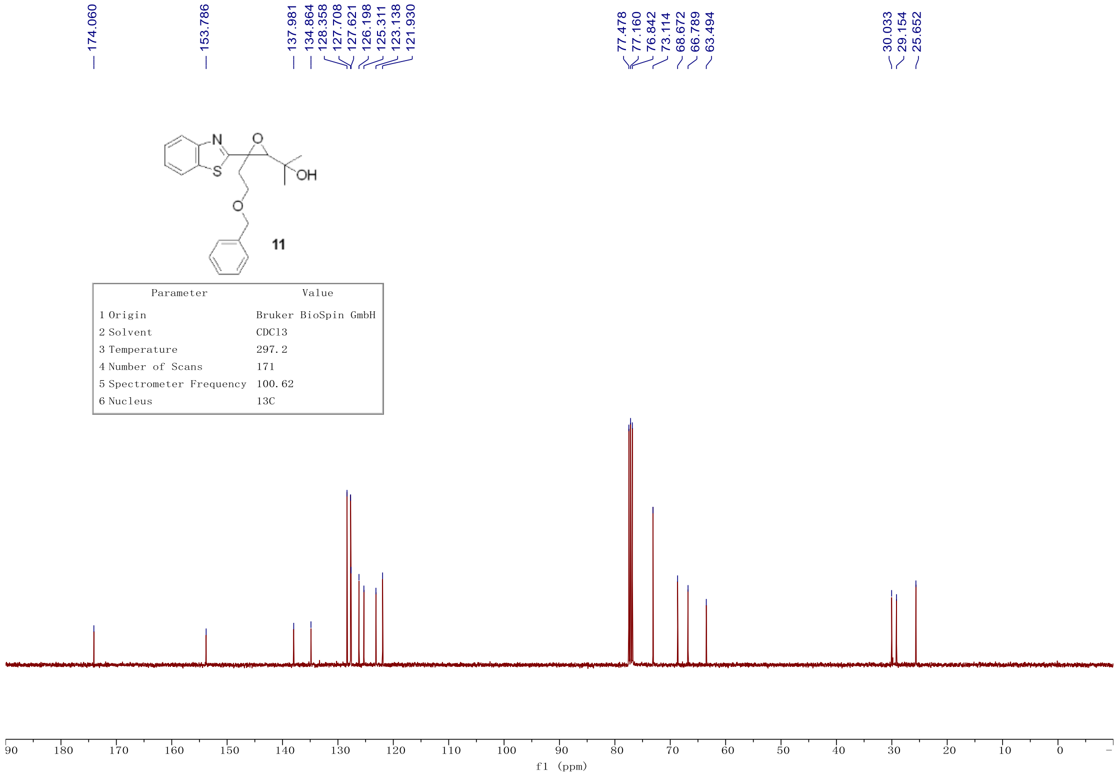

Figure S261. ^{13}C-NMR of **11.**

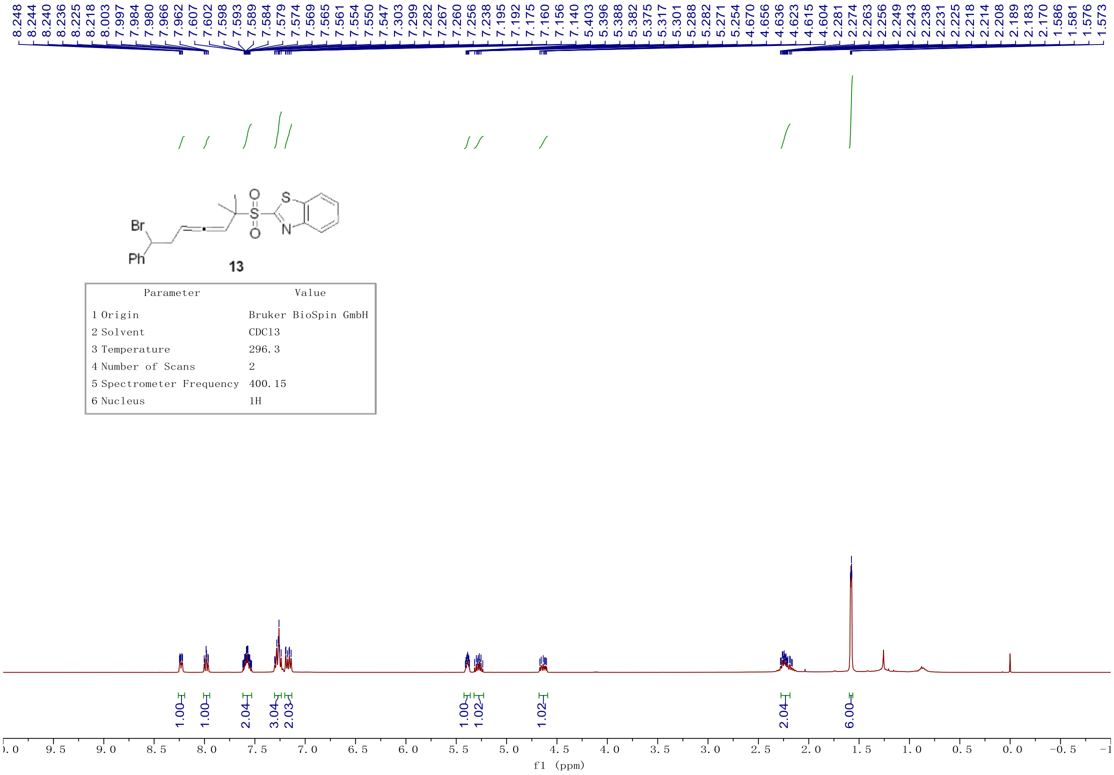

Figure S262. ^{1}H-NMR of **13.**

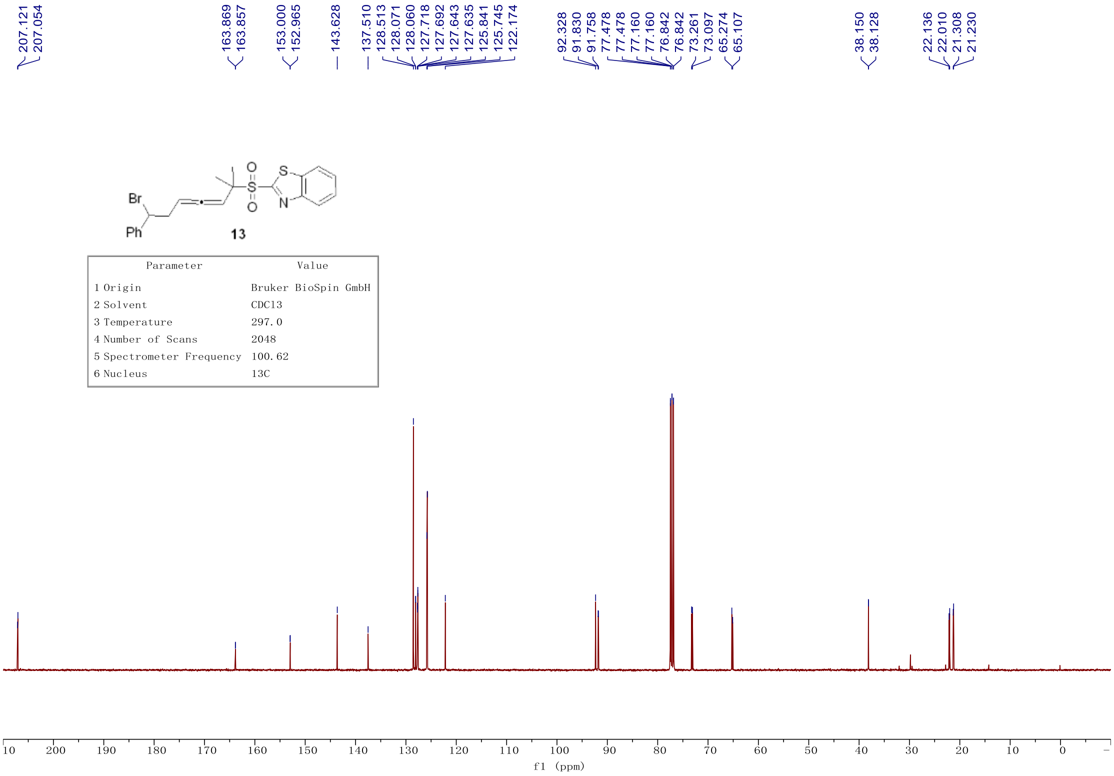

Figure S263. ^{13}C-NMR of **13.**

Reference

1. Lindsay, V. N. G.; Viart, H. M. F.; Sarpong, R. Stereodivergent Intramolecular C(sp3)-H Functionalization of Azavinyl Carbenes: Synthesis of Saturated Heterocycles and Fused N-Heterotricycles. *J. Am. Chem. Soc.* **2015**, *137*, 8368-8371.
2. Liu, Z.; Derosa, J.; Engle, K. M. Palladium(II)-Catalyzed Regioselective syn-Hydroarylation of Disubstituted Alkynes Using a Removable Directing Group. *J. Am. Chem. Soc.* **2016**, *138*, 13076-13081.
3. Yuan, T.; Tang, Q.; Shan, C.; Ye, X.; Wang, J.; Zhao, P.; Wojtas, L.; Hadler, N.; Chen, H.; Shi, X. Alkyne Trifunctionalization via Divergent Gold Catalysis: Combining π-Acid Activation, Vinyl-Gold Addition, and Redox Catalysis. *J. Am. Chem. Soc.* **2021**, *143*, 4074-4082.
4. Ji, K.; Zhang, L. Cyclopropanation of Benzene Rings by Oxidatively Generated α-Oxo Gold Carbene: One-Pot Access to Tetrahydropyranone-Fused Cycloheptatrienes from Propargyl from Propargyl Benzyl Ethers. *Adv. Synth. Catal.* **2018**, *360*, 647-651.
5. Fang, X.; Cacherat, B.; Morandi, B. CO- and HCl-free Synthesis of Acid Chlorides from Unsaturated Hydrocarbons via Shuttle Catalysis. *Nat. Chem.* **2017**, *9*, 1105-1109.
6. Yu, P.; Bismuto, A.; Morandi, B. Iridium-Catalyzed Hydrochlorination and Hydrobromination of Alkynes by Shuttle Catalysis. *Angew. Chem. Int. Ed.* **2020**, *59*, 2904-2910.
7. Wang, F.; Guo, Y.; Zhang, Y.; Tang, P. Silver-Catalyzed Dibromotrifluoromethoxylation of Terminal Alkynes. *ACS Catal.* **2021**, *11*, 3218-3223.
8. Parsons, A. T.; Senecal, T. D.; Buchwald, S. L. Iron(II)-Catalyzed Trifluoromethylation of Potassium Vinyltrifluoroborates. *Angew. Chem. Int. Ed.* **2012**, *51*, 2947-2950.
9. Zelenay, B.; Munton, P.; Tian, X.; Díez-Gonález, S. A Commercially Available and User-Friendly Catalyst for Hydroamination Reactions under Technical Conditions. *Eur. J. Org. Chem.* **2019**, *29*, 4725-4730.
10. Taber, D. F.; Berry, J. F.; Martin, T. J. Convenient Synthetic Route to an Enantiomerically Pure FMOC α-Amino Acid. *J. Org. Chem.* **2008**, *73*, 9334-9339.
11. Huang, Q.; Su, Y. X.; Sun, W.; Hu, M. Y.; Wang, W. N.; Zhu, S. F. Iron-Catalyzed Vinylzincation of Terminal Alkynes. *J. Am. Chem. Soc.* **2022**, *144*, 515-526.
12. Wang, Y.; Scrivener, S. G.; Zuo, X. D.; Wang, R.; Palermo, P. N.; Murphy, E.; Durham, A. C.; Wang, Y. M. Iron-Catalyzed Contrasteric Functionalization of Allenic C(sp^2)-H Bonds: Synthesis of α-Aminoalkyl 1,1-Disubstituted Allenes. *J. Am. Chem. Soc.* **2021**, *143*, 14998-15004.
13. Yu, J.; Zhang, X.; Wu, X.; Liu, T.; Zhang, Z.-Q.; Wu, J.; Zhu, C. Metal-Free Radical Difunctionaization of Ethylene. *Chem* **2023**, *9*, 472-482.
14. Cismesia, M. A.; Yoon, T. P. Characterizing Chain Processes Invisible Light Photoredox Catalysis. *Chem. Sci.* **2015**, *6*, 5426-5434.
15. Quach, L.; Dutta, S.; Pflüger, P. M.; Sandfort, F.; Belotti, P.; Glorius, F. Visible-Light-Initiated Hydrooxygenation of Unactivated Alkenes-A Strategy for Anti-Markovnikov Hydrofunctionalization. *ACS Catal.* **2022**, *12*, 2499-2504.
16. Frisch, M. J.; Trucks, G. W.; Schlegel, H. B.; Scuseria, G. E.; Robb, M. A.; Cheeseman, J. R.; Scalmani, G.; Barone, V.; Mennucci, B.; Petersson, G. A.; Nakatsuji, H.; Caricato, M.; Li, X.; Hratchian, H. P.; Izmaylov, A. F.; Bloino, J.; Zheng, G.; Sonnenberg, J. L.; Hada, M.; Ehara, M.;

Toyota, K.; Fukuda, R.; Hasegawa, J.; Ishida, M.; Nakajima, T.; Honda, Y.; Kitao, O.; Nakai, H.; Vreven, T.; Montgomery, J. A.; Jr, J. E. P.; Ogliaro, F.; Bearpark, M.; Heyd, J. J.; Brothers, E.; Kudin, K. N.; Staroverov, V. N.; Keith, T.; Kobayashi, R.; Normand, J.; Raghavachari, K.; Rendell, A.; Burant, J. C.; Iyengar, S. S.; Tomasi, J.; Cossi, M.; Rega, N.; Millam, J. M.; Klene, M.; Knox, J. E.; Cross, J. B.; Bakken, V.; Adamo, C.; Jaramillo, J.; Gomperts, R.; Stratmann, R. E.; Yazyev, O.; Austin, A. J.; Cammi, R.; Pomelli, C.; Ochterski, J. W.; Martin, R. L.; Morokuma, K.; Zakrzewski, V. G.; Voth, G. A.; Salvador, P.; Dannenberg, J. J.; Dapprich, S.; Daniels, A. D.; Farkas, O.; Foresman, J. B.; Ortiz, J. V.; Cioslowski, J.; Fox, D. J. Gaussian 09, Revision D.01, Gaussian, Inc., Wallingford CT, 2013.

17. Zhao, Y.; Truhlar, D. G. Density Functional with Broad Applicability in Chemistry. *Acc. Chem. Res.* **2008**, *41*, 157-167.
18. Zhao, Y.; Truhlar, D. G. Applications and Validations of the Minnesota Density Functionals. *Chem. Phys. Lett.* **2011**, *502*, 1-13.
19. Wang, M.; Li, M.; Yang, S.; Xue, X.-S.; Wu, X.; Zhu, C. Radical-Mediated C-C Cleavage of Unstrained Cycloketones and DFT Study for Unusual Regioselectivity. *Nat. Commun.* **2020**, *11*, 672-679.
20. Marenich, A. V.; Cramer, C. J.; Truhlar, D. G. Universal Solvation Model Based on Solute Electron Density and on a Continuum Model of the Solvent Defined by the Bulk Dielectric Constant and Atomic Surface Tensions. *J. Phys. Chem. B* **2009**, *113*, 6378-6396.
